# The MEME Suite

**DOI:** 10.1093/nar/gkv416

**Published:** 2015-05-07

**Authors:** Timothy L. Bailey, James Johnson, Charles E. Grant, William S. Noble

**Affiliations:** 1 Institute for Molecular Bioscience, The University of Queensland, Brisbane 4072, Queensland, Australia; 2 Department of Genome Sciences, Department of Computer Science and Engineering, University of Washington, Seattle, WA 98109, USA

## Abstract

The MEME Suite is a powerful, integrated set of web-based tools for studying sequence motifs in proteins, DNA and RNA. Such motifs encode many biological functions, and their detection and characterization is important in the study of molecular interactions in the cell, including the regulation of gene expression. Since the previous description of the MEME Suite in the 2009 *Nucleic Acids Research Web Server Issue*, we have added six new tools. Here we describe the capabilities of all the tools within the suite, give advice on their best use and provide several case studies to illustrate how to combine the results of various MEME Suite tools for successful motif-based analyses. The MEME Suite is freely available for academic use at http://meme-suite.org, and source code is also available for download and local installation.

## INTRODUCTION

A DNA, RNA or protein sequence *motif* is a short pattern that is conserved by evolution. In DNA, a motif may correspond to a protein-binding site; in proteins, a motif may correspond to the active site of an enzyme or a structural unit necessary for proper folding of the protein. Thus, sequence motifs are one of the basic functional units of molecular evolution. Consequently, identifying and understanding these motifs is fundamental to building models of cellular processes at the molecular scale and to understanding the mechanisms of human disease.

The MEME Suite is a software toolkit for performing motif-based sequence analysis, which is valuable in a wide variety of scientific contexts. The MEME Suite software has played an important role in the study of biological processes involving DNA, RNA and proteins in over 9800 published studies. With the advent of high-throughput genomics and proteomics, the importance of motif analysis continues to increase. The MEME Suite has been used to make a wide variety of biological discoveries, examples of which are listed in Supplementary Table S1.

**Figure 1.**
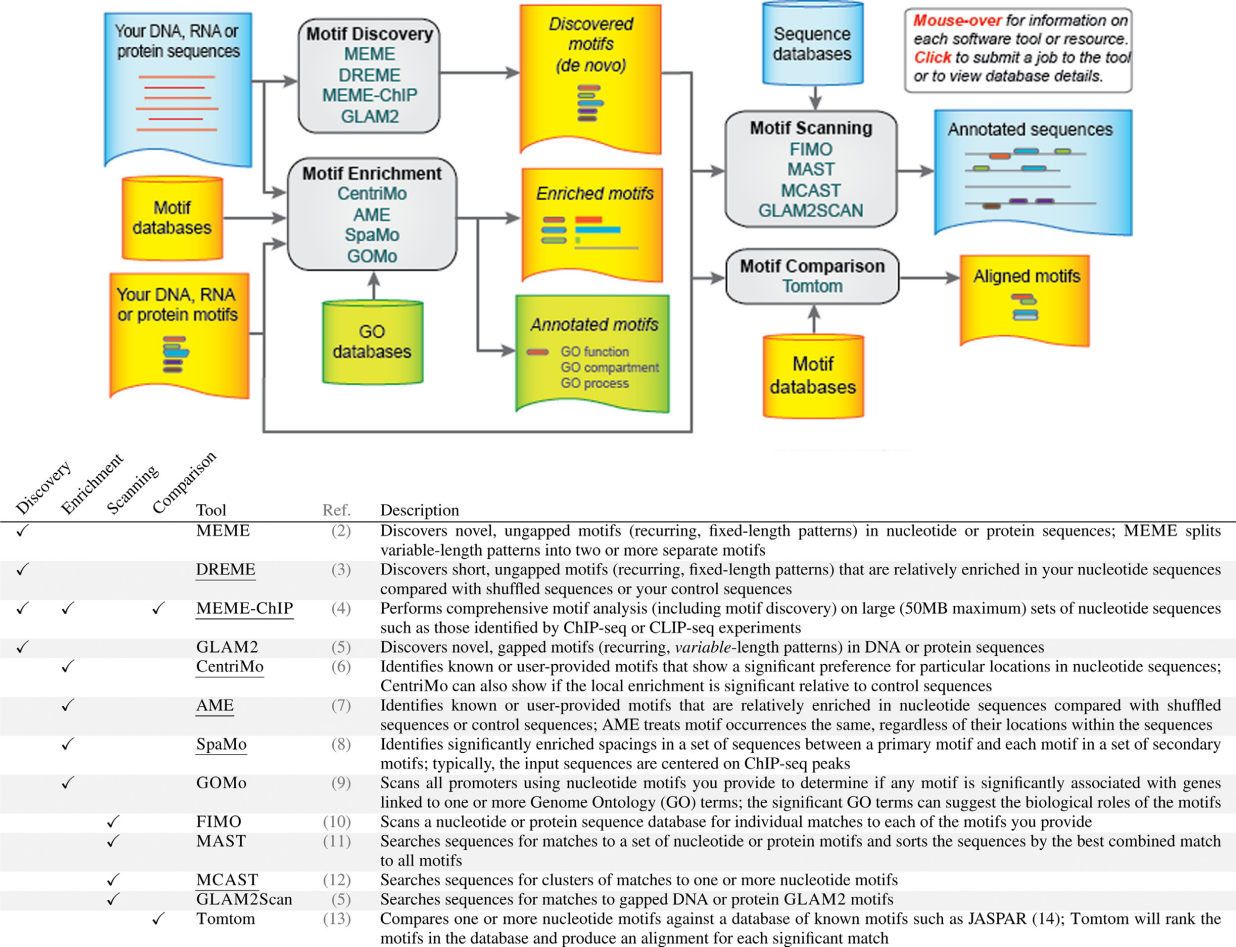
Overview of the integrated tools in the MEME Suite. Tools added since the MEME Suite web server was last described (15) are underlined.

The web-based version of the MEME Suite comprises an integrated set of tools and databases for performing motif-based sequence analyses (Figure 1). The core of the suite is the meme motif discovery algorithm, which finds motifs in unaligned collections of DNA, RNA and protein sequences (1). Initially described in 1994, meme has been continually maintained and improved in the ensuing 20 years. The meme web server came online in 1996 and is now widely used, with almost 20 000 unique users in 2014 alone.

### Using the MEME Suite

The web-based version of the MEME Suite includes 13 tools (1,2,3,4,5,6,7,8,9,10,11,12,13) for performing motif discovery, motif enrichment analysis, motif scanning and motif–motif comparisons (Figure 1). Six of these tools—DREME (3), MEME-ChIP (4), CentriMo (6), AME (7), SpaMo (8) and MCAST (12)—were developed or given web interfaces since the last publication describing the MEME Suite (15). For motif discovery and motif enrichment analyses, the user provides a set of unaligned DNA, RNA or protein sequences (Figure 1, upper left). Typically, these sequences might be ChIP-seq peak regions, cross-linking sites from a CLIP-seq experiment, promoters of co-expressed genes or proteins sharing a common function such as being modified by the same kinase.

Motif discovery finds *de novo* motifs in the user-provided sequences (Figure 1, upper middle). These motifs can then be input directly to the motif scanning and motif comparison tools of the MEME Suite (Figure 1, right) to identify other proteins or genomic sequences that may contain the discovered motifs, or to determine if the motifs are similar to previously studied motifs. The MEME Suite provides a large number of proteomic and genomic sequence databases (Figure 1, top right) for motif scanning and many motif databases for motif comparison (Figure 1, bottom right).

The four different motif discovery algorithms suit different purposes. meme is a general purpose motif discovery algorithm for both nucleotide and peptide motifs, but is less sensitive than DREME for finding short nucleotide motifs. Neither meme nor DREME allows insertions or deletions in the motifs they find, but glam2 does. Finally, meme-chip is adapted to very large datasets that cannot be handled by meme, and it actually performs motif discovery, motif enrichment and motif comparison on its input sequences, producing a fully integrated report. A comprehensive protocol for using meme-chip has recently been published (16).

Motif enrichment analysis tests *known* motifs for enrichment in a set of user-provided sequences. This approach is more sensitive than motif discovery, but motif enrichment analysis is limited to detecting enrichment of motifs contained in the database of motifs selected as input (Figure 1, middle left). Sensitivity is highest with CentriMo, which leverages the extra information sometimes contained in the position of the motif within each of the input sequences. The sequences input to CentriMo must all have the same length, which is not the case with the less sensitive motif enrichment algorithm AME. The SpaMo algorithm looks for preferred spacings in the input sequences between two motifs, rather than enrichment of a single motif. Finally, the gomo algorithm performs motif scanning of promoter sequences followed by a Gene Ontology enrichment analysis, so it is often applied to *de novo* discovered motifs to identify their possible biological functions.

Motif scanning involves identifying locations of occurrences of a given set of motifs within a given set of sequences. As with motif discovery, the four motif scanning tools suit distinct purposes (Figure 1, middle right). The fimo algorithm identifies all individual motif occurrences and is the method of choice for scanning genomes. Its output can be uploaded to the UCSC genome browser for viewing. In contrast, the mast algorithm is sequence oriented and assigns each sequence in the selected database a score based on how well it matches all of the motifs input by the user. Thus, mast is most suited to scanning short sequences such as proteins or promoters. The mcast algorithm scans genomes for clusters containing multiple matches to any or all of the motifs in its input. It was designed for detecting *cis*-regulatory modules (CRMs) bound by a known set of transcription factors. Finally, the glam2scan algorithm is similar to fimo but is designed to accept glam2 motifs; hence, the resulting motif matches may contain insertions and deletions.

The MEME Suite's motif comparison tool, Tomtom, allows the user to compare motifs discovered by the suite, by other tools, or taken from the literature to all of the motifs in a selected database of motifs (Figure 1, bottom right). For example, Tomtom can be used to determine if a reported consensus sequence for a transcription factor motif matches any known motifs in databases of motifs produced using SELEX or protein-binding microarrays. Tomtom aligns each input motif with each motif in the selected database and reports the most similar pairs, along with estimates of the statistical significance of each match.

Users can also use the MEME Suite with motifs generated by other motif analysis tools or taken directly from the literature (Figure 1, bottom left). As described in the next section, the web server allows user-specified motifs to be input in many convenient formats. Although omitted from Figure 1 for clarity, the motif scanning tools also allow for user-provided sequences, and the motif comparison tool allows for uploading user-provided motif databases.

### The MEME Suite web interface

**Figure 2.**
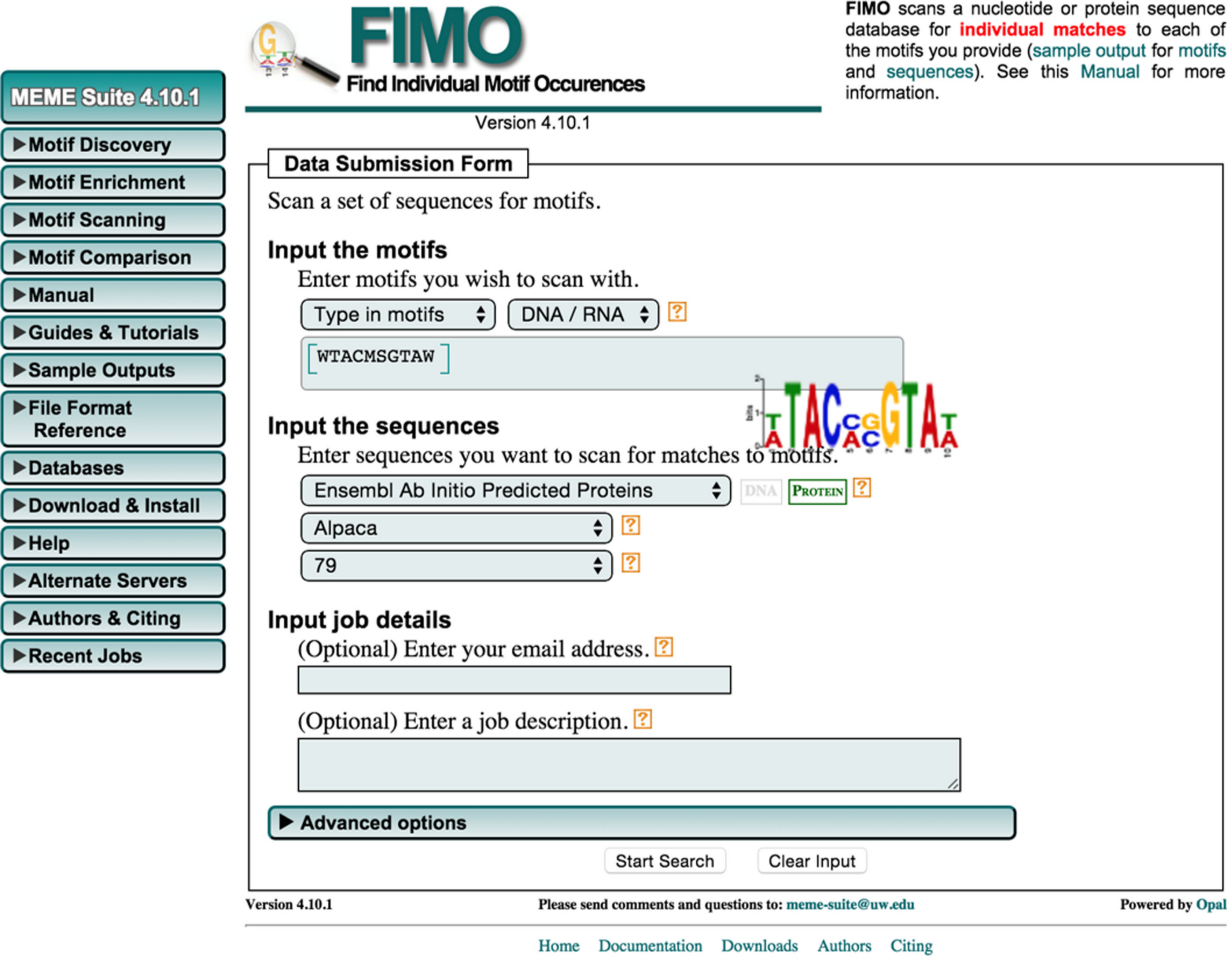
fimo input form showing interactive display of motif logo for typed in motifs.

The MEME Suite provides a set of consistent input forms for its 13 web-based tools (e.g. Figure 2). For each input field, ‘help bubbles’ provide an explanation of what information is required, how you can provide it and, in many cases, examples of valid input. You can view a help bubble by clicking on the question mark ‘?’ situated to the right of the input field. Within each of the groupings of MEME Suite motif analysis tools (discovery, enrichment, scanning, comparison), the user interfaces are consistent and flexible. For example, you can input sequences required by the first three groupings either by selecting a file for upload or by typing (or cut-and-paste). As a second example of consistency and versatility, all the tools that accept motifs as input (for enrichment, scanning or comparison) allow you to upload them by selecting a file name or by typing or cutting-and-pasting one or more motifs in any of a number of different formats.

When you enter motifs by typing, the web interface automatically detects whether you are specifying a motif as one or more sequence sites (e.g. a consensus sequence or a multiple alignment) or as a count or probability matrix and interactively displays a logo for the motif (Figure 2). Typed sequence sites allow the entire IUPAC alphabet (including ambiguous characters) for DNA and proteins. If you enter numbers instead of letters, then the web interface assumes you are entering either a count matrix or a probability matrix, and automatically determines whether rows correspond to positions in the motif or to letters in the alphabet. As you enter motifs by typing, the web interface reports errors such as unsupported characters or if the sequence sites are of inconsistent lengths. Multiple motifs may be specified simply by separating them by a blank line. All typed motifs are automatically converted to motifs in the meme motif format. Note, however, that glam2scan does not support typed motifs because it uses a different motif format.

The MEME Suite website also provides access to a large number of motif and sequence databases for use in your analyses. For example, you can select from among 38 different motif databases for use with the motif enrichment and comparison tools. These databases include *in vitro* compendia based on SELEX or protein-binding microarrays, as well as human-curated compendia of *in vivo* or *in vitro* motifs such as JASPAR (14). All of these databases are also available for you to download and use on your own computer via the ‘Download & Install’ menu on the MEME Suite website.

Similarly, you can select from a large menu of DNA and protein databases for use with the motif scanning tools. These include protein and genomic databases from Ensembl and GenBank, genomes from UCSC, as well as sets of promoters (upstream regions) for many organisms. To specify which sequence database you wish to search (Figure 2), you first select the database category (e.g. ‘Ensembl Ab Initio Predicted proteins’), then the organism, e.g. ‘Human’, followed by the version of the database (e.g. ‘75’).

After you submit a job to a MEME Suite tool, you will be taken to a status page showing the job's progress. The ‘Recent Jobs’ menu item on the left of most MEME Suite web-server pages will allow you to access this status page as long as your current browser session is active. If you plan to exit the browser or log off before your job completes, you should either bookmark the status page or you can choose to provide an (optional) email address when you submit jobs. Once your job completes, the status page will contain links to your results in HTML and other formats. Your results will be kept on our server for only a few days, so you should download them (using your browser's ‘File/Save’ feature) if you wish to save them indefinitely.

### User support for the MEME Suite

User support for the MEME Suite includes extensive online documentation, an online user forum and email support. All user support is accessible via the menu on the left side of all MEME Suite web pages (e.g. Figure 2). Clicking the ‘Manual’ tab of the menu reveals links to detailed information on each of the web-based tools. Clicking the ‘OVERVIEW’ link (located at the top of the list of tools in the ‘Manual’ sub-menu) takes you to a page describing the entire suite, including the many supplementary tools for manipulating sequences and motifs available when you download and install the meme suite on your own computer.

Additional information is provided for some of the tools under the ‘Guides & Tutorial’ tab (e.g. Figure 2, left). The ‘Sample Outputs’ tab reveals links to example outputs from each of the web-based tools. Viewing these outputs is extremely useful for gaining an understanding of the capabilities of the individual tools and of their suitability to any particular task.

Advanced use of the MEME Suite sometimes involves creating custom motif and sequence files. Details on the relevant file formats is provided under the ‘File Format Reference’ tab of the main menu. Finally, links to the user ‘Q&A’ forum and for emailing the webmaster or developers are shown by clicking the ‘Help’ tab. The ‘Q&A’ forum is a very useful source of answers to frequently asked questions and is constantly updated.

### Comparison with other motif analysis tools

Although many motif discovery and search tools have been described in the scientific literature, the MEME Suite is unique in terms of its broad functionality and consistent reporting of statistical significance for all of its outputs (Table 1). The MEME Suite provides motif discovery algorithms using both probabilistic (meme) and discrete models (DREME), which have complementary strengths. It also allows discovery of motifs with arbitrary insertions and deletions (glam2), which no other web-based tools do. Many other tool suites are DNA-only, but the MEME Suite supports motif discovery in and motif scanning of DNA, RNA and protein sequences. The meme, fimo and mcast algorithms also allow the use of sophisticated probabilistic priors for improving motif discovery and search with additional context-specific information (26–32). In addition to motif discovery, the MEME Suite provides tools for scanning sequences for matches to motifs (mast, fimo and glam2scan), scanning for clusters of motifs (mcast), comparing motifs to known motifs (Tomtom), finding preferred spacings between DNA motifs (SpaMo), predicting the biological roles of DNA motifs (gomo), measuring the positional enrichment of sequences for known DNA motifs (CentriMo), and analyzing ChIP-seq and other large DNA datasets (meme-chip). We are aware of no existing server that provides anything close to the MEME Suite's breadth of functionality.

**Table 1.** MEME Suite capabilities

Capability	meme Suite (17)	Consensus (18)	Gibbs Sampler (19)	RSAT (20)	Trawler (21)	W-ChIPMotifs (22)	MoDTools (23)	YMF 3.0 (24)	XXmotif (25)
DNA motif discovery	meme	✓	✓	✓	✓	✓	✓	✓	✓
Protein motif discovery	meme	✓	✓						
Probabilistic motif discovery	meme	✓	✓	✓		✓			✓
Discrete motif discovery	DREME			✓	✓		✓		
Arbitrary gaps in motifs	glam2, glam2scan			✓					
Positionally constrained motifs	meme			✓					
Discriminative PWM motif discovery	meme					✓			✓
Motif scanning	fimo, mcast, glam2scan	✓	✓	✓					✓
Motif enrichment analysis	CentriMo, AME, SpaMo, gomo			✓			✓		
Motif comparison	Tomtom			✓					
Motif cluster & spacing analysis	SpaMo								
Motif functional role analysis	gomo								
ChIP-seq analysis	meme-chip			✓	✓	✓	✓		✓

Comparison of MEME Suite capabilities with other web-based tools. Note that W-ChIPMotifs uses MEME for motif discovery. URLs of the servers are provided in the bibliography. The table does not include servers that require additional inputs, such as aligned orthologs, phylogenetic trees, microarray expression profiles or ChIP-chip data.

## CASE STUDY 1: DISCOVERING A HOST-TARGETING SIGNAL IN MALARIA

This case study uses three MEME Suite tools, meme, mast and fimo. It begins with a small number of protein sequences and reveals the identity and widespread presence of a protein motif in the genome of *Plasmodium falciparum*, the parasite responsible for the most lethal form of malaria. This case study describes the actual approach taken by Hiller *et al*. (33) to identify this motif. The authors subsequently validated the importance of this motif using functional studies, implicating the motif in targeting *P. falciparum* virulence proteins to red blood cells.

Hiller *et al*. first determined that regions of ∼40 amino acids (aa) in five *P. falciparum* proteins were necessary and sufficient for the export of those proteins to the cytoplasm during the erythrocyte stage of the parasite life cycle (34). From each of those five proteins, the authors extracted a 100 aa region containing the putative 40 aa signal regions in FASTA format to prepare an input file for meme. (All input files and the outputs generated for this case study are contained in supplementary file ‘case1.zip’.)

### Discovering a *P. falciparum* protein motif using meme.

**Figure 3.**
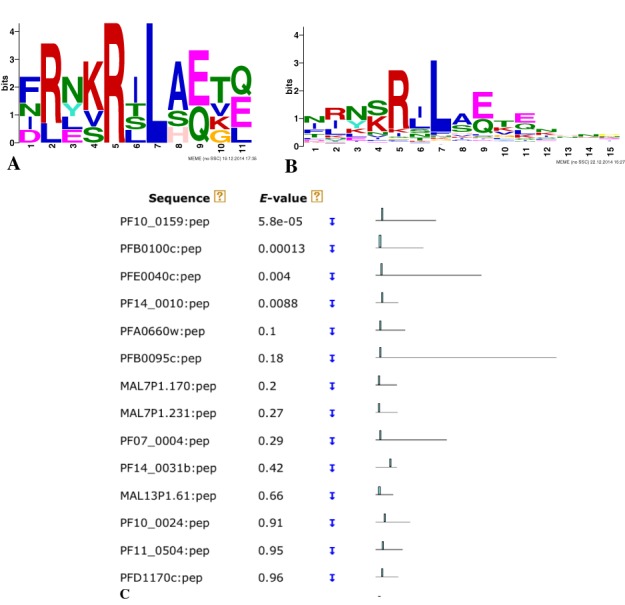
Secretory signal in *P. falciparum* proteins discovered using meme and mast. (**A**) The logo of the motif discovered by meme in the N-terminal regions of of five proteins known to be secreted by *P. falciparum*. (**B**) The logo of the motif discovered by meme in the 150 aa N-terminal regions of 184 proteins where fimo reports a match to the first motif. (**C**) A portion of the mast ‘Block Diagram’ output illustrating the relative positions of matches to the first motif.

To recreate their analysis, download the *P. falciparum* proteome from Ensembl (http://protists.ensembl.org/info/website/ftp/index.html) and extract the specified 100 aa regions of the five proteins using a BED file and the fastaFromBed utility in BedTools. (The BED file and all other inputs and outputs of our analysis are available in the Supplementary Data.) To run meme, on the meme web page click ‘Browse’ next to the menu under the heading ‘Input the primary sequences’ and upload the file ‘Hiller2004.1.fa’ from the supplementary file ‘case1.zip’. Then, under ‘Select the site distribution’ select ‘one occurrence per sequence’ and under ‘Select the number of motifs’ enter ‘1’. Click on the ‘Advanced options’ tab and under ‘How wide can motifs be?’ enter ‘11’ in both the ‘Minimum width:’ and ‘Maximum width:’ fields. Then click ‘Start Search’ to submit the job.

meme returns the motif reported by Hiller *et al*. (33) (Figure 3A). meme reports that this motif is *not* statistically significant (*E* = 4.3), but meme *E*-values tend to be very conservative estimates of true significance when the input dataset has so few sequences (five), and this is indeed a biologically functional motif.

### Observing a protein motif's positional preference using mast.

Some evidence of the biological significance of the motif is provided by the fact that it is found primarily in the N-terminal region of many other *P. falciparum* proteins. To observe this, use mast to scan the *P. falciparum* proteome with the motif. You can submit the motif directly to mast from the HTML meme output page. To the right of the motif logo, click on the arrow below the heading ‘Submit/Download’. Then click the button to the left of ‘MAST’ in the menu that appears and then click ‘Submit’. This procedure will bring you to the mast submission form with the motif already loaded, as indicated by the words ‘Submitted motifs’ in the ‘Input the motifs’ menu. Click the first box under ‘Input the sequences’ and choose ‘Upload sequences’. Then click ‘Browse’ and upload the *P. falciparum* proteome from the supplementary file ‘case1.zip’. Click the ‘Advanced options’ tab and then enter ‘250’ in the box under ‘Set a sequence display threshold’. Enter your (optional) email address to receive a link to the results. Then click the ‘Start Search’ button located at the bottom of the page.

mast identifies 264 proteins with the motif (*E*-value <250), and in the vast majority of these proteins the motif is very near the N-terminus, suggesting its role as a secretory signal (Figure 3C). (The block diagram output feature of mast makes it especially useful for visualizing the relative locations of motifs within protein sequences.) To prove the role of this motif, Hiller *et al*. (33) selected candidate proteins from the mast output and showed that they are exported into the cytoplasm of the host erythrocyte using green fluorescent protein (GFP)-fused constructs. For one protein predicted by mast to contain the meme motif, the authors also showed that export was blocked by replacing the core motif region with a different sequence (RILKQLE replaced by LNAKALA).

### Iterative motif refinement with fimo and meme.

The motif shown in Figure 3A is derived from only five proteins, yet versions of it appear in the N-terminal regions of many *P. falciparum* proteins. To refine this motif, Hiller *et al*. (33) applied meme and mast iteratively. For this purpose, it is actually more convenient to scan the proteome with the motif using fimo and then use fimo's text output to get the IDs of *P. falciparum* proteins where the motif occurs in the first 150 aa. We describe how to do this task if you have a Mac or Linux computer.

As above, you can submit the motif directly to fimo from the HTML meme output page. To the right of the motif logo, click on the arrow below the heading ‘Submit/Download’ and then click ‘Submit’. This will bring you to the fimo submission form. Click the first box under ‘Input the sequences’ and choose ‘Upload sequences’. Then click ‘Browse’ and upload the *P. falciparum* proteome from the supplementary file ‘case1.zip’. Enter your (optional) email address to receive a link to the results. Then click the ‘Start Search’ button located at the bottom of the page.

When fimo is finished, download the ‘FIMO plain text output’ file to your computer as a file ‘fimo.txt’. In a terminal window, extract the sequence IDs from the fimo output using the ‘awk’ and ‘sort’ utilities, fetch their 150 aa N-terminal regions using the FASTA utilities provided by the MEME Suite (and in supplementary file ‘case1.zip’ in case you do not have the MEME Suite installed):

**Figure:**
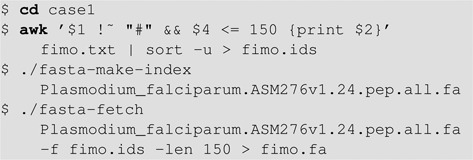


Next, run meme on the resulting expanded set of N-terminal regions. On the meme web page click ‘Browse’ next to the menu under the heading ‘Input the primary sequences’ and upload the file ‘fimo.fa’ that you just created. Then, under ‘Select the site distribution’ select ‘one occurrence per sequence’, and then click ‘Start Search’. When the submitted job completes, the file ‘MEME html output’ file will contain the more general motif whose logo is shown in Figure 3B, which is highly similar to the ‘vacuolar protein export’ motif reported by Hiller *et al*. (33).

## CASE STUDY 2: FINDING A PROTEIN INTERACTION MOTIF

The centromere is a protein complex that plays a crucial functional role, directing the assembly of microtubule-attachment sites, called kinetichores, that allow chromosome segregation on the mitotic spindle. Centromere proteins are conserved across the entire eukaryotic kingdom, but evolutionary divergence among these proteins makes direct alignment of centromeric sequences challenging. Schleiffer *et al*. (35) used advanced homology detection methods to assemble collections of homologous centromere proteins across widely diverged eukaryotic species. Their analysis focused on CENP-T, a human centromere protein that contains one histone domain but is otherwise not well characterized functionally. Schleiffer *et al*. identified Cnn1, a yeast homolog of the CENP-T, and demonstrated using size-exclusion chromatography and several other lines of evidence that Cnn1 is a binding partner to the Ndc80 complex, one of the core units of the kinetichore. Schleiffer *et al*. then used meme to analyze a set of CENP-T homologs, identifying within the unaligned N-terminal region of the proteins a 15-residue sequence motif. A knockout version of Cnn1 with the motif removed fails to interact with Ndc80, suggesting that this motif is directly involved in the interaction with the Ndc80 complex. Conversely, to establish that the 15-residue motif is sufficient for binding Ndc80, Schleiffer *et al*. synthesized a 25-amino-acid peptide including the motif and demonstrated evidence of binding using isothermal titration calorimetry. Overall, this analysis demonstrates the utility of meme in identifying functionally important sites among distantly related protein sequences. (All input files and the outputs generated for this case study are contained in supplementary file ‘case2.zip’.)

We can recapitulate the analysis of Schleiffer *et al*. as follows. First, we download Supplementary Table S2 from the original paper and extract from the Excel spreadsheet the protein identifier from the third column (‘NCBI GenPept’) for all of the CENP-T proteins (as indicated in the first column). We then upload these IDs to Batch Entrez (http://www.ncbi.nlm.nih.gov/sites/batchentrez), saving the resulting 31 proteins in FASTA format. This file can then be uploaded to the meme server, as described above in Case Study 1. Following the procedure described by Schleiffer *et al*., we use the ‘one occurrence per sequence’ model, and we set the minimum and maximum motif widths to 10 and 25 amino acids, respectively. All other parameters are left at their default values.

**Figure 4.**
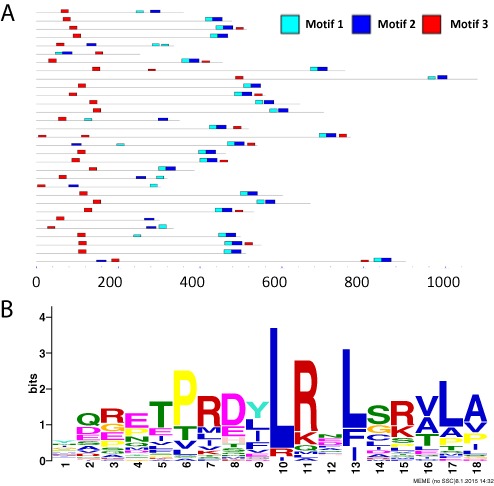
Discovery of CENP-T motif involved in interacting with Ndc80. (**A**) The locations of the three discovered motifs within the 31 CENP-T homologs. The figure includes non-overlapping sites with a *P*-value better than 0.0001. The height of the motif ‘block’ is proportional to −log (*P*-value), truncated at the height for a motif with a *P*-value of 10^−10^. (**B**) The third motif found by meme in CENP-T homologs.

In the resulting output file (‘cenp-t/meme.html’ in supplementary file ‘case2.zip’), the first two motifs correspond to components of the known histone domain. In most of the CENP-T homologs, these motifs occur side by side, with motif 1 immediately N-terminal to motif 2 (Figure 4A). Furthermore, in nearly every case these motifs occur near the C-terminus of the protein. The third motif (Figure 4B) corresponds to the Ndc80 interaction motif identified by Schleiffer *et al*. This width-18 motif corresponds to the width-15 alignment shown in Figure 6A of (35), with four additional amino acids on the left and one missing (and poorly conserved) amino acid on the right. Intriguingly, the pattern of motif occurrences in Figure 4A suggests that a significant proportion of the CENP-T homologs harbor a second copy of the Ndc80 interaction motif (motif 3, red) near the C-terminus of the protein. These secondary motif occurrences were not discussed by Schleiffer *et al.*

## CASE STUDY 3: IDENTIFYING LIVER-SPECIFIC GENES BASED ON CLUSTERS OF DNA MOTIFS

Precise regulatory control of genes, particularly in eukaryotes, frequently requires the joint action of multiple sequence-specific transcription factors. A CRM is a genomic locus that is responsible for gene regulation and that contains multiple transcription factor binding sites in close proximity. The MEME Suite tool mcast identifies, within a genomic sequence, candidate CRMs consisting of clusters of motifs.

An example of this type of analysis was carried out by Thompson *et al*. in their analysis of genetic variants of the human CYP3A gene (36). The study, carried out in 2004, involved targeted resequencing of genomic regions associated with the genes CYP3A4 and CYP3A5. In addition to exonic sequences, the resequencing included candidate CRMs identified as containing clusters of putative binding sites for liver-specific transcription factors. (All input files and the outputs generated for this case study are contained in supplementary file ‘case3.zip’.)

We carried out a similar analysis using mcast. To do so, we downloaded the MEME Suite motif databases by clicking on ‘Download MEME Suite and Databases’ under ‘Download & Install’. Within the databases, we identified, based on Thompson's analysis, six liver-specific motifs: HNF1α, HNF4, OCT-1, PPAR/RXR, CEBP and HNF3β. We then used the UCSC Genome Browser to identify, in assembly hg19 of the human genome, the coordinates of the longest isoforms of CYP3A4 (chr7:99,354,583-99,381,811) and CYP3A5 (chr7:99,245,813-99,332,819). On the browser, we used the ‘View→DNA’ option to extract the DNA for a single locus containing both genes, selecting the option to mask repeats to N's. Note that, to enable mcast to compute accurate statistics, we extracted a relatively large (1 Mbp) region (chr7:99,000,000-100,000,000). We then submitted the six motifs and the 1 Mbp sequence to the mcast web form (click on ‘MCAST’ on the MEME Suite homepage, Figure 1, to reach the mcast web form). Under ‘Advanced options’, we set the pseudocount to 4, the *P*-value threshold to 0.001, the motif spacing to 250 bp and the *E*-value threshold to 100. Finally, we filtered the resulting mcast output to consider only predicted CRMs that fall within 5 kbp of one of the two genes (i.e. within the range chr7:99,240,813-99,386,811) and achieve a *P*-value ≤0.05.

**Figure 5.**
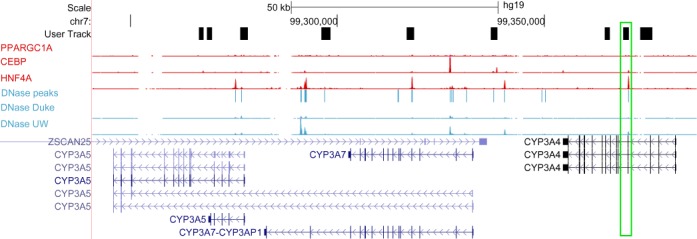
Using mcast to predict CRMs near CYP3A4 and CYP3A5. The figure shows nine mcast-predicted CRMs alongside five experimental tracks: three ChIP-seq experiments for liver-specific factors PPARGC1A, CEBP and HNF4α, and two DNaseI sensitivity analyses. The best-scoring CRM is boxed in green. The DNaseI experiments are illustrated both as signal tracks and as a combined set of peak calls. Various isoforms of several CYP3A genes are shown, as well as a portion of the gene ZSCAN25.

The result of this analysis is a set of nine predicted CRMs (Figure 5 and ‘mcast-web/mcast.html’ in supplementary file ‘case3.zip’). To evaluate the plausibility of these regions as CRMs, we used the ‘track hubs’ button on the UCSC Genome Browser to connect to the ENCODE Analysis Hub. From there, we turned on five specific tracks, corresponding to five experiments carried out in the HepG2 hepatocellular (liver) carcinoma cell line: three ChIP-seq analyses of liver-specific factors PPARGC1A, CEBP and HNF4α as well as two DNaseI sensitivity analyses. The top-scoring predicted CRM—in the third exon of CYP3A4—shows evidence of regulatory activity: it overlaps pronounced peaks in the ChIP-seq HNF4α track and the DNaseI sensitivity track. In total, five of the nine predicted CRMs overlap a DNaseI peak, most of which also correspond to peaks in the ChIP-seq tracks.

## CASE STUDY 4: ANALYSIS OF PEAKS FROM CHIP-SEQ OF A VITAMIN D RECEPTOR PROTEIN

Chromatin immunoprecipitation coupled with high-throughput sequencing (ChIP-seq) is a powerful assay for detecting in a high-throughput fashion genomic regions bound by a particular transcription factor or associated with a particular histone modification. Each ChIP-seq experiment produces as output hundreds or thousands of ‘peak’ regions, each of which potentially harbors one or more sequence-specific binding motifs. The MEME Suite offers many tools that can be brought to bear in the analysis of ChIP-seq data. To simplify this type of analysis, we have combined a variety of motif-based analyses into a single, simple user interface, called meme-Chip. Here we describe one straightforward application of meme-Chip. Readers interested in more detail can find it in our recently published protocol (16). (All input files and the outputs generated for this case study are contained in supplementary file ‘case4.zip’.)

For this case study, we use ChIP-seq data for the nuclear vitamin D receptor protein VDR (37). Motivated by the clinical relevance of vitamin D to diseases such as multiple sclerosis, rheumatoid arthritis, type I diabetes and conditions such as rickets-induced pelvic contraction, Ramagopalan *et al*. sought to gain a detailed understanding of the biological action of vitamin D by profiling the binding of VDR in a lymphoblastoid cell line. Their analysis, which was carried out prior to the availability of the meme-Chip pipeline, involved several MEME Suite tools, including MEME and Tomtom. You can easily reproduce and extend their results simply by uploading their data to the meme-Chip web form.

The analysis proceeds as follows. First, download Supplementary Table S1 from Ramagopalan *et al*. (37) and extract the first three columns into a text file called ‘Supplementary_Table_1.bed.’ meme-Chip works best on sequences of length ∼500 bp, so rather than using the variable-length sequences provided by the authors, you can convert the coordinates to 500 bp windows centered on each peak by using the following command:

**Figure:**
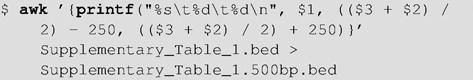


In this case, rather than using the fastaFromBed utility in BedTools, as in Case Study 1, we use the UCSC Table Browser to convert from a BED file to FASTA. This allows you to perform repeat masking on the sequence. The ‘Supplementary_Table_1.500bp.bed’ file can be uploaded to http://genome.ucsc.edu/cgi-bin/hgTables, selecting ‘sequence’ as the output format. After clicking ‘get output’, choose ‘Mask repeats to N’ and then save the resulting file to your hard drive. That file can then be uploaded to the meme-Chip web form, leaving the default set of motifs as ‘Jaspar Vertebrates and UniPROBE Mouse’ and leaving all other options at their default values.

**Figure 6.**
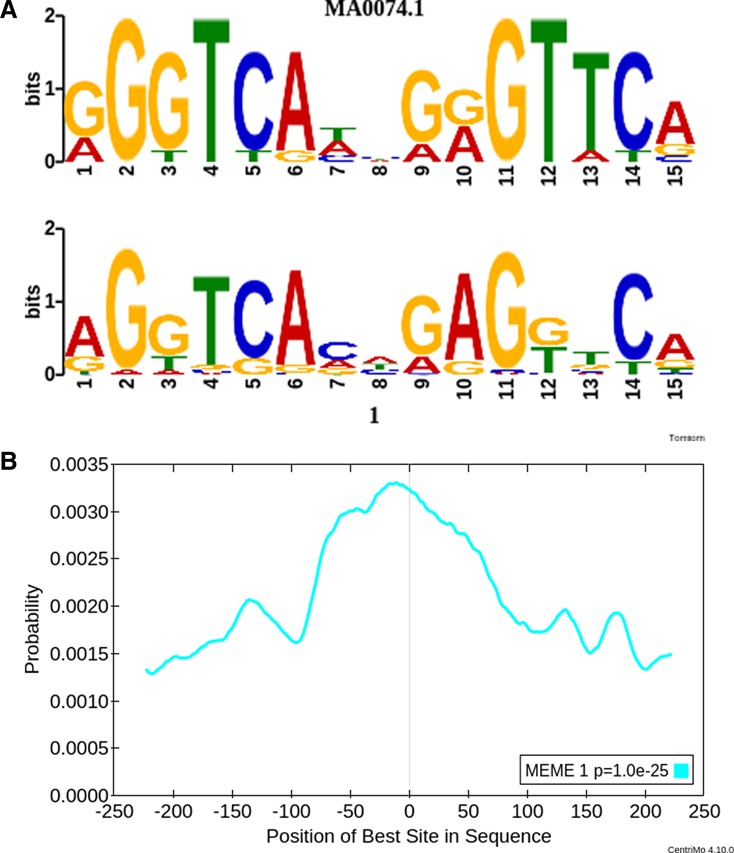
meme-Chip analysis of VDR Chip-seq data. (**A**) Tomtom alignment of the JASPAR motif for VDR (top) and the first motif discovered by meme (bottom). (**B**) CentriMo analysis showing central enrichment of the VDR motif within the given ChIP-seq peaks.

In the resulting meme-Chip output (‘meme-chip/meme-chip.html’ in supplementary file ‘case4.zip’), we see that meme successfully identified the known VDR motif as the top-ranked motif (Figure 6A). In addition, CentriMo analysis indicates that this motif is centrally enriched in the supplied 500 bp regions (Figure 6B). The CentriMo analysis provides additional evidence that the motif is real, since meme does not make use of positional information during the motif discovery phase and since we expect motifs to be enriched on average close to the centers of the ChIP-seq peak regions. Other motifs found by DREME correspond to other known transcription factors, including ELK4 and IRF1 (not shown). It is worth noting that, if you do this analysis but do not include the repeat masking step, then the results are slightly less compelling: in that case, the VDR motif is ranked second rather than first, and the top-ranked motif corresponds to a slightly degraded version of the IRF1 motif (data not shown).

## SUPPLEMENTARY DATA

Supplementary Data are available at NAR online.

SUPPLEMENTARY DATA
