# Supplementary material for: The MEME Suite
Source: Nucleic Acids Res. 2015 May 7;43(Web Server issue):W39–49. doi: 10.1093/nar/gkv416 (PMC4489269; doi:10.1093/nar/gkv416)
Supplement: SUPPLEMENTARY DATA [file supp_43_W1_W39__index.html]

The MEME Suite — SUPPLEMENTARY DATA 

# The MEME Suite

## SUPPLEMENTARY DATA

- SUPPLEMENTARY DATA
- SUPPLEMENTARY DATA
- SUPPLEMENTARY DATA
- SUPPLEMENTARY DATA
- SUPPLEMENTARY DATA
