# Supplementary material for: The MEME Suite
Source: Nucleic Acids Res. 2015 May 7;43(Web Server issue):W39–49. doi: 10.1093/nar/gkv416 (PMC4489269; doi:10.1093/nar/gkv416)
Supplement: SUPPLEMENTARY DATA [file supp_gkv416_nar-00283-web-b-2015-File002.zip › case1/Hiller2004.1.fimo.out/fimo.html]

FIMO Results


---

|  |  |  |
| --- | --- | --- |
| **Database and Motifs** | **High-scoring Motif Occurrences** | **Debugging Information** |

  
  

---

**FIMO - Motif search tool**


---

FIMO version 4.10.0,
(Release date: Wed May 21 10:35:36 2014 +1000)

For further information on how to interpret these results
or to get a copy of the FIMO software please access
http://meme.nbcr.net

If you use FIMO in your research, please cite the following paper:  
Charles E. Grant, Timothy L. Bailey, and William Stafford Noble,
"FIMO: Scanning for occurrences of a given motif",
*Bioinformatics*, **27**(7):1017-1018, 2011.
[full text]

---

**DATABASE AND MOTIFS**


---

DATABASE
/Users/t.bailey/Genomes\_local/Pf276v1.24/Plasmodium\_falciparum.ASM276v1.24.pep.all.fa  
Database contains
5414
sequences,
4115585
residues

MOTIFS
Hiller2004.1.out/meme.html
(protein)

| MOTIF | WIDTH | BEST POSSIBLE MATCH |
| --- | --- | --- |
| 1 | 11 | FRNKRILAETE |

Random model letter frequencies
(from non-redundant database):
  
A 0.073 C 0.018 D 0.052 E 0.062 F 0.040 G 0.069 H 0.022 I 0.056 K 0.058
  
L 0.092 M 0.023 N 0.046 P 0.051 Q 0.041 R 0.052 S 0.074 T 0.059 V 0.064
  
W 0.013 Y 0.033

---

**SECTION I: HIGH-SCORING MOTIF OCCURRENCES**


---

- There were
  469
  motif occurrences with a
  p-value less than
  0.0001.
- The p-value of a motif occurrence is defined as the
  probability of a random sequence of the same length as the motif
  matching that position of the sequence with as good or better a score.
- The score for the match of a position in a sequence to a motif
  is computed by summing the appropriate entries from each column of
  the position-dependent scoring matrix that represents the motif.
- The q-value of a motif occurrence is defined as the
  false discovery rate if the occurrence is accepted as significant.
- The table is sorted by increasing p-value.

| Motif | Sequence Name | Strand | Start | End | p-value | q-value | Matched Sequence |
| --- | --- | --- | --- | --- | --- | --- | --- |
| 1 | PFB0100c:pep | . | 50 | 60 | 1.57e-12 | 5.85e-06 | `FRNKRTLAQKQ` |
| 1 | PF10\_0159:pep | . | 80 | 90 | 7.27e-11 | 0.000106 | `FRESRILAEGE` |
| 1 | MAL7P1.231:pep | . | 41 | 51 | 9.79e-11 | 0.000106 | `NLNKRLLHETQ` |
| 1 | PFE0040c:pep | . | 71 | 81 | 1.13e-10 | 0.000106 | `DRYVRILSETE` |
| 1 | PFB0095c:pep | . | 56 | 66 | 4.57e-10 | 0.000341 | `IRLKRSLAQVL` |
| 1 | PF07\_0004:pep | . | 56 | 66 | 1.02e-07 | 0.0572 | `IRYQRILIQTE` |
| 1 | PFA0660w:pep | . | 54 | 64 | 1.17e-07 | 0.0572 | `IRYKRCLAEGN` |
| 1 | MAL13P1.480:pep | . | 41 | 51 | 1.23e-07 | 0.0572 | `NSNKRLLHESQ` |
| 1 | MAL7P1.170:pep | . | 41 | 51 | 1.41e-07 | 0.0583 | `FRNNRILSSKE` |
| 1 | PFL0765w:pep | . | 624 | 634 | 1.97e-07 | 0.0658 | `DRLKRISHFTE` |
| 1 | PF14\_0010:pep | . | 80 | 90 | 2.14e-07 | 0.0658 | `FRESRVLAEGK` |
| 1 | PFB0065w:pep | . | 44 | 54 | 2.29e-07 | 0.0658 | `TINSRLLAQTQ` |
| 1 | PFI0080w:pep | . | 44 | 54 | 2.29e-07 | 0.0658 | `TINSRLLAQTQ` |
| 1 | PFD1170c:pep | . | 82 | 92 | 2.63e-07 | 0.0699 | `NVGKRILSEGQ` |
| 1 | MAL7P1.7:pep | . | 79 | 89 | 2.81e-07 | 0.0699 | `NKYSRILIETE` |
| 1 | PFI1785w:pep | . | 82 | 92 | 4e-07 | 0.0934 | `KEYSRILSETE` |
| 1 | MAL7P1.223:pep | . | 44 | 54 | 4.57e-07 | 0.1 | `IIKSRLLAQTQ` |
| 1 | MAL13P1.75:pep | . | 84 | 94 | 5.8e-07 | 0.12 | `NRNKRLLQIGL` |
| 1 | MAL13P1.490:pep | . | 37 | 47 | 6.35e-07 | 0.125 | `ILKSRCLAETE` |
| 1 | PF11\_0203:pep | . | 864 | 874 | 6.71e-07 | 0.125 | `DNEVRSLIETQ` |
| 1 | PFI1525w:pep | . | 110 | 120 | 8.91e-07 | 0.158 | `IREKKLLKEKE` |
| 1 | PF14\_0206:pep | . | 16 | 26 | 9.7e-07 | 0.165 | `ISNVRIKAQKL` |
| 1 | PFA0430c:pep | . | 570 | 580 | 1.1e-06 | 0.179 | `NLLKRILYNVE` |
| 1 | PF13\_0126:pep | . | 59 | 69 | 1.58e-06 | 0.245 | `FLNSSILSQGY` |
| 1 | PF10\_0374:pep | . | 90 | 100 | 1.8e-06 | 0.26 | `FRGFRILAENE` |
| 1 | PF10\_0070:pep | . | 97 | 107 | 1.85e-06 | 0.26 | `NLNERILSVGL` |
| 1 | MAL13P1.395:pep | . | 733 | 743 | 1.91e-06 | 0.26 | `FLYKLSISEVE` |
| 1 | PF11\_0309:pep | . | 76 | 86 | 1.95e-06 | 0.26 | `ILNKYTLKEKL` |
| 1 | PF14\_0031b:pep | . | 193 | 203 | 2.27e-06 | 0.293 | `FRNVRILNEEK` |
| 1 | PF10\_0024:pep | . | 113 | 123 | 2.72e-06 | 0.306 | `LRYKRLLAEPI` |
| 1 | PF11\_0206:pep | . | 1309 | 1319 | 2.96e-06 | 0.306 | `NRNKTIHVQTQ` |
| 1 | PFE0385w:pep | . | 1505 | 1515 | 3.03e-06 | 0.306 | `NRTKRIIAQKM` |
| 1 | MAL13P1.61:pep | . | 42 | 52 | 3.11e-06 | 0.306 | `IRSFRSLAENQ` |
| 1 | PF13\_0071:pep | . | 507 | 517 | 3.3e-06 | 0.306 | `MLLVRLKHEKL` |
| 1 | PFB0555c:pep | . | 389 | 399 | 3.35e-06 | 0.306 | `FVYKRILIQIL` |
| 1 | PFI1750c:pep | . | 45 | 55 | 3.47e-06 | 0.306 | `IRNERILSEQH` |
| 1 | PFB0715w:pep | . | 349 | 359 | 3.65e-06 | 0.306 | `SREKRILYAKE` |
| 1 | PFC0055w:pep | . | 42 | 52 | 3.91e-06 | 0.306 | `IRNNRSLAKYE` |
| 1 | PF11\_0079:pep | . | 935 | 945 | 4e-06 | 0.306 | `QRYKTLLHITQ` |
| 1 | PF10\_0020:pep | . | 82 | 92 | 4.07e-06 | 0.306 | `DRYTRKLAEAL` |
| 1 | PFB0070w:pep | . | 42 | 52 | 4.24e-06 | 0.306 | `ARHKRLLSESE` |
| 1 | PFA0510w:pep | . | 1184 | 1194 | 4.43e-06 | 0.306 | `NSNKRLLKQWE` |
| 1 | PFD1185w:pep | . | 61 | 71 | 4.43e-06 | 0.306 | `NIYKRNLVQVE` |
| 1 | PFD1215w:pep | . | 61 | 71 | 4.43e-06 | 0.306 | `NIYKRNLVQVE` |
| 1 | PF10\_0015:pep | . | 93 | 103 | 4.43e-06 | 0.306 | `NIYKRNLVQVE` |
| 1 | PFL2590w:pep | . | 59 | 69 | 4.43e-06 | 0.306 | `NIYKRNLVQVE` |
| 1 | PF14\_0763:pep | . | 59 | 69 | 4.43e-06 | 0.306 | `NIYKRNLVQVE` |
| 1 | PF10\_0265:pep | . | 473 | 483 | 4.47e-06 | 0.306 | `FLKKHILQETQ` |
| 1 | PFE0320w:pep | . | 440 | 450 | 4.92e-06 | 0.306 | `NKNKETLNQTQ` |
| 1 | PF08\_0063:pep | . | 410 | 420 | 5.41e-06 | 0.306 | `ERLKSILKEVQ` |
| 1 | PF13\_0076:pep | . | 87 | 97 | 5.48e-06 | 0.306 | `SRINRSLSEKQ` |
| 1 | MAL13P1.70:pep | . | 1993 | 2003 | 5.48e-06 | 0.306 | `NILKRIKHETG` |
| 1 | PFL0870w:pep | . | 330 | 340 | 5.63e-06 | 0.306 | `IFYKRKGAEKE` |
| 1 | PF11\_0035:pep | . | 230 | 240 | 5.88e-06 | 0.306 | `QREKRLKKEKQ` |
| 1 | MAL7P1.174:pep | . | 48 | 58 | 5.93e-06 | 0.306 | `LSNIRILSEVE` |
| 1 | PFA0705c:pep | . | 44 | 54 | 5.99e-06 | 0.306 | `TIKSRLLAQTQ` |
| 1 | PFA0750w:pep | . | 44 | 54 | 5.99e-06 | 0.306 | `TIKSRLLAQTQ` |
| 1 | PFB0025c:pep | . | 44 | 54 | 5.99e-06 | 0.306 | `TIKSRLLAQTQ` |
| 1 | PFC0025c:pep | . | 44 | 54 | 5.99e-06 | 0.306 | `TIKSRLLAQTQ` |
| 1 | PFD0035c:pep | . | 44 | 54 | 5.99e-06 | 0.306 | `TIKSRLLAQTQ` |
| 1 | PFF1550w:pep | . | 44 | 54 | 5.99e-06 | 0.306 | `TIKSRLLAQTQ` |
| 1 | MAL8P1.217:pep | . | 44 | 54 | 5.99e-06 | 0.306 | `TIKSRLLAQTQ` |
| 1 | PF10\_0395:pep | . | 44 | 54 | 5.99e-06 | 0.306 | `TIKSRLLAQTQ` |
| 1 | PF11\_0516:pep | . | 44 | 54 | 5.99e-06 | 0.306 | `TIKSRLLAQTQ` |
| 1 | PFL2635w:pep | . | 44 | 54 | 5.99e-06 | 0.306 | `TIKSRLLAQTQ` |
| 1 | PF14\_0007:pep | . | 44 | 54 | 5.99e-06 | 0.306 | `TIKSRLLAQTQ` |
| 1 | PF14\_0767:pep | . | 44 | 54 | 5.99e-06 | 0.306 | `TIKSRLLAQTQ` |
| 1 | PF14\_0771:pep | . | 44 | 54 | 5.99e-06 | 0.306 | `TIKSRLLAQTQ` |
| 1 | PF11\_0305:pep | . | 401 | 411 | 6.13e-06 | 0.306 | `IKEIRDLAQKQ` |
| 1 | MAL7P1.225.1 | . | 61 | 71 | 6.21e-06 | 0.306 | `NIYRRNLSETE` |
| 1 | MAL7P1.225.2 | . | 61 | 71 | 6.21e-06 | 0.306 | `NIYRRNLSETE` |
| 1 | PF10\_0344:pep | . | 30 | 40 | 6.36e-06 | 0.306 | `NRNKRIGGPKL` |
| 1 | PFA0090c:pep | . | 44 | 54 | 6.48e-06 | 0.306 | `TINSRLLAQTK` |
| 1 | PFB1020w:pep | . | 44 | 54 | 6.48e-06 | 0.306 | `TINSRLLAQTK` |
| 1 | PFC1105w:pep | . | 44 | 54 | 6.48e-06 | 0.306 | `TINSRLLAQTK` |
| 1 | PF07\_0130:pep | . | 44 | 54 | 6.48e-06 | 0.306 | `TINSRLLAQTK` |
| 1 | PFI0045c:pep | . | 44 | 54 | 6.48e-06 | 0.306 | `TINSRLLAQTK` |
| 1 | PFL2610w:pep | . | 44 | 54 | 6.48e-06 | 0.306 | `TINSRLLAQTK` |
| 1 | MAL13P1.7:pep | . | 44 | 54 | 6.48e-06 | 0.306 | `TINSRLLAQTK` |
| 1 | MAL8P1.4:pep | . | 70 | 80 | 6.63e-06 | 0.309 | `NRDSRILYEFL` |
| 1 | PFE1480c:pep | . | 392 | 402 | 7.94e-06 | 0.366 | `FNNACILHEKL` |
| 1 | PFD0670c:pep | . | 36 | 46 | 8.22e-06 | 0.371 | `ARLVRILSEFV` |
| 1 | PF13\_0127:pep | . | 348 | 358 | 8.26e-06 | 0.371 | `NGNIFTLHQKQ` |
| 1 | PF14\_0013:pep | . | 61 | 71 | 8.54e-06 | 0.378 | `NVYSRNLSESQ` |
| 1 | PF10\_0076:pep | . | 492 | 502 | 8.62e-06 | 0.378 | `DREKKNLHTVE` |
| 1 | PFA0310c:pep | . | 376 | 386 | 8.74e-06 | 0.379 | `FRESDSLTEYQ` |
| 1 | PF11\_0249:pep | . | 3 | 13 | 9.04e-06 | 0.385 | `NLNKRKLTCVE` |
| 1 | PF07\_0024:pep | . | 49 | 59 | 9.08e-06 | 0.385 | `CLEKRILKEKI` |
| 1 | PFL1085w:pep | . | 2137 | 2147 | 9.4e-06 | 0.394 | `IRNSTILPQKG` |
| 1 | PFL0360c:pep | . | 891 | 901 | 9.53e-06 | 0.395 | `DKNKKSLQEKQ` |
| 1 | PF07\_0101:pep | . | 1419 | 1429 | 1.03e-05 | 0.417 | `IKEKKILLEKQ` |
| 1 | MAL8P1.205:pep | . | 45 | 55 | 1.06e-05 | 0.417 | `NICSRKLSETE` |
| 1 | MAL13P1.329:pep | . | 138 | 148 | 1.06e-05 | 0.417 | `NLENRILIESE` |
| 1 | PFE1095w:pep | . | 1738 | 1748 | 1.07e-05 | 0.417 | `LKNKRSLSTVE` |
| 1 | PFD0940w:pep | . | 1796 | 1806 | 1.08e-05 | 0.417 | `DNYKRLFHEVK` |
| 1 | PFF0275c:pep | . | 643 | 653 | 1.08e-05 | 0.417 | `DLIKRILKENL` |
| 1 | PF14\_0753:pep | . | 42 | 52 | 1.1e-05 | 0.417 | `ITYSRSLAEYD` |
| 1 | PFL1855w:pep | . | 591 | 601 | 1.12e-05 | 0.417 | `DRLKDIIQEGQ` |
| 1 | PF14\_0327:pep | . | 88 | 98 | 1.12e-05 | 0.417 | `DNNVRINVQGE` |
| 1 | PF11\_0358:pep | . | 194 | 204 | 1.13e-05 | 0.417 | `NLNKKELAQKG` |
| 1 | PF13\_0169:pep | . | 41 | 51 | 1.13e-05 | 0.417 | `LREKILLKEKE` |
| 1 | PF11\_0224:pep | . | 15 | 25 | 1.14e-05 | 0.417 | `IFNKESLAEKT` |
| 1 | PF10\_0186:pep | . | 1721 | 1731 | 1.16e-05 | 0.421 | `DFYKTTLAQKK` |
| 1 | PFE0765w:pep | . | 1526 | 1536 | 1.19e-05 | 0.428 | `FQYKRSSDEKL` |
| 1 | PFD1220c:pep | . | 37 | 47 | 1.23e-05 | 0.436 | `ILKSRCLAEIE` |
| 1 | MAL7P1.134:pep | . | 839 | 849 | 1.25e-05 | 0.439 | `FSYKIIIAEGL` |
| 1 | PF11\_0400:pep | . | 1466 | 1476 | 1.26e-05 | 0.44 | `DENKKSLTEKE` |
| 1 | PFF1440w:pep | . | 3234 | 3244 | 1.27e-05 | 0.44 | `FRCSFILYEVL` |
| 1 | PFC0865w:pep | . | 128 | 138 | 1.31e-05 | 0.448 | `NEYKPTGAEGQ` |
| 1 | PFL1815c:pep | . | 1627 | 1637 | 1.32e-05 | 0.448 | `IRNSRSLSDIS` |
| 1 | PFD1115c:pep | . | 1443 | 1453 | 1.36e-05 | 0.456 | `DSNKKILIEKL` |
| 1 | PFL2550w:pep | . | 36 | 46 | 1.37e-05 | 0.458 | `NVYSRILSDLE` |
| 1 | PFD0235c:pep | . | 39 | 49 | 1.39e-05 | 0.458 | `KLLKRVLHNVE` |
| 1 | MAL7P1.177:pep | . | 42 | 52 | 1.4e-05 | 0.458 | `IRISRLLSQNN` |
| 1 | MAL13P1.304:pep | . | 573 | 583 | 1.42e-05 | 0.46 | `NKNVELLNETQ` |
| 1 | PFA0125c:pep | . | 1182 | 1192 | 1.45e-05 | 0.462 | `NREDPIASETE` |
| 1 | PF11\_0077:pep | . | 462 | 472 | 1.45e-05 | 0.462 | `DILKRINIQTL` |
| 1 | MAL8P1.3:pep | . | 56 | 66 | 1.48e-05 | 0.468 | `DRLSRSLALRS` |
| 1 | MAL7P1.176:pep | . | 1227 | 1237 | 1.52e-05 | 0.478 | `DRNSNTLHLKD` |
| 1 | PFF1280w:pep | . | 104 | 114 | 1.54e-05 | 0.478 | `FRLSSILPIKE` |
| 1 | PF14\_0413:pep | . | 439 | 449 | 1.58e-05 | 0.484 | `NPYLRLLAHTL` |
| 1 | PF10\_0085:pep | . | 359 | 369 | 1.59e-05 | 0.484 | `GRISRSLAAKL` |
| 1 | MAL8P1.103:pep | . | 5 | 15 | 1.6e-05 | 0.484 | `DKLKNILDQKE` |
| 1 | PFL2285c:pep | . | 189 | 199 | 1.61e-05 | 0.484 | `FECKILLAEKL` |
| 1 | PFE1150w:pep | . | 253 | 263 | 1.68e-05 | 0.497 | `ILNKFNLSETF` |
| 1 | PFB0680w:pep | . | 937 | 947 | 1.7e-05 | 0.497 | `TLYVLPLHEKQ` |
| 1 | PF11\_0185:pep | . | 1347 | 1357 | 1.7e-05 | 0.497 | `NKEKAILSDTE` |
| 1 | PF14\_0419:pep | . | 2509 | 2519 | 1.7e-05 | 0.497 | `FLNETILSEKK` |
| 1 | PF11\_0420:pep | . | 538 | 548 | 1.72e-05 | 0.499 | `FLLKDILLQKK` |
| 1 | PFI0745w:pep | . | 209 | 219 | 1.77e-05 | 0.506 | `IKNKRSNFQKL` |
| 1 | MAL8P1.101:pep | . | 695 | 705 | 1.79e-05 | 0.506 | `DLNKILLYKTQ` |
| 1 | PF10\_0307:pep | . | 145 | 155 | 1.8e-05 | 0.506 | `DRNKISLLKKL` |
| 1 | PF13\_0292:pep | . | 47 | 57 | 1.8e-05 | 0.506 | `NVLKRIKSEKN` |
| 1 | PF14\_0694:pep | . | 334 | 344 | 1.85e-05 | 0.515 | `IFEKRLLNELL` |
| 1 | MAL7P1.173:pep | . | 193 | 203 | 1.92e-05 | 0.526 | `DNEDISLHEKQ` |
| 1 | PF14\_0281:pep | . | 42 | 52 | 1.92e-05 | 0.526 | `INNSRILSDVD` |
| 1 | PF10\_0244:pep | . | 816 | 826 | 2e-05 | 0.542 | `ITNKRLNIQKL` |
| 1 | PF07\_0082:pep | . | 1795 | 1805 | 2.02e-05 | 0.542 | `FLYKFLSKEKQ` |
| 1 | PFC0345w:pep | . | 234 | 244 | 2.03e-05 | 0.542 | `IREKCSVHTKE` |
| 1 | PF08\_0091:pep | . | 848 | 858 | 2.03e-05 | 0.542 | `LLIKRTLSLKE` |
| 1 | PFD1225w:pep | . | 15 | 25 | 2.08e-05 | 0.544 | `NTTSRLLSECE` |
| 1 | PFI1310w:pep | . | 342 | 352 | 2.08e-05 | 0.544 | `NKEKLSLLQKE` |
| 1 | PF10\_0369:pep | . | 790 | 800 | 2.08e-05 | 0.544 | `NLLKCILASTD` |
| 1 | MAL13P1.344:pep | . | 225 | 235 | 2.12e-05 | 0.548 | `DRNVEDLSGGE` |
| 1 | PF10\_0018:pep | . | 46 | 56 | 2.14e-05 | 0.548 | `NIYSRSLGELL` |
| 1 | PFI0320w:pep | . | 349 | 359 | 2.15e-05 | 0.548 | `NLLMKILAETK` |
| 1 | PF07\_0086:pep | . | 1727 | 1737 | 2.18e-05 | 0.548 | `FVYSPLLYEKE` |
| 1 | PFL1455w:pep | . | 900 | 910 | 2.2e-05 | 0.548 | `NLYEPLLHQTN` |
| 1 | PFE0190c:pep | . | 602 | 612 | 2.22e-05 | 0.548 | `IYDSRILHRGL` |
| 1 | PFB0425c:pep | . | 87 | 97 | 2.23e-05 | 0.548 | `INEKKILSEKK` |
| 1 | PFI0795w:pep | . | 381 | 391 | 2.23e-05 | 0.548 | `FLLKDILIEQL` |
| 1 | PF10\_0195:pep | . | 509 | 519 | 2.23e-05 | 0.548 | `NKEKEINSEVQ` |
| 1 | PFB0215c:pep | . | 311 | 321 | 2.28e-05 | 0.553 | `FLNSHIKDEKE` |
| 1 | PFC0960c:pep | . | 1359 | 1369 | 2.3e-05 | 0.553 | `NDLKNTLKEKE` |
| 1 | PF13\_0186:pep | . | 47 | 57 | 2.3e-05 | 0.553 | `ILEKRLLWINE` |
| 1 | PF14\_0569:pep | . | 430 | 440 | 2.32e-05 | 0.553 | `NEEKETLIETL` |
| 1 | PF14\_0711:pep | . | 401 | 411 | 2.33e-05 | 0.553 | `NLEKNILHNKI` |
| 1 | PFE0310c:pep | . | 239 | 249 | 2.38e-05 | 0.554 | `ILLKRKSNEKE` |
| 1 | PF11\_0218:pep | . | 777 | 787 | 2.39e-05 | 0.554 | `NLLKQELEQVQ` |
| 1 | PFL1360c:pep | . | 21 | 31 | 2.4e-05 | 0.554 | `DLEEISLHQLQ` |
| 1 | PF14\_0343:pep | . | 1956 | 1966 | 2.42e-05 | 0.554 | `NLNKCTLCDKE` |
| 1 | PFA0280w:pep | . | 1907 | 1917 | 2.44e-05 | 0.554 | `ILNKHSLEEKN` |
| 1 | PFI0086w:pep | . | 87 | 97 | 2.44e-05 | 0.554 | `KLLNRSLAEVN` |
| 1 | PFL2120w:pep | . | 351 | 361 | 2.44e-05 | 0.554 | `DRNLKLLSYVE` |
| 1 | PF10\_0243:pep | . | 486 | 496 | 2.45e-05 | 0.555 | `ILEKNILDVKQ` |
| 1 | PFC0820w:pep | . | 1893 | 1903 | 2.51e-05 | 0.564 | `IRNTLILSMGL` |
| 1 | PFC0245c:pep | . | 60 | 70 | 2.59e-05 | 0.578 | `FLLFRRLSQCL` |
| 1 | PF11\_0091:pep | . | 1783 | 1793 | 2.61e-05 | 0.578 | `INASRILSNTL` |
| 1 | PFC1005c:pep | . | 7 | 17 | 2.62e-05 | 0.578 | `NMYMCTLAEKL` |
| 1 | PF11\_0456:pep | . | 513 | 523 | 2.7e-05 | 0.593 | `NNNKTISSEKL` |
| 1 | PFA0220w:pep | . | 1100 | 1110 | 2.77e-05 | 0.605 | `DKYSPSDHEKQ` |
| 1 | PF11\_0118:pep | . | 389 | 399 | 2.84e-05 | 0.611 | `NLFITILHEKL` |
| 1 | PFL0130c:pep | . | 1990 | 2000 | 2.84e-05 | 0.611 | `DEESRLLHMKI` |
| 1 | MAL13P1.176:pep | . | 1678 | 1688 | 2.86e-05 | 0.611 | `DLLINILKEKQ` |
| 1 | PF13\_0198:pep | . | 1678 | 1688 | 2.86e-05 | 0.611 | `DLLINILKEKQ` |
| 1 | MAL8P1.12:pep | . | 527 | 537 | 2.92e-05 | 0.62 | `DLEKFQLTQTL` |
| 1 | PFF0275c:pep | . | 368 | 378 | 2.98e-05 | 0.621 | `ILLSRINCQTC` |
| 1 | PF14\_0018:pep | . | 144 | 154 | 2.98e-05 | 0.621 | `IRRSSTLSQGN` |
| 1 | PFL2125c:pep | . | 628 | 638 | 2.99e-05 | 0.621 | `FLENLTKAQKE` |
| 1 | PF10\_0245:pep | . | 583 | 593 | 3.01e-05 | 0.621 | `DLNVRKLSVVN` |
| 1 | PF10\_0337:pep | . | 15 | 25 | 3.01e-05 | 0.621 | `NKEVRILILGL` |
| 1 | PFL1795c:pep | . | 2123 | 2133 | 3.08e-05 | 0.632 | `DQEKRLYVEKL` |
| 1 | MAL7P1.89:pep | . | 4764 | 4774 | 3.11e-05 | 0.634 | `YELSRTLSEKH` |
| 1 | PF13\_0172:pep | . | 123 | 133 | 3.14e-05 | 0.636 | `IIESRLLHFGI` |
| 1 | PF14\_0337:pep | . | 1238 | 1248 | 3.15e-05 | 0.636 | `FDESLITAEKL` |
| 1 | PFB0530c:pep | . | 246 | 256 | 3.21e-05 | 0.642 | `FLLKKLNKEKQ` |
| 1 | PF10\_0281:pep | . | 385 | 395 | 3.24e-05 | 0.642 | `NNEKVSSSEKQ` |
| 1 | PF14\_0386:pep | . | 103 | 113 | 3.24e-05 | 0.642 | `DLYFVTLIEGE` |
| 1 | PFF1070c:pep | . | 634 | 644 | 3.26e-05 | 0.644 | `NLLKNILKSVE` |
| 1 | PF14\_0326:pep | . | 2389 | 2399 | 3.32e-05 | 0.652 | `DLLKNILLQHL` |
| 1 | PF08\_0064:pep | . | 54 | 64 | 3.35e-05 | 0.652 | `ITLVRTLAKDL` |
| 1 | PF11\_0368:pep | . | 1532 | 1542 | 3.35e-05 | 0.652 | `NSEKFSLQQKL` |
| 1 | PF10\_0218:pep | . | 12 | 22 | 3.42e-05 | 0.66 | `ILYKRTGSTKT` |
| 1 | PFE0045c:pep | . | 110 | 120 | 3.47e-05 | 0.664 | `IDENRLNHEVL` |
| 1 | MAL7P1.162:pep | . | 1686 | 1696 | 3.47e-05 | 0.664 | `DRLSDSLSKIQ` |
| 1 | PF11\_0483:pep | . | 221 | 231 | 3.51e-05 | 0.669 | `NKNSKTLKEKL` |
| 1 | PFF0655c:pep | . | 1191 | 1201 | 3.53e-05 | 0.669 | `ILNKKISSYVE` |
| 1 | PF08\_0063:pep | . | 758 | 768 | 3.64e-05 | 0.684 | `RLNKLLKSEKE` |
| 1 | PF10\_0224:pep | . | 5119 | 5129 | 3.65e-05 | 0.684 | `NLHVVSLGQGQ` |
| 1 | PF10\_0079:pep | . | 3453 | 3463 | 3.67e-05 | 0.685 | `DDNSRTPSNTL` |
| 1 | PFE0485w:pep | . | 571 | 581 | 3.7e-05 | 0.685 | `NSNDSTLSQGL` |
| 1 | PFD0330w:pep | . | 146 | 156 | 3.71e-05 | 0.685 | `NDLVNSSHEKQ` |
| 1 | PFB0090c:pep | . | 61 | 71 | 3.74e-05 | 0.688 | `LIIKRNLAQTQ` |
| 1 | MAL7P1.91:pep | . | 534 | 544 | 3.77e-05 | 0.688 | `NENMNSLSEKL` |
| 1 | PF14\_0160:pep | . | 2246 | 2256 | 3.78e-05 | 0.688 | `NRKSLSLSSTL` |
| 1 | PFL0925w:pep | . | 644 | 654 | 3.83e-05 | 0.694 | `ELNSDLLSESE` |
| 1 | PFF1345w:pep | . | 844 | 854 | 3.85e-05 | 0.694 | `DLLSRILSVIN` |
| 1 | PF11\_0504:pep | . | 88 | 98 | 3.91e-05 | 0.701 | `SRYRRIVAEEQ` |
| 1 | PFL1980c:pep | . | 61 | 71 | 3.97e-05 | 0.708 | `KRNKRDLIEKI` |
| 1 | PF13\_0243:pep | . | 250 | 260 | 3.98e-05 | 0.708 | `FLYFLLLSEYL` |
| 1 | PFF1485w:pep | . | 617 | 627 | 4.02e-05 | 0.71 | `FLYSVILTRGL` |
| 1 | PFL2545c:pep | . | 41 | 51 | 4.03e-05 | 0.71 | `LRNFRTLVELQ` |
| 1 | PFD0590c:pep | . | 766 | 776 | 4.12e-05 | 0.723 | `NLNSYILSRKK` |
| 1 | PFL1495w:pep | . | 265 | 275 | 4.18e-05 | 0.728 | `DLNLITLSEYL` |
| 1 | PFA0515w:pep | . | 1295 | 1305 | 4.2e-05 | 0.729 | `ILEKYSLGSVE` |
| 1 | PF13\_0214:pep | . | 237 | 247 | 4.23e-05 | 0.729 | `DLLSDDLAEKK` |
| 1 | PF14\_0708:pep | . | 478 | 488 | 4.25e-05 | 0.729 | `NLNSFISSKKQ` |
| 1 | PFA0610c:pep | . | 44 | 54 | 4.26e-05 | 0.729 | `NRFKRIIAEAS` |
| 1 | PF07\_0013:pep | . | 738 | 748 | 4.3e-05 | 0.733 | `NILVGLLHDKE` |
| 1 | PFC0515c:pep | . | 1129 | 1139 | 4.37e-05 | 0.737 | `ILLDITLDEKE` |
| 1 | PFE0230w:pep | . | 1390 | 1400 | 4.4e-05 | 0.737 | `EREKRRLKERE` |
| 1 | PFC0425w:pep | . | 746 | 756 | 4.44e-05 | 0.737 | `IEESRSISQGN` |
| 1 | PFI1468c:pep | . | 789 | 799 | 4.44e-05 | 0.737 | `ILLKKTFKEKE` |
| 1 | PFE1045c:pep | . | 945 | 955 | 4.48e-05 | 0.737 | `FRNIRKLRTKE` |
| 1 | PFE1045c:pep | . | 1068 | 1078 | 4.48e-05 | 0.737 | `ITNSLILSRVL` |
| 1 | PFD1110w:pep | . | 145 | 155 | 4.49e-05 | 0.737 | `DLLSSILRLVQ` |
| 1 | PFF0700c:pep | . | 131 | 141 | 4.51e-05 | 0.737 | `FKNKRTLIEAI` |
| 1 | PF11\_0175:pep | . | 326 | 336 | 4.52e-05 | 0.737 | `NLLKPVLSKGE` |
| 1 | PF13\_0237:pep | . | 644 | 654 | 4.53e-05 | 0.737 | `NLYKYSNEEKL` |
| 1 | PF13\_0213:pep | . | 77 | 87 | 4.57e-05 | 0.737 | `ILNSGLLAVVG` |
| 1 | PF14\_0139:pep | . | 453 | 463 | 4.64e-05 | 0.737 | `NLYKCLLCRKL` |
| 1 | PF11\_0348:pep | . | 33 | 43 | 4.66e-05 | 0.737 | `ILRKRTFSFKL` |
| 1 | PFB0575c:pep | . | 98 | 108 | 4.69e-05 | 0.737 | `NLNKAIISLGL` |
| 1 | PFI0910w:pep | . | 115 | 125 | 4.71e-05 | 0.737 | `LRNKKIVAETI` |
| 1 | PFC0795w:pep | . | 110 | 120 | 4.75e-05 | 0.737 | `NLLKKILSNAL` |
| 1 | PFL2420w:pep | . | 74 | 84 | 4.75e-05 | 0.737 | `DLLNKSLLEGE` |
| 1 | PF13\_0148:pep | . | 3429 | 3439 | 4.78e-05 | 0.737 | `VRNKRIRSECY` |
| 1 | PF14\_0342:pep | . | 441 | 451 | 4.78e-05 | 0.737 | `DRNKRINKNTN` |
| 1 | PFE0405c:pep | . | 93 | 103 | 4.8e-05 | 0.737 | `ILNKISNSLGE` |
| 1 | MAL8P1.113:pep | . | 2976 | 2986 | 4.86e-05 | 0.737 | `LRNKLILMNTE` |
| 1 | PF11\_0290:pep | . | 257 | 267 | 4.86e-05 | 0.737 | `NLLVRSGSNNQ` |
| 1 | PFB0932w:pep | . | 83 | 93 | 4.87e-05 | 0.737 | `FRINRLLVETK` |
| 1 | MAL13P1.234:pep | . | 1793 | 1803 | 4.87e-05 | 0.737 | `ILNDNILSKGL` |
| 1 | PF10\_0024:pep | . | 231 | 241 | 4.9e-05 | 0.737 | `DLESVISSEEE` |
| 1 | PF14\_0429:pep | . | 438 | 448 | 4.9e-05 | 0.737 | `FLLSATLSLTV` |
| 1 | PFE1195w:pep | . | 1100 | 1110 | 4.92e-05 | 0.737 | `NLASSTLSHKQ` |
| 1 | PFI1485c:pep | . | 1287 | 1297 | 4.94e-05 | 0.737 | `FRNIFHLFQVQ` |
| 1 | MAL13P1.23.1 | . | 441 | 451 | 4.96e-05 | 0.737 | `FLYVTSLVIVL` |
| 1 | MAL13P1.23.2 | . | 441 | 451 | 4.96e-05 | 0.737 | `FLYVTSLVIVL` |
| 1 | PFB0575c:pep | . | 314 | 324 | 4.98e-05 | 0.737 | `ILYSIFVHEKL` |
| 1 | MAL8P1.123:pep | . | 1181 | 1191 | 5.01e-05 | 0.737 | `ILLKNILSLLL` |
| 1 | PFB0930w:pep | . | 51 | 61 | 5.09e-05 | 0.737 | `FGNNRSLAEYY` |
| 1 | PF11\_0070:pep | . | 118 | 128 | 5.11e-05 | 0.737 | `FLLTSILSFKL` |
| 1 | PFI1805w:pep | . | 33 | 43 | 5.13e-05 | 0.737 | `TRNTRLLCECE` |
| 1 | PFI0755c:pep | . | 1334 | 1344 | 5.16e-05 | 0.737 | `NYYNRILFEEQ` |
| 1 | PF08\_0093:pep | . | 351 | 361 | 5.2e-05 | 0.737 | `FRNKHILYSKR` |
| 1 | PF14\_0649:pep | . | 441 | 451 | 5.2e-05 | 0.737 | `NNNKSQLHQLQ` |
| 1 | PFI0455w:pep | . | 450 | 460 | 5.23e-05 | 0.737 | `KREKRELQQKD` |
| 1 | PF11\_0090:pep | . | 445 | 455 | 5.23e-05 | 0.737 | `NEYKRIHIQPQ` |
| 1 | PFL1930w:pep | . | 4598 | 4608 | 5.24e-05 | 0.737 | `ILYKKLLSKIL` |
| 1 | PF14\_0715:pep | . | 302 | 312 | 5.25e-05 | 0.737 | `LLNKRNLHEII` |
| 1 | MAL8P1.141:pep | . | 17 | 27 | 5.26e-05 | 0.737 | `NRNNCYLHEEE` |
| 1 | PF13\_0010:pep | . | 80 | 90 | 5.27e-05 | 0.737 | `FTEGRVLAQCE` |
| 1 | PFI0130c:pep | . | 69 | 79 | 5.3e-05 | 0.737 | `NFHNRNLHETE` |
| 1 | PFD0680c:pep | . | 63 | 73 | 5.32e-05 | 0.737 | `NRLSKLSSEGK` |
| 1 | PFF1445c:pep | . | 516 | 526 | 5.32e-05 | 0.737 | `FINKGIQAETY` |
| 1 | PF13\_0149:pep | . | 50 | 60 | 5.33e-05 | 0.737 | `FLYSSLLKHKL` |
| 1 | PF14\_0733:pep | . | 92 | 102 | 5.33e-05 | 0.737 | `SRYSRGLCEIE` |
| 1 | MAL8P1.23:pep | . | 4503 | 4513 | 5.35e-05 | 0.737 | `FLFKRILPQFI` |
| 1 | PFI1100w:pep | . | 726 | 736 | 5.37e-05 | 0.737 | `INYKRILYKEQ` |
| 1 | PFC0750w:pep | . | 570 | 580 | 5.4e-05 | 0.737 | `DVLSRSNAHGL` |
| 1 | MAL7P1.147:pep | . | 1868 | 1878 | 5.4e-05 | 0.737 | `DANSNTLSKVL` |
| 1 | PF14\_0625:pep | . | 284 | 294 | 5.42e-05 | 0.737 | `ILLSRLLNPGK` |
| 1 | PFL2620w:pep | . | 44 | 54 | 5.47e-05 | 0.737 | `TIKSRRLAQTQ` |
| 1 | PFL2125c:pep | . | 500 | 510 | 5.51e-05 | 0.737 | `DLRSRIISLVL` |
| 1 | MAL7P1.207:pep | . | 355 | 365 | 5.55e-05 | 0.737 | `IKNKNDLHEEQ` |
| 1 | PFI0520w:pep | . | 531 | 541 | 5.55e-05 | 0.737 | `ILEKFSLQGKL` |
| 1 | PF14\_0614:pep | . | 39 | 49 | 5.59e-05 | 0.737 | `IKNERILDEYE` |
| 1 | MAL7P1.172:pep | . | 85 | 95 | 5.6e-05 | 0.737 | `NRYLRNLGEKS` |
| 1 | PFI1230c:pep | . | 97 | 107 | 5.66e-05 | 0.737 | `FLLVRLFSAVN` |
| 1 | PF10\_0099:pep | . | 479 | 489 | 5.68e-05 | 0.737 | `FRNSCILNEIV` |
| 1 | PFF0530w:pep | . | 225 | 235 | 5.69e-05 | 0.737 | `KDYKKILHEIE` |
| 1 | MAL8P1.97:pep | . | 508 | 518 | 5.69e-05 | 0.737 | `NNESIIFSEKL` |
| 1 | PF10\_0140:pep | . | 1752 | 1762 | 5.69e-05 | 0.737 | `LRNKRSLTSKD` |
| 1 | PFL2130w:pep | . | 387 | 397 | 5.7e-05 | 0.737 | `NRYKKILALLH` |
| 1 | MAL13P1.73:pep | . | 697 | 707 | 5.73e-05 | 0.737 | `NDNKDILHTEQ` |
| 1 | PFF1100c:pep | . | 1750 | 1760 | 5.75e-05 | 0.737 | `NAYKRILEKYE` |
| 1 | PFE0235c:pep | . | 4785 | 4795 | 5.82e-05 | 0.737 | `DLLYNTLYEKL` |
| 1 | PF11\_0303:pep | . | 300 | 310 | 5.82e-05 | 0.737 | `IRNTRVRAYKQ` |
| 1 | PF14\_0350:pep | . | 156 | 166 | 5.85e-05 | 0.737 | `EREKIILEQEE` |
| 1 | PF08\_0138:pep | . | 37 | 47 | 5.92e-05 | 0.737 | `IRAHRTLCECE` |
| 1 | PF14\_0148:pep | . | 30 | 40 | 5.95e-05 | 0.737 | `KRKKVILEEVE` |
| 1 | MAL13P1.122:pep | . | 6 | 16 | 6.04e-05 | 0.737 | `FKNKKILNEKV` |
| 1 | PFE1605w:pep | . | 83 | 93 | 6.06e-05 | 0.737 | `NRYSRKLCERF` |
| 1 | PFA0585w:pep | . | 30 | 40 | 6.14e-05 | 0.737 | `NRNNINFHETE` |
| 1 | PFE1330c:pep | . | 707 | 717 | 6.14e-05 | 0.737 | `DLLSYIKSLTE` |
| 1 | PFI0540w:pep | . | 918 | 928 | 6.14e-05 | 0.737 | `NRILSILNETE` |
| 1 | PFL1455w:pep | . | 49 | 59 | 6.15e-05 | 0.737 | `FGNIRALSCTE` |
| 1 | MAL13P1.148:pep | . | 183 | 193 | 6.17e-05 | 0.737 | `DIKKRSLIESQ` |
| 1 | MAL8P1.29:pep | . | 892 | 902 | 6.19e-05 | 0.737 | `NLLSKSYAEKP` |
| 1 | PF11\_0503:pep | . | 41 | 51 | 6.19e-05 | 0.737 | `HRNSRTLASAM` |
| 1 | PF14\_0278:pep | . | 1209 | 1219 | 6.19e-05 | 0.737 | `FCYKRDESETG` |
| 1 | PF10\_0195:pep | . | 53 | 63 | 6.23e-05 | 0.737 | `SRNKILLIQSE` |
| 1 | PF11\_0035:pep | . | 304 | 314 | 6.25e-05 | 0.737 | `EREKREKKEKE` |
| 1 | PF11\_0035:pep | . | 315 | 325 | 6.25e-05 | 0.737 | `EREKREKKEKE` |
| 1 | PF11\_0035:pep | . | 326 | 336 | 6.25e-05 | 0.737 | `EREKREKKEKE` |
| 1 | PF11\_0035:pep | . | 337 | 347 | 6.25e-05 | 0.737 | `EREKREKKEKE` |
| 1 | PF11\_0035:pep | . | 348 | 358 | 6.25e-05 | 0.737 | `EREKREKKEKE` |
| 1 | PF11\_0035:pep | . | 359 | 369 | 6.25e-05 | 0.737 | `EREKREKKEKE` |
| 1 | PF11\_0035:pep | . | 370 | 380 | 6.25e-05 | 0.737 | `EREKREKKEKE` |
| 1 | PFI1340w:pep | . | 144 | 154 | 6.27e-05 | 0.737 | `FLNKKHLKQLQ` |
| 1 | PFL0150w:pep | . | 277 | 287 | 6.28e-05 | 0.737 | `IQTKRTSHQSQ` |
| 1 | PF13\_0330:pep | . | 465 | 475 | 6.28e-05 | 0.737 | `SEEKRELHEKN` |
| 1 | PFD0320c:pep | . | 1046 | 1056 | 6.32e-05 | 0.737 | `FRNDKITLEKE` |
| 1 | PFD0320c:pep | . | 1193 | 1203 | 6.32e-05 | 0.737 | `FRNDKITLEKE` |
| 1 | PF10\_0183:pep | . | 1805 | 1815 | 6.32e-05 | 0.737 | `FLNKMNLTEEQ` |
| 1 | PF14\_0178:pep | . | 41 | 51 | 6.33e-05 | 0.737 | `NGNKIILPQTA` |
| 1 | PFA0665w:pep | . | 110 | 120 | 6.35e-05 | 0.737 | `YRIKRTTKEVE` |
| 1 | PF11\_0418:pep | . | 761 | 771 | 6.37e-05 | 0.737 | `TRKKRLQHEKK` |
| 1 | PF14\_0478:pep | . | 513 | 523 | 6.37e-05 | 0.737 | `KILKRIKGETQ` |
| 1 | MAL13P1.121:pep | . | 172 | 182 | 6.41e-05 | 0.737 | `DLLVNVLSGKE` |
| 1 | PF10\_0025:pep | . | 53 | 63 | 6.45e-05 | 0.737 | `VRCKRLLSEPA` |
| 1 | PFB0495w:pep | . | 466 | 476 | 6.47e-05 | 0.737 | `FLGKRIKTENE` |
| 1 | PFC0465c:pep | . | 551 | 561 | 6.48e-05 | 0.737 | `DRNKRNSSITV` |
| 1 | PFF0235c:pep | . | 1287 | 1297 | 6.52e-05 | 0.737 | `NKNKNILNEFE` |
| 1 | PF14\_0740:pep | . | 83 | 93 | 6.56e-05 | 0.737 | `RRLHRSLYEYQ` |
| 1 | PF14\_0758:pep | . | 87 | 97 | 6.56e-05 | 0.737 | `RRLHRSLYEFQ` |
| 1 | MAL13P1.164:pep | . | 8 | 18 | 6.58e-05 | 0.737 | `FLNSFTKSDKL` |
| 1 | PFA0430c:pep | . | 1050 | 1060 | 6.59e-05 | 0.737 | `LRNKVTNHDTE` |
| 1 | PF13\_0078:pep | . | 1578 | 1588 | 6.61e-05 | 0.737 | `NNNKRNLIYKE` |
| 1 | PFD0480w:pep | . | 184 | 194 | 6.65e-05 | 0.737 | `DRQKRIKYEDL` |
| 1 | PF07\_0055:pep | . | 227 | 237 | 6.71e-05 | 0.737 | `DREKRIKELQE` |
| 1 | PFL2365w:pep | . | 705 | 715 | 6.71e-05 | 0.737 | `DLNNVSESQGL` |
| 1 | PFB0895c:pep | . | 732 | 742 | 6.73e-05 | 0.737 | `NKNKRLLNELC` |
| 1 | PFF1370w:pep | . | 499 | 509 | 6.73e-05 | 0.737 | `NVYKRMLNEVI` |
| 1 | PF10\_0138:pep | . | 97 | 107 | 6.75e-05 | 0.737 | `DEEKRILHISG` |
| 1 | PF10\_0182:pep | . | 141 | 151 | 6.75e-05 | 0.737 | `NIYKYILASQQ` |
| 1 | PFF1265w:pep | . | 215 | 225 | 6.77e-05 | 0.737 | `LLYKRILACDT` |
| 1 | PF10\_0184:pep | . | 293 | 303 | 6.77e-05 | 0.737 | `PNNKIFLSETQ` |
| 1 | PF14\_0320:pep | . | 688 | 698 | 6.79e-05 | 0.737 | `NYNIRRLYEKE` |
| 1 | PF11\_0248:pep | . | 26 | 36 | 6.85e-05 | 0.737 | `FVSKRSLAPKV` |
| 1 | PF14\_0363:pep | . | 1266 | 1276 | 6.87e-05 | 0.737 | `INNKTILDITQ` |
| 1 | PF13\_0060:pep | . | 114 | 124 | 6.89e-05 | 0.737 | `EKEKRILREKA` |
| 1 | PFD1235w:pep | . | 155 | 165 | 6.91e-05 | 0.737 | `NLLVMARSEGE` |
| 1 | PFF0005c:pep | . | 154 | 164 | 6.91e-05 | 0.737 | `NLLVMARSEGE` |
| 1 | MAL8P1.207:pep | . | 155 | 165 | 6.91e-05 | 0.737 | `NLLVMARSEGE` |
| 1 | MAL13P1.274:pep | . | 531 | 541 | 6.91e-05 | 0.737 | `DRNKEPLDEGV` |
| 1 | PFB0105c:pep | . | 80 | 90 | 6.93e-05 | 0.737 | `KRNSRKFAEGY` |
| 1 | PFL2420w:pep | . | 351 | 361 | 6.93e-05 | 0.737 | `ISNKKILKETD` |
| 1 | PFB0150c:pep | . | 2193 | 2203 | 6.98e-05 | 0.737 | `DLLVYILDDVL` |
| 1 | MAL13P1.157:pep | . | 173 | 183 | 6.98e-05 | 0.737 | `FRSVYFLEQVQ` |
| 1 | PFL0485w:pep | . | 27 | 37 | 7.02e-05 | 0.737 | `IENKYILDQEE` |
| 1 | PFA0245w:pep | . | 356 | 366 | 7.04e-05 | 0.737 | `DSNKNILHDEE` |
| 1 | PF07\_0113:pep | . | 157 | 167 | 7.04e-05 | 0.737 | `LLNKRICHLFE` |
| 1 | PF14\_0315:pep | . | 2341 | 2351 | 7.06e-05 | 0.737 | `FSSKRSLSESK` |
| 1 | PFB0295w:pep | . | 16 | 26 | 7.09e-05 | 0.737 | `GRYKRSCQEVS` |
| 1 | PF10\_0313a:pep | . | 355 | 365 | 7.09e-05 | 0.737 | `IIYKTILHQIY` |
| 1 | PF14\_0019:pep | . | 49 | 59 | 7.09e-05 | 0.737 | `IKEKRELGERE` |
| 1 | PF14\_0225:pep | . | 315 | 325 | 7.09e-05 | 0.737 | `FLFQRIRAENE` |
| 1 | PF14\_0440:pep | . | 691 | 701 | 7.11e-05 | 0.737 | `NRYKTVLYEKY` |
| 1 | PF14\_0736:pep | . | 43 | 53 | 7.15e-05 | 0.739 | `VRNSRILSGPR` |
| 1 | PFC0770c:pep | . | 536 | 546 | 7.19e-05 | 0.739 | `KKNKEILLEKE` |
| 1 | MAL13P1.265:pep | . | 45 | 55 | 7.19e-05 | 0.739 | `KYNKDILNEKE` |
| 1 | PFD0090c:pep | . | 65 | 75 | 7.25e-05 | 0.744 | `NVYLRNLSELQ` |
| 1 | PF11\_0130:pep | . | 43 | 53 | 7.3e-05 | 0.745 | `DRNKLILLNKK` |
| 1 | MAL13P1.390:pep | . | 529 | 539 | 7.33e-05 | 0.745 | `DINKRILSLQG` |
| 1 | PFC0840w:pep | . | 834 | 844 | 7.35e-05 | 0.745 | `FYNKNSLANYE` |
| 1 | PFL1200c:pep | . | 3 | 13 | 7.35e-05 | 0.745 | `RRNIRLPAEVS` |
| 1 | PFL1975c:pep | . | 96 | 106 | 7.42e-05 | 0.745 | `NDGKRLLTGTQ` |
| 1 | PFL0895c:pep | . | 559 | 569 | 7.44e-05 | 0.745 | `FRNNNTDEQVQ` |
| 1 | PF11\_0240:pep | . | 3886 | 3896 | 7.47e-05 | 0.745 | `DLEEHLLSITL` |
| 1 | PF13\_0069:pep | . | 104 | 114 | 7.47e-05 | 0.745 | `FKYKPKLVQVE` |
| 1 | PFL1455w:pep | . | 957 | 967 | 7.52e-05 | 0.745 | `FANERTLLQWL` |
| 1 | PF11\_0422:pep | . | 928 | 938 | 7.57e-05 | 0.745 | `NLPKRILHNDS` |
| 1 | PF11\_0469:pep | . | 329 | 339 | 7.57e-05 | 0.745 | `FRNSNGLVEKN` |
| 1 | PF13\_0257:pep | . | 29 | 39 | 7.57e-05 | 0.745 | `NKNKKILEKTE` |
| 1 | MAL13P1.313:pep | . | 333 | 343 | 7.57e-05 | 0.745 | `NNYKEILNEYE` |
| 1 | PFF0965c:pep | . | 856 | 866 | 7.59e-05 | 0.745 | `TRYKYIYHEIL` |
| 1 | PFI0115c:pep | . | 45 | 55 | 7.59e-05 | 0.745 | `FNNSRYLSEYS` |
| 1 | PF14\_0479:pep | . | 1391 | 1401 | 7.59e-05 | 0.745 | `FVNARTLEECL` |
| 1 | PFI1340w:pep | . | 255 | 265 | 7.64e-05 | 0.747 | `NIKKEKLHEVQ` |
| 1 | PFL0060w:pep | . | 81 | 91 | 7.67e-05 | 0.747 | `FNNSRKLADVS` |
| 1 | PF14\_0399:pep | . | 14 | 24 | 7.67e-05 | 0.747 | `KRNLRILILGL` |
| 1 | MAL13P1.140:pep | . | 2119 | 2129 | 7.75e-05 | 0.749 | `DNTKRDIHQGE` |
| 1 | PF14\_0282:pep | . | 274 | 284 | 7.78e-05 | 0.749 | `VRYAVILSEAE` |
| 1 | MAL7P1.310:pep | . | 42 | 52 | 7.8e-05 | 0.749 | `PTNFRSLAEVS` |
| 1 | PFL2565w:pep | . | 42 | 52 | 7.8e-05 | 0.749 | `NIYSRNLAQLK` |
| 1 | PF10\_0125:pep | . | 665 | 675 | 7.83e-05 | 0.749 | `FLNKLIYHDIQ` |
| 1 | PF10\_0374:pep | . | 666 | 676 | 7.86e-05 | 0.749 | `KEYKEILHEGK` |
| 1 | PF13\_0069:pep | . | 22 | 32 | 7.86e-05 | 0.749 | `FREKLIKGRKQ` |
| 1 | PFB0615c:pep | . | 468 | 478 | 7.89e-05 | 0.749 | `DKNKRSYIQTT` |
| 1 | PFA0625w:pep | . | 1292 | 1302 | 7.91e-05 | 0.749 | `DNNSRNLLEDE` |
| 1 | PFD0110w:pep | . | 2280 | 2290 | 7.91e-05 | 0.749 | `DDYKDILIQNE` |
| 1 | PFE0230w:pep | . | 1472 | 1482 | 7.97e-05 | 0.749 | `LREKERLREKE` |
| 1 | PFE1120w:pep | . | 1719 | 1729 | 7.99e-05 | 0.749 | `DKNKYILDSTE` |
| 1 | PF11\_0035:pep | . | 44 | 54 | 7.99e-05 | 0.749 | `DSYIRILCEKH` |
| 1 | PF11\_0259:pep | . | 65 | 75 | 8.02e-05 | 0.749 | `PRYSRIPKEKK` |
| 1 | PF14\_0247:pep | . | 487 | 497 | 8.02e-05 | 0.749 | `DREKRNNHTKN` |
| 1 | PF14\_0536:pep | . | 329 | 339 | 8.02e-05 | 0.749 | `NNDPNILAETE` |
| 1 | PFB0501c:pep | . | 123 | 133 | 8.05e-05 | 0.749 | `IINKRLRHEQY` |
| 1 | PFD0445c:pep | . | 383 | 393 | 8.05e-05 | 0.749 | `DKVKRSEHEPE` |
| 1 | PF14\_0464:pep | . | 57 | 67 | 8.11e-05 | 0.752 | `FIEIRNLSEEE` |
| 1 | PF11\_0416:pep | . | 1037 | 1047 | 8.14e-05 | 0.752 | `KNNKRKLSVKE` |
| 1 | PF14\_0342:pep | . | 999 | 1009 | 8.17e-05 | 0.752 | `NQKKHILNEKQ` |
| 1 | MAL8P1.141:pep | . | 568 | 578 | 8.2e-05 | 0.752 | `NLNIRILHHEI` |
| 1 | PF11\_0420:pep | . | 115 | 125 | 8.2e-05 | 0.752 | `NLNIRILHILC` |
| 1 | PF14\_0183b:pep | . | 158 | 168 | 8.22e-05 | 0.752 | `LLNKHIYAQAQ` |
| 1 | PF07\_0004:pep | . | 351 | 361 | 8.25e-05 | 0.752 | `DPLKRDATQTQ` |
| 1 | PFI1545c:pep | . | 100 | 110 | 8.25e-05 | 0.752 | `NRKKGRFHEGE` |
| 1 | PFF1365c:pep | . | 6570 | 6580 | 8.28e-05 | 0.752 | `LRNKELLGEKN` |
| 1 | PFD1150c:pep | . | 1451 | 1461 | 8.31e-05 | 0.752 | `ITNKRKNSETN` |
| 1 | PFE1595c:pep | . | 50 | 60 | 8.34e-05 | 0.752 | `FRNTFSNHEKK` |
| 1 | PF10\_0078:pep | . | 300 | 310 | 8.34e-05 | 0.752 | `KRNKKSLSEEN` |
| 1 | PFL0130c:pep | . | 1551 | 1561 | 8.37e-05 | 0.752 | `FKNKDISSELE` |
| 1 | PF10\_0161a:pep | . | 64 | 74 | 8.43e-05 | 0.752 | `MKKSRILSEYQ` |
| 1 | PFE0055c:pep | . | 388 | 398 | 8.46e-05 | 0.752 | `PEQKRTLKETL` |
| 1 | PFF1440w:pep | . | 3611 | 3621 | 8.46e-05 | 0.752 | `NTNPRILSGTD` |
| 1 | PF14\_0096:pep | . | 115 | 125 | 8.46e-05 | 0.752 | `LHGKRTLCETL` |
| 1 | PFD1235w:pep | . | 357 | 367 | 8.49e-05 | 0.752 | `IRNKDILSDNP` |
| 1 | MAL8P1.207:pep | . | 357 | 367 | 8.49e-05 | 0.752 | `IRNKDILSDNP` |
| 1 | PFL0405w:pep | . | 1749 | 1759 | 8.49e-05 | 0.752 | `FDEYLNLHQKQ` |
| 1 | PFD0685c:pep | . | 225 | 235 | 8.59e-05 | 0.759 | `INYKNIYEETQ` |
| 1 | PFL0115w:pep | . | 1914 | 1924 | 8.62e-05 | 0.761 | `DLLSNSDTETL` |
| 1 | PFE1515w:pep | . | 483 | 493 | 8.68e-05 | 0.762 | `KNNKRSDDEKE` |
| 1 | MAL7P1.59:pep | . | 124 | 134 | 8.68e-05 | 0.762 | `IRNSIILRQCK` |
| 1 | PFB0106c:pep | . | 98 | 108 | 8.71e-05 | 0.763 | `IKYNRSLAELK` |
| 1 | PFL1645w:pep | . | 1934 | 1944 | 8.84e-05 | 0.77 | `PLYKRYLVEKR` |
| 1 | MAL13P1.243:pep | . | 13 | 23 | 8.87e-05 | 0.77 | `IRNICILAHVD` |
| 1 | PFB0505c:pep | . | 174 | 184 | 8.91e-05 | 0.77 | `NRYKNILIVGS` |
| 1 | MAL13P1.257:pep | . | 3 | 13 | 8.91e-05 | 0.77 | `NTVVRIKAELE` |
| 1 | MAL13P1.13:pep | . | 1901 | 1911 | 8.94e-05 | 0.77 | `EIAKRILNEGL` |
| 1 | MAL8P1.9:pep | . | 75 | 85 | 8.97e-05 | 0.77 | `DVDKNILHQKI` |
| 1 | PFB0560w:pep | . | 2410 | 2420 | 9.01e-05 | 0.77 | `FLTKRIEEEVF` |
| 1 | PF13\_0219:pep | . | 603 | 613 | 9.01e-05 | 0.77 | `NKNKGILHLKN` |
| 1 | PFB0227c:pep | . | 283 | 293 | 9.04e-05 | 0.77 | `KRLRRISHFKE` |
| 1 | MAL7P1.167:pep | . | 277 | 287 | 9.04e-05 | 0.77 | `ILNKRINLEHY` |
| 1 | PF14\_0142:pep | . | 44 | 54 | 9.04e-05 | 0.77 | `FLNQPILLELE` |
| 1 | PFE0070w:pep | . | 111 | 121 | 9.11e-05 | 0.77 | `FEEKFNLASVQ` |
| 1 | PF14\_0479:pep | . | 796 | 806 | 9.11e-05 | 0.77 | `IRNNALKHQDQ` |
| 1 | PF14\_0607:pep | . | 676 | 686 | 9.11e-05 | 0.77 | `FYNIRLKAEVI` |
| 1 | PFA0690w:pep | . | 3 | 13 | 9.17e-05 | 0.77 | `FFYVRILYVVI` |
| 1 | PFI0665w:pep | . | 54 | 64 | 9.17e-05 | 0.77 | `ELNKQINEETQ` |
| 1 | PF10\_0313a:pep | . | 565 | 575 | 9.17e-05 | 0.77 | `FLYKRKIKNKQ` |
| 1 | PFA0670c:pep | . | 48 | 58 | 9.21e-05 | 0.77 | `LKNSRWLSETS` |
| 1 | PF07\_0003:pep | . | 46 | 56 | 9.21e-05 | 0.77 | `IPNIRLLCECE` |
| 1 | PFL0815w:pep | . | 420 | 430 | 9.21e-05 | 0.77 | `AENKRIKLEKL` |
| 1 | PFC1015c:pep | . | 1690 | 1700 | 9.25e-05 | 0.77 | `YLNKIILHFYE` |
| 1 | MAL8P1.154:pep | . | 2009 | 2019 | 9.25e-05 | 0.77 | `FPIKRLLFAGE` |
| 1 | PFL0470w:pep | . | 463 | 473 | 9.32e-05 | 0.774 | `NHVVGGLHQTQ` |
| 1 | PFF0255c:pep | . | 26 | 36 | 9.43e-05 | 0.781 | `FRENLILDKKE` |
| 1 | MAL8P1.108:pep | . | 70 | 80 | 9.47e-05 | 0.781 | `MFLKRNLAQEL` |
| 1 | PFL2560c:pep | . | 45 | 55 | 9.47e-05 | 0.781 | `LIIKRRLSETL` |
| 1 | PFD0225w:pep | . | 1592 | 1602 | 9.51e-05 | 0.781 | `NNNKSILLEGT` |
| 1 | PF14\_0217:pep | . | 170 | 180 | 9.51e-05 | 0.781 | `DRYNDTLYENE` |
| 1 | MAL7P1.171:pep | . | 83 | 93 | 9.58e-05 | 0.784 | `IRPSRSLTERK` |
| 1 | MAL13P1.122:pep | . | 312 | 322 | 9.58e-05 | 0.784 | `YLNKRIFHNKS` |
| 1 | PFC0905c:pep | . | 2330 | 2340 | 9.62e-05 | 0.784 | `FRNSYILLNGN` |
| 1 | MAL13P1.170:pep | . | 574 | 584 | 9.62e-05 | 0.784 | `FLHSRNLNETY` |
| 1 | PFD0225w:pep | . | 734 | 744 | 9.66e-05 | 0.784 | `INNKVILFEKP` |
| 1 | PFL1400c:pep | . | 690 | 700 | 9.66e-05 | 0.784 | `NRNKYLKDELE` |
| 1 | PFC0640w:pep | . | 1888 | 1898 | 9.7e-05 | 0.785 | `KRNKKILTRKL` |
| 1 | PF10\_0246:pep | . | 575 | 585 | 9.78e-05 | 0.786 | `NNNNIINHQKQ` |
| 1 | PFL0700w:pep | . | 49 | 59 | 9.78e-05 | 0.786 | `DNSKFILSELQ` |
| 1 | PF14\_0607:pep | . | 47 | 57 | 9.78e-05 | 0.786 | `KLNNRILFEGS` |
| 1 | PFF0575c:pep | . | 1666 | 1676 | 9.82e-05 | 0.786 | `NMNKNILSEDI` |
| 1 | MAL7P1.95:pep | . | 49 | 59 | 9.82e-05 | 0.786 | `REYKRITSQKI` |
| 1 | PF14\_0626:pep | . | 4662 | 4672 | 9.85e-05 | 0.787 | `IRISSVEAETQ` |
| 1 | PFL2535w:pep | . | 81 | 91 | 9.93e-05 | 0.792 | `EKYSRTLFETN` |
| 1 | PF10\_0406:pep | . | 1311 | 1321 | 9.96e-05 | 0.793 | `IKKKRTEFEKQ` |

---

**DEBUGGING INFORMATION**


---

Command line:

```
fimo --oc Hiller2004.1.fimo.out Hiller2004.1.out/meme.html /Users/t.bailey/Genomes_local/Pf276v1.24/Plasmodium_falciparum.ASM276v1.24.pep.all.fa
```

Settings:

```
|  |  |  |
| --- | --- | --- |
| output directory = Hiller2004.1.fimo.out | MEME file name = Hiller2004.1.out/meme.html | sequence file name = /Users/t.bailey/Genomes_local/Pf276v1.24/Plasmodium_falciparum.ASM276v1.24.pep.all.fa |
| allow clobber = true | compute q-values = true | parse genomic coord. = false |
| text only = false | scan both strands = false | output threshold = 0.0001 |
| threshold type = p-value | max stored scores = 100000 | pseudocount = 0.1 |
| verbosity = 2 |  |  |
```

This information can be useful in the event you wish to report a
problem with the FIMO software.

---

**Go to top**
