# Supplementary material for: The MEME Suite
Source: Nucleic Acids Res. 2015 May 7;43(Web Server issue):W39–49. doi: 10.1093/nar/gkv416 (PMC4489269; doi:10.1093/nar/gkv416)
Supplement: SUPPLEMENTARY DATA [file supp_gkv416_nar-00283-web-b-2015-File002.zip › case1/Hiller2004.1.mast.out/mast.html]

MAST


The name of the sequence database file.

[close ]

The number of sequences in the database.

[close ]

The number of residues in the sequence database.

[close ]

The date of the last modification to the sequence database.

[close ]

The name of the motif. If the motif has been removed or removal is
recommended to avoid highly similar motifs then it will be displayed
in red text.

[close ]

The width of the motif. No gaps are allowed in motifs supplied to MAST
as it only works for motifs of a fixed width.

[close ]

The sequence that would achieve the best possible match score and its
reverse complement for nucleotide motifs.

[close ]

MAST computes the pairwise correlations between each pair of motifs.
The correlation between two motifs is the maximum sum of Pearson's
correlation coefficients for aligned columns divided by the width of
the shorter motif. The maximum is found by trying all alignments of the
two motifs. Motifs with correlations below 0.60 have little effect on
the accuracy of the combined scores. Pairs of motifs with higher
correlations should be removed from the query. Correlations above the
supplied threshold are shown in red text.

[close ]

This diagram shows the normal spacing of the motifs specified to MAST.

[close ]

The name of the sequence. This maybe be linked to search a sequence database for the sequence name.

[close ]

The *E*-value of the sequence. For DNA
only; if strands were scored seperately then there will be two
*E*-values for the sequence seperated by a "/". The score for the
provided sequence will be first and the score for the reverse-complement
will be second.

[close ]

The block diagram shows the best non-overlapping tiling of motif matches on the sequence.

- The length of the line shows the length of a sequence relative to all the other sequences.
- A block is shown where the positional *p*-value
  of a motif is less (more significant) than the significance threshold which is 0.0001 by default.
- If a significant motif match (as specified above) overlaps other significant motif matches then
  it is only displayed as a block if its positional *p*-value
  is less (more significant) then the product of the positional
  *p*-values of the significant matches that it overlaps.
- The position of a block shows where a motif has matched the sequence.
- The width of a block shows the width of the motif relative to the length of the sequence.
- The colour and border of a block identifies the matching motif as in the legend.
- The height of a block gives an indication of the significance of the match as
  taller blocks are more significant. The height is calculated to be proportional
  to the negative logarithm of the positional *p*-value,
  truncated at the height for a *p*-value of 1e-10.
- Hovering the mouse cursor over the block causes the display of the motif name
  and other details in the hovering text.
- DNA only; blocks displayed above the line are a match on the given DNA, whereas blocks
  displayed below the line are matches to the reverse-complement of the given DNA.
- DNA only; when strands are scored separately then blocks may overlap on opposing strands.

[close ]

The description appearing after the identifier in the fasta file used to specify the sequence.

[close ]

The combined *p*-value of the
sequence. DNA only; if strands were scored seperately then there will be
two *p*-values for the sequence seperated by a "/". The score for
the provided sequence will be first and the score for the
reverse-complement will be second.

[close ]

This indicates the offset used for translation of the DNA.

[close ]

The annotated sequence shows a portion of the sequence with the
matching motif sequences displayed above. The displayed portion of the
sequence can be modified by sliding the two buttons below the sequence
block diagram so that the portion you want to see is between the two
needles attached to the buttons. By default the two buttons move
together but you can drag one individually by holding shift before you
start the drag. If the strands were scored seperately then they can't
be both displayed at once due to overlaps and so a radio button offers
the choice of strand to display.

[close ]

# MAST

## Motif Alignment & Search Tool

For further information on how to interpret these results or to get a
copy of the MEME software please access
http://meme.nbcr.net.

If you use MAST in your research, please cite the following paper:  
Timothy L. Bailey and Michael Gribskov,
"Combining evidence using p-values: application to sequence homology searches",
*Bioinformatics*, **14**(1):48-54, 1998.
[pdf]

Inputs
  |  
Search Results
  |  
Program information
  |  
Explanation

|  |  |
| --- | --- |
| Inputs | Top |

#### Sequence Databases

The following sequence database was supplied to MAST.

| Database | Sequence Count | Residue Count | Last Modified |
| --- | --- | --- | --- |
| /Users/t.bailey/Genomes\_local/Pf276v1.24/Plasmodium\_falciparum.ASM276v1.24.pep.all.fa | 5414 | 4115585 | Thu Dec 18 10:56:09 2014 |
|  |  |  |  |
| --- | --- | --- | --- |
| Total | 5414 | 4115585 |  |

#### Motifs

The following motifs were supplied to MAST from "Hiller2004.1.out/meme.html" last modified on Fri Dec 19 17:23:03 2014.

|  |  |  | Similarity |
| --- | --- | --- | --- |
| Motif | Width | Best possible match | 1 |
| 1 | 11 | FRNKRILAETQ | - |

|  |  |
| --- | --- |
| Search Results | Top |

#### Top Scoring Sequences

Each of the following 264 sequences has an *E*-value less than
2.5e+02.  
The motif matches shown have a position p-value less than 0.0001.  
**Click on the arrow** (↧) next to the *E*-value to view more information about a sequence.

|  |  |
| --- | --- |
|  | Motif 1 |

Sequence | *E*-value |  | Block Diagram ||  |  |  |  |
| --- | --- | --- | --- |
|  | | | 0          1000          2000          3000          4000          5000          6000          7000          8000          9000 |
| PF10\_0159:pep | 5.8e-05 | ↧ |  |
| PFB0100c:pep | 0.00013 | ↧ |  |
| PFE0040c:pep | 0.004 | ↧ |  |
| PF14\_0010:pep | 0.0088 | ↧ |  |
| PFA0660w:pep | 0.1 | ↧ |  |
| PFB0095c:pep | 0.18 | ↧ |  |
| MAL7P1.170:pep | 0.2 | ↧ |  |
| MAL7P1.231:pep | 0.27 | ↧ |  |
| PF07\_0004:pep | 0.29 | ↧ |  |
| PF14\_0031b:pep | 0.42 | ↧ |  |
| MAL13P1.61:pep | 0.66 | ↧ |  |
| PF10\_0024:pep | 0.91 | ↧ |  |
| PF11\_0504:pep | 0.95 | ↧ |  |
| PFD1170c:pep | 0.96 | ↧ |  |
| PFB0070w:pep | 1 | ↧ |  |
| PFA0610c:pep | 1.1 | ↧ |  |
| PFI0080w:pep | 1.3 | ↧ |  |
| PFB0065w:pep | 1.3 | ↧ |  |
| PF10\_0374:pep | 1.4 | ↧ |  |
| PFI1750c:pep | 1.7 | ↧ |  |
| MAL7P1.7:pep | 1.8 | ↧ |  |
| MAL7P1.223:pep | 1.9 | ↧ |  |
| PFB0932w:pep | 2.1 | ↧ |  |
| PFL2545c:pep | 2.1 | ↧ |  |
| MAL13P1.490:pep | 2.5 | ↧ |  |
| PFE0385w:pep | 2.8 | ↧ |  |
| PFI1785w:pep | 3.1 | ↧ |  |
| PFE0050w:pep | 4.5 | ↧ |  |
| MAL13P1.480:pep | 5.1 | ↧ |  |
| PF13\_0076:pep | 5.1 | ↧ |  |
| PFC0055w:pep | 6.8 | ↧ |  |
| PF10\_0161a:pep | 7 | ↧ |  |
| PFD0670c:pep | 7.1 | ↧ |  |
| PFD0035c:pep | 7.2 | ↧ |  |
| PF14\_0007:pep | 7.3 | ↧ |  |
| PFC0025c:pep | 7.4 | ↧ |  |
| PF14\_0767:pep | 7.5 | ↧ |  |
| MAL8P1.217:pep | 7.5 | ↧ |  |
| PFA0750w:pep | 7.5 | ↧ |  |
| PFL2635w:pep | 7.6 | ↧ |  |
| PFF1550w:pep | 7.6 | ↧ |  |
| PFB0025c:pep | 7.6 | ↧ |  |
| PF14\_0771:pep | 7.6 | ↧ |  |
| PF10\_0395:pep | 7.6 | ↧ |  |
| PFA0705c:pep | 7.7 | ↧ |  |
| PF11\_0516:pep | 7.7 | ↧ |  |
| PF14\_0733:pep | 7.7 | ↧ |  |
| PFI1805w:pep | 9.3 | ↧ |  |
| PFF0700c:pep | 11 | ↧ |  |
| MAL8P1.4:pep | 11 | ↧ |  |
| PFI1525w:pep | 11 | ↧ |  |
| MAL7P1.174:pep | 12 | ↧ |  |
| MAL7P1.177:pep | 12 | ↧ |  |
| PF10\_0020:pep | 14 | ↧ |  |
| PFI0145w:pep | 15 | ↧ |  |
| PFA0090c:pep | 16 | ↧ |  |
| PFL2610w:pep | 17 | ↧ |  |
| PF07\_0130:pep | 17 | ↧ |  |
| PFI0045c:pep | 17 | ↧ |  |
| PFC1105w:pep | 17 | ↧ |  |
| PF13\_0010:pep | 17 | ↧ |  |
| PFB1020w:pep | 17 | ↧ |  |
| MAL13P1.7:pep | 18 | ↧ |  |
| PFD1220c:pep | 21 | ↧ |  |
| PF14\_0753:pep | 22 | ↧ |  |
| MAL13P1.75:pep | 24 | ↧ |  |
| MAL8P1.3:pep | 24 | ↧ |  |
| PFA0670c:pep | 24 | ↧ |  |
| MAL7P1.58:pep | 26 | ↧ |  |
| PFB0715w:pep | 27 | ↧ |  |
| PFB0310c:pep | 27 | ↧ |  |
| MAL7P1.225.1 | 29 | ↧ |  |
| MAL7P1.225.2 | 29 | ↧ |  |
| PFL1200c:pep | 31 | ↧ |  |
| PF10\_0025:pep | 33 | ↧ |  |
| PFI0910w:pep | 33 | ↧ |  |
| PFL1815c:pep | 34 | ↧ |  |
| PF14\_0019:pep | 35 | ↧ |  |
| PF13\_0220:pep | 39 | ↧ |  |
| PF14\_0744:pep | 39 | ↧ |  |
| PFB0955w:pep | 41 | ↧ |  |
| PF11\_0503:pep | 42 | ↧ |  |
| MAL7P1.106:pep | 42 | ↧ |  |
| PFB0090c:pep | 43 | ↧ |  |
| PF14\_0464:pep | 44 | ↧ |  |
| PF10\_0085:pep | 45 | ↧ |  |
| PF14\_0736:pep | 47 | ↧ |  |
| MAL13P1.264:pep | 50 | ↧ |  |
| MAL13P1.329:pep | 51 | ↧ |  |
| PFC0255c:pep | 52 | ↧ |  |
| PF13\_0055:pep | 53 | ↧ |  |
| PFL2620w:pep | 55 | ↧ |  |
| PF11\_0248:pep | 55 | ↧ |  |
| PF08\_0063:pep | 55 | ↧ |  |
| PF10\_0070:pep | 55 | ↧ |  |
| PFB0930w:pep | 56 | ↧ |  |
| PFC0045w:pep | 56 | ↧ |  |
| PFL2560c:pep | 57 | ↧ |  |
| PFI0055c:pep | 58 | ↧ |  |
| PF11\_0203:pep | 58 | ↧ |  |
| PFI0320w:pep | 58 | ↧ |  |
| PF11\_0038:pep | 58 | ↧ |  |
| PF08\_0138:pep | 59 | ↧ |  |
| PFL2625w:pep | 59 | ↧ |  |
| MAL7P1.160.1 | 63 | ↧ |  |
| MAL7P1.160.2 | 64 | ↧ |  |
| PF14\_0281:pep | 64 | ↧ |  |
| PFB0875c:pep | 64 | ↧ |  |
| PFB0106c:pep | 65 | ↧ |  |
| PFI1565w:pep | 67 | ↧ |  |
| PFD1215w:pep | 67 | ↧ |  |
| PFB0886c:pep | 69 | ↧ |  |
| PFL2550w:pep | 70 | ↧ |  |
| PFI1795c:pep | 72 | ↧ |  |
| PF14\_0399:pep | 72 | ↧ |  |
| PF11\_0041:pep | 73 | ↧ |  |
| PFD1225w:pep | 76 | ↧ |  |
| PFE0030c:pep | 77 | ↧ |  |
| PFF1195c:pep | 79 | ↧ |  |
| PFC1100w:pep | 80 | ↧ |  |
| PF10\_0017:pep | 81 | ↧ |  |
| PFD1185w:pep | 83 | ↧ |  |
| PFL0060w:pep | 83 | ↧ |  |
| PFL2590w:pep | 83 | ↧ |  |
| PF14\_0763:pep | 83 | ↧ |  |
| MAL13P1.16:pep | 87 | ↧ |  |
| PF14\_0743:pep | 88 | ↧ |  |
| MAL7P1.227:pep | 89 | ↧ |  |
| PF10\_0015:pep | 92 | ↧ |  |
| PFL0765w:pep | 93 | ↧ |  |
| PFB0555c:pep | 93 | ↧ |  |
| PFC0085c:pep | 98 | ↧ |  |
| PFD0150w:pep | 1e+02 | ↧ |  |
| PFB0415c:pep | 1e+02 | ↧ |  |
| PF13\_0079:pep | 1.1e+02 | ↧ |  |
| PF14\_0515:pep | 1.1e+02 | ↧ |  |
| PFB0105c:pep | 1.1e+02 | ↧ |  |
| PF14\_0739a:pep | 1.1e+02 | ↧ |  |
| PFB0605w:pep | 1.1e+02 | ↧ |  |
| PF10\_0160:pep | 1.1e+02 | ↧ |  |
| PF07\_0088:pep | 1.2e+02 | ↧ |  |
| PFA0615w:pep | 1.2e+02 | ↧ |  |
| PF14\_0096:pep | 1.2e+02 | ↧ |  |
| PF13\_0060:pep | 1.2e+02 | ↧ |  |
| MAL13P1.62:pep | 1.2e+02 | ↧ |  |
| PFL2645c:pep | 1.2e+02 | ↧ |  |
| PF11\_0305:pep | 1.2e+02 | ↧ |  |
| PFI0020w:pep | 1.2e+02 | ↧ |  |
| PF13\_0004:pep | 1.2e+02 | ↧ |  |
| PFA0310c:pep | 1.3e+02 | ↧ |  |
| PF07\_0003:pep | 1.3e+02 | ↧ |  |
| PF10\_0404:pep | 1.3e+02 | ↧ |  |
| PFL1660c:pep | 1.3e+02 | ↧ |  |
| PFA0745w:pep | 1.3e+02 | ↧ |  |
| PF14\_0148:pep | 1.3e+02 | ↧ |  |
| MAL7P1.171:pep | 1.3e+02 | ↧ |  |
| PFL2525c:pep | 1.3e+02 | ↧ |  |
| PF10\_0401:pep | 1.3e+02 | ↧ |  |
| PF14\_0013:pep | 1.3e+02 | ↧ |  |
| PF07\_0054:pep | 1.3e+02 | ↧ |  |
| PFF0025w:pep | 1.3e+02 | ↧ |  |
| PFF1565c:pep | 1.4e+02 | ↧ |  |
| PFI0065w:pep | 1.4e+02 | ↧ |  |
| PF10\_0402:pep | 1.4e+02 | ↧ |  |
| PF10\_0120:pep | 1.4e+02 | ↧ |  |
| PFB0055c:pep | 1.4e+02 | ↧ |  |
| PF11\_0259:pep | 1.4e+02 | ↧ |  |
| PFL2535w:pep | 1.4e+02 | ↧ |  |
| MAL8P1.108:pep | 1.4e+02 | ↧ |  |
| PF10\_0271:pep | 1.4e+02 | ↧ |  |
| PFD0070c.2 | 1.5e+02 | ↧ |  |
| PFD0070c.1 | 1.5e+02 | ↧ |  |
| PF10\_0337:pep | 1.5e+02 | ↧ |  |
| PFD1200c:pep | 1.5e+02 | ↧ |  |
| PFE0145w:pep | 1.5e+02 | ↧ |  |
| MAL8P1.219:pep | 1.5e+02 | ↧ |  |
| PFB0110w:pep | 1.5e+02 | ↧ |  |
| PF14\_0003:pep | 1.5e+02 | ↧ |  |
| PFB1040w:pep | 1.5e+02 | ↧ |  |
| PFL2655w:pep | 1.5e+02 | ↧ |  |
| PFD0480w:pep | 1.5e+02 | ↧ |  |
| PF08\_0054:pep | 1.5e+02 | ↧ |  |
| PF14\_0768:pep | 1.6e+02 | ↧ |  |
| PF14\_0615a:pep | 1.6e+02 | ↧ |  |
| PFE1605w:pep | 1.6e+02 | ↧ |  |
| PFC0080c:pep | 1.6e+02 | ↧ |  |
| PF11\_0134:pep | 1.6e+02 | ↧ |  |
| PF10\_0344:pep | 1.6e+02 | ↧ |  |
| PFI1025w:pep | 1.7e+02 | ↧ |  |
| PF11\_0303:pep | 1.7e+02 | ↧ |  |
| PFI1790w:pep | 1.7e+02 | ↧ |  |
| PF11\_0485:pep | 1.7e+02 | ↧ |  |
| MAL13P1.338:pep | 1.7e+02 | ↧ |  |
| PF11\_0510:pep | 1.7e+02 | ↧ |  |
| MAL7P1.310:pep | 1.7e+02 | ↧ |  |
| PF10\_0265:pep | 1.7e+02 | ↧ |  |
| PFL0050c:pep | 1.7e+02 | ↧ |  |
| PF08\_0064:pep | 1.7e+02 | ↧ |  |
| PFC0800w:pep | 1.7e+02 | ↧ |  |
| PF11\_0035:pep | 1.8e+02 | ↧ |  |
| PF14\_0643:pep | 1.8e+02 | ↧ |  |
| PFL1855w:pep | 1.8e+02 | ↧ |  |
| PF14\_0350:pep | 1.8e+02 | ↧ |  |
| PFE1095w:pep | 1.8e+02 | ↧ |  |
| PF13\_0038:pep | 1.8e+02 | ↧ |  |
| MAL7P1.218:pep | 1.8e+02 | ↧ |  |
| PF14\_0018:pep | 1.8e+02 | ↧ |  |
| PF13\_0169:pep | 1.8e+02 | ↧ |  |
| PFC0675c:pep | 1.9e+02 | ↧ |  |
| PFI0086w:pep | 1.9e+02 | ↧ |  |
| PFD0125c:pep | 1.9e+02 | ↧ |  |
| PF14\_0142:pep | 1.9e+02 | ↧ |  |
| PFA0640c:pep | 1.9e+02 | ↧ |  |
| PF10\_0140:pep | 1.9e+02 | ↧ |  |
| PFL0700w:pep | 1.9e+02 | ↧ |  |
| PF14\_0571:pep | 1.9e+02 | ↧ |  |
| PF14\_0815:pep | 2e+02 | ↧ |  |
| PFB0050c:pep | 2e+02 | ↧ |  |
| PF14\_0585:pep | 2e+02 | ↧ |  |
| PF11\_0459:pep | 2e+02 | ↧ |  |
| MAL13P1.505:pep | 2e+02 | ↧ |  |
| PF08\_0076:pep | 2e+02 | ↧ |  |
| PFE0230w:pep | 2e+02 | ↧ |  |
| PFF0255c:pep | 2e+02 | ↧ |  |
| PF10\_0194:pep | 2e+02 | ↧ |  |
| MAL13P1.395:pep | 2e+02 | ↧ |  |
| PFC0900w:pep | 2e+02 | ↧ |  |
| PFE0055c:pep | 2.1e+02 | ↧ |  |
| PFA0710c:pep | 2.1e+02 | ↧ |  |
| PFI0115c:pep | 2.1e+02 | ↧ |  |
| MAL7P1.172:pep | 2.1e+02 | ↧ |  |
| PFL2400w:pep | 2.1e+02 | ↧ |  |
| PF14\_0614:pep | 2.1e+02 | ↧ |  |
| PFD0660w:pep | 2.1e+02 | ↧ |  |
| PF13\_0205:pep | 2.2e+02 | ↧ |  |
| PFL0130c:pep | 2.2e+02 | ↧ |  |
| PFL1975c:pep | 2.2e+02 | ↧ |  |
| PF10\_0195:pep | 2.2e+02 | ↧ |  |
| PFA0115w:pep | 2.2e+02 | ↧ |  |
| PFF1135w:pep | 2.2e+02 | ↧ |  |
| PFA0430c:pep | 2.2e+02 | ↧ |  |
| PF14\_0627:pep | 2.2e+02 | ↧ |  |
| PF13\_0021:pep | 2.2e+02 | ↧ |  |
| MAL13P1.147:pep | 2.3e+02 | ↧ |  |
| PFC1005c:pep | 2.3e+02 | ↧ |  |
| PF11\_0087:pep | 2.3e+02 | ↧ |  |
| PFC0340w:pep | 2.3e+02 | ↧ |  |
| PF14\_0315:pep | 2.3e+02 | ↧ |  |
| PF10\_0304:pep | 2.4e+02 | ↧ |  |
| PF13\_0015:pep | 2.4e+02 | ↧ |  |
| PF11\_0224:pep | 2.4e+02 | ↧ |  |
| MAL7P1.59:pep | 2.4e+02 | ↧ |  |
| PF08\_0044:pep | 2.4e+02 | ↧ |  |
| PFF0573c:pep | 2.4e+02 | ↧ |  |
| PFC0582c.1 | 2.4e+02 | ↧ |  |
| MAL13P1.306.1 | 2.4e+02 | ↧ |  |
| PFL2395c:pep | 2.4e+02 | ↧ |  |
| PFF1110c:pep | 2.4e+02 | ↧ |  |
| PFL0190w:pep | 2.5e+02 | ↧ |  |
| PF14\_0694:pep | 2.5e+02 | ↧ |  |
| MAL7P1.139:pep | 2.5e+02 | ↧ |  |
| PF11\_0455a:pep | 2.5e+02 | ↧ |  |
| PF13\_0186:pep | 2.5e+02 | ↧ |  |
| MAL13P1.255:pep | 2.5e+02 | ↧ |  |

|  |  |
| --- | --- |
|  | Motif 1 |

Top

##### MAST version

4.10.0 (Release date: Wed May 21 10:35:36 2014 +1000)

##### Reference

Timothy L. Bailey and Michael Gribskov,
"Combining evidence using p-values: application to sequence homology searches",
*Bioinformatics*, **14**(1):48-54, 1998.

##### Command line summary

mast -oc Hiller2004.1.mast.out -ev 250 Hiller2004.1.out/meme.html /Users/t.bailey/Genomes\_local/Pf276v1.24/Plasmodium\_falciparum.ASM276v1.24.pep.all.fa  
Background letter frequencies (from non-redundant database):  

A: 0.073   C: 0.018   D: 0.052   E: 0.062   F: 0.040   G: 0.069   H: 0.022   I: 0.056   K: 0.058   L: 0.092   M: 0.023   N: 0.046   P: 0.051   Q: 0.041   R: 0.052   S: 0.074   T: 0.059   V: 0.064   W: 0.013   Y: 0.033

  
Result calculation took 0.184 seconds

show model parameters...

##### Model parameters

max\_correlation = 0.60
remove\_correlated = value: "n"
strand\_handling = value: "protein"
translate\_dna = value: "n"
max\_seq\_evalue = 2.5e+02
adj\_hit\_pvalue = value: "n"
max\_hit\_pvalue = 0.0001
max\_weak\_pvalue = 0.0001
host = IMB12-009806-LT
when = Fri Dec 19 18:09:56 2014

hide model parameters...

|  |  |
| --- | --- |
| Explanation of MAST Results | Top |

#### The MAST results consist of

- The **inputs** to MAST including:
  1. The **sequence databases** showing the sequence
     and residue counts. [View]
  2. The **motifs** showing the name, width, best scoring match
     and similarity to other motifs. [View]
  3. The **nominal order and spacing** diagram.
- The **search results** showing top scoring sequences with
  tiling of all of the motifs matches shown for each of the sequences. [View]
- The **program** details including:
  1. The **version** of MAST and the date it was released. [View]
  2. The **reference** to cite if you use MAST in your research. [View]
  3. The **command line summary** detailing the parameters with which you ran MAST. [View]
- This **explanation** of how to interpret MAST results.

#### Inputs

MAST received the following inputs.

##### Sequence Databases

This table summarises the sequence databases specified to MAST.

Database
:   The name of the database file.

Sequence Count
:   The number of sequences in the database.

Residue Count
:   The number of residues in the database.

##### Motifs

Summary of the motifs specified to MAST.

Name
:   The name of the motif. If the motif has been removed or removal is recommended to avoid highly similar motifs
    then it will be displayed in red text.

Width
:   The width of the motif. No gaps are allowed in motifs supplied to MAST as it only works for motifs of a fixed width.

Best possible match
:   The sequence that would achieve the best possible match score and its reverse complement for nucleotide motifs.

Similarity
:   MAST computes the pairwise correlations between each pair of motifs. The correlation between two motifs is the
    maximum sum of Pearson's correlation coefficients for aligned columns divided by the width of the shorter motif.
    The maximum is found by trying all alignments of the two motifs. Motifs with correlations below 0.60 have little
    effect on the accuracy of the combined scores. Pairs of motifs with higher correlations should be removed from
    the query. Correlations above the supplied threshold are shown in red text.

##### Nominal Order and Spacing

This diagram shows the normal spacing of the motifs specified to MAST.

#### Search Results

MAST provides the following motif search results.

##### Top Scoring Sequences

This table summarises the top scoring sequences with a Sequence *E*-value
better than the threshold (default 10). The sequences are sorted by the Sequence
*E*-value from most to least significant.

Sequence
:   The name of the sequence. This maybe be linked to search a sequence database for the sequence name.

*E*-value
:   The *E*-value of the sequence. For DNA only; if strands were scored seperately
    then there will be two *E*-values for the sequence seperated by a "/". The score for the provided sequence
    will be first and the score for the reverse-complement will be second.

↧
:   Click on this to show additional information about the sequence such as a
    description, combined p-value and the annotated sequence.

Block Diagram
:   The block diagram shows the best non-overlapping tiling of motif matches on the sequence.

    - The length of the line shows the length of a sequence relative to all the other sequences.
    - A block is shown where the positional *p*-value
      of a motif is less (more significant) than the significance threshold which is 0.0001 by default.
    - If a significant motif match (as specified above) overlaps other significant motif matches then
      it is only displayed as a block if its positional *p*-value
      is less (more significant) then the product of the positional
      *p*-values of the significant matches that it overlaps.
    - The position of a block shows where a motif has matched the sequence.
    - The width of a block shows the width of the motif relative to the length of the sequence.
    - The colour and border of a block identifies the matching motif as in the legend.
    - The height of a block gives an indication of the significance of the match as
      taller blocks are more significant. The height is calculated to be proportional
      to the negative logarithm of the positional *p*-value,
      truncated at the height for a *p*-value of 1e-10.
    - Hovering the mouse cursor over the block causes the display of the motif name
      and other details in the hovering text.
    - DNA only; blocks displayed above the line are a match on the given DNA, whereas blocks
      displayed below the line are matches to the reverse-complement of the given DNA.
    - DNA only; when strands are scored separately then blocks may overlap on opposing strands.

##### Additional Sequence Information

Clicking on the ↧ link expands a box below the sequence with additional information and adds two dragable buttons
below the block diagram.

Description
:   The description appearing after the identifier in the fasta file used to specify the sequence.

Combined *p*-value
:   The combined *p*-value of the sequence. DNA only; if strands were scored
    seperately then there will be two *p*-values for the sequence seperated by a "/". The score for the provided sequence
    will be first and the score for the reverse-complement will be second.

Annotated Sequence
:   The annotated sequence shows a portion of the sequence with the matching motif sequences displayed above.
    The displayed portion of the sequence can be modified by sliding the two buttons below the sequence block diagram
    so that the portion you want to see is between the two needles attached to the buttons. By default the two buttons
    move together but you can drag one individually by holding shift before you start the drag. If the strands were
    scored seperately then they can't be both displayed at once due to overlaps and so a radio button offers the choice
    of strand to display.

#### Scoring

MAST scores sequences using the following measures.

##### Position score calculation

The score for the match of a position in a sequence to a motif is computed by by summing the appropriate entry
from each column of the position-dependent scoring matrix that represents the motif. Sequences shorter than
one or more of the motifs are skipped.

##### Position *p*-value

The position p-value of a match is the probability of a single random subsequence of the length of the motif
scoring at least as well as the observed match.

##### Sequence *p*-value

The sequence p-value of a score is defined as the probability of a random sequence of the same length containing
some match with as good or better a score.

##### Combined *p*-value

The combined p-value of a sequence measures the strength of the match of the sequence to all the motifs and is calculated by

1. finding the score of the single best match of each motif to the sequence (best matches may overlap),
2. calculating the sequence p-value of each score,
3. forming the product of the p-values,
4. taking the p-value of the product.

##### Sequence *E*-value

The E-value of a sequence is the expected number of sequences in a random database of the same size that would match
the motifs as well as the sequence does and is equal to the combined p-value of the sequence times the number of
sequences in the database.


  
  
  
  
  
  
  
  
  
  
  
  
  
  
  
  
  
  
  
  
  
  
  
  
  
  
  
  
  
  
  
  
  
  
  
  
  
  
  
  
  
  
  
  
  
  
  
  
  
  
  
  
  
ACGTN
