# Supplementary figures and images for: The MEME Suite
Source: Nucleic Acids Res. 2015 May 7;43(Web Server issue):W39–49. doi: 10.1093/nar/gkv416 (PMC4489269; doi:10.1093/nar/gkv416)

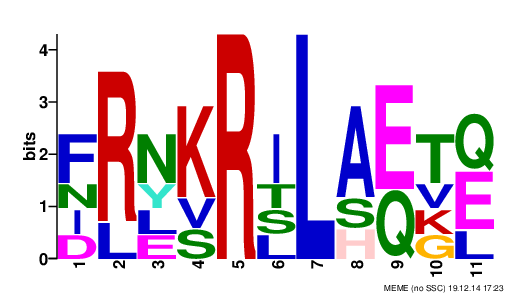

Supplement: SUPPLEMENTARY DATA [file supp_gkv416_nar-00283-web-b-2015-File002.zip › case1/Hiller2004.1.meme.out/logo1.png]

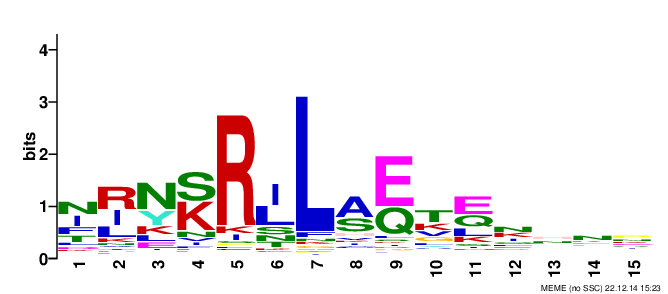

Supplement: SUPPLEMENTARY DATA [file supp_gkv416_nar-00283-web-b-2015-File002.zip › case1/Hiller2004.2.meme.out/logo1.png]

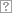

Supplement: SUPPLEMENTARY DATA [file supp_gkv416_nar-00283-web-b-2015-File003.zip › case2/cenp-t/help.gif]

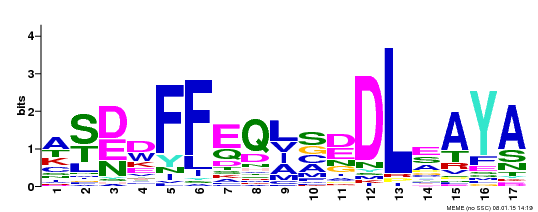

Supplement: SUPPLEMENTARY DATA [file supp_gkv416_nar-00283-web-b-2015-File003.zip › case2/cenp-t/logo1.png]

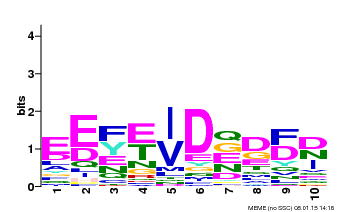

Supplement: SUPPLEMENTARY DATA [file supp_gkv416_nar-00283-web-b-2015-File003.zip › case2/cenp-t/logo10.png]

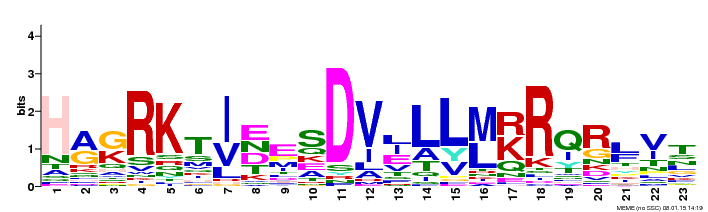

Supplement: SUPPLEMENTARY DATA [file supp_gkv416_nar-00283-web-b-2015-File003.zip › case2/cenp-t/logo2.png]

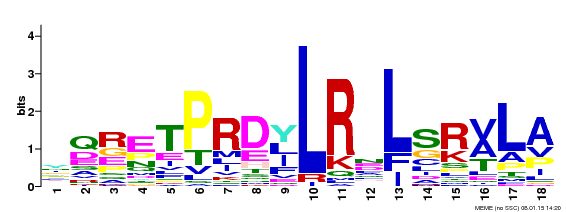

Supplement: SUPPLEMENTARY DATA [file supp_gkv416_nar-00283-web-b-2015-File003.zip › case2/cenp-t/logo3.png]

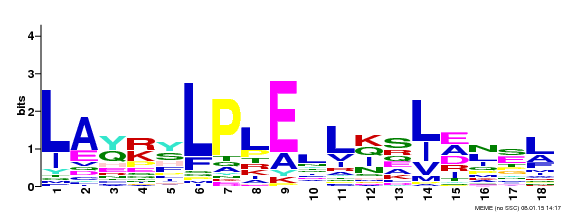

Supplement: SUPPLEMENTARY DATA [file supp_gkv416_nar-00283-web-b-2015-File003.zip › case2/cenp-t/logo4.png]

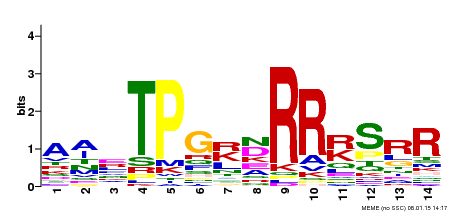

Supplement: SUPPLEMENTARY DATA [file supp_gkv416_nar-00283-web-b-2015-File003.zip › case2/cenp-t/logo5.png]

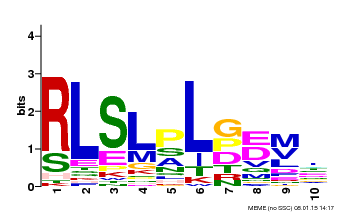

Supplement: SUPPLEMENTARY DATA [file supp_gkv416_nar-00283-web-b-2015-File003.zip › case2/cenp-t/logo6.png]

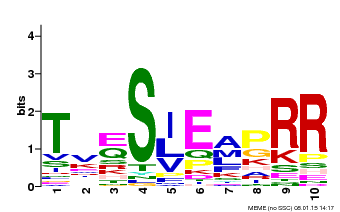

Supplement: SUPPLEMENTARY DATA [file supp_gkv416_nar-00283-web-b-2015-File003.zip › case2/cenp-t/logo7.png]

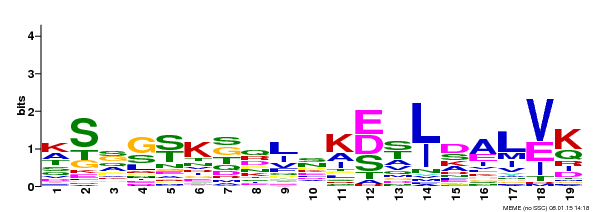

Supplement: SUPPLEMENTARY DATA [file supp_gkv416_nar-00283-web-b-2015-File003.zip › case2/cenp-t/logo8.png]

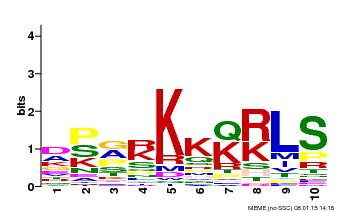

Supplement: SUPPLEMENTARY DATA [file supp_gkv416_nar-00283-web-b-2015-File003.zip › case2/cenp-t/logo9.png]

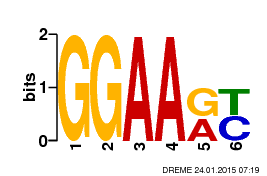

Supplement: SUPPLEMENTARY DATA [file supp_gkv416_nar-00283-web-b-2015-File005.zip › case4/meme-chip/dreme_out/m01nc_GGAARY.png]

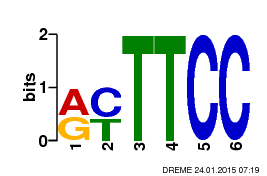

Supplement: SUPPLEMENTARY DATA [file supp_gkv416_nar-00283-web-b-2015-File005.zip › case4/meme-chip/dreme_out/m01rc_RYTTCC.png]

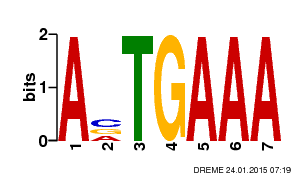

Supplement: SUPPLEMENTARY DATA [file supp_gkv416_nar-00283-web-b-2015-File005.zip › case4/meme-chip/dreme_out/m02nc_AVTGAAA.png]

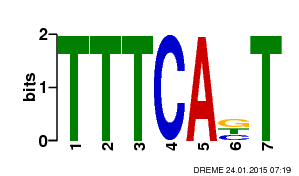

Supplement: SUPPLEMENTARY DATA [file supp_gkv416_nar-00283-web-b-2015-File005.zip › case4/meme-chip/dreme_out/m02rc_TTTCABT.png]

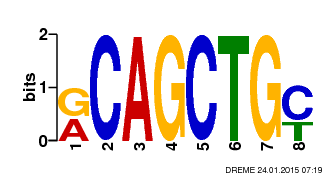

Supplement: SUPPLEMENTARY DATA [file supp_gkv416_nar-00283-web-b-2015-File005.zip › case4/meme-chip/dreme_out/m03nc_RCAGCTGY.png]

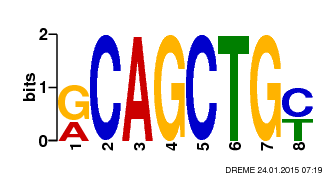

Supplement: SUPPLEMENTARY DATA [file supp_gkv416_nar-00283-web-b-2015-File005.zip › case4/meme-chip/dreme_out/m03rc_RCAGCTGY.png]

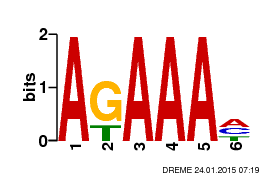

Supplement: SUPPLEMENTARY DATA [file supp_gkv416_nar-00283-web-b-2015-File005.zip › case4/meme-chip/dreme_out/m04nc_AKAAAH.png]

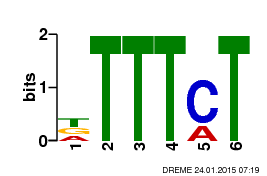

Supplement: SUPPLEMENTARY DATA [file supp_gkv416_nar-00283-web-b-2015-File005.zip › case4/meme-chip/dreme_out/m04rc_DTTTMT.png]

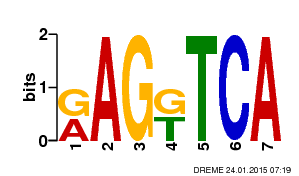

Supplement: SUPPLEMENTARY DATA [file supp_gkv416_nar-00283-web-b-2015-File005.zip › case4/meme-chip/dreme_out/m05nc_RAGKTCA.png]

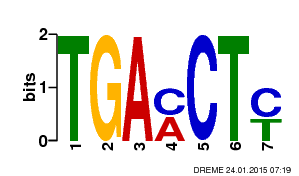

Supplement: SUPPLEMENTARY DATA [file supp_gkv416_nar-00283-web-b-2015-File005.zip › case4/meme-chip/dreme_out/m05rc_TGAMCTY.png]

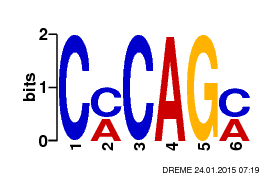

Supplement: SUPPLEMENTARY DATA [file supp_gkv416_nar-00283-web-b-2015-File005.zip › case4/meme-chip/dreme_out/m06nc_CMCAGM.png]

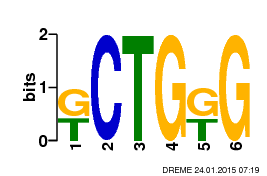

Supplement: SUPPLEMENTARY DATA [file supp_gkv416_nar-00283-web-b-2015-File005.zip › case4/meme-chip/dreme_out/m06rc_KCTGKG.png]

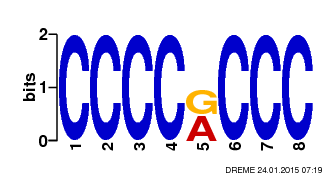

Supplement: SUPPLEMENTARY DATA [file supp_gkv416_nar-00283-web-b-2015-File005.zip › case4/meme-chip/dreme_out/m07nc_CCCCRCCC.png]

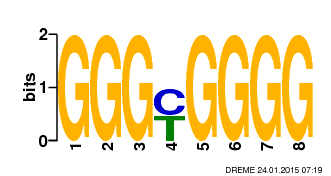

Supplement: SUPPLEMENTARY DATA [file supp_gkv416_nar-00283-web-b-2015-File005.zip › case4/meme-chip/dreme_out/m07rc_GGGYGGGG.png]

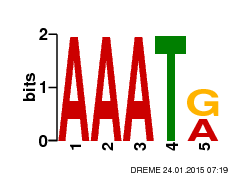

Supplement: SUPPLEMENTARY DATA [file supp_gkv416_nar-00283-web-b-2015-File005.zip › case4/meme-chip/dreme_out/m08nc_AAATR.png]

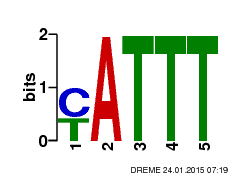

Supplement: SUPPLEMENTARY DATA [file supp_gkv416_nar-00283-web-b-2015-File005.zip › case4/meme-chip/dreme_out/m08rc_YATTT.png]

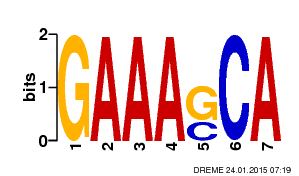

Supplement: SUPPLEMENTARY DATA [file supp_gkv416_nar-00283-web-b-2015-File005.zip › case4/meme-chip/dreme_out/m09nc_GAAASCA.png]

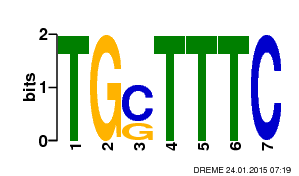

Supplement: SUPPLEMENTARY DATA [file supp_gkv416_nar-00283-web-b-2015-File005.zip › case4/meme-chip/dreme_out/m09rc_TGSTTTC.png]

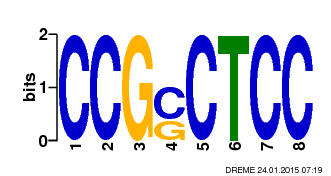

Supplement: SUPPLEMENTARY DATA [file supp_gkv416_nar-00283-web-b-2015-File005.zip › case4/meme-chip/dreme_out/m10nc_CCGSCTCC.png]

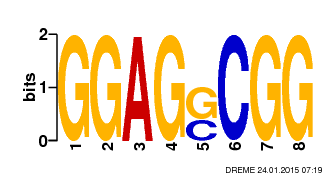

Supplement: SUPPLEMENTARY DATA [file supp_gkv416_nar-00283-web-b-2015-File005.zip › case4/meme-chip/dreme_out/m10rc_GGAGSCGG.png]

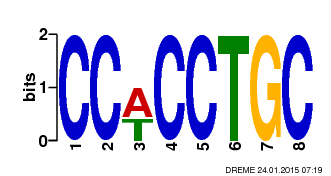

Supplement: SUPPLEMENTARY DATA [file supp_gkv416_nar-00283-web-b-2015-File005.zip › case4/meme-chip/dreme_out/m11nc_CCWCCTGC.png]

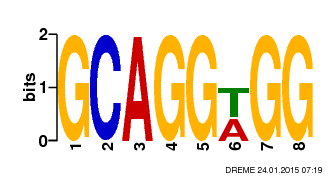

Supplement: SUPPLEMENTARY DATA [file supp_gkv416_nar-00283-web-b-2015-File005.zip › case4/meme-chip/dreme_out/m11rc_GCAGGWGG.png]

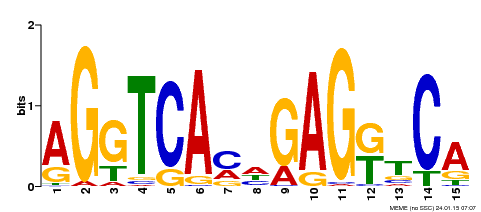

Supplement: SUPPLEMENTARY DATA [file supp_gkv416_nar-00283-web-b-2015-File005.zip › case4/meme-chip/meme_out/logo1.png]

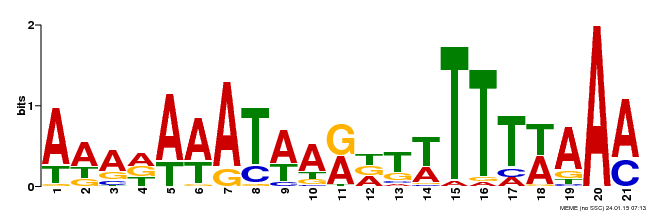

Supplement: SUPPLEMENTARY DATA [file supp_gkv416_nar-00283-web-b-2015-File005.zip › case4/meme-chip/meme_out/logo2.png]

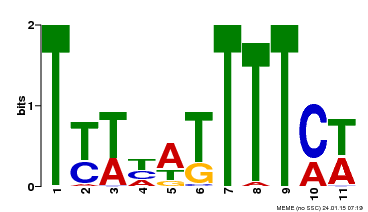

Supplement: SUPPLEMENTARY DATA [file supp_gkv416_nar-00283-web-b-2015-File005.zip › case4/meme-chip/meme_out/logo3.png]

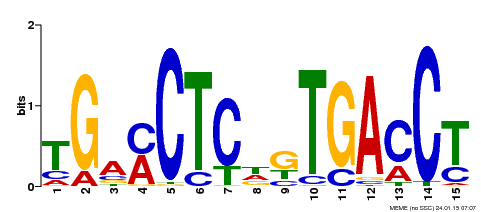

Supplement: SUPPLEMENTARY DATA [file supp_gkv416_nar-00283-web-b-2015-File005.zip › case4/meme-chip/meme_out/logo_rc1.png]

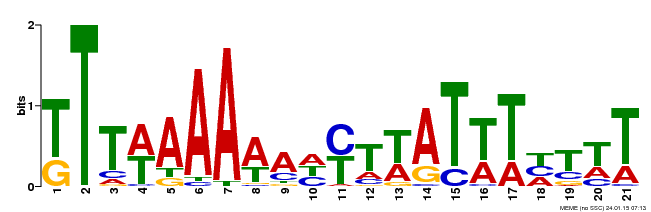

Supplement: SUPPLEMENTARY DATA [file supp_gkv416_nar-00283-web-b-2015-File005.zip › case4/meme-chip/meme_out/logo_rc2.png]

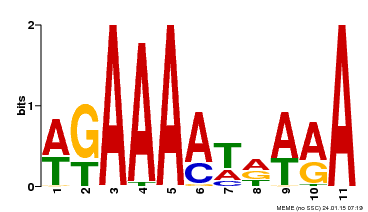

Supplement: SUPPLEMENTARY DATA [file supp_gkv416_nar-00283-web-b-2015-File005.zip › case4/meme-chip/meme_out/logo_rc3.png]
