# Supplementary material for: The MEME Suite
Source: Nucleic Acids Res. 2015 May 7;43(Web Server issue):W39–49. doi: 10.1093/nar/gkv416 (PMC4489269; doi:10.1093/nar/gkv416)
Supplement: SUPPLEMENTARY DATA [file supp_gkv416_nar-00283-web-b-2015-File004.zip › case3/mcast-web/mcast.html]

MCAST


# MCAST

## Motif Cluster Alignment and Search Tool

For further information on how to interpret these results or to get a
copy of the MEME software please access
http://meme.nbcr.net.

If you use MCAST in your research please cite the following paper:  
Timothy Bailey and William Stafford Noble,
"Searching for statistically significant regulatory modules",
*Bioinformatics (Proceedings of the European Conference on Computational Biology)*,
19(Suppl. 2):ii16-ii25, 2003.
[full text]

Inputs
  |  
Search Results
  |  
Program information
  |  
Documentation

|  |  |
| --- | --- |
| Inputs | Top |

#### Sequence Database

The sequence database
"cyp3a4-5.fa"
was supplied to MCAST.

| Sequence Count | Residue Count |
| --- | --- |
| 1 | 2021711 |

#### Motifs

The following motifs were supplied to MCAST from
"liver-motifs.meme"

|  |  |  |  |
| --- | --- | --- | --- |
| Motif | Width | Best possible match | Best possible RC match |
| MA0046.1 | 14 | GGTTAATAATTACC | GTTAATTATTAACC |
| HNF4A\_full | 16 | GGGTCCAAAGTCCAAT | ATTGGACTTTGGACCC |
| POU2F1\_DBD | 12 | AATATGCAAATT | AATTTGCATATT |
| MA0065.2 | 15 | GTAGGGCAAAGGTCA | TGACCTTTGCCCTAC |
| CEBPB\_full | 10 | ATTGCGCAAT | ATTGCGCAAT |
| hnf3beta | 6 | GTTGTT | AACAAC |

|  |  |
| --- | --- |
| Search Results | Top |

#### Top Scoring Matches

Each of the following
118 matches
has a *q*-value less than
1.
  
The motif matches shown have a position p-value less than
0.001.  
**Click on the arrow** (↧) next to the block diagram to view more information about a sequence.

|  |  |
| --- | --- |
|  | Motif MA0046.1 |

|  |  |
| --- | --- |
|  | Motif HNF4A\_full |

|  |  |
| --- | --- |
|  | Motif POU2F1\_DBD |

|  |  |
| --- | --- |
|  | Motif MA0065.2 |

|  |  |
| --- | --- |
|  | Motif CEBPB\_full |

|  |  |
| --- | --- |
|  | Motif hnf3beta |

|  | Sequence | Start | Stop | Score | *p*-value | *E*-value | *q*-value |  |  |
| --- | --- | --- | --- | --- | --- | --- | --- | --- | --- |
|  | chr7:99000000-100000000 | 457508 | 460609 | 50.63 | 0.00093 | 0.532 | 0.466 |
| ↧ | +  - | | | | | | | |
|  | chr7:99000000-100000000 | 483371 | 485875 | 44.3184 | 0.00245 | 1.4 | 0.466 |
| ↧ | +  - | | | | | | | |
|  | chr7:99000000-100000000 | 228846 | 231464 | 44.1347 | 0.00252 | 1.44 | 0.466 |
| ↧ | +  - | | | | | | | |
|  | chr7:99000000-100000000 | 882537 | 884356 | 40.7715 | 0.00422 | 2.42 | 0.55 |
| ↧ | +  - | | | | | | | |
|  | chr7:99000000-100000000 | 766952 | 768854 | 39.6858 | 0.00499 | 2.85 | 0.55 |
| ↧ | +  - | | | | | | | |
|  | chr7:99000000-100000000 | 655504 | 657270 | 38.5348 | 0.00595 | 3.41 | 0.55 |
| ↧ | +  - | | | | | | | |
|  | chr7:99000000-100000000 | 369153 | 370428 | 37.1271 | 0.00739 | 4.23 | 0.56 |
| ↧ | +  - | | | | | | | |
|  | chr7:99000000-100000000 | 436727 | 438261 | 35.8177 | 0.00904 | 5.17 | 0.56 |
| ↧ | +  - | | | | | | | |
|  | chr7:99000000-100000000 | 494623 | 495665 | 35.5799 | 0.00937 | 5.36 | 0.56 |
| ↧ | +  - | | | | | | | |
|  | chr7:99000000-100000000 | 453051 | 454231 | 34.8016 | 0.0106 | 6.04 | 0.56 |
| ↧ | +  - | | | | | | | |
|  | chr7:99000000-100000000 | 970135 | 971648 | 34.4617 | 0.0111 | 6.36 | 0.56 |
| ↧ | +  - | | | | | | | |
|  | chr7:99000000-100000000 | 442618 | 444051 | 33.7114 | 0.0125 | 7.14 | 0.56 |
| ↧ | +  - | | | | | | | |
|  | chr7:99000000-100000000 | 613132 | 615252 | 33.3837 | 0.0131 | 7.51 | 0.56 |
| ↧ | +  - | | | | | | | |
|  | chr7:99000000-100000000 | 955816 | 956294 | 31.292 | 0.0181 | 10.4 | 0.587 |
| ↧ | +  - | | | | | | | |
|  | chr7:99000000-100000000 | 390598 | 391276 | 31.1583 | 0.0185 | 10.6 | 0.587 |
| ↧ | +  - | | | | | | | |
|  | chr7:99000000-100000000 | 426210 | 428714 | 30.4264 | 0.0207 | 11.8 | 0.587 |
| ↧ | +  - | | | | | | | |
|  | chr7:99000000-100000000 | 937142 | 939547 | 31.7732 | 0.0213 | 12.2 | 0.587 |
| ↧ | +  - | | | | | | | |
|  | chr7:99000000-100000000 | 507962 | 510472 | 30.2197 | 0.0213 | 12.2 | 0.587 |
| ↧ | +  - | | | | | | | |
|  | chr7:99000000-100000000 | 989858 | 992535 | 32.9835 | 0.0215 | 12.3 | 0.587 |
| ↧ | +  - | | | | | | | |
|  | chr7:99000000-100000000 | 639260 | 639800 | 29.8824 | 0.0225 | 12.9 | 0.587 |
| ↧ | +  - | | | | | | | |
|  | chr7:99000000-100000000 | 316747 | 318531 | 28.8796 | 0.0262 | 15 | 0.587 |
| ↧ | +  - | | | | | | | |
|  | chr7:99000000-100000000 | 572396 | 573865 | 28.8399 | 0.0264 | 15.1 | 0.587 |
| ↧ | +  - | | | | | | | |
|  | chr7:99000000-100000000 | 987447 | 988641 | 28.7228 | 0.0275 | 15.7 | 0.587 |
| ↧ | +  - | | | | | | | |
|  | chr7:99000000-100000000 | 364688 | 365830 | 28.231 | 0.029 | 16.6 | 0.587 |
| ↧ | +  - | | | | | | | |
|  | chr7:99000000-100000000 | 867512 | 868606 | 28.0766 | 0.0297 | 17 | 0.587 |
| ↧ | +  - | | | | | | | |
|  | chr7:99000000-100000000 | 710342 | 711475 | 28.0612 | 0.0297 | 17 | 0.587 |
| ↧ | +  - | | | | | | | |
|  | chr7:99000000-100000000 | 691968 | 692786 | 27.914 | 0.0304 | 17.4 | 0.587 |
| ↧ | +  - | | | | | | | |
|  | chr7:99000000-100000000 | 879823 | 880487 | 27.8835 | 0.0305 | 17.5 | 0.587 |
| ↧ | +  - | | | | | | | |
|  | chr7:99000000-100000000 | 633265 | 634894 | 27.85 | 0.0307 | 17.6 | 0.587 |
| ↧ | +  - | | | | | | | |
|  | chr7:99000000-100000000 | 276609 | 278305 | 27.3243 | 0.0333 | 19 | 0.613 |
| ↧ | +  - | | | | | | | |
|  | chr7:99000000-100000000 | 117523 | 119526 | 26.9589 | 0.0352 | 20.1 | 0.613 |
| ↧ | +  - | | | | | | | |
|  | chr7:99000000-100000000 | 373260 | 376086 | 26.7084 | 0.0366 | 20.9 | 0.613 |
| ↧ | +  - | | | | | | | |
|  | chr7:99000000-100000000 | 580643 | 581537 | 26.6102 | 0.0371 | 21.2 | 0.613 |
| ↧ | +  - | | | | | | | |
|  | chr7:99000000-100000000 | 268525 | 269693 | 26.494 | 0.0378 | 21.6 | 0.613 |
| ↧ | +  - | | | | | | | |
|  | chr7:99000000-100000000 | 296160 | 298357 | 26.3428 | 0.0387 | 22.1 | 0.613 |
| ↧ | +  - | | | | | | | |
|  | chr7:99000000-100000000 | 337069 | 338708 | 25.7581 | 0.0423 | 24.2 | 0.629 |
| ↧ | +  - | | | | | | | |
|  | chr7:99000000-100000000 | 595371 | 596875 | 25.5537 | 0.0437 | 25 | 0.629 |
| ↧ | +  - | | | | | | | |
|  | chr7:99000000-100000000 | 157009 | 158174 | 25.2854 | 0.0455 | 26 | 0.629 |
| ↧ | +  - | | | | | | | |
|  | chr7:99000000-100000000 | 396849 | 398044 | 24.9917 | 0.0476 | 27.2 | 0.629 |
| ↧ | +  - | | | | | | | |
|  | chr7:99000000-100000000 | 810548 | 812598 | 25.5408 | 0.0479 | 27.4 | 0.629 |
| ↧ | +  - | | | | | | | |
|  | chr7:99000000-100000000 | 708387 | 709639 | 24.7508 | 0.0494 | 28.3 | 0.629 |
| ↧ | +  - | | | | | | | |
|  | chr7:99000000-100000000 | 266580 | 267658 | 24.7336 | 0.0495 | 28.3 | 0.629 |
| ↧ | +  - | | | | | | | |
|  | chr7:99000000-100000000 | 584216 | 584855 | 24.6666 | 0.0501 | 28.6 | 0.629 |
| ↧ | +  - | | | | | | | |
|  | chr7:99000000-100000000 | 168342 | 169175 | 24.6095 | 0.0505 | 28.9 | 0.629 |
| ↧ | +  - | | | | | | | |
|  | chr7:99000000-100000000 | 568861 | 570974 | 24.4452 | 0.0518 | 29.6 | 0.629 |
| ↧ | +  - | | | | | | | |
|  | chr7:99000000-100000000 | 870004 | 872088 | 24.2467 | 0.0534 | 30.5 | 0.629 |
| ↧ | +  - | | | | | | | |
|  | chr7:99000000-100000000 | 139054 | 139937 | 24.1406 | 0.0543 | 31 | 0.629 |
| ↧ | +  - | | | | | | | |
|  | chr7:99000000-100000000 | 586423 | 587404 | 23.9954 | 0.0555 | 31.7 | 0.629 |
| ↧ | +  - | | | | | | | |
|  | chr7:99000000-100000000 | 755098 | 755660 | 23.841 | 0.0568 | 32.5 | 0.629 |
| ↧ | +  - | | | | | | | |
|  | chr7:99000000-100000000 | 772827 | 773638 | 23.8063 | 0.0571 | 32.7 | 0.629 |
| ↧ | +  - | | | | | | | |
|  | chr7:99000000-100000000 | 995682 | 996632 | 23.7199 | 0.0579 | 33.1 | 0.629 |
| ↧ | +  - | | | | | | | |
|  | chr7:99000000-100000000 | 349154 | 349935 | 23.1034 | 0.0636 | 36.4 | 0.678 |
| ↧ | +  - | | | | | | | |
|  | chr7:99000000-100000000 | 236367 | 237271 | 22.5735 | 0.069 | 39.5 | 0.707 |
| ↧ | +  - | | | | | | | |
|  | chr7:99000000-100000000 | 311587 | 313121 | 22.4283 | 0.0706 | 40.4 | 0.707 |
| ↧ | +  - | | | | | | | |
|  | chr7:99000000-100000000 | 845866 | 846095 | 22.2547 | 0.0725 | 41.5 | 0.707 |
| ↧ | +  - | | | | | | | |
|  | chr7:99000000-100000000 | 42262 | 42722 | 22.0659 | 0.0746 | 42.7 | 0.707 |
| ↧ | +  - | | | | | | | |
|  | chr7:99000000-100000000 | 335195 | 336280 | 22.028 | 0.075 | 42.9 | 0.707 |
| ↧ | +  - | | | | | | | |
|  | chr7:99000000-100000000 | 135625 | 136283 | 21.9796 | 0.0756 | 43.2 | 0.707 |
| ↧ | +  - | | | | | | | |
|  | chr7:99000000-100000000 | 751888 | 752671 | 21.9563 | 0.0759 | 43.4 | 0.707 |
| ↧ | +  - | | | | | | | |
|  | chr7:99000000-100000000 | 166771 | 167859 | 21.9045 | 0.0765 | 43.7 | 0.707 |
| ↧ | +  - | | | | | | | |
|  | chr7:99000000-100000000 | 448845 | 449452 | 21.793 | 0.0778 | 44.5 | 0.707 |
| ↧ | +  - | | | | | | | |
|  | chr7:99000000-100000000 | 763740 | 764976 | 21.5686 | 0.0805 | 46.1 | 0.716 |
| ↧ | +  - | | | | | | | |
|  | chr7:99000000-100000000 | 24812 | 25686 | 21.5023 | 0.0814 | 46.5 | 0.716 |
| ↧ | +  - | | | | | | | |
|  | chr7:99000000-100000000 | 919328 | 920392 | 23.6601 | 0.0843 | 48.2 | 0.73 |
| ↧ | +  - | | | | | | | |
|  | chr7:99000000-100000000 | 384422 | 385405 | 21.0204 | 0.0876 | 50.1 | 0.747 |
| ↧ | +  - | | | | | | | |
|  | chr7:99000000-100000000 | 203646 | 204573 | 20.3329 | 0.0974 | 55.7 | 0.787 |
| ↧ | +  - | | | | | | | |
|  | chr7:99000000-100000000 | 832706 | 833218 | 20.3217 | 0.0979 | 56 | 0.787 |
| ↧ | +  - | | | | | | | |
|  | chr7:99000000-100000000 | 306903 | 308120 | 20.2747 | 0.0982 | 56.2 | 0.787 |
| ↧ | +  - | | | | | | | |
|  | chr7:99000000-100000000 | 550943 | 552540 | 20.0895 | 0.101 | 57.8 | 0.787 |
| ↧ | +  - | | | | | | | |
|  | chr7:99000000-100000000 | 423493 | 424263 | 20.0462 | 0.102 | 58.2 | 0.787 |
| ↧ | +  - | | | | | | | |
|  | chr7:99000000-100000000 | 478058 | 478720 | 20.0169 | 0.102 | 58.5 | 0.787 |
| ↧ | +  - | | | | | | | |
|  | chr7:99000000-100000000 | 915552 | 916097 | 20.2912 | 0.102 | 58.5 | 0.787 |
| ↧ | +  - | | | | | | | |
|  | chr7:99000000-100000000 | 292495 | 292878 | 19.733 | 0.107 | 61.1 | 0.81 |
| ↧ | +  - | | | | | | | |
|  | chr7:99000000-100000000 | 176824 | 177493 | 19.63 | 0.108 | 62 | 0.812 |
| ↧ | +  - | | | | | | | |
|  | chr7:99000000-100000000 | 541219 | 542233 | 19.4964 | 0.111 | 63.3 | 0.813 |
| ↧ | +  - | | | | | | | |
|  | chr7:99000000-100000000 | 467793 | 467830 | 19.3967 | 0.112 | 64.3 | 0.813 |
| ↧ | +  - | | | | | | | |
|  | chr7:99000000-100000000 | 662257 | 663010 | 19.3183 | 0.114 | 65.1 | 0.813 |
| ↧ | +  - | | | | | | | |
|  | chr7:99000000-100000000 | 299172 | 299568 | 19.2166 | 0.116 | 66.1 | 0.813 |
| ↧ | +  - | | | | | | | |
|  | chr7:99000000-100000000 | 66031 | 66673 | 18.9312 | 0.121 | 69.1 | 0.813 |
| ↧ | +  - | | | | | | | |
|  | chr7:99000000-100000000 | 6388 | 7362 | 18.8279 | 0.123 | 70.2 | 0.813 |
| ↧ | +  - | | | | | | | |
|  | chr7:99000000-100000000 | 497988 | 499818 | 18.7162 | 0.125 | 71.4 | 0.813 |
| ↧ | +  - | | | | | | | |
|  | chr7:99000000-100000000 | 744120 | 745372 | 20.0165 | 0.125 | 71.5 | 0.813 |
| ↧ | +  - | | | | | | | |
|  | chr7:99000000-100000000 | 13674 | 14563 | 18.5909 | 0.127 | 72.8 | 0.813 |
| ↧ | +  - | | | | | | | |
|  | chr7:99000000-100000000 | 224094 | 225649 | 18.587 | 0.127 | 72.8 | 0.813 |
| ↧ | +  - | | | | | | | |
|  | chr7:99000000-100000000 | 954600 | 955116 | 18.5505 | 0.128 | 73.2 | 0.813 |
| ↧ | +  - | | | | | | | |
|  | chr7:99000000-100000000 | 322171 | 322566 | 18.5131 | 0.129 | 73.6 | 0.813 |
| ↧ | +  - | | | | | | | |
|  | chr7:99000000-100000000 | 175034 | 176265 | 18.4895 | 0.129 | 73.9 | 0.813 |
| ↧ | +  - | | | | | | | |
|  | chr7:99000000-100000000 | 912658 | 913641 | 18.2365 | 0.134 | 76.8 | 0.813 |
| ↧ | +  - | | | | | | | |
|  | chr7:99000000-100000000 | 690925 | 691693 | 18.2014 | 0.135 | 77.2 | 0.813 |
| ↧ | +  - | | | | | | | |
|  | chr7:99000000-100000000 | 450121 | 451218 | 18.0281 | 0.139 | 79.3 | 0.813 |
| ↧ | +  - | | | | | | | |
|  | chr7:99000000-100000000 | 3040 | 4941 | 17.9873 | 0.14 | 79.8 | 0.813 |
| ↧ | +  - | | | | | | | |
|  | chr7:99000000-100000000 | 604078 | 605909 | 17.9193 | 0.141 | 80.7 | 0.813 |
| ↧ | +  - | | | | | | | |
|  | chr7:99000000-100000000 | 578172 | 579117 | 17.7935 | 0.144 | 82.2 | 0.813 |
| ↧ | +  - | | | | | | | |
|  | chr7:99000000-100000000 | 601407 | 601783 | 17.7376 | 0.145 | 82.9 | 0.813 |
| ↧ | +  - | | | | | | | |
|  | chr7:99000000-100000000 | 1630 | 2112 | 17.6818 | 0.146 | 83.7 | 0.813 |
| ↧ | +  - | | | | | | | |
|  | chr7:99000000-100000000 | 488319 | 488859 | 17.6251 | 0.148 | 84.4 | 0.813 |
| ↧ | +  - | | | | | | | |
|  | chr7:99000000-100000000 | 786835 | 787283 | 18.6342 | 0.148 | 84.4 | 0.813 |
| ↧ | +  - | | | | | | | |
|  | chr7:99000000-100000000 | 476989 | 477516 | 17.5945 | 0.148 | 84.8 | 0.813 |
| ↧ | +  - | | | | | | | |
|  | chr7:99000000-100000000 | 466757 | 467054 | 17.5177 | 0.15 | 85.8 | 0.813 |
| ↧ | +  - | | | | | | | |
|  | chr7:99000000-100000000 | 962029 | 963269 | 18.2723 | 0.154 | 88.2 | 0.813 |
| ↧ | +  - | | | | | | | |
|  | chr7:99000000-100000000 | 594101 | 594877 | 17.2737 | 0.156 | 89.1 | 0.813 |
| ↧ | +  - | | | | | | | |
|  | chr7:99000000-100000000 | 173113 | 173450 | 17.2508 | 0.156 | 89.4 | 0.813 |
| ↧ | +  - | | | | | | | |
|  | chr7:99000000-100000000 | 667779 | 668526 | 17.2506 | 0.156 | 89.4 | 0.813 |
| ↧ | +  - | | | | | | | |
|  | chr7:99000000-100000000 | 892068 | 892148 | 17.5041 | 0.157 | 89.8 | 0.813 |
| ↧ | +  - | | | | | | | |
|  | chr7:99000000-100000000 | 575471 | 575781 | 17.1988 | 0.158 | 90.1 | 0.813 |
| ↧ | +  - | | | | | | | |
|  | chr7:99000000-100000000 | 800592 | 801687 | 17.9651 | 0.159 | 90.7 | 0.813 |
| ↧ | +  - | | | | | | | |
|  | chr7:99000000-100000000 | 232710 | 233301 | 17.121 | 0.159 | 91.2 | 0.813 |
| ↧ | +  - | | | | | | | |
|  | chr7:99000000-100000000 | 946500 | 947059 | 17.7849 | 0.16 | 91.8 | 0.813 |
| ↧ | +  - | | | | | | | |
|  | chr7:99000000-100000000 | 47635 | 48944 | 17.0402 | 0.161 | 92.3 | 0.813 |
| ↧ | +  - | | | | | | | |
|  | chr7:99000000-100000000 | 676859 | 677074 | 16.9905 | 0.163 | 93 | 0.813 |
| ↧ | +  - | | | | | | | |
|  | chr7:99000000-100000000 | 252122 | 254496 | 16.9845 | 0.163 | 93.1 | 0.813 |
| ↧ | +  - | | | | | | | |
|  | chr7:99000000-100000000 | 522302 | 523413 | 16.9064 | 0.165 | 94.2 | 0.815 |
| ↧ | +  - | | | | | | | |
|  | chr7:99000000-100000000 | 629706 | 630115 | 16.799 | 0.167 | 95.8 | 0.818 |
| ↧ | +  - | | | | | | | |
|  | chr7:99000000-100000000 | 705220 | 705817 | 16.6205 | 0.172 | 98.5 | 0.818 |
| ↧ | +  - | | | | | | | |
|  | chr7:99000000-100000000 | 417739 | 418573 | 16.5993 | 0.173 | 98.8 | 0.818 |
| ↧ | +  - | | | | | | | |
|  | chr7:99000000-100000000 | 746476 | 747195 | 16.5864 | 0.173 | 99 | 0.818 |
| ↧ | +  - | | | | | | | |
|  | chr7:99000000-100000000 | 178198 | 178874 | 16.5742 | 0.173 | 99.2 | 0.818 |
| ↧ | +  - | | | | | | | |
|  | chr7:99000000-100000000 | 567041 | 568569 | 16.5411 | 0.174 | 99.7 | 0.818 |
| ↧ | +  - | | | | | | | |

Top

##### MCAST version

4.10.0 (Release date: Wed May 21 10:35:36 2014 +1000)

##### Command line summary

/ebi/sw/MEME/VM-cluster410/meme-versions/4.10.0/bin/mcast --oc . --verbosity 1 --bgweight 4.0 --output-ethresh 100.0 --max-gap 250 --motif-pthresh 0.001 liver-motifs.meme cyp3a4-5.fa  
Background letter frequencies (from non-redundant database):  

A: 0.282   C: 0.222   G: 0.229   T: 0.267

show model parameters...hide model parameters...

|  |  |
| --- | --- |
| Explanation of MCAST Results | Top |

#### The MCAST results consist of

- The **inputs** to MCAST including:
  1. The **sequence database** showing the sequence
     and residue counts. [View]
  2. The **motifs** showing the name, width,
     and best scoring match [View]
- The **search results** showing top scoring sequences with
  tiling of all of the motifs matches shown for each of the sequences. [View]
- The **program** details including:
  1. The **version** of MCAST and the date it was released. [View]
  2. The **command line summary** detailing the parameters with which you ran MCAST. [View]
- This **explanation** of how to interpret MCAST results.

#### Inputs

MCAST received the following inputs.

##### Sequence Databases

This table summarises the sequence databases specified to MCAST.

Database
:   The name of the database file.

Sequence Count
:   The number of sequences in the database.

Residue Count
:   The number of residues in the database.

##### Motifs

Summary of the motifs specified to MCAST.

Name
:   The name of the motif. If the motif has been removed or removal is recommended to avoid highly similar motifs
    then it will be displayed in red text.

Width
:   The width of the motif. No gaps are allowed in motifs supplied to MCAST as it only works for motifs of a fixed width.

Best possible match
:   The sequence that would achieve the best possible match score and its reverse complement for nucleotide motifs.

#### Search Results

MCAST provides the following motif search results.

##### Top Scoring Sequences

This table summarises the top scoring sequences.
The sequences are sorted by the Sequence
*q*-value from most to least significant.

Sequence
:   The name of the sequence. This maybe be linked to search a sequence database for the sequence name.

Start
:   The position of the start of the match in 1-based coordinates, starting at the
    first position in the sequence.

Stop
:   The position of the end of the match in 1-based coordinates, starting at the
    first position in the sequence.

Score
:   The MCAST score for the match.

*p*-value
:   The *p*-value of the match score.

*E*-value
:   The *E*-value of the match score.

*q*-value
:   The *q*-value of the match score.

↧
:   Click on this to show additional information about the sequence such as a
    description, combined p-value and the annotated sequence.

Block Diagram
:   The block diagram shows the best non-overlapping tiling of motif matches on the sequence.

    - The length of the line shows the length of a sequence relative to all the other sequences.
    - A block is shown where the positional *p*-value
      of a motif is less (more significant) than the significance threshold which is 0.0001 by default.
    - If a significant motif match (as specified above) overlaps other significant motif matches then
      it is only displayed as a block if its positional *p*-value
      is less (more significant) then the product of the positional
      *p*-values of the significant matches that it overlaps.
    - The position of a block shows where a motif has matched the sequence.
    - The width of a block shows the width of the motif relative to the length of the sequence.
    - The colour and border of a block identifies the matching motif as in the legend.
    - The height of a block gives an indication of the significance of the match as
      taller blocks are more significant. The height is calculated to be proportional
      to the negative logarithm of the positional *p*-value,
      truncated at the height for a *p*-value of 1e-10.
    - Hovering the mouse cursor over the block causes the display of the motif name
      and other details in the hovering text.
    - DNA only; blocks displayed above the line are a match on the given DNA, whereas blocks
      displayed below the line are matches to the reverse-complement of the given DNA.
    - DNA only; when strands are scored separately then blocks may overlap on opposing strands.


  
  
  
  
  
  
  
  
  
  
  
  
  
  
  
  
  
  
  
  
  
  
  
  
  
  
  
  
  
  
  
  
  
  
  
  
  
  
  
  
  
  
  
  
  
  
  
  
  
  
  
  
  
ACGTN
