# Supplementary material for: The MEME Suite
Source: Nucleic Acids Res. 2015 May 7;43(Web Server issue):W39–49. doi: 10.1093/nar/gkv416 (PMC4489269; doi:10.1093/nar/gkv416)
Supplement: SUPPLEMENTARY DATA [file supp_gkv416_nar-00283-web-b-2015-File005.zip › case4/meme-chip/fimo_out_1/fimo.html]

FIMO Results


---

|  |  |  |
| --- | --- | --- |
| **Database and Motifs** | **High-scoring Motif Occurrences** | **Debugging Information** |

  
  

---

**FIMO - Motif search tool**


---

FIMO version 4.10.0,
(Release date: Wed May 21 10:35:36 2014 +1000)

For further information on how to interpret these results
or to get a copy of the FIMO software please access
http://meme.nbcr.net

If you use FIMO in your research, please cite the following paper:  
Charles E. Grant, Timothy L. Bailey, and William Stafford Noble,
"FIMO: Scanning for occurrences of a given motif",
*Bioinformatics*, **27**(7):1017-1018, 2011.
[full text]

---

**DATABASE AND MOTIFS**


---

DATABASE
./Supplementary\_Table\_1.500bp.fa  
Database contains
2776
sequences,
1388000
residues

MOTIFS
meme\_out/meme.xml
(nucleotide)

| MOTIF | WIDTH | BEST POSSIBLE MATCH |
| --- | --- | --- |
| 1 | 15 | AGGTCACAGAGGTCA |
| 2 | 21 | AAAAAAATAAGTTTTTTTAAA |
| 3 | 11 | TTTTATTTTCT |

Random model letter frequencies
(from ./background):
  
A 0.241 C 0.259 G 0.259 T 0.241

---

**SECTION I: HIGH-SCORING MOTIF OCCURRENCES**


---

- There were
  832
  motif occurrences with a
  p-value less than
  0.0001.
- The p-value of a motif occurrence is defined as the
  probability of a random sequence of the same length as the motif
  matching that position of the sequence with as good or better a score.
- The score for the match of a position in a sequence to a motif
  is computed by summing the appropriate entries from each column of
  the position-dependent scoring matrix that represents the motif.
- The q-value of a motif occurrence is defined as the
  false discovery rate if the occurrence is accepted as significant.
- The table is sorted by increasing p-value.

| Motif | Sequence Name | Strand | Start | End | p-value | q-value | Matched Sequence |
| --- | --- | --- | --- | --- | --- | --- | --- |
| 1 | chr1 | + | 41973757 | 41973771 | 9.57e-10 | 0.00205 | `AGGTCACAGAGGTCA` |
| 1 | chr15 | + | 70554390 | 70554404 | 1.28e-08 | 0.01 | `AGGTCAACGAGGTCA` |
| 1 | chr4 | − | 144654241 | 144654255 | 2.49e-08 | 0.01 | `GGGTCACTGAGGTCA` |
| 1 | chr1 | + | 120065799 | 120065813 | 2.87e-08 | 0.01 | `AGGTCAGTGAGTTCA` |
| 1 | chr10 | + | 73603903 | 73603917 | 2.96e-08 | 0.01 | `AGTTCACCGAGTTCA` |
| 1 | chr11 | − | 85835751 | 85835765 | 3.24e-08 | 0.01 | `GGGTCACCGAGGTCA` |
| 1 | chr8 | + | 105750564 | 105750578 | 3.44e-08 | 0.01 | `AGGTCACTGAGGGCA` |
| 1 | chr6 | − | 47513526 | 47513540 | 4.23e-08 | 0.01 | `AGGTGACAGAGGTCA` |
| 1 | chr19 | + | 56072018 | 56072032 | 4.32e-08 | 0.01 | `AGGTGACAGAGTTCA` |
| 1 | chr3 | − | 113554016 | 113554030 | 4.69e-08 | 0.01 | `AGGTCACCAAGTTCA` |
| 1 | chr18 | − | 19994275 | 19994289 | 6.2e-08 | 0.0103 | `AGGTGACTGAGTTCA` |
| 1 | chrX | + | 46382470 | 46382484 | 7.3e-08 | 0.0103 | `AGGTCACAGGGGTCA` |
| 1 | chr21 | + | 15516392 | 15516406 | 7.3e-08 | 0.0103 | `AGGTCACAGGGGTCA` |
| 1 | chr22 | + | 38038035 | 38038049 | 7.3e-08 | 0.0103 | `AGGTCACAGGGGTCA` |
| 1 | chr16 | − | 29973977 | 29973991 | 7.59e-08 | 0.0103 | `AGGTCACAGGGTTCA` |
| 1 | chr1 | − | 39054533 | 39054547 | 7.78e-08 | 0.0103 | `AGGTGACCGAGTTCA` |
| 1 | chr10 | − | 82002464 | 82002478 | 8.16e-08 | 0.0103 | `GGGTCAATGAGGTCA` |
| 1 | chr4 | − | 7529606 | 7529620 | 9.48e-08 | 0.0107 | `AGGTCAATAAGTTCA` |
| 1 | chr10 | + | 24715963 | 24715977 | 9.48e-08 | 0.0107 | `AGGTCAATAAGTTCA` |
| 1 | chr12 | + | 26343051 | 26343065 | 1.09e-07 | 0.0112 | `AGGTCACTGGGTTCA` |
| 1 | chr1 | + | 76218527 | 76218541 | 1.1e-07 | 0.0112 | `GGGTCAGAGAGTTCA` |
| 1 | chr20 | + | 23263230 | 23263244 | 1.31e-07 | 0.0125 | `AGGTCAGAAAGTTCA` |
| 1 | chr3 | + | 53780095 | 53780109 | 1.34e-07 | 0.0125 | `GGGTCACAGAGTTCG` |
| 1 | chr18 | + | 667402 | 667416 | 1.63e-07 | 0.0142 | `AGTTCACAGAGGCCA` |
| 1 | chr8 | + | 67507527 | 67507541 | 1.75e-07 | 0.0142 | `AGGTCACAGAGTGCG` |
| 1 | chr11 | − | 86825988 | 86826002 | 1.78e-07 | 0.0142 | `AGTTGACAGAGTTCA` |
| 1 | chr19 | − | 2734436 | 2734450 | 1.8e-07 | 0.0142 | `AGGTCGCAGAGGTCA` |
| 1 | chr11 | − | 128216998 | 128217012 | 1.97e-07 | 0.0151 | `AGGTCAGTGAGGGCA` |
| 1 | chr20 | + | 23262834 | 23262848 | 2.09e-07 | 0.0151 | `AGGTCAGAGAGTCCA` |
| 1 | chr6 | + | 27965911 | 27965925 | 2.13e-07 | 0.0151 | `GGGTCACAGAGGACA` |
| 1 | chr15 | − | 19249000 | 19249014 | 2.18e-07 | 0.0151 | `GGGTCACCAAGTTCA` |
| 1 | chr3 | + | 181479352 | 181479366 | 2.4e-07 | 0.0156 | `AGTTCACTGAGGCCA` |
| 1 | chr17 | + | 9300475 | 9300489 | 2.4e-07 | 0.0156 | `AGTTCACTGAGGCCA` |
| 1 | chr15 | + | 49308123 | 49308137 | 2.59e-07 | 0.0163 | `GGGTCACAGAGTTCT` |
| 1 | chr1 | − | 228391071 | 228391085 | 2.84e-07 | 0.0172 | `GGGTCACAGAGTTTA` |
| 1 | chr12 | + | 103617457 | 103617471 | 2.94e-07 | 0.0172 | `GGGTCACTGAGGACA` |
| 1 | chr19 | + | 4218775 | 4218789 | 3.06e-07 | 0.0172 | `AGGTCACAGAGTTCC` |
| 1 | chr20 | + | 23263270 | 23263284 | 3.06e-07 | 0.0172 | `AGGTCAGAGAGTTTA` |
| 1 | chr17 | − | 1457475 | 1457489 | 3.21e-07 | 0.0176 | `AGGTCAGTGAGGACA` |
| 1 | chr10 | + | 80514230 | 80514244 | 3.33e-07 | 0.0178 | `GGGTCAAAGAGGGCA` |
| 1 | chr15 | + | 61757295 | 61757309 | 3.52e-07 | 0.018 | `AGGTCAGCGAGGCCA` |
| 1 | chr1 | − | 101609473 | 101609487 | 3.67e-07 | 0.018 | `AGGTGACAGAGGCCA` |
| 1 | chr2 | − | 216531519 | 216531533 | 3.67e-07 | 0.018 | `AGGTCAAGGAGTTCA` |
| 1 | chr8 | + | 141546779 | 141546793 | 3.8e-07 | 0.018 | `AGGTCAGTGAGTTCT` |
| 1 | chr1 | + | 148801589 | 148801603 | 3.94e-07 | 0.018 | `TGGTCACTGAGGTCA` |
| 1 | chr2 | + | 25048015 | 25048029 | 3.94e-07 | 0.018 | `AGGTCAAAAAGGTCG` |
| 1 | chrX | − | 7043378 | 7043392 | 4.16e-07 | 0.018 | `AGGTCACCAAGGACA` |
| 1 | chr7 | + | 41991181 | 41991195 | 4.24e-07 | 0.018 | `AGTTCACTGGGTTCA` |
| 1 | chr7 | + | 23359460 | 23359474 | 4.36e-07 | 0.018 | `AGGTCACTGAGGGCT` |
| 1 | chr8 | − | 146074883 | 146074897 | 4.38e-07 | 0.018 | `AGGTCACTGAGTGCT` |
| 1 | chr13 | + | 29630951 | 29630965 | 4.47e-07 | 0.018 | `GGGTCAATGAGGGCA` |
| 1 | chr7 | − | 25592856 | 25592870 | 4.57e-07 | 0.018 | `AGTTCAAAGAGGACA` |
| 1 | chr5 | + | 32212260 | 32212274 | 4.66e-07 | 0.018 | `AGTTCAAAGAGTACA` |
| 1 | chr11 | + | 72765802 | 72765816 | 4.66e-07 | 0.018 | `GGGTCACTGGGGTCA` |
| 1 | chr2 | − | 191243773 | 191243787 | 4.7e-07 | 0.018 | `AGTTCAACAAGTTCA` |
| 1 | chr2 | − | 161268856 | 161268870 | 4.72e-07 | 0.018 | `AGGCCACAGAGGTCA` |
| 1 | chr18 | − | 17966496 | 17966510 | 4.81e-07 | 0.018 | `AGGTCATAGAGGTCA` |
| 1 | chr18 | − | 19735183 | 19735197 | 4.97e-07 | 0.0182 | `AGGTCACTGAGTTTG` |
| 1 | chr3 | − | 40977898 | 40977912 | 5.03e-07 | 0.0182 | `AGTTCAGAAAGGTCA` |
| 1 | chr13 | + | 46123389 | 46123403 | 5.1e-07 | 0.0182 | `AGTTCAGAAAGTTCA` |
| 1 | chr2 | − | 157825939 | 157825953 | 5.22e-07 | 0.0182 | `AGGTCAATAAGGGCA` |
| 1 | chr5 | − | 75179414 | 75179428 | 5.26e-07 | 0.0182 | `AGTTCAGAGAGTGCA` |
| 1 | chr3 | − | 49257224 | 49257238 | 5.48e-07 | 0.0186 | `GGGTCAGAAAGTTCA` |
| 1 | chr22 | + | 48052038 | 48052052 | 5.74e-07 | 0.0192 | `GGGTCAGAGAGTGCA` |
| 1 | chr12 | − | 53664764 | 53664778 | 5.96e-07 | 0.0193 | `AGTTGAATGAGTTCA` |
| 1 | chr18 | + | 3583985 | 3583999 | 5.96e-07 | 0.0193 | `GGGTCAGAGAGTTCG` |
| 1 | chr1 | − | 207972888 | 207972902 | 6.26e-07 | 0.0193 | `AGGTCAGGGAGTTCA` |
| 1 | chr20 | + | 23262874 | 23262888 | 6.26e-07 | 0.0193 | `AGGTCAGGGAGTTCA` |
| 1 | chr6 | + | 51969572 | 51969586 | 6.38e-07 | 0.0193 | `AGGTCATTGAGGTCA` |
| 1 | chr6 | − | 138155093 | 138155107 | 6.38e-07 | 0.0193 | `AGGTCAAAGAGTACG` |
| 1 | chr6 | + | 2736672 | 2736686 | 6.47e-07 | 0.0193 | `AGGTCACCAGGTTCA` |
| 1 | chr19 | + | 62885001 | 62885015 | 6.5e-07 | 0.0193 | `AGTTCGCAGAGGTCA` |
| 1 | chr17 | + | 44650781 | 44650795 | 6.68e-07 | 0.0196 | `AGGTCAGAAAGGGCA` |
| 1 | chr12 | − | 27297405 | 27297419 | 7e-07 | 0.0202 | `AGTTCAAAGGGTTCA` |
| 1 | chr2 | + | 111642870 | 111642884 | 7.62e-07 | 0.0217 | `GGGTCAAAGGGGTCA` |
| 1 | chr22 | + | 48051971 | 48051985 | 7.74e-07 | 0.0218 | `GGGTCAGTGAGTGCA` |
| 1 | chr22 | + | 35971621 | 35971635 | 7.87e-07 | 0.0218 | `GGGTCAGAGAGGCCA` |
| 1 | chrX | − | 119643313 | 119643327 | 7.96e-07 | 0.0218 | `GGGTCAATGAGTTCT` |
| 1 | chr3 | − | 45000995 | 45001009 | 8.15e-07 | 0.0221 | `AGGTCACAGGGTTTA` |
| 1 | chr3 | + | 10237484 | 10237498 | 8.3e-07 | 0.0221 | `GGGTGAGAGAGGTCA` |
| 1 | chr16 | + | 10767363 | 10767377 | 8.48e-07 | 0.0221 | `AGGTCACTGGGGACA` |
| 1 | chr9 | − | 114586003 | 114586017 | 8.59e-07 | 0.0221 | `AGGTCACTGGGTACA` |
| 1 | chr11 | − | 35105614 | 35105628 | 8.59e-07 | 0.0221 | `GGGTCAATGAGTTTA` |
| 1 | chr8 | − | 105768101 | 105768115 | 9.12e-07 | 0.0232 | `AGTTCAGAGAGTTCT` |
| 1 | chr9 | − | 33294552 | 33294566 | 9.39e-07 | 0.0236 | `AAGTCAAAGAGTTCA` |
| 1 | chr12 | − | 2335601 | 2335615 | 9.58e-07 | 0.0238 | `AGGTCACGGAGGCCA` |
| 1 | chr5 | − | 72986296 | 72986310 | 1.01e-06 | 0.0249 | `AGATGACTGAGTTCA` |
| 1 | chr18 | + | 47227848 | 47227862 | 1.03e-06 | 0.025 | `AGGTCGGTGAGTTCA` |
| 1 | chr5 | − | 96529020 | 96529034 | 1.14e-06 | 0.0274 | `TGGTCAGAGAGTTCA` |
| 1 | chr22 | − | 49095625 | 49095639 | 1.16e-06 | 0.0275 | `AGTTCAAGGAGTTCA` |
| 1 | chr20 | − | 45820712 | 45820726 | 1.24e-06 | 0.029 | `GGGTGACTGAGGGCA` |
| 1 | chr8 | + | 105667455 | 105667469 | 1.25e-06 | 0.029 | `AGGTGGCAGAGGTCA` |
| 1 | chr1 | + | 210173058 | 210173072 | 1.3e-06 | 0.0296 | `AGTTCACTAAGTTCT` |
| 1 | chr12 | + | 132124208 | 132124222 | 1.3e-06 | 0.0296 | `AGTTCAGTGAGTTTA` |
| 1 | chr10 | − | 11691636 | 11691650 | 1.35e-06 | 0.0304 | `AGGCCAATGAGTTCA` |
| 1 | chr2 | − | 73365549 | 73365563 | 1.37e-06 | 0.0306 | `AGGTCGCCGAGTGCA` |
| 1 | chr8 | − | 10822266 | 10822280 | 1.44e-06 | 0.0318 | `GGGTCACAGGGGGCA` |
| 1 | chr6 | + | 124403749 | 124403763 | 1.49e-06 | 0.032 | `AGTTCATAGAGTTCA` |
| 1 | chr9 | + | 36995968 | 36995982 | 1.49e-06 | 0.032 | `AGTTCATAGAGTTCA` |
| 1 | chr1 | − | 95141283 | 95141297 | 1.51e-06 | 0.032 | `AGGTCAACGAGTCCT` |
| 1 | chr8 | + | 42158972 | 42158986 | 1.51e-06 | 0.032 | `AGGACACCGAGTTCA` |
| 1 | chr14 | − | 104463961 | 104463975 | 1.55e-06 | 0.0322 | `AGGTCAGAGGGGGCA` |
| 1 | chr17 | + | 39936551 | 39936565 | 1.55e-06 | 0.0322 | `AGGTCAGAGGGGGCA` |
| 1 | chr6 | − | 106718942 | 106718956 | 1.58e-06 | 0.0325 | `GGGCCACAGAGTTCA` |
| 1 | chr5 | + | 151022298 | 151022312 | 1.59e-06 | 0.0325 | `AGGTGAACGAGTTCT` |
| 1 | chr21 | + | 34270228 | 34270242 | 1.68e-06 | 0.034 | `TGGTCACAGAGGCCA` |
| 1 | chr20 | + | 23263190 | 23263204 | 1.73e-06 | 0.0343 | `AGGTCAGAGAGTCTA` |
| 1 | chr18 | + | 64811374 | 64811388 | 1.75e-06 | 0.0343 | `GGTTCACGGAGGTCA` |
| 1 | chr1 | + | 36381747 | 36381761 | 1.83e-06 | 0.0343 | `AGGTCATAAAGGTCA` |
| 1 | chr4 | + | 152885429 | 152885443 | 1.85e-06 | 0.0343 | `GAGTCACTGAGTTCA` |
| 1 | chr5 | − | 40443161 | 40443175 | 1.87e-06 | 0.0343 | `AGGTCAGCAAGGTTA` |
| 1 | chr6 | − | 3313035 | 3313049 | 1.87e-06 | 0.0343 | `GGGTCACTGGGTGCA` |
| 1 | chr19 | + | 8292538 | 8292552 | 1.87e-06 | 0.0343 | `AGGTCACCAAGGTCC` |
| 1 | chr2 | + | 129273094 | 129273108 | 1.92e-06 | 0.0343 | `AGGCCACAGAGTGCA` |
| 1 | chr5 | − | 5414272 | 5414286 | 1.92e-06 | 0.0343 | `AGTTCACTGAGGCTA` |
| 1 | chr3 | − | 116347706 | 116347720 | 1.93e-06 | 0.0343 | `GGTTGAATGAGGTCA` |
| 1 | chr15 | + | 86983765 | 86983779 | 1.93e-06 | 0.0343 | `GGGTCAGCGGGGTCA` |
| 1 | chr19 | − | 16604013 | 16604027 | 1.94e-06 | 0.0343 | `GGTTGAATGAGTTCA` |
| 1 | chr9 | + | 134847948 | 134847962 | 2.02e-06 | 0.0343 | `AGTTCACCGGGTGCA` |
| 1 | chr10 | − | 73404494 | 73404508 | 2.02e-06 | 0.0343 | `GGGGCACTGAGGTCA` |
| 1 | chr11 | − | 116973224 | 116973238 | 2.02e-06 | 0.0343 | `AGGTGACTGAGGCCG` |
| 1 | chr15 | + | 38191520 | 38191534 | 2.02e-06 | 0.0343 | `GGGTCAGGGAGGTCA` |
| 1 | chr18 | + | 55220849 | 55220863 | 2.02e-06 | 0.0343 | `AGGTGACTGAGGCCG` |
| 1 | chr20 | − | 47735357 | 47735371 | 2.02e-06 | 0.0343 | `GGGTCACAGAGTTTT` |
| 1 | chr4 | − | 15365197 | 15365211 | 2.04e-06 | 0.0343 | `GGGGCACTGAGTTCA` |
| 1 | chr17 | + | 38114327 | 38114341 | 2.04e-06 | 0.0343 | `GGGTCAACAAGTGCA` |
| 1 | chr9 | + | 116483891 | 116483905 | 2.05e-06 | 0.0343 | `AGATGAATGAGGTCA` |
| 1 | chr10 | + | 126879745 | 126879759 | 2.1e-06 | 0.0343 | `AGGTGAAGGAGTTCA` |
| 1 | chr17 | − | 52790263 | 52790277 | 2.1e-06 | 0.0343 | `AAGTCACTAAGGTCA` |
| 1 | chr2 | + | 73860861 | 73860875 | 2.11e-06 | 0.0343 | `AGGTCAATAAGGGCG` |
| 1 | chr2 | + | 85408932 | 85408946 | 2.11e-06 | 0.0343 | `AGGTCACTAGGGGCA` |
| 1 | chr2 | − | 191453399 | 191453413 | 2.13e-06 | 0.0343 | `AGGGCAGTGAGGTCA` |
| 1 | chr3 | + | 113529146 | 113529160 | 2.15e-06 | 0.0343 | `AGGTCACAGAGTATG` |
| 1 | chrX | − | 54087952 | 54087966 | 2.15e-06 | 0.0343 | `GAGTCACCGAGTTCA` |
| 1 | chr17 | − | 44625034 | 44625048 | 2.19e-06 | 0.0346 | `AGGTCAGTGAGGCTA` |
| 1 | chr6 | + | 170705377 | 170705391 | 2.25e-06 | 0.0354 | `GGTTCAGTGAGGTCG` |
| 1 | chr3 | − | 13108167 | 13108181 | 2.29e-06 | 0.0358 | `AGGTCAGCGGGGGCA` |
| 1 | chr17 | − | 9300436 | 9300450 | 2.39e-06 | 0.0369 | `AGATCAGAGAGGCCA` |
| 1 | chrX | + | 129054001 | 129054015 | 2.43e-06 | 0.0369 | `AGGTCAGGGAGTGCA` |
| 1 | chr17 | − | 30724255 | 30724269 | 2.43e-06 | 0.0369 | `AGGCCACTGAGTGCA` |
| 1 | chr19 | + | 12754247 | 12754261 | 2.43e-06 | 0.0369 | `AAGTCACAGAGGACA` |
| 1 | chr16 | + | 14358785 | 14358799 | 2.47e-06 | 0.0372 | `AAGTCACCAAGTTCA` |
| 1 | chr1 | + | 210172914 | 210172928 | 2.52e-06 | 0.0375 | `AGGTCATAGAGTCCA` |
| 1 | chr7 | − | 100515210 | 100515224 | 2.52e-06 | 0.0375 | `GGGTCACAGGGTTTA` |
| 1 | chr15 | − | 61757143 | 61757157 | 2.57e-06 | 0.0377 | `TGGTCAAAGAGGGCA` |
| 1 | chr16 | − | 55891771 | 55891785 | 2.57e-06 | 0.0377 | `AGATCAATGAGGTTA` |
| 1 | chr8 | − | 10839359 | 10839373 | 2.59e-06 | 0.0378 | `AGGTCAGTGGGTCCA` |
| 1 | chr20 | + | 45847127 | 45847141 | 2.62e-06 | 0.0378 | `GGGTCAACAAGGCCA` |
| 1 | chr17 | + | 2116772 | 2116786 | 2.65e-06 | 0.0381 | `TGGTGACCGAGGTCA` |
| 1 | chr3 | − | 23670142 | 23670156 | 2.7e-06 | 0.0386 | `AGGTCAGTGAGGTTT` |
| 1 | chr5 | − | 1366794 | 1366808 | 2.83e-06 | 0.0401 | `GGGTCACCGGGTCCA` |
| 1 | chr1 | − | 40278371 | 40278385 | 2.92e-06 | 0.0409 | `GGGTCAGTGAGGGCG` |
| 1 | chr6 | + | 80381209 | 80381223 | 2.92e-06 | 0.0409 | `GGGTCACGGAGGCCA` |
| 1 | chr1 | + | 181259716 | 181259730 | 2.95e-06 | 0.0411 | `GGGTGACCGGGGTCA` |
| 1 | chr20 | + | 23262854 | 23262868 | 3.13e-06 | 0.0431 | `AGGTCAGGGAGTCCA` |
| 1 | chr10 | − | 82002491 | 82002505 | 3.18e-06 | 0.0431 | `AGGTGGATGAGGTCA` |
| 1 | chr10 | + | 101407297 | 101407311 | 3.18e-06 | 0.0431 | `AGTTCAACAAGGTTA` |
| 1 | chr22 | + | 48052240 | 48052254 | 3.18e-06 | 0.0431 | `GGGTCAGCAAGTGCA` |
| 1 | chr2 | + | 231445978 | 231445992 | 3.28e-06 | 0.0441 | `AAGTCACCGAGGCCA` |
| 1 | chr3 | − | 109590040 | 109590054 | 3.34e-06 | 0.0447 | `AGGCCACTGAGGACA` |
| 1 | chr4 | − | 160317960 | 160317974 | 3.37e-06 | 0.0448 | `AGGTGATTGAGTTCA` |
| 1 | chr18 | + | 55568348 | 55568362 | 3.42e-06 | 0.0451 | `AGGTCATTGAGGACA` |
| 1 | chr15 | + | 62962578 | 62962592 | 3.44e-06 | 0.0451 | `GGTTCAGAGGGGTCA` |
| 1 | chr5 | − | 138925426 | 138925440 | 3.55e-06 | 0.046 | `AGTTCACAGAGCACA` |
| 1 | chr16 | + | 49704986 | 49705000 | 3.55e-06 | 0.046 | `AGGTCACGGAGGCCG` |
| 1 | chr11 | + | 33665872 | 33665886 | 3.61e-06 | 0.0465 | `GAGTCAATGAGTTCA` |
| 1 | chr22 | − | 39140905 | 39140919 | 3.69e-06 | 0.0473 | `AGGGCAAAGAGTGCA` |
| 1 | chr6 | − | 143166751 | 143166765 | 3.72e-06 | 0.0474 | `AGTTCAACAGGTTCA` |
| 1 | chr19 | − | 1599718 | 1599732 | 3.81e-06 | 0.0482 | `AGGCCACTGAGGTCT` |
| 1 | chr3 | − | 158289263 | 158289277 | 3.84e-06 | 0.0483 | `AGGTCACGGAGGACG` |
| 1 | chr8 | + | 131329100 | 131329114 | 3.87e-06 | 0.0484 | `AGGTCACTGGGGTTG` |
| 1 | chr2 | − | 230989605 | 230989619 | 3.9e-06 | 0.0485 | `GGGTGAAAGGGTTCA` |
| 1 | chr16 | − | 55891847 | 55891861 | 3.99e-06 | 0.0494 | `AGATCAGTGAGGTTA` |
| 1 | chr4 | + | 169519186 | 169519200 | 4.02e-06 | 0.0494 | `GGGTCAATGAGGACT` |
| 1 | chr6 | − | 111019006 | 111019020 | 4.04e-06 | 0.0494 | `GGGTCAATGAGTACT` |
| 1 | chr16 | − | 17226473 | 17226487 | 4.08e-06 | 0.0496 | `AGGTCACACAGGACA` |
| 1 | chr3 | − | 157886352 | 157886366 | 4.11e-06 | 0.0497 | `AGATCACAGGGTGCA` |
| 1 | chr2 | − | 98453934 | 98453948 | 4.14e-06 | 0.0498 | `AGGTCAACAAGGTTG` |
| 1 | chr12 | + | 46550582 | 46550596 | 4.21e-06 | 0.0503 | `GGGTGAGAGAGGACA` |
| 1 | chr3 | + | 4994414 | 4994428 | 4.3e-06 | 0.0509 | `AGGTCACAGAGCCCG` |
| 1 | chr14 | − | 92240478 | 92240492 | 4.33e-06 | 0.0509 | `GGTTCACCAAGTTCT` |
| 1 | chr17 | − | 1567316 | 1567330 | 4.37e-06 | 0.0509 | `AAGTCACTGGGTTCA` |
| 1 | chr17 | − | 58156311 | 58156325 | 4.39e-06 | 0.0509 | `GGGTCAGAGGGGGCA` |
| 1 | chr2 | + | 9755731 | 9755745 | 4.42e-06 | 0.0509 | `AGAGCACAGAGTTCA` |
| 1 | chr10 | − | 82002527 | 82002541 | 4.42e-06 | 0.0509 | `AGGTGAATGAGGTCC` |
| 1 | chr10 | − | 82002671 | 82002685 | 4.42e-06 | 0.0509 | `AGGTGAATGAGGTCC` |
| 1 | chr1 | − | 154212800 | 154212814 | 4.66e-06 | 0.0534 | `AGGGCAAAGAGGCCA` |
| 1 | chr20 | + | 45847260 | 45847274 | 4.7e-06 | 0.0535 | `GGGTCAAACAGGTCA` |
| 1 | chr18 | + | 4115859 | 4115873 | 4.73e-06 | 0.0535 | `AGGGCACTGGGTTCA` |
| 1 | chr1 | − | 110682603 | 110682617 | 4.76e-06 | 0.0536 | `GGGGCAGAGAGTTCA` |
| 1 | chr8 | + | 72918436 | 72918450 | 4.88e-06 | 0.0547 | `GGTTCACTGGGTGCA` |
| 1 | chr12 | − | 115844669 | 115844683 | 5.01e-06 | 0.0559 | `AGGGCAAAGAGGACA` |
| 1 | chr17 | − | 70246195 | 70246209 | 5.05e-06 | 0.056 | `AGGGCAACAAGGTCA` |
| 1 | chr5 | + | 131037847 | 131037861 | 5.09e-06 | 0.0561 | `AGGGCAACAAGTTCA` |
| 1 | chrX | + | 151750258 | 151750272 | 5.16e-06 | 0.0561 | `AGTTCAGTGGGTGCA` |
| 1 | chr11 | − | 59979928 | 59979942 | 5.16e-06 | 0.0561 | `AGTTCAGTGGGTGCA` |
| 1 | chr16 | + | 11743407 | 11743421 | 5.16e-06 | 0.0561 | `AGATCAATGAGTGCG` |
| 1 | chr20 | − | 51702595 | 51702609 | 5.24e-06 | 0.0563 | `AGATCAAAAAGGACA` |
| 1 | chr1 | − | 26010042 | 26010056 | 5.28e-06 | 0.0563 | `GGGGCACAGAGGGCA` |
| 1 | chr6 | − | 32253475 | 32253489 | 5.28e-06 | 0.0563 | `GGGTCGCAGGGTTCA` |
| 1 | chr1 | − | 45048004 | 45048018 | 5.31e-06 | 0.0563 | `GGGGCACAGAGTGCA` |
| 1 | chrX | − | 134483587 | 134483601 | 5.31e-06 | 0.0563 | `AGGCCAACGAGTGCA` |
| 1 | chr1 | − | 201597923 | 201597937 | 5.41e-06 | 0.0568 | `AGGACACAGAGGTCT` |
| 1 | chr5 | + | 55009705 | 55009719 | 5.41e-06 | 0.0568 | `AGGTGAACAAGGTTA` |
| 1 | chr1 | − | 195123740 | 195123754 | 5.5e-06 | 0.0568 | `AGTTCAGAGAGTTTT` |
| 1 | chr8 | − | 125770148 | 125770162 | 5.5e-06 | 0.0568 | `AGTTCAAGGAGGACA` |
| 1 | chr8 | − | 134563402 | 134563416 | 5.5e-06 | 0.0568 | `GAGTCAGTGAGTTCA` |
| 1 | chr4 | + | 39924391 | 39924405 | 5.59e-06 | 0.0569 | `AGGCCAGAGAGGGCA` |
| 1 | chr22 | − | 41993723 | 41993737 | 5.59e-06 | 0.0569 | `AGGGCAGAGAGGGCA` |
| 1 | chr10 | + | 89912836 | 89912850 | 5.63e-06 | 0.0569 | `GGGTCAATGGGTTCT` |
| 1 | chr17 | + | 44645930 | 44645944 | 5.63e-06 | 0.0569 | `GGTTGACTGAGTTTA` |
| 1 | chr22 | + | 36010180 | 36010194 | 5.63e-06 | 0.0569 | `GGGTCAATGGGTTCT` |
| 1 | chr1 | + | 95141274 | 95141288 | 5.68e-06 | 0.057 | `AGTTCAGTGAGGACT` |
| 1 | chr22 | + | 40195131 | 40195145 | 5.71e-06 | 0.0571 | `AGGTGAGCGAGCTCA` |
| 1 | chr1 | + | 87344583 | 87344597 | 5.89e-06 | 0.0584 | `AGTTCAGGAAGTTCA` |
| 1 | chr18 | − | 59755513 | 59755527 | 5.89e-06 | 0.0584 | `GGATCACAGAGTGCG` |
| 1 | chr1 | + | 165898896 | 165898910 | 5.94e-06 | 0.0585 | `GGGCCAGTGAGTTCA` |
| 1 | chr11 | + | 68580523 | 68580537 | 5.98e-06 | 0.0587 | `AGGTGAGAGAGGGTA` |
| 1 | chr12 | − | 14814356 | 14814370 | 6.05e-06 | 0.0587 | `AGGTGACCAAGCTCA` |
| 1 | chr8 | − | 142065797 | 142065811 | 6.09e-06 | 0.0587 | `AGTGCACTGAGGGCA` |
| 1 | chrX | − | 96468349 | 96468363 | 6.09e-06 | 0.0587 | `GAGTCACTGAGTGCA` |
| 1 | chr22 | + | 20715139 | 20715153 | 6.09e-06 | 0.0587 | `GGTTCACTGGGGCCA` |
| 1 | chr1 | − | 67913170 | 67913184 | 6.13e-06 | 0.0587 | `AGTTCAGGGAGTGCA` |
| 1 | chr2 | + | 125846118 | 125846132 | 6.22e-06 | 0.0587 | `AGGTGAACAGGGTCA` |
| 1 | chr4 | + | 152885560 | 152885574 | 6.22e-06 | 0.0587 | `GGGTGACAAGGGTCA` |
| 1 | chr9 | − | 5548504 | 5548518 | 6.27e-06 | 0.0587 | `AGTTCATTGAGTGCA` |
| 1 | chr15 | − | 43534631 | 43534645 | 6.27e-06 | 0.0587 | `GGGTGACAAGGTTCA` |
| 1 | chr5 | + | 139205135 | 139205149 | 6.31e-06 | 0.0587 | `AGGTCAATGGGTTCC` |
| 1 | chr12 | − | 26843913 | 26843927 | 6.31e-06 | 0.0587 | `GGTTCAAAGAGGTTG` |
| 1 | chr3 | − | 151298218 | 151298232 | 6.36e-06 | 0.0587 | `AGTTCACTGAGGCTG` |
| 1 | chr11 | − | 59979627 | 59979641 | 6.36e-06 | 0.0587 | `AGGTCCCTGAGGACA` |
| 1 | chr12 | + | 93480130 | 93480144 | 6.36e-06 | 0.0587 | `AGTTCACTGAGGCTG` |
| 1 | chr14 | + | 94798341 | 94798355 | 6.44e-06 | 0.0592 | `GGTTCACTGAGTTTT` |
| 1 | chr3 | − | 14668135 | 14668149 | 6.49e-06 | 0.0592 | `AGGTCATAGAGTGCG` |
| 1 | chr1 | + | 232725011 | 232725025 | 6.53e-06 | 0.0592 | `GGGGCACTGAGGGCA` |
| 1 | chr16 | + | 2195184 | 2195198 | 6.53e-06 | 0.0592 | `GGGTCAGGGAGGGCA` |
| 1 | chr11 | − | 69209934 | 69209948 | 6.57e-06 | 0.0593 | `GGGTCAGGGAGTGCA` |
| 1 | chr8 | + | 104021939 | 104021953 | 6.62e-06 | 0.0596 | `GAGTCACCAAGGTCA` |
| 1 | chr9 | + | 35832910 | 35832924 | 6.68e-06 | 0.0597 | `GGGTCAACCAGGTCA` |
| 1 | chr1 | − | 211221739 | 211221753 | 6.73e-06 | 0.0597 | `AGTTCACAGGGTTCC` |
| 1 | chr8 | + | 142197568 | 142197582 | 6.73e-06 | 0.0597 | `AGTTGACTGAGTTTG` |
| 1 | chr9 | + | 115319535 | 115319549 | 6.78e-06 | 0.0599 | `GGGTCAGAGGGTTCT` |
| 1 | chr2 | − | 61097764 | 61097778 | 6.83e-06 | 0.0599 | `AGGTGAAGGAGTGCA` |
| 1 | chr7 | + | 5701263 | 5701277 | 6.83e-06 | 0.0599 | `AGTTCAATGACTTCA` |
| 1 | chr1 | + | 181259725 | 181259739 | 6.92e-06 | 0.0604 | `GGGTCACCAAGTCCT` |
| 1 | chr1 | − | 158865070 | 158865084 | 7.16e-06 | 0.0623 | `AGTTCATCGAGTGCA` |
| 1 | chr15 | − | 68543048 | 68543062 | 7.2e-06 | 0.0624 | `GGGTCACAGGGTTCC` |
| 1 | chr3 | − | 42029444 | 42029458 | 7.25e-06 | 0.0625 | `GGGTCATAGAGGACA` |
| 1 | chr1 | − | 202697612 | 202697626 | 7.3e-06 | 0.0627 | `GGGTGAGTGGGTTCA` |
| 1 | chr7 | − | 154720876 | 154720890 | 7.36e-06 | 0.0627 | `GGGTCATCAAGTTCA` |
| 1 | chr21 | − | 42157753 | 42157767 | 7.36e-06 | 0.0627 | `AGGCGAGAGAGGTCA` |
| 1 | chr4 | + | 55127720 | 55127734 | 7.49e-06 | 0.0636 | `AGGCCAGAGAGGACA` |
| 1 | chr6 | + | 7828117 | 7828131 | 7.53e-06 | 0.0637 | `AGGTCAAAGGGGATA` |
| 1 | chr11 | − | 36722192 | 36722206 | 7.64e-06 | 0.0641 | `AGTTCAGGGAGGCCA` |
| 1 | chr19 | − | 45996568 | 45996582 | 7.64e-06 | 0.0641 | `GGGGCACCGAGTTCG` |
| 1 | chr15 | + | 73017180 | 73017194 | 7.75e-06 | 0.0648 | `CGGTCACCGGGTTCA` |
| 1 | chr3 | + | 197828819 | 197828833 | 7.95e-06 | 0.0662 | `AAGTCAATGGGTTCA` |
| 1 | chr5 | + | 139204977 | 139204991 | 8e-06 | 0.0663 | `AGATCGGTGAGTTCA` |
| 1 | chr2 | + | 64998668 | 64998682 | 8.05e-06 | 0.0665 | `AGTTCAATAAGGGCT` |
| 1 | chr8 | − | 129303686 | 129303700 | 8.11e-06 | 0.0665 | `GGGTCACAGGGTTTG` |
| 1 | chr22 | + | 49095939 | 49095953 | 8.11e-06 | 0.0665 | `AGGTCGGGGAGGTCA` |
| 1 | chr17 | − | 30929801 | 30929815 | 8.19e-06 | 0.0666 | `GGGTCATAGAGTTCT` |
| 1 | chr20 | + | 23262894 | 23262908 | 8.19e-06 | 0.0666 | `AGGTCGGGGAGTTCA` |
| 1 | chr5 | − | 145542285 | 145542299 | 8.31e-06 | 0.0674 | `AGGTCGCCAGGGTCA` |
| 1 | chr1 | + | 204919919 | 204919933 | 8.37e-06 | 0.0676 | `AGGGCACCAAGGGCA` |
| 1 | chr1 | − | 39229117 | 39229131 | 8.51e-06 | 0.0683 | `AGGTCATCAAGGGCA` |
| 1 | chr11 | − | 2987483 | 2987497 | 8.56e-06 | 0.0683 | `AGGGCAATGGGTTCA` |
| 1 | chr16 | + | 66109643 | 66109657 | 8.56e-06 | 0.0683 | `GGGTGAGGGAGGTCA` |
| 1 | chr19 | − | 43228919 | 43228933 | 8.62e-06 | 0.0686 | `AGGTCAAAGACTCCA` |
| 1 | chrX | + | 38545496 | 38545510 | 8.69e-06 | 0.0686 | `GGGTGAAAGAGGACG` |
| 1 | chr11 | + | 120943214 | 120943228 | 8.75e-06 | 0.0686 | `GGGTCAAAGAGGCCC` |
| 1 | chr14 | − | 105536835 | 105536849 | 8.75e-06 | 0.0686 | `GGGGCACTGAGGACA` |
| 1 | chr15 | − | 19962601 | 19962615 | 8.75e-06 | 0.0686 | `GGGGCACTGAGGACA` |
| 1 | chr2 | − | 191586459 | 191586473 | 8.91e-06 | 0.0696 | `AGATGAATGAGTACA` |
| 1 | chr6 | − | 80416343 | 80416357 | 9.09e-06 | 0.0707 | `AGGTCAAGAGGTTCA` |
| 1 | chr6 | − | 166675015 | 166675029 | 9.22e-06 | 0.0715 | `GGTTCAGAGAGTGTA` |
| 1 | chr1 | − | 1700624 | 1700638 | 9.3e-06 | 0.0718 | `AGTTCAGCAAGTGCG` |
| 1 | chr17 | + | 35964007 | 35964021 | 9.42e-06 | 0.0725 | `GGTTCAGAGAGTTTG` |
| 1 | chr16 | − | 51685874 | 51685888 | 9.53e-06 | 0.0731 | `GGGTCACCGGGTACG` |
| 1 | chr4 | − | 7824309 | 7824323 | 9.72e-06 | 0.0738 | `GGGTCACACAGGCCA` |
| 1 | chr12 | − | 91457251 | 91457265 | 9.72e-06 | 0.0738 | `AGGTCACAAAGGATT` |
| 1 | chr22 | + | 48052112 | 48052126 | 9.72e-06 | 0.0738 | `AGGTCCACGAGTGCA` |
| 1 | chr8 | − | 96312878 | 96312892 | 9.86e-06 | 0.0745 | `AGGTCAGGAAGTACA` |
| 1 | chr1 | − | 148398446 | 148398460 | 9.92e-06 | 0.0747 | `AGAGCAATGAGTTCA` |
| 1 | chr3 | − | 120781261 | 120781275 | 1e-05 | 0.0751 | `GGGTCACCGGGTTCC` |
| 1 | chr17 | + | 74225773 | 74225787 | 1e-05 | 0.0751 | `AGGCCAAGGAGGTCA` |
| 1 | chr3 | + | 128956995 | 128957009 | 1.04e-05 | 0.0773 | `AGGTCAATGGGTTTT` |
| 1 | chr4 | − | 14467264 | 14467278 | 1.04e-05 | 0.0773 | `AGGTCAAGGAGTACT` |
| 1 | chr11 | + | 36722135 | 36722149 | 1.04e-05 | 0.0773 | `AGGTCAGAGGGGACT` |
| 1 | chr5 | + | 158208446 | 158208460 | 1.05e-05 | 0.0775 | `AGATCAAGAAGTTCA` |
| 1 | chr16 | + | 30569369 | 30569383 | 1.08e-05 | 0.0797 | `GGTTCAACGAGGACT` |
| 1 | chr12 | − | 29196556 | 29196570 | 1.09e-05 | 0.0799 | `AGGTCACTAGGTGTA` |
| 1 | chr2 | − | 12786296 | 12786310 | 1.1e-05 | 0.0801 | `AGGCCACTGAGTTCC` |
| 1 | chr11 | − | 8660838 | 8660852 | 1.11e-05 | 0.0806 | `CGGTCACAGAGGCCG` |
| 1 | chr11 | + | 34219118 | 34219132 | 1.11e-05 | 0.0804 | `AGTTCACAGGGTTTT` |
| 1 | chr2 | − | 149133249 | 149133263 | 1.12e-05 | 0.0806 | `AGTTCACAGACTCCA` |
| 1 | chr2 | + | 231233665 | 231233679 | 1.12e-05 | 0.0806 | `AGGTGGCCGGGGTCA` |
| 1 | chr5 | + | 132607992 | 132608006 | 1.12e-05 | 0.0806 | `AGGTGAGTGGGGACA` |
| 1 | chr14 | − | 102304457 | 102304471 | 1.13e-05 | 0.0809 | `AGGACAACGAGGCCA` |
| 1 | chr7 | − | 22621416 | 22621430 | 1.14e-05 | 0.0812 | `AGTTCATTGGGTTCA` |
| 1 | chr1 | − | 171512852 | 171512866 | 1.15e-05 | 0.0812 | `GGTTGAATGGGTTCA` |
| 1 | chr4 | − | 88068949 | 88068963 | 1.15e-05 | 0.0812 | `AAGTCATAGAGTTCA` |
| 1 | chr5 | + | 138925357 | 138925371 | 1.15e-05 | 0.0815 | `AGATCACAGAGCTTA` |
| 1 | chr4 | − | 185501961 | 185501975 | 1.17e-05 | 0.0827 | `AGGTGACTGAGTTTC` |
| 1 | chr6 | + | 33237279 | 33237293 | 1.19e-05 | 0.0836 | `GGGTCAGGGGGTTCA` |
| 1 | chr17 | − | 60411582 | 60411596 | 1.2e-05 | 0.0837 | `GGGTGACTGGGTTCT` |
| 1 | chr17 | + | 63746779 | 63746793 | 1.2e-05 | 0.0837 | `GGGTGACTGGGTTCT` |
| 1 | chr3 | + | 13031925 | 13031939 | 1.21e-05 | 0.0837 | `GGGTCACAGAATACA` |
| 1 | chr4 | − | 186013060 | 186013074 | 1.21e-05 | 0.0837 | `GGGTCCCTGAGGTCG` |
| 1 | chr8 | + | 123865036 | 123865050 | 1.24e-05 | 0.0857 | `AGGGCAGCGAGTTTA` |
| 1 | chr5 | + | 43639561 | 43639575 | 1.25e-05 | 0.0857 | `AGGTCATAGAGGACT` |
| 1 | chr16 | − | 11364708 | 11364722 | 1.25e-05 | 0.0857 | `GGGTGACTCAGTTCA` |
| 1 | chr12 | + | 122999 | 123013 | 1.26e-05 | 0.0857 | `GGGTGCCAGAGTTCA` |
| 1 | chr12 | + | 123029 | 123043 | 1.26e-05 | 0.0857 | `GGGTGCCAGAGTTCA` |
| 1 | chr16 | − | 11671901 | 11671915 | 1.26e-05 | 0.0857 | `AGGTCACCAGGTTTG` |
| 1 | chr5 | + | 106935039 | 106935053 | 1.29e-05 | 0.0873 | `AGTTCACTGAATCCA` |
| 1 | chr1 | + | 155413055 | 155413069 | 1.3e-05 | 0.0873 | `AGAGCACAGAGTGCA` |
| 1 | chr4 | + | 54309388 | 54309402 | 1.3e-05 | 0.0873 | `GGGTGGCTAAGTTCA` |
| 1 | chr9 | − | 92950081 | 92950095 | 1.3e-05 | 0.0873 | `AGTGCAGAGAGTGCA` |
| 1 | chr10 | + | 43212732 | 43212746 | 1.31e-05 | 0.0876 | `GGGTCACGGGGTGCA` |
| 1 | chr4 | + | 114705653 | 114705667 | 1.34e-05 | 0.089 | `AGTTCAAAGAGTCTT` |
| 1 | chr11 | − | 63750888 | 63750902 | 1.35e-05 | 0.089 | `GGGTGGCTGAGTGCA` |
| 1 | chr17 | − | 16225380 | 16225394 | 1.35e-05 | 0.089 | `AGGTCAACAAGGCCC` |
| 1 | chr6 | − | 106670135 | 106670149 | 1.36e-05 | 0.089 | `GGGTCATCGGGGTCA` |
| 1 | chr20 | + | 51889590 | 51889604 | 1.36e-05 | 0.089 | `AAGCCACTGAGGTCA` |
| 1 | chr7 | + | 100019491 | 100019505 | 1.37e-05 | 0.089 | `AGGGCACTGGGGGCA` |
| 1 | chr8 | − | 98725241 | 98725255 | 1.37e-05 | 0.089 | `GGGTCACCGGGTCTA` |
| 1 | chr14 | − | 75016616 | 75016630 | 1.37e-05 | 0.089 | `AAGTCAGGGAGTTCA` |
| 1 | chr22 | − | 40588910 | 40588924 | 1.37e-05 | 0.089 | `AGGTCAGAAAGCTTA` |
| 1 | chr17 | − | 30415257 | 30415271 | 1.38e-05 | 0.0892 | `AGTTCCCTGAGTCCA` |
| 1 | chr14 | + | 21715758 | 21715772 | 1.39e-05 | 0.0894 | `AAGTCATTGAGTTCA` |
| 1 | chr2 | − | 233673548 | 233673562 | 1.4e-05 | 0.0894 | `GGGTGAGCGAGCTCA` |
| 1 | chr2 | + | 10140652 | 10140666 | 1.41e-05 | 0.0894 | `AGTTCACTCAGTTTA` |
| 1 | chr5 | + | 138753458 | 138753472 | 1.42e-05 | 0.0894 | `AGTTCAGCAAGTGTA` |
| 1 | chr9 | + | 130685061 | 130685075 | 1.42e-05 | 0.0894 | `GGGTCATGGAGTTCA` |
| 1 | chr15 | − | 62747025 | 62747039 | 1.42e-05 | 0.0894 | `AGGTCAGCCAGTCCA` |
| 1 | chr17 | − | 20542987 | 20543001 | 1.42e-05 | 0.0894 | `AGGTCACTGGGGCTG` |
| 1 | chr17 | − | 60412731 | 60412745 | 1.42e-05 | 0.0894 | `AGGACACAGAGGACG` |
| 1 | chr17 | + | 63745630 | 63745644 | 1.42e-05 | 0.0894 | `AGGACACAGAGGACG` |
| 1 | chr21 | + | 15500865 | 15500879 | 1.42e-05 | 0.0894 | `GGGTCAGCAAGGGCT` |
| 1 | chr14 | − | 105536913 | 105536927 | 1.43e-05 | 0.0897 | `GGGTCACCCAGGACA` |
| 1 | chr7 | + | 92280660 | 92280674 | 1.44e-05 | 0.09 | `AGTGCAATGAGTACA` |
| 1 | chr1 | + | 201597819 | 201597833 | 1.46e-05 | 0.0906 | `AATTCAAAGGGGTCA` |
| 1 | chr9 | + | 91269339 | 91269353 | 1.46e-05 | 0.0906 | `GGGACACTGAGGACA` |
| 1 | chr4 | + | 185973598 | 185973612 | 1.47e-05 | 0.0906 | `AGTTCACAGAGCCCT` |
| 1 | chr20 | + | 62053494 | 62053508 | 1.47e-05 | 0.0906 | `AGGGCACAGGGGACA` |
| 1 | chr20 | + | 62053536 | 62053550 | 1.47e-05 | 0.0906 | `AGGGCACAGGGGACA` |
| 1 | chr20 | + | 62053578 | 62053592 | 1.47e-05 | 0.0906 | `AGGGCACAGGGGACA` |
| 1 | chr7 | + | 92188274 | 92188288 | 1.48e-05 | 0.0909 | `AGGTCAGAGAAGTTA` |
| 1 | chr1 | − | 111566231 | 111566245 | 1.49e-05 | 0.0912 | `AGGTGAAAAAGTATA` |
| 1 | chr16 | − | 3161556 | 3161570 | 1.51e-05 | 0.0921 | `AGGTCACTGGGGATG` |
| 1 | chr5 | + | 131037795 | 131037809 | 1.53e-05 | 0.0927 | `AGGCCAGAAAGGGCA` |
| 1 | chr5 | − | 1845820 | 1845834 | 1.54e-05 | 0.0927 | `AAGTCAAAAAGTTTA` |
| 1 | chr17 | + | 35992416 | 35992430 | 1.54e-05 | 0.0927 | `GGGTGAGTGAGGACG` |
| 1 | chr6 | + | 26381535 | 26381549 | 1.55e-05 | 0.0927 | `CGGTCACCAAGTACA` |
| 1 | chr8 | − | 125003581 | 125003595 | 1.55e-05 | 0.0927 | `AGGGCACCGGGTGCA` |
| 1 | chr9 | + | 91269419 | 91269433 | 1.55e-05 | 0.0927 | `CAGTCACAGAGGTCA` |
| 1 | chr14 | + | 50358418 | 50358432 | 1.55e-05 | 0.0927 | `AGGTCGGTGAGTTCC` |
| 1 | chr13 | − | 33201072 | 33201086 | 1.56e-05 | 0.0927 | `AAATGACAGAGTTCA` |
| 1 | chr18 | − | 59136411 | 59136425 | 1.56e-05 | 0.0927 | `AGTTCGGTGGGGTCA` |
| 1 | chr6 | − | 24992002 | 24992016 | 1.57e-05 | 0.0928 | `AGTGCAGTGAGGGCA` |
| 1 | chr9 | + | 4752988 | 4753002 | 1.57e-05 | 0.0928 | `AGGTCATGAAGGTCA` |
| 1 | chr1 | + | 2480906 | 2480920 | 1.58e-05 | 0.0929 | `GGGTCATAGAGTGCG` |
| 1 | chr13 | − | 35519046 | 35519060 | 1.58e-05 | 0.0929 | `AGGTCATGAAGTTCA` |
| 1 | chr6 | − | 22107008 | 22107022 | 1.59e-05 | 0.093 | `AGTTCAATGGGTGTA` |
| 1 | chr1 | + | 204919910 | 204919924 | 1.6e-05 | 0.093 | `AGGGCACGGAGGGCA` |
| 1 | chr4 | − | 184602638 | 184602652 | 1.6e-05 | 0.093 | `AGGCCACGGAGGGCA` |
| 1 | chr18 | − | 12857920 | 12857934 | 1.6e-05 | 0.093 | `AGATCATTGAGGGCA` |
| 1 | chr19 | − | 1600017 | 1600031 | 1.61e-05 | 0.0935 | `AAGTCACACAGTTCA` |
| 1 | chr3 | − | 120554063 | 120554077 | 1.63e-05 | 0.0939 | `GGGTCAGAAGGGACA` |
| 1 | chr15 | − | 66874909 | 66874923 | 1.63e-05 | 0.0939 | `AAGTCACAGGGTTTA` |
| 1 | chr15 | + | 72879855 | 72879869 | 1.63e-05 | 0.0939 | `AGGTCATGGAGGGCA` |
| 1 | chr7 | − | 22621425 | 22621439 | 1.66e-05 | 0.095 | `GGTTCATCAAGTTCA` |
| 1 | chr17 | + | 24320398 | 24320412 | 1.66e-05 | 0.095 | `AGTTCAGGAAGGGCA` |
| 1 | chr5 | + | 106759279 | 106759293 | 1.68e-05 | 0.0959 | `AGGTCAGCCAGTTCT` |
| 1 | chr2 | − | 216531510 | 216531524 | 1.69e-05 | 0.096 | `AGTTCATTAAGGGCA` |
| 1 | chr20 | + | 23263170 | 23263184 | 1.69e-05 | 0.096 | `AGGTCAGGGGGTCCA` |
| 1 | chr5 | − | 89741335 | 89741349 | 1.71e-05 | 0.0968 | `AGGTCCCTGAGGCCG` |
| 1 | chr9 | + | 126002387 | 126002401 | 1.71e-05 | 0.0968 | `CGGTCACTGAGTTTG` |
| 1 | chr1 | − | 28372056 | 28372070 | 1.73e-05 | 0.0971 | `AGTTCATAAAGTCCA` |
| 1 | chr3 | − | 196746153 | 196746167 | 1.75e-05 | 0.098 | `AGGTCATACAGTTCA` |
| 1 | chr7 | + | 23025077 | 23025091 | 1.75e-05 | 0.098 | `GGATCAATGAGGTCC` |
| 1 | chr17 | + | 77779586 | 77779600 | 1.75e-05 | 0.098 | `AGTTCAGGGAGGGCG` |
| 1 | chr19 | + | 63611500 | 63611514 | 1.79e-05 | 0.0995 | `AGGTCACCGGGGTTC` |
| 1 | chr22 | − | 40588427 | 40588441 | 1.81e-05 | 0.1 | `AGGCCACAAAGCTCA` |
| 1 | chr1 | − | 67924612 | 67924626 | 1.82e-05 | 0.1 | `GGGTCGCTGGGGGCA` |
| 1 | chr8 | − | 146074793 | 146074807 | 1.82e-05 | 0.1 | `AGATCACTGAGGTTC` |
| 1 | chr20 | + | 36896492 | 36896506 | 1.82e-05 | 0.1 | `AGTTCATAGAGTCCG` |
| 1 | chr2 | − | 30413740 | 30413754 | 1.86e-05 | 0.102 | `GGGGCACTGAGGGCG` |
| 1 | chr3 | − | 184362390 | 184362404 | 1.87e-05 | 0.102 | `AGGTGAGAAGGGTCG` |
| 1 | chr12 | + | 67488815 | 67488829 | 1.87e-05 | 0.102 | `AGGTCACGGGGGCCG` |
| 1 | chr14 | + | 93495394 | 93495408 | 1.87e-05 | 0.102 | `GAGTCACAGAGGACG` |
| 1 | chr11 | − | 65076625 | 65076639 | 1.88e-05 | 0.103 | `GAGTCAATGGGGTCA` |
| 1 | chr9 | − | 116483863 | 116483877 | 1.89e-05 | 0.103 | `AGTGCAATGGGGTCA` |
| 1 | chr12 | + | 93480121 | 93480135 | 1.91e-05 | 0.103 | `AGTAGAAAGAGTTCA` |
| 1 | chr19 | + | 55091140 | 55091154 | 1.92e-05 | 0.104 | `GGGTCAACGGGGTTG` |
| 1 | chr9 | + | 113767932 | 113767946 | 1.93e-05 | 0.104 | `GGGGGACAGAGGGCA` |
| 1 | chr8 | − | 90839471 | 90839485 | 1.94e-05 | 0.104 | `AGGTCGGCGAGTTTG` |
| 1 | chr19 | + | 16050543 | 16050557 | 1.94e-05 | 0.104 | `AGTTCGAAAAGTTCT` |
| 1 | chr19 | + | 14352990 | 14353004 | 1.97e-05 | 0.105 | `AGGCCAAAGGGTTCG` |
| 1 | chr11 | + | 72765811 | 72765825 | 1.98e-05 | 0.105 | `GGGTCACGAAGTGCG` |
| 1 | chr18 | + | 44733434 | 44733448 | 1.98e-05 | 0.105 | `GAGTCAGAGAGTTTA` |
| 1 | chr17 | − | 34278800 | 34278814 | 2e-05 | 0.106 | `GGGTCAGACAGTTCG` |
| 1 | chr2 | − | 122798131 | 122798145 | 2.01e-05 | 0.107 | `AGGTCATCGAGTTTT` |
| 1 | chr17 | − | 38024488 | 38024502 | 2.05e-05 | 0.108 | `GGGTCCCTGGGGTCA` |
| 1 | chr18 | − | 59755532 | 59755546 | 2.05e-05 | 0.108 | `AGATGACCAGGTTCA` |
| 1 | chr5 | − | 138925464 | 138925478 | 2.06e-05 | 0.108 | `AAGTCAGAAAGGTCT` |
| 1 | chr17 | + | 59451670 | 59451684 | 2.06e-05 | 0.108 | `AGGTGACAAGGTGCG` |
| 1 | chr20 | + | 23262973 | 23262987 | 2.08e-05 | 0.109 | `AGGTCAGGCAGTTCA` |
| 1 | chr5 | − | 5414347 | 5414361 | 2.09e-05 | 0.109 | `AGGTCAACAGGGGTA` |
| 1 | chr6 | + | 18264026 | 18264040 | 2.09e-05 | 0.109 | `GGGTCGCCGGGTTCG` |
| 1 | chr22 | − | 40139913 | 40139927 | 2.11e-05 | 0.11 | `GGGTCGCGGAGGGCA` |
| 1 | chr3 | − | 144183560 | 144183574 | 2.12e-05 | 0.11 | `GAGTCAACGGGTTCA` |
| 1 | chr6 | + | 86444891 | 86444905 | 2.12e-05 | 0.11 | `AGGCCAGCAAGTTCG` |
| 1 | chr4 | − | 55127603 | 55127617 | 2.15e-05 | 0.111 | `GGGTGAGAGGGGACA` |
| 1 | chr16 | + | 3161905 | 3161919 | 2.19e-05 | 0.113 | `GGTTCACCAGGGTCT` |
| 1 | chr14 | − | 106218688 | 106218702 | 2.23e-05 | 0.114 | `AGGACACTGGGGGCA` |
| 1 | chr5 | − | 89741326 | 89741340 | 2.26e-05 | 0.115 | `AGGCCGCTGAGGTCG` |
| 1 | chr3 | − | 178552582 | 178552596 | 2.27e-05 | 0.116 | `AGGTGAACAGGGACA` |
| 1 | chr1 | + | 28374665 | 28374679 | 2.3e-05 | 0.117 | `AAGTGAGTGAGTGCA` |
| 1 | chr2 | + | 134705793 | 134705807 | 2.3e-05 | 0.117 | `AGATCACTGGGTTTG` |
| 1 | chr7 | − | 133768241 | 133768255 | 2.3e-05 | 0.117 | `CGGTCATCGAGTTCA` |
| 1 | chr7 | − | 134504517 | 134504531 | 2.31e-05 | 0.117 | `AGGTCAGAGGGCTCT` |
| 1 | chr8 | + | 67755371 | 67755385 | 2.31e-05 | 0.117 | `GGGTCACTAGGTTCC` |
| 1 | chr3 | + | 47397429 | 47397443 | 2.32e-05 | 0.117 | `GGGTGGCTGGGTTCA` |
| 1 | chr17 | − | 25714945 | 25714959 | 2.34e-05 | 0.117 | `AGGTGAATGGGTGCT` |
| 1 | chr3 | + | 45978769 | 45978783 | 2.35e-05 | 0.117 | `AGGTCATCCAGGTCA` |
| 1 | chr10 | − | 126375401 | 126375415 | 2.35e-05 | 0.117 | `GGGCCAAGGAGTTCA` |
| 1 | chr3 | − | 25235058 | 25235072 | 2.36e-05 | 0.118 | `AGGTCATCCAGTTCA` |
| 1 | chr14 | − | 106240888 | 106240902 | 2.36e-05 | 0.118 | `AGATGACAGGGGTTA` |
| 1 | chr8 | − | 144179102 | 144179116 | 2.38e-05 | 0.118 | `GGGTGAGAGGGGTCT` |
| 1 | chr5 | − | 149764046 | 149764060 | 2.4e-05 | 0.118 | `AGGGCGCCAAGGTCA` |
| 1 | chrX | + | 46373253 | 46373267 | 2.4e-05 | 0.118 | `TGGCCACAGAGTGCA` |
| 1 | chr10 | + | 71164636 | 71164650 | 2.41e-05 | 0.118 | `AGGTGGCTGAGTTTG` |
| 1 | chr15 | + | 88444305 | 88444319 | 2.41e-05 | 0.118 | `GGGCCAGTGAGTTCT` |
| 1 | chr21 | + | 33373363 | 33373377 | 2.41e-05 | 0.118 | `GGGTGAATGAAGTCA` |
| 1 | chr12 | + | 120951390 | 120951404 | 2.44e-05 | 0.119 | `GGGAGAATGAGTTCA` |
| 1 | chr1 | + | 177209073 | 177209087 | 2.46e-05 | 0.12 | `AGGTCACTCAGGTTT` |
| 1 | chr5 | − | 106759320 | 106759334 | 2.46e-05 | 0.12 | `AGTACAGTAAGTTCA` |
| 1 | chr5 | + | 33886073 | 33886087 | 2.49e-05 | 0.121 | `AGGTGAAAGGGGACT` |
| 1 | chr10 | − | 7560127 | 7560141 | 2.49e-05 | 0.121 | `AGTTCAGCCAGTGCA` |
| 1 | chr6 | + | 26360198 | 26360212 | 2.51e-05 | 0.121 | `AGGGCACTAAGGCCG` |
| 1 | chr17 | − | 72615376 | 72615390 | 2.51e-05 | 0.121 | `AAGTCAAGGAGGGCA` |
| 1 | chr1 | + | 42045248 | 42045262 | 2.6e-05 | 0.125 | `GGGCCACAGGGTGCA` |
| 1 | chr3 | − | 186699627 | 186699641 | 2.61e-05 | 0.125 | `AGGACAGAGAGGGCG` |
| 1 | chr7 | − | 22621434 | 22621448 | 2.61e-05 | 0.125 | `GGTTCATTGGGTTCA` |
| 1 | chr17 | + | 35964139 | 35964153 | 2.62e-05 | 0.126 | `AGGCCAGAAGGTTCA` |
| 1 | chr10 | + | 126375517 | 126375531 | 2.64e-05 | 0.126 | `AGGCCAACGAGGCCT` |
| 1 | chr17 | − | 60549926 | 60549940 | 2.65e-05 | 0.127 | `AGTGCACTGAGTTTG` |
| 1 | chr20 | + | 23263053 | 23263067 | 2.67e-05 | 0.127 | `GGGTCAGGAAGTTTA` |
| 1 | chr10 | + | 112524139 | 112524153 | 2.7e-05 | 0.128 | `TAGTCACAGAGGCCA` |
| 1 | chr11 | + | 64945791 | 64945805 | 2.7e-05 | 0.128 | `AGTTCACCAGGTTTG` |
| 1 | chr6 | − | 74287302 | 74287316 | 2.72e-05 | 0.128 | `GGGTGGGAGAGTTCG` |
| 1 | chr18 | + | 54888804 | 54888818 | 2.72e-05 | 0.128 | `AGTTCATCGAGTTCC` |
| 1 | chr11 | + | 118068488 | 118068502 | 2.74e-05 | 0.129 | `GAGTCACCGAGGGCT` |
| 1 | chr1 | + | 111560404 | 111560418 | 2.75e-05 | 0.129 | `GGTTCCCCAAGGTCA` |
| 1 | chr14 | − | 91410025 | 91410039 | 2.78e-05 | 0.13 | `AGGTCATGGGGTTCA` |
| 1 | chr6 | − | 26231757 | 26231771 | 2.8e-05 | 0.13 | `AGGGCACCAAGGCCG` |
| 1 | chr6 | + | 27891141 | 27891155 | 2.8e-05 | 0.13 | `AGGGCACCAAGGCCG` |
| 1 | chr20 | + | 23262993 | 23263007 | 2.8e-05 | 0.13 | `AGGTCAGGAAGTTCC` |
| 1 | chr7 | − | 75515107 | 75515121 | 2.83e-05 | 0.131 | `AGGTGGAAGAGGACG` |
| 1 | chr5 | + | 56001680 | 56001694 | 2.85e-05 | 0.132 | `GGGCCAGTGGGTTCA` |
| 1 | chr1 | + | 36381738 | 36381752 | 2.88e-05 | 0.133 | `GGATCATAAAGGTCA` |
| 1 | chr11 | + | 64651025 | 64651039 | 2.93e-05 | 0.135 | `GAGTCACAGGGGCCA` |
| 1 | chr12 | − | 93480208 | 93480222 | 2.93e-05 | 0.135 | `GGTTCACAGAGCTTG` |
| 1 | chr19 | − | 48959627 | 48959641 | 2.93e-05 | 0.135 | `GGTCCAGAGAGGGCA` |
| 1 | chr16 | − | 55523409 | 55523423 | 2.95e-05 | 0.135 | `GGGTGGCAGAGGGCG` |
| 1 | chr2 | − | 231162657 | 231162671 | 2.98e-05 | 0.136 | `GGGTGAGTCAGTTCA` |
| 1 | chr17 | − | 60412903 | 60412917 | 2.98e-05 | 0.136 | `GGGTCCCTAAGGGCA` |
| 1 | chr2 | − | 191453390 | 191453404 | 3e-05 | 0.136 | `AGGTCAGCAGGTTTG` |
| 1 | chr8 | − | 103617754 | 103617768 | 3e-05 | 0.136 | `AGGTCAGCAGGTTTG` |
| 1 | chr5 | − | 149760829 | 149760843 | 3.05e-05 | 0.138 | `GGGTCAACAAGGCCC` |
| 1 | chr7 | − | 2901136 | 2901150 | 3.05e-05 | 0.138 | `GGGTCAACAAGGCCC` |
| 1 | chr2 | − | 12115060 | 12115074 | 3.07e-05 | 0.139 | `AAGTCAGAGGGGCCA` |
| 1 | chr19 | − | 17081977 | 17081991 | 3.1e-05 | 0.14 | `GGGTCAGGGGGTGCA` |
| 1 | chr3 | − | 53112140 | 53112154 | 3.16e-05 | 0.142 | `AGGTCATTAAGTTTG` |
| 1 | chr18 | − | 54888850 | 54888864 | 3.21e-05 | 0.144 | `AAGTGAGAGGGTTCA` |
| 1 | chr2 | − | 204403304 | 204403318 | 3.23e-05 | 0.145 | `AGGTTACTGAGTTCA` |
| 1 | chr4 | + | 100098404 | 100098418 | 3.25e-05 | 0.145 | `GGGTCAACAAGTACC` |
| 1 | chr3 | + | 73104978 | 73104992 | 3.27e-05 | 0.145 | `AGTTCAAAAGGTGTA` |
| 1 | chr6 | + | 167436621 | 167436635 | 3.27e-05 | 0.145 | `AGGTGAGTGGGTGCT` |
| 1 | chr7 | + | 30637112 | 30637126 | 3.27e-05 | 0.145 | `TGGTCACTGAGGTTC` |
| 1 | chr7 | + | 73269946 | 73269960 | 3.3e-05 | 0.146 | `AGGGCAGTGGGTTCG` |
| 1 | chr10 | − | 2980899 | 2980913 | 3.32e-05 | 0.146 | `AGTTCATGAAGTTCA` |
| 1 | chr17 | − | 75843101 | 75843115 | 3.32e-05 | 0.146 | `AGGTCAGAAACGACA` |
| 1 | chr22 | − | 39363536 | 39363550 | 3.32e-05 | 0.146 | `AGGTGCCCGGGTTCA` |
| 1 | chr7 | − | 47944705 | 47944719 | 3.34e-05 | 0.147 | `AGGTCACACAGGGCC` |
| 1 | chr1 | − | 210173180 | 210173194 | 3.36e-05 | 0.147 | `GGTTCAATGGGTGCT` |
| 1 | chr6 | + | 43005767 | 43005781 | 3.36e-05 | 0.147 | `GAGTCAAAGAGGGCT` |
| 1 | chr16 | − | 45510555 | 45510569 | 3.36e-05 | 0.147 | `AGGTCACACAGTGCC` |
| 1 | chr1 | + | 210172905 | 210172919 | 3.41e-05 | 0.148 | `AGGGCCAAGAGGTCA` |
| 1 | chr9 | − | 125141632 | 125141646 | 3.41e-05 | 0.148 | `AAGTGACCGAGGCCG` |
| 1 | chr6 | + | 106696670 | 106696684 | 3.45e-05 | 0.15 | `AGGTGACAAGGGCCT` |
| 1 | chr6 | + | 64120759 | 64120773 | 3.47e-05 | 0.15 | `AGTTCACAGAAGTTG` |
| 1 | chr1 | + | 154462970 | 154462984 | 3.49e-05 | 0.15 | `AGGGCAGTGAGGACT` |
| 1 | chr2 | + | 156082340 | 156082354 | 3.49e-05 | 0.15 | `AGGGCAGCAGGTTCA` |
| 1 | chr5 | − | 149771457 | 149771471 | 3.49e-05 | 0.15 | `GGTTGACGGGGGTCA` |
| 1 | chr10 | − | 90165761 | 90165775 | 3.49e-05 | 0.15 | `GGGTCATGAAGGTCA` |
| 1 | chr13 | − | 108612853 | 108612867 | 3.49e-05 | 0.15 | `AGGTCCATGAGGTTG` |
| 1 | chr13 | + | 97939251 | 97939265 | 3.51e-05 | 0.15 | `GGGTCACAGACTTCC` |
| 1 | chr16 | − | 55523240 | 55523254 | 3.51e-05 | 0.15 | `GGGTGGCTGAGGGCG` |
| 1 | chr10 | − | 82002356 | 82002370 | 3.53e-05 | 0.15 | `AGGTGGATGAGGTCC` |
| 1 | chr10 | − | 82002563 | 82002577 | 3.53e-05 | 0.15 | `AGGTGGATGAGGTCC` |
| 1 | chr10 | − | 82002635 | 82002649 | 3.53e-05 | 0.15 | `AGGTGGATGAGGTCC` |
| 1 | chr10 | − | 82002743 | 82002757 | 3.53e-05 | 0.15 | `AGGTGGATGAGGTCC` |
| 1 | chr11 | + | 65098704 | 65098718 | 3.55e-05 | 0.15 | `GGGTGGCAGAGGCCG` |
| 1 | chr1 | − | 232898835 | 232898849 | 3.57e-05 | 0.15 | `AGTTGAAAGAGGGTT` |
| 1 | chr2 | − | 231446084 | 231446098 | 3.57e-05 | 0.15 | `GGTTCAGCAGGTGCA` |
| 1 | chr19 | + | 17366834 | 17366848 | 3.57e-05 | 0.15 | `GGTTGGCAGAGTTTA` |
| 1 | chr8 | − | 125770157 | 125770171 | 3.59e-05 | 0.15 | `TGGTGAGGGAGTTCA` |
| 1 | chr17 | − | 57526135 | 57526149 | 3.59e-05 | 0.15 | `AAGGCAGCGAGTTCA` |
| 1 | chr6 | + | 31239875 | 31239889 | 3.65e-05 | 0.152 | `TGGTGACTGAGTCTA` |
| 1 | chr14 | + | 96437438 | 96437452 | 3.65e-05 | 0.152 | `AGTTCATACAGTTCA` |
| 1 | chr10 | − | 82002428 | 82002442 | 3.67e-05 | 0.152 | `AGGTGATTGAGGTCC` |
| 1 | chr14 | − | 72013250 | 72013264 | 3.67e-05 | 0.152 | `GGTTGGCTGAGTACA` |
| 1 | chr16 | − | 3161565 | 3161579 | 3.67e-05 | 0.152 | `AGTTCCAGGAGGTCA` |
| 1 | chr17 | + | 73629179 | 73629193 | 3.69e-05 | 0.153 | `GAGTCACTGGGGACA` |
| 1 | chr15 | − | 42808635 | 42808649 | 3.72e-05 | 0.154 | `AGGCCAGGGAGGGCA` |
| 1 | chr3 | + | 151298151 | 151298165 | 3.74e-05 | 0.154 | `AGATGAGTAAGGTTA` |
| 1 | chr21 | + | 41739521 | 41739535 | 3.74e-05 | 0.154 | `AGGGCAGGGAGTGCA` |
| 1 | chr19 | − | 2561822 | 2561836 | 3.78e-05 | 0.155 | `GGGTCCCTGAGGCCG` |
| 1 | chr8 | + | 97316968 | 97316982 | 3.8e-05 | 0.156 | `AAGTCATAGAGGACA` |
| 1 | chr22 | − | 40195138 | 40195152 | 3.82e-05 | 0.156 | `AGGTCCCTGAGCTCG` |
| 1 | chr22 | + | 23160301 | 23160315 | 3.84e-05 | 0.157 | `AAGTCACCAAGGACT` |
| 1 | chr1 | − | 23755872 | 23755886 | 3.86e-05 | 0.157 | `GGGTGAACAGGGGCA` |
| 1 | chr5 | − | 32212309 | 32212323 | 3.86e-05 | 0.157 | `GGGTGAACAGGGGCA` |
| 1 | chr3 | + | 143427191 | 143427205 | 3.88e-05 | 0.157 | `AGGTGAGCGGGTTTG` |
| 1 | chr16 | + | 18720514 | 18720528 | 3.88e-05 | 0.157 | `AGGTCAAAGGAGACA` |
| 1 | chr19 | − | 44592718 | 44592732 | 3.88e-05 | 0.157 | `GGGTCGCAGGGGGCG` |
| 1 | chr20 | − | 31934919 | 31934933 | 3.91e-05 | 0.158 | `AGGTCACCAGGGCTG` |
| 1 | chr1 | + | 2481098 | 2481112 | 3.99e-05 | 0.161 | `GGGTGACAGAGCTCC` |
| 1 | chr20 | − | 51632166 | 51632180 | 3.99e-05 | 0.161 | `CGATCAACGAGGTCG` |
| 1 | chr18 | − | 17966721 | 17966735 | 4.01e-05 | 0.161 | `AGTTCACCCAGTGTA` |
| 1 | chr5 | − | 6536968 | 6536982 | 4.04e-05 | 0.162 | `AGGGCATAGAGGACA` |
| 1 | chr3 | − | 187990941 | 187990955 | 4.06e-05 | 0.162 | `AGTTGGCAGAGTGTA` |
| 1 | chr15 | + | 55210595 | 55210609 | 4.08e-05 | 0.163 | `GGGGCAAAAGGTTCA` |
| 1 | chr18 | − | 19187583 | 19187597 | 4.15e-05 | 0.165 | `AGGTCATTAGGGCCA` |
| 1 | chr12 | − | 109584183 | 109584197 | 4.17e-05 | 0.166 | `AGTGGAGAGAGGGCA` |
| 1 | chr22 | − | 36334674 | 36334688 | 4.17e-05 | 0.166 | `AGGCCAACGAGCTCG` |
| 1 | chr3 | + | 13100750 | 13100764 | 4.2e-05 | 0.166 | `AGGTCACCCAGCCCA` |
| 1 | chr12 | − | 46550475 | 46550489 | 4.2e-05 | 0.166 | `GGATGACAGGGTCCA` |
| 1 | chr1 | − | 110751825 | 110751839 | 4.22e-05 | 0.167 | `AAGTGACTGAGGCCT` |
| 1 | chr14 | − | 92240487 | 92240501 | 4.24e-05 | 0.167 | `AGTTGACACGGTTCA` |
| 1 | chr17 | − | 57526126 | 57526140 | 4.24e-05 | 0.167 | `AGTTCACAGAGCATG` |
| 1 | chr6 | − | 131991382 | 131991396 | 4.26e-05 | 0.167 | `AGGTCCAGAAGGTCA` |
| 1 | chr10 | − | 121239186 | 121239200 | 4.26e-05 | 0.167 | `AGTTCAGGCAGTTCA` |
| 1 | chr2 | + | 232287860 | 232287874 | 4.29e-05 | 0.168 | `AGGCCACACAGGGCA` |
| 1 | chr3 | + | 109112638 | 109112652 | 4.36e-05 | 0.17 | `GGGTCATGGAGTCCA` |
| 1 | chr11 | − | 65384122 | 65384136 | 4.36e-05 | 0.17 | `AGGGCAAAGAGTGTG` |
| 1 | chr7 | + | 135301025 | 135301039 | 4.38e-05 | 0.17 | `AGTTCATTGGGTTTA` |
| 1 | chr1 | + | 27824123 | 27824137 | 4.4e-05 | 0.171 | `GGTTCAGGAAGGCCA` |
| 1 | chr3 | − | 13108026 | 13108040 | 4.45e-05 | 0.172 | `AGGGCAGGGAGGCCA` |
| 1 | chr16 | − | 30490799 | 30490813 | 4.45e-05 | 0.172 | `AGGCCAGAGAAGTCA` |
| 1 | chr17 | − | 38793863 | 38793877 | 4.45e-05 | 0.172 | `GGATCACTGGGGTCC` |
| 1 | chr6 | + | 44330086 | 44330100 | 4.47e-05 | 0.172 | `AGGTGGCAGAGCACA` |
| 1 | chr13 | − | 98870838 | 98870852 | 4.52e-05 | 0.174 | `GGGTGAGTGAGTGTG` |
| 1 | chr1 | − | 148818363 | 148818377 | 4.55e-05 | 0.175 | `AGGTCCCCGACGTCA` |
| 1 | chr13 | − | 101836987 | 101837001 | 4.57e-05 | 0.175 | `GGGTCATTCAGTTCA` |
| 1 | chr1 | + | 165898887 | 165898901 | 4.59e-05 | 0.175 | `AGGTCATCAGGGCCA` |
| 1 | chr3 | + | 113662395 | 113662409 | 4.59e-05 | 0.175 | `GGTTCATAAAGGTTA` |
| 1 | chr16 | + | 11743042 | 11743056 | 4.59e-05 | 0.175 | `GGAGCAAAAAGTTCA` |
| 1 | chr22 | + | 40194954 | 40194968 | 4.59e-05 | 0.175 | `CGTTCACAGGGTTCT` |
| 1 | chr5 | − | 98391368 | 98391382 | 4.62e-05 | 0.176 | `AGGTCAATGGATACA` |
| 1 | chr2 | + | 9755806 | 9755820 | 4.69e-05 | 0.178 | `GGGGGAAAGAGTTTA` |
| 1 | chr12 | + | 37455714 | 37455728 | 4.69e-05 | 0.178 | `GAGTCAATGGGTGCA` |
| 1 | chr11 | − | 64656493 | 64656507 | 4.71e-05 | 0.178 | `AGATCGCTGGGGCCA` |
| 1 | chr12 | + | 15832706 | 15832720 | 4.71e-05 | 0.178 | `AGGGCAGGGAGTACA` |
| 1 | chr1 | + | 111548710 | 111548724 | 4.74e-05 | 0.178 | `GGGTGGAAGAGGGCG` |
| 1 | chr17 | + | 71989156 | 71989170 | 4.74e-05 | 0.178 | `TGGACACAGAGGACA` |
| 1 | chr20 | − | 57013442 | 57013456 | 4.74e-05 | 0.178 | `AGGTCAACAGGTGCC` |
| 1 | chr6 | + | 143170181 | 143170195 | 4.77e-05 | 0.178 | `AGTTCATCCAGGTCA` |
| 1 | chr1 | + | 2480928 | 2480942 | 4.79e-05 | 0.178 | `GGGTGGCTGAGGGCT` |
| 1 | chr2 | + | 179103533 | 179103547 | 4.79e-05 | 0.178 | `GATTCAGTGAGGTTA` |
| 1 | chr3 | + | 72296454 | 72296468 | 4.79e-05 | 0.178 | `AGGACAGAGAGGATA` |
| 1 | chr10 | + | 85889312 | 85889326 | 4.79e-05 | 0.178 | `AGGTGACCGGGGACC` |
| 1 | chr9 | − | 89269518 | 89269532 | 4.82e-05 | 0.179 | `AGTTCACAGGAGACA` |
| 1 | chr3 | − | 197308089 | 197308103 | 4.85e-05 | 0.179 | `AGTTCACCAGAGTCA` |
| 1 | chr10 | − | 103805932 | 103805946 | 4.85e-05 | 0.179 | `GGGTCGGGGAGGTCG` |
| 1 | chr13 | − | 108612862 | 108612876 | 4.85e-05 | 0.179 | `AGGTGGGTGAGGTCC` |
| 1 | chr8 | − | 96313027 | 96313041 | 4.88e-05 | 0.18 | `AGGAGAGTGAGTACA` |
| 1 | chr17 | − | 78000931 | 78000945 | 4.91e-05 | 0.181 | `AGGTGGAGGAGGGCA` |
| 1 | chr16 | + | 66464591 | 66464605 | 4.98e-05 | 0.183 | `GGGTGAGCGAGGGTG` |
| 1 | chr19 | − | 33312917 | 33312931 | 5.01e-05 | 0.184 | `GGTTGAGACAGGTCA` |
| 1 | chr19 | − | 47141353 | 47141367 | 5.01e-05 | 0.184 | `GGGTCACTGAGCCTG` |
| 1 | chr15 | + | 37877236 | 37877250 | 5.04e-05 | 0.184 | `CGGTCATTGAGTGCA` |
| 1 | chr21 | − | 25880576 | 25880590 | 5.04e-05 | 0.184 | `AGGTCCTAAAGTTCA` |
| 1 | chr6 | − | 150011528 | 150011542 | 5.07e-05 | 0.184 | `GAGTCGCCGAGGTCG` |
| 1 | chr12 | + | 97421827 | 97421841 | 5.07e-05 | 0.184 | `AGGGGAGAGAGGGCG` |
| 1 | chr19 | + | 33312899 | 33312913 | 5.07e-05 | 0.184 | `GGGTCATAAAGGTCC` |
| 1 | chr16 | + | 30377555 | 30377569 | 5.1e-05 | 0.184 | `AGATCACAAGGGCCT` |
| 1 | chr19 | − | 53809596 | 53809610 | 5.1e-05 | 0.184 | `GGGTCCCCGAGGTTG` |
| 1 | chr22 | + | 21608152 | 21608166 | 5.12e-05 | 0.185 | `GGGTCCCAGGGTCCA` |
| 1 | chr7 | + | 139114114 | 139114128 | 5.15e-05 | 0.186 | `AGGGCAACAAGGGCT` |
| 1 | chr1 | − | 158860121 | 158860135 | 5.17e-05 | 0.186 | `AAGTCACCAGGGTTA` |
| 1 | chr7 | − | 139263162 | 139263176 | 5.17e-05 | 0.186 | `AGTTCAAGGGGGGCG` |
| 1 | chr11 | + | 64650757 | 64650771 | 5.17e-05 | 0.186 | `GGGGCACAGAGTGCC` |
| 1 | chr9 | − | 115384007 | 115384021 | 5.22e-05 | 0.187 | `AGGTCGGGAAGGGCA` |
| 1 | chr12 | − | 111997847 | 111997861 | 5.22e-05 | 0.187 | `AGGGCACTGAGGCTT` |
| 1 | chr19 | + | 51914391 | 51914405 | 5.28e-05 | 0.188 | `GGGACAGAAAGGGCA` |
| 1 | chr19 | − | 55071970 | 55071984 | 5.28e-05 | 0.188 | `GGGTCAGGGACGTCA` |
| 1 | chr5 | + | 131786534 | 131786548 | 5.31e-05 | 0.188 | `GGGTGAAAGGGGACT` |
| 1 | chr7 | + | 114344983 | 114344997 | 5.31e-05 | 0.188 | `AGGTCATTGGGTTTG` |
| 1 | chr12 | + | 123119 | 123133 | 5.31e-05 | 0.188 | `GGGTGCCAGGGTTCA` |
| 1 | chr12 | + | 123149 | 123163 | 5.31e-05 | 0.188 | `GGGTGCCAGGGTTCA` |
| 1 | chr12 | − | 55014468 | 55014482 | 5.31e-05 | 0.188 | `GGGGCAACAGGGTCA` |
| 1 | chr19 | − | 2036138 | 2036152 | 5.31e-05 | 0.188 | `GGGACAACGAGGGCG` |
| 1 | chr4 | − | 8281445 | 8281459 | 5.37e-05 | 0.189 | `AGGGCAGAGAGGGCC` |
| 1 | chr7 | + | 4648153 | 4648167 | 5.4e-05 | 0.19 | `AGGTCAGCGGGCGCG` |
| 1 | chr19 | − | 55990296 | 55990310 | 5.43e-05 | 0.191 | `AAGTCACAGAAGCCA` |
| 1 | chr6 | − | 16612433 | 16612447 | 5.45e-05 | 0.191 | `GAGTCAGTGAGGGCT` |
| 1 | chr16 | − | 51685587 | 51685601 | 5.45e-05 | 0.191 | `TGGTCACCAAGCCCA` |
| 1 | chr17 | − | 63846555 | 63846569 | 5.45e-05 | 0.191 | `AGTCCAAGGAGGTCG` |
| 1 | chr8 | − | 126683551 | 126683565 | 5.48e-05 | 0.191 | `GGGCCACAAAGGTTG` |
| 1 | chr17 | − | 52790365 | 52790379 | 5.48e-05 | 0.191 | `GGTTCACTGAGTATC` |
| 1 | chr8 | + | 67507518 | 67507532 | 5.51e-05 | 0.191 | `GGAACAGCGAGGTCA` |
| 1 | chr19 | − | 10900215 | 10900229 | 5.51e-05 | 0.191 | `GGGACGACGAGTTCA` |
| 1 | chr9 | − | 111946257 | 111946271 | 5.57e-05 | 0.193 | `GGAGCAATGAGGGCA` |
| 1 | chr2 | + | 179103806 | 179103820 | 5.6e-05 | 0.193 | `GGGTGAGAAGGTTCT` |
| 1 | chr3 | + | 198153949 | 198153963 | 5.6e-05 | 0.193 | `AGGGCGGCGAGGTCG` |
| 1 | chr5 | + | 133996518 | 133996532 | 5.63e-05 | 0.194 | `AGTTGAGCGAGGCTG` |
| 1 | chr15 | − | 42791151 | 42791165 | 5.66e-05 | 0.195 | `GGGCCACAGAGGGTG` |
| 1 | chr8 | − | 110415637 | 110415651 | 5.72e-05 | 0.197 | `GGGTAACAGAGTTCA` |
| 1 | chr13 | − | 96725057 | 96725071 | 5.75e-05 | 0.197 | `TAGTCAGTAAGTTCA` |
| 1 | chr9 | + | 113767710 | 113767724 | 5.78e-05 | 0.198 | `AGGTCACTCAGTTTC` |
| 1 | chr21 | − | 44388394 | 44388408 | 5.81e-05 | 0.198 | `AGGGGACCGGGTCCA` |
| 1 | chr14 | − | 80991002 | 80991016 | 5.84e-05 | 0.198 | `GGGTCATGGGGGTCA` |
| 1 | chr19 | − | 47141447 | 47141461 | 5.84e-05 | 0.198 | `CGGTGACCGAGGTTG` |
| 1 | chr3 | + | 4994071 | 4994085 | 5.87e-05 | 0.198 | `AGGTCGTCAAGGGCA` |
| 1 | chr4 | − | 25694742 | 25694756 | 5.87e-05 | 0.198 | `TGGTCGCAGAGTCCT` |
| 1 | chr22 | + | 38246947 | 38246961 | 5.87e-05 | 0.198 | `GGGTCCCAGGGGTCT` |
| 1 | chr4 | − | 40545338 | 40545352 | 5.9e-05 | 0.198 | `AGTCCAATGGGGACA` |
| 1 | chr6 | − | 31430864 | 31430878 | 5.9e-05 | 0.198 | `AGGAGGAAGAGTTCA` |
| 1 | chr6 | − | 31430864 | 31430878 | 5.9e-05 | 0.198 | `AGGAGGAAGAGTTCA` |
| 1 | chr9 | + | 116483935 | 116483949 | 5.9e-05 | 0.198 | `GGGTGGGGGAGGTCA` |
| 1 | chr9 | − | 121838724 | 121838738 | 5.9e-05 | 0.198 | `AGTGCAATGGGGACA` |
| 1 | chr20 | + | 49437149 | 49437163 | 5.9e-05 | 0.198 | `GGGTGAGTGAGGTTC` |
| 1 | chr20 | − | 51702459 | 51702473 | 5.9e-05 | 0.198 | `AGACCAGTGGGGTCA` |
| 1 | chr2 | − | 54660633 | 54660647 | 6e-05 | 0.201 | `AGGTCATACAGGTCT` |
| 1 | chr17 | − | 78001080 | 78001094 | 6e-05 | 0.201 | `GGGTCCCTGGGGCCA` |
| 1 | chr20 | + | 5689580 | 5689594 | 6.03e-05 | 0.201 | `GGGTGGTTGAGTTCA` |
| 1 | chr11 | − | 62329662 | 62329676 | 6.09e-05 | 0.203 | `GGTTCCGCAAGTTCA` |
| 1 | chr4 | + | 186018387 | 186018401 | 6.12e-05 | 0.204 | `AGGCCACTGGGGACT` |
| 1 | chr19 | − | 51914569 | 51914583 | 6.12e-05 | 0.204 | `AGGGCACTGGGGACT` |
| 1 | chr8 | + | 28690995 | 28691009 | 6.15e-05 | 0.204 | `GGGTGAGAGGGTTTG` |
| 1 | chr12 | − | 74640210 | 74640224 | 6.15e-05 | 0.204 | `AGGTCAGAGAGTTAA` |
| 1 | chr14 | + | 34942667 | 34942681 | 6.15e-05 | 0.204 | `AGTTGAGGAAGGCCA` |
| 1 | chr3 | − | 120781293 | 120781307 | 6.18e-05 | 0.204 | `GGGTGGACGAGGGCG` |
| 1 | chr6 | − | 107887395 | 107887409 | 6.18e-05 | 0.204 | `GGGTGGCCGGGGGCA` |
| 1 | chrX | + | 138635646 | 138635660 | 6.18e-05 | 0.204 | `AGGACAGTGAGGTTT` |
| 1 | chr12 | + | 52132102 | 52132116 | 6.18e-05 | 0.204 | `GGGTCGAAGGGTGCG` |
| 1 | chr7 | + | 5701197 | 5701211 | 6.22e-05 | 0.204 | `GGGCCAATGGGGACA` |
| 1 | chr2 | − | 33557923 | 33557937 | 6.25e-05 | 0.205 | `AGGCCAGAAGGGGCA` |
| 1 | chr6 | + | 135685756 | 135685770 | 6.28e-05 | 0.205 | `GGGTCCCCGAGGATA` |
| 1 | chr6 | + | 135685756 | 135685770 | 6.28e-05 | 0.205 | `GGGTCCCCGAGGATA` |
| 1 | chr12 | + | 120951740 | 120951754 | 6.32e-05 | 0.205 | `TGGGCAGAGAGGCCA` |
| 1 | chr14 | − | 58712089 | 58712103 | 6.32e-05 | 0.205 | `AGTGCATAGAGTGCA` |
| 1 | chr16 | + | 55523138 | 55523152 | 6.32e-05 | 0.205 | `GGGTGAAAAAGGGTG` |
| 1 | chr19 | + | 4293690 | 4293704 | 6.32e-05 | 0.205 | `AGGGGACAAAGGCCG` |
| 1 | chr6 | + | 91240068 | 91240082 | 6.35e-05 | 0.206 | `AGATGACAGAAGGCA` |
| 1 | chr9 | + | 114698278 | 114698292 | 6.38e-05 | 0.207 | `AAGTCACTGAAGCCA` |
| 1 | chr10 | − | 45236547 | 45236561 | 6.41e-05 | 0.207 | `AAGCCAGAGAGGGCA` |
| 1 | chr16 | + | 20793691 | 20793705 | 6.41e-05 | 0.207 | `AGGCCCCAGAGTACA` |
| 1 | chr1 | − | 1700472 | 1700486 | 6.44e-05 | 0.208 | `AGGCCACCGGGTCCT` |
| 1 | chr6 | + | 88670529 | 88670543 | 6.55e-05 | 0.211 | `AGGTCACCAAATATA` |
| 1 | chr12 | − | 127881178 | 127881192 | 6.55e-05 | 0.211 | `AGGTGGAAAAGTACG` |
| 1 | chr15 | + | 57492381 | 57492395 | 6.55e-05 | 0.211 | `GGTTGAGAAAGGTCC` |
| 1 | chr1 | + | 36381756 | 36381770 | 6.62e-05 | 0.211 | `AGGTCAACCAGCCCA` |
| 1 | chr2 | − | 239891078 | 239891092 | 6.62e-05 | 0.211 | `AGTTCAGCAGGGCCT` |
| 1 | chr17 | − | 30723992 | 30724006 | 6.62e-05 | 0.211 | `AGGACAAAGAGCTCT` |
| 1 | chr10 | + | 26816688 | 26816702 | 6.65e-05 | 0.211 | `GGTTCAGTGGGTTTT` |
| 1 | chr17 | + | 3561024 | 3561038 | 6.65e-05 | 0.211 | `AGTTCAGCGGGTCCC` |
| 1 | chr3 | + | 39431068 | 39431082 | 6.68e-05 | 0.211 | `AGTTCAAACAGTTTT` |
| 1 | chr5 | + | 138748538 | 138748552 | 6.68e-05 | 0.211 | `AGGGGGCTGAGTGCA` |
| 1 | chr6 | − | 344685 | 344699 | 6.68e-05 | 0.211 | `AGATGCCAGAGGGCA` |
| 1 | chr7 | + | 4689662 | 4689676 | 6.68e-05 | 0.211 | `GGGTTACTGAGGTCA` |
| 1 | chr11 | + | 73644698 | 73644712 | 6.68e-05 | 0.211 | `GGTTCATCAGGTTCA` |
| 1 | chr5 | − | 172395127 | 172395141 | 6.72e-05 | 0.212 | `AGTTCAGACAGTTTG` |
| 1 | chr15 | − | 66048557 | 66048571 | 6.72e-05 | 0.212 | `AGGTCATGGGGTTCG` |
| 1 | chr17\_random | + | 85911 | 85925 | 6.75e-05 | 0.213 | `AGGAGAAAGAGGCCG` |
| 1 | chr3 | − | 198153681 | 198153695 | 6.79e-05 | 0.213 | `GGGTGAGGGAGGCCG` |
| 1 | chr20 | + | 23263210 | 23263224 | 6.79e-05 | 0.213 | `AGGTCAGGGAATCCA` |
| 1 | chr20 | + | 62053452 | 62053466 | 6.79e-05 | 0.213 | `AGAGCACAGGGGACA` |
| 1 | chr22 | − | 46872396 | 46872410 | 6.79e-05 | 0.213 | `GGGGCAGAGGGGCCA` |
| 1 | chr16 | − | 28765020 | 28765034 | 6.87e-05 | 0.215 | `TGGTCAGGGAGGGCG` |
| 1 | chr1 | + | 27824206 | 27824220 | 6.93e-05 | 0.216 | `GGGTCAGTGGGGCTG` |
| 1 | chr12 | − | 121914753 | 121914767 | 6.93e-05 | 0.216 | `AGGTCCAAGAGGGTG` |
| 1 | chr8 | + | 98712790 | 98712804 | 6.96e-05 | 0.216 | `TATTCAAAAAGTTCA` |
| 1 | chr10 | − | 11368642 | 11368656 | 6.96e-05 | 0.216 | `GGGTCACACAGTGCC` |
| 1 | chr12 | − | 112095642 | 112095656 | 6.96e-05 | 0.216 | `GGTTCACTGGGTTTC` |
| 1 | chr8 | + | 105667295 | 105667309 | 7e-05 | 0.217 | `AGGAGACAGAGGTTT` |
| 1 | chr18 | − | 12410368 | 12410382 | 7e-05 | 0.217 | `GGTTCATCGGGTTCG` |
| 1 | chr10 | + | 81946405 | 81946419 | 7.03e-05 | 0.217 | `AAGTCACCGAAGCCA` |
| 1 | chr19 | − | 44591447 | 44591461 | 7.07e-05 | 0.218 | `GGGGGAGAGGGTTCA` |
| 1 | chr5 | + | 43639643 | 43639657 | 7.11e-05 | 0.219 | `AGTTCGGAGAGGGTG` |
| 1 | chr6 | − | 355157 | 355171 | 7.14e-05 | 0.22 | `TGTCCACTGAGTACA` |
| 1 | chr19 | − | 47141506 | 47141520 | 7.14e-05 | 0.22 | `GGGTGAGTCAGTTCG` |
| 1 | chr1 | − | 171646647 | 171646661 | 7.22e-05 | 0.22 | `AGGACACTGGGTTCC` |
| 1 | chr20 | + | 47339075 | 47339089 | 7.22e-05 | 0.22 | `AGTTCCACAAGGTTA` |
| 1 | chr22 | + | 49310713 | 49310727 | 7.22e-05 | 0.22 | `AGTCCAGCGAGTCCT` |
| 1 | chr1 | − | 8380179 | 8380193 | 7.25e-05 | 0.22 | `GGATCAGAAAGGTTT` |
| 1 | chr3 | + | 4994271 | 4994285 | 7.25e-05 | 0.22 | `GGAGCGCAGAGGTCA` |
| 1 | chr4 | − | 185973867 | 185973881 | 7.25e-05 | 0.22 | `TGGGCAGAGAGGTCT` |
| 1 | chr5 | + | 56001651 | 56001665 | 7.25e-05 | 0.22 | `AAATCAGAGAGCTCA` |
| 1 | chr10 | − | 97582147 | 97582161 | 7.25e-05 | 0.22 | `AGTTCATTAAGGCTA` |
| 1 | chr16 | − | 79267826 | 79267840 | 7.25e-05 | 0.22 | `AGGTCAGGAAGGGTG` |
| 1 | chr9 | + | 115383943 | 115383957 | 7.33e-05 | 0.222 | `AGGGCAAGAAGGCCA` |
| 1 | chr16 | − | 11330366 | 11330380 | 7.33e-05 | 0.222 | `AGGGCAGTAGGGGCA` |
| 1 | chr2 | − | 169060577 | 169060591 | 7.36e-05 | 0.222 | `AGGTGCACAAGGGCA` |
| 1 | chr2 | − | 231558505 | 231558519 | 7.36e-05 | 0.222 | `AGGTGGGAAAGCTCA` |
| 1 | chr6 | + | 90127166 | 90127180 | 7.36e-05 | 0.222 | `TGGCCAATGGGTTCA` |
| 1 | chr10 | + | 104144020 | 104144034 | 7.36e-05 | 0.222 | `GGGTCAGCGGGTACC` |
| 1 | chr19 | + | 10091720 | 10091734 | 7.4e-05 | 0.222 | `GGGCCAGCGGGGGCA` |
| 1 | chr6 | + | 134610426 | 134610440 | 7.44e-05 | 0.223 | `TGGTGGCTGAGGTTA` |
| 1 | chr8 | − | 146075110 | 146075124 | 7.48e-05 | 0.224 | `AGATCACTGGGTGTT` |
| 1 | chr9 | − | 86499360 | 86499374 | 7.48e-05 | 0.224 | `TGGTCCCTGGGTTCA` |
| 1 | chr17 | + | 73652495 | 73652509 | 7.52e-05 | 0.225 | `TGGTCAGCAGGGCCA` |
| 1 | chr1 | + | 146273159 | 146273173 | 7.56e-05 | 0.225 | `AGAGCACTGGGGCCA` |
| 1 | chr4 | − | 160317803 | 160317817 | 7.56e-05 | 0.225 | `AGTTGAATGAATTCT` |
| 1 | chr8 | − | 96219162 | 96219176 | 7.56e-05 | 0.225 | `GGGTCACCGGAGTTA` |
| 1 | chr3 | − | 109327558 | 109327572 | 7.6e-05 | 0.226 | `AGACCACTGGGTCCA` |
| 1 | chr1 | − | 28374432 | 28374446 | 7.63e-05 | 0.226 | `AGATGACTGGGTCCT` |
| 1 | chr1 | − | 84744987 | 84745001 | 7.63e-05 | 0.226 | `GGGTCAGCGGGGCTG` |
| 1 | chr6 | − | 30933508 | 30933522 | 7.63e-05 | 0.226 | `GGAGCAGTGAGGTCG` |
| 1 | chr8 | + | 126683410 | 126683424 | 7.67e-05 | 0.226 | `TGGGGAGTGAGGTCA` |
| 1 | chr17 | − | 46585668 | 46585682 | 7.67e-05 | 0.226 | `AGTCCAAAGAGTGCC` |
| 1 | chr3 | + | 58002896 | 58002910 | 7.71e-05 | 0.227 | `GGTTGAACAAGTCCT` |
| 1 | chr12 | + | 6425317 | 6425331 | 7.71e-05 | 0.227 | `TGTTCCCAGAGTGCA` |
| 1 | chr16 | − | 11253414 | 11253428 | 7.71e-05 | 0.227 | `GGGTGAGCCAGGGCA` |
| 1 | chr11 | − | 64656536 | 64656550 | 7.75e-05 | 0.227 | `AGTTCCCAGAGGGCC` |
| 1 | chr19 | + | 13139491 | 13139505 | 7.75e-05 | 0.227 | `AGGCCAGAGGGGCCG` |
| 1 | chr7 | + | 5564268 | 5564282 | 7.79e-05 | 0.227 | `TGGGCAGTGAGGACA` |
| 1 | chr17 | − | 3811719 | 3811733 | 7.79e-05 | 0.227 | `AGGGCAGAAGGGACA` |
| 1 | chr17 | − | 59274062 | 59274076 | 7.79e-05 | 0.227 | `GGGGCAGGGAGTTCG` |
| 1 | chr5 | + | 106935030 | 106935044 | 7.83e-05 | 0.227 | `CAGTCAACAAGTTCA` |
| 1 | chr10 | − | 112145642 | 112145656 | 7.83e-05 | 0.227 | `GATACACAGAGGTCA` |
| 1 | chr11 | + | 117387036 | 117387050 | 7.83e-05 | 0.227 | `AGGGGACAGAGGGTG` |
| 1 | chr14 | − | 93499257 | 93499271 | 7.83e-05 | 0.227 | `AGTTGACAGGGGATG` |
| 1 | chr16 | + | 11363591 | 11363605 | 7.87e-05 | 0.228 | `AGATGCCTGAGTGCA` |
| 1 | chr1 | − | 94085979 | 94085993 | 7.91e-05 | 0.229 | `GGGCCACCGAGGCCC` |
| 1 | chr1 | − | 201541605 | 201541619 | 7.95e-05 | 0.23 | `GGGCCACCGAGTCCC` |
| 1 | chr11 | − | 85336738 | 85336752 | 7.95e-05 | 0.23 | `AGGTCAGCGAGTTGA` |
| 1 | chr2 | + | 46310315 | 46310329 | 7.99e-05 | 0.23 | `GGTTCGTAGAGTGCA` |
| 1 | chr22 | + | 49314178 | 49314192 | 7.99e-05 | 0.23 | `AGGGGAAAGGGGTCT` |
| 1 | chr1 | + | 112040650 | 112040664 | 8.03e-05 | 0.23 | `GGGTGGATGAGTTTG` |
| 1 | chr16 | + | 10967022 | 10967036 | 8.03e-05 | 0.23 | `AAGTGACAGAGTCCC` |
| 1 | chr20 | + | 62053696 | 62053710 | 8.07e-05 | 0.23 | `GGGGCAGACAGGTCA` |
| 1 | chr2 | + | 239890856 | 239890870 | 8.11e-05 | 0.23 | `AGGACACAAAGCCCA` |
| 1 | chr7 | − | 21972615 | 21972629 | 8.11e-05 | 0.23 | `AGGTGACAGAGGTAA` |
| 1 | chr7 | − | 22357563 | 22357577 | 8.11e-05 | 0.23 | `AGGGCAAAGGGTCTA` |
| 1 | chr7 | + | 101420896 | 101420910 | 8.11e-05 | 0.23 | `AGGTCCCTGGGGGTA` |
| 1 | chr11 | + | 65076379 | 65076393 | 8.11e-05 | 0.23 | `TGGTCAGTGGGTGCT` |
| 1 | chr11 | + | 65076379 | 65076393 | 8.11e-05 | 0.23 | `TGGTCAGTGGGTGCT` |
| 1 | chr11 | + | 85603463 | 85603477 | 8.11e-05 | 0.23 | `AGGGCAGCAGGTGCA` |
| 1 | chr11 | + | 85835391 | 85835405 | 8.11e-05 | 0.23 | `AGGTGATGGGGTTCA` |
| 1 | chrX | + | 7043740 | 7043754 | 8.15e-05 | 0.23 | `AGGTCACTGTGGGCA` |
| 1 | chr12 | + | 109584126 | 109584140 | 8.15e-05 | 0.23 | `AGTTCAGCCAGTGCT` |
| 1 | chr12 | − | 122134394 | 122134408 | 8.15e-05 | 0.23 | `GGGTGAAGGAGTTTT` |
| 1 | chr10 | + | 70495688 | 70495702 | 8.19e-05 | 0.231 | `TGGTCAGTAGGGTTA` |
| 1 | chr22 | + | 37208279 | 37208293 | 8.23e-05 | 0.232 | `GGATCAGTAGGTCCA` |
| 1 | chr4 | + | 154628818 | 154628832 | 8.28e-05 | 0.232 | `GGGTGCGTGAGTGCA` |
| 1 | chr12 | + | 93480231 | 93480245 | 8.28e-05 | 0.232 | `AGAAGACCGAGTGCA` |
| 1 | chr18 | − | 47227669 | 47227683 | 8.28e-05 | 0.232 | `AGGTGCCTGGGGCCA` |
| 1 | chr12 | + | 107535426 | 107535440 | 8.32e-05 | 0.233 | `AGGTCAGCAGGGCTG` |
| 1 | chr20 | + | 47800942 | 47800956 | 8.36e-05 | 0.234 | `AGGGCAGGAAGTGCA` |
| 1 | chr20 | + | 48981443 | 48981457 | 8.36e-05 | 0.234 | `GGGGCAGTGGGGACA` |
| 1 | chr17 | + | 72006596 | 72006610 | 8.4e-05 | 0.235 | `GAGTCACTGAGGGTT` |
| 1 | chr12 | − | 67521206 | 67521220 | 8.44e-05 | 0.235 | `GGATCAGAAGGTTTA` |
| 1 | chr12 | + | 6941620 | 6941634 | 8.48e-05 | 0.236 | `AGGTCCCAAAGCCCA` |
| 1 | chr4 | − | 122280298 | 122280312 | 8.53e-05 | 0.237 | `GGTACAGTGGGGTCA` |
| 1 | chr8 | − | 103617782 | 103617796 | 8.53e-05 | 0.237 | `AGATCAGCAAGTCCC` |
| 1 | chr9 | − | 135193034 | 135193048 | 8.53e-05 | 0.237 | `GAGGCACTGAGGCCA` |
| 1 | chr1 | + | 144094092 | 144094106 | 8.61e-05 | 0.238 | `GGGGCGGAGAGTGCA` |
| 1 | chrX | − | 7012086 | 7012100 | 8.61e-05 | 0.238 | `GGGTGACAGGGTTTC` |
| 1 | chr12 | − | 6941703 | 6941717 | 8.61e-05 | 0.238 | `AGTTCCAGGAGGTCG` |
| 1 | chr1 | + | 154741707 | 154741721 | 8.65e-05 | 0.239 | `AGGACAGAAAGTTTG` |
| 1 | chr18 | − | 44733311 | 44733325 | 8.65e-05 | 0.239 | `GGGTGGCGGAGGTCT` |
| 1 | chr8 | + | 126718773 | 126718787 | 8.7e-05 | 0.24 | `AGTCCAGTGGGTTCT` |
| 1 | chr2 | + | 181885803 | 181885817 | 8.74e-05 | 0.24 | `AAGTCACCAGGCTCA` |
| 1 | chr5 | + | 158208426 | 158208440 | 8.74e-05 | 0.24 | `AGGTGACAGATGTCA` |
| 1 | chr11 | + | 65112262 | 65112276 | 8.74e-05 | 0.24 | `GGGTCGTGGAGGTCA` |
| 1 | chr2 | − | 177572614 | 177572628 | 8.79e-05 | 0.24 | `GGTTCATTCAGTTCA` |
| 1 | chr11 | − | 34761772 | 34761786 | 8.79e-05 | 0.24 | `GGTTCATTCAGTTCA` |
| 1 | chr22 | + | 49316155 | 49316169 | 8.79e-05 | 0.24 | `AGGGCAGAGGGTGCT` |
| 1 | chr4 | + | 40002190 | 40002204 | 8.83e-05 | 0.241 | `GGTTCACAGACTACT` |
| 1 | chr5 | − | 156862315 | 156862329 | 8.87e-05 | 0.241 | `AGGTCAAAGAGAACA` |
| 1 | chr12 | − | 91457379 | 91457393 | 8.87e-05 | 0.241 | `GGGAGGCTGAGGTCA` |
| 1 | chr7 | + | 4689511 | 4689525 | 8.91e-05 | 0.242 | `GGATCCCTGGGGTCA` |
| 1 | chr1 | + | 232576037 | 232576051 | 8.96e-05 | 0.243 | `CGGGCAGAGAGGTCG` |
| 1 | chr10 | + | 135052918 | 135052932 | 8.96e-05 | 0.243 | `GGGTCAGAGGCGGCA` |
| 1 | chr1 | + | 181706057 | 181706071 | 9e-05 | 0.243 | `GGGTGATTGGGGGCA` |
| 1 | chr20 | + | 23263033 | 23263047 | 9e-05 | 0.243 | `AGGTCAGGGGATTCA` |
| 1 | chr2 | + | 111650145 | 111650159 | 9.05e-05 | 0.244 | `AGGCCAGACAGGGCA` |
| 1 | chr6 | + | 91240351 | 91240365 | 9.14e-05 | 0.246 | `AGGTGAGTAGGTCTA` |
| 1 | chr11 | − | 8661003 | 8661017 | 9.14e-05 | 0.246 | `GGATGACTAGGGGCA` |
| 1 | chr8 | + | 101576250 | 101576264 | 9.19e-05 | 0.247 | `AGGACACTGGGGACT` |
| 1 | chr1 | − | 21493969 | 21493983 | 9.23e-05 | 0.248 | `CGGCCACAAAGGGCA` |
| 1 | chr5 | − | 106935181 | 106935195 | 9.23e-05 | 0.248 | `AATTCAGAGAGCCCA` |
| 1 | chr15 | − | 29296351 | 29296365 | 9.23e-05 | 0.248 | `GGGTGAACAAGGACC` |
| 1 | chr2 | + | 38715519 | 38715533 | 9.32e-05 | 0.248 | `AGGTCACGCAGTACT` |
| 1 | chr3 | + | 109327314 | 109327328 | 9.32e-05 | 0.248 | `GGTTCACTGAAGCCT` |
| 1 | chr5 | − | 139029887 | 139029901 | 9.32e-05 | 0.248 | `AGGCGAGAGGGGCCA` |
| 1 | chr9 | + | 113789194 | 113789208 | 9.32e-05 | 0.248 | `AGGTCATGGGGGTTA` |
| 1 | chr17 | + | 24320389 | 24320403 | 9.32e-05 | 0.248 | `GGGTGAAGCAGTTCA` |
| 1 | chr1 | + | 165905142 | 165905156 | 9.36e-05 | 0.249 | `AGTGGAAAAAGTTCT` |
| 1 | chr5 | − | 40443170 | 40443184 | 9.36e-05 | 0.249 | `TGACCAGAGAGGTCA` |
| 1 | chr7 | + | 4648130 | 4648144 | 9.36e-05 | 0.249 | `AGGGGAGTGGGGTCG` |
| 1 | chr10 | + | 81946231 | 81946245 | 9.46e-05 | 0.25 | `GGGGCAGGGAGGACA` |
| 1 | chr16 | + | 28765367 | 28765381 | 9.46e-05 | 0.25 | `CGGTCGCAGGGGGCA` |
| 1 | chr2 | − | 174538671 | 174538685 | 9.5e-05 | 0.251 | `GAGTCACCGGGGACG` |
| 1 | chr5 | + | 142763770 | 142763784 | 9.5e-05 | 0.251 | `GGGCCACCGAGTTTC` |
| 1 | chr3 | + | 173307808 | 173307822 | 9.55e-05 | 0.252 | `AGTTCATAGAATTCG` |
| 1 | chr16 | − | 66666516 | 66666530 | 9.55e-05 | 0.252 | `GGGTGGCAAAGGTTG` |
| 1 | chr5 | − | 131845099 | 131845113 | 9.6e-05 | 0.252 | `AGGACACTGGGGATA` |
| 1 | chr5 | − | 139907453 | 139907467 | 9.6e-05 | 0.252 | `AGGTCGAGGAGCGCA` |
| 1 | chr6 | + | 138070693 | 138070707 | 9.6e-05 | 0.252 | `AGTTCATTAGGGTTA` |
| 1 | chr19 | − | 53809885 | 53809899 | 9.6e-05 | 0.252 | `TGATGAGTGAGGCCA` |
| 1 | chr2 | − | 201796350 | 201796364 | 9.64e-05 | 0.252 | `GGGTCATTGGGGTCC` |
| 1 | chr11 | − | 601429 | 601443 | 9.64e-05 | 0.252 | `AGGCCACTGAGGGTC` |
| 1 | chr17 | + | 38801079 | 38801093 | 9.64e-05 | 0.252 | `AGTTCACACAGTGTT` |
| 1 | chr19 | + | 52153998 | 52154012 | 9.64e-05 | 0.252 | `AGGTCACAGATGTCT` |
| 1 | chr1 | − | 79699191 | 79699205 | 9.69e-05 | 0.253 | `AGGGGAATCAGTTCA` |
| 1 | chr15 | + | 73184352 | 73184366 | 9.74e-05 | 0.253 | `AGTTGAAAGGGTGTG` |
| 1 | chr11 | + | 65112562 | 65112576 | 9.78e-05 | 0.254 | `AGGCCAGAGAGGATT` |
| 1 | chr12 | − | 115844413 | 115844427 | 9.78e-05 | 0.254 | `GGGGGAAAAAGGTCT` |
| 1 | chr18 | + | 22257074 | 22257088 | 9.78e-05 | 0.254 | `GGGAGACAGAGGGCT` |
| 1 | chr16 | − | 10967007 | 10967021 | 9.83e-05 | 0.254 | `AGGTCACTAACCCCA` |
| 1 | chr18 | − | 59136339 | 59136353 | 9.83e-05 | 0.254 | `GGATGACTGAGTACC` |
| 1 | chr3 | + | 113763331 | 113763345 | 9.93e-05 | 0.256 | `AGGGCAGCGGGGCCG` |
| 1 | chr6 | − | 138246385 | 138246399 | 9.93e-05 | 0.256 | `GGGCCACAGGGTCCT` |
| 1 | chr9 | + | 116151120 | 116151134 | 9.93e-05 | 0.256 | `AGGGCAGTGGGTTCC` |
| 1 | chr6 | − | 26070752 | 26070766 | 9.98e-05 | 0.257 | `AGTTCACAGACTCTG` |
| 1 | chr11 | + | 62413686 | 62413700 | 9.98e-05 | 0.257 | `GGTGCAGAGAGTTTG` |

---

**DEBUGGING INFORMATION**


---

Command line:

```
/ebi/sw/MEME/VM-cluster410/meme-versions/4.10.0/bin/fimo --parse-genomic-coord --verbosity 1 --oc fimo_out_1 --bgfile ./background --motif 1 meme_out/meme.xml ./Supplementary_Table_1.500bp.fa
```

Settings:

```
|  |  |  |
| --- | --- | --- |
| output directory = fimo_out_1 | MEME file name = meme_out/meme.xml | sequence file name = ./Supplementary_Table_1.500bp.fa |
| background file name = ./background | allow clobber = true | compute q-values = true |
| parse genomic coord. = true | text only = false | scan both strands = true |
| max sequence length = 250000000 | output threshold = 0.0001 | threshold type = p-value |
| max stored scores = 100000 | pseudocount = 0.1 | verbosity = 1 |
| selected motif = 1 |  |  |
```

This information can be useful in the event you wish to report a
problem with the FIMO software.

---

**Go to top**
