# Supplementary material for: The MEME Suite
Source: Nucleic Acids Res. 2015 May 7;43(Web Server issue):W39–49. doi: 10.1093/nar/gkv416 (PMC4489269; doi:10.1093/nar/gkv416)
Supplement: SUPPLEMENTARY DATA [file supp_gkv416_nar-00283-web-b-2015-File005.zip › case4/meme-chip/fimo_out_10/fimo.html]

FIMO Results


---

|  |  |  |
| --- | --- | --- |
| **Database and Motifs** | **High-scoring Motif Occurrences** | **Debugging Information** |

  
  

---

**FIMO - Motif search tool**


---

FIMO version 4.10.0,
(Release date: Wed May 21 10:35:36 2014 +1000)

For further information on how to interpret these results
or to get a copy of the FIMO software please access
http://meme.nbcr.net

If you use FIMO in your research, please cite the following paper:  
Charles E. Grant, Timothy L. Bailey, and William Stafford Noble,
"FIMO: Scanning for occurrences of a given motif",
*Bioinformatics*, **27**(7):1017-1018, 2011.
[full text]

---

**DATABASE AND MOTIFS**


---

DATABASE
./Supplementary\_Table\_1.500bp.fa  
Database contains
2776
sequences,
1388000
residues

MOTIFS
dreme\_out/dreme.xml
(nucleotide)

| MOTIF | WIDTH | BEST POSSIBLE MATCH |
| --- | --- | --- |
| GGAARY | 6 | GGAAGT |
| AVTGAAA | 7 | ACTGAAA |
| RCAGCTGY | 8 | GCAGCTGC |
| AKAAAH | 6 | AGAAAA |
| RAGKTCA | 7 | GAGGTCA |
| CMCAGM | 6 | CCCAGC |
| CCCCRCCC | 8 | CCCCGCCC |
| AAATR | 5 | AAATG |
| GAAASCA | 7 | GAAAGCA |
| CCGSCTCC | 8 | CCGCCTCC |
| CCWCCTGC | 8 | CCACCTGC |

Random model letter frequencies
(from ./background):
  
A 0.241 C 0.259 G 0.259 T 0.241

---

**SECTION I: HIGH-SCORING MOTIF OCCURRENCES**


---

- There were
  1161
  motif occurrences with a
  p-value less than
  0.0001.
  **Only the most significant
  1000
  are shown here.**
  The full set of motif occurrences can be seen in the
  tab-delimited plain text output file
  fimo.txt,
  the GFF file
  fimo.gff
  which may be suitable for uploading to the
  UCSC Genome Table Browser
  (assuming the FASTA input sequences included genomic coordinates in UCSC or Galaxy format),
  or the XML file
  fimo.xml.
- The p-value of a motif occurrence is defined as the
  probability of a random sequence of the same length as the motif
  matching that position of the sequence with as good or better a score.
- The score for the match of a position in a sequence to a motif
  is computed by summing the appropriate entries from each column of
  the position-dependent scoring matrix that represents the motif.
- The q-value of a motif occurrence is defined as the
  false discovery rate if the occurrence is accepted as significant.
- The table is sorted by increasing p-value.

| Motif | Sequence Name | Strand | Start | End | p-value | q-value | Matched Sequence |
| --- | --- | --- | --- | --- | --- | --- | --- |
| CCCCRCCC | chr1 | − | 25099419 | 25099426 | 1.9e-05 | 0.111 | `CCCCACCC` |
| CCCCRCCC | chr1 | + | 26010192 | 26010199 | 1.9e-05 | 0.111 | `CCCCACCC` |
| CCCCRCCC | chr1 | + | 27824139 | 27824146 | 1.9e-05 | 0.111 | `CCCCACCC` |
| CCCCRCCC | chr1 | − | 27825394 | 27825401 | 1.9e-05 | 0.111 | `CCCCACCC` |
| CCCCRCCC | chr1 | − | 30995356 | 30995363 | 1.9e-05 | 0.111 | `CCCCACCC` |
| CCCCRCCC | chr1 | + | 39228996 | 39229003 | 1.9e-05 | 0.111 | `CCCCACCC` |
| CCCCRCCC | chr1 | − | 39229331 | 39229338 | 1.9e-05 | 0.111 | `CCCCACCC` |
| CCCCRCCC | chr1 | − | 39229337 | 39229344 | 1.9e-05 | 0.111 | `CCCCACCC` |
| CCCCRCCC | chr1 | + | 42045168 | 42045175 | 1.9e-05 | 0.111 | `CCCCACCC` |
| CCCCRCCC | chr1 | + | 44960008 | 44960015 | 1.9e-05 | 0.111 | `CCCCACCC` |
| CCCCRCCC | chr1 | + | 45759984 | 45759991 | 1.9e-05 | 0.111 | `CCCCACCC` |
| CCCCRCCC | chr1 | + | 45760324 | 45760331 | 1.9e-05 | 0.111 | `CCCCACCC` |
| CCCCRCCC | chr1 | + | 111565979 | 111565986 | 1.9e-05 | 0.111 | `CCCCACCC` |
| CCCCRCCC | chr1 | + | 111936955 | 111936962 | 1.9e-05 | 0.111 | `CCCCACCC` |
| CCCCRCCC | chr1 | + | 148125044 | 148125051 | 1.9e-05 | 0.111 | `CCCCACCC` |
| CCCCRCCC | chr1 | − | 148125276 | 148125283 | 1.9e-05 | 0.111 | `CCCCACCC` |
| CCCCRCCC | chr1 | − | 148398348 | 148398355 | 1.9e-05 | 0.111 | `CCCCACCC` |
| CCCCRCCC | chr1 | + | 149850945 | 149850952 | 1.9e-05 | 0.111 | `CCCCACCC` |
| CCCCRCCC | chr1 | + | 153176045 | 153176052 | 1.9e-05 | 0.111 | `CCCCACCC` |
| CCCCRCCC | chr1 | + | 154453137 | 154453144 | 1.9e-05 | 0.111 | `CCCCACCC` |
| CCCCRCCC | chr1 | − | 154741487 | 154741494 | 1.9e-05 | 0.111 | `CCCCACCC` |
| CCCCRCCC | chr1 | + | 154741806 | 154741813 | 1.9e-05 | 0.111 | `CCCCACCC` |
| CCCCRCCC | chr1 | − | 158865964 | 158865971 | 1.9e-05 | 0.111 | `CCCCACCC` |
| CCCCRCCC | chr1 | + | 158866966 | 158866973 | 1.9e-05 | 0.111 | `CCCCACCC` |
| CCCCRCCC | chr1 | − | 158992593 | 158992600 | 1.9e-05 | 0.111 | `CCCCACCC` |
| CCCCRCCC | chr1 | + | 179634457 | 179634464 | 1.9e-05 | 0.111 | `CCCCACCC` |
| CCCCRCCC | chr1 | − | 201557100 | 201557107 | 1.9e-05 | 0.111 | `CCCCACCC` |
| CCCCRCCC | chr1 | − | 204919938 | 204919945 | 1.9e-05 | 0.111 | `CCCCACCC` |
| CCCCRCCC | chr1 | + | 211219706 | 211219713 | 1.9e-05 | 0.111 | `CCCCACCC` |
| CCCCRCCC | chr1 | + | 228391021 | 228391028 | 1.9e-05 | 0.111 | `CCCCACCC` |
| CCCCRCCC | chr1 | − | 232576068 | 232576075 | 1.9e-05 | 0.111 | `CCCCACCC` |
| CCCCRCCC | chr2 | − | 9699526 | 9699533 | 1.9e-05 | 0.111 | `CCCCACCC` |
| CCCCRCCC | chr2 | − | 3600912 | 3600919 | 1.9e-05 | 0.111 | `CCCCACCC` |
| CCCCRCCC | chr2 | + | 11888260 | 11888267 | 1.9e-05 | 0.111 | `CCCCACCC` |
| CCCCRCCC | chr2 | − | 12786007 | 12786014 | 1.9e-05 | 0.111 | `CCCCACCC` |
| CCCCRCCC | chr2 | + | 27433209 | 27433216 | 1.9e-05 | 0.111 | `CCCCACCC` |
| CCCCRCCC | chr2 | + | 33557994 | 33558001 | 1.9e-05 | 0.111 | `CCCCACCC` |
| CCCCRCCC | chr2 | + | 47391358 | 47391365 | 1.9e-05 | 0.111 | `CCCCACCC` |
| CCCCRCCC | chr2 | − | 54649821 | 54649828 | 1.9e-05 | 0.111 | `CCCCACCC` |
| CCCCRCCC | chr2 | + | 54650522 | 54650529 | 1.9e-05 | 0.111 | `CCCCACCC` |
| CCCCRCCC | chr2 | − | 70167544 | 70167551 | 1.9e-05 | 0.111 | `CCCCACCC` |
| CCCCRCCC | chr2 | + | 70170160 | 70170167 | 1.9e-05 | 0.111 | `CCCCACCC` |
| CCCCRCCC | chr2 | − | 111642734 | 111642741 | 1.9e-05 | 0.111 | `CCCCACCC` |
| CCCCRCCC | chr2 | − | 157826046 | 157826053 | 1.9e-05 | 0.111 | `CCCCACCC` |
| CCCCRCCC | chr2 | + | 177837815 | 177837822 | 1.9e-05 | 0.111 | `CCCCACCC` |
| CCCCRCCC | chr2 | − | 177838167 | 177838174 | 1.9e-05 | 0.111 | `CCCCACCC` |
| CCCCRCCC | chr2 | − | 201689208 | 201689215 | 1.9e-05 | 0.111 | `CCCCACCC` |
| CCCCRCCC | chr2 | − | 201831436 | 201831443 | 1.9e-05 | 0.111 | `CCCCACCC` |
| CCCCRCCC | chr2 | − | 231233731 | 231233738 | 1.9e-05 | 0.111 | `CCCCACCC` |
| CCCCRCCC | chr2 | + | 232235163 | 232235170 | 1.9e-05 | 0.111 | `CCCCACCC` |
| CCCCRCCC | chr2 | − | 233632769 | 233632776 | 1.9e-05 | 0.111 | `CCCCACCC` |
| CCCCRCCC | chr2 | + | 241903770 | 241903777 | 1.9e-05 | 0.111 | `CCCCACCC` |
| CCCCRCCC | chr3 | + | 9413226 | 9413233 | 1.9e-05 | 0.111 | `CCCCACCC` |
| CCCCRCCC | chr3 | + | 9413693 | 9413700 | 1.9e-05 | 0.111 | `CCCCACCC` |
| CCCCRCCC | chr3 | − | 13100601 | 13100608 | 1.9e-05 | 0.111 | `CCCCACCC` |
| CCCCRCCC | chr3 | + | 13104555 | 13104562 | 1.9e-05 | 0.111 | `CCCCACCC` |
| CCCCRCCC | chr3 | + | 14668013 | 14668020 | 1.9e-05 | 0.111 | `CCCCACCC` |
| CCCCRCCC | chr3 | − | 23933955 | 23933962 | 1.9e-05 | 0.111 | `CCCCACCC` |
| CCCCRCCC | chr3 | − | 40977949 | 40977956 | 1.9e-05 | 0.111 | `CCCCACCC` |
| CCCCRCCC | chr3 | + | 45570796 | 45570803 | 1.9e-05 | 0.111 | `CCCCACCC` |
| CCCCRCCC | chr3 | + | 45570801 | 45570808 | 1.9e-05 | 0.111 | `CCCCACCC` |
| CCCCRCCC | chr3 | + | 46943699 | 46943706 | 1.9e-05 | 0.111 | `CCCCACCC` |
| CCCCRCCC | chr3 | − | 46943818 | 46943825 | 1.9e-05 | 0.111 | `CCCCACCC` |
| CCCCRCCC | chr3 | + | 49019452 | 49019459 | 1.9e-05 | 0.111 | `CCCCACCC` |
| CCCCRCCC | chr3 | + | 57077714 | 57077721 | 1.9e-05 | 0.111 | `CCCCACCC` |
| CCCCRCCC | chr3 | − | 72117628 | 72117635 | 1.9e-05 | 0.111 | `CCCCACCC` |
| CCCCRCCC | chr3 | − | 113662380 | 113662387 | 1.9e-05 | 0.111 | `CCCCACCC` |
| CCCCRCCC | chr3 | − | 116347917 | 116347924 | 1.9e-05 | 0.111 | `CCCCACCC` |
| CCCCRCCC | chr3 | + | 121296953 | 121296960 | 1.9e-05 | 0.111 | `CCCCACCC` |
| CCCCRCCC | chr3 | − | 123811899 | 123811906 | 1.9e-05 | 0.111 | `CCCCACCC` |
| CCCCRCCC | chr3 | − | 126342700 | 126342707 | 1.9e-05 | 0.111 | `CCCCACCC` |
| CCCCRCCC | chr3 | − | 126342705 | 126342712 | 1.9e-05 | 0.111 | `CCCCACCC` |
| CCCCRCCC | chr3 | + | 157874990 | 157874997 | 1.9e-05 | 0.111 | `CCCCACCC` |
| CCCCRCCC | chr3 | + | 178559196 | 178559203 | 1.9e-05 | 0.111 | `CCCCACCC` |
| CCCCRCCC | chr3 | + | 178559202 | 178559209 | 1.9e-05 | 0.111 | `CCCCACCC` |
| CCCCRCCC | chr3 | + | 178560512 | 178560519 | 1.9e-05 | 0.111 | `CCCCACCC` |
| CCCCRCCC | chr3 | + | 196746266 | 196746273 | 1.9e-05 | 0.111 | `CCCCACCC` |
| CCCCRCCC | chr4 | + | 2717911 | 2717918 | 1.9e-05 | 0.111 | `CCCCACCC` |
| CCCCRCCC | chr4 | − | 2758904 | 2758911 | 1.9e-05 | 0.111 | `CCCCACCC` |
| CCCCRCCC | chr4 | − | 2758945 | 2758952 | 1.9e-05 | 0.111 | `CCCCACCC` |
| CCCCRCCC | chr4 | + | 79754263 | 79754270 | 1.9e-05 | 0.111 | `CCCCACCC` |
| CCCCRCCC | chr4 | + | 102159482 | 102159489 | 1.9e-05 | 0.111 | `CCCCACCC` |
| CCCCRCCC | chr4 | + | 147240047 | 147240054 | 1.9e-05 | 0.111 | `CCCCACCC` |
| CCCCRCCC | chr5 | − | 32212552 | 32212559 | 1.9e-05 | 0.111 | `CCCCACCC` |
| CCCCRCCC | chr5 | + | 32622333 | 32622340 | 1.9e-05 | 0.111 | `CCCCACCC` |
| CCCCRCCC | chr5 | − | 55480421 | 55480428 | 1.9e-05 | 0.111 | `CCCCACCC` |
| CCCCRCCC | chr5 | + | 90712593 | 90712600 | 1.9e-05 | 0.111 | `CCCCACCC` |
| CCCCRCCC | chr5 | + | 107746351 | 107746358 | 1.9e-05 | 0.111 | `CCCCACCC` |
| CCCCRCCC | chr5 | + | 131037719 | 131037726 | 1.9e-05 | 0.111 | `CCCCACCC` |
| CCCCRCCC | chr5 | + | 138925212 | 138925219 | 1.9e-05 | 0.111 | `CCCCACCC` |
| CCCCRCCC | chr5 | + | 150455285 | 150455292 | 1.9e-05 | 0.111 | `CCCCACCC` |
| CCCCRCCC | chr5 | − | 150455410 | 150455417 | 1.9e-05 | 0.111 | `CCCCACCC` |
| CCCCRCCC | chr5 | − | 172504076 | 172504083 | 1.9e-05 | 0.111 | `CCCCACCC` |
| CCCCRCCC | chr5 | + | 180169738 | 180169745 | 1.9e-05 | 0.111 | `CCCCACCC` |
| CCCCRCCC | chr5 | − | 180582737 | 180582744 | 1.9e-05 | 0.111 | `CCCCACCC` |
| CCCCRCCC | chr6 | − | 174297 | 174304 | 1.9e-05 | 0.111 | `CCCCACCC` |
| CCCCRCCC | chr6 | + | 237742 | 237749 | 1.9e-05 | 0.111 | `CCCCACCC` |
| CCCCRCCC | chr6 | + | 237747 | 237754 | 1.9e-05 | 0.111 | `CCCCACCC` |
| CCCCRCCC | chr6 | + | 310003 | 310010 | 1.9e-05 | 0.111 | `CCCCACCC` |
| CCCCRCCC | chr6 | − | 24828310 | 24828317 | 1.9e-05 | 0.111 | `CCCCACCC` |
| CCCCRCCC | chr6 | − | 26071149 | 26071156 | 1.9e-05 | 0.111 | `CCCCACCC` |
| CCCCRCCC | chr6 | + | 26129684 | 26129691 | 1.9e-05 | 0.111 | `CCCCACCC` |
| CCCCRCCC | chr6 | − | 26152022 | 26152029 | 1.9e-05 | 0.111 | `CCCCACCC` |
| CCCCRCCC | chr6 | + | 30585277 | 30585284 | 1.9e-05 | 0.111 | `CCCCACCC` |
| CCCCRCCC | chr6 | − | 30796770 | 30796777 | 1.9e-05 | 0.111 | `CCCCACCC` |
| CCCCRCCC | chr6 | + | 31430839 | 31430846 | 1.9e-05 | 0.111 | `CCCCACCC` |
| CCCCRCCC | chr6 | + | 31430839 | 31430846 | 1.9e-05 | 0.111 | `CCCCACCC` |
| CCCCRCCC | chr6 | + | 31728452 | 31728459 | 1.9e-05 | 0.111 | `CCCCACCC` |
| CCCCRCCC | chr6 | − | 33047860 | 33047867 | 1.9e-05 | 0.111 | `CCCCACCC` |
| CCCCRCCC | chr6 | + | 33646984 | 33646991 | 1.9e-05 | 0.111 | `CCCCACCC` |
| CCCCRCCC | chr6 | + | 36756218 | 36756225 | 1.9e-05 | 0.111 | `CCCCACCC` |
| CCCCRCCC | chr6 | − | 36829233 | 36829240 | 1.9e-05 | 0.111 | `CCCCACCC` |
| CCCCRCCC | chr6 | − | 42822004 | 42822011 | 1.9e-05 | 0.111 | `CCCCACCC` |
| CCCCRCCC | chr6 | − | 42859142 | 42859149 | 1.9e-05 | 0.111 | `CCCCACCC` |
| CCCCRCCC | chr6 | − | 52471268 | 52471275 | 1.9e-05 | 0.111 | `CCCCACCC` |
| CCCCRCCC | chr6 | + | 143310393 | 143310400 | 1.9e-05 | 0.111 | `CCCCACCC` |
| CCCCRCCC | chr6 | + | 157061406 | 157061413 | 1.9e-05 | 0.111 | `CCCCACCC` |
| CCCCRCCC | chr6 | + | 157061412 | 157061419 | 1.9e-05 | 0.111 | `CCCCACCC` |
| CCCCRCCC | chr7 | − | 2984824 | 2984831 | 1.9e-05 | 0.111 | `CCCCACCC` |
| CCCCRCCC | chr7 | − | 5564408 | 5564415 | 1.9e-05 | 0.111 | `CCCCACCC` |
| CCCCRCCC | chr7 | + | 44071181 | 44071188 | 1.9e-05 | 0.111 | `CCCCACCC` |
| CCCCRCCC | chr7 | + | 55569798 | 55569805 | 1.9e-05 | 0.111 | `CCCCACCC` |
| CCCCRCCC | chr7 | + | 73263395 | 73263402 | 1.9e-05 | 0.111 | `CCCCACCC` |
| CCCCRCCC | chr7 | − | 73281738 | 73281745 | 1.9e-05 | 0.111 | `CCCCACCC` |
| CCCCRCCC | chr7 | − | 86686901 | 86686908 | 1.9e-05 | 0.111 | `CCCCACCC` |
| CCCCRCCC | chr7 | − | 100019312 | 100019319 | 1.9e-05 | 0.111 | `CCCCACCC` |
| CCCCRCCC | chr7 | − | 100515578 | 100515585 | 1.9e-05 | 0.111 | `CCCCACCC` |
| CCCCRCCC | chr7 | − | 101719267 | 101719274 | 1.9e-05 | 0.111 | `CCCCACCC` |
| CCCCRCCC | chr7 | + | 101854212 | 101854219 | 1.9e-05 | 0.111 | `CCCCACCC` |
| CCCCRCCC | chr7 | + | 104440763 | 104440770 | 1.9e-05 | 0.111 | `CCCCACCC` |
| CCCCRCCC | chr7 | − | 106597528 | 106597535 | 1.9e-05 | 0.111 | `CCCCACCC` |
| CCCCRCCC | chr7 | + | 128362308 | 128362315 | 1.9e-05 | 0.111 | `CCCCACCC` |
| CCCCRCCC | chr7 | − | 149733643 | 149733650 | 1.9e-05 | 0.111 | `CCCCACCC` |
| CCCCRCCC | chr8 | + | 144179114 | 144179121 | 1.9e-05 | 0.111 | `CCCCACCC` |
| CCCCRCCC | chr8 | − | 17824563 | 17824570 | 1.9e-05 | 0.111 | `CCCCACCC` |
| CCCCRCCC | chr8 | + | 21953489 | 21953496 | 1.9e-05 | 0.111 | `CCCCACCC` |
| CCCCRCCC | chr8 | + | 21953520 | 21953527 | 1.9e-05 | 0.111 | `CCCCACCC` |
| CCCCRCCC | chr8 | − | 41600079 | 41600086 | 1.9e-05 | 0.111 | `CCCCACCC` |
| CCCCRCCC | chr8 | + | 42159006 | 42159013 | 1.9e-05 | 0.111 | `CCCCACCC` |
| CCCCRCCC | chr8 | − | 57149806 | 57149813 | 1.9e-05 | 0.111 | `CCCCACCC` |
| CCCCRCCC | chr8 | − | 62073565 | 62073572 | 1.9e-05 | 0.111 | `CCCCACCC` |
| CCCCRCCC | chr8 | + | 72914176 | 72914183 | 1.9e-05 | 0.111 | `CCCCACCC` |
| CCCCRCCC | chr8 | + | 98725488 | 98725495 | 1.9e-05 | 0.111 | `CCCCACCC` |
| CCCCRCCC | chr8 | + | 101998684 | 101998691 | 1.9e-05 | 0.111 | `CCCCACCC` |
| CCCCRCCC | chr8 | − | 105768258 | 105768265 | 1.9e-05 | 0.111 | `CCCCACCC` |
| CCCCRCCC | chr8 | + | 126730296 | 126730303 | 1.9e-05 | 0.111 | `CCCCACCC` |
| CCCCRCCC | chr8 | + | 129395597 | 129395604 | 1.9e-05 | 0.111 | `CCCCACCC` |
| CCCCRCCC | chr8 | + | 134563958 | 134563965 | 1.9e-05 | 0.111 | `CCCCACCC` |
| CCCCRCCC | chr8 | − | 134581024 | 134581031 | 1.9e-05 | 0.111 | `CCCCACCC` |
| CCCCRCCC | chr9 | − | 3515989 | 3515996 | 1.9e-05 | 0.111 | `CCCCACCC` |
| CCCCRCCC | chr9 | − | 3516078 | 3516085 | 1.9e-05 | 0.111 | `CCCCACCC` |
| CCCCRCCC | chr9 | − | 36142061 | 36142068 | 1.9e-05 | 0.111 | `CCCCACCC` |
| CCCCRCCC | chr9 | − | 36983983 | 36983990 | 1.9e-05 | 0.111 | `CCCCACCC` |
| CCCCRCCC | chr9 | + | 36984083 | 36984090 | 1.9e-05 | 0.111 | `CCCCACCC` |
| CCCCRCCC | chr9 | + | 70788049 | 70788056 | 1.9e-05 | 0.111 | `CCCCACCC` |
| CCCCRCCC | chr9 | + | 91268853 | 91268860 | 1.9e-05 | 0.111 | `CCCCACCC` |
| CCCCRCCC | chr9 | − | 91269091 | 91269098 | 1.9e-05 | 0.111 | `CCCCACCC` |
| CCCCRCCC | chr9 | + | 116151225 | 116151232 | 1.9e-05 | 0.111 | `CCCCACCC` |
| CCCCRCCC | chr9 | − | 116483935 | 116483942 | 1.9e-05 | 0.111 | `CCCCACCC` |
| CCCCRCCC | chr9 | + | 121838831 | 121838838 | 1.9e-05 | 0.111 | `CCCCACCC` |
| CCCCRCCC | chr9 | − | 122699570 | 122699577 | 1.9e-05 | 0.111 | `CCCCACCC` |
| CCCCRCCC | chr9 | + | 125141365 | 125141372 | 1.9e-05 | 0.111 | `CCCCACCC` |
| CCCCRCCC | chr9 | − | 131688287 | 131688294 | 1.9e-05 | 0.111 | `CCCCACCC` |
| CCCCRCCC | chrX | + | 134483647 | 134483654 | 1.9e-05 | 0.111 | `CCCCACCC` |
| CCCCRCCC | chrX | − | 151750378 | 151750385 | 1.9e-05 | 0.111 | `CCCCACCC` |
| CCCCRCCC | chrX | + | 153282950 | 153282957 | 1.9e-05 | 0.111 | `CCCCACCC` |
| CCCCRCCC | chr10 | + | 1085103 | 1085110 | 1.9e-05 | 0.111 | `CCCCACCC` |
| CCCCRCCC | chr10 | − | 73705635 | 73705642 | 1.9e-05 | 0.111 | `CCCCACCC` |
| CCCCRCCC | chr10 | − | 73761544 | 73761551 | 1.9e-05 | 0.111 | `CCCCACCC` |
| CCCCRCCC | chr10 | − | 73761697 | 73761704 | 1.9e-05 | 0.111 | `CCCCACCC` |
| CCCCRCCC | chr10 | − | 81946338 | 81946345 | 1.9e-05 | 0.111 | `CCCCACCC` |
| CCCCRCCC | chr10 | + | 81946515 | 81946522 | 1.9e-05 | 0.111 | `CCCCACCC` |
| CCCCRCCC | chr10 | − | 104411036 | 104411043 | 1.9e-05 | 0.111 | `CCCCACCC` |
| CCCCRCCC | chr10 | − | 104411168 | 104411175 | 1.9e-05 | 0.111 | `CCCCACCC` |
| CCCCRCCC | chr10 | − | 104411276 | 104411283 | 1.9e-05 | 0.111 | `CCCCACCC` |
| CCCCRCCC | chr10 | − | 105221133 | 105221140 | 1.9e-05 | 0.111 | `CCCCACCC` |
| CCCCRCCC | chr10 | − | 105221260 | 105221267 | 1.9e-05 | 0.111 | `CCCCACCC` |
| CCCCRCCC | chr10 | − | 112145893 | 112145900 | 1.9e-05 | 0.111 | `CCCCACCC` |
| CCCCRCCC | chr10 | + | 112164580 | 112164587 | 1.9e-05 | 0.111 | `CCCCACCC` |
| CCCCRCCC | chr10 | − | 121239106 | 121239113 | 1.9e-05 | 0.111 | `CCCCACCC` |
| CCCCRCCC | chr10 | − | 126397795 | 126397802 | 1.9e-05 | 0.111 | `CCCCACCC` |
| CCCCRCCC | chr11 | − | 1830849 | 1830856 | 1.9e-05 | 0.111 | `CCCCACCC` |
| CCCCRCCC | chr11 | − | 34219466 | 34219473 | 1.9e-05 | 0.111 | `CCCCACCC` |
| CCCCRCCC | chr11 | − | 35104843 | 35104850 | 1.9e-05 | 0.111 | `CCCCACCC` |
| CCCCRCCC | chr11 | + | 48088087 | 48088094 | 1.9e-05 | 0.111 | `CCCCACCC` |
| CCCCRCCC | chr11 | − | 59979662 | 59979669 | 1.9e-05 | 0.111 | `CCCCACCC` |
| CCCCRCCC | chr11 | − | 64375752 | 64375759 | 1.9e-05 | 0.111 | `CCCCACCC` |
| CCCCRCCC | chr11 | − | 64620941 | 64620948 | 1.9e-05 | 0.111 | `CCCCACCC` |
| CCCCRCCC | chr11 | + | 64641866 | 64641873 | 1.9e-05 | 0.111 | `CCCCACCC` |
| CCCCRCCC | chr11 | + | 64651051 | 64651058 | 1.9e-05 | 0.111 | `CCCCACCC` |
| CCCCRCCC | chr11 | + | 64656473 | 64656480 | 1.9e-05 | 0.111 | `CCCCACCC` |
| CCCCRCCC | chr11 | − | 64943218 | 64943225 | 1.9e-05 | 0.111 | `CCCCACCC` |
| CCCCRCCC | chr11 | + | 64945888 | 64945895 | 1.9e-05 | 0.111 | `CCCCACCC` |
| CCCCRCCC | chr11 | + | 64945894 | 64945901 | 1.9e-05 | 0.111 | `CCCCACCC` |
| CCCCRCCC | chr11 | − | 67796465 | 67796472 | 1.9e-05 | 0.111 | `CCCCACCC` |
| CCCCRCCC | chr11 | + | 73168157 | 73168164 | 1.9e-05 | 0.111 | `CCCCACCC` |
| CCCCRCCC | chr11 | + | 85603707 | 85603714 | 1.9e-05 | 0.111 | `CCCCACCC` |
| CCCCRCCC | chr11 | − | 101693372 | 101693379 | 1.9e-05 | 0.111 | `CCCCACCC` |
| CCCCRCCC | chr11 | + | 118072025 | 118072032 | 1.9e-05 | 0.111 | `CCCCACCC` |
| CCCCRCCC | chr11 | − | 122438325 | 122438332 | 1.9e-05 | 0.111 | `CCCCACCC` |
| CCCCRCCC | chr11 | + | 124445883 | 124445890 | 1.9e-05 | 0.111 | `CCCCACCC` |
| CCCCRCCC | chr11 | − | 124445997 | 124446004 | 1.9e-05 | 0.111 | `CCCCACCC` |
| CCCCRCCC | chr11 | − | 124446245 | 124446252 | 1.9e-05 | 0.111 | `CCCCACCC` |
| CCCCRCCC | chr11 | − | 127844603 | 127844610 | 1.9e-05 | 0.111 | `CCCCACCC` |
| CCCCRCCC | chr12 | − | 4098150 | 4098157 | 1.9e-05 | 0.111 | `CCCCACCC` |
| CCCCRCCC | chr12 | − | 4122363 | 4122370 | 1.9e-05 | 0.111 | `CCCCACCC` |
| CCCCRCCC | chr12 | + | 4122601 | 4122608 | 1.9e-05 | 0.111 | `CCCCACCC` |
| CCCCRCCC | chr12 | − | 15832722 | 15832729 | 1.9e-05 | 0.111 | `CCCCACCC` |
| CCCCRCCC | chr12 | − | 46493037 | 46493044 | 1.9e-05 | 0.111 | `CCCCACCC` |
| CCCCRCCC | chr12 | + | 46493246 | 46493253 | 1.9e-05 | 0.111 | `CCCCACCC` |
| CCCCRCCC | chr12 | + | 46493399 | 46493406 | 1.9e-05 | 0.111 | `CCCCACCC` |
| CCCCRCCC | chr12 | − | 46550535 | 46550542 | 1.9e-05 | 0.111 | `CCCCACCC` |
| CCCCRCCC | chr12 | + | 48343243 | 48343250 | 1.9e-05 | 0.111 | `CCCCACCC` |
| CCCCRCCC | chr12 | + | 67488021 | 67488028 | 1.9e-05 | 0.111 | `CCCCACCC` |
| CCCCRCCC | chr12 | + | 67488049 | 67488056 | 1.9e-05 | 0.111 | `CCCCACCC` |
| CCCCRCCC | chr12 | − | 67488773 | 67488780 | 1.9e-05 | 0.111 | `CCCCACCC` |
| CCCCRCCC | chr12 | + | 91062675 | 91062682 | 1.9e-05 | 0.111 | `CCCCACCC` |
| CCCCRCCC | chr12 | + | 91322015 | 91322022 | 1.9e-05 | 0.111 | `CCCCACCC` |
| CCCCRCCC | chr12 | + | 91457402 | 91457409 | 1.9e-05 | 0.111 | `CCCCACCC` |
| CCCCRCCC | chr12 | − | 107552006 | 107552013 | 1.9e-05 | 0.111 | `CCCCACCC` |
| CCCCRCCC | chr12 | + | 109584071 | 109584078 | 1.9e-05 | 0.111 | `CCCCACCC` |
| CCCCRCCC | chr12 | − | 115481823 | 115481830 | 1.9e-05 | 0.111 | `CCCCACCC` |
| CCCCRCCC | chr12 | + | 121914750 | 121914757 | 1.9e-05 | 0.111 | `CCCCACCC` |
| CCCCRCCC | chr13 | + | 30089758 | 30089765 | 1.9e-05 | 0.111 | `CCCCACCC` |
| CCCCRCCC | chr13 | − | 49838282 | 49838289 | 1.9e-05 | 0.111 | `CCCCACCC` |
| CCCCRCCC | chr13 | + | 76799293 | 76799300 | 1.9e-05 | 0.111 | `CCCCACCC` |
| CCCCRCCC | chr14 | + | 22089234 | 22089241 | 1.9e-05 | 0.111 | `CCCCACCC` |
| CCCCRCCC | chr14 | − | 23700291 | 23700298 | 1.9e-05 | 0.111 | `CCCCACCC` |
| CCCCRCCC | chr14 | + | 23700694 | 23700701 | 1.9e-05 | 0.111 | `CCCCACCC` |
| CCCCRCCC | chr14 | − | 23700738 | 23700745 | 1.9e-05 | 0.111 | `CCCCACCC` |
| CCCCRCCC | chr14 | − | 34938400 | 34938407 | 1.9e-05 | 0.111 | `CCCCACCC` |
| CCCCRCCC | chr14 | + | 50358273 | 50358280 | 1.9e-05 | 0.111 | `CCCCACCC` |
| CCCCRCCC | chr14 | + | 67747691 | 67747698 | 1.9e-05 | 0.111 | `CCCCACCC` |
| CCCCRCCC | chr14 | − | 68325399 | 68325406 | 1.9e-05 | 0.111 | `CCCCACCC` |
| CCCCRCCC | chr14 | − | 68325408 | 68325415 | 1.9e-05 | 0.111 | `CCCCACCC` |
| CCCCRCCC | chr14 | + | 69303421 | 69303428 | 1.9e-05 | 0.111 | `CCCCACCC` |
| CCCCRCCC | chr14 | + | 95637033 | 95637040 | 1.9e-05 | 0.111 | `CCCCACCC` |
| CCCCRCCC | chr14 | + | 104585325 | 104585332 | 1.9e-05 | 0.111 | `CCCCACCC` |
| CCCCRCCC | chr15 | − | 29295149 | 29295156 | 1.9e-05 | 0.111 | `CCCCACCC` |
| CCCCRCCC | chr15 | − | 29408822 | 29408829 | 1.9e-05 | 0.111 | `CCCCACCC` |
| CCCCRCCC | chr15 | − | 29441015 | 29441022 | 1.9e-05 | 0.111 | `CCCCACCC` |
| CCCCRCCC | chr15 | + | 43534676 | 43534683 | 1.9e-05 | 0.111 | `CCCCACCC` |
| CCCCRCCC | chr15 | − | 56533899 | 56533906 | 1.9e-05 | 0.111 | `CCCCACCC` |
| CCCCRCCC | chr15 | − | 57623530 | 57623537 | 1.9e-05 | 0.111 | `CCCCACCC` |
| CCCCRCCC | chr15 | + | 61583571 | 61583578 | 1.9e-05 | 0.111 | `CCCCACCC` |
| CCCCRCCC | chr15 | + | 62973070 | 62973077 | 1.9e-05 | 0.111 | `CCCCACCC` |
| CCCCRCCC | chr15 | − | 62973234 | 62973241 | 1.9e-05 | 0.111 | `CCCCACCC` |
| CCCCRCCC | chr15 | − | 68584133 | 68584140 | 1.9e-05 | 0.111 | `CCCCACCC` |
| CCCCRCCC | chr15 | − | 72476767 | 72476774 | 1.9e-05 | 0.111 | `CCCCACCC` |
| CCCCRCCC | chr15 | + | 72879909 | 72879916 | 1.9e-05 | 0.111 | `CCCCACCC` |
| CCCCRCCC | chr15 | + | 73293226 | 73293233 | 1.9e-05 | 0.111 | `CCCCACCC` |
| CCCCRCCC | chr16 | − | 2672252 | 2672259 | 1.9e-05 | 0.111 | `CCCCACCC` |
| CCCCRCCC | chr16 | + | 9129058 | 9129065 | 1.9e-05 | 0.111 | `CCCCACCC` |
| CCCCRCCC | chr16 | + | 11363468 | 11363475 | 1.9e-05 | 0.111 | `CCCCACCC` |
| CCCCRCCC | chr16 | − | 27151176 | 27151183 | 1.9e-05 | 0.111 | `CCCCACCC` |
| CCCCRCCC | chr16 | − | 30377770 | 30377777 | 1.9e-05 | 0.111 | `CCCCACCC` |
| CCCCRCCC | chr16 | − | 51653243 | 51653250 | 1.9e-05 | 0.111 | `CCCCACCC` |
| CCCCRCCC | chr16 | − | 55523148 | 55523155 | 1.9e-05 | 0.111 | `CCCCACCC` |
| CCCCRCCC | chr16 | + | 55523221 | 55523228 | 1.9e-05 | 0.111 | `CCCCACCC` |
| CCCCRCCC | chr16 | + | 68972464 | 68972471 | 1.9e-05 | 0.111 | `CCCCACCC` |
| CCCCRCCC | chr16 | − | 87245326 | 87245333 | 1.9e-05 | 0.111 | `CCCCACCC` |
| CCCCRCCC | chr16 | + | 87572727 | 87572734 | 1.9e-05 | 0.111 | `CCCCACCC` |
| CCCCRCCC | chr17 | + | 2561858 | 2561865 | 1.9e-05 | 0.111 | `CCCCACCC` |
| CCCCRCCC | chr17 | + | 2562201 | 2562208 | 1.9e-05 | 0.111 | `CCCCACCC` |
| CCCCRCCC | chr17 | − | 3811692 | 3811699 | 1.9e-05 | 0.111 | `CCCCACCC` |
| CCCCRCCC | chr17 | − | 4789189 | 4789196 | 1.9e-05 | 0.111 | `CCCCACCC` |
| CCCCRCCC | chr17 | − | 8139787 | 8139794 | 1.9e-05 | 0.111 | `CCCCACCC` |
| CCCCRCCC | chr17 | − | 8737522 | 8737529 | 1.9e-05 | 0.111 | `CCCCACCC` |
| CCCCRCCC | chr17 | − | 22683631 | 22683638 | 1.9e-05 | 0.111 | `CCCCACCC` |
| CCCCRCCC | chr17 | − | 33853482 | 33853489 | 1.9e-05 | 0.111 | `CCCCACCC` |
| CCCCRCCC | chr17 | − | 35992530 | 35992537 | 1.9e-05 | 0.111 | `CCCCACCC` |
| CCCCRCCC | chr17 | − | 38024634 | 38024641 | 1.9e-05 | 0.111 | `CCCCACCC` |
| CCCCRCCC | chr17 | − | 45580960 | 45580967 | 1.9e-05 | 0.111 | `CCCCACCC` |
| CCCCRCCC | chr17 | + | 59172982 | 59172989 | 1.9e-05 | 0.111 | `CCCCACCC` |
| CCCCRCCC | chr17 | + | 59274202 | 59274209 | 1.9e-05 | 0.111 | `CCCCACCC` |
| CCCCRCCC | chr17 | + | 71776113 | 71776120 | 1.9e-05 | 0.111 | `CCCCACCC` |
| CCCCRCCC | chr17 | − | 71776161 | 71776168 | 1.9e-05 | 0.111 | `CCCCACCC` |
| CCCCRCCC | chr17 | − | 71776208 | 71776215 | 1.9e-05 | 0.111 | `CCCCACCC` |
| CCCCRCCC | chr17 | + | 71779197 | 71779204 | 1.9e-05 | 0.111 | `CCCCACCC` |
| CCCCRCCC | chr17 | + | 72000341 | 72000348 | 1.9e-05 | 0.111 | `CCCCACCC` |
| CCCCRCCC | chr17 | − | 72006627 | 72006634 | 1.9e-05 | 0.111 | `CCCCACCC` |
| CCCCRCCC | chr17 | + | 72615292 | 72615299 | 1.9e-05 | 0.111 | `CCCCACCC` |
| CCCCRCCC | chr17 | + | 73637185 | 73637192 | 1.9e-05 | 0.111 | `CCCCACCC` |
| CCCCRCCC | chr17 | − | 73648094 | 73648101 | 1.9e-05 | 0.111 | `CCCCACCC` |
| CCCCRCCC | chr17 | + | 73652209 | 73652216 | 1.9e-05 | 0.111 | `CCCCACCC` |
| CCCCRCCC | chr17 | − | 73653282 | 73653289 | 1.9e-05 | 0.111 | `CCCCACCC` |
| CCCCRCCC | chr17 | + | 73681688 | 73681695 | 1.9e-05 | 0.111 | `CCCCACCC` |
| CCCCRCCC | chr17 | + | 73682686 | 73682693 | 1.9e-05 | 0.111 | `CCCCACCC` |
| CCCCRCCC | chr17 | − | 73863969 | 73863976 | 1.9e-05 | 0.111 | `CCCCACCC` |
| CCCCRCCC | chr18 | − | 18968432 | 18968439 | 1.9e-05 | 0.111 | `CCCCACCC` |
| CCCCRCCC | chr18 | − | 44733261 | 44733268 | 1.9e-05 | 0.111 | `CCCCACCC` |
| CCCCRCCC | chr18 | − | 54888571 | 54888578 | 1.9e-05 | 0.111 | `CCCCACCC` |
| CCCCRCCC | chr18 | − | 55723511 | 55723518 | 1.9e-05 | 0.111 | `CCCCACCC` |
| CCCCRCCC | chr18 | − | 58979019 | 58979026 | 1.9e-05 | 0.111 | `CCCCACCC` |
| CCCCRCCC | chr18 | + | 64811392 | 64811399 | 1.9e-05 | 0.111 | `CCCCACCC` |
| CCCCRCCC | chr18 | − | 65767283 | 65767290 | 1.9e-05 | 0.111 | `CCCCACCC` |
| CCCCRCCC | chr19 | − | 1602290 | 1602297 | 1.9e-05 | 0.111 | `CCCCACCC` |
| CCCCRCCC | chr19 | + | 4218761 | 4218768 | 1.9e-05 | 0.111 | `CCCCACCC` |
| CCCCRCCC | chr19 | − | 4218866 | 4218873 | 1.9e-05 | 0.111 | `CCCCACCC` |
| CCCCRCCC | chr19 | + | 6539658 | 6539665 | 1.9e-05 | 0.111 | `CCCCACCC` |
| CCCCRCCC | chr19 | + | 6539720 | 6539727 | 1.9e-05 | 0.111 | `CCCCACCC` |
| CCCCRCCC | chr19 | − | 6752710 | 6752717 | 1.9e-05 | 0.111 | `CCCCACCC` |
| CCCCRCCC | chr19 | − | 6752716 | 6752723 | 1.9e-05 | 0.111 | `CCCCACCC` |
| CCCCRCCC | chr19 | + | 7672749 | 7672756 | 1.9e-05 | 0.111 | `CCCCACCC` |
| CCCCRCCC | chr19 | + | 7676658 | 7676665 | 1.9e-05 | 0.111 | `CCCCACCC` |
| CCCCRCCC | chr19 | − | 8292594 | 8292601 | 1.9e-05 | 0.111 | `CCCCACCC` |
| CCCCRCCC | chr19 | + | 12755667 | 12755674 | 1.9e-05 | 0.111 | `CCCCACCC` |
| CCCCRCCC | chr19 | + | 12755792 | 12755799 | 1.9e-05 | 0.111 | `CCCCACCC` |
| CCCCRCCC | chr19 | + | 12755838 | 12755845 | 1.9e-05 | 0.111 | `CCCCACCC` |
| CCCCRCCC | chr19 | + | 12910167 | 12910174 | 1.9e-05 | 0.111 | `CCCCACCC` |
| CCCCRCCC | chr19 | + | 13127056 | 13127063 | 1.9e-05 | 0.111 | `CCCCACCC` |
| CCCCRCCC | chr19 | + | 13139584 | 13139591 | 1.9e-05 | 0.111 | `CCCCACCC` |
| CCCCRCCC | chr19 | − | 13139752 | 13139759 | 1.9e-05 | 0.111 | `CCCCACCC` |
| CCCCRCCC | chr19 | − | 16343491 | 16343498 | 1.9e-05 | 0.111 | `CCCCACCC` |
| CCCCRCCC | chr19 | + | 17097351 | 17097358 | 1.9e-05 | 0.111 | `CCCCACCC` |
| CCCCRCCC | chr19 | + | 17239128 | 17239135 | 1.9e-05 | 0.111 | `CCCCACCC` |
| CCCCRCCC | chr19 | − | 18070883 | 18070890 | 1.9e-05 | 0.111 | `CCCCACCC` |
| CCCCRCCC | chr19 | − | 44589782 | 44589789 | 1.9e-05 | 0.111 | `CCCCACCC` |
| CCCCRCCC | chr19 | − | 44590109 | 44590116 | 1.9e-05 | 0.111 | `CCCCACCC` |
| CCCCRCCC | chr19 | − | 44590170 | 44590177 | 1.9e-05 | 0.111 | `CCCCACCC` |
| CCCCRCCC | chr19 | + | 44591646 | 44591653 | 1.9e-05 | 0.111 | `CCCCACCC` |
| CCCCRCCC | chr19 | − | 47055621 | 47055628 | 1.9e-05 | 0.111 | `CCCCACCC` |
| CCCCRCCC | chr19 | − | 47080322 | 47080329 | 1.9e-05 | 0.111 | `CCCCACCC` |
| CCCCRCCC | chr19 | − | 48951082 | 48951089 | 1.9e-05 | 0.111 | `CCCCACCC` |
| CCCCRCCC | chr19 | + | 48959649 | 48959656 | 1.9e-05 | 0.111 | `CCCCACCC` |
| CCCCRCCC | chr19 | − | 50274580 | 50274587 | 1.9e-05 | 0.111 | `CCCCACCC` |
| CCCCRCCC | chr19 | + | 51913388 | 51913395 | 1.9e-05 | 0.111 | `CCCCACCC` |
| CCCCRCCC | chr19 | + | 51914448 | 51914455 | 1.9e-05 | 0.111 | `CCCCACCC` |
| CCCCRCCC | chr19 | + | 53809739 | 53809746 | 1.9e-05 | 0.111 | `CCCCACCC` |
| CCCCRCCC | chr19 | − | 54157576 | 54157583 | 1.9e-05 | 0.111 | `CCCCACCC` |
| CCCCRCCC | chr19 | − | 56766490 | 56766497 | 1.9e-05 | 0.111 | `CCCCACCC` |
| CCCCRCCC | chr19 | − | 63598613 | 63598620 | 1.9e-05 | 0.111 | `CCCCACCC` |
| CCCCRCCC | chr20 | + | 29760036 | 29760043 | 1.9e-05 | 0.111 | `CCCCACCC` |
| CCCCRCCC | chr20 | + | 30409396 | 30409403 | 1.9e-05 | 0.111 | `CCCCACCC` |
| CCCCRCCC | chr20 | − | 36904711 | 36904718 | 1.9e-05 | 0.111 | `CCCCACCC` |
| CCCCRCCC | chr20 | + | 36904752 | 36904759 | 1.9e-05 | 0.111 | `CCCCACCC` |
| CCCCRCCC | chr20 | − | 39074012 | 39074019 | 1.9e-05 | 0.111 | `CCCCACCC` |
| CCCCRCCC | chr20 | + | 44426028 | 44426035 | 1.9e-05 | 0.111 | `CCCCACCC` |
| CCCCRCCC | chr20 | + | 48981550 | 48981557 | 1.9e-05 | 0.111 | `CCCCACCC` |
| CCCCRCCC | chr20 | + | 57013479 | 57013486 | 1.9e-05 | 0.111 | `CCCCACCC` |
| CCCCRCCC | chr20 | + | 57013565 | 57013572 | 1.9e-05 | 0.111 | `CCCCACCC` |
| CCCCRCCC | chr20 | + | 62053628 | 62053635 | 1.9e-05 | 0.111 | `CCCCACCC` |
| CCCCRCCC | chr21 | − | 34270095 | 34270102 | 1.9e-05 | 0.111 | `CCCCACCC` |
| CCCCRCCC | chr21 | + | 39643231 | 39643238 | 1.9e-05 | 0.111 | `CCCCACCC` |
| CCCCRCCC | chr21 | + | 41739706 | 41739713 | 1.9e-05 | 0.111 | `CCCCACCC` |
| CCCCRCCC | chr21 | − | 41754141 | 41754148 | 1.9e-05 | 0.111 | `CCCCACCC` |
| CCCCRCCC | chr21 | − | 44451581 | 44451588 | 1.9e-05 | 0.111 | `CCCCACCC` |
| CCCCRCCC | chr22 | − | 21604042 | 21604049 | 1.9e-05 | 0.111 | `CCCCACCC` |
| CCCCRCCC | chr22 | − | 21607772 | 21607779 | 1.9e-05 | 0.111 | `CCCCACCC` |
| CCCCRCCC | chr22 | + | 21608071 | 21608078 | 1.9e-05 | 0.111 | `CCCCACCC` |
| CCCCRCCC | chr22 | + | 21608076 | 21608083 | 1.9e-05 | 0.111 | `CCCCACCC` |
| CCCCRCCC | chr22 | − | 36010326 | 36010333 | 1.9e-05 | 0.111 | `CCCCACCC` |
| CCCCRCCC | chr22 | − | 36334641 | 36334648 | 1.9e-05 | 0.111 | `CCCCACCC` |
| CCCCRCCC | chr22 | + | 39141715 | 39141722 | 1.9e-05 | 0.111 | `CCCCACCC` |
| CCCCRCCC | chr22 | + | 40139725 | 40139732 | 1.9e-05 | 0.111 | `CCCCACCC` |
| CCCCRCCC | chr22 | + | 40172803 | 40172810 | 1.9e-05 | 0.111 | `CCCCACCC` |
| CCCCRCCC | chr22 | − | 40662442 | 40662449 | 1.9e-05 | 0.111 | `CCCCACCC` |
| CCCCRCCC | chr22 | + | 41993657 | 41993664 | 1.9e-05 | 0.111 | `CCCCACCC` |
| CCCCRCCC | chr22 | − | 48052055 | 48052062 | 1.9e-05 | 0.111 | `CCCCACCC` |
| CCCCRCCC | chr22 | − | 49086362 | 49086369 | 1.9e-05 | 0.111 | `CCCCACCC` |
| CCCCRCCC | chr22 | + | 49089793 | 49089800 | 1.9e-05 | 0.111 | `CCCCACCC` |
| CCCCRCCC | chr22 | − | 49314275 | 49314282 | 1.9e-05 | 0.111 | `CCCCACCC` |
| CCCCRCCC | chr22 | − | 49314302 | 49314309 | 1.9e-05 | 0.111 | `CCCCACCC` |
| CCCCRCCC | chr22 | − | 49314966 | 49314973 | 1.9e-05 | 0.111 | `CCCCACCC` |
| CCCCRCCC | chr1 | + | 1700216 | 1700223 | 3.95e-05 | 0.117 | `CCCCGCCC` |
| CCCCRCCC | chr1 | + | 16034166 | 16034173 | 3.95e-05 | 0.117 | `CCCCGCCC` |
| CCCCRCCC | chr1 | + | 16034198 | 16034205 | 3.95e-05 | 0.117 | `CCCCGCCC` |
| CCCCRCCC | chr1 | − | 21982836 | 21982843 | 3.95e-05 | 0.117 | `CCCCGCCC` |
| CCCCRCCC | chr1 | − | 26819632 | 26819639 | 3.95e-05 | 0.117 | `CCCCGCCC` |
| CCCCRCCC | chr1 | − | 27521134 | 27521141 | 3.95e-05 | 0.117 | `CCCCGCCC` |
| CCCCRCCC | chr1 | + | 27521325 | 27521332 | 3.95e-05 | 0.117 | `CCCCGCCC` |
| CCCCRCCC | chr1 | − | 33055678 | 33055685 | 3.95e-05 | 0.117 | `CCCCGCCC` |
| CCCCRCCC | chr1 | − | 39229325 | 39229332 | 3.95e-05 | 0.117 | `CCCCGCCC` |
| CCCCRCCC | chr1 | + | 40496267 | 40496274 | 3.95e-05 | 0.117 | `CCCCGCCC` |
| CCCCRCCC | chr1 | − | 40929772 | 40929779 | 3.95e-05 | 0.117 | `CCCCGCCC` |
| CCCCRCCC | chr1 | − | 40929810 | 40929817 | 3.95e-05 | 0.117 | `CCCCGCCC` |
| CCCCRCCC | chr1 | + | 42045278 | 42045285 | 3.95e-05 | 0.117 | `CCCCGCCC` |
| CCCCRCCC | chr1 | + | 45925454 | 45925461 | 3.95e-05 | 0.117 | `CCCCGCCC` |
| CCCCRCCC | chr1 | − | 51198433 | 51198440 | 3.95e-05 | 0.117 | `CCCCGCCC` |
| CCCCRCCC | chr1 | + | 67924602 | 67924609 | 3.95e-05 | 0.117 | `CCCCGCCC` |
| CCCCRCCC | chr1 | + | 84744912 | 84744919 | 3.95e-05 | 0.117 | `CCCCGCCC` |
| CCCCRCCC | chr1 | − | 84745054 | 84745061 | 3.95e-05 | 0.117 | `CCCCGCCC` |
| CCCCRCCC | chr1 | + | 110682777 | 110682784 | 3.95e-05 | 0.117 | `CCCCGCCC` |
| CCCCRCCC | chr1 | − | 111548049 | 111548056 | 3.95e-05 | 0.117 | `CCCCGCCC` |
| CCCCRCCC | chr1 | + | 111548239 | 111548246 | 3.95e-05 | 0.117 | `CCCCGCCC` |
| CCCCRCCC | chr1 | + | 111548541 | 111548548 | 3.95e-05 | 0.117 | `CCCCGCCC` |
| CCCCRCCC | chr1 | − | 111937232 | 111937239 | 3.95e-05 | 0.117 | `CCCCGCCC` |
| CCCCRCCC | chr1 | − | 117976459 | 117976466 | 3.95e-05 | 0.117 | `CCCCGCCC` |
| CCCCRCCC | chr1 | − | 147490546 | 147490553 | 3.95e-05 | 0.117 | `CCCCGCCC` |
| CCCCRCCC | chr1 | + | 149851061 | 149851068 | 3.95e-05 | 0.117 | `CCCCGCCC` |
| CCCCRCCC | chr1 | + | 153176075 | 153176082 | 3.95e-05 | 0.117 | `CCCCGCCC` |
| CCCCRCCC | chr1 | + | 154213128 | 154213135 | 3.95e-05 | 0.117 | `CCCCGCCC` |
| CCCCRCCC | chr1 | + | 154449298 | 154449305 | 3.95e-05 | 0.117 | `CCCCGCCC` |
| CCCCRCCC | chr1 | + | 154449304 | 154449311 | 3.95e-05 | 0.117 | `CCCCGCCC` |
| CCCCRCCC | chr1 | − | 154449699 | 154449706 | 3.95e-05 | 0.117 | `CCCCGCCC` |
| CCCCRCCC | chr1 | − | 171647039 | 171647046 | 3.95e-05 | 0.117 | `CCCCGCCC` |
| CCCCRCCC | chr1 | − | 181706048 | 181706055 | 3.95e-05 | 0.117 | `CCCCGCCC` |
| CCCCRCCC | chr1 | + | 234228915 | 234228922 | 3.95e-05 | 0.117 | `CCCCGCCC` |
| CCCCRCCC | chr2 | − | 65513541 | 65513548 | 3.95e-05 | 0.117 | `CCCCGCCC` |
| CCCCRCCC | chr2 | + | 70223309 | 70223316 | 3.95e-05 | 0.117 | `CCCCGCCC` |
| CCCCRCCC | chr2 | + | 73365332 | 73365339 | 3.95e-05 | 0.117 | `CCCCGCCC` |
| CCCCRCCC | chr2 | − | 98437652 | 98437659 | 3.95e-05 | 0.117 | `CCCCGCCC` |
| CCCCRCCC | chr2 | − | 134705835 | 134705842 | 3.95e-05 | 0.117 | `CCCCGCCC` |
| CCCCRCCC | chr2 | − | 160276921 | 160276928 | 3.95e-05 | 0.117 | `CCCCGCCC` |
| CCCCRCCC | chr2 | − | 177837750 | 177837757 | 3.95e-05 | 0.117 | `CCCCGCCC` |
| CCCCRCCC | chr2 | + | 191453614 | 191453621 | 3.95e-05 | 0.117 | `CCCCGCCC` |
| CCCCRCCC | chr2 | + | 191453632 | 191453639 | 3.95e-05 | 0.117 | `CCCCGCCC` |
| CCCCRCCC | chr2 | + | 198072952 | 198072959 | 3.95e-05 | 0.117 | `CCCCGCCC` |
| CCCCRCCC | chr2 | + | 201691897 | 201691904 | 3.95e-05 | 0.117 | `CCCCGCCC` |
| CCCCRCCC | chr2 | + | 203811938 | 203811945 | 3.95e-05 | 0.117 | `CCCCGCCC` |
| CCCCRCCC | chr2 | + | 218979489 | 218979496 | 3.95e-05 | 0.117 | `CCCCGCCC` |
| CCCCRCCC | chr2 | + | 218979506 | 218979513 | 3.95e-05 | 0.117 | `CCCCGCCC` |
| CCCCRCCC | chr2 | + | 224530262 | 224530269 | 3.95e-05 | 0.117 | `CCCCGCCC` |
| CCCCRCCC | chr2 | + | 224530304 | 224530311 | 3.95e-05 | 0.117 | `CCCCGCCC` |
| CCCCRCCC | chr2 | − | 241149231 | 241149238 | 3.95e-05 | 0.117 | `CCCCGCCC` |
| CCCCRCCC | chr2 | − | 241149329 | 241149336 | 3.95e-05 | 0.117 | `CCCCGCCC` |
| CCCCRCCC | chr2 | + | 241903435 | 241903442 | 3.95e-05 | 0.117 | `CCCCGCCC` |
| CCCCRCCC | chr3 | − | 45705832 | 45705839 | 3.95e-05 | 0.117 | `CCCCGCCC` |
| CCCCRCCC | chr3 | + | 47397351 | 47397358 | 3.95e-05 | 0.117 | `CCCCGCCC` |
| CCCCRCCC | chr3 | − | 47397511 | 47397518 | 3.95e-05 | 0.117 | `CCCCGCCC` |
| CCCCRCCC | chr3 | + | 48489614 | 48489621 | 3.95e-05 | 0.117 | `CCCCGCCC` |
| CCCCRCCC | chr3 | − | 48489651 | 48489658 | 3.95e-05 | 0.117 | `CCCCGCCC` |
| CCCCRCCC | chr3 | − | 49034363 | 49034370 | 3.95e-05 | 0.117 | `CCCCGCCC` |
| CCCCRCCC | chr3 | + | 49133515 | 49133522 | 3.95e-05 | 0.117 | `CCCCGCCC` |
| CCCCRCCC | chr3 | + | 49289495 | 49289502 | 3.95e-05 | 0.117 | `CCCCGCCC` |
| CCCCRCCC | chr3 | + | 49370858 | 49370865 | 3.95e-05 | 0.117 | `CCCCGCCC` |
| CCCCRCCC | chr3 | − | 49732161 | 49732168 | 3.95e-05 | 0.117 | `CCCCGCCC` |
| CCCCRCCC | chr3 | − | 99724177 | 99724184 | 3.95e-05 | 0.117 | `CCCCGCCC` |
| CCCCRCCC | chr3 | + | 99724231 | 99724238 | 3.95e-05 | 0.117 | `CCCCGCCC` |
| CCCCRCCC | chr3 | + | 109112500 | 109112507 | 3.95e-05 | 0.117 | `CCCCGCCC` |
| CCCCRCCC | chr3 | − | 116347910 | 116347917 | 3.95e-05 | 0.117 | `CCCCGCCC` |
| CCCCRCCC | chr3 | + | 120700041 | 120700048 | 3.95e-05 | 0.117 | `CCCCGCCC` |
| CCCCRCCC | chr3 | + | 120781426 | 120781433 | 3.95e-05 | 0.117 | `CCCCGCCC` |
| CCCCRCCC | chr3 | + | 158289242 | 158289249 | 3.95e-05 | 0.117 | `CCCCGCCC` |
| CCCCRCCC | chr3 | + | 158375429 | 158375436 | 3.95e-05 | 0.117 | `CCCCGCCC` |
| CCCCRCCC | chr3 | + | 178559208 | 178559215 | 3.95e-05 | 0.117 | `CCCCGCCC` |
| CCCCRCCC | chr3 | + | 186699606 | 186699613 | 3.95e-05 | 0.117 | `CCCCGCCC` |
| CCCCRCCC | chr3 | − | 187983780 | 187983787 | 3.95e-05 | 0.117 | `CCCCGCCC` |
| CCCCRCCC | chr3 | + | 197293398 | 197293405 | 3.95e-05 | 0.117 | `CCCCGCCC` |
| CCCCRCCC | chr3 | − | 197293480 | 197293487 | 3.95e-05 | 0.117 | `CCCCGCCC` |
| CCCCRCCC | chr3 | − | 197293501 | 197293508 | 3.95e-05 | 0.117 | `CCCCGCCC` |
| CCCCRCCC | chr4 | − | 658317 | 658324 | 3.95e-05 | 0.117 | `CCCCGCCC` |
| CCCCRCCC | chr4 | − | 1684164 | 1684171 | 3.95e-05 | 0.117 | `CCCCGCCC` |
| CCCCRCCC | chr4 | − | 24923371 | 24923378 | 3.95e-05 | 0.117 | `CCCCGCCC` |
| CCCCRCCC | chr4 | + | 24923414 | 24923421 | 3.95e-05 | 0.117 | `CCCCGCCC` |
| CCCCRCCC | chr4 | + | 24923634 | 24923641 | 3.95e-05 | 0.117 | `CCCCGCCC` |
| CCCCRCCC | chr4 | + | 79002899 | 79002906 | 3.95e-05 | 0.117 | `CCCCGCCC` |
| CCCCRCCC | chr4 | − | 103968064 | 103968071 | 3.95e-05 | 0.117 | `CCCCGCCC` |
| CCCCRCCC | chr4 | + | 144654262 | 144654269 | 3.95e-05 | 0.117 | `CCCCGCCC` |
| CCCCRCCC | chr5 | − | 90712682 | 90712689 | 3.95e-05 | 0.117 | `CCCCGCCC` |
| CCCCRCCC | chr5 | + | 96296806 | 96296813 | 3.95e-05 | 0.117 | `CCCCGCCC` |
| CCCCRCCC | chr5 | + | 96297354 | 96297361 | 3.95e-05 | 0.117 | `CCCCGCCC` |
| CCCCRCCC | chr5 | + | 134268236 | 134268243 | 3.95e-05 | 0.117 | `CCCCGCCC` |
| CCCCRCCC | chr5 | − | 134268448 | 134268455 | 3.95e-05 | 0.117 | `CCCCGCCC` |
| CCCCRCCC | chr5 | − | 139907671 | 139907678 | 3.95e-05 | 0.117 | `CCCCGCCC` |
| CCCCRCCC | chr5 | + | 142763817 | 142763824 | 3.95e-05 | 0.117 | `CCCCGCCC` |
| CCCCRCCC | chr5 | + | 180582781 | 180582788 | 3.95e-05 | 0.117 | `CCCCGCCC` |
| CCCCRCCC | chr6 | + | 273587 | 273594 | 3.95e-05 | 0.117 | `CCCCGCCC` |
| CCCCRCCC | chr6 | − | 12119535 | 12119542 | 3.95e-05 | 0.117 | `CCCCGCCC` |
| CCCCRCCC | chr6 | + | 12119620 | 12119627 | 3.95e-05 | 0.117 | `CCCCGCCC` |
| CCCCRCCC | chr6 | − | 24828364 | 24828371 | 3.95e-05 | 0.117 | `CCCCGCCC` |
| CCCCRCCC | chr6 | − | 24828433 | 24828440 | 3.95e-05 | 0.117 | `CCCCGCCC` |
| CCCCRCCC | chr6 | + | 24829452 | 24829459 | 3.95e-05 | 0.117 | `CCCCGCCC` |
| CCCCRCCC | chr6 | + | 27886683 | 27886690 | 3.95e-05 | 0.117 | `CCCCGCCC` |
| CCCCRCCC | chr6 | − | 30565953 | 30565960 | 3.95e-05 | 0.117 | `CCCCGCCC` |
| CCCCRCCC | chr6 | − | 30565984 | 30565991 | 3.95e-05 | 0.117 | `CCCCGCCC` |
| CCCCRCCC | chr6 | + | 30793146 | 30793153 | 3.95e-05 | 0.117 | `CCCCGCCC` |
| CCCCRCCC | chr6 | − | 32047821 | 32047828 | 3.95e-05 | 0.117 | `CCCCGCCC` |
| CCCCRCCC | chr6 | + | 33046110 | 33046117 | 3.95e-05 | 0.117 | `CCCCGCCC` |
| CCCCRCCC | chr6 | − | 33237573 | 33237580 | 3.95e-05 | 0.117 | `CCCCGCCC` |
| CCCCRCCC | chr6 | + | 33353027 | 33353034 | 3.95e-05 | 0.117 | `CCCCGCCC` |
| CCCCRCCC | chr6 | − | 33375189 | 33375196 | 3.95e-05 | 0.117 | `CCCCGCCC` |
| CCCCRCCC | chr6 | + | 33647140 | 33647147 | 3.95e-05 | 0.117 | `CCCCGCCC` |
| CCCCRCCC | chr6 | + | 36103445 | 36103452 | 3.95e-05 | 0.117 | `CCCCGCCC` |
| CCCCRCCC | chr6 | + | 36758716 | 36758723 | 3.95e-05 | 0.117 | `CCCCGCCC` |
| CCCCRCCC | chr6 | − | 41781701 | 41781708 | 3.95e-05 | 0.117 | `CCCCGCCC` |
| CCCCRCCC | chr6 | − | 42821954 | 42821961 | 3.95e-05 | 0.117 | `CCCCGCCC` |
| CCCCRCCC | chr6 | + | 44322659 | 44322666 | 3.95e-05 | 0.117 | `CCCCGCCC` |
| CCCCRCCC | chr6 | − | 106653376 | 106653383 | 3.95e-05 | 0.117 | `CCCCGCCC` |
| CCCCRCCC | chr6 | + | 106696408 | 106696415 | 3.95e-05 | 0.117 | `CCCCGCCC` |
| CCCCRCCC | chr6 | + | 136652716 | 136652723 | 3.95e-05 | 0.117 | `CCCCGCCC` |
| CCCCRCCC | chr6 | + | 138071017 | 138071024 | 3.95e-05 | 0.117 | `CCCCGCCC` |
| CCCCRCCC | chr6 | + | 151753169 | 151753176 | 3.95e-05 | 0.117 | `CCCCGCCC` |
| CCCCRCCC | chr6 | + | 151753469 | 151753476 | 3.95e-05 | 0.117 | `CCCCGCCC` |
| CCCCRCCC | chr6 | + | 170705218 | 170705225 | 3.95e-05 | 0.117 | `CCCCGCCC` |
| CCCCRCCC | chr7 | − | 999340 | 999347 | 3.95e-05 | 0.117 | `CCCCGCCC` |
| CCCCRCCC | chr7 | + | 44802561 | 44802568 | 3.95e-05 | 0.117 | `CCCCGCCC` |
| CCCCRCCC | chr7 | − | 44802756 | 44802763 | 3.95e-05 | 0.117 | `CCCCGCCC` |
| CCCCRCCC | chr7 | + | 73263400 | 73263407 | 3.95e-05 | 0.117 | `CCCCGCCC` |
| CCCCRCCC | chr7 | + | 86687027 | 86687034 | 3.95e-05 | 0.117 | `CCCCGCCC` |
| CCCCRCCC | chr7 | + | 100310582 | 100310589 | 3.95e-05 | 0.117 | `CCCCGCCC` |
| CCCCRCCC | chr7 | + | 100310612 | 100310619 | 3.95e-05 | 0.117 | `CCCCGCCC` |
| CCCCRCCC | chr7 | + | 100675124 | 100675131 | 3.95e-05 | 0.117 | `CCCCGCCC` |
| CCCCRCCC | chr7 | + | 101287141 | 101287148 | 3.95e-05 | 0.117 | `CCCCGCCC` |
| CCCCRCCC | chr7 | − | 101737254 | 101737261 | 3.95e-05 | 0.117 | `CCCCGCCC` |
| CCCCRCCC | chr7 | − | 149733594 | 149733601 | 3.95e-05 | 0.117 | `CCCCGCCC` |
| CCCCRCCC | chr8 | − | 6553554 | 6553561 | 3.95e-05 | 0.117 | `CCCCGCCC` |
| CCCCRCCC | chr8 | + | 29443410 | 29443417 | 3.95e-05 | 0.117 | `CCCCGCCC` |
| CCCCRCCC | chr8 | + | 71476869 | 71476876 | 3.95e-05 | 0.117 | `CCCCGCCC` |
| CCCCRCCC | chr8 | + | 71476911 | 71476918 | 3.95e-05 | 0.117 | `CCCCGCCC` |
| CCCCRCCC | chr8 | − | 95634861 | 95634868 | 3.95e-05 | 0.117 | `CCCCGCCC` |
| CCCCRCCC | chr8 | + | 98725498 | 98725505 | 3.95e-05 | 0.117 | `CCCCGCCC` |
| CCCCRCCC | chr8 | + | 110415720 | 110415727 | 3.95e-05 | 0.117 | `CCCCGCCC` |
| CCCCRCCC | chr8 | − | 126689573 | 126689580 | 3.95e-05 | 0.117 | `CCCCGCCC` |
| CCCCRCCC | chr8 | − | 145988614 | 145988621 | 3.95e-05 | 0.117 | `CCCCGCCC` |
| CCCCRCCC | chr9 | − | 3516941 | 3516948 | 3.95e-05 | 0.117 | `CCCCGCCC` |
| CCCCRCCC | chr9 | + | 6403042 | 6403049 | 3.95e-05 | 0.117 | `CCCCGCCC` |
| CCCCRCCC | chr9 | + | 27563736 | 27563743 | 3.95e-05 | 0.117 | `CCCCGCCC` |
| CCCCRCCC | chr9 | − | 27563915 | 27563922 | 3.95e-05 | 0.117 | `CCCCGCCC` |
| CCCCRCCC | chr9 | − | 116483929 | 116483936 | 3.95e-05 | 0.117 | `CCCCGCCC` |
| CCCCRCCC | chr9 | + | 129253808 | 129253815 | 3.95e-05 | 0.117 | `CCCCGCCC` |
| CCCCRCCC | chr9 | − | 129581677 | 129581684 | 3.95e-05 | 0.117 | `CCCCGCCC` |
| CCCCRCCC | chr9 | − | 138959159 | 138959166 | 3.95e-05 | 0.117 | `CCCCGCCC` |
| CCCCRCCC | chrX | + | 48661290 | 48661297 | 3.95e-05 | 0.117 | `CCCCGCCC` |
| CCCCRCCC | chrX | + | 48679384 | 48679391 | 3.95e-05 | 0.117 | `CCCCGCCC` |
| CCCCRCCC | chrX | − | 54087691 | 54087698 | 3.95e-05 | 0.117 | `CCCCGCCC` |
| CCCCRCCC | chrX | + | 129053982 | 129053989 | 3.95e-05 | 0.117 | `CCCCGCCC` |
| CCCCRCCC | chr10 | − | 42598049 | 42598056 | 3.95e-05 | 0.117 | `CCCCGCCC` |
| CCCCRCCC | chr10 | − | 69957292 | 69957299 | 3.95e-05 | 0.117 | `CCCCGCCC` |
| CCCCRCCC | chr10 | − | 69957333 | 69957340 | 3.95e-05 | 0.117 | `CCCCGCCC` |
| CCCCRCCC | chr10 | + | 85889110 | 85889117 | 3.95e-05 | 0.117 | `CCCCGCCC` |
| CCCCRCCC | chr10 | + | 89612108 | 89612115 | 3.95e-05 | 0.117 | `CCCCGCCC` |
| CCCCRCCC | chr10 | − | 89612254 | 89612261 | 3.95e-05 | 0.117 | `CCCCGCCC` |
| CCCCRCCC | chr10 | + | 104144090 | 104144097 | 3.95e-05 | 0.117 | `CCCCGCCC` |
| CCCCRCCC | chr10 | − | 121239101 | 121239108 | 3.95e-05 | 0.117 | `CCCCGCCC` |
| CCCCRCCC | chr10 | − | 121239112 | 121239119 | 3.95e-05 | 0.117 | `CCCCGCCC` |
| CCCCRCCC | chr11 | + | 601408 | 601415 | 3.95e-05 | 0.117 | `CCCCGCCC` |
| CCCCRCCC | chr11 | + | 606050 | 606057 | 3.95e-05 | 0.117 | `CCCCGCCC` |
| CCCCRCCC | chr11 | + | 8660824 | 8660831 | 3.95e-05 | 0.117 | `CCCCGCCC` |
| CCCCRCCC | chr11 | + | 8660869 | 8660876 | 3.95e-05 | 0.117 | `CCCCGCCC` |
| CCCCRCCC | chr11 | − | 13255825 | 13255832 | 3.95e-05 | 0.117 | `CCCCGCCC` |
| CCCCRCCC | chr11 | − | 13441469 | 13441476 | 3.95e-05 | 0.117 | `CCCCGCCC` |
| CCCCRCCC | chr11 | − | 59335028 | 59335035 | 3.95e-05 | 0.117 | `CCCCGCCC` |
| CCCCRCCC | chr11 | + | 62146081 | 62146088 | 3.95e-05 | 0.117 | `CCCCGCCC` |
| CCCCRCCC | chr11 | − | 62189453 | 62189460 | 3.95e-05 | 0.117 | `CCCCGCCC` |
| CCCCRCCC | chr11 | − | 62329626 | 62329633 | 3.95e-05 | 0.117 | `CCCCGCCC` |
| CCCCRCCC | chr11 | + | 62329648 | 62329655 | 3.95e-05 | 0.117 | `CCCCGCCC` |
| CCCCRCCC | chr11 | − | 62379992 | 62379999 | 3.95e-05 | 0.117 | `CCCCGCCC` |
| CCCCRCCC | chr11 | + | 63750778 | 63750785 | 3.95e-05 | 0.117 | `CCCCGCCC` |
| CCCCRCCC | chr11 | − | 63794020 | 63794027 | 3.95e-05 | 0.117 | `CCCCGCCC` |
| CCCCRCCC | chr11 | + | 64402783 | 64402790 | 3.95e-05 | 0.117 | `CCCCGCCC` |
| CCCCRCCC | chr11 | + | 64402818 | 64402825 | 3.95e-05 | 0.117 | `CCCCGCCC` |
| CCCCRCCC | chr11 | + | 64402891 | 64402898 | 3.95e-05 | 0.117 | `CCCCGCCC` |
| CCCCRCCC | chr11 | + | 64641925 | 64641932 | 3.95e-05 | 0.117 | `CCCCGCCC` |
| CCCCRCCC | chr11 | + | 65021498 | 65021505 | 3.95e-05 | 0.117 | `CCCCGCCC` |
| CCCCRCCC | chr11 | − | 65021539 | 65021546 | 3.95e-05 | 0.117 | `CCCCGCCC` |
| CCCCRCCC | chr11 | + | 65021764 | 65021771 | 3.95e-05 | 0.117 | `CCCCGCCC` |
| CCCCRCCC | chr11 | + | 65098340 | 65098347 | 3.95e-05 | 0.117 | `CCCCGCCC` |
| CCCCRCCC | chr11 | − | 65098379 | 65098386 | 3.95e-05 | 0.117 | `CCCCGCCC` |
| CCCCRCCC | chr11 | − | 65098580 | 65098587 | 3.95e-05 | 0.117 | `CCCCGCCC` |
| CCCCRCCC | chr11 | + | 67796025 | 67796032 | 3.95e-05 | 0.117 | `CCCCGCCC` |
| CCCCRCCC | chr11 | − | 67796057 | 67796064 | 3.95e-05 | 0.117 | `CCCCGCCC` |
| CCCCRCCC | chr11 | + | 72531023 | 72531030 | 3.95e-05 | 0.117 | `CCCCGCCC` |
| CCCCRCCC | chr11 | − | 73168297 | 73168304 | 3.95e-05 | 0.117 | `CCCCGCCC` |
| CCCCRCCC | chr11 | − | 110723149 | 110723156 | 3.95e-05 | 0.117 | `CCCCGCCC` |
| CCCCRCCC | chr11 | − | 118302025 | 118302032 | 3.95e-05 | 0.117 | `CCCCGCCC` |
| CCCCRCCC | chr11 | − | 122438370 | 122438377 | 3.95e-05 | 0.117 | `CCCCGCCC` |
| CCCCRCCC | chr12 | + | 4122329 | 4122336 | 3.95e-05 | 0.117 | `CCCCGCCC` |
| CCCCRCCC | chr12 | − | 14409790 | 14409797 | 3.95e-05 | 0.117 | `CCCCGCCC` |
| CCCCRCCC | chr12 | + | 14409820 | 14409827 | 3.95e-05 | 0.117 | `CCCCGCCC` |
| CCCCRCCC | chr12 | − | 22588801 | 22588808 | 3.95e-05 | 0.117 | `CCCCGCCC` |
| CCCCRCCC | chr12 | − | 31940856 | 31940863 | 3.95e-05 | 0.117 | `CCCCGCCC` |
| CCCCRCCC | chr12 | + | 46493253 | 46493260 | 3.95e-05 | 0.117 | `CCCCGCCC` |
| CCCCRCCC | chr12 | − | 47810385 | 47810392 | 3.95e-05 | 0.117 | `CCCCGCCC` |
| CCCCRCCC | chr12 | − | 70344257 | 70344264 | 3.95e-05 | 0.117 | `CCCCGCCC` |
| CCCCRCCC | chr12 | − | 97421859 | 97421866 | 3.95e-05 | 0.117 | `CCCCGCCC` |
| CCCCRCCC | chr12 | + | 120811009 | 120811016 | 3.95e-05 | 0.117 | `CCCCGCCC` |
| CCCCRCCC | chr12 | + | 122025141 | 122025148 | 3.95e-05 | 0.117 | `CCCCGCCC` |
| CCCCRCCC | chr12 | − | 123967948 | 123967955 | 3.95e-05 | 0.117 | `CCCCGCCC` |
| CCCCRCCC | chr13 | − | 26922728 | 26922735 | 3.95e-05 | 0.117 | `CCCCGCCC` |
| CCCCRCCC | chr13 | + | 30089874 | 30089881 | 3.95e-05 | 0.117 | `CCCCGCCC` |
| CCCCRCCC | chr13 | + | 33014809 | 33014816 | 3.95e-05 | 0.117 | `CCCCGCCC` |
| CCCCRCCC | chr13 | + | 40243335 | 40243342 | 3.95e-05 | 0.117 | `CCCCGCCC` |
| CCCCRCCC | chr13 | − | 48005066 | 48005073 | 3.95e-05 | 0.117 | `CCCCGCCC` |
| CCCCRCCC | chr14 | + | 23734670 | 23734677 | 3.95e-05 | 0.117 | `CCCCGCCC` |
| CCCCRCCC | chr14 | + | 34942972 | 34942979 | 3.95e-05 | 0.117 | `CCCCGCCC` |
| CCCCRCCC | chr14 | + | 34943093 | 34943100 | 3.95e-05 | 0.117 | `CCCCGCCC` |
| CCCCRCCC | chr14 | − | 34943761 | 34943768 | 3.95e-05 | 0.117 | `CCCCGCCC` |
| CCCCRCCC | chr14 | + | 57834637 | 57834644 | 3.95e-05 | 0.117 | `CCCCGCCC` |
| CCCCRCCC | chr14 | − | 61105283 | 61105290 | 3.95e-05 | 0.117 | `CCCCGCCC` |
| CCCCRCCC | chr14 | + | 61287610 | 61287617 | 3.95e-05 | 0.117 | `CCCCGCCC` |
| CCCCRCCC | chr14 | + | 89932864 | 89932871 | 3.95e-05 | 0.117 | `CCCCGCCC` |
| CCCCRCCC | chr15 | − | 29295096 | 29295103 | 3.95e-05 | 0.117 | `CCCCGCCC` |
| CCCCRCCC | chr15 | + | 29295991 | 29295998 | 3.95e-05 | 0.117 | `CCCCGCCC` |
| CCCCRCCC | chr15 | + | 29345926 | 29345933 | 3.95e-05 | 0.117 | `CCCCGCCC` |
| CCCCRCCC | chr15 | + | 29345931 | 29345938 | 3.95e-05 | 0.117 | `CCCCGCCC` |
| CCCCRCCC | chr15 | + | 38013556 | 38013563 | 3.95e-05 | 0.117 | `CCCCGCCC` |
| CCCCRCCC | chr15 | + | 38013617 | 38013624 | 3.95e-05 | 0.117 | `CCCCGCCC` |
| CCCCRCCC | chr15 | + | 43280031 | 43280038 | 3.95e-05 | 0.117 | `CCCCGCCC` |
| CCCCRCCC | chr15 | + | 70310647 | 70310654 | 3.95e-05 | 0.117 | `CCCCGCCC` |
| CCCCRCCC | chr15 | + | 70310686 | 70310693 | 3.95e-05 | 0.117 | `CCCCGCCC` |
| CCCCRCCC | chr15 | + | 70311040 | 70311047 | 3.95e-05 | 0.117 | `CCCCGCCC` |
| CCCCRCCC | chr15 | − | 70554738 | 70554745 | 3.95e-05 | 0.117 | `CCCCGCCC` |
| CCCCRCCC | chr15 | + | 73293220 | 73293227 | 3.95e-05 | 0.117 | `CCCCGCCC` |
| CCCCRCCC | chr15 | + | 91154156 | 91154163 | 3.95e-05 | 0.117 | `CCCCGCCC` |
| CCCCRCCC | chr15 | − | 91248639 | 91248646 | 3.95e-05 | 0.117 | `CCCCGCCC` |
| CCCCRCCC | chr16 | + | 1954888 | 1954895 | 3.95e-05 | 0.117 | `CCCCGCCC` |
| CCCCRCCC | chr16 | + | 1954950 | 1954957 | 3.95e-05 | 0.117 | `CCCCGCCC` |
| CCCCRCCC | chr16 | + | 2195360 | 2195367 | 3.95e-05 | 0.117 | `CCCCGCCC` |
| CCCCRCCC | chr16 | + | 2195394 | 2195401 | 3.95e-05 | 0.117 | `CCCCGCCC` |
| CCCCRCCC | chr16 | + | 2521994 | 2522001 | 3.95e-05 | 0.117 | `CCCCGCCC` |
| CCCCRCCC | chr16 | + | 8937661 | 8937668 | 3.95e-05 | 0.117 | `CCCCGCCC` |
| CCCCRCCC | chr16 | + | 11744310 | 11744317 | 3.95e-05 | 0.117 | `CCCCGCCC` |
| CCCCRCCC | chr16 | + | 11744363 | 11744370 | 3.95e-05 | 0.117 | `CCCCGCCC` |
| CCCCRCCC | chr16 | + | 14287139 | 14287146 | 3.95e-05 | 0.117 | `CCCCGCCC` |
| CCCCRCCC | chr16 | + | 18720596 | 18720603 | 3.95e-05 | 0.117 | `CCCCGCCC` |
| CCCCRCCC | chr16 | − | 18720776 | 18720783 | 3.95e-05 | 0.117 | `CCCCGCCC` |
| CCCCRCCC | chr16 | − | 22216120 | 22216127 | 3.95e-05 | 0.117 | `CCCCGCCC` |
| CCCCRCCC | chr16 | − | 22216179 | 22216186 | 3.95e-05 | 0.117 | `CCCCGCCC` |
| CCCCRCCC | chr16 | − | 27320948 | 27320955 | 3.95e-05 | 0.117 | `CCCCGCCC` |
| CCCCRCCC | chr16 | + | 30841578 | 30841585 | 3.95e-05 | 0.117 | `CCCCGCCC` |
| CCCCRCCC | chr16 | − | 51685686 | 51685693 | 3.95e-05 | 0.117 | `CCCCGCCC` |
| CCCCRCCC | chr16 | − | 66464362 | 66464369 | 3.95e-05 | 0.117 | `CCCCGCCC` |
| CCCCRCCC | chr16 | − | 66464641 | 66464648 | 3.95e-05 | 0.117 | `CCCCGCCC` |
| CCCCRCCC | chr16 | − | 66527114 | 66527121 | 3.95e-05 | 0.117 | `CCCCGCCC` |
| CCCCRCCC | chr16 | + | 66527179 | 66527186 | 3.95e-05 | 0.117 | `CCCCGCCC` |
| CCCCRCCC | chr16 | + | 85974968 | 85974975 | 3.95e-05 | 0.117 | `CCCCGCCC` |
| CCCCRCCC | chr16 | − | 86542496 | 86542503 | 3.95e-05 | 0.117 | `CCCCGCCC` |
| CCCCRCCC | chr16 | − | 88158951 | 88158958 | 3.95e-05 | 0.117 | `CCCCGCCC` |
| CCCCRCCC | chr17 | + | 2561923 | 2561930 | 3.95e-05 | 0.117 | `CCCCGCCC` |
| CCCCRCCC | chr17 | − | 2562010 | 2562017 | 3.95e-05 | 0.117 | `CCCCGCCC` |
| CCCCRCCC | chr17 | + | 5283199 | 5283206 | 3.95e-05 | 0.117 | `CCCCGCCC` |
| CCCCRCCC | chr17 | + | 7328027 | 7328034 | 3.95e-05 | 0.117 | `CCCCGCCC` |
| CCCCRCCC | chr17 | − | 7328357 | 7328364 | 3.95e-05 | 0.117 | `CCCCGCCC` |
| CCCCRCCC | chr17 | + | 30724370 | 30724377 | 3.95e-05 | 0.117 | `CCCCGCCC` |
| CCCCRCCC | chr17 | + | 30724405 | 30724412 | 3.95e-05 | 0.117 | `CCCCGCCC` |
| CCCCRCCC | chr17 | + | 30724410 | 30724417 | 3.95e-05 | 0.117 | `CCCCGCCC` |
| CCCCRCCC | chr17 | + | 30929613 | 30929620 | 3.95e-05 | 0.117 | `CCCCGCCC` |
| CCCCRCCC | chr17 | − | 33853673 | 33853680 | 3.95e-05 | 0.117 | `CCCCGCCC` |
| CCCCRCCC | chr17 | − | 35164342 | 35164349 | 3.95e-05 | 0.117 | `CCCCGCCC` |
| CCCCRCCC | chr17 | − | 35164347 | 35164354 | 3.95e-05 | 0.117 | `CCCCGCCC` |
| CCCCRCCC | chr17 | − | 35273709 | 35273716 | 3.95e-05 | 0.117 | `CCCCGCCC` |
| CCCCRCCC | chr17 | + | 35963741 | 35963748 | 3.95e-05 | 0.117 | `CCCCGCCC` |
| CCCCRCCC | chr17 | − | 40581574 | 40581581 | 3.95e-05 | 0.117 | `CCCCGCCC` |
| CCCCRCCC | chr17 | + | 40594718 | 40594725 | 3.95e-05 | 0.117 | `CCCCGCCC` |
| CCCCRCCC | chr17 | − | 44625237 | 44625244 | 3.95e-05 | 0.117 | `CCCCGCCC` |
| CCCCRCCC | chr17 | − | 44625376 | 44625383 | 3.95e-05 | 0.117 | `CCCCGCCC` |
| CCCCRCCC | chr17 | + | 44650522 | 44650529 | 3.95e-05 | 0.117 | `CCCCGCCC` |
| CCCCRCCC | chr17 | + | 44656557 | 44656564 | 3.95e-05 | 0.117 | `CCCCGCCC` |
| CCCCRCCC | chr17 | + | 59172987 | 59172994 | 3.95e-05 | 0.117 | `CCCCGCCC` |
| CCCCRCCC | chr17 | + | 59274073 | 59274080 | 3.95e-05 | 0.117 | `CCCCGCCC` |
| CCCCRCCC | chr17 | + | 59932957 | 59932964 | 3.95e-05 | 0.117 | `CCCCGCCC` |
| CCCCRCCC | chr17 | + | 59933128 | 59933135 | 3.95e-05 | 0.117 | `CCCCGCCC` |
| CCCCRCCC | chr17 | + | 59933150 | 59933157 | 3.95e-05 | 0.117 | `CCCCGCCC` |
| CCCCRCCC | chr17 | + | 60402077 | 60402084 | 3.95e-05 | 0.117 | `CCCCGCCC` |
| CCCCRCCC | chr17 | + | 71861724 | 71861731 | 3.95e-05 | 0.117 | `CCCCGCCC` |
| CCCCRCCC | chr17 | − | 71861947 | 71861954 | 3.95e-05 | 0.117 | `CCCCGCCC` |
| CCCCRCCC | chr17 | − | 73636881 | 73636888 | 3.95e-05 | 0.117 | `CCCCGCCC` |
| CCCCRCCC | chr17 | − | 73648212 | 73648219 | 3.95e-05 | 0.117 | `CCCCGCCC` |
| CCCCRCCC | chr17 | − | 73648240 | 73648247 | 3.95e-05 | 0.117 | `CCCCGCCC` |
| CCCCRCCC | chr17 | − | 73648378 | 73648385 | 3.95e-05 | 0.117 | `CCCCGCCC` |
| CCCCRCCC | chr17 | − | 77779508 | 77779515 | 3.95e-05 | 0.117 | `CCCCGCCC` |
| CCCCRCCC | chr18 | − | 12410500 | 12410507 | 3.95e-05 | 0.117 | `CCCCGCCC` |
| CCCCRCCC | chr18 | − | 58978980 | 58978987 | 3.95e-05 | 0.117 | `CCCCGCCC` |
| CCCCRCCC | chr19 | + | 1046414 | 1046421 | 3.95e-05 | 0.117 | `CCCCGCCC` |
| CCCCRCCC | chr19 | + | 2691055 | 2691062 | 3.95e-05 | 0.117 | `CCCCGCCC` |
| CCCCRCCC | chr19 | + | 6542322 | 6542329 | 3.95e-05 | 0.117 | `CCCCGCCC` |
| CCCCRCCC | chr19 | + | 7672755 | 7672762 | 3.95e-05 | 0.117 | `CCCCGCCC` |
| CCCCRCCC | chr19 | + | 10900149 | 10900156 | 3.95e-05 | 0.117 | `CCCCGCCC` |
| CCCCRCCC | chr19 | + | 12754525 | 12754532 | 3.95e-05 | 0.117 | `CCCCGCCC` |
| CCCCRCCC | chr19 | + | 12755785 | 12755792 | 3.95e-05 | 0.117 | `CCCCGCCC` |
| CCCCRCCC | chr19 | − | 12765159 | 12765166 | 3.95e-05 | 0.117 | `CCCCGCCC` |
| CCCCRCCC | chr19 | + | 12765399 | 12765406 | 3.95e-05 | 0.117 | `CCCCGCCC` |
| CCCCRCCC | chr19 | − | 12910113 | 12910120 | 3.95e-05 | 0.117 | `CCCCGCCC` |
| CCCCRCCC | chr19 | + | 12917524 | 12917531 | 3.95e-05 | 0.117 | `CCCCGCCC` |
| CCCCRCCC | chr19 | + | 13122076 | 13122083 | 3.95e-05 | 0.117 | `CCCCGCCC` |
| CCCCRCCC | chr19 | + | 13122214 | 13122221 | 3.95e-05 | 0.117 | `CCCCGCCC` |
| CCCCRCCC | chr19 | − | 13126759 | 13126766 | 3.95e-05 | 0.117 | `CCCCGCCC` |
| CCCCRCCC | chr19 | + | 14352940 | 14352947 | 3.95e-05 | 0.117 | `CCCCGCCC` |
| CCCCRCCC | chr19 | + | 15351801 | 15351808 | 3.95e-05 | 0.117 | `CCCCGCCC` |
| CCCCRCCC | chr19 | + | 15351818 | 15351825 | 3.95e-05 | 0.117 | `CCCCGCCC` |
| CCCCRCCC | chr19 | + | 15351823 | 15351830 | 3.95e-05 | 0.117 | `CCCCGCCC` |
| CCCCRCCC | chr19 | + | 17081990 | 17081997 | 3.95e-05 | 0.117 | `CCCCGCCC` |
| CCCCRCCC | chr19 | + | 17904350 | 17904357 | 3.95e-05 | 0.117 | `CCCCGCCC` |
| CCCCRCCC | chr19 | + | 44585331 | 44585338 | 3.95e-05 | 0.117 | `CCCCGCCC` |
| CCCCRCCC | chr19 | + | 44586450 | 44586457 | 3.95e-05 | 0.117 | `CCCCGCCC` |
| CCCCRCCC | chr19 | + | 45623801 | 45623808 | 3.95e-05 | 0.117 | `CCCCGCCC` |
| CCCCRCCC | chr19 | − | 45996588 | 45996595 | 3.95e-05 | 0.117 | `CCCCGCCC` |
| CCCCRCCC | chr19 | + | 45996636 | 45996643 | 3.95e-05 | 0.117 | `CCCCGCCC` |
| CCCCRCCC | chr19 | − | 45996730 | 45996737 | 3.95e-05 | 0.117 | `CCCCGCCC` |
| CCCCRCCC | chr19 | + | 48950467 | 48950474 | 3.95e-05 | 0.117 | `CCCCGCCC` |
| CCCCRCCC | chr19 | + | 49500845 | 49500852 | 3.95e-05 | 0.117 | `CCCCGCCC` |
| CCCCRCCC | chr19 | + | 50270618 | 50270625 | 3.95e-05 | 0.117 | `CCCCGCCC` |
| CCCCRCCC | chr19 | + | 51058314 | 51058321 | 3.95e-05 | 0.117 | `CCCCGCCC` |
| CCCCRCCC | chr19 | − | 51081642 | 51081649 | 3.95e-05 | 0.117 | `CCCCGCCC` |
| CCCCRCCC | chr19 | + | 53809734 | 53809741 | 3.95e-05 | 0.117 | `CCCCGCCC` |
| CCCCRCCC | chr19 | − | 53814263 | 53814270 | 3.95e-05 | 0.117 | `CCCCGCCC` |
| CCCCRCCC | chr19 | + | 54070613 | 54070620 | 3.95e-05 | 0.117 | `CCCCGCCC` |
| CCCCRCCC | chr19 | + | 54691410 | 54691417 | 3.95e-05 | 0.117 | `CCCCGCCC` |
| CCCCRCCC | chr19 | + | 55528502 | 55528509 | 3.95e-05 | 0.117 | `CCCCGCCC` |
| CCCCRCCC | chr19 | − | 55571535 | 55571542 | 3.95e-05 | 0.117 | `CCCCGCCC` |
| CCCCRCCC | chr19 | − | 60462263 | 60462270 | 3.95e-05 | 0.117 | `CCCCGCCC` |
| CCCCRCCC | chr19 | − | 60802880 | 60802887 | 3.95e-05 | 0.117 | `CCCCGCCC` |
| CCCCRCCC | chr19 | − | 63598719 | 63598726 | 3.95e-05 | 0.117 | `CCCCGCCC` |
| CCCCRCCC | chr19 | + | 63611688 | 63611695 | 3.95e-05 | 0.117 | `CCCCGCCC` |
| CCCCRCCC | chr20 | − | 3749315 | 3749322 | 3.95e-05 | 0.117 | `CCCCGCCC` |
| CCCCRCCC | chr20 | + | 5934545 | 5934552 | 3.95e-05 | 0.117 | `CCCCGCCC` |
| CCCCRCCC | chr20 | + | 29760117 | 29760124 | 3.95e-05 | 0.117 | `CCCCGCCC` |
| CCCCRCCC | chr20 | − | 48560084 | 48560091 | 3.95e-05 | 0.117 | `CCCCGCCC` |
| CCCCRCCC | chr20 | + | 62053476 | 62053483 | 3.95e-05 | 0.117 | `CCCCGCCC` |
| CCCCRCCC | chr20 | + | 62053518 | 62053525 | 3.95e-05 | 0.117 | `CCCCGCCC` |
| CCCCRCCC | chr20 | + | 62053560 | 62053567 | 3.95e-05 | 0.117 | `CCCCGCCC` |
| CCCCRCCC | chr20 | + | 62053602 | 62053609 | 3.95e-05 | 0.117 | `CCCCGCCC` |
| CCCCRCCC | chr21 | + | 46530651 | 46530658 | 3.95e-05 | 0.117 | `CCCCGCCC` |
| CCCCRCCC | chr21 | − | 33836773 | 33836780 | 3.95e-05 | 0.117 | `CCCCGCCC` |
| CCCCRCCC | chr22 | − | 17799854 | 17799861 | 3.95e-05 | 0.117 | `CCCCGCCC` |
| CCCCRCCC | chr22 | − | 17799943 | 17799950 | 3.95e-05 | 0.117 | `CCCCGCCC` |
| CCCCRCCC | chr22 | + | 38045617 | 38045624 | 3.95e-05 | 0.117 | `CCCCGCCC` |
| CCCCRCCC | chr22 | + | 38246573 | 38246580 | 3.95e-05 | 0.117 | `CCCCGCCC` |
| CCCCRCCC | chr22 | − | 39363573 | 39363580 | 3.95e-05 | 0.117 | `CCCCGCCC` |
| CCCCRCCC | chr22 | − | 40172839 | 40172846 | 3.95e-05 | 0.117 | `CCCCGCCC` |
| CCCCRCCC | chr22 | − | 40558642 | 40558649 | 3.95e-05 | 0.117 | `CCCCGCCC` |
| CCCCRCCC | chr22 | − | 48052261 | 48052268 | 3.95e-05 | 0.117 | `CCCCGCCC` |
| CCCCRCCC | chr22 | + | 49086509 | 49086516 | 3.95e-05 | 0.117 | `CCCCGCCC` |
| CCCCRCCC | chr22 | − | 49311475 | 49311482 | 3.95e-05 | 0.117 | `CCCCGCCC` |
| CCCCRCCC | chr22 | − | 49311776 | 49311783 | 3.95e-05 | 0.117 | `CCCCGCCC` |
| CCCCRCCC | chr22 | + | 49315187 | 49315194 | 3.95e-05 | 0.117 | `CCCCGCCC` |
| CCCCRCCC | chr1 | + | 3583543 | 3583550 | 7.9e-05 | 0.145 | `CCCCTCCC` |
| CCCCRCCC | chr1 | + | 12034749 | 12034756 | 7.9e-05 | 0.145 | `CCCCTCCC` |
| CCCCRCCC | chr1 | − | 12061671 | 12061678 | 7.9e-05 | 0.145 | `CCCCTCCC` |
| CCCCRCCC | chr1 | − | 12061676 | 12061683 | 7.9e-05 | 0.145 | `CCCCTCCC` |
| CCCCRCCC | chr1 | + | 21489667 | 21489674 | 7.9e-05 | 0.145 | `CCCCTCCC` |
| CCCCRCCC | chr1 | − | 21492949 | 21492956 | 7.9e-05 | 0.145 | `CCCCTCCC` |
| CCCCRCCC | chr1 | + | 26819687 | 26819694 | 7.9e-05 | 0.145 | `CCCCTCCC` |
| CCCCRCCC | chr1 | + | 26892507 | 26892514 | 7.9e-05 | 0.145 | `CCCCTCCC` |
| CCCCRCCC | chr1 | − | 27824234 | 27824241 | 7.9e-05 | 0.145 | `CCCCTCCC` |
| CCCCRCCC | chr1 | − | 28083400 | 28083407 | 7.9e-05 | 0.145 | `CCCCTCCC` |
| CCCCRCCC | chr1 | − | 29957690 | 29957697 | 7.9e-05 | 0.145 | `CCCCTCCC` |
| CCCCRCCC | chr1 | + | 32488371 | 32488378 | 7.9e-05 | 0.145 | `CCCCTCCC` |
| CCCCRCCC | chr1 | − | 33055801 | 33055808 | 7.9e-05 | 0.145 | `CCCCTCCC` |
| CCCCRCCC | chr1 | + | 39229232 | 39229239 | 7.9e-05 | 0.145 | `CCCCTCCC` |
| CCCCRCCC | chr1 | − | 39229320 | 39229327 | 7.9e-05 | 0.145 | `CCCCTCCC` |
| CCCCRCCC | chr1 | − | 40929934 | 40929941 | 7.9e-05 | 0.145 | `CCCCTCCC` |
| CCCCRCCC | chr1 | + | 40930035 | 40930042 | 7.9e-05 | 0.145 | `CCCCTCCC` |
| CCCCRCCC | chr1 | − | 59206974 | 59206981 | 7.9e-05 | 0.145 | `CCCCTCCC` |
| CCCCRCCC | chr1 | − | 67913035 | 67913042 | 7.9e-05 | 0.145 | `CCCCTCCC` |
| CCCCRCCC | chr1 | − | 67923568 | 67923575 | 7.9e-05 | 0.145 | `CCCCTCCC` |
| CCCCRCCC | chr1 | − | 93070450 | 93070457 | 7.9e-05 | 0.145 | `CCCCTCCC` |
| CCCCRCCC | chr1 | + | 111936961 | 111936968 | 7.9e-05 | 0.145 | `CCCCTCCC` |
| CCCCRCCC | chr1 | + | 114249183 | 114249190 | 7.9e-05 | 0.145 | `CCCCTCCC` |
| CCCCRCCC | chr1 | − | 117976538 | 117976545 | 7.9e-05 | 0.145 | `CCCCTCCC` |
| CCCCRCCC | chr1 | − | 118002858 | 118002865 | 7.9e-05 | 0.145 | `CCCCTCCC` |
| CCCCRCCC | chr1 | − | 144094025 | 144094032 | 7.9e-05 | 0.145 | `CCCCTCCC` |
| CCCCRCCC | chr1 | − | 148398701 | 148398708 | 7.9e-05 | 0.145 | `CCCCTCCC` |
| CCCCRCCC | chr1 | + | 148806955 | 148806962 | 7.9e-05 | 0.145 | `CCCCTCCC` |
| CCCCRCCC | chr1 | + | 148818477 | 148818484 | 7.9e-05 | 0.145 | `CCCCTCCC` |
| CCCCRCCC | chr1 | − | 149850871 | 149850878 | 7.9e-05 | 0.145 | `CCCCCCCC` |
| CCCCRCCC | chr1 | − | 149850872 | 149850879 | 7.9e-05 | 0.145 | `CCCCCCCC` |
| CCCCRCCC | chr1 | − | 149850873 | 149850880 | 7.9e-05 | 0.145 | `CCCCCCCC` |
| CCCCRCCC | chr1 | − | 154216062 | 154216069 | 7.9e-05 | 0.145 | `CCCCTCCC` |
| CCCCRCCC | chr1 | + | 154741554 | 154741561 | 7.9e-05 | 0.145 | `CCCCCCCC` |
| CCCCRCCC | chr1 | + | 154741570 | 154741577 | 7.9e-05 | 0.145 | `CCCCTCCC` |
| CCCCRCCC | chr1 | + | 154977518 | 154977525 | 7.9e-05 | 0.145 | `CCCCTCCC` |
| CCCCRCCC | chr1 | − | 158910919 | 158910926 | 7.9e-05 | 0.145 | `CCCCTCCC` |
| CCCCRCCC | chr1 | + | 176817390 | 176817397 | 7.9e-05 | 0.145 | `CCCCTCCC` |
| CCCCRCCC | chr1 | − | 179370785 | 179370792 | 7.9e-05 | 0.145 | `CCCCTCCC` |
| CCCCRCCC | chr1 | − | 181707664 | 181707671 | 7.9e-05 | 0.145 | `CCCCTCCC` |
| CCCCRCCC | chr1 | − | 191357644 | 191357651 | 7.9e-05 | 0.145 | `CCCCTCCC` |
| CCCCRCCC | chr1 | − | 191357943 | 191357950 | 7.9e-05 | 0.145 | `CCCCCCCC` |
| CCCCRCCC | chr1 | − | 201543868 | 201543875 | 7.9e-05 | 0.145 | `CCCCCCCC` |
| CCCCRCCC | chr1 | + | 201544178 | 201544185 | 7.9e-05 | 0.145 | `CCCCTCCC` |
| CCCCRCCC | chr1 | + | 201556710 | 201556717 | 7.9e-05 | 0.145 | `CCCCTCCC` |
| CCCCRCCC | chr1 | − | 205170441 | 205170448 | 7.9e-05 | 0.145 | `CCCCTCCC` |
| CCCCRCCC | chr1 | − | 232725007 | 232725014 | 7.9e-05 | 0.145 | `CCCCTCCC` |
| CCCCRCCC | chr2 | + | 25048167 | 25048174 | 7.9e-05 | 0.145 | `CCCCTCCC` |
| CCCCRCCC | chr2 | − | 33556953 | 33556960 | 7.9e-05 | 0.145 | `CCCCTCCC` |
| CCCCRCCC | chr2 | − | 42183029 | 42183036 | 7.9e-05 | 0.145 | `CCCCTCCC` |
| CCCCRCCC | chr2 | + | 54650613 | 54650620 | 7.9e-05 | 0.145 | `CCCCCCCC` |
| CCCCRCCC | chr2 | + | 54650614 | 54650621 | 7.9e-05 | 0.145 | `CCCCCCCC` |
| CCCCRCCC | chr2 | + | 54650615 | 54650622 | 7.9e-05 | 0.145 | `CCCCCCCC` |
| CCCCRCCC | chr2 | + | 65513326 | 65513333 | 7.9e-05 | 0.145 | `CCCCTCCC` |
| CCCCRCCC | chr2 | + | 65513498 | 65513505 | 7.9e-05 | 0.145 | `CCCCTCCC` |
| CCCCRCCC | chr2 | + | 70167639 | 70167646 | 7.9e-05 | 0.145 | `CCCCTCCC` |
| CCCCRCCC | chr2 | + | 70167666 | 70167673 | 7.9e-05 | 0.145 | `CCCCTCCC` |
| CCCCRCCC | chr2 | − | 70170973 | 70170980 | 7.9e-05 | 0.145 | `CCCCTCCC` |
| CCCCRCCC | chr2 | + | 70223204 | 70223211 | 7.9e-05 | 0.145 | `CCCCTCCC` |
| CCCCRCCC | chr2 | − | 111649832 | 111649839 | 7.9e-05 | 0.145 | `CCCCTCCC` |
| CCCCRCCC | chr2 | − | 136856471 | 136856478 | 7.9e-05 | 0.145 | `CCCCTCCC` |
| CCCCRCCC | chr2 | − | 160277237 | 160277244 | 7.9e-05 | 0.145 | `CCCCTCCC` |
| CCCCRCCC | chr2 | − | 177838171 | 177838178 | 7.9e-05 | 0.145 | `CCCCCCCC` |
| CCCCRCCC | chr2 | + | 198072846 | 198072853 | 7.9e-05 | 0.145 | `CCCCTCCC` |
| CCCCRCCC | chr2 | + | 198072957 | 198072964 | 7.9e-05 | 0.145 | `CCCCTCCC` |
| CCCCRCCC | chr2 | − | 198073100 | 198073107 | 7.9e-05 | 0.145 | `CCCCTCCC` |
| CCCCRCCC | chr2 | − | 198073261 | 198073268 | 7.9e-05 | 0.145 | `CCCCTCCC` |
| CCCCRCCC | chr2 | + | 208343970 | 208343977 | 7.9e-05 | 0.145 | `CCCCTCCC` |
| CCCCRCCC | chr2 | + | 231447005 | 231447012 | 7.9e-05 | 0.145 | `CCCCTCCC` |
| CCCCRCCC | chr2 | − | 231558422 | 231558429 | 7.9e-05 | 0.145 | `CCCCCCCC` |
| CCCCRCCC | chr2 | − | 231558423 | 231558430 | 7.9e-05 | 0.145 | `CCCCCCCC` |
| CCCCRCCC | chr2 | − | 231558424 | 231558431 | 7.9e-05 | 0.145 | `CCCCCCCC` |
| CCCCRCCC | chr2 | + | 232281236 | 232281243 | 7.9e-05 | 0.145 | `CCCCTCCC` |
| CCCCRCCC | chr2 | − | 233632764 | 233632771 | 7.9e-05 | 0.145 | `CCCCTCCC` |
| CCCCRCCC | chr2 | + | 233632786 | 233632793 | 7.9e-05 | 0.145 | `CCCCTCCC` |
| CCCCRCCC | chr2 | − | 241149268 | 241149275 | 7.9e-05 | 0.145 | `CCCCTCCC` |
| CCCCRCCC | chr2 | + | 241903513 | 241903520 | 7.9e-05 | 0.145 | `CCCCTCCC` |
| CCCCRCCC | chr2 | + | 241903766 | 241903773 | 7.9e-05 | 0.145 | `CCCCCCCC` |
| CCCCRCCC | chr3 | + | 9413089 | 9413096 | 7.9e-05 | 0.145 | `CCCCTCCC` |
| CCCCRCCC | chr3 | + | 9413625 | 9413632 | 7.9e-05 | 0.145 | `CCCCTCCC` |
| CCCCRCCC | chr3 | + | 10209910 | 10209917 | 7.9e-05 | 0.145 | `CCCCTCCC` |
| CCCCRCCC | chr3 | + | 13032047 | 13032054 | 7.9e-05 | 0.145 | `CCCCTCCC` |
| CCCCRCCC | chr3 | + | 13104575 | 13104582 | 7.9e-05 | 0.145 | `CCCCTCCC` |
| CCCCRCCC | chr3 | + | 17198907 | 17198914 | 7.9e-05 | 0.145 | `CCCCTCCC` |
| CCCCRCCC | chr3 | + | 45571142 | 45571149 | 7.9e-05 | 0.145 | `CCCCTCCC` |
| CCCCRCCC | chr3 | − | 73105052 | 73105059 | 7.9e-05 | 0.145 | `CCCCTCCC` |
| CCCCRCCC | chr3 | − | 113763671 | 113763678 | 7.9e-05 | 0.145 | `CCCCTCCC` |
| CCCCRCCC | chr3 | + | 116349153 | 116349160 | 7.9e-05 | 0.145 | `CCCCTCCC` |
| CCCCRCCC | chr3 | + | 120554134 | 120554141 | 7.9e-05 | 0.145 | `CCCCTCCC` |
| CCCCRCCC | chr3 | + | 120781420 | 120781427 | 7.9e-05 | 0.145 | `CCCCTCCC` |
| CCCCRCCC | chr3 | + | 178560444 | 178560451 | 7.9e-05 | 0.145 | `CCCCTCCC` |
| CCCCRCCC | chr3 | − | 178560687 | 178560694 | 7.9e-05 | 0.145 | `CCCCTCCC` |
| CCCCRCCC | chr3 | − | 179737990 | 179737997 | 7.9e-05 | 0.145 | `CCCCTCCC` |
| CCCCRCCC | chr3 | + | 185085511 | 185085518 | 7.9e-05 | 0.145 | `CCCCTCCC` |
| CCCCRCCC | chr3 | − | 186699549 | 186699556 | 7.9e-05 | 0.145 | `CCCCTCCC` |
| CCCCRCCC | chr3 | + | 187138000 | 187138007 | 7.9e-05 | 0.145 | `CCCCTCCC` |
| CCCCRCCC | chr4 | − | 7529749 | 7529756 | 7.9e-05 | 0.145 | `CCCCTCCC` |
| CCCCRCCC | chr4 | − | 39924093 | 39924100 | 7.9e-05 | 0.145 | `CCCCTCCC` |
| CCCCRCCC | chr4 | + | 39998609 | 39998616 | 7.9e-05 | 0.145 | `CCCCTCCC` |
| CCCCRCCC | chr4 | + | 103967865 | 103967872 | 7.9e-05 | 0.145 | `CCCCTCCC` |
| CCCCRCCC | chr4 | + | 103968207 | 103968214 | 7.9e-05 | 0.145 | `CCCCTCCC` |
| CCCCRCCC | chr4 | − | 185973638 | 185973645 | 7.9e-05 | 0.145 | `CCCCCCCC` |
| CCCCRCCC | chr5 | + | 56001573 | 56001580 | 7.9e-05 | 0.145 | `CCCCCCCC` |
| CCCCRCCC | chr5 | + | 81082786 | 81082793 | 7.9e-05 | 0.145 | `CCCCTCCC` |
| CCCCRCCC | chr5 | + | 81083010 | 81083017 | 7.9e-05 | 0.145 | `CCCCTCCC` |
| CCCCRCCC | chr5 | − | 134268048 | 134268055 | 7.9e-05 | 0.145 | `CCCCTCCC` |
| CCCCRCCC | chr5 | + | 138753566 | 138753573 | 7.9e-05 | 0.145 | `CCCCTCCC` |
| CCCCRCCC | chr5 | − | 139205293 | 139205300 | 7.9e-05 | 0.145 | `CCCCTCCC` |
| CCCCRCCC | chr5 | − | 139908174 | 139908181 | 7.9e-05 | 0.145 | `CCCCTCCC` |
| CCCCRCCC | chr5 | + | 145542322 | 145542329 | 7.9e-05 | 0.145 | `CCCCTCCC` |
| CCCCRCCC | chr5 | − | 149764245 | 149764252 | 7.9e-05 | 0.145 | `CCCCTCCC` |
| CCCCRCCC | chr5 | − | 149765083 | 149765090 | 7.9e-05 | 0.145 | `CCCCTCCC` |
| CCCCRCCC | chr5 | − | 149765088 | 149765095 | 7.9e-05 | 0.145 | `CCCCTCCC` |
| CCCCRCCC | chr5 | + | 149766535 | 149766542 | 7.9e-05 | 0.145 | `CCCCTCCC` |
| CCCCRCCC | chr5 | + | 149766563 | 149766570 | 7.9e-05 | 0.145 | `CCCCTCCC` |
| CCCCRCCC | chr5 | + | 149766607 | 149766614 | 7.9e-05 | 0.145 | `CCCCTCCC` |
| CCCCRCCC | chr5 | − | 150455350 | 150455357 | 7.9e-05 | 0.145 | `CCCCTCCC` |
| CCCCRCCC | chr5 | + | 180169788 | 180169795 | 7.9e-05 | 0.145 | `CCCCTCCC` |
| CCCCRCCC | chr5 | − | 180170044 | 180170051 | 7.9e-05 | 0.145 | `CCCCTCCC` |
| CCCCRCCC | chr5 | + | 180595125 | 180595132 | 7.9e-05 | 0.145 | `CCCCTCCC` |
| CCCCRCCC | chr6 | + | 174250 | 174257 | 7.9e-05 | 0.145 | `CCCCTCCC` |
| CCCCRCCC | chr6 | − | 237684 | 237691 | 7.9e-05 | 0.145 | `CCCCTCCC` |
| CCCCRCCC | chr6 | + | 237942 | 237949 | 7.9e-05 | 0.145 | `CCCCCCCC` |
| CCCCRCCC | chr6 | − | 250647 | 250654 | 7.9e-05 | 0.145 | `CCCCTCCC` |
| CCCCRCCC | chr6 | − | 340239 | 340246 | 7.9e-05 | 0.145 | `CCCCCCCC` |
| CCCCRCCC | chr6 | − | 340240 | 340247 | 7.9e-05 | 0.145 | `CCCCCCCC` |
| CCCCRCCC | chr6 | − | 7831555 | 7831562 | 7.9e-05 | 0.145 | `CCCCTCCC` |
| CCCCRCCC | chr6 | − | 7844047 | 7844054 | 7.9e-05 | 0.145 | `CCCCTCCC` |
| CCCCRCCC | chr6 | + | 12119173 | 12119180 | 7.9e-05 | 0.145 | `CCCCTCCC` |
| CCCCRCCC | chr6 | − | 21696489 | 21696496 | 7.9e-05 | 0.145 | `CCCCTCCC` |
| CCCCRCCC | chr6 | + | 24828388 | 24828395 | 7.9e-05 | 0.145 | `CCCCTCCC` |
| CCCCRCCC | chr6 | − | 24829381 | 24829388 | 7.9e-05 | 0.145 | `CCCCCCCC` |
| CCCCRCCC | chr6 | − | 26304889 | 26304896 | 7.9e-05 | 0.145 | `CCCCTCCC` |
| CCCCRCCC | chr6 | + | 30565785 | 30565792 | 7.9e-05 | 0.145 | `CCCCTCCC` |
| CCCCRCCC | chr6 | + | 30792933 | 30792940 | 7.9e-05 | 0.145 | `CCCCTCCC` |
| CCCCRCCC | chr6 | + | 31241311 | 31241318 | 7.9e-05 | 0.145 | `CCCCTCCC` |
| CCCCRCCC | chr6 | + | 31728458 | 31728465 | 7.9e-05 | 0.145 | `CCCCTCCC` |
| CCCCRCCC | chr6 | + | 32047812 | 32047819 | 7.9e-05 | 0.145 | `CCCCTCCC` |
| CCCCRCCC | chr6 | + | 32047991 | 32047998 | 7.9e-05 | 0.145 | `CCCCTCCC` |
| CCCCRCCC | chr6 | − | 32253418 | 32253425 | 7.9e-05 | 0.145 | `CCCCTCCC` |
| CCCCRCCC | chr6 | + | 32266318 | 32266325 | 7.9e-05 | 0.145 | `CCCCTCCC` |
| CCCCRCCC | chr6 | − | 33045966 | 33045973 | 7.9e-05 | 0.145 | `CCCCTCCC` |
| CCCCRCCC | chr6 | + | 33047447 | 33047454 | 7.9e-05 | 0.145 | `CCCCTCCC` |
| CCCCRCCC | chr6 | − | 33352902 | 33352909 | 7.9e-05 | 0.145 | `CCCCTCCC` |
| CCCCRCCC | chr6 | + | 36756199 | 36756206 | 7.9e-05 | 0.145 | `CCCCTCCC` |
| CCCCRCCC | chr6 | + | 36756213 | 36756220 | 7.9e-05 | 0.145 | `CCCCTCCC` |
| CCCCRCCC | chr6 | + | 36756371 | 36756378 | 7.9e-05 | 0.145 | `CCCCTCCC` |
| CCCCRCCC | chr6 | + | 42822150 | 42822157 | 7.9e-05 | 0.145 | `CCCCTCCC` |
| CCCCRCCC | chr6 | − | 43005734 | 43005741 | 7.9e-05 | 0.145 | `CCCCTCCC` |
| CCCCRCCC | chr6 | + | 45996648 | 45996655 | 7.9e-05 | 0.145 | `CCCCTCCC` |
| CCCCRCCC | chr6 | + | 106656515 | 106656522 | 7.9e-05 | 0.145 | `CCCCTCCC` |
| CCCCRCCC | chr6 | + | 111019211 | 111019218 | 7.9e-05 | 0.145 | `CCCCTCCC` |
| CCCCRCCC | chr6 | − | 114058910 | 114058917 | 7.9e-05 | 0.145 | `CCCCTCCC` |
| CCCCRCCC | chr6 | − | 143310512 | 143310519 | 7.9e-05 | 0.145 | `CCCCTCCC` |
| CCCCRCCC | chr6 | + | 151753175 | 151753182 | 7.9e-05 | 0.145 | `CCCCTCCC` |
| CCCCRCCC | chr6 | − | 151753212 | 151753219 | 7.9e-05 | 0.145 | `CCCCTCCC` |
| CCCCRCCC | chr7 | − | 929567 | 929574 | 7.9e-05 | 0.145 | `CCCCTCCC` |
| CCCCRCCC | chr7 | + | 4648309 | 4648316 | 7.9e-05 | 0.145 | `CCCCTCCC` |
| CCCCRCCC | chr7 | + | 12217430 | 12217437 | 7.9e-05 | 0.145 | `CCCCTCCC` |
| CCCCRCCC | chr7 | − | 44072817 | 44072824 | 7.9e-05 | 0.145 | `CCCCTCCC` |
| CCCCRCCC | chr7 | + | 47944298 | 47944305 | 7.9e-05 | 0.145 | `CCCCTCCC` |
| CCCCRCCC | chr7 | + | 73263158 | 73263165 | 7.9e-05 | 0.145 | `CCCCTCCC` |
| CCCCRCCC | chr7 | + | 75515398 | 75515405 | 7.9e-05 | 0.145 | `CCCCTCCC` |
| CCCCRCCC | chr7 | + | 86942094 | 86942101 | 7.9e-05 | 0.145 | `CCCCTCCC` |
| CCCCRCCC | chr7 | − | 86942155 | 86942162 | 7.9e-05 | 0.145 | `CCCCTCCC` |
| CCCCRCCC | chr7 | − | 92281003 | 92281010 | 7.9e-05 | 0.145 | `CCCCTCCC` |
| CCCCRCCC | chr7 | + | 100019332 | 100019339 | 7.9e-05 | 0.145 | `CCCCTCCC` |
| CCCCRCCC | chr7 | + | 100019556 | 100019563 | 7.9e-05 | 0.145 | `CCCCTCCC` |
| CCCCRCCC | chr7 | − | 100019675 | 100019682 | 7.9e-05 | 0.145 | `CCCCTCCC` |
| CCCCRCCC | chr7 | + | 104441077 | 104441084 | 7.9e-05 | 0.145 | `CCCCTCCC` |
| CCCCRCCC | chr7 | − | 106597499 | 106597506 | 7.9e-05 | 0.145 | `CCCCTCCC` |
| CCCCRCCC | chr7 | + | 135312052 | 135312059 | 7.9e-05 | 0.145 | `CCCCTCCC` |
| CCCCRCCC | chr8 | − | 29443466 | 29443473 | 7.9e-05 | 0.145 | `CCCCTCCC` |
| CCCCRCCC | chr8 | − | 33531542 | 33531549 | 7.9e-05 | 0.145 | `CCCCTCCC` |
| CCCCRCCC | chr8 | + | 61985112 | 61985119 | 7.9e-05 | 0.145 | `CCCCTCCC` |
| CCCCRCCC | chr8 | − | 72918681 | 72918688 | 7.9e-05 | 0.145 | `CCCCTCCC` |
| CCCCRCCC | chr8 | − | 91065663 | 91065670 | 7.9e-05 | 0.145 | `CCCCTCCC` |
| CCCCRCCC | chr8 | − | 91065678 | 91065685 | 7.9e-05 | 0.145 | `CCCCTCCC` |
| CCCCRCCC | chr8 | + | 98725392 | 98725399 | 7.9e-05 | 0.145 | `CCCCTCCC` |
| CCCCRCCC | chr8 | − | 103945683 | 103945690 | 7.9e-05 | 0.145 | `CCCCTCCC` |
| CCCCRCCC | chr8 | − | 103945729 | 103945736 | 7.9e-05 | 0.145 | `CCCCTCCC` |
| CCCCRCCC | chr8 | − | 125718987 | 125718994 | 7.9e-05 | 0.145 | `CCCCTCCC` |
| CCCCRCCC | chr8 | + | 129395376 | 129395383 | 7.9e-05 | 0.145 | `CCCCTCCC` |
| CCCCRCCC | chr8 | + | 129395580 | 129395587 | 7.9e-05 | 0.145 | `CCCCTCCC` |
| CCCCRCCC | chr9 | − | 3515984 | 3515991 | 7.9e-05 | 0.145 | `CCCCTCCC` |
| CCCCRCCC | chr9 | − | 36983946 | 36983953 | 7.9e-05 | 0.145 | `CCCCTCCC` |
| CCCCRCCC | chr9 | − | 91247440 | 91247447 | 7.9e-05 | 0.145 | `CCCCTCCC` |
| CCCCRCCC | chr9 | + | 97678125 | 97678132 | 7.9e-05 | 0.145 | `CCCCTCCC` |
| CCCCRCCC | chr9 | + | 115212660 | 115212667 | 7.9e-05 | 0.145 | `CCCCTCCC` |
| CCCCRCCC | chr9 | − | 115325763 | 115325770 | 7.9e-05 | 0.145 | `CCCCTCCC` |
| CCCCRCCC | chr9 | + | 115384054 | 115384061 | 7.9e-05 | 0.145 | `CCCCTCCC` |
| CCCCRCCC | chr9 | + | 127043610 | 127043617 | 7.9e-05 | 0.145 | `CCCCCCCC` |
| CCCCRCCC | chr9 | + | 129253813 | 129253820 | 7.9e-05 | 0.145 | `CCCCTCCC` |
| CCCCRCCC | chr9 | + | 130684574 | 130684581 | 7.9e-05 | 0.145 | `CCCCTCCC` |
| CCCCRCCC | chrX | − | 24078220 | 24078227 | 7.9e-05 | 0.145 | `CCCCCCCC` |
| CCCCRCCC | chrX | − | 24078221 | 24078228 | 7.9e-05 | 0.145 | `CCCCCCCC` |
| CCCCRCCC | chrX | − | 24078293 | 24078300 | 7.9e-05 | 0.145 | `CCCCTCCC` |
| CCCCRCCC | chrX | − | 29483887 | 29483894 | 7.9e-05 | 0.145 | `CCCCTCCC` |
| CCCCRCCC | chrX | − | 38545861 | 38545868 | 7.9e-05 | 0.145 | `CCCCTCCC` |
| CCCCRCCC | chrX | + | 48655497 | 48655504 | 7.9e-05 | 0.145 | `CCCCTCCC` |
| CCCCRCCC | chrX | + | 48681153 | 48681160 | 7.9e-05 | 0.145 | `CCCCTCCC` |
| CCCCRCCC | chrX | + | 70759328 | 70759335 | 7.9e-05 | 0.145 | `CCCCTCCC` |
| CCCCRCCC | chrX | − | 151750091 | 151750098 | 7.9e-05 | 0.145 | `CCCCTCCC` |
| CCCCRCCC | chrX | − | 151750224 | 151750231 | 7.9e-05 | 0.145 | `CCCCTCCC` |
| CCCCRCCC | chr10 | + | 1085404 | 1085411 | 7.9e-05 | 0.145 | `CCCCCCCC` |
| CCCCRCCC | chr10 | + | 1085408 | 1085415 | 7.9e-05 | 0.145 | `CCCCTCCC` |
| CCCCRCCC | chr10 | + | 1085413 | 1085420 | 7.9e-05 | 0.145 | `CCCCCCCC` |
| CCCCRCCC | chr10 | + | 1085414 | 1085421 | 7.9e-05 | 0.145 | `CCCCCCCC` |
| CCCCRCCC | chr10 | + | 1085415 | 1085422 | 7.9e-05 | 0.145 | `CCCCCCCC` |
| CCCCRCCC | chr10 | − | 35455886 | 35455893 | 7.9e-05 | 0.145 | `CCCCTCCC` |
| CCCCRCCC | chr10 | − | 69957328 | 69957335 | 7.9e-05 | 0.145 | `CCCCTCCC` |
| CCCCRCCC | chr10 | − | 71164845 | 71164852 | 7.9e-05 | 0.145 | `CCCCTCCC` |
| CCCCRCCC | chr10 | − | 73705349 | 73705356 | 7.9e-05 | 0.145 | `CCCCTCCC` |
| CCCCRCCC | chr10 | − | 73705621 | 73705628 | 7.9e-05 | 0.145 | `CCCCTCCC` |
| CCCCRCCC | chr10 | + | 73705650 | 73705657 | 7.9e-05 | 0.145 | `CCCCTCCC` |
| CCCCRCCC | chr10 | + | 73727427 | 73727434 | 7.9e-05 | 0.145 | `CCCCTCCC` |
| CCCCRCCC | chr10 | + | 73765470 | 73765477 | 7.9e-05 | 0.145 | `CCCCTCCC` |
| CCCCRCCC | chr10 | + | 89912723 | 89912730 | 7.9e-05 | 0.145 | `CCCCTCCC` |
| CCCCRCCC | chr10 | + | 104411135 | 104411142 | 7.9e-05 | 0.145 | `CCCCTCCC` |
| CCCCRCCC | chr10 | − | 105221297 | 105221304 | 7.9e-05 | 0.145 | `CCCCTCCC` |
| CCCCRCCC | chr10 | + | 112145578 | 112145585 | 7.9e-05 | 0.145 | `CCCCTCCC` |
| CCCCRCCC | chr10 | + | 115732478 | 115732485 | 7.9e-05 | 0.145 | `CCCCTCCC` |
| CCCCRCCC | chr10 | − | 115732902 | 115732909 | 7.9e-05 | 0.145 | `CCCCTCCC` |
| CCCCRCCC | chr10 | − | 121239314 | 121239321 | 7.9e-05 | 0.145 | `CCCCTCCC` |
| CCCCRCCC | chr10 | + | 126375212 | 126375219 | 7.9e-05 | 0.145 | `CCCCTCCC` |
| CCCCRCCC | chr11 | + | 1830617 | 1830624 | 7.9e-05 | 0.145 | `CCCCTCCC` |
| CCCCRCCC | chr11 | − | 35104969 | 35104976 | 7.9e-05 | 0.145 | `CCCCTCCC` |
| CCCCRCCC | chr11 | + | 47386215 | 47386222 | 7.9e-05 | 0.145 | `CCCCTCCC` |
| CCCCRCCC | chr11 | − | 47556902 | 47556909 | 7.9e-05 | 0.145 | `CCCCTCCC` |
| CCCCRCCC | chr11 | + | 47556938 | 47556945 | 7.9e-05 | 0.145 | `CCCCTCCC` |
| CCCCRCCC | chr11 | + | 62081471 | 62081478 | 7.9e-05 | 0.145 | `CCCCTCCC` |
| CCCCRCCC | chr11 | − | 63794262 | 63794269 | 7.9e-05 | 0.145 | `CCCCTCCC` |
| CCCCRCCC | chr11 | + | 64402773 | 64402780 | 7.9e-05 | 0.145 | `CCCCTCCC` |
| CCCCRCCC | chr11 | − | 64650932 | 64650939 | 7.9e-05 | 0.145 | `CCCCTCCC` |
| CCCCRCCC | chr11 | − | 64656557 | 64656564 | 7.9e-05 | 0.145 | `CCCCTCCC` |
| CCCCRCCC | chr11 | + | 64656756 | 64656763 | 7.9e-05 | 0.145 | `CCCCTCCC` |
| CCCCRCCC | chr11 | − | 64943135 | 64943142 | 7.9e-05 | 0.145 | `CCCCTCCC` |
| CCCCRCCC | chr11 | + | 65021712 | 65021719 | 7.9e-05 | 0.145 | `CCCCTCCC` |
| CCCCRCCC | chr11 | + | 65098331 | 65098338 | 7.9e-05 | 0.145 | `CCCCTCCC` |
| CCCCRCCC | chr11 | + | 65098336 | 65098343 | 7.9e-05 | 0.145 | `CCCCCCCC` |
| CCCCRCCC | chr11 | − | 65098699 | 65098706 | 7.9e-05 | 0.145 | `CCCCTCCC` |
| CCCCRCCC | chr11 | − | 68821376 | 68821383 | 7.9e-05 | 0.145 | `CCCCTCCC` |
| CCCCRCCC | chr11 | − | 69209817 | 69209824 | 7.9e-05 | 0.145 | `CCCCTCCC` |
| CCCCRCCC | chr11 | − | 82450868 | 82450875 | 7.9e-05 | 0.145 | `CCCCTCCC` |
| CCCCRCCC | chr11 | + | 85603397 | 85603404 | 7.9e-05 | 0.145 | `CCCCTCCC` |
| CCCCRCCC | chr11 | − | 118071644 | 118071651 | 7.9e-05 | 0.145 | `CCCCTCCC` |
| CCCCRCCC | chr11 | − | 118302030 | 118302037 | 7.9e-05 | 0.145 | `CCCCTCCC` |
| CCCCRCCC | chr11 | − | 122438376 | 122438383 | 7.9e-05 | 0.145 | `CCCCTCCC` |
| CCCCRCCC | chr11 | − | 122438424 | 122438431 | 7.9e-05 | 0.145 | `CCCCTCCC` |
| CCCCRCCC | chr11 | + | 127844463 | 127844470 | 7.9e-05 | 0.145 | `CCCCTCCC` |
| CCCCRCCC | chr12 | + | 4122637 | 4122644 | 7.9e-05 | 0.145 | `CCCCTCCC` |
| CCCCRCCC | chr12 | − | 6514168 | 6514175 | 7.9e-05 | 0.145 | `CCCCTCCC` |
| CCCCRCCC | chr12 | − | 6514173 | 6514180 | 7.9e-05 | 0.145 | `CCCCTCCC` |
| CCCCRCCC | chr12 | + | 6933161 | 6933168 | 7.9e-05 | 0.145 | `CCCCTCCC` |
| CCCCRCCC | chr12 | + | 14410002 | 14410009 | 7.9e-05 | 0.145 | `CCCCTCCC` |
| CCCCRCCC | chr12 | − | 31940878 | 31940885 | 7.9e-05 | 0.145 | `CCCCTCCC` |
| CCCCRCCC | chr12 | + | 52132148 | 52132155 | 7.9e-05 | 0.145 | `CCCCTCCC` |
| CCCCRCCC | chr12 | + | 67487685 | 67487692 | 7.9e-05 | 0.145 | `CCCCTCCC` |
| CCCCRCCC | chr12 | − | 67524551 | 67524558 | 7.9e-05 | 0.145 | `CCCCTCCC` |
| CCCCRCCC | chr12 | + | 91281519 | 91281526 | 7.9e-05 | 0.145 | `CCCCTCCC` |
| CCCCRCCC | chr12 | + | 93479848 | 93479855 | 7.9e-05 | 0.145 | `CCCCTCCC` |
| CCCCRCCC | chr12 | − | 97421824 | 97421831 | 7.9e-05 | 0.145 | `CCCCTCCC` |
| CCCCRCCC | chr12 | + | 102867607 | 102867614 | 7.9e-05 | 0.145 | `CCCCTCCC` |
| CCCCRCCC | chr12 | − | 109592434 | 109592441 | 7.9e-05 | 0.145 | `CCCCTCCC` |
| CCCCRCCC | chr12 | − | 115481838 | 115481845 | 7.9e-05 | 0.145 | `CCCCTCCC` |
| CCCCRCCC | chr12 | + | 123965536 | 123965543 | 7.9e-05 | 0.145 | `CCCCTCCC` |
| CCCCRCCC | chr12 | + | 123967990 | 123967997 | 7.9e-05 | 0.145 | `CCCCTCCC` |
| CCCCRCCC | chr13 | + | 45319738 | 45319745 | 7.9e-05 | 0.145 | `CCCCTCCC` |
| CCCCRCCC | chr13 | − | 48005071 | 48005078 | 7.9e-05 | 0.145 | `CCCCTCCC` |
| CCCCRCCC | chr13 | + | 51666612 | 51666619 | 7.9e-05 | 0.145 | `CCCCTCCC` |
| CCCCRCCC | chr14 | + | 20744233 | 20744240 | 7.9e-05 | 0.145 | `CCCCTCCC` |
| CCCCRCCC | chr14 | − | 22095383 | 22095390 | 7.9e-05 | 0.145 | `CCCCTCCC` |
| CCCCRCCC | chr14 | + | 22095656 | 22095663 | 7.9e-05 | 0.145 | `CCCCCCCC` |
| CCCCRCCC | chr14 | + | 22095657 | 22095664 | 7.9e-05 | 0.145 | `CCCCCCCC` |
| CCCCRCCC | chr14 | − | 23734853 | 23734860 | 7.9e-05 | 0.145 | `CCCCTCCC` |
| CCCCRCCC | chr14 | + | 34943008 | 34943015 | 7.9e-05 | 0.145 | `CCCCTCCC` |
| CCCCRCCC | chr14 | + | 34943961 | 34943968 | 7.9e-05 | 0.145 | `CCCCTCCC` |
| CCCCRCCC | chr14 | + | 64693208 | 64693215 | 7.9e-05 | 0.145 | `CCCCTCCC` |
| CCCCRCCC | chr14 | + | 64840387 | 64840394 | 7.9e-05 | 0.145 | `CCCCTCCC` |
| CCCCRCCC | chr14 | + | 67747686 | 67747693 | 7.9e-05 | 0.145 | `CCCCTCCC` |
| CCCCRCCC | chr14 | − | 68325403 | 68325410 | 7.9e-05 | 0.145 | `CCCCCCCC` |
| CCCCRCCC | chr14 | − | 68329681 | 68329688 | 7.9e-05 | 0.145 | `CCCCTCCC` |
| CCCCRCCC | chr14 | + | 102855351 | 102855358 | 7.9e-05 | 0.145 | `CCCCTCCC` |

---

**DEBUGGING INFORMATION**


---

Command line:

```
/ebi/sw/MEME/VM-cluster410/meme-versions/4.10.0/bin/fimo --parse-genomic-coord --verbosity 1 --oc fimo_out_10 --bgfile ./background --motif CCCCRCCC dreme_out/dreme.xml ./Supplementary_Table_1.500bp.fa
```

Settings:

```
|  |  |  |
| --- | --- | --- |
| output directory = fimo_out_10 | MEME file name = dreme_out/dreme.xml | sequence file name = ./Supplementary_Table_1.500bp.fa |
| background file name = ./background | allow clobber = true | compute q-values = true |
| parse genomic coord. = true | text only = false | scan both strands = true |
| max sequence length = 250000000 | output threshold = 0.0001 | threshold type = p-value |
| max stored scores = 100000 | pseudocount = 0.1 | verbosity = 1 |
| selected motif = CCCCRCCC |  |  |
```

This information can be useful in the event you wish to report a
problem with the FIMO software.

---

**Go to top**
