# Supplementary material for: The MEME Suite
Source: Nucleic Acids Res. 2015 May 7;43(Web Server issue):W39–49. doi: 10.1093/nar/gkv416 (PMC4489269; doi:10.1093/nar/gkv416)
Supplement: SUPPLEMENTARY DATA [file supp_gkv416_nar-00283-web-b-2015-File005.zip › case4/meme-chip/fimo_out_11/fimo.html]

FIMO Results


---

|  |  |  |
| --- | --- | --- |
| **Database and Motifs** | **High-scoring Motif Occurrences** | **Debugging Information** |

  
  

---

**FIMO - Motif search tool**


---

FIMO version 4.10.0,
(Release date: Wed May 21 10:35:36 2014 +1000)

For further information on how to interpret these results
or to get a copy of the FIMO software please access
http://meme.nbcr.net

If you use FIMO in your research, please cite the following paper:  
Charles E. Grant, Timothy L. Bailey, and William Stafford Noble,
"FIMO: Scanning for occurrences of a given motif",
*Bioinformatics*, **27**(7):1017-1018, 2011.
[full text]

---

**DATABASE AND MOTIFS**


---

DATABASE
./Supplementary\_Table\_1.500bp.fa  
Database contains
2776
sequences,
1388000
residues

MOTIFS
db/uniprobe\_mouse.meme
(nucleotide)

| MOTIF | WIDTH | BEST POSSIBLE MATCH |
| --- | --- | --- |
| UP00078\_1 | 17 | GGGTTTAATTAAAATTC |
| UP00059\_1 | 14 | CTAATATTGCTAAA |
| UP00099\_1 | 17 | CTCAGCAGCTGCTCCTG |
| UP00020\_1 | 16 | ACGATGACGTCATCGA |
| UP00012\_1 | 15 | TAATTCAATGAAGTG |
| UP00043\_1 | 16 | TCTTTCGAGGAATTTG |
| UP00050\_1 | 22 | GGAAGAGTCACGTGACCAATAC |
| UP00001\_1 | 15 | ATAAAGGCGCGCGAT |
| UP00003\_1 | 15 | ATAAGGGCGCGCGAT |
| UP00007\_1 | 14 | TCCGCCCCCGCATT |
| UP00015\_1 | 15 | AGGACCCGGAAGTAA |
| UP00407\_1 | 13 | TACAAGGAAGTAA |
| UP00068\_1 | 17 | TAAAAGGTGTGAAAATT |
| UP00079\_1 | 17 | TATTCAAGGTCATGCGA |
| UP00073\_1 | 17 | AAAAAGTAAACAAAGAC |
| UP00041\_1 | 16 | AAAGTAAACAAAAATT |
| UP00039\_1 | 17 | AAAAAGTAAACAAACCC |
| UP00025\_1 | 17 | AAAATGTAAACAAACAG |
| UP00061\_1 | 17 | TAAATGTAAACAAAGGT |
| UP00408\_1 | 17 | CAATACCGGAAGTGTAA |
| UP00032\_1 | 22 | TTTTTAGAGATAAGAAATAAAG |
| UP00080\_1 | 17 | TAAACTGATAAGAAGAT |
| UP00100\_1 | 17 | TATAGAGATAAGAATTG |
| UP00070\_1 | 16 | TCGTACCCGCATCATT |
| UP00024\_1 | 16 | TATCGACCCCCCACAG |
| UP00042\_1 | 17 | CAGATGTGCACATACGT |
| UP00084\_1 | 17 | GAGTGTACGTACGATGG |
| UP00055\_1 | 16 | ACTATGAATGAATGAT |
| UP00035\_1 | 16 | ACTATGCCAACCTACC |
| UP00066\_1 | 17 | CTTCAGGGGTCAATTGA |
| UP00391\_1 | 14 | TGGAGGTAATTAAC |
| UP00072\_1 | 16 | ATTTACGACAAATAGC |
| UP00086\_1 | 14 | GAGAACCGAAACTG |
| UP00018\_1 | 15 | CGTATCGAAACCAAA |
| UP00040\_1 | 15 | ATAAACCGAAACCAA |
| UP00011\_1 | 17 | CTGATCGAAACCAAAGT |
| UP00074\_1 | 15 | CAAAATCGAAACTAA |
| UP00103\_1 | 16 | CCGATGACGTCATCGT |
| UP00093\_1 | 16 | TCGACCCCGCCCCTAT |
| UP00067\_1 | 17 | AATCCCTTTGATCTATC |
| UP00045\_1 | 17 | AAATTTGCTGACTTAGC |
| UP00044\_1 | 15 | TAAAAATGCTGACTT |
| UP00060\_1 | 16 | TGACCACGTGGTCGGG |
| UP00097\_1 | 16 | GGGCCGTGTGCAAAAA |
| UP00092\_1 | 17 | ATGGAAACCGTTATTTT |
| UP00081\_1 | 17 | TTGAAAACCGTTAATTT |
| UP00036\_1 | 16 | GAAGAACAGGTGTCCG |
| UP00017\_1 | 17 | CTTAACCACTTAAGGAT |
| UP00009\_1 | 16 | TCTCAAAGGTCACGAG |
| UP00027\_1 | 16 | TTTTACAGTAGCAAAA |
| UP00052\_1 | 16 | ATGTACAGTAGCAAAG |
| UP00088\_1 | 16 | TTGGGGGCGCCCCTAG |
| UP00048\_1 | 16 | TCTCAAAGGTCACCTG |
| UP00098\_1 | 23 | TGTGACCCTTAGCAACCGATTAA |
| UP00056\_1 | 15 | TACCATAGCAACGGT |
| UP00076\_1 | 15 | CCGCATAGCAACGGA |
| UP00053\_1 | 17 | TGTCGTGACCCCTTAAT |
| UP00085\_1 | 14 | TTAAGAGGAAGTTA |
| UP00008\_1 | 17 | AATAGGGTATCATATAT |
| UP00000\_1 | 17 | CAAATCCAGACATCAGA |
| UP00030\_1 | 17 | ATAAGAACAAAGGACTA |
| UP00101\_1 | 14 | TAATTGTTCTAAAC |
| UP00096\_1 | 16 | TTAAGAACAATAATTT |
| UP00004\_1 | 16 | GCTAATTATAATTATC |
| UP00075\_1 | 17 | TAGTGAACAATAGATTT |
| UP00014\_1 | 15 | ATAAACAATTAATCA |
| UP00064\_1 | 16 | TTCAATTGTTCTAAAA |
| UP00069\_1 | 16 | AATCAATTCAATAATT |
| UP00071\_1 | 16 | TTTAATTATAATTAAG |
| UP00023\_1 | 16 | ATTGAACAATGGAATT |
| UP00062\_1 | 17 | AGAAGAACAAAGGACTA |
| UP00091\_1 | 16 | TTTAGAACAATAAAAT |
| UP00034\_1 | 22 | AATAAAGAACAATAGAATTTCA |
| UP00051\_1 | 17 | TTATCTATTGTTCTTTA |
| UP00049\_1 | 14 | ATTTTACGGAAAAT |
| UP00002\_1 | 17 | GGTCCCGCCCCCTTCTC |
| UP00406\_1 | 16 | GTACATCCGGATTTTT |
| UP00077\_1 | 14 | TTCCATATATGGAA |
| UP00016\_1 | 16 | TATAATTATAATATTC |
| UP00029\_1 | 16 | TCTTTATATATAAATA |
| UP00089\_1 | 17 | ACTTAGTTAACTAAAAA |
| UP00058\_1 | 17 | TATAGATCAAAGGAAAA |
| UP00054\_1 | 17 | TATAGATCAAAGGAAAA |
| UP00083\_1 | 17 | ATTTCCTTTGATCTATA |
| UP00005\_1 | 15 | ATTCCCTGAGGGGAA |
| UP00010\_1 | 14 | TTGCCCTAGGGCAT |
| UP00087\_1 | 15 | ATTGCCTGAGGCGAA |
| UP00028\_1 | 15 | ATTGCCTGAGGCGAT |
| UP00046\_1 | 17 | ATCCACAGGTGCGAAAA |
| UP00019\_1 | 17 | CTAAGGTTCTAGATCAC |
| UP00031\_1 | 17 | AATCGCACTGCATTCCG |
| UP00047\_1 | 15 | AAGCCCCCCAAAAAT |
| UP00037\_1 | 15 | AACAAACAACAAGAG |
| UP00094\_1 | 17 | TCTTTGGCGTACCCTAA |
| UP00065\_1 | 16 | TGGCGCGCGCGCCTGA |
| UP00082\_1 | 14 | TTATGTACTAATAA |
| UP00021\_1 | 15 | TCCCCCCCCCCCCCC |
| UP00033\_1 | 17 | TATTATGGGATGGATAA |
| UP00095\_1 | 17 | CGAACAGTGCTCACTAT |
| UP00022\_1 | 16 | CCCCCCCCCCCACTTG |
| UP00102\_1 | 14 | CACCCCCGGGGGGG |
| UP00057\_1 | 15 | CCCCCCCGGGGGGGT |
| UP00006\_1 | 15 | CCCCCCCGGGGGGGT |
| UP00026\_1 | 17 | TACATGTGCACATAAAA |
| UP00078\_2 | 15 | ACCCGTATCAAATTT |
| UP00059\_2 | 17 | CGTACAATACGAAATAA |
| UP00099\_2 | 16 | CTATCCCCGCCCTATT |
| UP00020\_2 | 14 | GAATGACGAATAAC |
| UP00012\_2 | 17 | TGATTGTTAACAGTTGG |
| UP00043\_2 | 16 | ATCCCCGCCCCTAAAA |
| UP00050\_2 | 23 | TGTCGTTACACGTGGAAGGCGGT |
| UP00001\_2 | 17 | CGTTCGGCGCCAAAAGG |
| UP00003\_2 | 17 | CGCTCGGCGCCAAAAGC |
| UP00007\_2 | 16 | TGCGGAGTGGGACTGG |
| UP00015\_2 | 16 | TAGTATTTCCGATCTT |
| UP00407\_2 | 17 | GTTCAAAAAAAAAATTC |
| UP00068\_2 | 16 | GCGGAGGTGTCGCCTC |
| UP00079\_2 | 17 | GGCGAGGGGTCAAGGGC |
| UP00073\_2 | 15 | AAAAATAACAAACGG |
| UP00041\_2 | 15 | ATGTCACAACAACAC |
| UP00039\_2 | 17 | AACACCAAAACAAAGGA |
| UP00025\_2 | 15 | CAAACAACAACACCT |
| UP00061\_2 | 16 | ATATCAAAACAAAACA |
| UP00408\_2 | 16 | CCGTCTTCCCCCTCAC |
| UP00032\_2 | 22 | TTTTGTAGATTTTATCGACTTA |
| UP00080\_2 | 17 | GACAGAGATATCAGTTT |
| UP00100\_2 | 17 | GCGGCGATATCGCAGCG |
| UP00070\_2 | 17 | TGCGCATAGGGGAGGAG |
| UP00024\_2 | 14 | AATATTAATAAAGA |
| UP00042\_2 | 16 | AGCGGCACACACGCAA |
| UP00084\_2 | 16 | TGGGCGACGTCGTTAA |
| UP00055\_2 | 17 | TGTTCCCATTGTGTACT |
| UP00035\_2 | 16 | GGGTGTGCCCAAAAGG |
| UP00066\_2 | 16 | TGCAAAAGTCCAATAT |
| UP00391\_2 | 14 | AAAAACCATTAAGG |
| UP00072\_2 | 16 | ATGGAAAGTCGTAAAA |
| UP00086\_2 | 14 | GGAGAAAGGTGCGA |
| UP00018\_2 | 15 | AGTATTCTCGGTTGC |
| UP00040\_2 | 15 | TTGATCGAGAATTCC |
| UP00011\_2 | 15 | ACCACTCTCGGTCAC |
| UP00074\_2 | 14 | GCAAAACATTACTA |
| UP00103\_2 | 16 | ATTGATGAGTCACCAA |
| UP00093\_2 | 17 | AAGCATACGCCCAACTT |
| UP00067\_2 | 16 | GAAGATCAATCACTTA |
| UP00045\_2 | 15 | CAATTGCAAAAATAT |
| UP00044\_2 | 15 | GAAAAAATTGCAAGG |
| UP00060\_2 | 14 | GTGCCACGCGACTG |
| UP00097\_2 | 14 | AAATAAGAAAAAAC |
| UP00092\_2 | 16 | CGACCAACTGCCATGC |
| UP00081\_2 | 15 | CGACCAACTGCCGTG |
| UP00036\_2 | 15 | AGCAACAGCCGCACC |
| UP00017\_2 | 17 | ACTCCAAGTACTTGGAA |
| UP00009\_2 | 16 | CGCGCCGGGTCACGTA |
| UP00027\_2 | 16 | ACATGCTACCTAATAC |
| UP00052\_2 | 16 | ACTTGCTACCTACACC |
| UP00088\_2 | 17 | GCTGGGGGGTACCCCTT |
| UP00048\_2 | 16 | AGAGCGGGGTCAAGTA |
| UP00098\_2 | 23 | ACTGACGCTTGGTTACCACAAAG |
| UP00056\_2 | 15 | TACCCTAGTTACCGA |
| UP00076\_2 | 17 | CTACTTGGATACGGAAT |
| UP00053\_2 | 16 | TCGCGAAGGTTGTACT |
| UP00085\_2 | 14 | CAAATTCCGGAACC |
| UP00008\_2 | 17 | ATGGGATATATCCGCCT |
| UP00000\_2 | 17 | TACGCCCCGCCACTCTG |
| UP00030\_2 | 14 | AAAATTGTTATGAA |
| UP00101\_2 | 16 | AAATAGACAAAGGAAT |
| UP00096\_2 | 17 | GTATTGGGTGGGTATTT |
| UP00004\_2 | 15 | CTCACACAATGGCGC |
| UP00075\_2 | 15 | TTGAATGAAATTCGA |
| UP00014\_2 | 17 | GACCACATTCATACAAT |
| UP00064\_2 | 16 | GGACTGAATTCATGCC |
| UP00069\_2 | 15 | CTATAATTGTTATCG |
| UP00071\_2 | 17 | CATCAATTGTTCCGCTA |
| UP00023\_2 | 16 | TAAGATTATAATACGG |
| UP00062\_2 | 17 | GGAAAAATTGTTAGGAA |
| UP00091\_2 | 15 | TATCATAATTAAGGA |
| UP00034\_2 | 22 | GTGCTAATTGTGTGTGTACGCT |
| UP00051\_2 | 14 | ACATTCATGACACG |
| UP00049\_2 | 15 | TCCGTCGCTTAAAAG |
| UP00002\_2 | 15 | CAAAGGCGTGGCCAG |
| UP00406\_2 | 16 | GATAACATCCTAGTAG |
| UP00077\_2 | 17 | GTTAAAAAAAAAAATTT |
| UP00016\_2 | 17 | TCACGGAACAATAGGTG |
| UP00029\_2 | 15 | CCGATTTAAGCGATC |
| UP00089\_2 | 14 | TTGCCCGGATTAGG |
| UP00058\_2 | 15 | AGCCGAAAAAAAAAT |
| UP00054\_2 | 15 | CCGTATTATAAACAA |
| UP00083\_2 | 16 | GAAGATCAATCACTAA |
| UP00005\_2 | 14 | TCACCTCTGGGCAG |
| UP00010\_2 | 15 | ATTGCCTCAGGCAAT |
| UP00087\_2 | 14 | CCGCCCAAGGGCAG |
| UP00028\_2 | 14 | TACTGGAAAAAAAA |
| UP00046\_2 | 17 | AAGGCCAGATGGTCCGG |
| UP00019\_2 | 15 | TATCATTAGAACGCT |
| UP00031\_2 | 16 | CAATCACTGGCAGAAT |
| UP00047\_2 | 17 | CTTAAGACCACCATTAC |
| UP00037\_2 | 17 | GTGGTTCAATAATTTTG |
| UP00094\_2 | 14 | TGTATATATATACC |
| UP00065\_2 | 14 | GCCGCGCAGTGCGT |
| UP00082\_2 | 16 | GAGCCCTTGTCCCTTG |
| UP00021\_2 | 17 | AGGAGACCCCCAATTTG |
| UP00033\_2 | 17 | TCACCCCGCCCCTAATT |
| UP00095\_2 | 17 | TACGAGACTCCTCTAAC |
| UP00022\_2 | 17 | AAATTCCCCCCGGAAGT |
| UP00102\_2 | 15 | CCACACAGCAGGAGA |
| UP00057\_2 | 15 | CCACACAGCAGGAGA |
| UP00006\_2 | 15 | GAGCACAGCAGGACA |
| UP00026\_2 | 16 | CGAAGCACACAAAATA |
| UP00108\_1 | 17 | TAAACTAATTAGCTGAG |
| UP00187\_1 | 17 | CGCATTAATTAATTACC |
| UP00152\_1 | 17 | GTCCATTAATTAATGGA |
| UP00228\_1 | 17 | CATAACCACTTAACAAC |
| UP00166\_1 | 16 | AACAACCAATTAATTC |
| UP00145\_1 | 16 | AAAAACCAATTAAGAA |
| UP00181\_1 | 16 | AAAGTAATTAGTGAAT |
| UP00151\_1 | 16 | TAAGTAATTAGTTATA |
| UP00138\_1 | 16 | CAGGTAATTACCTCAG |
| UP00209\_1 | 17 | CGAATTAATTAATCACC |
| UP00209\_2 | 17 | CGCATTAATTAATTGGC |
| UP00240\_1 | 16 | TAAGGTAATAAAATTA |
| UP00133\_1 | 16 | AACGGTAATAAAATTT |
| UP00198\_1 | 14 | ATGATCGAATCAAA |
| UP00176\_1 | 16 | CGTTGGGGATTAGCCT |
| UP00219\_1 | 17 | ACCGGTTGATCACCTGA |
| UP00219\_2 | 15 | TAATGATGATCACTA |
| UP00255\_1 | 17 | TAATTAATTAATAATTA |
| UP00218\_1 | 16 | TTTAATTAATTAATTC |
| UP00202\_1 | 14 | CTGAGGTAATTAAT |
| UP00126\_1 | 16 | GGAATAATTACTTCAG |
| UP00154\_1 | 17 | TCGCGATAATTACCGAC |
| UP00110\_1 | 17 | TCGCTATAATTACCGAC |
| UP00230\_1 | 16 | GGGGTAATTAGCTCTG |
| UP00111\_1 | 17 | TGAACCGGATTAATGAA |
| UP00232\_1 | 17 | TAAATAGATACCCCATA |
| UP00143\_1 | 17 | GGAAGGGATTAATTATC |
| UP00227\_1 | 17 | CGACCCAATCAACGGTG |
| UP00201\_1 | 17 | ACCACTAATTAGTGGAC |
| UP00167\_1 | 16 | GCGAACTAATTAATGC |
| UP00163\_1 | 17 | TGCACTAATTAGTGGAA |
| UP00251\_1 | 17 | ATCCATTAATTAATTGA |
| UP00162\_1 | 17 | AGAACTAATTAGTGGAC |
| UP00132\_1 | 17 | CACCGCTAATTAGCGTT |
| UP00204\_1 | 17 | TGCCACTAATTAGTGTA |
| UP00131\_1 | 17 | AGCGCTAATTAGCGATT |
| UP00112\_1 | 17 | AATCGTTAATCCCTTTA |
| UP00127\_1 | 16 | AGGTTAATTAGCTGAT |
| UP00148\_1 | 17 | AAGGCGAAATCATCGCA |
| UP00225\_1 | 15 | CCATAATTAATTACA |
| UP00123\_1 | 16 | GTACTAATTAGTGGCG |
| UP00161\_1 | 17 | GAAAACTAGTTAACATC |
| UP00104\_1 | 17 | ACAAGCAATTAATGAAT |
| UP00155\_1 | 17 | ACAAGCAATTAAAGAAT |
| UP00157\_1 | 17 | ACAAGCAATTAAAGAAT |
| UP00114\_1 | 17 | AAAACATCGTTTTTAAG |
| UP00264\_1 | 16 | CTGAGCTAATTACCGT |
| UP00217\_1 | 16 | TAGGTAATAAAATTCA |
| UP00246\_1 | 16 | TAAAGTCGTAAAACAT |
| UP00183\_1 | 16 | AAAGCTCGTAAAATTT |
| UP00174\_1 | 16 | AAGGTAATTAGCTCAT |
| UP00391\_3 | 14 | TTGAGGTAATTAGT |
| UP00196\_1 | 17 | GATTATTAATTAACTTG |
| UP00189\_1 | 16 | ACGGTAATTAGCTCAG |
| UP00182\_1 | 16 | AAGGTAATTACCTAAT |
| UP00164\_1 | 17 | CGAGTTAATTAATAAGC |
| UP00164\_2 | 16 | GTAGTAATTAATGGAA |
| UP00213\_1 | 17 | ACGGCCATAAAATTAAT |
| UP00134\_1 | 16 | AACCCAATAAAATTCG |
| UP00137\_1 | 17 | TGAGCTAATTAGTTGGA |
| UP00144\_1 | 17 | CGCGTTAATTAATTACC |
| UP00214\_1 | 16 | ACGGTAATTAGCTCAT |
| UP00259\_1 | 16 | TATTGGTAATTACCTT |
| UP00206\_1 | 16 | GTAGTAATTAATGCAA |
| UP00263\_1 | 16 | ACCGGCAATTAATAAA |
| UP00207\_1 | 16 | GGAGCCATAAAATTCG |
| UP00245\_1 | 16 | TAAAGTCGTAAAACGT |
| UP00235\_1 | 16 | TAAAGTCGTAAAATAG |
| UP00135\_1 | 17 | TTAGGTCGTAAAATTTC |
| UP00173\_1 | 16 | AAAGCTCGTAAAATTT |
| UP00113\_1 | 17 | CGAATTAATTAACAATA |
| UP00252\_1 | 17 | CGAATTAATTAATTACT |
| UP00260\_1 | 17 | CAAATTAATTAATAAAA |
| UP00242\_1 | 16 | TTGGGGTAATTAACGT |
| UP00197\_1 | 16 | GGAGGTCATTAATTAT |
| UP00140\_1 | 17 | TAAACTAATTAGCTGTA |
| UP00121\_1 | 17 | AATGCAATAAAATTTAT |
| UP00117\_1 | 17 | TAAGGTCGTAAAATCCT |
| UP00177\_1 | 17 | CAAGGTCGTAAAATCTT |
| UP00180\_1 | 16 | CTACCAATAAAATTCT |
| UP00241\_1 | 16 | TTGAGTTAATTAACCT |
| UP00168\_1 | 17 | TAATTAATTAATGGCTA |
| UP00124\_1 | 16 | AAGGTAATTAGCTCAT |
| UP00236\_1 | 17 | TAAATACATGTAAAATT |
| UP00223\_1 | 17 | AAAATACATGTAATACT |
| UP00223\_2 | 17 | AATATACATGTAATATT |
| UP00194\_1 | 17 | AATATACATGTAAAACA |
| UP00250\_1 | 17 | TATATACATGTAAAATT |
| UP00150\_1 | 17 | AAAATACATGTAAAAAT |
| UP00170\_1 | 16 | CAAAATCAATTAATTT |
| UP00243\_1 | 16 | ACTCCTAATTAGTCGT |
| UP00120\_1 | 17 | TGCATTAATTAATGCGA |
| UP00262\_1 | 17 | CGAATTAATTAATAATG |
| UP00115\_1 | 17 | TAAACTAATTAGTGAAC |
| UP00130\_1 | 17 | GTAATTAATTAAATAAT |
| UP00261\_1 | 17 | TAAACTAATTAGCTTTG |
| UP00212\_1 | 17 | CGAATTAATTAAATACT |
| UP00256\_1 | 17 | GAGCGTTAATTAATGTA |
| UP00256\_2 | 17 | TCCACTAATTAGCGGTT |
| UP00184\_1 | 17 | ACCCCTAATTAGCGGTG |
| UP00175\_1 | 17 | CCCATTAATTAATCACC |
| UP00188\_1 | 17 | CGAATTAATTAAAAACC |
| UP00169\_1 | 17 | AGTTTTTAATTAATTTG |
| UP00186\_1 | 16 | AAGGAGCTGTCAATAC |
| UP00233\_1 | 16 | GAGGTAATTACCTCAG |
| UP00226\_1 | 16 | AAAGACCTGTCAATAC |
| UP00210\_1 | 16 | AATTACCTGTCAATAC |
| UP00234\_1 | 16 | TGCAACTAATTAATTC |
| UP00156\_1 | 17 | GAAGACCAATTAGCGCT |
| UP00171\_1 | 16 | CAAAACCAATTAATTT |
| UP00220\_1 | 17 | TGCGCTAATTAGTGGGA |
| UP00139\_1 | 17 | GTGCACTAATTAGTGCA |
| UP00231\_1 | 17 | TTAACCACTTGAAAATT |
| UP00190\_1 | 16 | CTTTAAGTACTTAATG |
| UP00107\_1 | 16 | TAAGCCACTTGAAATT |
| UP00249\_1 | 16 | TAAGCCACTTGAATTT |
| UP00147\_1 | 16 | TAAGCCACTTAACATT |
| UP00119\_1 | 17 | TTTTAAGTACTTAAATT |
| UP00017\_3 | 17 | TACTAAGTACTTAAATG |
| UP00200\_1 | 17 | GAAAATTAATTACTTCG |
| UP00200\_2 | 16 | AGTAATTAATTACTTC |
| UP00238\_1 | 17 | GATAATTAATTACTTTG |
| UP00216\_1 | 17 | TTAAGGGGATTAACTAC |
| UP00239\_1 | 17 | TGAGGGGGATTAACTAT |
| UP00160\_1 | 17 | TGAGGGGGATTAACTAT |
| UP00208\_1 | 17 | TAGAGGGATTAAATTTC |
| UP00208\_2 | 17 | GATAATTAATCCCTCTT |
| UP00109\_1 | 15 | AAAAACGGATTATTG |
| UP00178\_1 | 17 | CGCGCTAATTAGGTATC |
| UP00237\_1 | 17 | CGTAATTAATTAATTGG |
| UP00229\_1 | 17 | GGAGGGGATTAATTTAT |
| UP00267\_1 | 17 | TGTAGGGATTAATTGTC |
| UP00247\_1 | 17 | TGAACTAATTAGCCCAC |
| UP00224\_1 | 16 | TGATTAATTAATTGAC |
| UP00248\_1 | 17 | CGAACTAATTAGTACTA |
| UP00185\_1 | 17 | TCACCCATCAATAATCA |
| UP00221\_1 | 16 | CAGCATTAATTAGTAG |
| UP00149\_1 | 17 | CGGAATTAATTAATAGG |
| UP00153\_1 | 17 | TTAGAGGGATTAACAAT |
| UP00125\_1 | 17 | TGAAGGGATTAATCATC |
| UP00265\_1 | 16 | AGGGGGATTAGCTGCC |
| UP00203\_1 | 16 | AAAGACCTGTCAATCC |
| UP00205\_1 | 16 | AAGCACCTGTCAATAT |
| UP00158\_1 | 17 | GATTAATTAATTAAGTC |
| UP00254\_1 | 16 | ATGTATTAATTAAGTA |
| UP00191\_1 | 16 | TTGTATGCAAATTAGA |
| UP00179\_1 | 16 | TTGTATGCAAATTAGA |
| UP00129\_1 | 17 | AATTAATTAATTAATTC |
| UP00128\_1 | 17 | GATAATTAATTAGTTTG |
| UP00211\_1 | 17 | AAAATATGCATAATAAA |
| UP00105\_1 | 17 | AATTAATTAATTAATTC |
| UP00118\_1 | 16 | AGTTATTAATGAGGTC |
| UP00146\_1 | 17 | GACGATAATGAGGTTGC |
| UP00146\_2 | 17 | AAACATAATGAGGTTGC |
| UP00172\_1 | 17 | CGAATTAATTAAGAAAC |
| UP00266\_1 | 17 | GTAACTAATTAACTACT |
| UP00136\_1 | 17 | AAAGCTAATTAGCGAAA |
| UP00253\_1 | 17 | TGCACTAATTAGCGCAC |
| UP00193\_1 | 17 | AAGACGCTGTAAAGCGA |
| UP00193\_2 | 17 | AGGACGCTGTAAAGGGA |
| UP00116\_1 | 17 | TGCCTTAATTAATGCTC |
| UP00257\_1 | 17 | CGCGTTAATTAATTGTG |
| UP00192\_1 | 17 | GATGGGGTATCATTTTT |
| UP00159\_1 | 17 | AATGGGGTATCACTTTT |
| UP00195\_1 | 17 | GATAGGGTATCACTTAT |
| UP00199\_1 | 17 | ATAAATGACACCTATCA |
| UP00008\_3 | 17 | AATAGGGTATCAATTAT |
| UP00008\_4 | 17 | AATAGGGTATCAATATT |
| UP00089\_3 | 17 | CCTTAGTTAACTAAAAT |
| UP00222\_1 | 17 | AGCTGTTAACTAGCCGT |
| UP00122\_1 | 17 | GATATTGACAGCTGCGT |
| UP00258\_1 | 16 | AACTAGCTGTCAATAC |
| UP00165\_1 | 16 | TAAGCCACTTGAAATT |
| UP00244\_1 | 17 | TAATTAATTAATAACTT |
| UP00142\_1 | 17 | CATAATTAATTAACGCG |
| UP00215\_1 | 16 | ACGTTAATTAACCCAG |
| UP00106\_1 | 16 | GTGCACTAATTAAGAC |
| UP00141\_1 | 17 | CGAGTTAATTAATAATT |

Random model letter frequencies
(from ./background):
  
A 0.241 C 0.259 G 0.259 T 0.241

---

**SECTION I: HIGH-SCORING MOTIF OCCURRENCES**


---

- There were
  179
  motif occurrences with a
  p-value less than
  0.0001.
- The p-value of a motif occurrence is defined as the
  probability of a random sequence of the same length as the motif
  matching that position of the sequence with as good or better a score.
- The score for the match of a position in a sequence to a motif
  is computed by summing the appropriate entries from each column of
  the position-dependent scoring matrix that represents the motif.
- The q-value of a motif occurrence is defined as the
  false discovery rate if the occurrence is accepted as significant.
- The table is sorted by increasing p-value.

| Motif | Sequence Name | Strand | Start | End | p-value | q-value | Matched Sequence |
| --- | --- | --- | --- | --- | --- | --- | --- |
| UP00406\_1 | chr9 | + | 115212586 | 115212601 | 5.54e-08 | 0.119 | `GCGCATCCGGATTTTG` |
| UP00406\_1 | chr15 | − | 88238416 | 88238431 | 3.89e-06 | 1 | `ATAGATCCGGATTCGC` |
| UP00406\_1 | chr21 | + | 45184273 | 45184288 | 4.02e-06 | 1 | `CCGCTTCCGGGTACTT` |
| UP00406\_1 | chr1 | + | 35419366 | 35419381 | 4.06e-06 | 1 | `CTACATCCGGGTCCTA` |
| UP00406\_1 | chr19 | + | 44594870 | 44594885 | 5.7e-06 | 1 | `CCAGTTCCGGATTCTC` |
| UP00406\_1 | chr15 | + | 88238416 | 88238431 | 6.1e-06 | 1 | `GCGAATCCGGATCTAT` |
| UP00406\_1 | chr17 | − | 59273969 | 59273984 | 7.25e-06 | 1 | `CTGGATCCGGAATCTC` |
| UP00406\_1 | chr4 | + | 152885448 | 152885463 | 7.43e-06 | 1 | `AGACATCCGGTTAACT` |
| UP00406\_1 | chr12 | + | 105275599 | 105275614 | 9.97e-06 | 1 | `CCACATCCGGGTTCCA` |
| UP00406\_1 | chr21 | + | 26029257 | 26029272 | 1.05e-05 | 1 | `GTGCTTCCGGGTCCCC` |
| UP00406\_1 | chr22 | + | 25336491 | 25336506 | 1.08e-05 | 1 | `CCACATCCGGTAGCTT` |
| UP00406\_1 | chr17 | + | 8227246 | 8227261 | 1.16e-05 | 1 | `CTGCTTCCGGTTCTGT` |
| UP00406\_1 | chr3 | + | 10237605 | 10237620 | 1.21e-05 | 1 | `TCACATCCTGTTTTTT` |
| UP00406\_1 | chr1 | − | 171448910 | 171448925 | 1.22e-05 | 1 | `GTGCATCCGGTTTCAA` |
| UP00406\_1 | chr6 | + | 33375207 | 33375222 | 1.27e-05 | 1 | `CCACATCCGGTTCCAG` |
| UP00406\_1 | chr15 | − | 43279727 | 43279742 | 1.27e-05 | 1 | `AGGCTTCCGGGTCCTC` |
| UP00406\_1 | chr2 | − | 178125778 | 178125793 | 1.39e-05 | 1 | `CCGCTTCCGGTTGCTC` |
| UP00406\_1 | chr19 | − | 60838134 | 60838149 | 1.39e-05 | 1 | `CAGCTTCCGGATTCGG` |
| UP00406\_1 | chr22 | − | 35588447 | 35588462 | 1.4e-05 | 1 | `CTGCTTCCGGAAGATT` |
| UP00406\_1 | chr11 | + | 131733407 | 131733422 | 1.56e-05 | 1 | `GAACATCCTGAATCTC` |
| UP00406\_1 | chr6 | − | 32253588 | 32253603 | 1.74e-05 | 1 | `CTTCATCCGGGTCCTT` |
| UP00406\_1 | chr13 | − | 97938971 | 97938986 | 1.77e-05 | 1 | `ATGATTCCGGGTTCTT` |
| UP00406\_1 | chr14 | − | 72013286 | 72013301 | 1.82e-05 | 1 | `CTGTTTCCGGATGTCC` |
| UP00406\_1 | chr3 | + | 116347656 | 116347671 | 1.88e-05 | 1 | `CTACTTCCTGATTCAT` |
| UP00406\_1 | chr14 | + | 57834761 | 57834776 | 1.88e-05 | 1 | `GCACATCCGGAGTCGT` |
| UP00406\_1 | chr6 | − | 26153800 | 26153815 | 1.96e-05 | 1 | `CAGCTTCCGGATCAGC` |
| UP00406\_1 | chr20 | − | 10363223 | 10363238 | 1.96e-05 | 1 | `AAAAATCCTGATTTTC` |
| UP00406\_1 | chr14 | + | 91409702 | 91409717 | 2.05e-05 | 1 | `CCACATCCTGTTTGTT` |
| UP00406\_1 | chr5 | + | 139907805 | 139907820 | 2.08e-05 | 1 | `TAGCTTCCGGGTGTTT` |
| UP00406\_1 | chr14 | + | 72013286 | 72013301 | 2.09e-05 | 1 | `GGACATCCGGAAACAG` |
| UP00406\_1 | chr19 | + | 54070622 | 54070637 | 2.12e-05 | 1 | `CCCCATCCGGATTCCC` |
| UP00406\_1 | chr15 | − | 61583758 | 61583773 | 2.15e-05 | 1 | `CGAGATCCGGGTTCTG` |
| UP00406\_1 | chr14 | + | 101412780 | 101412795 | 2.37e-05 | 1 | `GTGCTTCCTGATATTA` |
| UP00406\_1 | chr5 | + | 172504175 | 172504190 | 2.4e-05 | 1 | `CCACGTCCGGATCTGT` |
| UP00406\_1 | chr6 | − | 30818795 | 30818810 | 2.43e-05 | 1 | `CAGCATCCGGTTGCCG` |
| UP00406\_1 | chr16 | − | 10383659 | 10383674 | 2.46e-05 | 1 | `GCACTTCCTGAAATTT` |
| UP00406\_1 | chr9 | + | 91269464 | 91269479 | 2.52e-05 | 1 | `CCACATCCTCATTCTT` |
| UP00406\_1 | chr11 | + | 95588215 | 95588230 | 2.55e-05 | 1 | `CCACTTCCTGTTATTT` |
| UP00406\_1 | chr8 | + | 134564057 | 134564072 | 2.62e-05 | 1 | `ACACATCCGGGGACTT` |
| UP00406\_1 | chr7 | + | 101420970 | 101420985 | 2.65e-05 | 1 | `GTATTTCCGCATCTTT` |
| UP00406\_1 | chr16 | + | 17226602 | 17226617 | 2.65e-05 | 1 | `CTGTTTCCTGATCTTT` |
| UP00406\_1 | chr3 | − | 109112687 | 109112702 | 2.69e-05 | 1 | `CCACATCCTGTTCATC` |
| UP00406\_1 | chr17 | + | 39936599 | 39936614 | 2.78e-05 | 1 | `TCACTTCCGGGTGTCT` |
| UP00406\_1 | chrX | − | 12903825 | 12903840 | 2.82e-05 | 1 | `CCGGATCCGGCTTTCT` |
| UP00406\_1 | chr9 | − | 129253439 | 129253454 | 2.83e-05 | 1 | `CCGAATCCGGGTTCAT` |
| UP00406\_1 | chr17 | + | 5283305 | 5283320 | 2.85e-05 | 1 | `TCGAATCCGGATTCCG` |
| UP00406\_1 | chr6 | − | 24829500 | 24829515 | 2.89e-05 | 1 | `CTCCATCCGGGTGTTT` |
| UP00406\_1 | chr6 | − | 7825638 | 7825653 | 2.95e-05 | 1 | `GTGCTTCCGTGTTTTT` |
| UP00406\_1 | chr19 | + | 9799573 | 9799588 | 2.95e-05 | 1 | `CCGCTTCCGGTTCCTA` |
| UP00406\_1 | chr11 | + | 62177136 | 62177151 | 3.01e-05 | 1 | `TAGGATCCGGGTTTCT` |
| UP00406\_1 | chr8 | + | 57149657 | 57149672 | 3.09e-05 | 1 | `TTGCATCCGGGTCCCA` |
| UP00406\_1 | chr4 | − | 186012966 | 186012981 | 3.15e-05 | 1 | `GTGCTTCCTGTTTTCT` |
| UP00406\_1 | chr2 | − | 74535640 | 74535655 | 3.19e-05 | 1 | `TTACTTCCGGTTCATG` |
| UP00406\_1 | chr6 | + | 7862420 | 7862435 | 3.23e-05 | 1 | `TTACATCCTGTTGTTC` |
| UP00406\_1 | chr7 | − | 92277114 | 92277129 | 3.23e-05 | 1 | `GCACTTCCTGGTTTCT` |
| UP00406\_1 | chr1 | − | 152185276 | 152185291 | 3.29e-05 | 1 | `CTGGTTCCGGTTCCTC` |
| UP00406\_1 | chr6 | + | 151815113 | 151815128 | 3.31e-05 | 1 | `CTGCTTCCGGCTTTCG` |
| UP00406\_1 | chr16 | + | 29973969 | 29973984 | 3.31e-05 | 1 | `CCACATCCTGAACCCT` |
| UP00406\_1 | chr14 | − | 61198555 | 61198570 | 3.34e-05 | 1 | `AGACATCCTGTTTGTT` |
| UP00406\_1 | chr6 | + | 264367 | 264382 | 3.4e-05 | 1 | `CTACATCCTGTTGTCC` |
| UP00406\_1 | chr13 | − | 49837933 | 49837948 | 3.42e-05 | 1 | `CTGGATCCGGGTTAAC` |
| UP00406\_1 | chr9 | − | 115212586 | 115212601 | 3.56e-05 | 1 | `CAAAATCCGGATGCGC` |
| UP00406\_1 | chr1 | − | 28751997 | 28752012 | 3.79e-05 | 1 | `AGGCTTCCGGATCGCG` |
| UP00406\_1 | chr1 | − | 28374405 | 28374420 | 3.86e-05 | 1 | `GGGCATCCGGGGTTTC` |
| UP00406\_1 | chr1 | + | 84745260 | 84745275 | 3.89e-05 | 1 | `GTGTTTCCGGAAAGTT` |
| UP00406\_1 | chr1 | − | 110979434 | 110979449 | 3.89e-05 | 1 | `CCATTTCCTGATTCTC` |
| UP00406\_1 | chr20 | + | 51889747 | 51889762 | 4.06e-05 | 1 | `CTGCATCCTGCTCCTC` |
| UP00406\_1 | chrX | − | 38545548 | 38545563 | 4.11e-05 | 1 | `ATGAATCCTGATGCTC` |
| UP00406\_1 | chr19 | + | 63723060 | 63723075 | 4.45e-05 | 1 | `ACGTTTCCGGTATTTT` |
| UP00406\_1 | chr8 | + | 22518252 | 22518267 | 4.51e-05 | 1 | `GTGGTTCCGGGTGTCT` |
| UP00406\_1 | chr18 | + | 19735150 | 19735165 | 4.59e-05 | 1 | `CAGTATCCGGGATTCT` |
| UP00406\_1 | chr1 | + | 201597668 | 201597683 | 4.62e-05 | 1 | `TGGCATCCGGGAATTG` |
| UP00406\_1 | chr10 | − | 125964550 | 125964565 | 4.82e-05 | 1 | `GCGCTTCCGGCTCTGC` |
| UP00406\_1 | chr22 | + | 40172790 | 40172805 | 4.82e-05 | 1 | `ACGGATCCGGCTTCCC` |
| UP00406\_1 | chr14 | + | 68325523 | 68325538 | 4.85e-05 | 1 | `CTGGTTCCGGGTCTCC` |
| UP00406\_1 | chr15 | − | 64577352 | 64577367 | 4.94e-05 | 1 | `CAAAATCCTGATCATT` |
| UP00406\_1 | chr2 | + | 99164189 | 99164204 | 5.03e-05 | 1 | `GTGTATCCGGGAACCC` |
| UP00406\_1 | chr2 | + | 161269189 | 161269204 | 5.09e-05 | 1 | `TGGCATCCGGGACTTG` |
| UP00406\_1 | chr5 | + | 138925477 | 138925492 | 5.09e-05 | 1 | `TTGCTTCCTGTTTTTC` |
| UP00406\_1 | chr19 | − | 46508046 | 46508061 | 5.12e-05 | 1 | `CTCCATCCTGATTCTC` |
| UP00406\_1 | chr14 | + | 49598318 | 49598333 | 5.15e-05 | 1 | `TCACTTCCTGATTTTA` |
| UP00406\_1 | chr16 | − | 84494264 | 84494279 | 5.21e-05 | 1 | `CCACTTCCTGATTATA` |
| UP00406\_1 | chr17 | − | 53282406 | 53282421 | 5.25e-05 | 1 | `CCGCTTCCGGTTCCGG` |
| UP00406\_1 | chr21 | + | 46530617 | 46530632 | 5.28e-05 | 1 | `CGACTTCCGGTTCGCT` |
| UP00406\_1 | chr9 | − | 131841867 | 131841882 | 5.47e-05 | 1 | `CCACATCCTGCTTTGT` |
| UP00406\_1 | chr2 | + | 241149316 | 241149331 | 5.5e-05 | 1 | `GCACTTCCGGGTTGGG` |
| UP00406\_1 | chr12 | + | 6950326 | 6950341 | 5.57e-05 | 1 | `CAACTTCCGGTATTGT` |
| UP00406\_1 | chr19 | + | 6752676 | 6752691 | 5.63e-05 | 1 | `GCATTTCCGGTTTCCG` |
| UP00406\_1 | chr18 | + | 53448530 | 53448545 | 5.73e-05 | 1 | `CCACTTCCGCGTTTCT` |
| UP00406\_1 | chr11 | − | 82450785 | 82450800 | 5.8e-05 | 1 | `CTGCTTCCTGTTCTTG` |
| UP00406\_1 | chr19 | + | 60838134 | 60838149 | 5.84e-05 | 1 | `CCGAATCCGGAAGCTG` |
| UP00406\_1 | chr5 | − | 138937145 | 138937160 | 5.98e-05 | 1 | `TGGCATCCTGTTTTCT` |
| UP00406\_1 | chr12 | + | 67487896 | 67487911 | 5.98e-05 | 1 | `GGAGATCCGCATTTGT` |
| UP00406\_1 | chr17 | + | 60402019 | 60402034 | 6.05e-05 | 1 | `CTACATCCTCATCCCT` |
| UP00406\_1 | chrX | − | 48661309 | 48661324 | 6.15e-05 | 1 | `CCGCGTCCGGATTGAT` |
| UP00406\_1 | chr10 | + | 35455623 | 35455638 | 6.15e-05 | 1 | `CAGCTTCCGGTTTCCA` |
| UP00406\_1 | chr6 | + | 136652559 | 136652574 | 6.19e-05 | 1 | `GCGGTTCCGGGAATTT` |
| UP00406\_1 | chr12 | + | 109424463 | 109424478 | 6.26e-05 | 1 | `CAACATCCGGGCACCT` |
| UP00406\_1 | chr19 | + | 16310886 | 16310901 | 6.26e-05 | 1 | `ACACATCCTGACATCT` |
| UP00406\_1 | chr7 | + | 134506111 | 134506126 | 6.41e-05 | 1 | `ACACTTCCGGCTCCCG` |
| UP00406\_1 | chr19 | − | 11896893 | 11896908 | 6.41e-05 | 1 | `CCATTTCCGGATGAAC` |
| UP00406\_1 | chr10 | + | 27571092 | 27571107 | 6.45e-05 | 1 | `GCGCTTCCGGACACGT` |
| UP00406\_1 | chr6 | − | 133177275 | 133177290 | 6.48e-05 | 1 | `CTGTTTCCGGATTGAG` |
| UP00406\_1 | chr6 | + | 30818788 | 30818803 | 6.52e-05 | 1 | `CTACATCCGGCAACCG` |
| UP00406\_1 | chr16 | − | 84494224 | 84494239 | 6.56e-05 | 1 | `AGGGATCCGGAACATG` |
| UP00406\_1 | chr7 | + | 134506187 | 134506202 | 6.6e-05 | 1 | `TGGCATCCGGTTGGCT` |
| UP00406\_1 | chr6 | − | 31430541 | 31430556 | 6.64e-05 | 1 | `GTGTTTCCTGATCCTG` |
| UP00406\_1 | chr6 | − | 31430541 | 31430556 | 6.64e-05 | 1 | `GTGTTTCCTGATCCTG` |
| UP00406\_1 | chr6 | + | 42822060 | 42822075 | 6.64e-05 | 1 | `TTATATCCGGGATTGT` |
| UP00406\_1 | chr12 | + | 54798242 | 54798257 | 6.64e-05 | 1 | `ATGGTTCCGGGTCTGC` |
| UP00406\_1 | chr1 | − | 45760416 | 45760431 | 6.71e-05 | 1 | `GAGAATCCTGGTTTTC` |
| UP00406\_1 | chr17 | − | 35277433 | 35277448 | 6.71e-05 | 1 | `GCGCTTCCGGGCCTTC` |
| UP00406\_1 | chr22 | − | 36334419 | 36334434 | 6.71e-05 | 1 | `CCACTTCCGGAGCCCT` |
| UP00406\_1 | chr1 | + | 51198395 | 51198410 | 6.79e-05 | 1 | `GCGCACCCGGATACCT` |
| UP00406\_1 | chr7 | − | 126128434 | 126128449 | 6.83e-05 | 1 | `TTGCTTCCTGTTTCTC` |
| UP00406\_1 | chr12 | − | 6941304 | 6941319 | 6.87e-05 | 1 | `GCGCTTCCGGCTGTTA` |
| UP00406\_1 | chr17 | − | 5283305 | 5283320 | 6.99e-05 | 1 | `CGGAATCCGGATTCGA` |
| UP00406\_1 | chr17 | − | 33853650 | 33853665 | 7.03e-05 | 1 | `GTGCTTCCTGTTGTCT` |
| UP00406\_1 | chr6 | + | 42822350 | 42822365 | 7.07e-05 | 1 | `ATGCCTCCGGTTTTTT` |
| UP00406\_1 | chr19 | − | 56766324 | 56766339 | 7.15e-05 | 1 | `CCACTTCCGGTTTGGG` |
| UP00406\_1 | chr13 | + | 21587345 | 21587360 | 7.19e-05 | 1 | `TCACTTCCTGTTATTC` |
| UP00406\_1 | chr1 | − | 158849917 | 158849932 | 7.23e-05 | 1 | `TGGCATCCGCATCTTA` |
| UP00406\_1 | chr5 | + | 40870943 | 40870958 | 7.27e-05 | 1 | `CCTTATCCGGAATCTT` |
| UP00406\_1 | chr10 | − | 64083360 | 64083375 | 7.27e-05 | 1 | `TATCATCCTGATTTTT` |
| UP00406\_1 | chr17 | + | 35992311 | 35992326 | 7.27e-05 | 1 | `ATGCTTCCTGTTTTGC` |
| UP00406\_1 | chr17 | − | 38024526 | 38024541 | 7.31e-05 | 1 | `GAGCTTCCTGATATAG` |
| UP00406\_1 | chr6 | − | 30585106 | 30585121 | 7.4e-05 | 1 | `CAACTTCCTGGTTCCT` |
| UP00406\_1 | chr19 | + | 63679333 | 63679348 | 7.4e-05 | 1 | `CCACATCCGGGAAGCG` |
| UP00406\_1 | chr14 | − | 75023933 | 75023948 | 7.44e-05 | 1 | `CCACATCCTGGTTCCA` |
| UP00406\_1 | chr3 | + | 45705621 | 45705636 | 7.48e-05 | 1 | `GGGCTTCCGGGTACCA` |
| UP00406\_1 | chr11 | + | 47557097 | 47557112 | 7.52e-05 | 1 | `CCGCTTCCGGCTCCAC` |
| UP00406\_1 | chr19 | − | 54691477 | 54691492 | 7.61e-05 | 1 | `CAGCGTCCGGGTTTCC` |
| UP00406\_1 | chr15 | − | 72483549 | 72483564 | 7.65e-05 | 1 | `GAGCATCCTCATCTCT` |
| UP00406\_1 | chr16 | − | 30569246 | 30569261 | 7.78e-05 | 1 | `CATCTTCCGGTTCTTT` |
| UP00406\_1 | chr17 | − | 38794013 | 38794028 | 7.78e-05 | 1 | `TTAAATCCGGGTTTCA` |
| UP00406\_1 | chr3 | − | 198153688 | 198153703 | 7.83e-05 | 1 | `GCACTTCCGGGTGAGG` |
| UP00406\_1 | chr5 | + | 145542388 | 145542403 | 7.92e-05 | 1 | `AATCATCCGGCTCCTT` |
| UP00406\_1 | chr11 | + | 110727337 | 110727352 | 7.92e-05 | 1 | `CTGGATCCTGATTGAT` |
| UP00406\_1 | chr19 | + | 62483615 | 62483630 | 7.92e-05 | 1 | `GGACTTCCGCTTCTTC` |
| UP00406\_1 | chr6 | + | 7862362 | 7862377 | 8.01e-05 | 1 | `GTGCATCCTGAGTTAT` |
| UP00406\_1 | chr7 | + | 1510568 | 1510583 | 8.01e-05 | 1 | `CGACTTCCGGAACAAC` |
| UP00406\_1 | chr1 | − | 51198395 | 51198410 | 8.23e-05 | 1 | `AGGTATCCGGGTGCGC` |
| UP00406\_1 | chr6 | + | 119805740 | 119805755 | 8.33e-05 | 1 | `CTGTTTCCTGTTTTTC` |
| UP00406\_1 | chrX | − | 129054139 | 129054154 | 8.33e-05 | 1 | `CTGCATCCGCTTGTAC` |
| UP00406\_1 | chr15 | + | 29343922 | 29343937 | 8.37e-05 | 1 | `GGACTTCCTGTTTCTG` |
| UP00406\_1 | chr22 | + | 35588481 | 35588496 | 8.42e-05 | 1 | `GCACTTCCTGCTTTCT` |
| UP00406\_1 | chrX | + | 70760454 | 70760469 | 8.51e-05 | 1 | `AAACATCCTGCTGCTC` |
| UP00406\_1 | chr12 | − | 123965385 | 123965400 | 8.56e-05 | 1 | `GAGCGTCCTGATCCTT` |
| UP00406\_1 | chr14 | + | 80991101 | 80991116 | 8.7e-05 | 1 | `CCACATCCTCTTCTTT` |
| UP00406\_1 | chr15 | − | 61757498 | 61757513 | 8.7e-05 | 1 | `GCTCATCCGTATCCTT` |
| UP00406\_1 | chr19 | + | 44590078 | 44590093 | 8.7e-05 | 1 | `TGGCATCCGGAACCCA` |
| UP00406\_1 | chr8 | − | 145205495 | 145205510 | 8.75e-05 | 1 | `TCACTTCCGGTTCCGG` |
| UP00406\_1 | chr2 | + | 8360942 | 8360957 | 8.9e-05 | 1 | `ACACATCCTGGACCCT` |
| UP00406\_1 | chr8 | + | 72918605 | 72918620 | 8.9e-05 | 1 | `GCGCATCCGGGCACGC` |
| UP00406\_1 | chr18 | + | 45267733 | 45267748 | 8.9e-05 | 1 | `AGGCTTCCGGCTCTGC` |
| UP00406\_1 | chr2 | + | 174538933 | 174538948 | 9.15e-05 | 1 | `CAGCTTCCTGATTCAG` |
| UP00406\_1 | chr3 | − | 186699300 | 186699315 | 9.15e-05 | 1 | `GGGAATCCGGGTTCGG` |
| UP00406\_1 | chr16 | − | 30274323 | 30274338 | 9.15e-05 | 1 | `CTACTTCCGGCCATTT` |
| UP00406\_1 | chr19 | + | 11896893 | 11896908 | 9.15e-05 | 1 | `GTTCATCCGGAAATGG` |
| UP00406\_1 | chr18 | + | 55568358 | 55568373 | 9.25e-05 | 1 | `GGACATCCTGTTCTAG` |
| UP00406\_1 | chr2 | − | 73365460 | 73365475 | 9.3e-05 | 1 | `CCCCTTCCGGATTCAC` |
| UP00406\_1 | chr17 | − | 60550015 | 60550030 | 9.3e-05 | 1 | `AAACCTCCGGATTCCT` |
| UP00406\_1 | chr19 | − | 7676721 | 7676736 | 9.35e-05 | 1 | `CCACATCCGCACAATT` |
| UP00406\_1 | chr6 | + | 26307437 | 26307452 | 9.5e-05 | 1 | `CCGCGTCCGGACATTT` |
| UP00406\_1 | chr12 | − | 122025250 | 122025265 | 9.5e-05 | 1 | `GCGCTTCCGGTCCCTC` |
| UP00406\_1 | chr15 | + | 99493356 | 99493371 | 9.5e-05 | 1 | `GAAGATCCTGTTCATT` |
| UP00406\_1 | chr7 | + | 101719230 | 101719245 | 9.61e-05 | 1 | `ATACTTCCGGGCCATC` |
| UP00406\_1 | chr2 | + | 99163931 | 99163946 | 9.66e-05 | 1 | `GCGCTTCCGGGAAGTG` |
| UP00406\_1 | chr5 | + | 1369040 | 1369055 | 9.66e-05 | 1 | `GTGCTTCCTGAAAACT` |
| UP00406\_1 | chr15 | + | 88238253 | 88238268 | 9.66e-05 | 1 | `TCACTTCCGGTTGGTG` |
| UP00406\_1 | chr4 | − | 483048 | 483063 | 9.71e-05 | 1 | `GCGCTTCCGGCTCCAG` |
| UP00406\_1 | chr17 | + | 59273969 | 59273984 | 9.71e-05 | 1 | `GAGATTCCGGATCCAG` |
| UP00406\_1 | chr12 | + | 6923418 | 6923433 | 9.76e-05 | 1 | `GCGCTTCCGGCTGCGC` |
| UP00406\_1 | chr1 | + | 39054203 | 39054218 | 9.82e-05 | 1 | `GTGAATCCTGGTACCT` |
| UP00406\_1 | chr19 | − | 44594870 | 44594885 | 9.82e-05 | 1 | `GAGAATCCGGAACTGG` |
| UP00406\_1 | chr9 | + | 126056439 | 126056454 | 9.87e-05 | 1 | `CTGAATCCTGAAACTC` |
| UP00406\_1 | chr11 | − | 67796119 | 67796134 | 9.87e-05 | 1 | `CCACTTCCGGCTCGCC` |
| UP00406\_1 | chr15 | + | 70554379 | 70554394 | 9.98e-05 | 1 | `ATGTATCCGGGAGGTC` |
| UP00406\_1 | chr16 | − | 30274143 | 30274158 | 9.98e-05 | 1 | `CCACTTCCGGGCTTCC` |

---

**DEBUGGING INFORMATION**


---

Command line:

```
/ebi/sw/MEME/VM-cluster410/meme-versions/4.10.0/bin/fimo --parse-genomic-coord --verbosity 1 --oc fimo_out_11 --bgfile ./background --motif UP00406_1 db/uniprobe_mouse.meme ./Supplementary_Table_1.500bp.fa
```

Settings:

```
|  |  |  |
| --- | --- | --- |
| output directory = fimo_out_11 | MEME file name = db/uniprobe_mouse.meme | sequence file name = ./Supplementary_Table_1.500bp.fa |
| background file name = ./background | allow clobber = true | compute q-values = true |
| parse genomic coord. = true | text only = false | scan both strands = true |
| max sequence length = 250000000 | output threshold = 0.0001 | threshold type = p-value |
| max stored scores = 100000 | pseudocount = 0.1 | verbosity = 1 |
| selected motif = UP00406_1 |  |  |
```

This information can be useful in the event you wish to report a
problem with the FIMO software.

---

**Go to top**
