# Supplementary material for: The MEME Suite
Source: Nucleic Acids Res. 2015 May 7;43(Web Server issue):W39–49. doi: 10.1093/nar/gkv416 (PMC4489269; doi:10.1093/nar/gkv416)
Supplement: SUPPLEMENTARY DATA [file supp_gkv416_nar-00283-web-b-2015-File005.zip › case4/meme-chip/fimo_out_12/fimo.html]

FIMO Results


---

|  |  |  |
| --- | --- | --- |
| **Database and Motifs** | **High-scoring Motif Occurrences** | **Debugging Information** |

  
  

---

**FIMO - Motif search tool**


---

FIMO version 4.10.0,
(Release date: Wed May 21 10:35:36 2014 +1000)

For further information on how to interpret these results
or to get a copy of the FIMO software please access
http://meme.nbcr.net

If you use FIMO in your research, please cite the following paper:  
Charles E. Grant, Timothy L. Bailey, and William Stafford Noble,
"FIMO: Scanning for occurrences of a given motif",
*Bioinformatics*, **27**(7):1017-1018, 2011.
[full text]

---

**DATABASE AND MOTIFS**


---

DATABASE
./Supplementary\_Table\_1.500bp.fa  
Database contains
2776
sequences,
1388000
residues

MOTIFS
db/uniprobe\_mouse.meme
(nucleotide)

| MOTIF | WIDTH | BEST POSSIBLE MATCH |
| --- | --- | --- |
| UP00078\_1 | 17 | GGGTTTAATTAAAATTC |
| UP00059\_1 | 14 | CTAATATTGCTAAA |
| UP00099\_1 | 17 | CTCAGCAGCTGCTCCTG |
| UP00020\_1 | 16 | ACGATGACGTCATCGA |
| UP00012\_1 | 15 | TAATTCAATGAAGTG |
| UP00043\_1 | 16 | TCTTTCGAGGAATTTG |
| UP00050\_1 | 22 | GGAAGAGTCACGTGACCAATAC |
| UP00001\_1 | 15 | ATAAAGGCGCGCGAT |
| UP00003\_1 | 15 | ATAAGGGCGCGCGAT |
| UP00007\_1 | 14 | TCCGCCCCCGCATT |
| UP00015\_1 | 15 | AGGACCCGGAAGTAA |
| UP00407\_1 | 13 | TACAAGGAAGTAA |
| UP00068\_1 | 17 | TAAAAGGTGTGAAAATT |
| UP00079\_1 | 17 | TATTCAAGGTCATGCGA |
| UP00073\_1 | 17 | AAAAAGTAAACAAAGAC |
| UP00041\_1 | 16 | AAAGTAAACAAAAATT |
| UP00039\_1 | 17 | AAAAAGTAAACAAACCC |
| UP00025\_1 | 17 | AAAATGTAAACAAACAG |
| UP00061\_1 | 17 | TAAATGTAAACAAAGGT |
| UP00408\_1 | 17 | CAATACCGGAAGTGTAA |
| UP00032\_1 | 22 | TTTTTAGAGATAAGAAATAAAG |
| UP00080\_1 | 17 | TAAACTGATAAGAAGAT |
| UP00100\_1 | 17 | TATAGAGATAAGAATTG |
| UP00070\_1 | 16 | TCGTACCCGCATCATT |
| UP00024\_1 | 16 | TATCGACCCCCCACAG |
| UP00042\_1 | 17 | CAGATGTGCACATACGT |
| UP00084\_1 | 17 | GAGTGTACGTACGATGG |
| UP00055\_1 | 16 | ACTATGAATGAATGAT |
| UP00035\_1 | 16 | ACTATGCCAACCTACC |
| UP00066\_1 | 17 | CTTCAGGGGTCAATTGA |
| UP00391\_1 | 14 | TGGAGGTAATTAAC |
| UP00072\_1 | 16 | ATTTACGACAAATAGC |
| UP00086\_1 | 14 | GAGAACCGAAACTG |
| UP00018\_1 | 15 | CGTATCGAAACCAAA |
| UP00040\_1 | 15 | ATAAACCGAAACCAA |
| UP00011\_1 | 17 | CTGATCGAAACCAAAGT |
| UP00074\_1 | 15 | CAAAATCGAAACTAA |
| UP00103\_1 | 16 | CCGATGACGTCATCGT |
| UP00093\_1 | 16 | TCGACCCCGCCCCTAT |
| UP00067\_1 | 17 | AATCCCTTTGATCTATC |
| UP00045\_1 | 17 | AAATTTGCTGACTTAGC |
| UP00044\_1 | 15 | TAAAAATGCTGACTT |
| UP00060\_1 | 16 | TGACCACGTGGTCGGG |
| UP00097\_1 | 16 | GGGCCGTGTGCAAAAA |
| UP00092\_1 | 17 | ATGGAAACCGTTATTTT |
| UP00081\_1 | 17 | TTGAAAACCGTTAATTT |
| UP00036\_1 | 16 | GAAGAACAGGTGTCCG |
| UP00017\_1 | 17 | CTTAACCACTTAAGGAT |
| UP00009\_1 | 16 | TCTCAAAGGTCACGAG |
| UP00027\_1 | 16 | TTTTACAGTAGCAAAA |
| UP00052\_1 | 16 | ATGTACAGTAGCAAAG |
| UP00088\_1 | 16 | TTGGGGGCGCCCCTAG |
| UP00048\_1 | 16 | TCTCAAAGGTCACCTG |
| UP00098\_1 | 23 | TGTGACCCTTAGCAACCGATTAA |
| UP00056\_1 | 15 | TACCATAGCAACGGT |
| UP00076\_1 | 15 | CCGCATAGCAACGGA |
| UP00053\_1 | 17 | TGTCGTGACCCCTTAAT |
| UP00085\_1 | 14 | TTAAGAGGAAGTTA |
| UP00008\_1 | 17 | AATAGGGTATCATATAT |
| UP00000\_1 | 17 | CAAATCCAGACATCAGA |
| UP00030\_1 | 17 | ATAAGAACAAAGGACTA |
| UP00101\_1 | 14 | TAATTGTTCTAAAC |
| UP00096\_1 | 16 | TTAAGAACAATAATTT |
| UP00004\_1 | 16 | GCTAATTATAATTATC |
| UP00075\_1 | 17 | TAGTGAACAATAGATTT |
| UP00014\_1 | 15 | ATAAACAATTAATCA |
| UP00064\_1 | 16 | TTCAATTGTTCTAAAA |
| UP00069\_1 | 16 | AATCAATTCAATAATT |
| UP00071\_1 | 16 | TTTAATTATAATTAAG |
| UP00023\_1 | 16 | ATTGAACAATGGAATT |
| UP00062\_1 | 17 | AGAAGAACAAAGGACTA |
| UP00091\_1 | 16 | TTTAGAACAATAAAAT |
| UP00034\_1 | 22 | AATAAAGAACAATAGAATTTCA |
| UP00051\_1 | 17 | TTATCTATTGTTCTTTA |
| UP00049\_1 | 14 | ATTTTACGGAAAAT |
| UP00002\_1 | 17 | GGTCCCGCCCCCTTCTC |
| UP00406\_1 | 16 | GTACATCCGGATTTTT |
| UP00077\_1 | 14 | TTCCATATATGGAA |
| UP00016\_1 | 16 | TATAATTATAATATTC |
| UP00029\_1 | 16 | TCTTTATATATAAATA |
| UP00089\_1 | 17 | ACTTAGTTAACTAAAAA |
| UP00058\_1 | 17 | TATAGATCAAAGGAAAA |
| UP00054\_1 | 17 | TATAGATCAAAGGAAAA |
| UP00083\_1 | 17 | ATTTCCTTTGATCTATA |
| UP00005\_1 | 15 | ATTCCCTGAGGGGAA |
| UP00010\_1 | 14 | TTGCCCTAGGGCAT |
| UP00087\_1 | 15 | ATTGCCTGAGGCGAA |
| UP00028\_1 | 15 | ATTGCCTGAGGCGAT |
| UP00046\_1 | 17 | ATCCACAGGTGCGAAAA |
| UP00019\_1 | 17 | CTAAGGTTCTAGATCAC |
| UP00031\_1 | 17 | AATCGCACTGCATTCCG |
| UP00047\_1 | 15 | AAGCCCCCCAAAAAT |
| UP00037\_1 | 15 | AACAAACAACAAGAG |
| UP00094\_1 | 17 | TCTTTGGCGTACCCTAA |
| UP00065\_1 | 16 | TGGCGCGCGCGCCTGA |
| UP00082\_1 | 14 | TTATGTACTAATAA |
| UP00021\_1 | 15 | TCCCCCCCCCCCCCC |
| UP00033\_1 | 17 | TATTATGGGATGGATAA |
| UP00095\_1 | 17 | CGAACAGTGCTCACTAT |
| UP00022\_1 | 16 | CCCCCCCCCCCACTTG |
| UP00102\_1 | 14 | CACCCCCGGGGGGG |
| UP00057\_1 | 15 | CCCCCCCGGGGGGGT |
| UP00006\_1 | 15 | CCCCCCCGGGGGGGT |
| UP00026\_1 | 17 | TACATGTGCACATAAAA |
| UP00078\_2 | 15 | ACCCGTATCAAATTT |
| UP00059\_2 | 17 | CGTACAATACGAAATAA |
| UP00099\_2 | 16 | CTATCCCCGCCCTATT |
| UP00020\_2 | 14 | GAATGACGAATAAC |
| UP00012\_2 | 17 | TGATTGTTAACAGTTGG |
| UP00043\_2 | 16 | ATCCCCGCCCCTAAAA |
| UP00050\_2 | 23 | TGTCGTTACACGTGGAAGGCGGT |
| UP00001\_2 | 17 | CGTTCGGCGCCAAAAGG |
| UP00003\_2 | 17 | CGCTCGGCGCCAAAAGC |
| UP00007\_2 | 16 | TGCGGAGTGGGACTGG |
| UP00015\_2 | 16 | TAGTATTTCCGATCTT |
| UP00407\_2 | 17 | GTTCAAAAAAAAAATTC |
| UP00068\_2 | 16 | GCGGAGGTGTCGCCTC |
| UP00079\_2 | 17 | GGCGAGGGGTCAAGGGC |
| UP00073\_2 | 15 | AAAAATAACAAACGG |
| UP00041\_2 | 15 | ATGTCACAACAACAC |
| UP00039\_2 | 17 | AACACCAAAACAAAGGA |
| UP00025\_2 | 15 | CAAACAACAACACCT |
| UP00061\_2 | 16 | ATATCAAAACAAAACA |
| UP00408\_2 | 16 | CCGTCTTCCCCCTCAC |
| UP00032\_2 | 22 | TTTTGTAGATTTTATCGACTTA |
| UP00080\_2 | 17 | GACAGAGATATCAGTTT |
| UP00100\_2 | 17 | GCGGCGATATCGCAGCG |
| UP00070\_2 | 17 | TGCGCATAGGGGAGGAG |
| UP00024\_2 | 14 | AATATTAATAAAGA |
| UP00042\_2 | 16 | AGCGGCACACACGCAA |
| UP00084\_2 | 16 | TGGGCGACGTCGTTAA |
| UP00055\_2 | 17 | TGTTCCCATTGTGTACT |
| UP00035\_2 | 16 | GGGTGTGCCCAAAAGG |
| UP00066\_2 | 16 | TGCAAAAGTCCAATAT |
| UP00391\_2 | 14 | AAAAACCATTAAGG |
| UP00072\_2 | 16 | ATGGAAAGTCGTAAAA |
| UP00086\_2 | 14 | GGAGAAAGGTGCGA |
| UP00018\_2 | 15 | AGTATTCTCGGTTGC |
| UP00040\_2 | 15 | TTGATCGAGAATTCC |
| UP00011\_2 | 15 | ACCACTCTCGGTCAC |
| UP00074\_2 | 14 | GCAAAACATTACTA |
| UP00103\_2 | 16 | ATTGATGAGTCACCAA |
| UP00093\_2 | 17 | AAGCATACGCCCAACTT |
| UP00067\_2 | 16 | GAAGATCAATCACTTA |
| UP00045\_2 | 15 | CAATTGCAAAAATAT |
| UP00044\_2 | 15 | GAAAAAATTGCAAGG |
| UP00060\_2 | 14 | GTGCCACGCGACTG |
| UP00097\_2 | 14 | AAATAAGAAAAAAC |
| UP00092\_2 | 16 | CGACCAACTGCCATGC |
| UP00081\_2 | 15 | CGACCAACTGCCGTG |
| UP00036\_2 | 15 | AGCAACAGCCGCACC |
| UP00017\_2 | 17 | ACTCCAAGTACTTGGAA |
| UP00009\_2 | 16 | CGCGCCGGGTCACGTA |
| UP00027\_2 | 16 | ACATGCTACCTAATAC |
| UP00052\_2 | 16 | ACTTGCTACCTACACC |
| UP00088\_2 | 17 | GCTGGGGGGTACCCCTT |
| UP00048\_2 | 16 | AGAGCGGGGTCAAGTA |
| UP00098\_2 | 23 | ACTGACGCTTGGTTACCACAAAG |
| UP00056\_2 | 15 | TACCCTAGTTACCGA |
| UP00076\_2 | 17 | CTACTTGGATACGGAAT |
| UP00053\_2 | 16 | TCGCGAAGGTTGTACT |
| UP00085\_2 | 14 | CAAATTCCGGAACC |
| UP00008\_2 | 17 | ATGGGATATATCCGCCT |
| UP00000\_2 | 17 | TACGCCCCGCCACTCTG |
| UP00030\_2 | 14 | AAAATTGTTATGAA |
| UP00101\_2 | 16 | AAATAGACAAAGGAAT |
| UP00096\_2 | 17 | GTATTGGGTGGGTATTT |
| UP00004\_2 | 15 | CTCACACAATGGCGC |
| UP00075\_2 | 15 | TTGAATGAAATTCGA |
| UP00014\_2 | 17 | GACCACATTCATACAAT |
| UP00064\_2 | 16 | GGACTGAATTCATGCC |
| UP00069\_2 | 15 | CTATAATTGTTATCG |
| UP00071\_2 | 17 | CATCAATTGTTCCGCTA |
| UP00023\_2 | 16 | TAAGATTATAATACGG |
| UP00062\_2 | 17 | GGAAAAATTGTTAGGAA |
| UP00091\_2 | 15 | TATCATAATTAAGGA |
| UP00034\_2 | 22 | GTGCTAATTGTGTGTGTACGCT |
| UP00051\_2 | 14 | ACATTCATGACACG |
| UP00049\_2 | 15 | TCCGTCGCTTAAAAG |
| UP00002\_2 | 15 | CAAAGGCGTGGCCAG |
| UP00406\_2 | 16 | GATAACATCCTAGTAG |
| UP00077\_2 | 17 | GTTAAAAAAAAAAATTT |
| UP00016\_2 | 17 | TCACGGAACAATAGGTG |
| UP00029\_2 | 15 | CCGATTTAAGCGATC |
| UP00089\_2 | 14 | TTGCCCGGATTAGG |
| UP00058\_2 | 15 | AGCCGAAAAAAAAAT |
| UP00054\_2 | 15 | CCGTATTATAAACAA |
| UP00083\_2 | 16 | GAAGATCAATCACTAA |
| UP00005\_2 | 14 | TCACCTCTGGGCAG |
| UP00010\_2 | 15 | ATTGCCTCAGGCAAT |
| UP00087\_2 | 14 | CCGCCCAAGGGCAG |
| UP00028\_2 | 14 | TACTGGAAAAAAAA |
| UP00046\_2 | 17 | AAGGCCAGATGGTCCGG |
| UP00019\_2 | 15 | TATCATTAGAACGCT |
| UP00031\_2 | 16 | CAATCACTGGCAGAAT |
| UP00047\_2 | 17 | CTTAAGACCACCATTAC |
| UP00037\_2 | 17 | GTGGTTCAATAATTTTG |
| UP00094\_2 | 14 | TGTATATATATACC |
| UP00065\_2 | 14 | GCCGCGCAGTGCGT |
| UP00082\_2 | 16 | GAGCCCTTGTCCCTTG |
| UP00021\_2 | 17 | AGGAGACCCCCAATTTG |
| UP00033\_2 | 17 | TCACCCCGCCCCTAATT |
| UP00095\_2 | 17 | TACGAGACTCCTCTAAC |
| UP00022\_2 | 17 | AAATTCCCCCCGGAAGT |
| UP00102\_2 | 15 | CCACACAGCAGGAGA |
| UP00057\_2 | 15 | CCACACAGCAGGAGA |
| UP00006\_2 | 15 | GAGCACAGCAGGACA |
| UP00026\_2 | 16 | CGAAGCACACAAAATA |
| UP00108\_1 | 17 | TAAACTAATTAGCTGAG |
| UP00187\_1 | 17 | CGCATTAATTAATTACC |
| UP00152\_1 | 17 | GTCCATTAATTAATGGA |
| UP00228\_1 | 17 | CATAACCACTTAACAAC |
| UP00166\_1 | 16 | AACAACCAATTAATTC |
| UP00145\_1 | 16 | AAAAACCAATTAAGAA |
| UP00181\_1 | 16 | AAAGTAATTAGTGAAT |
| UP00151\_1 | 16 | TAAGTAATTAGTTATA |
| UP00138\_1 | 16 | CAGGTAATTACCTCAG |
| UP00209\_1 | 17 | CGAATTAATTAATCACC |
| UP00209\_2 | 17 | CGCATTAATTAATTGGC |
| UP00240\_1 | 16 | TAAGGTAATAAAATTA |
| UP00133\_1 | 16 | AACGGTAATAAAATTT |
| UP00198\_1 | 14 | ATGATCGAATCAAA |
| UP00176\_1 | 16 | CGTTGGGGATTAGCCT |
| UP00219\_1 | 17 | ACCGGTTGATCACCTGA |
| UP00219\_2 | 15 | TAATGATGATCACTA |
| UP00255\_1 | 17 | TAATTAATTAATAATTA |
| UP00218\_1 | 16 | TTTAATTAATTAATTC |
| UP00202\_1 | 14 | CTGAGGTAATTAAT |
| UP00126\_1 | 16 | GGAATAATTACTTCAG |
| UP00154\_1 | 17 | TCGCGATAATTACCGAC |
| UP00110\_1 | 17 | TCGCTATAATTACCGAC |
| UP00230\_1 | 16 | GGGGTAATTAGCTCTG |
| UP00111\_1 | 17 | TGAACCGGATTAATGAA |
| UP00232\_1 | 17 | TAAATAGATACCCCATA |
| UP00143\_1 | 17 | GGAAGGGATTAATTATC |
| UP00227\_1 | 17 | CGACCCAATCAACGGTG |
| UP00201\_1 | 17 | ACCACTAATTAGTGGAC |
| UP00167\_1 | 16 | GCGAACTAATTAATGC |
| UP00163\_1 | 17 | TGCACTAATTAGTGGAA |
| UP00251\_1 | 17 | ATCCATTAATTAATTGA |
| UP00162\_1 | 17 | AGAACTAATTAGTGGAC |
| UP00132\_1 | 17 | CACCGCTAATTAGCGTT |
| UP00204\_1 | 17 | TGCCACTAATTAGTGTA |
| UP00131\_1 | 17 | AGCGCTAATTAGCGATT |
| UP00112\_1 | 17 | AATCGTTAATCCCTTTA |
| UP00127\_1 | 16 | AGGTTAATTAGCTGAT |
| UP00148\_1 | 17 | AAGGCGAAATCATCGCA |
| UP00225\_1 | 15 | CCATAATTAATTACA |
| UP00123\_1 | 16 | GTACTAATTAGTGGCG |
| UP00161\_1 | 17 | GAAAACTAGTTAACATC |
| UP00104\_1 | 17 | ACAAGCAATTAATGAAT |
| UP00155\_1 | 17 | ACAAGCAATTAAAGAAT |
| UP00157\_1 | 17 | ACAAGCAATTAAAGAAT |
| UP00114\_1 | 17 | AAAACATCGTTTTTAAG |
| UP00264\_1 | 16 | CTGAGCTAATTACCGT |
| UP00217\_1 | 16 | TAGGTAATAAAATTCA |
| UP00246\_1 | 16 | TAAAGTCGTAAAACAT |
| UP00183\_1 | 16 | AAAGCTCGTAAAATTT |
| UP00174\_1 | 16 | AAGGTAATTAGCTCAT |
| UP00391\_3 | 14 | TTGAGGTAATTAGT |
| UP00196\_1 | 17 | GATTATTAATTAACTTG |
| UP00189\_1 | 16 | ACGGTAATTAGCTCAG |
| UP00182\_1 | 16 | AAGGTAATTACCTAAT |
| UP00164\_1 | 17 | CGAGTTAATTAATAAGC |
| UP00164\_2 | 16 | GTAGTAATTAATGGAA |
| UP00213\_1 | 17 | ACGGCCATAAAATTAAT |
| UP00134\_1 | 16 | AACCCAATAAAATTCG |
| UP00137\_1 | 17 | TGAGCTAATTAGTTGGA |
| UP00144\_1 | 17 | CGCGTTAATTAATTACC |
| UP00214\_1 | 16 | ACGGTAATTAGCTCAT |
| UP00259\_1 | 16 | TATTGGTAATTACCTT |
| UP00206\_1 | 16 | GTAGTAATTAATGCAA |
| UP00263\_1 | 16 | ACCGGCAATTAATAAA |
| UP00207\_1 | 16 | GGAGCCATAAAATTCG |
| UP00245\_1 | 16 | TAAAGTCGTAAAACGT |
| UP00235\_1 | 16 | TAAAGTCGTAAAATAG |
| UP00135\_1 | 17 | TTAGGTCGTAAAATTTC |
| UP00173\_1 | 16 | AAAGCTCGTAAAATTT |
| UP00113\_1 | 17 | CGAATTAATTAACAATA |
| UP00252\_1 | 17 | CGAATTAATTAATTACT |
| UP00260\_1 | 17 | CAAATTAATTAATAAAA |
| UP00242\_1 | 16 | TTGGGGTAATTAACGT |
| UP00197\_1 | 16 | GGAGGTCATTAATTAT |
| UP00140\_1 | 17 | TAAACTAATTAGCTGTA |
| UP00121\_1 | 17 | AATGCAATAAAATTTAT |
| UP00117\_1 | 17 | TAAGGTCGTAAAATCCT |
| UP00177\_1 | 17 | CAAGGTCGTAAAATCTT |
| UP00180\_1 | 16 | CTACCAATAAAATTCT |
| UP00241\_1 | 16 | TTGAGTTAATTAACCT |
| UP00168\_1 | 17 | TAATTAATTAATGGCTA |
| UP00124\_1 | 16 | AAGGTAATTAGCTCAT |
| UP00236\_1 | 17 | TAAATACATGTAAAATT |
| UP00223\_1 | 17 | AAAATACATGTAATACT |
| UP00223\_2 | 17 | AATATACATGTAATATT |
| UP00194\_1 | 17 | AATATACATGTAAAACA |
| UP00250\_1 | 17 | TATATACATGTAAAATT |
| UP00150\_1 | 17 | AAAATACATGTAAAAAT |
| UP00170\_1 | 16 | CAAAATCAATTAATTT |
| UP00243\_1 | 16 | ACTCCTAATTAGTCGT |
| UP00120\_1 | 17 | TGCATTAATTAATGCGA |
| UP00262\_1 | 17 | CGAATTAATTAATAATG |
| UP00115\_1 | 17 | TAAACTAATTAGTGAAC |
| UP00130\_1 | 17 | GTAATTAATTAAATAAT |
| UP00261\_1 | 17 | TAAACTAATTAGCTTTG |
| UP00212\_1 | 17 | CGAATTAATTAAATACT |
| UP00256\_1 | 17 | GAGCGTTAATTAATGTA |
| UP00256\_2 | 17 | TCCACTAATTAGCGGTT |
| UP00184\_1 | 17 | ACCCCTAATTAGCGGTG |
| UP00175\_1 | 17 | CCCATTAATTAATCACC |
| UP00188\_1 | 17 | CGAATTAATTAAAAACC |
| UP00169\_1 | 17 | AGTTTTTAATTAATTTG |
| UP00186\_1 | 16 | AAGGAGCTGTCAATAC |
| UP00233\_1 | 16 | GAGGTAATTACCTCAG |
| UP00226\_1 | 16 | AAAGACCTGTCAATAC |
| UP00210\_1 | 16 | AATTACCTGTCAATAC |
| UP00234\_1 | 16 | TGCAACTAATTAATTC |
| UP00156\_1 | 17 | GAAGACCAATTAGCGCT |
| UP00171\_1 | 16 | CAAAACCAATTAATTT |
| UP00220\_1 | 17 | TGCGCTAATTAGTGGGA |
| UP00139\_1 | 17 | GTGCACTAATTAGTGCA |
| UP00231\_1 | 17 | TTAACCACTTGAAAATT |
| UP00190\_1 | 16 | CTTTAAGTACTTAATG |
| UP00107\_1 | 16 | TAAGCCACTTGAAATT |
| UP00249\_1 | 16 | TAAGCCACTTGAATTT |
| UP00147\_1 | 16 | TAAGCCACTTAACATT |
| UP00119\_1 | 17 | TTTTAAGTACTTAAATT |
| UP00017\_3 | 17 | TACTAAGTACTTAAATG |
| UP00200\_1 | 17 | GAAAATTAATTACTTCG |
| UP00200\_2 | 16 | AGTAATTAATTACTTC |
| UP00238\_1 | 17 | GATAATTAATTACTTTG |
| UP00216\_1 | 17 | TTAAGGGGATTAACTAC |
| UP00239\_1 | 17 | TGAGGGGGATTAACTAT |
| UP00160\_1 | 17 | TGAGGGGGATTAACTAT |
| UP00208\_1 | 17 | TAGAGGGATTAAATTTC |
| UP00208\_2 | 17 | GATAATTAATCCCTCTT |
| UP00109\_1 | 15 | AAAAACGGATTATTG |
| UP00178\_1 | 17 | CGCGCTAATTAGGTATC |
| UP00237\_1 | 17 | CGTAATTAATTAATTGG |
| UP00229\_1 | 17 | GGAGGGGATTAATTTAT |
| UP00267\_1 | 17 | TGTAGGGATTAATTGTC |
| UP00247\_1 | 17 | TGAACTAATTAGCCCAC |
| UP00224\_1 | 16 | TGATTAATTAATTGAC |
| UP00248\_1 | 17 | CGAACTAATTAGTACTA |
| UP00185\_1 | 17 | TCACCCATCAATAATCA |
| UP00221\_1 | 16 | CAGCATTAATTAGTAG |
| UP00149\_1 | 17 | CGGAATTAATTAATAGG |
| UP00153\_1 | 17 | TTAGAGGGATTAACAAT |
| UP00125\_1 | 17 | TGAAGGGATTAATCATC |
| UP00265\_1 | 16 | AGGGGGATTAGCTGCC |
| UP00203\_1 | 16 | AAAGACCTGTCAATCC |
| UP00205\_1 | 16 | AAGCACCTGTCAATAT |
| UP00158\_1 | 17 | GATTAATTAATTAAGTC |
| UP00254\_1 | 16 | ATGTATTAATTAAGTA |
| UP00191\_1 | 16 | TTGTATGCAAATTAGA |
| UP00179\_1 | 16 | TTGTATGCAAATTAGA |
| UP00129\_1 | 17 | AATTAATTAATTAATTC |
| UP00128\_1 | 17 | GATAATTAATTAGTTTG |
| UP00211\_1 | 17 | AAAATATGCATAATAAA |
| UP00105\_1 | 17 | AATTAATTAATTAATTC |
| UP00118\_1 | 16 | AGTTATTAATGAGGTC |
| UP00146\_1 | 17 | GACGATAATGAGGTTGC |
| UP00146\_2 | 17 | AAACATAATGAGGTTGC |
| UP00172\_1 | 17 | CGAATTAATTAAGAAAC |
| UP00266\_1 | 17 | GTAACTAATTAACTACT |
| UP00136\_1 | 17 | AAAGCTAATTAGCGAAA |
| UP00253\_1 | 17 | TGCACTAATTAGCGCAC |
| UP00193\_1 | 17 | AAGACGCTGTAAAGCGA |
| UP00193\_2 | 17 | AGGACGCTGTAAAGGGA |
| UP00116\_1 | 17 | TGCCTTAATTAATGCTC |
| UP00257\_1 | 17 | CGCGTTAATTAATTGTG |
| UP00192\_1 | 17 | GATGGGGTATCATTTTT |
| UP00159\_1 | 17 | AATGGGGTATCACTTTT |
| UP00195\_1 | 17 | GATAGGGTATCACTTAT |
| UP00199\_1 | 17 | ATAAATGACACCTATCA |
| UP00008\_3 | 17 | AATAGGGTATCAATTAT |
| UP00008\_4 | 17 | AATAGGGTATCAATATT |
| UP00089\_3 | 17 | CCTTAGTTAACTAAAAT |
| UP00222\_1 | 17 | AGCTGTTAACTAGCCGT |
| UP00122\_1 | 17 | GATATTGACAGCTGCGT |
| UP00258\_1 | 16 | AACTAGCTGTCAATAC |
| UP00165\_1 | 16 | TAAGCCACTTGAAATT |
| UP00244\_1 | 17 | TAATTAATTAATAACTT |
| UP00142\_1 | 17 | CATAATTAATTAACGCG |
| UP00215\_1 | 16 | ACGTTAATTAACCCAG |
| UP00106\_1 | 16 | GTGCACTAATTAAGAC |
| UP00141\_1 | 17 | CGAGTTAATTAATAATT |

Random model letter frequencies
(from ./background):
  
A 0.241 C 0.259 G 0.259 T 0.241

---

**SECTION I: HIGH-SCORING MOTIF OCCURRENCES**


---

- There were
  379
  motif occurrences with a
  p-value less than
  0.0001.
- The p-value of a motif occurrence is defined as the
  probability of a random sequence of the same length as the motif
  matching that position of the sequence with as good or better a score.
- The score for the match of a position in a sequence to a motif
  is computed by summing the appropriate entries from each column of
  the position-dependent scoring matrix that represents the motif.
- The q-value of a motif occurrence is defined as the
  false discovery rate if the occurrence is accepted as significant.
- The table is sorted by increasing p-value.

| Motif | Sequence Name | Strand | Start | End | p-value | q-value | Matched Sequence |
| --- | --- | --- | --- | --- | --- | --- | --- |
| UP00053\_1 | chr2 | + | 231444145 | 231444161 | 1.79e-07 | 0.286 | `CCACTTGACCCCTAAAT` |
| UP00053\_1 | chr6 | + | 44330156 | 44330172 | 2.68e-07 | 0.286 | `TCTCTTGACCCCAAACT` |
| UP00053\_1 | chr1 | − | 148801603 | 148801619 | 9.33e-07 | 0.527 | `CGCATTGACCCCATAAT` |
| UP00053\_1 | chr7 | + | 101719100 | 101719116 | 1.9e-06 | 0.527 | `AGGTGTGACCCCTCAAC` |
| UP00053\_1 | chr11 | − | 72765941 | 72765957 | 2.98e-06 | 0.527 | `CCACTTGACCCCACACG` |
| UP00053\_1 | chr11 | + | 65076595 | 65076611 | 3.02e-06 | 0.527 | `TCATGTGACCCCTGAAA` |
| UP00053\_1 | chr17 | + | 57525929 | 57525945 | 3.11e-06 | 0.527 | `TCTCTTGACCCCATAGC` |
| UP00053\_1 | chr4 | − | 186018574 | 186018590 | 3.31e-06 | 0.527 | `TGTCTTGACCCCTCCTC` |
| UP00053\_1 | chr6 | − | 33352621 | 33352637 | 3.49e-06 | 0.527 | `CTAGATGACCCCTTAAA` |
| UP00053\_1 | chr10 | + | 45276601 | 45276617 | 3.57e-06 | 0.527 | `AGCCATGACCTCATGAT` |
| UP00053\_1 | chr1 | − | 32065523 | 32065539 | 3.8e-06 | 0.527 | `TCCCTTGACCTCATGAC` |
| UP00053\_1 | chr19 | − | 12764601 | 12764617 | 3.89e-06 | 0.527 | `TCCCTTGACCCCAAGCA` |
| UP00053\_1 | chr1 | − | 32488421 | 32488437 | 4.14e-06 | 0.527 | `GGCCTTGACCCCACCAG` |
| UP00053\_1 | chr17 | + | 63747068 | 63747084 | 4.19e-06 | 0.527 | `GTCTGTGACCCCAAATT` |
| UP00053\_1 | chr19 | + | 11896619 | 11896635 | 4.73e-06 | 0.527 | `TCCTGTGACCCCGTGCA` |
| UP00053\_1 | chr8 | − | 103611880 | 103611896 | 4.84e-06 | 0.527 | `GGACTTGACCTCATGAG` |
| UP00053\_1 | chr3 | + | 58002815 | 58002831 | 4.89e-06 | 0.527 | `TCAGTTGACCCCATTCT` |
| UP00053\_1 | chr12 | − | 52965691 | 52965707 | 5.88e-06 | 0.527 | `TTGTGTGACCCCTTGTG` |
| UP00053\_1 | chr6 | + | 31431045 | 31431061 | 6.21e-06 | 0.527 | `AGATATGACCCCTCATC` |
| UP00053\_1 | chr14 | + | 57834722 | 57834738 | 6.41e-06 | 0.527 | `CTTCCTGACCCCTCAAT` |
| UP00053\_1 | chr19 | − | 1046664 | 1046680 | 6.84e-06 | 0.527 | `CCCCTTGACCCCATCCA` |
| UP00053\_1 | chr1 | + | 118008473 | 118008489 | 6.99e-06 | 0.527 | `AGTCATGACCTCATCAT` |
| UP00053\_1 | chr6 | − | 26070801 | 26070817 | 7.13e-06 | 0.527 | `TCATGTGACCCCAACAC` |
| UP00053\_1 | chr16 | + | 8937711 | 8937727 | 7.99e-06 | 0.527 | `TCTCTTGACCTCTCATC` |
| UP00053\_1 | chr16 | − | 2141327 | 2141343 | 8.32e-06 | 0.527 | `ACTCATGACCCCACCAG` |
| UP00053\_1 | chr2 | − | 233632669 | 233632685 | 8.83e-06 | 0.527 | `CTCTGTGACCCCATTAC` |
| UP00053\_1 | chr8 | + | 67507183 | 67507199 | 8.83e-06 | 0.527 | `ACAGGTGACCCCGCGAA` |
| UP00053\_1 | chr20 | − | 23262908 | 23262924 | 8.83e-06 | 0.527 | `ACCCTTGACCTCAGAAT` |
| UP00053\_1 | chr3 | − | 99738195 | 99738211 | 9.09e-06 | 0.527 | `ACTTGTGACCCCTAGTA` |
| UP00053\_1 | chr13 | + | 35518795 | 35518811 | 9.54e-06 | 0.527 | `TGTTATGACCCCATTCC` |
| UP00053\_1 | chr10 | + | 13805635 | 13805651 | 1e-05 | 0.527 | `TTAGATGACCCCGAAAA` |
| UP00053\_1 | chr18 | + | 44733449 | 44733465 | 1e-05 | 0.527 | `TTCCTTGACCCCAGGTC` |
| UP00053\_1 | chr1 | + | 148125036 | 148125052 | 1.04e-05 | 0.527 | `GTTCTTGACCCCACCCC` |
| UP00053\_1 | chr6 | − | 16581727 | 16581743 | 1.05e-05 | 0.527 | `CCCTTTGACCTCAAAAT` |
| UP00053\_1 | chr22 | − | 39141579 | 39141595 | 1.1e-05 | 0.527 | `CCAGATGACCCCGAGAT` |
| UP00053\_1 | chr13 | − | 97939245 | 97939261 | 1.11e-05 | 0.527 | `GTCTGTGACCCCGTGTG` |
| UP00053\_1 | chr20 | + | 36896540 | 36896556 | 1.11e-05 | 0.527 | `TTTGTTGACCTCATGTT` |
| UP00053\_1 | chr5 | − | 40871179 | 40871195 | 1.2e-05 | 0.527 | `CCTCTTGACCCCACCTA` |
| UP00053\_1 | chr10 | − | 26816561 | 26816577 | 1.2e-05 | 0.527 | `TCTCGTGACCTCTCCCT` |
| UP00053\_1 | chr10 | + | 90165765 | 90165781 | 1.2e-05 | 0.527 | `CTTCATGACCCCTACTT` |
| UP00053\_1 | chr14 | + | 80991006 | 80991022 | 1.2e-05 | 0.527 | `CCCCATGACCCCTACAC` |
| UP00053\_1 | chr11 | + | 65076748 | 65076764 | 1.22e-05 | 0.527 | `TCATGTGACCCCGGACC` |
| UP00053\_1 | chr17 | − | 73652414 | 73652430 | 1.22e-05 | 0.527 | `TCTGTTGACCCCTCGGT` |
| UP00053\_1 | chr7 | − | 130459590 | 130459606 | 1.27e-05 | 0.527 | `GTCTGTGACCTCTTGAC` |
| UP00053\_1 | chr2 | + | 12114943 | 12114959 | 1.29e-05 | 0.527 | `GCATGTGACCTCGAAAT` |
| UP00053\_1 | chr4 | + | 121207794 | 121207810 | 1.29e-05 | 0.527 | `CCATGTGACCCCTAGTG` |
| UP00053\_1 | chr11 | − | 601236 | 601252 | 1.33e-05 | 0.527 | `AGGCTTGACCCCAGGCC` |
| UP00053\_1 | chr15 | − | 82440512 | 82440528 | 1.37e-05 | 0.527 | `GGAGGTGAACCCATAAT` |
| UP00053\_1 | chr6 | + | 30569852 | 30569868 | 1.38e-05 | 0.527 | `TCCCATGACCCTTTAAC` |
| UP00053\_1 | chr8 | − | 67755365 | 67755381 | 1.38e-05 | 0.527 | `CCTAGTGACCCCATGCA` |
| UP00053\_1 | chr19 | + | 16310635 | 16310651 | 1.42e-05 | 0.527 | `AACCTTGACCCCAGGTT` |
| UP00053\_1 | chr3 | − | 17168013 | 17168029 | 1.49e-05 | 0.527 | `TGTTATGACCCCTCCTG` |
| UP00053\_1 | chr17 | − | 8139786 | 8139802 | 1.51e-05 | 0.527 | `TTATGTGACCCCACCCG` |
| UP00053\_1 | chr4 | − | 54309498 | 54309514 | 1.53e-05 | 0.527 | `AGCTGTGACCCCTCAGC` |
| UP00053\_1 | chr9 | − | 125939861 | 125939877 | 1.55e-05 | 0.527 | `AAAGTTGACCCCAAGAC` |
| UP00053\_1 | chr7 | − | 101719114 | 101719130 | 1.56e-05 | 0.527 | `GGCTGTGACCTCGTGTT` |
| UP00053\_1 | chr7 | + | 135694503 | 135694519 | 1.56e-05 | 0.527 | `ACAGATGACCCCAGAAA` |
| UP00053\_1 | chr17 | + | 59933080 | 59933096 | 1.56e-05 | 0.527 | `TCTCTTGACCTCACCTT` |
| UP00053\_1 | chr10 | − | 80514224 | 80514240 | 1.58e-05 | 0.527 | `CTCTTTGACCCCAACCT` |
| UP00053\_1 | chr22 | − | 27518523 | 27518539 | 1.58e-05 | 0.527 | `CCAGCTGACCCCATGAT` |
| UP00053\_1 | chr22 | − | 40558452 | 40558468 | 1.58e-05 | 0.527 | `CGCCGTGACCTCGCGCT` |
| UP00053\_1 | chr2 | − | 65635618 | 65635634 | 1.62e-05 | 0.527 | `GGGCTTGACCTCTTCTT` |
| UP00053\_1 | chr3 | + | 4994145 | 4994161 | 1.62e-05 | 0.527 | `GGCAGTGACCCCATTTT` |
| UP00053\_1 | chr1 | − | 40929771 | 40929787 | 1.66e-05 | 0.527 | `AGTTTTGACCCCGCCCC` |
| UP00053\_1 | chr20 | + | 45725816 | 45725832 | 1.68e-05 | 0.527 | `ATCAGTGACCCCATGTC` |
| UP00053\_1 | chr1 | − | 101646224 | 101646240 | 1.7e-05 | 0.527 | `TTTCCTGACCCCTCATC` |
| UP00053\_1 | chr2 | − | 231549798 | 231549814 | 1.72e-05 | 0.527 | `TGGCATGACCTCTGGAT` |
| UP00053\_1 | chr12 | + | 6425266 | 6425282 | 1.74e-05 | 0.527 | `CTCTATGACCCCAGATT` |
| UP00053\_1 | chr7 | − | 4689345 | 4689361 | 1.79e-05 | 0.527 | `AGGGGTGACCCCAGGAG` |
| UP00053\_1 | chr12 | + | 122134565 | 122134581 | 1.79e-05 | 0.527 | `TGGGGTGACCCCGGGAA` |
| UP00053\_1 | chr15 | − | 38191514 | 38191530 | 1.87e-05 | 0.527 | `CTCCCTGACCCCTTTAT` |
| UP00053\_1 | chr12 | + | 122134646 | 122134662 | 1.9e-05 | 0.527 | `GATGGTGACCCCAGAAG` |
| UP00053\_1 | chr1 | + | 28776568 | 28776584 | 1.91e-05 | 0.527 | `TTCCCTGACCCCATCCT` |
| UP00053\_1 | chr1 | + | 201597863 | 201597879 | 1.94e-05 | 0.527 | `GTCTGTGACCTCAGAAT` |
| UP00053\_1 | chr20 | − | 56659861 | 56659877 | 1.97e-05 | 0.527 | `CCTCCTGACCCCTTCCT` |
| UP00053\_1 | chr5 | − | 43639515 | 43639531 | 1.99e-05 | 0.527 | `CGAAATGACCCCGTACG` |
| UP00053\_1 | chr11 | + | 65112660 | 65112676 | 2e-05 | 0.527 | `CCCCATGACCCCGACAC` |
| UP00053\_1 | chr14 | − | 102306336 | 102306352 | 2.03e-05 | 0.527 | `CCCAGTGACCCCACATC` |
| UP00053\_1 | chr8 | − | 29253704 | 29253720 | 2.08e-05 | 0.527 | `CTTAGTGACCCCATTAA` |
| UP00053\_1 | chr17 | − | 38801022 | 38801038 | 2.08e-05 | 0.527 | `TACCGTGACCTCAGGAT` |
| UP00053\_1 | chr1 | − | 201557099 | 201557115 | 2.1e-05 | 0.527 | `TGACCTGACCCCACCCT` |
| UP00053\_1 | chr14 | + | 102855453 | 102855469 | 2.1e-05 | 0.527 | `GTACCTGACCCCAAAAA` |
| UP00053\_1 | chr3 | + | 126342442 | 126342458 | 2.19e-05 | 0.527 | `TTTTTTGACCTCAGAAG` |
| UP00053\_1 | chr13 | + | 45862325 | 45862341 | 2.19e-05 | 0.527 | `AGCTTTGACCCCAGTTT` |
| UP00053\_1 | chr15 | + | 99493420 | 99493436 | 2.19e-05 | 0.527 | `CCTCCTGACCCCTAGAC` |
| UP00053\_1 | chr19 | + | 2036421 | 2036437 | 2.21e-05 | 0.527 | `TGGTATGACCCCTCCCC` |
| UP00053\_1 | chr7 | + | 129903864 | 129903880 | 2.32e-05 | 0.527 | `TCCCCTGACCCCAGAAA` |
| UP00053\_1 | chr3 | − | 15378228 | 15378244 | 2.34e-05 | 0.527 | `AGGTTTGACCCCAGCCT` |
| UP00053\_1 | chr4 | − | 25694981 | 25694997 | 2.36e-05 | 0.527 | `TGTCTTGACCTTTTAAG` |
| UP00053\_1 | chr5 | + | 40864420 | 40864436 | 2.39e-05 | 0.527 | `TCTCGTGACCTCTTGGC` |
| UP00053\_1 | chr5 | − | 78089855 | 78089871 | 2.39e-05 | 0.527 | `CTTTTTGACCCCAAGGT` |
| UP00053\_1 | chr8 | − | 130634154 | 130634170 | 2.43e-05 | 0.527 | `TTTCATGACCTCGTGTA` |
| UP00053\_1 | chr12 | + | 112095585 | 112095601 | 2.43e-05 | 0.527 | `ACTGGTGACCCCAGCAG` |
| UP00053\_1 | chr4 | + | 39876455 | 39876471 | 2.44e-05 | 0.527 | `AGAAGTGACCCCATTCA` |
| UP00053\_1 | chr16 | + | 17226477 | 17226493 | 2.46e-05 | 0.527 | `CTGTGTGACCTCGTAAG` |
| UP00053\_1 | chr12 | − | 46493492 | 46493508 | 2.48e-05 | 0.527 | `TGAGGTGACCCCAGCCC` |
| UP00053\_1 | chr14 | + | 64693536 | 64693552 | 2.5e-05 | 0.527 | `AGACGTGAACCCGTGAG` |
| UP00053\_1 | chr11 | + | 67984109 | 67984125 | 2.52e-05 | 0.527 | `CACCTTGACCCCGCTAC` |
| UP00053\_1 | chr22 | − | 37207730 | 37207746 | 2.52e-05 | 0.527 | `GATTTTGACCCCACTAG` |
| UP00053\_1 | chr2 | + | 161268955 | 161268971 | 2.53e-05 | 0.527 | `AGCTTTGACCCCTGCCC` |
| UP00053\_1 | chr3 | + | 113554020 | 113554036 | 2.53e-05 | 0.527 | `CTTGGTGACCTCAAAAA` |
| UP00053\_1 | chr9 | + | 115384177 | 115384193 | 2.55e-05 | 0.527 | `CAGCGTGACCCCTGACA` |
| UP00053\_1 | chr21 | − | 35160034 | 35160050 | 2.57e-05 | 0.527 | `AGAAATGACCCCGAGCT` |
| UP00053\_1 | chr16 | + | 66464213 | 66464229 | 2.59e-05 | 0.527 | `TAAGTTGACCCCGTCCC` |
| UP00053\_1 | chr7 | + | 5701517 | 5701533 | 2.61e-05 | 0.527 | `TGTCCTGACCTCACACT` |
| UP00053\_1 | chr14 | + | 22071355 | 22071371 | 2.62e-05 | 0.527 | `CATGTTGACCCCATCCC` |
| UP00053\_1 | chr19 | + | 43228923 | 43228939 | 2.64e-05 | 0.527 | `GTCTTTGACCTCATGCC` |
| UP00053\_1 | chr19 | + | 3136494 | 3136510 | 2.68e-05 | 0.527 | `TCCCGTGACCCTTTGTG` |
| UP00053\_1 | chr11 | + | 8666609 | 8666625 | 2.7e-05 | 0.527 | `TTTCTTGAACCCGCATT` |
| UP00053\_1 | chr15 | + | 86983914 | 86983930 | 2.72e-05 | 0.527 | `ACCTGTGACCCCACTTG` |
| UP00053\_1 | chr12 | + | 81276496 | 81276512 | 2.77e-05 | 0.533 | `CCCGGTGACCCCGGACC` |
| UP00053\_1 | chr13 | + | 109922261 | 109922277 | 2.81e-05 | 0.536 | `GGTGGTGACCTCAGAAG` |
| UP00053\_1 | chr16 | + | 11671905 | 11671921 | 2.91e-05 | 0.537 | `CCTGGTGACCTCATGTC` |
| UP00053\_1 | chr10 | − | 35791606 | 35791622 | 2.93e-05 | 0.537 | `TGGAATGACCCCTCCAC` |
| UP00053\_1 | chr11 | + | 59995623 | 59995639 | 2.95e-05 | 0.537 | `TCTATTGACCCCAGCCT` |
| UP00053\_1 | chr12 | + | 92048732 | 92048748 | 2.95e-05 | 0.537 | `TATCATGACCCCATTGT` |
| UP00053\_1 | chr11 | + | 65076714 | 65076730 | 2.97e-05 | 0.537 | `ATCCCTGACCCCATGTG` |
| UP00053\_1 | chr20 | + | 13176018 | 13176034 | 2.97e-05 | 0.537 | `CTTCCTGACCCCTGATT` |
| UP00053\_1 | chr7 | − | 24924318 | 24924334 | 3.03e-05 | 0.537 | `TCTCTTGACCTCTCAGG` |
| UP00053\_1 | chr10 | + | 112593009 | 112593025 | 3.03e-05 | 0.537 | `ACTTGTGACCTCTCCTT` |
| UP00053\_1 | chr12 | − | 92048744 | 92048760 | 3.05e-05 | 0.537 | `GACCGTGAACCCACAAT` |
| UP00053\_1 | chr6 | − | 36828883 | 36828899 | 3.07e-05 | 0.537 | `TCCCCTGACCCCAGGCT` |
| UP00053\_1 | chr10 | − | 45236748 | 45236764 | 3.19e-05 | 0.551 | `CTGTTTGACCTCAAAAC` |
| UP00053\_1 | chr3 | − | 109327536 | 109327552 | 3.25e-05 | 0.551 | `TCCTGTGACCCCAGTCC` |
| UP00053\_1 | chr16 | + | 51685878 | 51685894 | 3.4e-05 | 0.551 | `CCCGGTGACCCCAGGTG` |
| UP00053\_1 | chr2 | + | 98443949 | 98443965 | 3.47e-05 | 0.551 | `TCCCGTGACCTCATGGG` |
| UP00053\_1 | chr11 | + | 65076569 | 65076585 | 3.51e-05 | 0.551 | `GTCTCTGACCCCATATG` |
| UP00053\_1 | chr12 | + | 92048446 | 92048462 | 3.58e-05 | 0.551 | `TTCCCTGACCCCTTTCC` |
| UP00053\_1 | chr17 | + | 38024483 | 38024499 | 3.58e-05 | 0.551 | `TGATGTGACCCCAGGGA` |
| UP00053\_1 | chr9 | − | 67903541 | 67903557 | 3.6e-05 | 0.551 | `GCTCGTGACCCCGGAGG` |
| UP00053\_1 | chr22 | − | 49314980 | 49314996 | 3.6e-05 | 0.551 | `CTTGATGACCCCGGGAA` |
| UP00053\_1 | chr17 | − | 53282790 | 53282806 | 3.63e-05 | 0.551 | `GCTGGTGACCTCAAATC` |
| UP00053\_1 | chr1 | + | 171513168 | 171513184 | 3.65e-05 | 0.551 | `CCTATTGACCCCAATTT` |
| UP00053\_1 | chr9 | + | 33294607 | 33294623 | 3.74e-05 | 0.551 | `AGATCTGACCCCAAACA` |
| UP00053\_1 | chr7 | − | 104440779 | 104440795 | 3.79e-05 | 0.551 | `GGCGATGACCTCATCCT` |
| UP00053\_1 | chr9 | − | 17853358 | 17853374 | 3.79e-05 | 0.551 | `AAGCATGACCTCAAGAT` |
| UP00053\_1 | chr22 | − | 20880045 | 20880061 | 3.81e-05 | 0.551 | `TCCTTTGACCTCAGACA` |
| UP00053\_1 | chr16 | − | 30274470 | 30274486 | 3.86e-05 | 0.551 | `TGACTTGAACCCAGGAC` |
| UP00053\_1 | chr17 | − | 44650643 | 44650659 | 3.91e-05 | 0.551 | `TGCAGTGACCTCAAACC` |
| UP00053\_1 | chr1 | − | 42045102 | 42045118 | 3.93e-05 | 0.551 | `CGTCTTGACCTCTCTTG` |
| UP00053\_1 | chr19 | − | 33312893 | 33312909 | 3.98e-05 | 0.551 | `CTTTATGACCCCTCTTG` |
| UP00053\_1 | chr7 | + | 24924399 | 24924415 | 4.03e-05 | 0.551 | `CTACTTGACCCTATCCT` |
| UP00053\_1 | chr22 | − | 36010174 | 36010190 | 4.05e-05 | 0.551 | `CCCATTGACCCCATTTA` |
| UP00053\_1 | chr1 | + | 171431752 | 171431768 | 4.18e-05 | 0.551 | `AAGCATGACCTCACAAG` |
| UP00053\_1 | chr9 | + | 85770573 | 85770589 | 4.18e-05 | 0.551 | `CATAATGACCCCATCAC` |
| UP00053\_1 | chr17 | − | 34235097 | 34235113 | 4.18e-05 | 0.551 | `GGAAGTGACCTCTAGAG` |
| UP00053\_1 | chr19 | − | 54162824 | 54162840 | 4.2e-05 | 0.551 | `TGTGATGAACCCTAAAC` |
| UP00053\_1 | chr12 | − | 123961348 | 123961364 | 4.23e-05 | 0.551 | `ACAATTGACCTCTGGAT` |
| UP00053\_1 | chr1 | − | 205170691 | 205170707 | 4.25e-05 | 0.551 | `GTGGTTGACCCCAGCCT` |
| UP00053\_1 | chr5 | − | 141761040 | 141761056 | 4.33e-05 | 0.551 | `ATCCATGACCCCTGAGA` |
| UP00053\_1 | chr6 | + | 42859501 | 42859517 | 4.33e-05 | 0.551 | `CTCCCTGACCCCGCACC` |
| UP00053\_1 | chr5 | − | 138749012 | 138749028 | 4.38e-05 | 0.551 | `GCCTTTGACCCCAGCTG` |
| UP00053\_1 | chr8 | − | 125688762 | 125688778 | 4.38e-05 | 0.551 | `TTAAATGACCCCAGATG` |
| UP00053\_1 | chr19 | − | 50965749 | 50965765 | 4.44e-05 | 0.551 | `GGTGTTGACCTCGCCCT` |
| UP00053\_1 | chr9 | − | 24922972 | 24922988 | 4.46e-05 | 0.551 | `AAATGTGACCTCAAGAA` |
| UP00053\_1 | chr11 | − | 72582711 | 72582727 | 4.46e-05 | 0.551 | `GTCCCTGACCCCTGGAA` |
| UP00053\_1 | chr7 | + | 148952910 | 148952926 | 4.49e-05 | 0.551 | `GGAGCTGACCCCGCGAC` |
| UP00053\_1 | chr11 | + | 64375652 | 64375668 | 4.49e-05 | 0.551 | `TTGGGTGACCCCTGCCC` |
| UP00053\_1 | chr5 | + | 5414351 | 5414367 | 4.52e-05 | 0.551 | `CCTGTTGACCTCTGGTT` |
| UP00053\_1 | chr14 | − | 64840502 | 64840518 | 4.52e-05 | 0.551 | `CCACCTGACCCCAGGTT` |
| UP00053\_1 | chr18 | + | 7943239 | 7943255 | 4.52e-05 | 0.551 | `ACTCATGACCCTTTGTG` |
| UP00053\_1 | chr14 | + | 105536917 | 105536933 | 4.57e-05 | 0.551 | `CTGGGTGACCCCTGGTG` |
| UP00053\_1 | chr15 | + | 19962664 | 19962680 | 4.57e-05 | 0.551 | `CTGGGTGACCCCTGGTG` |
| UP00053\_1 | chr12 | + | 122025133 | 122025149 | 4.6e-05 | 0.551 | `ACACCTGACCCCGCCCT` |
| UP00053\_1 | chr2 | − | 25498042 | 25498058 | 4.62e-05 | 0.551 | `TCTTTTGACCTCGGATA` |
| UP00053\_1 | chr20 | − | 23263204 | 23263220 | 4.62e-05 | 0.551 | `TTCCCTGACCTCAGAAT` |
| UP00053\_1 | chr7 | + | 7974291 | 7974307 | 4.71e-05 | 0.551 | `CTTGTTGACCCCTGCTA` |
| UP00053\_1 | chr4 | + | 15365028 | 15365044 | 4.73e-05 | 0.551 | `TAGTGTGACCCCATTGT` |
| UP00053\_1 | chrX | − | 46382464 | 46382480 | 4.73e-05 | 0.551 | `CCCTGTGACCTCGATAT` |
| UP00053\_1 | chr19 | − | 6724219 | 6724235 | 4.76e-05 | 0.551 | `GCCCGTGAACCCACACA` |
| UP00053\_1 | chr1 | + | 21489737 | 21489753 | 4.79e-05 | 0.551 | `CACAGTGACCCCTCCTT` |
| UP00053\_1 | chr9 | + | 129883326 | 129883342 | 4.79e-05 | 0.551 | `CCCCTTGACCTCTCTCC` |
| UP00053\_1 | chr10 | + | 125969175 | 125969191 | 4.82e-05 | 0.551 | `CCAGATGACCCCAACCG` |
| UP00053\_1 | chr7 | − | 44757097 | 44757113 | 4.84e-05 | 0.551 | `ATCCTTGACCCTGTGCC` |
| UP00053\_1 | chr16 | + | 55663375 | 55663391 | 4.84e-05 | 0.551 | `TTACATGACCTTTTACT` |
| UP00053\_1 | chr11 | − | 605839 | 605855 | 4.87e-05 | 0.551 | `CTCCGTGACCCCGGAGG` |
| UP00053\_1 | chr9 | − | 130685055 | 130685071 | 4.9e-05 | 0.551 | `CTCCATGACCCCAGCTA` |
| UP00053\_1 | chr13 | + | 21587388 | 21587404 | 4.93e-05 | 0.551 | `ATGTTTGACCTCTCCCT` |
| UP00053\_1 | chr15 | − | 29295968 | 29295984 | 4.93e-05 | 0.551 | `CGGCCTGACCCCTACAC` |
| UP00053\_1 | chr10 | − | 26816754 | 26816770 | 4.99e-05 | 0.551 | `TCTCCTGAACCCTTAAA` |
| UP00053\_1 | chr6 | − | 20985711 | 20985727 | 5.01e-05 | 0.551 | `GGCAGTGACCTCAGACT` |
| UP00053\_1 | chr12 | − | 109583764 | 109583780 | 5.01e-05 | 0.551 | `CACCGTGACCTCATGGT` |
| UP00053\_1 | chr1 | − | 201597822 | 201597838 | 5.07e-05 | 0.551 | `CCAGGTGACCCCTTTGA` |
| UP00053\_1 | chr6 | − | 32734231 | 32734247 | 5.07e-05 | 0.551 | `GGCCATGACCCTGAAAG` |
| UP00053\_1 | chr1 | + | 148398539 | 148398555 | 5.1e-05 | 0.551 | `GGAAGTGACCCCGCAGC` |
| UP00053\_1 | chr1 | + | 158877401 | 158877417 | 5.1e-05 | 0.551 | `TAATGTGACCCCTTTGG` |
| UP00053\_1 | chr2 | + | 20270553 | 20270569 | 5.1e-05 | 0.551 | `GGTTTTGACCCTAGAAG` |
| UP00053\_1 | chr10 | − | 115732901 | 115732917 | 5.1e-05 | 0.551 | `TGATCTGACCCCTCCCA` |
| UP00053\_1 | chr16 | − | 79267969 | 79267985 | 5.19e-05 | 0.551 | `GAACTTGACCCCAGTTC` |
| UP00053\_1 | chr18 | + | 59136406 | 59136422 | 5.22e-05 | 0.551 | `ACACATGACCCCACCGA` |
| UP00053\_1 | chr9 | − | 129248941 | 129248957 | 5.25e-05 | 0.551 | `TGCGATGAACCCTAAAG` |
| UP00053\_1 | chr2 | + | 122798135 | 122798151 | 5.28e-05 | 0.551 | `CTCGATGACCTCTTCAG` |
| UP00053\_1 | chr11 | + | 118068827 | 118068843 | 5.28e-05 | 0.551 | `CCAAGTGACCCCAACTC` |
| UP00053\_1 | chr1 | − | 45047795 | 45047811 | 5.31e-05 | 0.551 | `GACTGTGACCCCAAAGA` |
| UP00053\_1 | chr2 | − | 191535313 | 191535329 | 5.34e-05 | 0.551 | `AACCATGACCTCATTAG` |
| UP00053\_1 | chr15 | − | 62236914 | 62236930 | 5.37e-05 | 0.551 | `GAAAATGACCCCATTTT` |
| UP00053\_1 | chr6 | + | 26468904 | 26468920 | 5.43e-05 | 0.551 | `CTGGGTGACCCCTATTC` |
| UP00053\_1 | chr7 | + | 4689675 | 4689691 | 5.46e-05 | 0.551 | `CACCTTGACCCCAGTCC` |
| UP00053\_1 | chr2 | − | 111642873 | 111642889 | 5.52e-05 | 0.551 | `TTGGGTGACCCCTTTGA` |
| UP00053\_1 | chr12 | − | 103617451 | 103617467 | 5.52e-05 | 0.551 | `CTCAGTGACCCTACAAT` |
| UP00053\_1 | chr4 | − | 40909459 | 40909475 | 5.55e-05 | 0.551 | `CTGTATGACCCCGAAGT` |
| UP00053\_1 | chr9 | + | 116483858 | 116483874 | 5.55e-05 | 0.551 | `TCTAGTGACCCCATTGC` |
| UP00053\_1 | chr16 | − | 18720508 | 18720524 | 5.55e-05 | 0.551 | `TCCTTTGACCTCGATTT` |
| UP00053\_1 | chr5 | + | 90712893 | 90712909 | 5.61e-05 | 0.551 | `AGTCTTAACCCCTTGAG` |
| UP00053\_1 | chr3 | − | 10237487 | 10237503 | 5.67e-05 | 0.551 | `AGTGGTGACCTCTCTCA` |
| UP00053\_1 | chr6 | + | 36762874 | 36762890 | 5.67e-05 | 0.551 | `GGGTCTGACCCCAAACA` |
| UP00053\_1 | chr8 | + | 96313004 | 96313020 | 5.67e-05 | 0.551 | `GGCCATGACCCTAGAAA` |
| UP00053\_1 | chr15 | − | 56533999 | 56534015 | 5.67e-05 | 0.551 | `CTTCCTGACCCCATTTG` |
| UP00053\_1 | chr15 | + | 57623414 | 57623430 | 5.67e-05 | 0.551 | `AAAGGTGACCCCAAAGA` |
| UP00053\_1 | chr19 | − | 59404070 | 59404086 | 5.7e-05 | 0.551 | `TGCCCTGACCTCAAACC` |
| UP00053\_1 | chr1 | − | 12039504 | 12039520 | 5.73e-05 | 0.551 | `GGCTGTGAACCCTCATG` |
| UP00053\_1 | chr3 | − | 51403596 | 51403612 | 5.73e-05 | 0.551 | `CCAGGTGACCCTGTGAG` |
| UP00053\_1 | chr3 | + | 42029448 | 42029464 | 5.8e-05 | 0.551 | `CTCTATGACCCCTGGGT` |
| UP00053\_1 | chr11 | + | 61292764 | 61292780 | 5.8e-05 | 0.551 | `CGTGTTGACCTCTTGGG` |
| UP00053\_1 | chr6 | − | 30189202 | 30189218 | 5.83e-05 | 0.551 | `TTGGTTGACCCTGTGAC` |
| UP00053\_1 | chrX | + | 41095181 | 41095197 | 5.86e-05 | 0.551 | `AGTCATGAACTCAAAAT` |
| UP00053\_1 | chr11 | − | 74489218 | 74489234 | 5.86e-05 | 0.551 | `TGAAATGAACCCTTCAT` |
| UP00053\_1 | chr2 | + | 231558458 | 231558474 | 5.89e-05 | 0.551 | `TCAGGCGACCCCTTAAG` |
| UP00053\_1 | chr11 | + | 63764394 | 63764410 | 5.89e-05 | 0.551 | `GGATTTGACCTCAACTC` |
| UP00053\_1 | chr19 | − | 63611494 | 63611510 | 5.89e-05 | 0.551 | `CCCGGTGACCTCACAGT` |
| UP00053\_1 | chr3 | − | 72308415 | 72308431 | 5.92e-05 | 0.551 | `GGATCTGACCCCAGACA` |
| UP00053\_1 | chr19 | + | 6724404 | 6724420 | 5.92e-05 | 0.551 | `TCCTTTGACCCCCAGAG` |
| UP00053\_1 | chr1 | − | 158865167 | 158865183 | 5.96e-05 | 0.551 | `GCAGGTGACCCTGCATT` |
| UP00053\_1 | chr6 | + | 7844353 | 7844369 | 5.96e-05 | 0.551 | `AGCCCTGACCCCAGGTG` |
| UP00053\_1 | chr19 | − | 10565758 | 10565774 | 5.99e-05 | 0.551 | `TTCTCTGACCCCAAGTA` |
| UP00053\_1 | chr20 | − | 22170581 | 22170597 | 5.99e-05 | 0.551 | `TGAATTGACCCTTTCTT` |
| UP00053\_1 | chr1 | + | 111937040 | 111937056 | 6.02e-05 | 0.551 | `AGTAATGAACCCAAAAT` |
| UP00053\_1 | chr6 | + | 3313039 | 3313055 | 6.02e-05 | 0.551 | `CCCAGTGACCCCAACTG` |
| UP00053\_1 | chr1 | − | 204920090 | 204920106 | 6.05e-05 | 0.551 | `GAAAATGACCTCAAAAT` |
| UP00053\_1 | chr2 | + | 74051830 | 74051846 | 6.05e-05 | 0.551 | `GAACTTGACCTCAGATG` |
| UP00053\_1 | chr2 | + | 179103631 | 179103647 | 6.05e-05 | 0.551 | `CCAGGTGACCTCTTTTG` |
| UP00053\_1 | chrX | − | 70760617 | 70760633 | 6.05e-05 | 0.551 | `CTGTTTGACCTCACCCT` |
| UP00053\_1 | chr6 | − | 86439943 | 86439959 | 6.12e-05 | 0.551 | `TGTATTGACCTTGTAAT` |
| UP00053\_1 | chr18 | − | 3583979 | 3583995 | 6.12e-05 | 0.551 | `CTCTCTGACCCCTCCCT` |
| UP00053\_1 | chr3 | + | 99765232 | 99765248 | 6.15e-05 | 0.551 | `GTATATGACCCTACAAG` |
| UP00053\_1 | chr11 | + | 103274758 | 103274774 | 6.19e-05 | 0.551 | `TGACTTGAACTCTTGCA` |
| UP00053\_1 | chr21 | + | 42157748 | 42157764 | 6.19e-05 | 0.551 | `CGCTGTGACCTCTCTCG` |
| UP00053\_1 | chr2 | − | 152740677 | 152740693 | 6.22e-05 | 0.551 | `CCGCCTGACCCCTACAA` |
| UP00053\_1 | chr3 | + | 48489728 | 48489744 | 6.22e-05 | 0.551 | `GGGCCTGACCTCAGAAT` |
| UP00053\_1 | chr16 | − | 88159131 | 88159147 | 6.22e-05 | 0.551 | `AAAAATGACCTCTTGCT` |
| UP00053\_1 | chr19 | + | 17837572 | 17837588 | 6.29e-05 | 0.551 | `AGGCTTGACCCTTCTAC` |
| UP00053\_1 | chr1 | + | 220016953 | 220016969 | 6.32e-05 | 0.551 | `CACATTGACCTCACACT` |
| UP00053\_1 | chr15 | − | 68094287 | 68094303 | 6.36e-05 | 0.551 | `AAATGTGAACCCACAAC` |
| UP00053\_1 | chr1 | + | 27825406 | 27825422 | 6.39e-05 | 0.551 | `CCTTGTGAACCCTGATT` |
| UP00053\_1 | chr1 | − | 201557048 | 201557064 | 6.39e-05 | 0.551 | `TGAAATGACCTCTTCCA` |
| UP00053\_1 | chr21 | − | 44451305 | 44451321 | 6.39e-05 | 0.551 | `AAAAGTGACCCCTGCAC` |
| UP00053\_1 | chr8 | + | 126519991 | 126520007 | 6.46e-05 | 0.551 | `AGACTTGAACCCATTTG` |
| UP00053\_1 | chr17 | − | 1920854 | 1920870 | 6.46e-05 | 0.551 | `GTCAGTGACCCCGCCTC` |
| UP00053\_1 | chr1 | + | 148398466 | 148398482 | 6.5e-05 | 0.551 | `ATGCCTGACCCCACTAC` |
| UP00053\_1 | chr20 | − | 23262848 | 23262864 | 6.5e-05 | 0.551 | `CTCCCTGACCTCAGAAT` |
| UP00053\_1 | chr20 | − | 23262868 | 23262884 | 6.5e-05 | 0.551 | `CTCCCTGACCTCAGAAT` |
| UP00053\_1 | chr11 | + | 33662627 | 33662643 | 6.56e-05 | 0.551 | `CCTCATGACCTCTGTAC` |
| UP00053\_1 | chr11 | + | 48087991 | 48088007 | 6.56e-05 | 0.551 | `GTAATTGACCCCGAGGT` |
| UP00053\_1 | chr19 | − | 9799661 | 9799677 | 6.56e-05 | 0.551 | `CCGCTTGACCCCCTCAC` |
| UP00053\_1 | chr12 | + | 107487063 | 107487079 | 6.6e-05 | 0.552 | `GTGTATGACCTCAGAAC` |
| UP00053\_1 | chr11 | − | 72765796 | 72765812 | 6.63e-05 | 0.553 | `CCCAGTGACCCCGGGTC` |
| UP00053\_1 | chr11 | + | 75190334 | 75190350 | 6.7e-05 | 0.554 | `CCACATGACCCTGCCAT` |
| UP00053\_1 | chr1 | − | 67923342 | 67923358 | 6.74e-05 | 0.554 | `CAATTTGACCCCAGCTC` |
| UP00053\_1 | chr4 | + | 7824313 | 7824329 | 6.74e-05 | 0.554 | `CTGTGTGACCCCAGAGC` |
| UP00053\_1 | chr9 | + | 37393207 | 37393223 | 6.78e-05 | 0.554 | `CCACATGACCTCATGGG` |
| UP00053\_1 | chr3 | + | 13104824 | 13104840 | 6.85e-05 | 0.554 | `TCGCTTGACCTCTGCTC` |
| UP00053\_1 | chr2 | − | 238014466 | 238014482 | 6.89e-05 | 0.554 | `TGCTGTGACCCCAGTGC` |
| UP00053\_1 | chr17 | − | 59274278 | 59274294 | 6.89e-05 | 0.554 | `ACCTCTGACCCCGCGCC` |
| UP00053\_1 | chr6 | + | 31636278 | 31636294 | 6.92e-05 | 0.554 | `TCCTCTGACCCCTTGGG` |
| UP00053\_1 | chr3 | − | 123304339 | 123304355 | 6.99e-05 | 0.554 | `TTCAGTGACCTCTCTAA` |
| UP00053\_1 | chr20 | + | 61730049 | 61730065 | 6.99e-05 | 0.554 | `TCTCCTGACCTCTCCAG` |
| UP00053\_1 | chr22 | + | 41341104 | 41341120 | 7.03e-05 | 0.554 | `GATCATGACCTCAACAG` |
| UP00053\_1 | chr3 | − | 123460475 | 123460491 | 7.07e-05 | 0.554 | `GTCCATGACCTCACCCG` |
| UP00053\_1 | chr4 | + | 145688380 | 145688396 | 7.07e-05 | 0.554 | `TGTGATGACCCCAATGC` |
| UP00053\_1 | chr10 | − | 112118232 | 112118248 | 7.11e-05 | 0.554 | `TATCTTGACCTTTCAAC` |
| UP00053\_1 | chr17 | − | 38087480 | 38087496 | 7.14e-05 | 0.554 | `GAAAGTGACCCCGTAGA` |
| UP00053\_1 | chr11 | − | 65075999 | 65076015 | 7.18e-05 | 0.554 | `TAGCCTGACCCCTTCTG` |
| UP00053\_1 | chr17 | + | 2561831 | 2561847 | 7.18e-05 | 0.554 | `GCCTTTGACCCCGCCGC` |
| UP00053\_1 | chr20 | + | 51702454 | 51702470 | 7.18e-05 | 0.554 | `TCATATGACCCCACTGG` |
| UP00053\_1 | chr1 | − | 181259719 | 181259735 | 7.22e-05 | 0.554 | `CTTGGTGACCCCGGTCA` |
| UP00053\_1 | chr1 | − | 2480900 | 2480916 | 7.26e-05 | 0.554 | `CTCTATGACCCTGTGCT` |
| UP00053\_1 | chr11 | − | 9438574 | 9438590 | 7.26e-05 | 0.554 | `AGACCTGACCTCTCCCT` |
| UP00053\_1 | chr5 | − | 149764165 | 149764181 | 7.29e-05 | 0.554 | `ACAGGTGACCTCTCTCA` |
| UP00053\_1 | chr15 | − | 29296589 | 29296605 | 7.29e-05 | 0.554 | `TATCCTGACCCCGTTTC` |
| UP00053\_1 | chr2 | + | 43483574 | 43483590 | 7.33e-05 | 0.554 | `CTGTGTGAACCCAAACT` |
| UP00053\_1 | chr10 | − | 26816845 | 26816861 | 7.33e-05 | 0.554 | `TTTGCTGACCTCAAAAC` |
| UP00053\_1 | chr2 | − | 231233668 | 231233684 | 7.37e-05 | 0.554 | `GTGTTTGACCCCGGCCA` |
| UP00053\_1 | chr1 | + | 87344546 | 87344562 | 7.41e-05 | 0.554 | `GCCTGTGAACTCTTAAA` |
| UP00053\_1 | chr6 | − | 135685707 | 135685723 | 7.41e-05 | 0.554 | `TCATTTGACCCTGCCAC` |
| UP00053\_1 | chr9 | − | 3516817 | 3516833 | 7.52e-05 | 0.554 | `AGTGATGACCTCGCTTT` |
| UP00053\_1 | chr11 | + | 117786184 | 117786200 | 7.56e-05 | 0.554 | `TGAAATGACCTCTTTTC` |
| UP00053\_1 | chr14 | + | 67747672 | 67747688 | 7.56e-05 | 0.554 | `CTTTGTGACCCTTTCCC` |
| UP00053\_1 | chr7 | + | 2901140 | 2901156 | 7.6e-05 | 0.554 | `CTTGTTGACCCCAGGGG` |
| UP00053\_1 | chr14 | + | 61198237 | 61198253 | 7.6e-05 | 0.554 | `TTTCTTGAACCCTCTCA` |
| UP00053\_1 | chr17 | + | 73652170 | 73652186 | 7.6e-05 | 0.554 | `CCCAGTGACCTCTGGCT` |
| UP00053\_1 | chr5 | + | 180595345 | 180595361 | 7.68e-05 | 0.554 | `TGAGGTGAACTCATGTT` |
| UP00053\_1 | chr9 | − | 116483894 | 116483910 | 7.68e-05 | 0.554 | `CTGTTTGACCTCATTCA` |
| UP00053\_1 | chr10 | − | 1085086 | 1085102 | 7.68e-05 | 0.554 | `CGTGTTGACCCCCTTAA` |
| UP00053\_1 | chr2 | + | 235074099 | 235074115 | 7.72e-05 | 0.554 | `ATAAGTGACCCCCTGCT` |
| UP00053\_1 | chr17 | − | 16225273 | 16225289 | 7.72e-05 | 0.554 | `CACCTTGACCTCACCCA` |
| UP00053\_1 | chr17 | + | 63077716 | 63077732 | 7.72e-05 | 0.554 | `GCAGTTGACCTTTTAAG` |
| UP00053\_1 | chr19 | + | 55536281 | 55536297 | 7.72e-05 | 0.554 | `CCATTTGACCTCTAAGA` |
| UP00053\_1 | chr22 | − | 27526727 | 27526743 | 7.8e-05 | 0.555 | `GAAAATGACCCCAAGTA` |
| UP00053\_1 | chr9 | − | 113814376 | 113814392 | 7.84e-05 | 0.555 | `CAGCCTGACCTCTTAAG` |
| UP00053\_1 | chr11 | − | 72765805 | 72765821 | 7.88e-05 | 0.555 | `CTTCGTGACCCCAGTGA` |
| UP00053\_1 | chr15 | − | 29440839 | 29440855 | 7.88e-05 | 0.555 | `CCAAGTGACCTCACCAA` |
| UP00053\_1 | chr7 | + | 35736543 | 35736559 | 7.92e-05 | 0.555 | `ATGGGTGACCCTGCACT` |
| UP00053\_1 | chr16 | − | 2141335 | 2141351 | 7.92e-05 | 0.555 | `TTATTTGAACTCATGAC` |
| UP00053\_1 | chr17 | − | 63745891 | 63745907 | 7.92e-05 | 0.555 | `TCAGTTGAACCCTGGAG` |
| UP00053\_1 | chr9 | + | 84959274 | 84959290 | 7.96e-05 | 0.555 | `TGCCCTGACCCTGTGTT` |
| UP00053\_1 | chr11 | + | 65076579 | 65076595 | 7.96e-05 | 0.555 | `CCATATGACCCTGAACT` |
| UP00053\_1 | chr5 | − | 139908173 | 139908189 | 8e-05 | 0.555 | `AATCCTGACCCCTCCCA` |
| UP00053\_1 | chrX | − | 153282888 | 153282904 | 8.04e-05 | 0.555 | `GCAGGTGACCTCAGCAG` |
| UP00053\_1 | chr17 | − | 38114321 | 38114337 | 8.08e-05 | 0.555 | `CTTGTTGACCCTGTGCC` |
| UP00053\_1 | chr20 | − | 23263224 | 23263240 | 8.08e-05 | 0.555 | `CTTTCTGACCTCAGAAT` |
| UP00053\_1 | chr12 | − | 14325135 | 14325151 | 8.12e-05 | 0.555 | `ACTCATGAACCCAGGAA` |
| UP00053\_1 | chr19 | + | 7672790 | 7672806 | 8.12e-05 | 0.555 | `GGTTGTGACCTCCTGCT` |
| UP00053\_1 | chr8 | + | 125003635 | 125003651 | 8.16e-05 | 0.555 | `TCACATGACCTCAGCTG` |
| UP00053\_1 | chr10 | + | 63326676 | 63326692 | 8.24e-05 | 0.555 | `TCACATGACCCTTCCCA` |
| UP00053\_1 | chr10 | − | 121413759 | 121413775 | 8.28e-05 | 0.555 | `AAAAATGACCCCAGATG` |
| UP00053\_1 | chr18 | − | 9663456 | 9663472 | 8.28e-05 | 0.555 | `ATGCCTGACCCCTGCAC` |
| UP00053\_1 | chr19 | − | 52153992 | 52154008 | 8.28e-05 | 0.555 | `ATCTGTGACCTCAATCC` |
| UP00053\_1 | chr8 | + | 102218650 | 102218666 | 8.32e-05 | 0.555 | `GAGTATGACCCCAGCAG` |
| UP00053\_1 | chr11 | + | 74489157 | 74489173 | 8.32e-05 | 0.555 | `TGAGATGACCCTGTTAC` |
| UP00053\_1 | chr15 | − | 73184394 | 73184410 | 8.32e-05 | 0.555 | `GGGCATGACCTCAGAGT` |
| UP00053\_1 | chr20 | − | 23262967 | 23262983 | 8.41e-05 | 0.557 | `CTGCCTGACCTCAGAAT` |
| UP00053\_1 | chr22 | + | 46771213 | 46771229 | 8.41e-05 | 0.557 | `GGTTTTGACCTCAGCTG` |
| UP00053\_1 | chr5 | + | 86448793 | 86448809 | 8.75e-05 | 0.561 | `AACAGTGACCCCACTTA` |
| UP00053\_1 | chr7 | + | 101286910 | 101286926 | 8.75e-05 | 0.561 | `GGGTGTGAACCCTCCCT` |
| UP00053\_1 | chr8 | − | 102218454 | 102218470 | 8.79e-05 | 0.561 | `CGCACTGACCCCTCCAC` |
| UP00053\_1 | chr14 | − | 58698463 | 58698479 | 8.79e-05 | 0.561 | `GGGGCTGACCCCATCTA` |
| UP00053\_1 | chr16 | + | 11679800 | 11679816 | 8.79e-05 | 0.561 | `GAGCCTGACCCCTGGAA` |
| UP00053\_1 | chr6 | + | 106670130 | 106670146 | 8.84e-05 | 0.561 | `ACATTTGACCCCGATGA` |
| UP00053\_1 | chr14 | + | 94850881 | 94850897 | 8.84e-05 | 0.561 | `TGGCCTGACCCCAGCCA` |
| UP00053\_1 | chr17 | + | 73634991 | 73635007 | 8.84e-05 | 0.561 | `CCCTGTGACCTCAAAGA` |
| UP00053\_1 | chr1 | + | 114156839 | 114156855 | 8.88e-05 | 0.561 | `TCCTGTGACCCTTCCTC` |
| UP00053\_1 | chr15 | + | 43279829 | 43279845 | 8.88e-05 | 0.561 | `TGTTGTGAACTCAGGAT` |
| UP00053\_1 | chr3 | − | 53780089 | 53780105 | 8.92e-05 | 0.561 | `CTCTGTGACCCTTCCAG` |
| UP00053\_1 | chr19 | + | 12871776 | 12871792 | 8.97e-05 | 0.561 | `ACATCTGACCCCAAGGT` |
| UP00053\_1 | chr3 | − | 6277315 | 6277331 | 9.01e-05 | 0.561 | `CGAGATGACCTCTGCTT` |
| UP00053\_1 | chr3 | − | 113662389 | 113662405 | 9.06e-05 | 0.561 | `CTTTATGAACCCATCTT` |
| UP00053\_1 | chr1 | − | 27824233 | 27824249 | 9.1e-05 | 0.561 | `GTCTCTGACCCCTCCCA` |
| UP00053\_1 | chr1 | + | 207972892 | 207972908 | 9.1e-05 | 0.561 | `CTCCCTGACCTCATGTG` |
| UP00053\_1 | chr1 | + | 108051388 | 108051404 | 9.15e-05 | 0.561 | `CTCTATGACCCTGAAAC` |
| UP00053\_1 | chr9 | − | 131689326 | 131689342 | 9.15e-05 | 0.561 | `TGTATTGACCTTGTAAC` |
| UP00053\_1 | chr10 | − | 96986307 | 96986323 | 9.15e-05 | 0.561 | `GTTCATGACCTTTCATT` |
| UP00053\_1 | chr20 | − | 23262828 | 23262844 | 9.15e-05 | 0.561 | `CTCTCTGACCTCAGAAT` |
| UP00053\_1 | chr20 | − | 23263184 | 23263200 | 9.15e-05 | 0.561 | `CTCTCTGACCTCAGAAT` |
| UP00053\_1 | chr20 | − | 23263264 | 23263280 | 9.15e-05 | 0.561 | `CTCTCTGACCTCAGAAT` |
| UP00053\_1 | chr12 | − | 70344055 | 70344071 | 9.19e-05 | 0.561 | `GGGTGTGACCCTAGGAC` |
| UP00053\_1 | chr6 | + | 33237129 | 33237145 | 9.24e-05 | 0.561 | `TAATGTGACCCTGAGAG` |
| UP00053\_1 | chr19 | − | 3136594 | 3136610 | 9.28e-05 | 0.561 | `GGACGTGACCTTGCGAC` |
| UP00053\_1 | chr22 | − | 46770822 | 46770838 | 9.28e-05 | 0.561 | `ATTCATGACCCTGTCCC` |
| UP00053\_1 | chr1 | − | 210172908 | 210172924 | 9.33e-05 | 0.561 | `CTCTATGACCTCTTGGC` |
| UP00053\_1 | chr6 | − | 27208650 | 27208666 | 9.33e-05 | 0.561 | `AGACCTGACCTCTGACG` |
| UP00053\_1 | chr14 | + | 49118932 | 49118948 | 9.33e-05 | 0.561 | `TTCTCTGACCTCAACAT` |
| UP00053\_1 | chr7 | − | 2984566 | 2984582 | 9.37e-05 | 0.561 | `TTCCTTGACCCTGCCTC` |
| UP00053\_1 | chr7 | − | 104411490 | 104411506 | 9.37e-05 | 0.561 | `TCCGTTGAACCCTTTTC` |
| UP00053\_1 | chr8 | + | 101998430 | 101998446 | 9.42e-05 | 0.561 | `TTTCTTGACCTTTCCCT` |
| UP00053\_1 | chr13 | − | 97939031 | 97939047 | 9.42e-05 | 0.561 | `TCGTGTGACCTCTGCCA` |
| UP00053\_1 | chr3 | + | 49257228 | 49257244 | 9.47e-05 | 0.561 | `CTTTCTGACCCCAGCCT` |
| UP00053\_1 | chr8 | + | 125277450 | 125277466 | 9.47e-05 | 0.561 | `TCGATTGACCTCTATAA` |
| UP00053\_1 | chrX | − | 141893745 | 141893761 | 9.51e-05 | 0.561 | `CTGCTTGACCTCTGGGT` |
| UP00053\_1 | chr18 | + | 17966500 | 17966516 | 9.51e-05 | 0.561 | `CTCTATGACCTCTCCTC` |
| UP00053\_1 | chr4 | + | 122280293 | 122280309 | 9.56e-05 | 0.561 | `CAGTTTGACCCCACTGT` |
| UP00053\_1 | chr12 | + | 29196648 | 29196664 | 9.56e-05 | 0.561 | `CTCTCTGACCTCAAAAC` |
| UP00053\_1 | chr11 | + | 36722251 | 36722267 | 9.61e-05 | 0.561 | `ATCCATGAACCCACCAG` |
| UP00053\_1 | chr8 | − | 128291753 | 128291769 | 9.7e-05 | 0.561 | `TCACATGACCCTACCTG` |
| UP00053\_1 | chr13 | + | 47614743 | 47614759 | 9.7e-05 | 0.561 | `ATAAGTGACCTCAGATA` |
| UP00053\_1 | chr9 | − | 131833445 | 131833461 | 9.75e-05 | 0.561 | `GACTATGACCTCACCTT` |
| UP00053\_1 | chr11 | + | 110735302 | 110735318 | 9.75e-05 | 0.561 | `CTGCCTGACCCCTGCAG` |
| UP00053\_1 | chr15 | − | 62962581 | 62962597 | 9.79e-05 | 0.561 | `TGAAATGACCCCTCTGA` |
| UP00053\_1 | chr4 | + | 182481469 | 182481485 | 9.84e-05 | 0.561 | `ACCATTGACCTCAATAA` |
| UP00053\_1 | chr13 | + | 76218238 | 76218254 | 9.84e-05 | 0.561 | `CCCATTGACCTCTTTTG` |
| UP00053\_1 | chr22 | − | 35971487 | 35971503 | 9.84e-05 | 0.561 | `TGTTGTGAACCCAGTAA` |
| UP00053\_1 | chr1 | − | 29957628 | 29957644 | 9.89e-05 | 0.561 | `CACATTGACCCCACTTG` |
| UP00053\_1 | chr1 | − | 101473865 | 101473881 | 9.89e-05 | 0.561 | `AGATGTGAACTCAAAAA` |
| UP00053\_1 | chr14 | + | 105119402 | 105119418 | 9.89e-05 | 0.561 | `CTAGCTGACCCCGGGCT` |
| UP00053\_1 | chr20 | + | 45820990 | 45821006 | 9.89e-05 | 0.561 | `TGCTGTGACCTTGAAAG` |
| UP00053\_1 | chr1 | + | 30978136 | 30978152 | 9.94e-05 | 0.561 | `CGCCATGACCCCAGTGG` |
| UP00053\_1 | chr7 | − | 100515544 | 100515560 | 9.94e-05 | 0.561 | `GATCTTGACCTCTAGGA` |
| UP00053\_1 | chr9 | − | 93010356 | 93010372 | 9.94e-05 | 0.561 | `TTTCTTGACCTTTCCAA` |
| UP00053\_1 | chr19 | + | 9799340 | 9799356 | 9.94e-05 | 0.561 | `GCGCCTGACCTCTGACT` |
| UP00053\_1 | chr15 | − | 72483676 | 72483692 | 9.99e-05 | 0.562 | `CTTCCTGACCCCAGCCC` |

---

**DEBUGGING INFORMATION**


---

Command line:

```
/ebi/sw/MEME/VM-cluster410/meme-versions/4.10.0/bin/fimo --parse-genomic-coord --verbosity 1 --oc fimo_out_12 --bgfile ./background --motif UP00053_1 db/uniprobe_mouse.meme ./Supplementary_Table_1.500bp.fa
```

Settings:

```
|  |  |  |
| --- | --- | --- |
| output directory = fimo_out_12 | MEME file name = db/uniprobe_mouse.meme | sequence file name = ./Supplementary_Table_1.500bp.fa |
| background file name = ./background | allow clobber = true | compute q-values = true |
| parse genomic coord. = true | text only = false | scan both strands = true |
| max sequence length = 250000000 | output threshold = 0.0001 | threshold type = p-value |
| max stored scores = 100000 | pseudocount = 0.1 | verbosity = 1 |
| selected motif = UP00053_1 |  |  |
```

This information can be useful in the event you wish to report a
problem with the FIMO software.

---

**Go to top**
