# Supplementary material for: The MEME Suite
Source: Nucleic Acids Res. 2015 May 7;43(Web Server issue):W39–49. doi: 10.1093/nar/gkv416 (PMC4489269; doi:10.1093/nar/gkv416)
Supplement: SUPPLEMENTARY DATA [file supp_gkv416_nar-00283-web-b-2015-File005.zip › case4/meme-chip/fimo_out_13/fimo.html]

FIMO Results


---

|  |  |  |
| --- | --- | --- |
| **Database and Motifs** | **High-scoring Motif Occurrences** | **Debugging Information** |

  
  

---

**FIMO - Motif search tool**


---

FIMO version 4.10.0,
(Release date: Wed May 21 10:35:36 2014 +1000)

For further information on how to interpret these results
or to get a copy of the FIMO software please access
http://meme.nbcr.net

If you use FIMO in your research, please cite the following paper:  
Charles E. Grant, Timothy L. Bailey, and William Stafford Noble,
"FIMO: Scanning for occurrences of a given motif",
*Bioinformatics*, **27**(7):1017-1018, 2011.
[full text]

---

**DATABASE AND MOTIFS**


---

DATABASE
./Supplementary\_Table\_1.500bp.fa  
Database contains
2776
sequences,
1388000
residues

MOTIFS
dreme\_out/dreme.xml
(nucleotide)

| MOTIF | WIDTH | BEST POSSIBLE MATCH |
| --- | --- | --- |
| GGAARY | 6 | GGAAGT |
| AVTGAAA | 7 | ACTGAAA |
| RCAGCTGY | 8 | GCAGCTGC |
| AKAAAH | 6 | AGAAAA |
| RAGKTCA | 7 | GAGGTCA |
| CMCAGM | 6 | CCCAGC |
| CCCCRCCC | 8 | CCCCGCCC |
| AAATR | 5 | AAATG |
| GAAASCA | 7 | GAAAGCA |
| CCGSCTCC | 8 | CCGCCTCC |
| CCWCCTGC | 8 | CCACCTGC |

Random model letter frequencies
(from ./background):
  
A 0.241 C 0.259 G 0.259 T 0.241

---

**SECTION I: HIGH-SCORING MOTIF OCCURRENCES**


---

- There were
  315
  motif occurrences with a
  p-value less than
  0.0001.
- The p-value of a motif occurrence is defined as the
  probability of a random sequence of the same length as the motif
  matching that position of the sequence with as good or better a score.
- The score for the match of a position in a sequence to a motif
  is computed by summing the appropriate entries from each column of
  the position-dependent scoring matrix that represents the motif.
- The q-value of a motif occurrence is defined as the
  false discovery rate if the occurrence is accepted as significant.
- The table is sorted by increasing p-value.

| Motif | Sequence Name | Strand | Start | End | p-value | q-value | Matched Sequence |
| --- | --- | --- | --- | --- | --- | --- | --- |
| GAAASCA | chr1 | + | 12061454 | 12061460 | 5.85e-05 | 0.405 | `GAAAGCA` |
| GAAASCA | chr1 | − | 25151639 | 25151645 | 5.85e-05 | 0.405 | `GAAAGCA` |
| GAAASCA | chr1 | − | 26892328 | 26892334 | 5.85e-05 | 0.405 | `GAAAGCA` |
| GAAASCA | chr1 | − | 28083163 | 28083169 | 5.85e-05 | 0.405 | `GAAAGCA` |
| GAAASCA | chr1 | − | 28776465 | 28776471 | 5.85e-05 | 0.405 | `GAAAGCA` |
| GAAASCA | chr1 | − | 28776589 | 28776595 | 5.85e-05 | 0.405 | `GAAAGCA` |
| GAAASCA | chr1 | + | 28847693 | 28847699 | 5.85e-05 | 0.405 | `GAAAGCA` |
| GAAASCA | chr1 | − | 31761212 | 31761218 | 5.85e-05 | 0.405 | `GAAAGCA` |
| GAAASCA | chr1 | + | 67913147 | 67913153 | 5.85e-05 | 0.405 | `GAAAGCA` |
| GAAASCA | chr1 | + | 67923684 | 67923690 | 5.85e-05 | 0.405 | `GAAAGCA` |
| GAAASCA | chr1 | + | 89129817 | 89129823 | 5.85e-05 | 0.405 | `GAAAGCA` |
| GAAASCA | chr1 | + | 95141423 | 95141429 | 5.85e-05 | 0.405 | `GAAAGCA` |
| GAAASCA | chr1 | + | 101609503 | 101609509 | 5.85e-05 | 0.405 | `GAAAGCA` |
| GAAASCA | chr1 | − | 101646094 | 101646100 | 5.85e-05 | 0.405 | `GAAAGCA` |
| GAAASCA | chr1 | − | 111560128 | 111560134 | 5.85e-05 | 0.405 | `GAAAGCA` |
| GAAASCA | chr1 | − | 111564222 | 111564228 | 5.85e-05 | 0.405 | `GAAAGCA` |
| GAAASCA | chr1 | − | 114249251 | 114249257 | 5.85e-05 | 0.405 | `GAAAGCA` |
| GAAASCA | chr1 | + | 117998603 | 117998609 | 5.85e-05 | 0.405 | `GAAAGCA` |
| GAAASCA | chr1 | + | 118008744 | 118008750 | 5.85e-05 | 0.405 | `GAAAGCA` |
| GAAASCA | chr1 | + | 152184948 | 152184954 | 5.85e-05 | 0.405 | `GAAAGCA` |
| GAAASCA | chr1 | − | 155418386 | 155418392 | 5.85e-05 | 0.405 | `GAAAGCA` |
| GAAASCA | chr1 | + | 158862808 | 158862814 | 5.85e-05 | 0.405 | `GAAAGCA` |
| GAAASCA | chr1 | − | 158882693 | 158882699 | 5.85e-05 | 0.405 | `GAAAGCA` |
| GAAASCA | chr1 | − | 158882815 | 158882821 | 5.85e-05 | 0.405 | `GAAAGCA` |
| GAAASCA | chr1 | + | 158882517 | 158882523 | 5.85e-05 | 0.405 | `GAAAGCA` |
| GAAASCA | chr1 | − | 165951526 | 165951532 | 5.85e-05 | 0.405 | `GAAAGCA` |
| GAAASCA | chr1 | + | 171431750 | 171431756 | 5.85e-05 | 0.405 | `GAAAGCA` |
| GAAASCA | chr1 | + | 171449194 | 171449200 | 5.85e-05 | 0.405 | `GAAAGCA` |
| GAAASCA | chr1 | + | 171512895 | 171512901 | 5.85e-05 | 0.405 | `GAAAGCA` |
| GAAASCA | chr1 | − | 173757581 | 173757587 | 5.85e-05 | 0.405 | `GAAAGCA` |
| GAAASCA | chr1 | − | 176817416 | 176817422 | 5.85e-05 | 0.405 | `GAAAGCA` |
| GAAASCA | chr1 | − | 204439687 | 204439693 | 5.85e-05 | 0.405 | `GAAAGCA` |
| GAAASCA | chr1 | + | 207972935 | 207972941 | 5.85e-05 | 0.405 | `GAAAGCA` |
| GAAASCA | chr1 | − | 207996237 | 207996243 | 5.85e-05 | 0.405 | `GAAAGCA` |
| GAAASCA | chr1 | − | 224917186 | 224917192 | 5.85e-05 | 0.405 | `GAAAGCA` |
| GAAASCA | chr1 | − | 228391106 | 228391112 | 5.85e-05 | 0.405 | `GAAAGCA` |
| GAAASCA | chr2 | − | 3600942 | 3600948 | 5.85e-05 | 0.405 | `GAAAGCA` |
| GAAASCA | chr2 | − | 9837920 | 9837926 | 5.85e-05 | 0.405 | `GAAAGCA` |
| GAAASCA | chr2 | − | 11888062 | 11888068 | 5.85e-05 | 0.405 | `GAAAGCA` |
| GAAASCA | chr2 | + | 25498172 | 25498178 | 5.85e-05 | 0.405 | `GAAAGCA` |
| GAAASCA | chr2 | + | 30413645 | 30413651 | 5.85e-05 | 0.405 | `GAAAGCA` |
| GAAASCA | chr2 | + | 38015137 | 38015143 | 5.85e-05 | 0.405 | `GAAAGCA` |
| GAAASCA | chr2 | + | 54649899 | 54649905 | 5.85e-05 | 0.405 | `GAAAGCA` |
| GAAASCA | chr2 | + | 54660483 | 54660489 | 5.85e-05 | 0.405 | `GAAAGCA` |
| GAAASCA | chr2 | + | 70204918 | 70204924 | 5.85e-05 | 0.405 | `GAAAGCA` |
| GAAASCA | chr2 | − | 111643118 | 111643124 | 5.85e-05 | 0.405 | `GAAAGCA` |
| GAAASCA | chr2 | − | 122173764 | 122173770 | 5.85e-05 | 0.405 | `GAAAGCA` |
| GAAASCA | chr2 | + | 156082332 | 156082338 | 5.85e-05 | 0.405 | `GAAAGCA` |
| GAAASCA | chr2 | − | 156965515 | 156965521 | 5.85e-05 | 0.405 | `GAAAGCA` |
| GAAASCA | chr2 | − | 157978417 | 157978423 | 5.85e-05 | 0.405 | `GAAAGCA` |
| GAAASCA | chr2 | − | 169060192 | 169060198 | 5.85e-05 | 0.405 | `GAAAGCA` |
| GAAASCA | chr2 | − | 179103660 | 179103666 | 5.85e-05 | 0.405 | `GAAAGCA` |
| GAAASCA | chr2 | − | 179103706 | 179103712 | 5.85e-05 | 0.405 | `GAAAGCA` |
| GAAASCA | chr2 | + | 185463498 | 185463504 | 5.85e-05 | 0.405 | `GAAAGCA` |
| GAAASCA | chr2 | − | 191537483 | 191537489 | 5.85e-05 | 0.405 | `GAAAGCA` |
| GAAASCA | chr2 | − | 191593365 | 191593371 | 5.85e-05 | 0.405 | `GAAAGCA` |
| GAAASCA | chr2 | − | 201696914 | 201696920 | 5.85e-05 | 0.405 | `GAAAGCA` |
| GAAASCA | chr2 | − | 201697017 | 201697023 | 5.85e-05 | 0.405 | `GAAAGCA` |
| GAAASCA | chr2 | + | 227409379 | 227409385 | 5.85e-05 | 0.405 | `GAAAGCA` |
| GAAASCA | chr2 | + | 230830914 | 230830920 | 5.85e-05 | 0.405 | `GAAAGCA` |
| GAAASCA | chr2 | + | 231232239 | 231232245 | 5.85e-05 | 0.405 | `GAAAGCA` |
| GAAASCA | chr2 | + | 232037247 | 232037253 | 5.85e-05 | 0.405 | `GAAAGCA` |
| GAAASCA | chr2 | − | 232235271 | 232235277 | 5.85e-05 | 0.405 | `GAAAGCA` |
| GAAASCA | chr2 | + | 232600064 | 232600070 | 5.85e-05 | 0.405 | `GAAAGCA` |
| GAAASCA | chr2 | + | 239891000 | 239891006 | 5.85e-05 | 0.405 | `GAAAGCA` |
| GAAASCA | chr3 | + | 45612960 | 45612966 | 5.85e-05 | 0.405 | `GAAAGCA` |
| GAAASCA | chr3 | + | 13430626 | 13430632 | 5.85e-05 | 0.405 | `GAAAGCA` |
| GAAASCA | chr3 | − | 23670327 | 23670333 | 5.85e-05 | 0.405 | `GAAAGCA` |
| GAAASCA | chr3 | − | 45978638 | 45978644 | 5.85e-05 | 0.405 | `GAAAGCA` |
| GAAASCA | chr3 | + | 48489530 | 48489536 | 5.85e-05 | 0.405 | `GAAAGCA` |
| GAAASCA | chr3 | − | 53111962 | 53111968 | 5.85e-05 | 0.405 | `GAAAGCA` |
| GAAASCA | chr3 | − | 58002706 | 58002712 | 5.85e-05 | 0.405 | `GAAAGCA` |
| GAAASCA | chr3 | + | 99734256 | 99734262 | 5.85e-05 | 0.405 | `GAAAGCA` |
| GAAASCA | chr3 | + | 99757935 | 99757941 | 5.85e-05 | 0.405 | `GAAAGCA` |
| GAAASCA | chr3 | − | 109326053 | 109326059 | 5.85e-05 | 0.405 | `GAAAGCA` |
| GAAASCA | chr3 | + | 109590216 | 109590222 | 5.85e-05 | 0.405 | `GAAAGCA` |
| GAAASCA | chr3 | + | 128952361 | 128952367 | 5.85e-05 | 0.405 | `GAAAGCA` |
| GAAASCA | chr3 | − | 157886334 | 157886340 | 5.85e-05 | 0.405 | `GAAAGCA` |
| GAAASCA | chr3 | − | 170496660 | 170496666 | 5.85e-05 | 0.405 | `GAAAGCA` |
| GAAASCA | chr3 | − | 178552484 | 178552490 | 5.85e-05 | 0.405 | `GAAAGCA` |
| GAAASCA | chr3 | + | 178552546 | 178552552 | 5.85e-05 | 0.405 | `GAAAGCA` |
| GAAASCA | chr3 | + | 196746240 | 196746246 | 5.85e-05 | 0.405 | `GAAAGCA` |
| GAAASCA | chr3 | + | 199168553 | 199168559 | 5.85e-05 | 0.405 | `GAAAGCA` |
| GAAASCA | chr4 | − | 2759023 | 2759029 | 5.85e-05 | 0.405 | `GAAAGCA` |
| GAAASCA | chr4 | − | 39876584 | 39876590 | 5.85e-05 | 0.405 | `GAAAGCA` |
| GAAASCA | chr4 | − | 77338920 | 77338926 | 5.85e-05 | 0.405 | `GAAAGCA` |
| GAAASCA | chr4 | + | 77340048 | 77340054 | 5.85e-05 | 0.405 | `GAAAGCA` |
| GAAASCA | chr4 | − | 79779176 | 79779182 | 5.85e-05 | 0.405 | `GAAAGCA` |
| GAAASCA | chr4 | − | 102159496 | 102159502 | 5.85e-05 | 0.405 | `GAAAGCA` |
| GAAASCA | chr4 | + | 114705638 | 114705644 | 5.85e-05 | 0.405 | `GAAAGCA` |
| GAAASCA | chr4 | + | 148824965 | 148824971 | 5.85e-05 | 0.405 | `GAAAGCA` |
| GAAASCA | chr4 | + | 185440037 | 185440043 | 5.85e-05 | 0.405 | `GAAAGCA` |
| GAAASCA | chr4 | − | 185505508 | 185505514 | 5.85e-05 | 0.405 | `GAAAGCA` |
| GAAASCA | chr4 | + | 185505346 | 185505352 | 5.85e-05 | 0.405 | `GAAAGCA` |
| GAAASCA | chr4 | + | 185632680 | 185632686 | 5.85e-05 | 0.405 | `GAAAGCA` |
| GAAASCA | chr5 | + | 5414547 | 5414553 | 5.85e-05 | 0.405 | `GAAAGCA` |
| GAAASCA | chr5 | + | 32567461 | 32567467 | 5.85e-05 | 0.405 | `GAAAGCA` |
| GAAASCA | chr5 | + | 40443035 | 40443041 | 5.85e-05 | 0.405 | `GAAAGCA` |
| GAAASCA | chr5 | + | 55474810 | 55474816 | 5.85e-05 | 0.405 | `GAAAGCA` |
| GAAASCA | chr5 | − | 78089812 | 78089818 | 5.85e-05 | 0.405 | `GAAAGCA` |
| GAAASCA | chr5 | − | 86448941 | 86448947 | 5.85e-05 | 0.405 | `GAAAGCA` |
| GAAASCA | chr5 | + | 106759056 | 106759062 | 5.85e-05 | 0.405 | `GAAAGCA` |
| GAAASCA | chr5 | + | 107746304 | 107746310 | 5.85e-05 | 0.405 | `GAAAGCA` |
| GAAASCA | chr5 | + | 138748948 | 138748954 | 5.85e-05 | 0.405 | `GAAAGCA` |
| GAAASCA | chr5 | + | 139205071 | 139205077 | 5.85e-05 | 0.405 | `GAAAGCA` |
| GAAASCA | chr5 | − | 156291733 | 156291739 | 5.85e-05 | 0.405 | `GAAAGCA` |
| GAAASCA | chr5 | + | 180582975 | 180582981 | 5.85e-05 | 0.405 | `GAAAGCA` |
| GAAASCA | chr6 | + | 349823 | 349829 | 5.85e-05 | 0.405 | `GAAAGCA` |
| GAAASCA | chr6 | − | 7827772 | 7827778 | 5.85e-05 | 0.405 | `GAAAGCA` |
| GAAASCA | chr6 | − | 7828005 | 7828011 | 5.85e-05 | 0.405 | `GAAAGCA` |
| GAAASCA | chr6 | + | 7831827 | 7831833 | 5.85e-05 | 0.405 | `GAAAGCA` |
| GAAASCA | chr6 | + | 7838878 | 7838884 | 5.85e-05 | 0.405 | `GAAAGCA` |
| GAAASCA | chr6 | + | 7862412 | 7862418 | 5.85e-05 | 0.405 | `GAAAGCA` |
| GAAASCA | chr6 | + | 16612506 | 16612512 | 5.85e-05 | 0.405 | `GAAAGCA` |
| GAAASCA | chr6 | + | 21696986 | 21696992 | 5.85e-05 | 0.405 | `GAAAGCA` |
| GAAASCA | chr6 | − | 24991949 | 24991955 | 5.85e-05 | 0.405 | `GAAAGCA` |
| GAAASCA | chr6 | − | 26139721 | 26139727 | 5.85e-05 | 0.405 | `GAAAGCA` |
| GAAASCA | chr6 | − | 26152186 | 26152192 | 5.85e-05 | 0.405 | `GAAAGCA` |
| GAAASCA | chr6 | + | 31239730 | 31239736 | 5.85e-05 | 0.405 | `GAAAGCA` |
| GAAASCA | chr6 | + | 31239837 | 31239843 | 5.85e-05 | 0.405 | `GAAAGCA` |
| GAAASCA | chr6 | + | 31430560 | 31430566 | 5.85e-05 | 0.405 | `GAAAGCA` |
| GAAASCA | chr6 | + | 31430560 | 31430566 | 5.85e-05 | 0.405 | `GAAAGCA` |
| GAAASCA | chr6 | − | 31431029 | 31431035 | 5.85e-05 | 0.405 | `GAAAGCA` |
| GAAASCA | chr6 | − | 32047770 | 32047776 | 5.85e-05 | 0.405 | `GAAAGCA` |
| GAAASCA | chr6 | − | 32733881 | 32733887 | 5.85e-05 | 0.405 | `GAAAGCA` |
| GAAASCA | chr6 | − | 32734229 | 32734235 | 5.85e-05 | 0.405 | `GAAAGCA` |
| GAAASCA | chr6 | + | 36828837 | 36828843 | 5.85e-05 | 0.405 | `GAAAGCA` |
| GAAASCA | chr6 | − | 41782071 | 41782077 | 5.85e-05 | 0.405 | `GAAAGCA` |
| GAAASCA | chr6 | + | 44322860 | 44322866 | 5.85e-05 | 0.405 | `GAAAGCA` |
| GAAASCA | chr6 | + | 44330001 | 44330007 | 5.85e-05 | 0.405 | `GAAAGCA` |
| GAAASCA | chr6 | − | 51969348 | 51969354 | 5.85e-05 | 0.405 | `GAAAGCA` |
| GAAASCA | chr6 | + | 74287188 | 74287194 | 5.85e-05 | 0.405 | `GAAAGCA` |
| GAAASCA | chr6 | − | 86440060 | 86440066 | 5.85e-05 | 0.405 | `GAAAGCA` |
| GAAASCA | chr6 | − | 106656617 | 106656623 | 5.85e-05 | 0.405 | `GAAAGCA` |
| GAAASCA | chr6 | − | 106664236 | 106664242 | 5.85e-05 | 0.405 | `GAAAGCA` |
| GAAASCA | chr6 | + | 106670151 | 106670157 | 5.85e-05 | 0.405 | `GAAAGCA` |
| GAAASCA | chr6 | − | 106718854 | 106718860 | 5.85e-05 | 0.405 | `GAAAGCA` |
| GAAASCA | chr6 | − | 107887348 | 107887354 | 5.85e-05 | 0.405 | `GAAAGCA` |
| GAAASCA | chr6 | + | 133181391 | 133181397 | 5.85e-05 | 0.405 | `GAAAGCA` |
| GAAASCA | chr6 | + | 134610489 | 134610495 | 5.85e-05 | 0.405 | `GAAAGCA` |
| GAAASCA | chr6 | − | 157061230 | 157061236 | 5.85e-05 | 0.405 | `GAAAGCA` |
| GAAASCA | chr7 | − | 7951145 | 7951151 | 5.85e-05 | 0.405 | `GAAAGCA` |
| GAAASCA | chr7 | − | 24924392 | 24924398 | 5.85e-05 | 0.405 | `GAAAGCA` |
| GAAASCA | chr7 | − | 26195703 | 26195709 | 5.85e-05 | 0.405 | `GAAAGCA` |
| GAAASCA | chr7 | − | 30637266 | 30637272 | 5.85e-05 | 0.405 | `GAAAGCA` |
| GAAASCA | chr7 | + | 41991122 | 41991128 | 5.85e-05 | 0.405 | `GAAAGCA` |
| GAAASCA | chr7 | − | 44071156 | 44071162 | 5.85e-05 | 0.405 | `GAAAGCA` |
| GAAASCA | chr7 | + | 55604957 | 55604963 | 5.85e-05 | 0.405 | `GAAAGCA` |
| GAAASCA | chr7 | − | 73263471 | 73263477 | 5.85e-05 | 0.405 | `GAAAGCA` |
| GAAASCA | chr7 | + | 73281701 | 73281707 | 5.85e-05 | 0.405 | `GAAAGCA` |
| GAAASCA | chr7 | + | 135694935 | 135694941 | 5.85e-05 | 0.405 | `GAAAGCA` |
| GAAASCA | chr8 | + | 8179607 | 8179613 | 5.85e-05 | 0.405 | `GAAAGCA` |
| GAAASCA | chr8 | − | 38934138 | 38934144 | 5.85e-05 | 0.405 | `GAAAGCA` |
| GAAASCA | chr8 | + | 38934259 | 38934265 | 5.85e-05 | 0.405 | `GAAAGCA` |
| GAAASCA | chr8 | − | 57149949 | 57149955 | 5.85e-05 | 0.405 | `GAAAGCA` |
| GAAASCA | chr8 | − | 61978897 | 61978903 | 5.85e-05 | 0.405 | `GAAAGCA` |
| GAAASCA | chr8 | + | 61979226 | 61979232 | 5.85e-05 | 0.405 | `GAAAGCA` |
| GAAASCA | chr8 | + | 61987408 | 61987414 | 5.85e-05 | 0.405 | `GAAAGCA` |
| GAAASCA | chr8 | + | 62004602 | 62004608 | 5.85e-05 | 0.405 | `GAAAGCA` |
| GAAASCA | chr8 | − | 103666816 | 103666822 | 5.85e-05 | 0.405 | `GAAAGCA` |
| GAAASCA | chr8 | − | 119363384 | 119363390 | 5.85e-05 | 0.405 | `GAAAGCA` |
| GAAASCA | chr8 | + | 125688968 | 125688974 | 5.85e-05 | 0.405 | `GAAAGCA` |
| GAAASCA | chr8 | + | 125688997 | 125689003 | 5.85e-05 | 0.405 | `GAAAGCA` |
| GAAASCA | chr8 | + | 125718977 | 125718983 | 5.85e-05 | 0.405 | `GAAAGCA` |
| GAAASCA | chr8 | + | 126521229 | 126521235 | 5.85e-05 | 0.405 | `GAAAGCA` |
| GAAASCA | chr8 | + | 126683506 | 126683512 | 5.85e-05 | 0.405 | `GAAAGCA` |
| GAAASCA | chr8 | + | 126689484 | 126689490 | 5.85e-05 | 0.405 | `GAAAGCA` |
| GAAASCA | chr8 | − | 126730479 | 126730485 | 5.85e-05 | 0.405 | `GAAAGCA` |
| GAAASCA | chr8 | + | 129395549 | 129395555 | 5.85e-05 | 0.405 | `GAAAGCA` |
| GAAASCA | chr8 | − | 134563984 | 134563990 | 5.85e-05 | 0.405 | `GAAAGCA` |
| GAAASCA | chr8 | − | 135683436 | 135683442 | 5.85e-05 | 0.405 | `GAAAGCA` |
| GAAASCA | chr8 | + | 135683153 | 135683159 | 5.85e-05 | 0.405 | `GAAAGCA` |
| GAAASCA | chr9 | − | 3516606 | 3516612 | 5.85e-05 | 0.405 | `GAAAGCA` |
| GAAASCA | chr9 | − | 5548479 | 5548485 | 5.85e-05 | 0.405 | `GAAAGCA` |
| GAAASCA | chr9 | − | 85770720 | 85770726 | 5.85e-05 | 0.405 | `GAAAGCA` |
| GAAASCA | chr9 | − | 130685088 | 130685094 | 5.85e-05 | 0.405 | `GAAAGCA` |
| GAAASCA | chr9 | − | 131833409 | 131833415 | 5.85e-05 | 0.405 | `GAAAGCA` |
| GAAASCA | chrX | − | 7012178 | 7012184 | 5.85e-05 | 0.405 | `GAAAGCA` |
| GAAASCA | chrX | + | 7043471 | 7043477 | 5.85e-05 | 0.405 | `GAAAGCA` |
| GAAASCA | chrX | − | 12906313 | 12906319 | 5.85e-05 | 0.405 | `GAAAGCA` |
| GAAASCA | chrX | − | 96468142 | 96468148 | 5.85e-05 | 0.405 | `GAAAGCA` |
| GAAASCA | chrX | − | 96468503 | 96468509 | 5.85e-05 | 0.405 | `GAAAGCA` |
| GAAASCA | chrX | + | 96468315 | 96468321 | 5.85e-05 | 0.405 | `GAAAGCA` |
| GAAASCA | chrX | + | 141893811 | 141893817 | 5.85e-05 | 0.405 | `GAAAGCA` |
| GAAASCA | chrX | − | 151750410 | 151750416 | 5.85e-05 | 0.405 | `GAAAGCA` |
| GAAASCA | chr10 | − | 6430026 | 6430032 | 5.85e-05 | 0.405 | `GAAAGCA` |
| GAAASCA | chr10 | − | 11328920 | 11328926 | 5.85e-05 | 0.405 | `GAAAGCA` |
| GAAASCA | chr10 | − | 11368679 | 11368685 | 5.85e-05 | 0.405 | `GAAAGCA` |
| GAAASCA | chr10 | + | 45235425 | 45235431 | 5.85e-05 | 0.405 | `GAAAGCA` |
| GAAASCA | chr10 | + | 63326904 | 63326910 | 5.85e-05 | 0.405 | `GAAAGCA` |
| GAAASCA | chr10 | + | 64067348 | 64067354 | 5.85e-05 | 0.405 | `GAAAGCA` |
| GAAASCA | chr10 | + | 89840650 | 89840656 | 5.85e-05 | 0.405 | `GAAAGCA` |
| GAAASCA | chr10 | + | 89860835 | 89860841 | 5.85e-05 | 0.405 | `GAAAGCA` |
| GAAASCA | chr10 | − | 89912957 | 89912963 | 5.85e-05 | 0.405 | `GAAAGCA` |
| GAAASCA | chr10 | + | 90010516 | 90010522 | 5.85e-05 | 0.405 | `GAAAGCA` |
| GAAASCA | chr10 | + | 97581868 | 97581874 | 5.85e-05 | 0.405 | `GAAAGCA` |
| GAAASCA | chr10 | − | 112592924 | 112592930 | 5.85e-05 | 0.405 | `GAAAGCA` |
| GAAASCA | chr10 | − | 121413780 | 121413786 | 5.85e-05 | 0.405 | `GAAAGCA` |
| GAAASCA | chr11 | + | 1830874 | 1830880 | 5.85e-05 | 0.405 | `GAAAGCA` |
| GAAASCA | chr11 | − | 8666606 | 8666612 | 5.85e-05 | 0.405 | `GAAAGCA` |
| GAAASCA | chr11 | − | 33872022 | 33872028 | 5.85e-05 | 0.405 | `GAAAGCA` |
| GAAASCA | chr11 | − | 35105751 | 35105757 | 5.85e-05 | 0.405 | `GAAAGCA` |
| GAAASCA | chr11 | − | 58741067 | 58741073 | 5.85e-05 | 0.405 | `GAAAGCA` |
| GAAASCA | chr11 | + | 62379002 | 62379008 | 5.85e-05 | 0.405 | `GAAAGCA` |
| GAAASCA | chr11 | − | 64945948 | 64945954 | 5.85e-05 | 0.405 | `GAAAGCA` |
| GAAASCA | chr11 | − | 67790534 | 67790540 | 5.85e-05 | 0.405 | `GAAAGCA` |
| GAAASCA | chr11 | + | 101693313 | 101693319 | 5.85e-05 | 0.405 | `GAAAGCA` |
| GAAASCA | chr11 | + | 103274695 | 103274701 | 5.85e-05 | 0.405 | `GAAAGCA` |
| GAAASCA | chr11 | + | 122430309 | 122430315 | 5.85e-05 | 0.405 | `GAAAGCA` |
| GAAASCA | chr12 | + | 12054482 | 12054488 | 5.85e-05 | 0.405 | `GAAAGCA` |
| GAAASCA | chr12 | + | 14325098 | 14325104 | 5.85e-05 | 0.405 | `GAAAGCA` |
| GAAASCA | chr12 | + | 22588731 | 22588737 | 5.85e-05 | 0.405 | `GAAAGCA` |
| GAAASCA | chr12 | − | 46563458 | 46563464 | 5.85e-05 | 0.405 | `GAAAGCA` |
| GAAASCA | chr12 | + | 52132124 | 52132130 | 5.85e-05 | 0.405 | `GAAAGCA` |
| GAAASCA | chr12 | + | 52965956 | 52965962 | 5.85e-05 | 0.405 | `GAAAGCA` |
| GAAASCA | chr12 | − | 55014332 | 55014338 | 5.85e-05 | 0.405 | `GAAAGCA` |
| GAAASCA | chr12 | − | 74640124 | 74640130 | 5.85e-05 | 0.405 | `GAAAGCA` |
| GAAASCA | chr12 | − | 75061643 | 75061649 | 5.85e-05 | 0.405 | `GAAAGCA` |
| GAAASCA | chr12 | + | 75061767 | 75061773 | 5.85e-05 | 0.405 | `GAAAGCA` |
| GAAASCA | chr12 | − | 107486148 | 107486154 | 5.85e-05 | 0.405 | `GAAAGCA` |
| GAAASCA | chr12 | − | 107486148 | 107486154 | 5.85e-05 | 0.405 | `GAAAGCA` |
| GAAASCA | chr12 | + | 107556259 | 107556265 | 5.85e-05 | 0.405 | `GAAAGCA` |
| GAAASCA | chr12 | + | 107556346 | 107556352 | 5.85e-05 | 0.405 | `GAAAGCA` |
| GAAASCA | chr12 | − | 115481767 | 115481773 | 5.85e-05 | 0.405 | `GAAAGCA` |
| GAAASCA | chr12 | − | 119117661 | 119117667 | 5.85e-05 | 0.405 | `GAAAGCA` |
| GAAASCA | chr12 | + | 121914719 | 121914725 | 5.85e-05 | 0.405 | `GAAAGCA` |
| GAAASCA | chr12 | + | 123972235 | 123972241 | 5.85e-05 | 0.405 | `GAAAGCA` |
| GAAASCA | chr13 | − | 26922985 | 26922991 | 5.85e-05 | 0.405 | `GAAAGCA` |
| GAAASCA | chr13 | − | 33015047 | 33015053 | 5.85e-05 | 0.405 | `GAAAGCA` |
| GAAASCA | chr13 | + | 45319886 | 45319892 | 5.85e-05 | 0.405 | `GAAAGCA` |
| GAAASCA | chr13 | − | 45849839 | 45849845 | 5.85e-05 | 0.405 | `GAAAGCA` |
| GAAASCA | chr13 | − | 51666622 | 51666628 | 5.85e-05 | 0.405 | `GAAAGCA` |
| GAAASCA | chr13 | + | 52089581 | 52089587 | 5.85e-05 | 0.405 | `GAAAGCA` |
| GAAASCA | chr13 | + | 76218199 | 76218205 | 5.85e-05 | 0.405 | `GAAAGCA` |
| GAAASCA | chr13 | + | 76799488 | 76799494 | 5.85e-05 | 0.405 | `GAAAGCA` |
| GAAASCA | chr13 | − | 97962877 | 97962883 | 5.85e-05 | 0.405 | `GAAAGCA` |
| GAAASCA | chr13 | + | 97962852 | 97962858 | 5.85e-05 | 0.405 | `GAAAGCA` |
| GAAASCA | chr13 | + | 98882825 | 98882831 | 5.85e-05 | 0.405 | `GAAAGCA` |
| GAAASCA | chr13 | − | 101836943 | 101836949 | 5.85e-05 | 0.405 | `GAAAGCA` |
| GAAASCA | chr14 | − | 20779674 | 20779680 | 5.85e-05 | 0.405 | `GAAAGCA` |
| GAAASCA | chr14 | − | 61198634 | 61198640 | 5.85e-05 | 0.405 | `GAAAGCA` |
| GAAASCA | chr14 | − | 80495689 | 80495695 | 5.85e-05 | 0.405 | `GAAAGCA` |
| GAAASCA | chr14 | − | 92240688 | 92240694 | 5.85e-05 | 0.405 | `GAAAGCA` |
| GAAASCA | chr14 | + | 96424393 | 96424399 | 5.85e-05 | 0.405 | `GAAAGCA` |
| GAAASCA | chr14 | − | 96437463 | 96437469 | 5.85e-05 | 0.405 | `GAAAGCA` |
| GAAASCA | chr15 | + | 29425705 | 29425711 | 5.85e-05 | 0.405 | `GAAAGCA` |
| GAAASCA | chr15 | − | 42808887 | 42808893 | 5.85e-05 | 0.405 | `GAAAGCA` |
| GAAASCA | chr15 | − | 43280126 | 43280132 | 5.85e-05 | 0.405 | `GAAAGCA` |
| GAAASCA | chr15 | − | 55210618 | 55210624 | 5.85e-05 | 0.405 | `GAAAGCA` |
| GAAASCA | chr15 | − | 61583843 | 61583849 | 5.85e-05 | 0.405 | `GAAAGCA` |
| GAAASCA | chr15 | − | 62962427 | 62962433 | 5.85e-05 | 0.405 | `GAAAGCA` |
| GAAASCA | chr15 | + | 70554570 | 70554576 | 5.85e-05 | 0.405 | `GAAAGCA` |
| GAAASCA | chr15 | − | 73126460 | 73126466 | 5.85e-05 | 0.405 | `GAAAGCA` |
| GAAASCA | chr15 | − | 84035793 | 84035799 | 5.85e-05 | 0.405 | `GAAAGCA` |
| GAAASCA | chr16 | − | 1951423 | 1951429 | 5.85e-05 | 0.405 | `GAAAGCA` |
| GAAASCA | chr16 | + | 8937792 | 8937798 | 5.85e-05 | 0.405 | `GAAAGCA` |
| GAAASCA | chr16 | + | 20793340 | 20793346 | 5.85e-05 | 0.405 | `GAAAGCA` |
| GAAASCA | chr16 | − | 23253184 | 23253190 | 5.85e-05 | 0.405 | `GAAAGCA` |
| GAAASCA | chr16 | − | 80674172 | 80674178 | 5.85e-05 | 0.405 | `GAAAGCA` |
| GAAASCA | chr16 | + | 87245552 | 87245558 | 5.85e-05 | 0.405 | `GAAAGCA` |
| GAAASCA | chr17 | − | 1457379 | 1457385 | 5.85e-05 | 0.405 | `GAAAGCA` |
| GAAASCA | chr17 | − | 3561212 | 3561218 | 5.85e-05 | 0.405 | `GAAAGCA` |
| GAAASCA | chr17 | + | 8017308 | 8017314 | 5.85e-05 | 0.405 | `GAAAGCA` |
| GAAASCA | chr17 | + | 8227052 | 8227058 | 5.85e-05 | 0.405 | `GAAAGCA` |
| GAAASCA | chr17 | − | 37984273 | 37984279 | 5.85e-05 | 0.405 | `GAAAGCA` |
| GAAASCA | chr17 | − | 43168058 | 43168064 | 5.85e-05 | 0.405 | `GAAAGCA` |
| GAAASCA | chr17 | + | 45581072 | 45581078 | 5.85e-05 | 0.405 | `GAAAGCA` |
| GAAASCA | chr17 | + | 45581202 | 45581208 | 5.85e-05 | 0.405 | `GAAAGCA` |
| GAAASCA | chr17 | + | 46585770 | 46585776 | 5.85e-05 | 0.405 | `GAAAGCA` |
| GAAASCA | chr17 | − | 52789256 | 52789262 | 5.85e-05 | 0.405 | `GAAAGCA` |
| GAAASCA | chr17 | − | 52790112 | 52790118 | 5.85e-05 | 0.405 | `GAAAGCA` |
| GAAASCA | chr17 | − | 52790341 | 52790347 | 5.85e-05 | 0.405 | `GAAAGCA` |
| GAAASCA | chr17 | − | 55274105 | 55274111 | 5.85e-05 | 0.405 | `GAAAGCA` |
| GAAASCA | chr17 | + | 55273937 | 55273943 | 5.85e-05 | 0.405 | `GAAAGCA` |
| GAAASCA | chr17 | − | 60550203 | 60550209 | 5.85e-05 | 0.405 | `GAAAGCA` |
| GAAASCA | chr17 | + | 71284686 | 71284692 | 5.85e-05 | 0.405 | `GAAAGCA` |
| GAAASCA | chr18 | + | 667196 | 667202 | 5.85e-05 | 0.405 | `GAAAGCA` |
| GAAASCA | chr18 | + | 3583784 | 3583790 | 5.85e-05 | 0.405 | `GAAAGCA` |
| GAAASCA | chr18 | − | 9663584 | 9663590 | 5.85e-05 | 0.405 | `GAAAGCA` |
| GAAASCA | chr18 | + | 11149965 | 11149971 | 5.85e-05 | 0.405 | `GAAAGCA` |
| GAAASCA | chr18 | − | 42007651 | 42007657 | 5.85e-05 | 0.405 | `GAAAGCA` |
| GAAASCA | chr18 | − | 59755260 | 59755266 | 5.85e-05 | 0.405 | `GAAAGCA` |
| GAAASCA | chr19 | + | 1028201 | 1028207 | 5.85e-05 | 0.405 | `GAAAGCA` |
| GAAASCA | chr19 | − | 2562082 | 2562088 | 5.85e-05 | 0.405 | `GAAAGCA` |
| GAAASCA | chr19 | + | 2650025 | 2650031 | 5.85e-05 | 0.405 | `GAAAGCA` |
| GAAASCA | chr19 | + | 11896740 | 11896746 | 5.85e-05 | 0.405 | `GAAAGCA` |
| GAAASCA | chr19 | + | 14353043 | 14353049 | 5.85e-05 | 0.405 | `GAAAGCA` |
| GAAASCA | chr19 | + | 17494924 | 17494930 | 5.85e-05 | 0.405 | `GAAAGCA` |
| GAAASCA | chr19 | + | 17904394 | 17904400 | 5.85e-05 | 0.405 | `GAAAGCA` |
| GAAASCA | chr19 | − | 40900092 | 40900098 | 5.85e-05 | 0.405 | `GAAAGCA` |
| GAAASCA | chr19 | − | 40900215 | 40900221 | 5.85e-05 | 0.405 | `GAAAGCA` |
| GAAASCA | chr19 | − | 45553703 | 45553709 | 5.85e-05 | 0.405 | `GAAAGCA` |
| GAAASCA | chr19 | + | 54070562 | 54070568 | 5.85e-05 | 0.405 | `GAAAGCA` |
| GAAASCA | chr19 | − | 54157768 | 54157774 | 5.85e-05 | 0.405 | `GAAAGCA` |
| GAAASCA | chr19 | + | 57100236 | 57100242 | 5.85e-05 | 0.405 | `GAAAGCA` |
| GAAASCA | chr19 | − | 60838117 | 60838123 | 5.85e-05 | 0.405 | `GAAAGCA` |
| GAAASCA | chr20 | + | 3587714 | 3587720 | 5.85e-05 | 0.405 | `GAAAGCA` |
| GAAASCA | chr20 | + | 24771752 | 24771758 | 5.85e-05 | 0.405 | `GAAAGCA` |
| GAAASCA | chr20 | − | 36937771 | 36937777 | 5.85e-05 | 0.405 | `GAAAGCA` |
| GAAASCA | chr20 | − | 47337867 | 47337873 | 5.85e-05 | 0.405 | `GAAAGCA` |
| GAAASCA | chr20 | − | 47339043 | 47339049 | 5.85e-05 | 0.405 | `GAAAGCA` |
| GAAASCA | chr20 | − | 47339343 | 47339349 | 5.85e-05 | 0.405 | `GAAAGCA` |
| GAAASCA | chr20 | + | 47735388 | 47735394 | 5.85e-05 | 0.405 | `GAAAGCA` |
| GAAASCA | chr20 | + | 51632041 | 51632047 | 5.85e-05 | 0.405 | `GAAAGCA` |
| GAAASCA | chr20 | − | 51702200 | 51702206 | 5.85e-05 | 0.405 | `GAAAGCA` |
| GAAASCA | chr20 | + | 51702423 | 51702429 | 5.85e-05 | 0.405 | `GAAAGCA` |
| GAAASCA | chr20 | − | 61112387 | 61112393 | 5.85e-05 | 0.405 | `GAAAGCA` |
| GAAASCA | chr20 | − | 61830307 | 61830313 | 5.85e-05 | 0.405 | `GAAAGCA` |
| GAAASCA | chr21 | + | 25866084 | 25866090 | 5.85e-05 | 0.405 | `GAAAGCA` |
| GAAASCA | chr21 | + | 41739689 | 41739695 | 5.85e-05 | 0.405 | `GAAAGCA` |
| GAAASCA | chr22 | − | 27525220 | 27525226 | 5.85e-05 | 0.405 | `GAAAGCA` |
| GAAASCA | chr22 | − | 16141407 | 16141413 | 5.85e-05 | 0.405 | `GAAAGCA` |
| GAAASCA | chr22 | + | 27520592 | 27520598 | 5.85e-05 | 0.405 | `GAAAGCA` |
| GAAASCA | chr22 | − | 35588489 | 35588495 | 5.85e-05 | 0.405 | `GAAAGCA` |
| GAAASCA | chr22 | + | 38038151 | 38038157 | 5.85e-05 | 0.405 | `GAAAGCA` |

---

**DEBUGGING INFORMATION**


---

Command line:

```
/ebi/sw/MEME/VM-cluster410/meme-versions/4.10.0/bin/fimo --parse-genomic-coord --verbosity 1 --oc fimo_out_13 --bgfile ./background --motif GAAASCA dreme_out/dreme.xml ./Supplementary_Table_1.500bp.fa
```

Settings:

```
|  |  |  |
| --- | --- | --- |
| output directory = fimo_out_13 | MEME file name = dreme_out/dreme.xml | sequence file name = ./Supplementary_Table_1.500bp.fa |
| background file name = ./background | allow clobber = true | compute q-values = true |
| parse genomic coord. = true | text only = false | scan both strands = true |
| max sequence length = 250000000 | output threshold = 0.0001 | threshold type = p-value |
| max stored scores = 100000 | pseudocount = 0.1 | verbosity = 1 |
| selected motif = GAAASCA |  |  |
```

This information can be useful in the event you wish to report a
problem with the FIMO software.

---

**Go to top**
