# Supplementary material for: The MEME Suite
Source: Nucleic Acids Res. 2015 May 7;43(Web Server issue):W39–49. doi: 10.1093/nar/gkv416 (PMC4489269; doi:10.1093/nar/gkv416)
Supplement: SUPPLEMENTARY DATA [file supp_gkv416_nar-00283-web-b-2015-File005.zip › case4/meme-chip/fimo_out_15/fimo.html]

FIMO Results


---

|  |  |  |
| --- | --- | --- |
| **Database and Motifs** | **High-scoring Motif Occurrences** | **Debugging Information** |

  
  

---

**FIMO - Motif search tool**


---

FIMO version 4.10.0,
(Release date: Wed May 21 10:35:36 2014 +1000)

For further information on how to interpret these results
or to get a copy of the FIMO software please access
http://meme.nbcr.net

If you use FIMO in your research, please cite the following paper:  
Charles E. Grant, Timothy L. Bailey, and William Stafford Noble,
"FIMO: Scanning for occurrences of a given motif",
*Bioinformatics*, **27**(7):1017-1018, 2011.
[full text]

---

**DATABASE AND MOTIFS**


---

DATABASE
./Supplementary\_Table\_1.500bp.fa  
Database contains
2776
sequences,
1388000
residues

MOTIFS
dreme\_out/dreme.xml
(nucleotide)

| MOTIF | WIDTH | BEST POSSIBLE MATCH |
| --- | --- | --- |
| GGAARY | 6 | GGAAGT |
| AVTGAAA | 7 | ACTGAAA |
| RCAGCTGY | 8 | GCAGCTGC |
| AKAAAH | 6 | AGAAAA |
| RAGKTCA | 7 | GAGGTCA |
| CMCAGM | 6 | CCCAGC |
| CCCCRCCC | 8 | CCCCGCCC |
| AAATR | 5 | AAATG |
| GAAASCA | 7 | GAAAGCA |
| CCGSCTCC | 8 | CCGCCTCC |
| CCWCCTGC | 8 | CCACCTGC |

Random model letter frequencies
(from ./background):
  
A 0.241 C 0.259 G 0.259 T 0.241

---

**SECTION I: HIGH-SCORING MOTIF OCCURRENCES**


---

- There were
  355
  motif occurrences with a
  p-value less than
  0.0001.
- The p-value of a motif occurrence is defined as the
  probability of a random sequence of the same length as the motif
  matching that position of the sequence with as good or better a score.
- The score for the match of a position in a sequence to a motif
  is computed by summing the appropriate entries from each column of
  the position-dependent scoring matrix that represents the motif.
- The q-value of a motif occurrence is defined as the
  false discovery rate if the occurrence is accepted as significant.
- The table is sorted by increasing p-value.

| Motif | Sequence Name | Strand | Start | End | p-value | q-value | Matched Sequence |
| --- | --- | --- | --- | --- | --- | --- | --- |
| CCGSCTCC | chr1 | − | 26819941 | 26819948 | 1.9e-05 | 0.243 | `CCGCCTCC` |
| CCGSCTCC | chr1 | + | 28374544 | 28374551 | 1.9e-05 | 0.243 | `CCGCCTCC` |
| CCGSCTCC | chr1 | + | 40929839 | 40929846 | 1.9e-05 | 0.243 | `CCGCCTCC` |
| CCGSCTCC | chr1 | − | 110682905 | 110682912 | 1.9e-05 | 0.243 | `CCGCCTCC` |
| CCGSCTCC | chr1 | + | 111548201 | 111548208 | 1.9e-05 | 0.243 | `CCGCCTCC` |
| CCGSCTCC | chr1 | + | 153201326 | 153201333 | 1.9e-05 | 0.243 | `CCGCCTCC` |
| CCGSCTCC | chr1 | + | 154453257 | 154453264 | 1.9e-05 | 0.243 | `CCGCCTCC` |
| CCGSCTCC | chr1 | − | 155413349 | 155413356 | 1.9e-05 | 0.243 | `CCGCCTCC` |
| CCGSCTCC | chr1 | − | 161558372 | 161558379 | 1.9e-05 | 0.243 | `CCGCCTCC` |
| CCGSCTCC | chr2 | − | 9699479 | 9699486 | 1.9e-05 | 0.243 | `CCGCCTCC` |
| CCGSCTCC | chr2 | − | 65513094 | 65513101 | 1.9e-05 | 0.243 | `CCGCCTCC` |
| CCGSCTCC | chr2 | − | 65513452 | 65513459 | 1.9e-05 | 0.243 | `CCGCCTCC` |
| CCGSCTCC | chr2 | − | 74535505 | 74535512 | 1.9e-05 | 0.243 | `CCGCCTCC` |
| CCGSCTCC | chr2 | + | 174538557 | 174538564 | 1.9e-05 | 0.243 | `CCGCCTCC` |
| CCGSCTCC | chr2 | + | 177837732 | 177837739 | 1.9e-05 | 0.243 | `CCGCCTCC` |
| CCGSCTCC | chr2 | + | 198073175 | 198073182 | 1.9e-05 | 0.243 | `CCGCCTCC` |
| CCGSCTCC | chr2 | + | 232281524 | 232281531 | 1.9e-05 | 0.243 | `CCGCCTCC` |
| CCGSCTCC | chr3 | + | 9413341 | 9413348 | 1.9e-05 | 0.243 | `CCGCCTCC` |
| CCGSCTCC | chr3 | + | 9413431 | 9413438 | 1.9e-05 | 0.243 | `CCGCCTCC` |
| CCGSCTCC | chr3 | + | 9413341 | 9413348 | 1.9e-05 | 0.243 | `CCGCCTCC` |
| CCGSCTCC | chr3 | + | 9413431 | 9413438 | 1.9e-05 | 0.243 | `CCGCCTCC` |
| CCGSCTCC | chr3 | + | 45705802 | 45705809 | 1.9e-05 | 0.243 | `CCGCCTCC` |
| CCGSCTCC | chr3 | − | 48931176 | 48931183 | 1.9e-05 | 0.243 | `CCGCCTCC` |
| CCGSCTCC | chr3 | − | 49133357 | 49133364 | 1.9e-05 | 0.243 | `CCGCCTCC` |
| CCGSCTCC | chr3 | − | 49133442 | 49133449 | 1.9e-05 | 0.243 | `CCGCCTCC` |
| CCGSCTCC | chr3 | − | 49732196 | 49732203 | 1.9e-05 | 0.243 | `CCGCCTCC` |
| CCGSCTCC | chr3 | − | 52005072 | 52005079 | 1.9e-05 | 0.243 | `CCGCCTCC` |
| CCGSCTCC | chr3 | + | 121296826 | 121296833 | 1.9e-05 | 0.243 | `CCGCCTCC` |
| CCGSCTCC | chr3 | + | 186699570 | 186699577 | 1.9e-05 | 0.243 | `CCGCCTCC` |
| CCGSCTCC | chr4 | − | 2758939 | 2758946 | 1.9e-05 | 0.243 | `CCGCCTCC` |
| CCGSCTCC | chr4 | − | 144654144 | 144654151 | 1.9e-05 | 0.243 | `CCGCCTCC` |
| CCGSCTCC | chr4 | − | 185632841 | 185632848 | 1.9e-05 | 0.243 | `CCGCCTCC` |
| CCGSCTCC | chr4 | + | 186301356 | 186301363 | 1.9e-05 | 0.243 | `CCGCCTCC` |
| CCGSCTCC | chr5 | + | 89741199 | 89741206 | 1.9e-05 | 0.243 | `CCGCCTCC` |
| CCGSCTCC | chr5 | + | 133996486 | 133996493 | 1.9e-05 | 0.243 | `CCGCCTCC` |
| CCGSCTCC | chr5 | − | 142763436 | 142763443 | 1.9e-05 | 0.243 | `CCGCCTCC` |
| CCGSCTCC | chr6 | + | 337402 | 337409 | 1.9e-05 | 0.243 | `CCGCCTCC` |
| CCGSCTCC | chr6 | + | 21696141 | 21696148 | 1.9e-05 | 0.243 | `CCGCCTCC` |
| CCGSCTCC | chr6 | + | 26070965 | 26070972 | 1.9e-05 | 0.243 | `CCGCCTCC` |
| CCGSCTCC | chr6 | − | 33047563 | 33047570 | 1.9e-05 | 0.243 | `CCGCCTCC` |
| CCGSCTCC | chr6 | − | 33276466 | 33276473 | 1.9e-05 | 0.243 | `CCGCCTCC` |
| CCGSCTCC | chr6 | + | 44333256 | 44333263 | 1.9e-05 | 0.243 | `CCGCCTCC` |
| CCGSCTCC | chr6 | − | 133177429 | 133177436 | 1.9e-05 | 0.243 | `CCGCCTCC` |
| CCGSCTCC | chr6 | + | 150011641 | 150011648 | 1.9e-05 | 0.243 | `CCGCCTCC` |
| CCGSCTCC | chr6 | − | 151753484 | 151753491 | 1.9e-05 | 0.243 | `CCGCCTCC` |
| CCGSCTCC | chr7 | − | 1510379 | 1510386 | 1.9e-05 | 0.243 | `CCGCCTCC` |
| CCGSCTCC | chr7 | − | 1510677 | 1510684 | 1.9e-05 | 0.243 | `CCGCCTCC` |
| CCGSCTCC | chr7 | − | 44072114 | 44072121 | 1.9e-05 | 0.243 | `CCGCCTCC` |
| CCGSCTCC | chr7 | + | 101823755 | 101823762 | 1.9e-05 | 0.243 | `CCGCCTCC` |
| CCGSCTCC | chr7 | + | 101823877 | 101823884 | 1.9e-05 | 0.243 | `CCGCCTCC` |
| CCGSCTCC | chr7 | − | 134504626 | 134504633 | 1.9e-05 | 0.243 | `CCGCCTCC` |
| CCGSCTCC | chr7 | + | 148952577 | 148952584 | 1.9e-05 | 0.243 | `CCGCCTCC` |
| CCGSCTCC | chr7 | + | 148952658 | 148952665 | 1.9e-05 | 0.243 | `CCGCCTCC` |
| CCGSCTCC | chr7 | − | 149733325 | 149733332 | 1.9e-05 | 0.243 | `CCGCCTCC` |
| CCGSCTCC | chr8 | − | 6553548 | 6553555 | 1.9e-05 | 0.243 | `CCGCCTCC` |
| CCGSCTCC | chr8 | + | 6553822 | 6553829 | 1.9e-05 | 0.243 | `CCGCCTCC` |
| CCGSCTCC | chr8 | − | 67507228 | 67507235 | 1.9e-05 | 0.243 | `CCGCCTCC` |
| CCGSCTCC | chr8 | − | 72918890 | 72918897 | 1.9e-05 | 0.243 | `CCGCCTCC` |
| CCGSCTCC | chr8 | − | 103945850 | 103945857 | 1.9e-05 | 0.243 | `CCGCCTCC` |
| CCGSCTCC | chr8 | − | 110415669 | 110415676 | 1.9e-05 | 0.243 | `CCGCCTCC` |
| CCGSCTCC | chr9 | − | 3516011 | 3516018 | 1.9e-05 | 0.243 | `CCGCCTCC` |
| CCGSCTCC | chr9 | + | 3516630 | 3516637 | 1.9e-05 | 0.243 | `CCGCCTCC` |
| CCGSCTCC | chr9 | + | 6671387 | 6671394 | 1.9e-05 | 0.243 | `CCGCCTCC` |
| CCGSCTCC | chr9 | + | 91269518 | 91269525 | 1.9e-05 | 0.243 | `CCGCCTCC` |
| CCGSCTCC | chr9 | + | 98020467 | 98020474 | 1.9e-05 | 0.243 | `CCGCCTCC` |
| CCGSCTCC | chr9 | + | 115212565 | 115212572 | 1.9e-05 | 0.243 | `CCGCCTCC` |
| CCGSCTCC | chr9 | + | 116151027 | 116151034 | 1.9e-05 | 0.243 | `CCGCCTCC` |
| CCGSCTCC | chr9 | + | 116151259 | 116151266 | 1.9e-05 | 0.243 | `CCGCCTCC` |
| CCGSCTCC | chr9 | − | 125141594 | 125141601 | 1.9e-05 | 0.243 | `CCGCCTCC` |
| CCGSCTCC | chr9 | − | 129253421 | 129253428 | 1.9e-05 | 0.243 | `CCGCCTCC` |
| CCGSCTCC | chr9 | + | 130685211 | 130685218 | 1.9e-05 | 0.243 | `CCGCCTCC` |
| CCGSCTCC | chr9 | + | 138959202 | 138959209 | 1.9e-05 | 0.243 | `CCGCCTCC` |
| CCGSCTCC | chrX | − | 24078314 | 24078321 | 1.9e-05 | 0.243 | `CCGCCTCC` |
| CCGSCTCC | chrX | − | 48661425 | 48661432 | 1.9e-05 | 0.243 | `CCGCCTCC` |
| CCGSCTCC | chrX | + | 53727674 | 53727681 | 1.9e-05 | 0.243 | `CCGCCTCC` |
| CCGSCTCC | chrX | − | 54087643 | 54087650 | 1.9e-05 | 0.243 | `CCGCCTCC` |
| CCGSCTCC | chr10 | − | 70495663 | 70495670 | 1.9e-05 | 0.243 | `CCGCCTCC` |
| CCGSCTCC | chr10 | − | 73703516 | 73703523 | 1.9e-05 | 0.243 | `CCGCCTCC` |
| CCGSCTCC | chr10 | + | 73727615 | 73727622 | 1.9e-05 | 0.243 | `CCGCCTCC` |
| CCGSCTCC | chr10 | + | 73727633 | 73727640 | 1.9e-05 | 0.243 | `CCGCCTCC` |
| CCGSCTCC | chr10 | + | 85889133 | 85889140 | 1.9e-05 | 0.243 | `CCGCCTCC` |
| CCGSCTCC | chr10 | + | 85889154 | 85889161 | 1.9e-05 | 0.243 | `CCGCCTCC` |
| CCGSCTCC | chr11 | + | 9438719 | 9438726 | 1.9e-05 | 0.243 | `CCGCCTCC` |
| CCGSCTCC | chr11 | + | 58102517 | 58102524 | 1.9e-05 | 0.243 | `CCGCCTCC` |
| CCGSCTCC | chr11 | − | 62405227 | 62405234 | 1.9e-05 | 0.243 | `CCGCCTCC` |
| CCGSCTCC | chr11 | + | 67796157 | 67796164 | 1.9e-05 | 0.243 | `CCGCCTCC` |
| CCGSCTCC | chr11 | + | 69210038 | 69210045 | 1.9e-05 | 0.243 | `CCGCCTCC` |
| CCGSCTCC | chr12 | − | 6513870 | 6513877 | 1.9e-05 | 0.243 | `CCGCCTCC` |
| CCGSCTCC | chr12 | − | 14818477 | 14818484 | 1.9e-05 | 0.243 | `CCGCCTCC` |
| CCGSCTCC | chr12 | − | 44407554 | 44407561 | 1.9e-05 | 0.243 | `CCGCCTCC` |
| CCGSCTCC | chr12 | + | 46493146 | 46493153 | 1.9e-05 | 0.243 | `CCGCCTCC` |
| CCGSCTCC | chr12 | + | 46493160 | 46493167 | 1.9e-05 | 0.243 | `CCGCCTCC` |
| CCGSCTCC | chr12 | + | 53664857 | 53664864 | 1.9e-05 | 0.243 | `CCGCCTCC` |
| CCGSCTCC | chr12 | − | 54798375 | 54798382 | 1.9e-05 | 0.243 | `CCGCCTCC` |
| CCGSCTCC | chr12 | − | 91063621 | 91063628 | 1.9e-05 | 0.243 | `CCGCCTCC` |
| CCGSCTCC | chr12 | + | 91063693 | 91063700 | 1.9e-05 | 0.243 | `CCGCCTCC` |
| CCGSCTCC | chr12 | − | 91063904 | 91063911 | 1.9e-05 | 0.243 | `CCGCCTCC` |
| CCGSCTCC | chr12 | − | 102847420 | 102847427 | 1.9e-05 | 0.243 | `CCGCCTCC` |
| CCGSCTCC | chr12 | − | 109424408 | 109424415 | 1.9e-05 | 0.243 | `CCGCCTCC` |
| CCGSCTCC | chr13 | − | 20648940 | 20648947 | 1.9e-05 | 0.243 | `CCGCCTCC` |
| CCGSCTCC | chr13 | + | 48005193 | 48005200 | 1.9e-05 | 0.243 | `CCGCCTCC` |
| CCGSCTCC | chr13 | − | 48916235 | 48916242 | 1.9e-05 | 0.243 | `CCGCCTCC` |
| CCGSCTCC | chr14 | + | 22095463 | 22095470 | 1.9e-05 | 0.243 | `CCGCCTCC` |
| CCGSCTCC | chr14 | − | 91046509 | 91046516 | 1.9e-05 | 0.243 | `CCGCCTCC` |
| CCGSCTCC | chr14 | − | 91046516 | 91046523 | 1.9e-05 | 0.243 | `CCGCCTCC` |
| CCGSCTCC | chr14 | − | 91046563 | 91046570 | 1.9e-05 | 0.243 | `CCGCCTCC` |
| CCGSCTCC | chr14 | + | 91046608 | 91046615 | 1.9e-05 | 0.243 | `CCGCCTCC` |
| CCGSCTCC | chr14 | − | 105309055 | 105309062 | 1.9e-05 | 0.243 | `CCGCCTCC` |
| CCGSCTCC | chr15 | + | 29295949 | 29295956 | 1.9e-05 | 0.243 | `CCGCCTCC` |
| CCGSCTCC | chr15 | − | 29296052 | 29296059 | 1.9e-05 | 0.243 | `CCGCCTCC` |
| CCGSCTCC | chr15 | − | 29309123 | 29309130 | 1.9e-05 | 0.243 | `CCGCCTCC` |
| CCGSCTCC | chr15 | − | 36533879 | 36533886 | 1.9e-05 | 0.243 | `CCGCCTCC` |
| CCGSCTCC | chr15 | − | 62466986 | 62466993 | 1.9e-05 | 0.243 | `CCGCCTCC` |
| CCGSCTCC | chr15 | + | 62974192 | 62974199 | 1.9e-05 | 0.243 | `CCGCCTCC` |
| CCGSCTCC | chr15 | + | 68094549 | 68094556 | 1.9e-05 | 0.243 | `CCGCCTCC` |
| CCGSCTCC | chr16 | + | 11743579 | 11743586 | 1.9e-05 | 0.243 | `CCGCCTCC` |
| CCGSCTCC | chr16 | − | 23560352 | 23560359 | 1.9e-05 | 0.243 | `CCGCCTCC` |
| CCGSCTCC | chr16 | − | 29734955 | 29734962 | 1.9e-05 | 0.243 | `CCGCCTCC` |
| CCGSCTCC | chr16 | − | 29915057 | 29915064 | 1.9e-05 | 0.243 | `CCGCCTCC` |
| CCGSCTCC | chr16 | − | 30490867 | 30490874 | 1.9e-05 | 0.243 | `CCGCCTCC` |
| CCGSCTCC | chr16 | − | 30569619 | 30569626 | 1.9e-05 | 0.243 | `CCGCCTCC` |
| CCGSCTCC | chr16 | − | 31098949 | 31098956 | 1.9e-05 | 0.243 | `CCGCCTCC` |
| CCGSCTCC | chr16 | − | 66464482 | 66464489 | 1.9e-05 | 0.243 | `CCGCCTCC` |
| CCGSCTCC | chr16 | + | 86542654 | 86542661 | 1.9e-05 | 0.243 | `CCGCCTCC` |
| CCGSCTCC | chr17 | − | 1920853 | 1920860 | 1.9e-05 | 0.243 | `CCGCCTCC` |
| CCGSCTCC | chr17 | + | 7328646 | 7328653 | 1.9e-05 | 0.243 | `CCGCCTCC` |
| CCGSCTCC | chr17 | − | 7328748 | 7328755 | 1.9e-05 | 0.243 | `CCGCCTCC` |
| CCGSCTCC | chr17 | + | 16130229 | 16130236 | 1.9e-05 | 0.243 | `CCGCCTCC` |
| CCGSCTCC | chr17 | + | 39936345 | 39936352 | 1.9e-05 | 0.243 | `CCGCCTCC` |
| CCGSCTCC | chr17 | + | 59172873 | 59172880 | 1.9e-05 | 0.243 | `CCGCCTCC` |
| CCGSCTCC | chr17 | + | 70803570 | 70803577 | 1.9e-05 | 0.243 | `CCGCCTCC` |
| CCGSCTCC | chr17 | + | 71861844 | 71861851 | 1.9e-05 | 0.243 | `CCGCCTCC` |
| CCGSCTCC | chr18 | − | 42008083 | 42008090 | 1.9e-05 | 0.243 | `CCGCCTCC` |
| CCGSCTCC | chr18 | + | 45272694 | 45272701 | 1.9e-05 | 0.243 | `CCGCCTCC` |
| CCGSCTCC | chr18 | + | 58974134 | 58974141 | 1.9e-05 | 0.243 | `CCGCCTCC` |
| CCGSCTCC | chr19 | + | 748243 | 748250 | 1.9e-05 | 0.243 | `CCGCCTCC` |
| CCGSCTCC | chr19 | − | 1602667 | 1602674 | 1.9e-05 | 0.243 | `CCGCCTCC` |
| CCGSCTCC | chr19 | − | 2691345 | 2691352 | 1.9e-05 | 0.243 | `CCGCCTCC` |
| CCGSCTCC | chr19 | − | 2734372 | 2734379 | 1.9e-05 | 0.243 | `CCGCCTCC` |
| CCGSCTCC | chr19 | − | 10258607 | 10258614 | 1.9e-05 | 0.243 | `CCGCCTCC` |
| CCGSCTCC | chr19 | + | 12266747 | 12266754 | 1.9e-05 | 0.243 | `CCGCCTCC` |
| CCGSCTCC | chr19 | + | 13126892 | 13126899 | 1.9e-05 | 0.243 | `CCGCCTCC` |
| CCGSCTCC | chr19 | + | 17391757 | 17391764 | 1.9e-05 | 0.243 | `CCGCCTCC` |
| CCGSCTCC | chr19 | − | 19127486 | 19127493 | 1.9e-05 | 0.243 | `CCGCCTCC` |
| CCGSCTCC | chr19 | − | 19292254 | 19292261 | 1.9e-05 | 0.243 | `CCGCCTCC` |
| CCGSCTCC | chr19 | + | 19292628 | 19292635 | 1.9e-05 | 0.243 | `CCGCCTCC` |
| CCGSCTCC | chr19 | + | 40900105 | 40900112 | 1.9e-05 | 0.243 | `CCGCCTCC` |
| CCGSCTCC | chr19 | + | 43228735 | 43228742 | 1.9e-05 | 0.243 | `CCGCCTCC` |
| CCGSCTCC | chr19 | + | 44627783 | 44627790 | 1.9e-05 | 0.243 | `CCGCCTCC` |
| CCGSCTCC | chr19 | − | 48951023 | 48951030 | 1.9e-05 | 0.243 | `CCGCCTCC` |
| CCGSCTCC | chr19 | + | 51058141 | 51058148 | 1.9e-05 | 0.243 | `CCGCCTCC` |
| CCGSCTCC | chr19 | + | 51058352 | 51058359 | 1.9e-05 | 0.243 | `CCGCCTCC` |
| CCGSCTCC | chr19 | − | 53814617 | 53814624 | 1.9e-05 | 0.243 | `CCGCCTCC` |
| CCGSCTCC | chr19 | + | 55071658 | 55071665 | 1.9e-05 | 0.243 | `CCGCCTCC` |
| CCGSCTCC | chr19 | + | 55071758 | 55071765 | 1.9e-05 | 0.243 | `CCGCCTCC` |
| CCGSCTCC | chr19 | + | 55071778 | 55071785 | 1.9e-05 | 0.243 | `CCGCCTCC` |
| CCGSCTCC | chr19 | + | 60462624 | 60462631 | 1.9e-05 | 0.243 | `CCGCCTCC` |
| CCGSCTCC | chr19 | + | 62483346 | 62483353 | 1.9e-05 | 0.243 | `CCGCCTCC` |
| CCGSCTCC | chr19 | − | 62483608 | 62483615 | 1.9e-05 | 0.243 | `CCGCCTCC` |
| CCGSCTCC | chr20 | − | 44033906 | 44033913 | 1.9e-05 | 0.243 | `CCGCCTCC` |
| CCGSCTCC | chr20 | + | 61730228 | 61730235 | 1.9e-05 | 0.243 | `CCGCCTCC` |
| CCGSCTCC | chr21 | − | 44388067 | 44388074 | 1.9e-05 | 0.243 | `CCGCCTCC` |
| CCGSCTCC | chr22 | + | 18484953 | 18484960 | 1.9e-05 | 0.243 | `CCGCCTCC` |
| CCGSCTCC | chr22 | − | 36334476 | 36334483 | 1.9e-05 | 0.243 | `CCGCCTCC` |
| CCGSCTCC | chr22 | + | 38173053 | 38173060 | 1.9e-05 | 0.243 | `CCGCCTCC` |
| CCGSCTCC | chr22 | − | 40558694 | 40558701 | 1.9e-05 | 0.243 | `CCGCCTCC` |
| CCGSCTCC | chr22 | + | 49310997 | 49311004 | 1.9e-05 | 0.243 | `CCGCCTCC` |
| CCGSCTCC | chr22 | − | 49311506 | 49311513 | 1.9e-05 | 0.243 | `CCGCCTCC` |
| CCGSCTCC | chr22 | + | 49311837 | 49311844 | 1.9e-05 | 0.243 | `CCGCCTCC` |
| CCGSCTCC | chr22 | + | 49316175 | 49316182 | 1.9e-05 | 0.243 | `CCGCCTCC` |
| CCGSCTCC | chr1 | − | 36327266 | 36327273 | 3.8e-05 | 0.301 | `CCGGCTCC` |
| CCGSCTCC | chr1 | − | 84745044 | 84745051 | 3.8e-05 | 0.301 | `CCGGCTCC` |
| CCGSCTCC | chr1 | + | 101474114 | 101474121 | 3.8e-05 | 0.301 | `CCGGCTCC` |
| CCGSCTCC | chr1 | − | 110378785 | 110378792 | 3.8e-05 | 0.301 | `CCGGCTCC` |
| CCGSCTCC | chr1 | + | 111548387 | 111548394 | 3.8e-05 | 0.301 | `CCGGCTCC` |
| CCGSCTCC | chr1 | − | 111937238 | 111937245 | 3.8e-05 | 0.301 | `CCGGCTCC` |
| CCGSCTCC | chr1 | + | 153201188 | 153201195 | 3.8e-05 | 0.301 | `CCGGCTCC` |
| CCGSCTCC | chr1 | + | 159635945 | 159635952 | 3.8e-05 | 0.301 | `CCGGCTCC` |
| CCGSCTCC | chr1 | + | 181259949 | 181259956 | 3.8e-05 | 0.301 | `CCGGCTCC` |
| CCGSCTCC | chr1 | − | 228845070 | 228845077 | 3.8e-05 | 0.301 | `CCGGCTCC` |
| CCGSCTCC | chr2 | − | 3600576 | 3600583 | 3.8e-05 | 0.301 | `CCGGCTCC` |
| CCGSCTCC | chr2 | − | 27433452 | 27433459 | 3.8e-05 | 0.301 | `CCGGCTCC` |
| CCGSCTCC | chr2 | + | 33556142 | 33556149 | 3.8e-05 | 0.301 | `CCGGCTCC` |
| CCGSCTCC | chr2 | − | 88772286 | 88772293 | 3.8e-05 | 0.301 | `CCGGCTCC` |
| CCGSCTCC | chr2 | + | 98448728 | 98448735 | 3.8e-05 | 0.301 | `CCGGCTCC` |
| CCGSCTCC | chr2 | − | 111642768 | 111642775 | 3.8e-05 | 0.301 | `CCGGCTCC` |
| CCGSCTCC | chr2 | + | 201689734 | 201689741 | 3.8e-05 | 0.301 | `CCGGCTCC` |
| CCGSCTCC | chr2 | + | 203811840 | 203811847 | 3.8e-05 | 0.301 | `CCGGCTCC` |
| CCGSCTCC | chr2 | − | 231549823 | 231549830 | 3.8e-05 | 0.301 | `CCGGCTCC` |
| CCGSCTCC | chr2 | + | 232235197 | 232235204 | 3.8e-05 | 0.301 | `CCGGCTCC` |
| CCGSCTCC | chr3 | + | 50372010 | 50372017 | 3.8e-05 | 0.301 | `CCGGCTCC` |
| CCGSCTCC | chr3 | + | 120781274 | 120781281 | 3.8e-05 | 0.301 | `CCGGCTCC` |
| CCGSCTCC | chr3 | + | 143427129 | 143427136 | 3.8e-05 | 0.301 | `CCGGCTCC` |
| CCGSCTCC | chr3 | + | 186699281 | 186699288 | 3.8e-05 | 0.301 | `CCGGCTCC` |
| CCGSCTCC | chr4 | − | 482894 | 482901 | 3.8e-05 | 0.301 | `CCGGCTCC` |
| CCGSCTCC | chr4 | − | 483050 | 483057 | 3.8e-05 | 0.301 | `CCGGCTCC` |
| CCGSCTCC | chr4 | − | 41864508 | 41864515 | 3.8e-05 | 0.301 | `CCGGCTCC` |
| CCGSCTCC | chr5 | − | 81083097 | 81083104 | 3.8e-05 | 0.301 | `CCGGCTCC` |
| CCGSCTCC | chr5 | − | 90714465 | 90714472 | 3.8e-05 | 0.301 | `CCGGCTCC` |
| CCGSCTCC | chr5 | + | 138748401 | 138748408 | 3.8e-05 | 0.301 | `CCGGCTCC` |
| CCGSCTCC | chr5 | + | 145542394 | 145542401 | 3.8e-05 | 0.301 | `CCGGCTCC` |
| CCGSCTCC | chr6 | − | 174274 | 174281 | 3.8e-05 | 0.301 | `CCGGCTCC` |
| CCGSCTCC | chr6 | − | 26152322 | 26152329 | 3.8e-05 | 0.301 | `CCGGCTCC` |
| CCGSCTCC | chr6 | − | 27223019 | 27223026 | 3.8e-05 | 0.301 | `CCGGCTCC` |
| CCGSCTCC | chr6 | − | 32265894 | 32265901 | 3.8e-05 | 0.301 | `CCGGCTCC` |
| CCGSCTCC | chr6 | − | 33276687 | 33276694 | 3.8e-05 | 0.301 | `CCGGCTCC` |
| CCGSCTCC | chr6 | + | 36756023 | 36756030 | 3.8e-05 | 0.301 | `CCGGCTCC` |
| CCGSCTCC | chr6 | − | 100123364 | 100123371 | 3.8e-05 | 0.301 | `CCGGCTCC` |
| CCGSCTCC | chr7 | + | 12217488 | 12217495 | 3.8e-05 | 0.301 | `CCGGCTCC` |
| CCGSCTCC | chr7 | + | 73281461 | 73281468 | 3.8e-05 | 0.301 | `CCGGCTCC` |
| CCGSCTCC | chr7 | + | 134506117 | 134506124 | 3.8e-05 | 0.301 | `CCGGCTCC` |
| CCGSCTCC | chr7 | − | 148952795 | 148952802 | 3.8e-05 | 0.301 | `CCGGCTCC` |
| CCGSCTCC | chr8 | + | 67026744 | 67026751 | 3.8e-05 | 0.301 | `CCGGCTCC` |
| CCGSCTCC | chr9 | + | 67903481 | 67903488 | 3.8e-05 | 0.301 | `CCGGCTCC` |
| CCGSCTCC | chr9 | − | 122703810 | 122703817 | 3.8e-05 | 0.301 | `CCGGCTCC` |
| CCGSCTCC | chr9 | − | 125141600 | 125141607 | 3.8e-05 | 0.301 | `CCGGCTCC` |
| CCGSCTCC | chr9 | + | 130684553 | 130684560 | 3.8e-05 | 0.301 | `CCGGCTCC` |
| CCGSCTCC | chr9 | − | 131688068 | 131688075 | 3.8e-05 | 0.301 | `CCGGCTCC` |
| CCGSCTCC | chr9 | − | 131688099 | 131688106 | 3.8e-05 | 0.301 | `CCGGCTCC` |
| CCGSCTCC | chrX | + | 53727855 | 53727862 | 3.8e-05 | 0.301 | `CCGGCTCC` |
| CCGSCTCC | chrX | + | 54087665 | 54087672 | 3.8e-05 | 0.301 | `CCGGCTCC` |
| CCGSCTCC | chr10 | − | 3817806 | 3817813 | 3.8e-05 | 0.301 | `CCGGCTCC` |
| CCGSCTCC | chr10 | + | 35456010 | 35456017 | 3.8e-05 | 0.301 | `CCGGCTCC` |
| CCGSCTCC | chr10 | + | 63326733 | 63326740 | 3.8e-05 | 0.301 | `CCGGCTCC` |
| CCGSCTCC | chr11 | + | 440169 | 440176 | 3.8e-05 | 0.301 | `CCGGCTCC` |
| CCGSCTCC | chr11 | + | 9438759 | 9438766 | 3.8e-05 | 0.301 | `CCGGCTCC` |
| CCGSCTCC | chr11 | + | 13255645 | 13255652 | 3.8e-05 | 0.301 | `CCGGCTCC` |
| CCGSCTCC | chr11 | + | 13255751 | 13255758 | 3.8e-05 | 0.301 | `CCGGCTCC` |
| CCGSCTCC | chr11 | + | 13441377 | 13441384 | 3.8e-05 | 0.301 | `CCGGCTCC` |
| CCGSCTCC | chr11 | + | 47557103 | 47557110 | 3.8e-05 | 0.301 | `CCGGCTCC` |
| CCGSCTCC | chr11 | + | 62146063 | 62146070 | 3.8e-05 | 0.301 | `CCGGCTCC` |
| CCGSCTCC | chr11 | + | 64641692 | 64641699 | 3.8e-05 | 0.301 | `CCGGCTCC` |
| CCGSCTCC | chr11 | − | 65443491 | 65443498 | 3.8e-05 | 0.301 | `CCGGCTCC` |
| CCGSCTCC | chr11 | + | 65443624 | 65443631 | 3.8e-05 | 0.301 | `CCGGCTCC` |
| CCGSCTCC | chr11 | + | 118301951 | 118301958 | 3.8e-05 | 0.301 | `CCGGCTCC` |
| CCGSCTCC | chr11 | + | 122438191 | 122438198 | 3.8e-05 | 0.301 | `CCGGCTCC` |
| CCGSCTCC | chr12 | − | 55014541 | 55014548 | 3.8e-05 | 0.301 | `CCGGCTCC` |
| CCGSCTCC | chr12 | − | 120811109 | 120811116 | 3.8e-05 | 0.301 | `CCGGCTCC` |
| CCGSCTCC | chr13 | + | 23742937 | 23742944 | 3.8e-05 | 0.301 | `CCGGCTCC` |
| CCGSCTCC | chr14 | + | 104583137 | 104583144 | 3.8e-05 | 0.301 | `CCGGCTCC` |
| CCGSCTCC | chr15 | + | 29343772 | 29343779 | 3.8e-05 | 0.301 | `CCGGCTCC` |
| CCGSCTCC | chr15 | − | 36533666 | 36533673 | 3.8e-05 | 0.301 | `CCGGCTCC` |
| CCGSCTCC | chr15 | + | 70310666 | 70310673 | 3.8e-05 | 0.301 | `CCGGCTCC` |
| CCGSCTCC | chr16 | + | 1981494 | 1981501 | 3.8e-05 | 0.301 | `CCGGCTCC` |
| CCGSCTCC | chr16 | + | 2195464 | 2195471 | 3.8e-05 | 0.301 | `CCGGCTCC` |
| CCGSCTCC | chr16 | − | 11678899 | 11678906 | 3.8e-05 | 0.301 | `CCGGCTCC` |
| CCGSCTCC | chr16 | − | 28765112 | 28765119 | 3.8e-05 | 0.301 | `CCGGCTCC` |
| CCGSCTCC | chr16 | + | 30377959 | 30377966 | 3.8e-05 | 0.301 | `CCGGCTCC` |
| CCGSCTCC | chr16 | − | 30569579 | 30569586 | 3.8e-05 | 0.301 | `CCGGCTCC` |
| CCGSCTCC | chr16 | − | 30841623 | 30841630 | 3.8e-05 | 0.301 | `CCGGCTCC` |
| CCGSCTCC | chr17 | + | 8139620 | 8139627 | 3.8e-05 | 0.301 | `CCGGCTCC` |
| CCGSCTCC | chr17 | − | 20542892 | 20542899 | 3.8e-05 | 0.301 | `CCGGCTCC` |
| CCGSCTCC | chr17 | + | 27693351 | 27693358 | 3.8e-05 | 0.301 | `CCGGCTCC` |
| CCGSCTCC | chr17 | − | 35274062 | 35274069 | 3.8e-05 | 0.301 | `CCGGCTCC` |
| CCGSCTCC | chr18 | − | 42007831 | 42007838 | 3.8e-05 | 0.301 | `CCGGCTCC` |
| CCGSCTCC | chr18 | + | 45272644 | 45272651 | 3.8e-05 | 0.301 | `CCGGCTCC` |
| CCGSCTCC | chr18 | + | 59136191 | 59136198 | 3.8e-05 | 0.301 | `CCGGCTCC` |
| CCGSCTCC | chr19 | + | 1389466 | 1389473 | 3.8e-05 | 0.301 | `CCGGCTCC` |
| CCGSCTCC | chr19 | − | 5641437 | 5641444 | 3.8e-05 | 0.301 | `CCGGCTCC` |
| CCGSCTCC | chr19 | + | 6752693 | 6752700 | 3.8e-05 | 0.301 | `CCGGCTCC` |
| CCGSCTCC | chr19 | − | 10084972 | 10084979 | 3.8e-05 | 0.301 | `CCGGCTCC` |
| CCGSCTCC | chr19 | + | 10091422 | 10091429 | 3.8e-05 | 0.301 | `CCGGCTCC` |
| CCGSCTCC | chr19 | − | 16050685 | 16050692 | 3.8e-05 | 0.301 | `CCGGCTCC` |
| CCGSCTCC | chr19 | + | 16339346 | 16339353 | 3.8e-05 | 0.301 | `CCGGCTCC` |
| CCGSCTCC | chr19 | − | 17187178 | 17187185 | 3.8e-05 | 0.301 | `CCGGCTCC` |
| CCGSCTCC | chr19 | − | 47080239 | 47080246 | 3.8e-05 | 0.301 | `CCGGCTCC` |
| CCGSCTCC | chr19 | − | 48951117 | 48951124 | 3.8e-05 | 0.301 | `CCGGCTCC` |
| CCGSCTCC | chr19 | + | 60462422 | 60462429 | 3.8e-05 | 0.301 | `CCGGCTCC` |
| CCGSCTCC | chr20 | − | 45846916 | 45846923 | 3.8e-05 | 0.301 | `CCGGCTCC` |
| CCGSCTCC | chr20 | − | 45847350 | 45847357 | 3.8e-05 | 0.301 | `CCGGCTCC` |
| CCGSCTCC | chr20 | + | 48560214 | 48560221 | 3.8e-05 | 0.301 | `CCGGCTCC` |
| CCGSCTCC | chr20 | − | 56659761 | 56659768 | 3.8e-05 | 0.301 | `CCGGCTCC` |
| CCGSCTCC | chr21 | − | 25901835 | 25901842 | 3.8e-05 | 0.301 | `CCGGCTCC` |
| CCGSCTCC | chr22 | + | 40139732 | 40139739 | 3.8e-05 | 0.301 | `CCGGCTCC` |
| CCGSCTCC | chr22 | + | 40172949 | 40172956 | 3.8e-05 | 0.301 | `CCGGCTCC` |
| CCGSCTCC | chr1 | + | 12034838 | 12034845 | 7.33e-05 | 0.449 | `CCGTCTCC` |
| CCGSCTCC | chr1 | − | 27521521 | 27521528 | 7.33e-05 | 0.449 | `CCGACTCC` |
| CCGSCTCC | chr1 | + | 110752134 | 110752141 | 7.33e-05 | 0.449 | `CCGACTCC` |
| CCGSCTCC | chr1 | + | 114249339 | 114249346 | 7.33e-05 | 0.449 | `CCGACTCC` |
| CCGSCTCC | chr1 | − | 148806982 | 148806989 | 7.33e-05 | 0.449 | `CCGTCTCC` |
| CCGSCTCC | chr1 | + | 148818675 | 148818682 | 7.33e-05 | 0.449 | `CCGACTCC` |
| CCGSCTCC | chr1 | + | 152185174 | 152185181 | 7.33e-05 | 0.449 | `CCGACTCC` |
| CCGSCTCC | chr1 | + | 154212837 | 154212844 | 7.33e-05 | 0.449 | `CCGACTCC` |
| CCGSCTCC | chr1 | + | 154453015 | 154453022 | 7.33e-05 | 0.449 | `CCGACTCC` |
| CCGSCTCC | chr1 | + | 178118684 | 178118691 | 7.33e-05 | 0.449 | `CCGTCTCC` |
| CCGSCTCC | chr1 | + | 228325938 | 228325945 | 7.33e-05 | 0.449 | `CCGACTCC` |
| CCGSCTCC | chr2 | − | 27433249 | 27433256 | 7.33e-05 | 0.449 | `CCGACTCC` |
| CCGSCTCC | chr2 | − | 42182945 | 42182952 | 7.33e-05 | 0.449 | `CCGTCTCC` |
| CCGSCTCC | chr2 | − | 233654306 | 233654313 | 7.33e-05 | 0.449 | `CCGACTCC` |
| CCGSCTCC | chr3 | + | 9413560 | 9413567 | 7.33e-05 | 0.449 | `CCGACTCC` |
| CCGSCTCC | chr5 | + | 96296820 | 96296827 | 7.33e-05 | 0.449 | `CCGTCTCC` |
| CCGSCTCC | chr6 | − | 237715 | 237722 | 7.33e-05 | 0.449 | `CCGACTCC` |
| CCGSCTCC | chr6 | − | 7838774 | 7838781 | 7.33e-05 | 0.449 | `CCGACTCC` |
| CCGSCTCC | chr6 | − | 7838793 | 7838800 | 7.33e-05 | 0.449 | `CCGACTCC` |
| CCGSCTCC | chr6 | + | 26303919 | 26303926 | 7.33e-05 | 0.449 | `CCGTCTCC` |
| CCGSCTCC | chr6 | − | 30818923 | 30818930 | 7.33e-05 | 0.449 | `CCGACTCC` |
| CCGSCTCC | chr6 | − | 33047635 | 33047642 | 7.33e-05 | 0.449 | `CCGACTCC` |
| CCGSCTCC | chr6 | + | 33486197 | 33486204 | 7.33e-05 | 0.449 | `CCGACTCC` |
| CCGSCTCC | chr6 | − | 36103616 | 36103623 | 7.33e-05 | 0.449 | `CCGACTCC` |
| CCGSCTCC | chr7 | − | 2984758 | 2984765 | 7.33e-05 | 0.449 | `CCGTCTCC` |
| CCGSCTCC | chr7 | + | 3963029 | 3963036 | 7.33e-05 | 0.449 | `CCGTCTCC` |
| CCGSCTCC | chr7 | + | 44071187 | 44071194 | 7.33e-05 | 0.449 | `CCGACTCC` |
| CCGSCTCC | chr7 | + | 44802480 | 44802487 | 7.33e-05 | 0.449 | `CCGTCTCC` |
| CCGSCTCC | chr7 | + | 75515240 | 75515247 | 7.33e-05 | 0.449 | `CCGACTCC` |
| CCGSCTCC | chr7 | − | 100019669 | 100019676 | 7.33e-05 | 0.449 | `CCGTCTCC` |
| CCGSCTCC | chr7 | − | 149733309 | 149733316 | 7.33e-05 | 0.449 | `CCGTCTCC` |
| CCGSCTCC | chr8 | + | 28803665 | 28803672 | 7.33e-05 | 0.449 | `CCGACTCC` |
| CCGSCTCC | chr8 | − | 95635167 | 95635174 | 7.33e-05 | 0.449 | `CCGTCTCC` |
| CCGSCTCC | chr8 | − | 95635181 | 95635188 | 7.33e-05 | 0.449 | `CCGTCTCC` |
| CCGSCTCC | chr8 | − | 135681827 | 135681834 | 7.33e-05 | 0.449 | `CCGTCTCC` |
| CCGSCTCC | chr9 | + | 6671433 | 6671440 | 7.33e-05 | 0.449 | `CCGACTCC` |
| CCGSCTCC | chr9 | − | 97677781 | 97677788 | 7.33e-05 | 0.449 | `CCGTCTCC` |
| CCGSCTCC | chr9 | − | 99214280 | 99214287 | 7.33e-05 | 0.449 | `CCGTCTCC` |
| CCGSCTCC | chr9 | − | 130458827 | 130458834 | 7.33e-05 | 0.449 | `CCGTCTCC` |
| CCGSCTCC | chr9 | + | 131688323 | 131688330 | 7.33e-05 | 0.449 | `CCGTCTCC` |
| CCGSCTCC | chr9 | − | 135193003 | 135193010 | 7.33e-05 | 0.449 | `CCGACTCC` |
| CCGSCTCC | chr10 | − | 69761868 | 69761875 | 7.33e-05 | 0.449 | `CCGTCTCC` |
| CCGSCTCC | chr10 | − | 73404319 | 73404326 | 7.33e-05 | 0.449 | `CCGTCTCC` |
| CCGSCTCC | chr10 | + | 126375200 | 126375207 | 7.33e-05 | 0.449 | `CCGACTCC` |
| CCGSCTCC | chr11 | − | 64402957 | 64402964 | 7.33e-05 | 0.449 | `CCGTCTCC` |
| CCGSCTCC | chr11 | + | 117387146 | 117387153 | 7.33e-05 | 0.449 | `CCGTCTCC` |
| CCGSCTCC | chr12 | − | 6431749 | 6431756 | 7.33e-05 | 0.449 | `CCGACTCC` |
| CCGSCTCC | chr12 | − | 54798415 | 54798422 | 7.33e-05 | 0.449 | `CCGACTCC` |
| CCGSCTCC | chr12 | − | 81276676 | 81276683 | 7.33e-05 | 0.449 | `CCGTCTCC` |
| CCGSCTCC | chr12 | + | 91063826 | 91063833 | 7.33e-05 | 0.449 | `CCGACTCC` |
| CCGSCTCC | chr12 | + | 132123863 | 132123870 | 7.33e-05 | 0.449 | `CCGTCTCC` |
| CCGSCTCC | chr13 | + | 33014922 | 33014929 | 7.33e-05 | 0.449 | `CCGTCTCC` |
| CCGSCTCC | chr13 | − | 47509920 | 47509927 | 7.33e-05 | 0.449 | `CCGACTCC` |
| CCGSCTCC | chr13 | − | 47509926 | 47509933 | 7.33e-05 | 0.449 | `CCGACTCC` |
| CCGSCTCC | chr13 | + | 48916359 | 48916366 | 7.33e-05 | 0.449 | `CCGACTCC` |
| CCGSCTCC | chr14 | − | 64693423 | 64693430 | 7.33e-05 | 0.449 | `CCGTCTCC` |
| CCGSCTCC | chr14 | − | 91409829 | 91409836 | 7.33e-05 | 0.449 | `CCGACTCC` |
| CCGSCTCC | chr15 | − | 61583804 | 61583811 | 7.33e-05 | 0.449 | `CCGTCTCC` |
| CCGSCTCC | chr15 | + | 62233116 | 62233123 | 7.33e-05 | 0.449 | `CCGTCTCC` |
| CCGSCTCC | chr16 | + | 14287048 | 14287055 | 7.33e-05 | 0.449 | `CCGACTCC` |
| CCGSCTCC | chr16 | + | 49705256 | 49705263 | 7.33e-05 | 0.449 | `CCGTCTCC` |
| CCGSCTCC | chr17 | − | 7416824 | 7416831 | 7.33e-05 | 0.449 | `CCGACTCC` |
| CCGSCTCC | chr17 | + | 8139614 | 8139621 | 7.33e-05 | 0.449 | `CCGACTCC` |
| CCGSCTCC | chr17 | − | 16225054 | 16225061 | 7.33e-05 | 0.449 | `CCGTCTCC` |
| CCGSCTCC | chr17 | + | 53784480 | 53784487 | 7.33e-05 | 0.449 | `CCGTCTCC` |
| CCGSCTCC | chr17 | − | 77779531 | 77779538 | 7.33e-05 | 0.449 | `CCGACTCC` |
| CCGSCTCC | chr18 | + | 3237953 | 3237960 | 7.33e-05 | 0.449 | `CCGTCTCC` |
| CCGSCTCC | chr19 | + | 747990 | 747997 | 7.33e-05 | 0.449 | `CCGACTCC` |
| CCGSCTCC | chr19 | + | 5641127 | 5641134 | 7.33e-05 | 0.449 | `CCGACTCC` |
| CCGSCTCC | chr19 | − | 9799686 | 9799693 | 7.33e-05 | 0.449 | `CCGACTCC` |
| CCGSCTCC | chr19 | + | 17187347 | 17187354 | 7.33e-05 | 0.449 | `CCGACTCC` |
| CCGSCTCC | chr19 | + | 17187390 | 17187397 | 7.33e-05 | 0.449 | `CCGACTCC` |
| CCGSCTCC | chr19 | − | 44586366 | 44586373 | 7.33e-05 | 0.449 | `CCGTCTCC` |
| CCGSCTCC | chr19 | + | 47055632 | 47055639 | 7.33e-05 | 0.449 | `CCGTCTCC` |
| CCGSCTCC | chr19 | − | 47079915 | 47079922 | 7.33e-05 | 0.449 | `CCGTCTCC` |
| CCGSCTCC | chr19 | + | 51914467 | 51914474 | 7.33e-05 | 0.449 | `CCGTCTCC` |
| CCGSCTCC | chr19 | − | 54695507 | 54695514 | 7.33e-05 | 0.449 | `CCGTCTCC` |
| CCGSCTCC | chr19 | + | 63722923 | 63722930 | 7.33e-05 | 0.449 | `CCGACTCC` |
| CCGSCTCC | chr21 | + | 35160141 | 35160148 | 7.33e-05 | 0.449 | `CCGACTCC` |
| CCGSCTCC | chr22 | − | 38246419 | 38246426 | 7.33e-05 | 0.449 | `CCGTCTCC` |

---

**DEBUGGING INFORMATION**


---

Command line:

```
/ebi/sw/MEME/VM-cluster410/meme-versions/4.10.0/bin/fimo --parse-genomic-coord --verbosity 1 --oc fimo_out_15 --bgfile ./background --motif CCGSCTCC dreme_out/dreme.xml ./Supplementary_Table_1.500bp.fa
```

Settings:

```
|  |  |  |
| --- | --- | --- |
| output directory = fimo_out_15 | MEME file name = dreme_out/dreme.xml | sequence file name = ./Supplementary_Table_1.500bp.fa |
| background file name = ./background | allow clobber = true | compute q-values = true |
| parse genomic coord. = true | text only = false | scan both strands = true |
| max sequence length = 250000000 | output threshold = 0.0001 | threshold type = p-value |
| max stored scores = 100000 | pseudocount = 0.1 | verbosity = 1 |
| selected motif = CCGSCTCC |  |  |
```

This information can be useful in the event you wish to report a
problem with the FIMO software.

---

**Go to top**
