# Supplementary material for: The MEME Suite
Source: Nucleic Acids Res. 2015 May 7;43(Web Server issue):W39–49. doi: 10.1093/nar/gkv416 (PMC4489269; doi:10.1093/nar/gkv416)
Supplement: SUPPLEMENTARY DATA [file supp_gkv416_nar-00283-web-b-2015-File005.zip › case4/meme-chip/fimo_out_16/fimo.html]

FIMO Results


---

|  |  |  |
| --- | --- | --- |
| **Database and Motifs** | **High-scoring Motif Occurrences** | **Debugging Information** |

  
  

---

**FIMO - Motif search tool**


---

FIMO version 4.10.0,
(Release date: Wed May 21 10:35:36 2014 +1000)

For further information on how to interpret these results
or to get a copy of the FIMO software please access
http://meme.nbcr.net

If you use FIMO in your research, please cite the following paper:  
Charles E. Grant, Timothy L. Bailey, and William Stafford Noble,
"FIMO: Scanning for occurrences of a given motif",
*Bioinformatics*, **27**(7):1017-1018, 2011.
[full text]

---

**DATABASE AND MOTIFS**


---

DATABASE
./Supplementary\_Table\_1.500bp.fa  
Database contains
2776
sequences,
1388000
residues

MOTIFS
db/JASPAR\_CORE\_2014\_vertebrates.meme
(nucleotide)

| MOTIF | WIDTH | BEST POSSIBLE MATCH |
| --- | --- | --- |
| MA0004.1 | 6 | CACGTG |
| MA0006.1 | 6 | TGCGTG |
| MA0009.1 | 11 | CTAGGTGTGAA |
| MA0017.1 | 14 | TGACCTTTGAACCT |
| MA0019.1 | 12 | AGATGCAATCCC |
| MA0025.1 | 11 | TTATGTAACGT |
| MA0027.1 | 11 | AAGTAGTGGCC |
| MA0028.1 | 10 | GAGCCGGAAG |
| MA0029.1 | 14 | AAGATAAGATAAGA |
| MA0030.1 | 14 | CAAACGTAAACAAT |
| MA0031.1 | 8 | GTAAACAT |
| MA0032.1 | 8 | GGTAAGTA |
| MA0033.1 | 8 | TATACATA |
| MA0038.1 | 10 | CAAATCACTG |
| MA0040.1 | 11 | TATTGTTTATT |
| MA0041.1 | 12 | GAATGTTTGTTT |
| MA0042.1 | 12 | GGATGTTTGTTT |
| MA0043.1 | 12 | GGTTACGCAATC |
| MA0046.1 | 14 | GGTTAATAATTACC |
| MA0048.1 | 12 | GCGCAGCTGCGT |
| MA0051.1 | 18 | GGAAAGCGAAAGCAAAAC |
| MA0056.1 | 6 | TGGGGA |
| MA0057.1 | 10 | GGAGGGGGAA |
| MA0059.1 | 11 | GAGCACGTGGT |
| MA0063.1 | 7 | TTAATTG |
| MA0066.1 | 20 | GTAGGTCACGGTGACCTACT |
| MA0067.1 | 8 | AGTCACGG |
| MA0068.1 | 30 | GAAAAATTTCCCATACTCCACTCCCCCCCC |
| MA0069.1 | 14 | TTCACGCATGAGTT |
| MA0070.1 | 12 | CCATCAATCAAA |
| MA0071.1 | 10 | ATCAAGGTCA |
| MA0072.1 | 14 | TATAAGTAGGTCAA |
| MA0073.1 | 20 | CCCCAAACCACCCCCCCCCC |
| MA0074.1 | 15 | GGGTCATCGGGTTCA |
| MA0075.1 | 5 | AATTA |
| MA0077.1 | 9 | CCATTGTTC |
| MA0078.1 | 9 | CTCATTGTC |
| MA0081.1 | 7 | AGAGGAA |
| MA0084.1 | 9 | GTAAACAAT |
| MA0087.1 | 7 | ATTGTTT |
| MA0088.1 | 20 | GATTTCCCATCATGCCTTGC |
| MA0089.1 | 6 | CATGAC |
| MA0090.1 | 12 | CACATTCCTCCG |
| MA0091.1 | 12 | CGACCATCTGTT |
| MA0092.1 | 10 | GGTCTGGCAT |
| MA0101.1 | 10 | GGGGATTTCC |
| MA0107.1 | 10 | GGGAATTTCC |
| MA0108.2 | 15 | GTATAAAAGGCGGGG |
| MA0109.1 | 10 | AACCTTATAT |
| MA0111.1 | 11 | AGGGTAACAGC |
| MA0115.1 | 17 | AAAGGTCAAAGGTCAAC |
| MA0116.1 | 15 | GGCACCCAGGGGTGC |
| MA0117.1 | 8 | GCTGACGG |
| MA0119.1 | 14 | TGGCACCATGCCAA |
| MA0122.1 | 9 | TTAAGTGGA |
| MA0124.1 | 7 | ATACTTA |
| MA0125.1 | 8 | TAATTGGT |
| MA0130.1 | 6 | ATCCAC |
| MA0131.1 | 10 | TAACGTCCGC |
| MA0132.1 | 6 | CTAATT |
| MA0133.1 | 7 | ACAACAC |
| MA0135.1 | 13 | AAATTAATTAATC |
| MA0136.1 | 9 | TACTTCCTT |
| MA0139.1 | 19 | TGGCCACCAGGGGGCGCTA |
| MA0142.1 | 15 | CTTTGTTATGCAAAT |
| MA0149.1 | 18 | GGAAGGAAGGAAGGAAGG |
| MA0062.2 | 11 | CCGGAAGTGGC |
| MA0039.2 | 10 | TGGGTGGGGC |
| MA0138.2 | 21 | TTCAGCACCATGGACAGCGCC |
| MA0002.2 | 11 | GTCTGTGGTTT |
| MA0047.2 | 12 | TGTTTACTTAGG |
| MA0112.2 | 20 | GGCCCAGGTCACCCTGACCT |
| MA0065.2 | 15 | GTAGGGCAAAGGTCA |
| MA0151.1 | 6 | ATTAAA |
| MA0152.1 | 7 | TTTTCCA |
| MA0153.1 | 12 | TTAATATTTAAC |
| MA0155.1 | 12 | TGTCAGGGGGCG |
| MA0156.1 | 8 | CAGGAAAT |
| MA0157.1 | 8 | TGTAAACA |
| MA0158.1 | 8 | CACTAATT |
| MA0159.1 | 17 | AGGTCACGGAGAGGTCA |
| MA0160.1 | 8 | AAGGTCAC |
| MA0161.1 | 6 | TTGGCA |
| MA0163.1 | 14 | GGGGCCCAAGGGGG |
| MA0164.1 | 7 | CAAGCTT |
| MA0018.2 | 8 | TGACGTCA |
| MA0099.2 | 7 | TGACTCA |
| MA0259.1 | 8 | GGACGTGC |
| MA0442.1 | 6 | CTTTGT |
| MA0141.2 | 12 | AGGTCAAGGTCA |
| MA0145.2 | 14 | CCAGTTCAAACCAG |
| MA0146.2 | 14 | GGGGCCGAGGCCTG |
| MA0461.1 | 8 | CAGATGGC |
| MA0462.1 | 11 | GAAATGACTCA |
| MA0463.1 | 14 | TTTCCTAGAAAGCA |
| MA0464.1 | 11 | CTCACGTGCAC |
| MA0465.1 | 11 | AAGCCATAAAA |
| MA0466.1 | 11 | TATTGCACAAT |
| MA0467.1 | 11 | AAGAGGATTAG |
| MA0468.1 | 11 | TAATTTAATCA |
| MA0469.1 | 15 | CTCCCGCCCCCACTC |
| MA0470.1 | 11 | GGGCGGGAAGG |
| MA0471.1 | 11 | GGGCGGGAAGG |
| MA0472.1 | 15 | CCCCCGCCCACGCAC |
| MA0473.1 | 13 | GAACCAGGAAGTG |
| MA0474.1 | 11 | ACAGGAAGTGG |
| MA0475.1 | 11 | ACAGGAAGTGG |
| MA0476.1 | 11 | TGTGACTCATT |
| MA0477.1 | 11 | GGTGACTCATG |
| MA0478.1 | 11 | GGATGACTCAT |
| MA0479.1 | 11 | TCCAATCCACA |
| MA0480.1 | 11 | TCCTGTTTACA |
| MA0481.1 | 15 | CAAAAGTAAACAAAG |
| MA0482.1 | 11 | TCTTATCTCCC |
| MA0483.1 | 11 | AAATCACAGCA |
| MA0484.1 | 15 | AGAGTCCAAAGTCCA |
| MA0485.1 | 13 | GGCCATAAATCAC |
| MA0486.1 | 15 | CTTCTAGAAGGTTCT |
| MA0488.1 | 13 | AAGATGATGTCAT |
| MA0489.1 | 14 | AGGAGATGACTCAT |
| MA0490.1 | 11 | GGATGACTCAT |
| MA0491.1 | 11 | GGTGACTCATC |
| MA0492.1 | 15 | AAAGATGATGTCATC |
| MA0493.1 | 11 | GGCCACACCCA |
| MA0494.1 | 19 | TGACCTAAAGTAACCTCTG |
| MA0495.1 | 18 | GCTGAGTCAGCAATTTTT |
| MA0496.1 | 15 | CTGAGTCAGCAATTT |
| MA0497.1 | 15 | ATGCTAAAAATAGAA |
| MA0498.1 | 15 | AGCTGTCACTCACCT |
| MA0499.1 | 13 | TGCAGCTGTCCCT |
| MA0500.1 | 11 | GACAGCTGCAG |
| MA0501.1 | 15 | ATGACTCAGCAATTT |
| MA0502.1 | 15 | AAATGGACCAATCAG |
| MA0503.1 | 11 | AGCCACTCAAG |
| MA0504.1 | 15 | AGGGGTCAGAGGTCA |
| MA0505.1 | 15 | AAGTTCAAGGTCAGC |
| MA0506.1 | 11 | GCGCCTGCGCA |
| MA0507.1 | 13 | TTCATTTGCATAT |
| MA0508.1 | 15 | AGAAAGTGAAAGTGA |
| MA0509.1 | 14 | GTTGCCATGGCAAC |
| MA0510.1 | 15 | CTCCCTGGCAACAGC |
| MA0511.1 | 15 | GGGGTTTGTGGTTTG |
| MA0512.1 | 11 | CAAAGGTCAGA |
| MA0513.1 | 13 | CTGTCTGTCACCT |
| MA0514.1 | 10 | CCTTTGTTTT |
| MA0515.1 | 10 | CCATTGTTTT |
| MA0516.1 | 15 | GCCCCGCCCCCTCCC |
| MA0517.1 | 15 | TCAGTTTCATTTTCC |
| MA0518.1 | 14 | TTTCCAGGAAATGG |
| MA0519.1 | 11 | ATTTCCAAGAA |
| MA0520.1 | 15 | CATTTCCTGAGAAAT |
| MA0521.1 | 11 | AACAGCTGCAG |
| MA0522.1 | 11 | CACAGCTGCAG |
| MA0523.1 | 14 | AAAGATCAAAGGAA |
| MA0524.1 | 15 | CATGGCCCCAGGGCA |
| MA0525.1 | 20 | AGACATGCCCAGACATGCCC |
| MA0526.1 | 11 | GTCATGTGACC |
| MA0527.1 | 15 | CTCTCGCGAGATCTG |
| MA0528.1 | 21 | GGAGGAGGAGGGGGAGGAGGA |
| MA0007.2 | 15 | AAGAACAGAATGTTC |
| MA0102.3 | 11 | ATTGCACAATA |
| MA0024.2 | 11 | CGGGCGGGAGG |
| MA0154.2 | 11 | GTCCCCAGGGA |
| MA0162.2 | 14 | CCCCCGCCCCCGCC |
| MA0076.2 | 11 | CCACTTCCGGC |
| MA0258.2 | 15 | AGGTCACCCTGACCT |
| MA0098.2 | 15 | CCCACTTCCTGTCTC |
| MA0148.3 | 15 | TCCATGTTTACTTTG |
| MA0035.3 | 11 | TTCTTATCTGT |
| MA0036.2 | 14 | AGATTCTTATCTGT |
| MA0037.2 | 8 | AGATAAGA |
| MA0114.2 | 15 | CTGGACTTTGGACTC |
| MA0050.2 | 21 | TTTTACTTTCACTTTCACTTT |
| MA0058.2 | 10 | AAGCACATGG |
| MA0052.2 | 15 | AGCTAAAAATAGCAT |
| MA0100.2 | 10 | CCAACTGCCA |
| MA0147.2 | 10 | CCATGTGCTT |
| MA0104.3 | 8 | GCCACGTG |
| MA0150.2 | 15 | CAGCATGACTCAGCA |
| MA0105.3 | 11 | GGGAATTTCCC |
| MA0060.2 | 18 | AGAGTGCTGATTGGTCCA |
| MA0014.2 | 19 | GAGGGCAGCCAAGCGTGAC |
| MA0080.3 | 15 | AAAAAGAGGAAGTGA |
| MA0143.3 | 8 | CCTTTGTT |
| MA0079.3 | 11 | GCCCCGCCCCC |
| MA0083.2 | 18 | CATGCCCAAATAAGGCAA |
| MA0137.3 | 11 | TTTCCAGGAAA |
| MA0144.2 | 11 | CTTCTGGGAAA |
| MA0140.2 | 18 | CTTATCTGTGAGGAGCAG |
| MA0003.2 | 15 | CATTGCCTCAGGGCA |
| MA0106.2 | 15 | ACATGCCCAGACATG |
| MA0093.2 | 11 | GCCACGTGACC |
| MA0095.2 | 12 | CAAGATGGCGGC |
| MA0103.2 | 9 | CCTCACCTG |
| MA0591.1 | 15 | AGGATGACTCAGCAC |
| MA0592.1 | 11 | CCAAGGTCACA |
| MA0593.1 | 11 | AAGTAAACAAA |
| MA0594.1 | 11 | GCCATAAATCA |
| MA0595.1 | 10 | ATCACCCCAC |
| MA0596.1 | 10 | ATGGGGTGAT |
| MA0597.1 | 9 | CTGCCCGCA |
| MA0598.1 | 8 | CCTTCCTG |
| MA0599.1 | 10 | GCCCCGCCCC |
| MA0600.1 | 19 | GTTGCCATGGCAACCGCGG |
| MA0113.2 | 15 | AGAACAGAATGTTCT |

Random model letter frequencies
(from ./background):
  
A 0.241 C 0.259 G 0.259 T 0.241

---

**SECTION I: HIGH-SCORING MOTIF OCCURRENCES**


---

- There were
  524
  motif occurrences with a
  p-value less than
  0.0001.
- The p-value of a motif occurrence is defined as the
  probability of a random sequence of the same length as the motif
  matching that position of the sequence with as good or better a score.
- The score for the match of a position in a sequence to a motif
  is computed by summing the appropriate entries from each column of
  the position-dependent scoring matrix that represents the motif.
- The q-value of a motif occurrence is defined as the
  false discovery rate if the occurrence is accepted as significant.
- The table is sorted by increasing p-value.

| Motif | Sequence Name | Strand | Start | End | p-value | q-value | Matched Sequence |
| --- | --- | --- | --- | --- | --- | --- | --- |
| MA0478.1 | chr2 | − | 8359758 | 8359768 | 2.28e-07 | 0.144 | `GGATGACTCAT` |
| MA0478.1 | chr5 | − | 10685372 | 10685382 | 2.28e-07 | 0.144 | `GGATGACTCAT` |
| MA0478.1 | chr14 | − | 72013530 | 72013540 | 2.28e-07 | 0.144 | `GGATGACTCAT` |
| MA0478.1 | chr1 | + | 148852782 | 148852792 | 4.73e-07 | 0.144 | `GGATGACTCAG` |
| MA0478.1 | chr21 | − | 33373382 | 33373392 | 4.73e-07 | 0.144 | `GGATGACTCAG` |
| MA0478.1 | chr1 | − | 224917252 | 224917262 | 7.19e-07 | 0.144 | `GGGTGACTCAT` |
| MA0478.1 | chr2 | + | 204600295 | 204600305 | 7.19e-07 | 0.144 | `GGGTGACTCAT` |
| MA0478.1 | chr1 | − | 42045206 | 42045216 | 9.3e-07 | 0.144 | `TGATGACTCAT` |
| MA0478.1 | chr1 | − | 233214233 | 233214243 | 9.3e-07 | 0.144 | `TGATGACTCAT` |
| MA0478.1 | chr2 | + | 149133355 | 149133365 | 9.3e-07 | 0.144 | `TGATGACTCAT` |
| MA0478.1 | chr2 | + | 169060465 | 169060475 | 9.3e-07 | 0.144 | `TGATGACTCAT` |
| MA0478.1 | chr10 | − | 105221286 | 105221296 | 9.3e-07 | 0.144 | `TGATGACTCAT` |
| MA0478.1 | chr15 | − | 81298544 | 81298554 | 9.3e-07 | 0.144 | `TGATGACTCAT` |
| MA0478.1 | chr20 | + | 47338123 | 47338133 | 9.3e-07 | 0.144 | `TGATGACTCAT` |
| MA0478.1 | chr2 | + | 12115292 | 12115302 | 1.2e-06 | 0.144 | `GGGTGACTCAG` |
| MA0478.1 | chr4 | + | 2717835 | 2717845 | 1.2e-06 | 0.144 | `GGGTGACTCAG` |
| MA0478.1 | chr10 | − | 112164438 | 112164448 | 1.2e-06 | 0.144 | `GGGTGACTCAG` |
| MA0478.1 | chr16 | − | 11364712 | 11364722 | 1.2e-06 | 0.144 | `GGGTGACTCAG` |
| MA0478.1 | chr14 | − | 93495648 | 93495658 | 1.67e-06 | 0.173 | `TGATGACTCAG` |
| MA0478.1 | chr15 | − | 62973249 | 62973259 | 1.67e-06 | 0.173 | `TGATGACTCAG` |
| MA0478.1 | chr19 | − | 56072077 | 56072087 | 1.67e-06 | 0.173 | `GGATGACTCAC` |
| MA0478.1 | chr1 | + | 42045204 | 42045214 | 2.11e-06 | 0.195 | `GGATGAGTCAT` |
| MA0478.1 | chr10 | + | 105221284 | 105221294 | 2.11e-06 | 0.195 | `GGATGAGTCAT` |
| MA0478.1 | chr1 | − | 101473809 | 101473819 | 2.34e-06 | 0.195 | `GAATGACTCAG` |
| MA0478.1 | chr2 | − | 33556968 | 33556978 | 2.34e-06 | 0.195 | `GAATGACTCAG` |
| MA0478.1 | chr3 | − | 109327133 | 109327143 | 2.34e-06 | 0.195 | `GAATGACTCAG` |
| MA0478.1 | chr1 | − | 111937070 | 111937080 | 2.56e-06 | 0.199 | `TGGTGACTCAT` |
| MA0478.1 | chr6 | − | 158102532 | 158102542 | 2.56e-06 | 0.199 | `TGGTGACTCAT` |
| MA0478.1 | chr2 | + | 9714103 | 9714113 | 2.81e-06 | 0.204 | `GGATGAGTCAG` |
| MA0478.1 | chr10 | + | 93851196 | 93851206 | 2.81e-06 | 0.204 | `GGATGAGTCAG` |
| MA0478.1 | chr1 | − | 84744895 | 84744905 | 3.32e-06 | 0.206 | `GGGTGACTCAC` |
| MA0478.1 | chr3 | − | 151964085 | 151964095 | 3.32e-06 | 0.206 | `TGGTGACTCAG` |
| MA0478.1 | chr14 | − | 67747367 | 67747377 | 3.32e-06 | 0.206 | `TGGTGACTCAG` |
| MA0478.1 | chr16 | + | 80674077 | 80674087 | 3.32e-06 | 0.206 | `TGGTGACTCAG` |
| MA0478.1 | chr17 | + | 39781215 | 39781225 | 3.32e-06 | 0.206 | `TGGTGACTCAG` |
| MA0478.1 | chr9 | + | 126056587 | 126056597 | 3.55e-06 | 0.214 | `CGATGACTCAT` |
| MA0478.1 | chr6 | − | 133182587 | 133182597 | 4e-06 | 0.234 | `GAGTGACTCAT` |
| MA0478.1 | chr5 | − | 72986174 | 72986184 | 4.2e-06 | 0.234 | `TAATGACTCAT` |
| MA0478.1 | chr17 | + | 55275450 | 55275460 | 4.2e-06 | 0.234 | `TAATGACTCAT` |
| MA0478.1 | chr1 | + | 12034810 | 12034820 | 4.66e-06 | 0.236 | `AGATGACTCAT` |
| MA0478.1 | chr6 | + | 43005922 | 43005932 | 4.66e-06 | 0.236 | `AGATGACTCAT` |
| MA0478.1 | chr7 | − | 92277129 | 92277139 | 4.66e-06 | 0.236 | `CGATGACTCAG` |
| MA0478.1 | chr14 | − | 96842961 | 96842971 | 4.66e-06 | 0.236 | `AGATGACTCAT` |
| MA0478.1 | chr1 | + | 32065517 | 32065527 | 4.9e-06 | 0.237 | `GGGTGAGTCAT` |
| MA0478.1 | chr19 | + | 56072075 | 56072085 | 4.9e-06 | 0.237 | `GGGTGAGTCAT` |
| MA0478.1 | chr2 | + | 8359756 | 8359766 | 5.36e-06 | 0.238 | `TGATGAGTCAT` |
| MA0478.1 | chr3 | − | 10237514 | 10237524 | 5.36e-06 | 0.238 | `TGATGAGTCAT` |
| MA0478.1 | chr15 | − | 49308103 | 49308113 | 5.36e-06 | 0.238 | `TGATGAGTCAT` |
| MA0478.1 | chr2 | − | 8361013 | 8361023 | 5.8e-06 | 0.238 | `GAATGACTCAC` |
| MA0478.1 | chr15 | + | 57623463 | 57623473 | 5.8e-06 | 0.238 | `TAATGACTCAG` |
| MA0478.1 | chr18 | − | 64811167 | 64811177 | 5.8e-06 | 0.238 | `TAATGACTCAG` |
| MA0478.1 | chr3 | + | 151967109 | 151967119 | 6.03e-06 | 0.238 | `AGATGACTCAG` |
| MA0478.1 | chr7 | − | 42875702 | 42875712 | 6.03e-06 | 0.238 | `AGATGACTCAG` |
| MA0478.1 | chr8 | − | 126415642 | 126415652 | 6.03e-06 | 0.238 | `AGATGACTCAG` |
| MA0478.1 | chr12 | − | 99906083 | 99906093 | 6.03e-06 | 0.238 | `AGATGACTCAG` |
| MA0478.1 | chr2 | − | 231162661 | 231162671 | 6.29e-06 | 0.24 | `GGGTGAGTCAG` |
| MA0478.1 | chr19 | − | 47141510 | 47141520 | 6.29e-06 | 0.24 | `GGGTGAGTCAG` |
| MA0478.1 | chr1 | + | 201598014 | 201598024 | 6.76e-06 | 0.241 | `TGATGAGTCAG` |
| MA0478.1 | chr6 | − | 36756287 | 36756297 | 6.76e-06 | 0.241 | `TGATGAGTCAG` |
| MA0478.1 | chr10 | − | 73603928 | 73603938 | 6.76e-06 | 0.241 | `TGATGAGTCAG` |
| MA0478.1 | chr12 | + | 123967749 | 123967759 | 6.76e-06 | 0.241 | `GGATGAGTCAC` |
| MA0478.1 | chrX | − | 48654513 | 48654523 | 7.26e-06 | 0.242 | `TGGTGACTCAC` |
| MA0478.1 | chr10 | + | 45236780 | 45236790 | 7.26e-06 | 0.242 | `TGGTGACTCAC` |
| MA0478.1 | chr16 | + | 9129158 | 9129168 | 7.26e-06 | 0.242 | `TGGTGACTCAC` |
| MA0478.1 | chr20 | + | 5689557 | 5689567 | 7.26e-06 | 0.242 | `TGGTGACTCAC` |
| MA0478.1 | chr15 | + | 81298542 | 81298552 | 7.47e-06 | 0.242 | `GAATGAGTCAT` |
| MA0478.1 | chr12 | − | 50651584 | 50651594 | 7.68e-06 | 0.242 | `TAGTGACTCAT` |
| MA0478.1 | chr9 | + | 113814511 | 113814521 | 8.17e-06 | 0.242 | `AGGTGACTCAT` |
| MA0478.1 | chr10 | − | 126880012 | 126880022 | 8.17e-06 | 0.242 | `AGGTGACTCAT` |
| MA0478.1 | chr11 | − | 118068486 | 118068496 | 8.17e-06 | 0.242 | `CGGTGACTCAG` |
| MA0478.1 | chr6 | − | 74282326 | 74282336 | 8.64e-06 | 0.242 | `GAATGAGTCAG` |
| MA0478.1 | chrX | + | 46382445 | 46382455 | 8.64e-06 | 0.242 | `GAATGAGTCAG` |
| MA0478.1 | chr10 | + | 126879781 | 126879791 | 8.64e-06 | 0.242 | `GAATGAGTCAG` |
| MA0478.1 | chr14 | + | 94801763 | 94801773 | 8.87e-06 | 0.242 | `TGGTGAGTCAT` |
| MA0478.1 | chr18 | + | 58976996 | 58977006 | 8.87e-06 | 0.242 | `TGGTGAGTCAT` |
| MA0478.1 | chr11 | − | 124446208 | 124446218 | 9.35e-06 | 0.242 | `TAGTGACTCAG` |
| MA0478.1 | chr1 | + | 22318195 | 22318205 | 9.59e-06 | 0.242 | `AGGTGACTCAG` |
| MA0478.1 | chr3 | − | 10240586 | 10240596 | 9.59e-06 | 0.242 | `AGGTGACTCAG` |
| MA0478.1 | chr6 | − | 134610528 | 134610538 | 9.8e-06 | 0.242 | `CAATGACTCAT` |
| MA0478.1 | chr18 | + | 19994231 | 19994241 | 9.8e-06 | 0.242 | `CAATGACTCAT` |
| MA0478.1 | chr18 | − | 58976998 | 58977008 | 1e-05 | 0.242 | `TAATGACTCAC` |
| MA0478.1 | chr2 | − | 201831001 | 201831011 | 1.02e-05 | 0.242 | `AGATGACTCAC` |
| MA0478.1 | chr18 | + | 54888942 | 54888952 | 1.02e-05 | 0.242 | `AGATGACTCAC` |
| MA0478.1 | chr1 | + | 158862658 | 158862668 | 1.08e-05 | 0.242 | `TGGTGAGTCAG` |
| MA0478.1 | chr3 | + | 17168390 | 17168400 | 1.08e-05 | 0.242 | `GGGTGAGTCAC` |
| MA0478.1 | chr3 | − | 179719594 | 179719604 | 1.08e-05 | 0.242 | `GGGTGAGTCAC` |
| MA0478.1 | chr16 | − | 80674051 | 80674061 | 1.08e-05 | 0.242 | `TGGTGAGTCAG` |
| MA0478.1 | chr18 | + | 47227916 | 47227926 | 1.08e-05 | 0.242 | `TGGTGAGTCAG` |
| MA0478.1 | chr1 | + | 165898971 | 165898981 | 1.16e-05 | 0.242 | `AAATGACTCAT` |
| MA0478.1 | chr5 | + | 33886137 | 33886147 | 1.16e-05 | 0.242 | `CAATGACTCAG` |
| MA0478.1 | chrX | − | 29483721 | 29483731 | 1.16e-05 | 0.242 | `AAATGACTCAT` |
| MA0478.1 | chr12 | − | 26343401 | 26343411 | 1.16e-05 | 0.242 | `AAATGACTCAT` |
| MA0478.1 | chr12 | + | 50651582 | 50651592 | 1.16e-05 | 0.242 | `TGATGAGTCAC` |
| MA0478.1 | chr14 | + | 105119553 | 105119563 | 1.16e-05 | 0.242 | `CAATGACTCAG` |
| MA0478.1 | chr14 | + | 105238482 | 105238492 | 1.16e-05 | 0.242 | `CAATGACTCAG` |
| MA0478.1 | chr15 | + | 42028331 | 42028341 | 1.16e-05 | 0.242 | `TGATGAGTCAC` |
| MA0478.1 | chr22 | + | 38045269 | 38045279 | 1.16e-05 | 0.242 | `AAATGACTCAT` |
| MA0478.1 | chr2 | + | 201830999 | 201831009 | 1.19e-05 | 0.242 | `GAGTGAGTCAT` |
| MA0478.1 | chr2 | − | 149133357 | 149133367 | 1.21e-05 | 0.242 | `TAATGAGTCAT` |
| MA0478.1 | chrX | + | 29483719 | 29483729 | 1.21e-05 | 0.242 | `TAATGAGTCAT` |
| MA0478.1 | chr12 | + | 26343399 | 26343409 | 1.21e-05 | 0.242 | `TAATGAGTCAT` |
| MA0478.1 | chr1 | − | 12034812 | 12034822 | 1.25e-05 | 0.242 | `AGATGAGTCAT` |
| MA0478.1 | chr1 | − | 165898973 | 165898983 | 1.25e-05 | 0.242 | `AGATGAGTCAT` |
| MA0478.1 | chr5 | + | 10685370 | 10685380 | 1.25e-05 | 0.242 | `AGATGAGTCAT` |
| MA0478.1 | chr6 | − | 43005924 | 43005934 | 1.25e-05 | 0.242 | `AGATGAGTCAT` |
| MA0478.1 | chr10 | − | 26816704 | 26816714 | 1.25e-05 | 0.242 | `CGATGAGTCAG` |
| MA0478.1 | chr14 | + | 96842959 | 96842969 | 1.25e-05 | 0.242 | `AGATGAGTCAT` |
| MA0478.1 | chr17 | − | 22684097 | 22684107 | 1.25e-05 | 0.242 | `AGATGAGTCAT` |
| MA0478.1 | chr20 | − | 47338125 | 47338135 | 1.25e-05 | 0.242 | `AGATGAGTCAT` |
| MA0478.1 | chr1 | + | 190815194 | 190815204 | 1.27e-05 | 0.242 | `AAATGACTCAG` |
| MA0478.1 | chr6 | − | 24992019 | 24992029 | 1.27e-05 | 0.242 | `AAATGACTCAG` |
| MA0478.1 | chr7 | − | 126128608 | 126128618 | 1.27e-05 | 0.242 | `AAATGACTCAG` |
| MA0478.1 | chr10 | − | 125969161 | 125969171 | 1.27e-05 | 0.242 | `AAATGACTCAG` |
| MA0478.1 | chr19 | − | 38463469 | 38463479 | 1.27e-05 | 0.242 | `AAATGACTCAG` |
| MA0478.1 | chr19 | − | 2513188 | 2513198 | 1.32e-05 | 0.242 | `CGGTGACTCAC` |
| MA0478.1 | chr1 | − | 228312594 | 228312604 | 1.37e-05 | 0.242 | `GAATGAGTCAC` |
| MA0478.1 | chr2 | − | 11887987 | 11887997 | 1.37e-05 | 0.242 | `GAATGAGTCAC` |
| MA0478.1 | chr2 | − | 33573009 | 33573019 | 1.37e-05 | 0.242 | `GAATGAGTCAC` |
| MA0478.1 | chr3 | − | 123811782 | 123811792 | 1.37e-05 | 0.242 | `TAATGAGTCAG` |
| MA0478.1 | chr7 | + | 23025052 | 23025062 | 1.37e-05 | 0.242 | `GAATGAGTCAC` |
| MA0478.1 | chrX | + | 7012308 | 7012318 | 1.37e-05 | 0.242 | `GAATGAGTCAC` |
| MA0478.1 | chr10 | + | 126880010 | 126880020 | 1.37e-05 | 0.242 | `GAATGAGTCAC` |
| MA0478.1 | chr13 | − | 108612748 | 108612758 | 1.37e-05 | 0.242 | `TAATGAGTCAG` |
| MA0478.1 | chr4 | − | 152885427 | 152885437 | 1.41e-05 | 0.242 | `CAGTGACTCAT` |
| MA0478.1 | chr12 | − | 123967751 | 123967761 | 1.41e-05 | 0.242 | `CAGTGACTCAT` |
| MA0478.1 | chr17 | − | 35168152 | 35168162 | 1.41e-05 | 0.242 | `CAGTGACTCAT` |
| MA0478.1 | chr4 | + | 77441700 | 77441710 | 1.46e-05 | 0.242 | `AGATGAGTCAG` |
| MA0478.1 | chr6 | + | 20985758 | 20985768 | 1.46e-05 | 0.242 | `TAGTGACTCAC` |
| MA0478.1 | chr10 | − | 51941704 | 51941714 | 1.46e-05 | 0.242 | `AGATGAGTCAG` |
| MA0478.1 | chr17 | + | 1573179 | 1573189 | 1.46e-05 | 0.242 | `AGATGAGTCAG` |
| MA0478.1 | chr17 | − | 35970777 | 35970787 | 1.46e-05 | 0.242 | `AGATGAGTCAG` |
| MA0478.1 | chr2 | + | 33573007 | 33573017 | 1.6e-05 | 0.246 | `AAGTGACTCAT` |
| MA0478.1 | chr6 | − | 31239665 | 31239675 | 1.6e-05 | 0.246 | `AAGTGACTCAT` |
| MA0478.1 | chr7 | + | 41991197 | 41991207 | 1.6e-05 | 0.246 | `TGGTGAGTCAC` |
| MA0478.1 | chr8 | − | 125003771 | 125003781 | 1.6e-05 | 0.246 | `CAGTGACTCAG` |
| MA0478.1 | chr14 | − | 61199144 | 61199154 | 1.6e-05 | 0.246 | `GCATGACTCAG` |
| MA0478.1 | chr3 | − | 109302685 | 109302695 | 1.62e-05 | 0.246 | `GTATGACTCAT` |
| MA0478.1 | chr10 | − | 11327674 | 11327684 | 1.62e-05 | 0.246 | `GTATGACTCAT` |
| MA0478.1 | chr11 | + | 90673017 | 90673027 | 1.65e-05 | 0.246 | `CAATGACTCAC` |
| MA0478.1 | chr1 | − | 12039274 | 12039284 | 1.72e-05 | 0.246 | `AGGTGAGTCAT` |
| MA0478.1 | chr8 | + | 57149550 | 57149560 | 1.72e-05 | 0.246 | `CGGTGAGTCAG` |
| MA0478.1 | chr9 | + | 131688104 | 131688114 | 1.72e-05 | 0.246 | `CGGTGAGTCAG` |
| MA0478.1 | chr10 | − | 90021194 | 90021204 | 1.72e-05 | 0.246 | `AGGTGAGTCAT` |
| MA0478.1 | chr11 | − | 65076286 | 65076296 | 1.72e-05 | 0.246 | `CGGTGAGTCAG` |
| MA0478.1 | chr11 | + | 127847469 | 127847479 | 1.72e-05 | 0.246 | `AGGTGAGTCAT` |
| MA0478.1 | chr16 | − | 55536592 | 55536602 | 1.72e-05 | 0.246 | `AGGTGAGTCAT` |
| MA0478.1 | chr9 | + | 93010338 | 93010348 | 1.74e-05 | 0.246 | `AAGTGACTCAG` |
| MA0478.1 | chr9 | + | 37399525 | 37399535 | 1.76e-05 | 0.246 | `GTATGACTCAG` |
| MA0478.1 | chr1 | + | 12039272 | 12039282 | 1.81e-05 | 0.246 | `AAATGACTCAC` |
| MA0478.1 | chrX | − | 46373353 | 46373363 | 1.81e-05 | 0.246 | `AAATGACTCAC` |
| MA0478.1 | chr16 | + | 55536590 | 55536600 | 1.81e-05 | 0.246 | `AAATGACTCAC` |
| MA0478.1 | chr20 | − | 30409267 | 30409277 | 1.81e-05 | 0.246 | `AAATGACTCAC` |
| MA0478.1 | chr1 | − | 171431391 | 171431401 | 1.86e-05 | 0.246 | `TAGTGAGTCAG` |
| MA0478.1 | chr9 | − | 6671231 | 6671241 | 1.86e-05 | 0.246 | `TAGTGAGTCAG` |
| MA0478.1 | chr10 | − | 45236782 | 45236792 | 1.86e-05 | 0.246 | `GAGTGAGTCAC` |
| MA0478.1 | chr12 | − | 46493077 | 46493087 | 1.86e-05 | 0.246 | `GAGTGAGTCAC` |
| MA0478.1 | chr17 | + | 55218813 | 55218823 | 1.86e-05 | 0.246 | `GAGTGAGTCAC` |
| MA0478.1 | chr19 | + | 44586463 | 44586473 | 1.86e-05 | 0.246 | `GAGTGAGTCAC` |
| MA0478.1 | chr7 | + | 139263068 | 139263078 | 1.9e-05 | 0.246 | `AGGTGAGTCAG` |
| MA0478.1 | chr16 | − | 8937932 | 8937942 | 1.9e-05 | 0.246 | `AGGTGAGTCAG` |
| MA0478.1 | chr19 | − | 48961417 | 48961427 | 1.9e-05 | 0.246 | `AGGTGAGTCAG` |
| MA0478.1 | chr19 | − | 49500955 | 49500965 | 1.9e-05 | 0.246 | `AGGTGAGTCAG` |
| MA0478.1 | chr6 | + | 31239663 | 31239673 | 1.95e-05 | 0.246 | `TAATGAGTCAC` |
| MA0478.1 | chr3 | + | 151966737 | 151966747 | 1.97e-05 | 0.246 | `TCATGACTCAT` |
| MA0478.1 | chr5 | + | 53152560 | 53152570 | 1.97e-05 | 0.246 | `TCATGACTCAT` |
| MA0478.1 | chr15 | + | 49308101 | 49308111 | 1.97e-05 | 0.246 | `TCATGACTCAT` |
| MA0478.1 | chr16 | + | 19474229 | 19474239 | 1.97e-05 | 0.246 | `TCATGACTCAT` |
| MA0478.1 | chr11 | + | 131733040 | 131733050 | 1.99e-05 | 0.246 | `AGATGAGTCAC` |
| MA0478.1 | chr6 | − | 41782081 | 41782091 | 2.02e-05 | 0.246 | `GCGTGACTCAG` |
| MA0478.1 | chr13 | − | 49838144 | 49838154 | 2.04e-05 | 0.246 | `GTGTGACTCAT` |
| MA0478.1 | chr1 | + | 233214231 | 233214241 | 2.08e-05 | 0.246 | `AAATGAGTCAT` |
| MA0478.1 | chr3 | + | 109302683 | 109302693 | 2.08e-05 | 0.246 | `AAATGAGTCAT` |
| MA0478.1 | chr3 | − | 151966739 | 151966749 | 2.08e-05 | 0.246 | `AAATGAGTCAT` |
| MA0478.1 | chr5 | − | 53152562 | 53152572 | 2.08e-05 | 0.246 | `AAATGAGTCAT` |
| MA0478.1 | chr6 | + | 45996705 | 45996715 | 2.08e-05 | 0.246 | `AAATGAGTCAT` |
| MA0478.1 | chr9 | − | 126056589 | 126056599 | 2.08e-05 | 0.246 | `AAATGAGTCAT` |
| MA0478.1 | chr9 | + | 129248832 | 129248842 | 2.08e-05 | 0.246 | `AAATGAGTCAT` |
| MA0478.1 | chr10 | + | 121454160 | 121454170 | 2.08e-05 | 0.246 | `CAATGAGTCAG` |
| MA0478.1 | chr14 | + | 72013528 | 72013538 | 2.08e-05 | 0.246 | `AAATGAGTCAT` |
| MA0478.1 | chr14 | − | 75023987 | 75023997 | 2.08e-05 | 0.246 | `AAATGAGTCAT` |
| MA0478.1 | chr17 | − | 55275452 | 55275462 | 2.08e-05 | 0.246 | `AAATGAGTCAT` |
| MA0478.1 | chr1 | − | 110979061 | 110979071 | 2.15e-05 | 0.246 | `CAGTGACTCAC` |
| MA0478.1 | chr3 | − | 53111878 | 53111888 | 2.15e-05 | 0.246 | `TCATGACTCAG` |
| MA0478.1 | chr7 | − | 41991199 | 41991209 | 2.15e-05 | 0.246 | `CAGTGACTCAC` |
| MA0478.1 | chr10 | + | 112164636 | 112164646 | 2.15e-05 | 0.246 | `CAGTGACTCAC` |
| MA0478.1 | chr12 | − | 91457285 | 91457295 | 2.15e-05 | 0.246 | `GCATGACTCAC` |
| MA0478.1 | chr17 | + | 3561076 | 3561086 | 2.15e-05 | 0.246 | `GCATGACTCAC` |
| MA0478.1 | chr20 | + | 45821057 | 45821067 | 2.15e-05 | 0.246 | `TCATGACTCAG` |
| MA0478.1 | chr1 | − | 3583624 | 3583634 | 2.2e-05 | 0.246 | `GTGTGACTCAG` |
| MA0478.1 | chr3 | + | 58835701 | 58835711 | 2.22e-05 | 0.246 | `AAATGAGTCAG` |
| MA0478.1 | chr3 | − | 99757907 | 99757917 | 2.22e-05 | 0.246 | `AAATGAGTCAG` |
| MA0478.1 | chr4 | − | 2908517 | 2908527 | 2.22e-05 | 0.246 | `AAATGAGTCAG` |
| MA0478.1 | chr7 | + | 126128452 | 126128462 | 2.22e-05 | 0.246 | `AAATGAGTCAG` |
| MA0478.1 | chr8 | − | 105750857 | 105750867 | 2.22e-05 | 0.246 | `AAATGAGTCAG` |
| MA0478.1 | chr10 | − | 81946158 | 81946168 | 2.22e-05 | 0.246 | `AAATGAGTCAG` |
| MA0478.1 | chr12 | + | 120810849 | 120810859 | 2.22e-05 | 0.246 | `AAATGAGTCAG` |
| MA0478.1 | chr12 | + | 26844142 | 26844152 | 2.27e-05 | 0.251 | `AAGTGACTCAC` |
| MA0478.1 | chr11 | − | 127847471 | 127847481 | 2.31e-05 | 0.253 | `GTATGACTCAC` |
| MA0478.1 | chr2 | + | 232809343 | 232809353 | 2.36e-05 | 0.253 | `CAGTGAGTCAT` |
| MA0478.1 | chr4 | + | 14467328 | 14467338 | 2.36e-05 | 0.253 | `CAGTGAGTCAT` |
| MA0478.1 | chr17 | − | 3561078 | 3561088 | 2.36e-05 | 0.253 | `CAGTGAGTCAT` |
| MA0478.1 | chr18 | − | 19994233 | 19994243 | 2.36e-05 | 0.253 | `GCATGAGTCAT` |
| MA0478.1 | chr20 | + | 30409265 | 30409275 | 2.36e-05 | 0.253 | `CAGTGAGTCAT` |
| MA0478.1 | chr12 | − | 26844144 | 26844154 | 2.38e-05 | 0.254 | `TAGTGAGTCAC` |
| MA0478.1 | chr9 | − | 91268840 | 91268850 | 2.43e-05 | 0.258 | `AGGTGAGTCAC` |
| MA0478.1 | chr2 | + | 8361011 | 8361021 | 2.5e-05 | 0.26 | `AAGTGAGTCAT` |
| MA0478.1 | chr11 | − | 90673019 | 90673029 | 2.5e-05 | 0.26 | `AAGTGAGTCAT` |
| MA0478.1 | chr12 | + | 2315912 | 2315922 | 2.5e-05 | 0.26 | `CAGTGAGTCAG` |
| MA0478.1 | chr19 | + | 54070503 | 54070513 | 2.5e-05 | 0.26 | `CAGTGAGTCAG` |
| MA0478.1 | chr1 | + | 101609570 | 101609580 | 2.52e-05 | 0.261 | `GTATGAGTCAT` |
| MA0478.1 | chr11 | + | 58102357 | 58102367 | 2.57e-05 | 0.263 | `TCGTGACTCAG` |
| MA0478.1 | chr1 | + | 228312592 | 228312602 | 2.59e-05 | 0.263 | `TTGTGACTCAT` |
| MA0478.1 | chrX | − | 7012310 | 7012320 | 2.59e-05 | 0.263 | `TTGTGACTCAT` |
| MA0478.1 | chr11 | − | 131733042 | 131733052 | 2.59e-05 | 0.263 | `TTGTGACTCAT` |
| MA0478.1 | chr13 | + | 49838142 | 49838152 | 2.61e-05 | 0.263 | `CAATGAGTCAC` |
| MA0478.1 | chr3 | + | 10237512 | 10237522 | 2.64e-05 | 0.263 | `CCATGACTCAT` |
| MA0478.1 | chr5 | − | 86448877 | 86448887 | 2.64e-05 | 0.263 | `CCATGACTCAT` |
| MA0478.1 | chr1 | − | 32065519 | 32065529 | 2.66e-05 | 0.263 | `TCATGACTCAC` |
| MA0478.1 | chr3 | + | 49257467 | 49257477 | 2.68e-05 | 0.263 | `AAGTGAGTCAG` |
| MA0478.1 | chr3 | + | 151298169 | 151298179 | 2.68e-05 | 0.263 | `AAGTGAGTCAG` |
| MA0478.1 | chr17 | + | 46585738 | 46585748 | 2.68e-05 | 0.263 | `AAGTGAGTCAG` |
| MA0478.1 | chr18 | + | 667132 | 667142 | 2.68e-05 | 0.263 | `AAGTGAGTCAG` |
| MA0478.1 | chr16 | + | 3161761 | 3161771 | 2.71e-05 | 0.264 | `GTATGAGTCAG` |
| MA0478.1 | chr3 | − | 17168392 | 17168402 | 2.75e-05 | 0.264 | `GTGTGACTCAC` |
| MA0478.1 | chr7 | + | 134505028 | 134505038 | 2.75e-05 | 0.264 | `TTGTGACTCAG` |
| MA0478.1 | chr9 | + | 91268838 | 91268848 | 2.75e-05 | 0.264 | `GTGTGACTCAC` |
| MA0478.1 | chr1 | + | 111937068 | 111937078 | 2.77e-05 | 0.264 | `AAATGAGTCAC` |
| MA0478.1 | chr1 | − | 25748941 | 25748951 | 2.82e-05 | 0.264 | `ACATGACTCAT` |
| MA0478.1 | chr4 | − | 8281379 | 8281389 | 2.82e-05 | 0.264 | `CCATGACTCAG` |
| MA0478.1 | chr6 | + | 20985890 | 20985900 | 2.82e-05 | 0.264 | `ACATGACTCAT` |
| MA0478.1 | chr6 | − | 45996707 | 45996717 | 2.82e-05 | 0.264 | `ACATGACTCAT` |
| MA0478.1 | chr12 | − | 121952549 | 121952559 | 2.82e-05 | 0.264 | `CCATGACTCAG` |
| MA0478.1 | chrX | + | 46373351 | 46373361 | 2.87e-05 | 0.265 | `GCGTGAGTCAT` |
| MA0478.1 | chr17 | + | 22684095 | 22684105 | 2.87e-05 | 0.265 | `CTATGACTCAT` |
| MA0478.1 | chr2 | − | 232809345 | 232809355 | 2.89e-05 | 0.265 | `TTATGACTCAC` |
| MA0478.1 | chr4 | − | 14467330 | 14467340 | 2.89e-05 | 0.265 | `TTATGACTCAC` |
| MA0478.1 | chr10 | + | 90021192 | 90021202 | 2.89e-05 | 0.265 | `TTATGACTCAC` |
| MA0478.1 | chr6 | − | 20985892 | 20985902 | 2.91e-05 | 0.266 | `TCATGAGTCAT` |
| MA0478.1 | chr1 | − | 223683190 | 223683200 | 2.93e-05 | 0.266 | `ACATGACTCAG` |
| MA0478.1 | chr10 | − | 122356078 | 122356088 | 2.93e-05 | 0.266 | `ACATGACTCAG` |
| MA0478.1 | chr1 | − | 101609572 | 101609582 | 3e-05 | 0.266 | `ATATGACTCAT` |
| MA0478.1 | chr9 | + | 116151308 | 116151318 | 3e-05 | 0.266 | `GCGTGAGTCAG` |
| MA0478.1 | chr9 | − | 129248834 | 129248844 | 3e-05 | 0.266 | `ATATGACTCAT` |
| MA0478.1 | chr14 | + | 75023985 | 75023995 | 3e-05 | 0.266 | `ATATGACTCAT` |
| MA0478.1 | chr17 | − | 30414913 | 30414923 | 3e-05 | 0.266 | `GCGTGAGTCAG` |
| MA0478.1 | chr6 | − | 20985760 | 20985770 | 3.09e-05 | 0.267 | `CAGTGAGTCAC` |
| MA0478.1 | chr6 | − | 31430624 | 31430634 | 3.09e-05 | 0.267 | `TCATGAGTCAG` |
| MA0478.1 | chr6 | − | 31430624 | 31430634 | 3.09e-05 | 0.267 | `TCATGAGTCAG` |
| MA0478.1 | chr9 | + | 84959215 | 84959225 | 3.09e-05 | 0.267 | `GCATGAGTCAC` |
| MA0478.1 | chr11 | − | 63748279 | 63748289 | 3.09e-05 | 0.267 | `GCATGAGTCAC` |
| MA0478.1 | chr18 | + | 19065528 | 19065538 | 3.09e-05 | 0.267 | `CAGTGAGTCAC` |
| MA0478.1 | chr22 | − | 27523904 | 27523914 | 3.09e-05 | 0.267 | `TCATGAGTCAG` |
| MA0478.1 | chr7 | − | 23025054 | 23025064 | 3.14e-05 | 0.269 | `CCGTGACTCAT` |
| MA0478.1 | chr1 | − | 101609490 | 101609500 | 3.18e-05 | 0.269 | `ATATGACTCAG` |
| MA0478.1 | chr22 | − | 27525167 | 27525177 | 3.18e-05 | 0.269 | `ATATGACTCAG` |
| MA0478.1 | chr3 | + | 120553959 | 120553969 | 3.21e-05 | 0.269 | `GTGTGAGTCAG` |
| MA0478.1 | chr8 | − | 61984273 | 61984283 | 3.21e-05 | 0.269 | `GTGTGAGTCAG` |
| MA0478.1 | chr16 | − | 11330070 | 11330080 | 3.21e-05 | 0.269 | `GTGTGAGTCAG` |
| MA0478.1 | chr19 | + | 2427627 | 2427637 | 3.21e-05 | 0.269 | `GTGTGAGTCAG` |
| MA0478.1 | chrX | + | 48654511 | 48654521 | 3.23e-05 | 0.269 | `AAGTGAGTCAC` |
| MA0478.1 | chr16 | − | 9129160 | 9129170 | 3.23e-05 | 0.269 | `AAGTGAGTCAC` |
| MA0478.1 | chr2 | − | 96172386 | 96172396 | 3.32e-05 | 0.271 | `CCGTGACTCAG` |
| MA0478.1 | chr7 | − | 120706231 | 120706241 | 3.32e-05 | 0.271 | `TTATGAGTCAG` |
| MA0478.1 | chr2 | + | 11887985 | 11887995 | 3.35e-05 | 0.271 | `CTGTGACTCAT` |
| MA0478.1 | chr9 | − | 84959217 | 84959227 | 3.35e-05 | 0.271 | `CTGTGACTCAT` |
| MA0478.1 | chr10 | − | 11324629 | 11324639 | 3.35e-05 | 0.271 | `CTGTGACTCAT` |
| MA0478.1 | chr11 | − | 63764553 | 63764563 | 3.35e-05 | 0.271 | `CTGTGACTCAT` |
| MA0478.1 | chr1 | − | 28709105 | 28709115 | 3.37e-05 | 0.271 | `TTGTGACTCAC` |
| MA0478.1 | chr4 | − | 14467377 | 14467387 | 3.37e-05 | 0.271 | `TTGTGACTCAC` |
| MA0478.1 | chr22 | − | 40588207 | 40588217 | 3.39e-05 | 0.271 | `CCATGACTCAC` |
| MA0478.1 | chr3 | − | 13348780 | 13348790 | 3.44e-05 | 0.271 | `ACGTGACTCAG` |
| MA0478.1 | chr2 | + | 10140716 | 10140726 | 3.49e-05 | 0.271 | `ATGTGACTCAT` |
| MA0478.1 | chr3 | − | 45570318 | 45570328 | 3.49e-05 | 0.271 | `CTGTGACTCAG` |
| MA0478.1 | chr5 | + | 131789420 | 131789430 | 3.49e-05 | 0.271 | `CTGTGACTCAG` |
| MA0478.1 | chr7 | − | 929692 | 929702 | 3.49e-05 | 0.271 | `CTGTGACTCAG` |
| MA0478.1 | chr9 | + | 67903670 | 67903680 | 3.49e-05 | 0.271 | `CTGTGACTCAG` |
| MA0478.1 | chr10 | − | 11319672 | 11319682 | 3.49e-05 | 0.271 | `CTGTGACTCAG` |
| MA0478.1 | chr11 | + | 63748277 | 63748287 | 3.49e-05 | 0.271 | `ATGTGACTCAT` |
| MA0478.1 | chr11 | − | 74489139 | 74489149 | 3.49e-05 | 0.271 | `CTGTGACTCAG` |
| MA0478.1 | chr15 | − | 42028333 | 42028343 | 3.49e-05 | 0.271 | `ATGTGACTCAT` |
| MA0478.1 | chr2 | + | 231499110 | 231499120 | 3.51e-05 | 0.272 | `GGATGATTCAT` |
| MA0478.1 | chr1 | + | 84744893 | 84744903 | 3.61e-05 | 0.275 | `GCGTGAGTCAC` |
| MA0478.1 | chr8 | + | 126511704 | 126511714 | 3.61e-05 | 0.275 | `GCGTGAGTCAC` |
| MA0478.1 | chr22 | + | 40588205 | 40588215 | 3.63e-05 | 0.275 | `TTGTGAGTCAT` |
| MA0478.1 | chr5 | + | 72986172 | 72986182 | 3.65e-05 | 0.275 | `CCATGAGTCAT` |
| MA0478.1 | chr6 | + | 134610526 | 134610536 | 3.65e-05 | 0.275 | `CCATGAGTCAT` |
| MA0478.1 | chr22 | − | 38045271 | 38045281 | 3.65e-05 | 0.275 | `CCATGAGTCAT` |
| MA0478.1 | chr4 | + | 152885425 | 152885435 | 3.7e-05 | 0.275 | `TCATGAGTCAC` |
| MA0478.1 | chr10 | + | 11324627 | 11324637 | 3.7e-05 | 0.275 | `TCATGAGTCAC` |
| MA0478.1 | chr11 | + | 63764551 | 63764561 | 3.7e-05 | 0.275 | `TCATGAGTCAC` |
| MA0478.1 | chr12 | + | 2315944 | 2315954 | 3.7e-05 | 0.275 | `ATGTGACTCAG` |
| MA0478.1 | chr17 | + | 57525746 | 57525756 | 3.7e-05 | 0.275 | `ATGTGACTCAG` |
| MA0478.1 | chr14 | − | 94801765 | 94801775 | 3.74e-05 | 0.278 | `ATATGACTCAC` |
| MA0478.1 | chr3 | − | 57077565 | 57077575 | 3.79e-05 | 0.278 | `TTGTGAGTCAG` |
| MA0478.1 | chr4 | + | 14467375 | 14467385 | 3.79e-05 | 0.278 | `GTGTGAGTCAC` |
| MA0478.1 | chr1 | + | 25748939 | 25748949 | 3.83e-05 | 0.278 | `ACATGAGTCAT` |
| MA0478.1 | chr2 | − | 169060467 | 169060477 | 3.83e-05 | 0.278 | `ACATGAGTCAT` |
| MA0478.1 | chr5 | + | 86448875 | 86448885 | 3.83e-05 | 0.278 | `ACATGAGTCAT` |
| MA0478.1 | chr11 | + | 68821641 | 68821651 | 3.83e-05 | 0.278 | `CCATGAGTCAG` |
| MA0478.1 | chr16 | − | 51690416 | 51690426 | 3.83e-05 | 0.278 | `CCATGAGTCAG` |
| MA0478.1 | chr16 | − | 19474231 | 19474241 | 3.85e-05 | 0.279 | `CTATGAGTCAT` |
| MA0478.1 | chr6 | + | 158102530 | 158102540 | 3.9e-05 | 0.279 | `TTATGAGTCAC` |
| MA0478.1 | chr12 | + | 46493075 | 46493085 | 3.9e-05 | 0.279 | `CCGTGACTCAC` |
| MA0478.1 | chr19 | − | 44586465 | 44586475 | 3.9e-05 | 0.279 | `CCGTGACTCAC` |
| MA0478.1 | chr10 | + | 11327672 | 11327682 | 3.99e-05 | 0.285 | `ATATGAGTCAT` |
| MA0478.1 | chr18 | − | 19065530 | 19065540 | 4.01e-05 | 0.285 | `ACGTGACTCAC` |
| MA0478.1 | chr17 | − | 55218815 | 55218825 | 4.04e-05 | 0.285 | `CTGTGACTCAC` |
| MA0478.1 | chr9 | − | 92950032 | 92950042 | 4.06e-05 | 0.285 | `TGATGATTCAT` |
| MA0478.1 | chr10 | − | 112524064 | 112524074 | 4.06e-05 | 0.285 | `TGATGATTCAT` |
| MA0478.1 | chr14 | + | 94850921 | 94850931 | 4.06e-05 | 0.285 | `TGATGATTCAT` |
| MA0478.1 | chr22 | + | 49095896 | 49095906 | 4.17e-05 | 0.292 | `ATATGAGTCAG` |
| MA0478.1 | chr3 | + | 179719592 | 179719602 | 4.2e-05 | 0.293 | `ATGTGACTCAC` |
| MA0478.1 | chr3 | − | 179722993 | 179723003 | 4.24e-05 | 0.294 | `TGATGATTCAG` |
| MA0478.1 | chr10 | + | 26816613 | 26816623 | 4.24e-05 | 0.294 | `GGATGATTCAC` |
| MA0478.1 | chr12 | + | 91457283 | 91457293 | 4.29e-05 | 0.296 | `ACGTGAGTCAT` |
| MA0478.1 | chr1 | + | 205170376 | 205170386 | 4.36e-05 | 0.299 | `GGATGACTAAG` |
| MA0478.1 | chr7 | − | 104779747 | 104779757 | 4.38e-05 | 0.299 | `GAATGATTCAT` |
| MA0478.1 | chr10 | − | 112524034 | 112524044 | 4.38e-05 | 0.299 | `GAATGATTCAT` |
| MA0478.1 | chr2 | − | 204600297 | 204600307 | 4.4e-05 | 0.299 | `CCATGAGTCAC` |
| MA0478.1 | chr9 | − | 113814513 | 113814523 | 4.4e-05 | 0.299 | `CCATGAGTCAC` |
| MA0478.1 | chr10 | − | 112117882 | 112117892 | 4.43e-05 | 0.3 | `ACGTGAGTCAG` |
| MA0478.1 | chr18 | − | 54888944 | 54888954 | 4.47e-05 | 0.302 | `ATGTGAGTCAT` |
| MA0478.1 | chr4 | + | 2908584 | 2908594 | 4.5e-05 | 0.303 | `GGATGGCTCAT` |
| MA0478.1 | chr3 | − | 123460576 | 123460586 | 4.52e-05 | 0.303 | `GAATGATTCAG` |
| MA0478.1 | chr2 | − | 10140718 | 10140728 | 4.56e-05 | 0.303 | `ACATGAGTCAC` |
| MA0478.1 | chr3 | + | 58002928 | 58002938 | 4.56e-05 | 0.303 | `TGGTGATTCAT` |
| MA0478.1 | chr6 | + | 133182585 | 133182595 | 4.56e-05 | 0.303 | `ACATGAGTCAC` |
| MA0478.1 | chr17 | + | 35168150 | 35168160 | 4.56e-05 | 0.303 | `ACATGAGTCAC` |
| MA0478.1 | chr1 | + | 224917250 | 224917260 | 4.58e-05 | 0.303 | `CTATGAGTCAC` |
| MA0478.1 | chr12 | + | 107535392 | 107535402 | 4.61e-05 | 0.304 | `GGGTGACTAAT` |
| MA0478.1 | chr18 | − | 55220779 | 55220789 | 4.63e-05 | 0.304 | `ATGTGAGTCAG` |
| MA0478.1 | chr1 | − | 228312473 | 228312483 | 4.66e-05 | 0.305 | `GGATGGCTCAG` |
| MA0478.1 | chr3 | − | 51530331 | 51530341 | 4.72e-05 | 0.309 | `TGGTGATTCAG` |
| MA0478.1 | chr18 | + | 9057180 | 9057190 | 4.81e-05 | 0.313 | `TGATGATTCAC` |
| MA0478.1 | chr1 | + | 111566209 | 111566219 | 4.83e-05 | 0.313 | `GAGTGATTCAT` |
| MA0478.1 | chr22 | − | 16119058 | 16119068 | 4.83e-05 | 0.313 | `GAGTGATTCAT` |
| MA0478.1 | chr19 | + | 2513186 | 2513196 | 4.86e-05 | 0.314 | `CCGTGAGTCAC` |
| MA0478.1 | chr4 | − | 40318657 | 40318667 | 4.9e-05 | 0.315 | `TGATGACTAAG` |
| MA0478.1 | chr5 | + | 37774174 | 37774184 | 4.9e-05 | 0.315 | `TGATGACTAAG` |
| MA0478.1 | chr1 | − | 207899988 | 207899998 | 5.03e-05 | 0.322 | `GAATGACTAAT` |
| MA0478.1 | chr1 | + | 28709103 | 28709113 | 5.06e-05 | 0.322 | `ACGTGAGTCAC` |
| MA0478.1 | chr1 | + | 110979059 | 110979069 | 5.1e-05 | 0.322 | `CTGTGAGTCAC` |
| MA0478.1 | chr2 | + | 238014439 | 238014449 | 5.1e-05 | 0.322 | `GGTTGACTCAT` |
| MA0478.1 | chr10 | + | 301222 | 301232 | 5.1e-05 | 0.322 | `GGTTGACTCAT` |
| MA0478.1 | chr22 | − | 37207760 | 37207770 | 5.1e-05 | 0.322 | `GGTTGACTCAT` |
| MA0478.1 | chr1 | + | 202742578 | 202742588 | 5.13e-05 | 0.322 | `TGATGGCTCAT` |
| MA0478.1 | chr15 | + | 56534045 | 56534055 | 5.17e-05 | 0.324 | `GAATGATTCAC` |
| MA0478.1 | chr15 | − | 88238177 | 88238187 | 5.19e-05 | 0.324 | `AGATGATTCAG` |
| MA0478.1 | chr3 | − | 157886549 | 157886559 | 5.24e-05 | 0.326 | `GGATGAGTAAT` |
| MA0478.1 | chr5 | − | 106935206 | 106935216 | 5.24e-05 | 0.326 | `GGGTGGCTCAG` |
| MA0478.1 | chr10 | − | 112164638 | 112164648 | 5.33e-05 | 0.327 | `ATGTGAGTCAC` |
| MA0478.1 | chr12 | + | 67488943 | 67488953 | 5.33e-05 | 0.327 | `GGTTGACTCAG` |
| MA0478.1 | chr16 | + | 9128968 | 9128978 | 5.33e-05 | 0.327 | `GGTTGACTCAG` |
| MA0478.1 | chr20 | − | 5689559 | 5689569 | 5.33e-05 | 0.327 | `ATGTGAGTCAC` |
| MA0478.1 | chr20 | + | 49437383 | 49437393 | 5.38e-05 | 0.33 | `TGATGGCTCAG` |
| MA0478.1 | chr3 | − | 113529206 | 113529216 | 5.47e-05 | 0.334 | `GAATGGCTCAT` |
| MA0478.1 | chr2 | + | 171884042 | 171884052 | 5.63e-05 | 0.343 | `TGATGACTAAC` |
| MA0478.1 | chr1 | − | 21493790 | 21493800 | 5.65e-05 | 0.343 | `GAGTGACTAAT` |
| MA0478.1 | chr3 | − | 23670437 | 23670447 | 5.74e-05 | 0.345 | `TGGTGGCTCAT` |
| MA0478.1 | chr10 | − | 11319923 | 11319933 | 5.74e-05 | 0.345 | `TGGTGGCTCAT` |
| MA0478.1 | chr1 | − | 190816394 | 190816404 | 5.81e-05 | 0.345 | `TAGTGATTCAG` |
| MA0478.1 | chr6 | + | 16529620 | 16529630 | 5.81e-05 | 0.345 | `TAATGACTAAT` |
| MA0478.1 | chr14 | + | 90909826 | 90909836 | 5.81e-05 | 0.345 | `TAATGACTAAT` |
| MA0478.1 | chr17 | + | 8070901 | 8070911 | 5.81e-05 | 0.345 | `TAGTGATTCAG` |
| MA0478.1 | chr22 | − | 27521811 | 27521821 | 5.81e-05 | 0.345 | `TAATGACTAAT` |
| MA0478.1 | chr1 | − | 118008759 | 118008769 | 5.89e-05 | 0.345 | `AGATGACTAAT` |
| MA0478.1 | chr21 | + | 44388173 | 44388183 | 5.89e-05 | 0.345 | `AGGTGATTCAG` |
| MA0478.1 | chr2 | + | 111643129 | 111643139 | 5.93e-05 | 0.345 | `GGGTGAGTAAT` |
| MA0478.1 | chr21 | − | 34271098 | 34271108 | 5.93e-05 | 0.345 | `CAATGATTCAT` |
| MA0478.1 | chr1 | + | 233191420 | 233191430 | 5.98e-05 | 0.345 | `GAGTGACTAAG` |
| MA0478.1 | chr4 | − | 182481508 | 182481518 | 5.98e-05 | 0.345 | `GAGTGACTAAG` |
| MA0478.1 | chr20 | − | 61953032 | 61953042 | 5.98e-05 | 0.345 | `GAGTGACTAAG` |
| MA0478.1 | chr14 | + | 54638961 | 54638971 | 6.05e-05 | 0.345 | `AGATGATTCAC` |
| MA0478.1 | chr1 | + | 234228866 | 234228876 | 6.12e-05 | 0.345 | `TGGTGGCTCAG` |
| MA0478.1 | chr5 | + | 55480158 | 55480168 | 6.12e-05 | 0.345 | `TGATGAGTAAT` |
| MA0478.1 | chr5 | + | 124067908 | 124067918 | 6.12e-05 | 0.345 | `TGGTGGCTCAG` |
| MA0478.1 | chr6 | − | 30887014 | 30887024 | 6.12e-05 | 0.345 | `TGGTGGCTCAG` |
| MA0478.1 | chr6 | + | 36742954 | 36742964 | 6.12e-05 | 0.345 | `GGGTGGCTCAC` |
| MA0478.1 | chr7 | + | 134504765 | 134504775 | 6.12e-05 | 0.345 | `TGGTGGCTCAG` |
| MA0478.1 | chr16 | + | 8937627 | 8937637 | 6.12e-05 | 0.345 | `GGGTGGCTCAC` |
| MA0478.1 | chr1 | + | 111945577 | 111945587 | 6.16e-05 | 0.345 | `TAATGACTAAG` |
| MA0478.1 | chr13 | − | 39917844 | 39917854 | 6.16e-05 | 0.345 | `GAATGACTAAC` |
| MA0478.1 | chr14 | + | 58698226 | 58698236 | 6.16e-05 | 0.345 | `GAATGACTAAC` |
| MA0478.1 | chr17 | + | 7423543 | 7423553 | 6.16e-05 | 0.345 | `TAATGACTAAG` |
| MA0478.1 | chr21 | + | 18075029 | 18075039 | 6.16e-05 | 0.345 | `TAATGACTAAG` |
| MA0478.1 | chr10 | + | 301104 | 301114 | 6.26e-05 | 0.345 | `GGTTGACTCAC` |
| MA0478.1 | chr10 | + | 301163 | 301173 | 6.26e-05 | 0.345 | `GGTTGACTCAC` |
| MA0478.1 | chr12 | − | 27265988 | 27265998 | 6.26e-05 | 0.345 | `TGTTGACTCAG` |
| MA0478.1 | chr1 | − | 29957519 | 29957529 | 6.35e-05 | 0.345 | `CAATGATTCAG` |
| MA0478.1 | chr1 | + | 110975596 | 110975606 | 6.35e-05 | 0.345 | `TGATGGCTCAC` |
| MA0478.1 | chrX | + | 46382547 | 46382557 | 6.35e-05 | 0.345 | `AAATGATTCAT` |
| MA0478.1 | chr10 | + | 38423423 | 38423433 | 6.35e-05 | 0.345 | `GGGTGAGTAAG` |
| MA0478.1 | chr11 | + | 33665960 | 33665970 | 6.35e-05 | 0.345 | `GGGTGAGTAAG` |
| MA0478.1 | chr19 | − | 55091208 | 55091218 | 6.35e-05 | 0.345 | `GGGTGAGTAAG` |
| MA0478.1 | chr2 | − | 122005275 | 122005285 | 6.4e-05 | 0.345 | `GGCTGACTCAG` |
| MA0478.1 | chr7 | − | 55568660 | 55568670 | 6.4e-05 | 0.345 | `GGCTGACTCAG` |
| MA0478.1 | chr8 | − | 129303557 | 129303567 | 6.4e-05 | 0.345 | `GGCTGACTCAG` |
| MA0478.1 | chr11 | + | 47386270 | 47386280 | 6.4e-05 | 0.345 | `GGCTGACTCAG` |
| MA0478.1 | chr19 | + | 44584026 | 44584036 | 6.4e-05 | 0.345 | `GGCTGACTCAG` |
| MA0478.1 | chr21 | + | 15516293 | 15516303 | 6.4e-05 | 0.345 | `GGCTGACTCAG` |
| MA0478.1 | chr1 | + | 224917490 | 224917500 | 6.46e-05 | 0.345 | `GATTGACTCAT` |
| MA0478.1 | chr6 | − | 133182605 | 133182615 | 6.46e-05 | 0.345 | `GGATGAGTAAC` |
| MA0478.1 | chr8 | + | 105768019 | 105768029 | 6.46e-05 | 0.345 | `TGATGAGTAAG` |
| MA0478.1 | chr11 | + | 72766002 | 72766012 | 6.46e-05 | 0.345 | `TGATGAGTAAG` |
| MA0478.1 | chr14 | + | 99602279 | 99602289 | 6.46e-05 | 0.345 | `GATTGACTCAT` |
| MA0478.1 | chr15 | + | 29567779 | 29567789 | 6.46e-05 | 0.345 | `GATTGACTCAT` |
| MA0478.1 | chr22 | − | 37207458 | 37207468 | 6.46e-05 | 0.345 | `GGATGAGTAAC` |
| MA0478.1 | chr2 | + | 105740010 | 105740020 | 6.51e-05 | 0.346 | `TAATGGCTCAT` |
| MA0478.1 | chr10 | − | 11327340 | 11327350 | 6.51e-05 | 0.346 | `TAATGGCTCAT` |
| MA0478.1 | chrX | − | 38545749 | 38545759 | 6.58e-05 | 0.349 | `AGATGGCTCAT` |
| MA0478.1 | chr1 | − | 149850906 | 149850916 | 6.62e-05 | 0.351 | `AAATGATTCAG` |
| MA0478.1 | chr8 | + | 141668252 | 141668262 | 6.69e-05 | 0.354 | `GAATGAGTAAT` |
| MA0478.1 | chr5 | − | 107746360 | 107746370 | 6.88e-05 | 0.36 | `GAATGGCTCAC` |
| MA0478.1 | chr3 | + | 10209789 | 10209799 | 6.92e-05 | 0.36 | `CAGTGATTCAT` |
| MA0478.1 | chr3 | − | 198923676 | 198923686 | 6.92e-05 | 0.36 | `CAGTGATTCAT` |
| MA0478.1 | chr2 | − | 86103931 | 86103941 | 6.96e-05 | 0.36 | `AGATGGCTCAG` |
| MA0478.1 | chr4 | − | 2908394 | 2908404 | 6.96e-05 | 0.36 | `AGATGGCTCAG` |
| MA0478.1 | chr14 | − | 99601905 | 99601915 | 6.96e-05 | 0.36 | `AGATGGCTCAG` |
| MA0478.1 | chr1 | − | 154453047 | 154453057 | 6.99e-05 | 0.36 | `GGGTGGGTCAG` |
| MA0478.1 | chr5 | + | 149764234 | 149764244 | 6.99e-05 | 0.36 | `GGGTGGGTCAG` |
| MA0478.1 | chr17 | − | 3811739 | 3811749 | 6.99e-05 | 0.36 | `GGGTGGGTCAG` |
| MA0478.1 | chr17 | − | 34278702 | 34278712 | 6.99e-05 | 0.36 | `GGGTGGGTCAG` |
| MA0478.1 | chr12 | + | 26343179 | 26343189 | 7.06e-05 | 0.363 | `AGGTGATTCAC` |
| MA0478.1 | chr8 | + | 72913968 | 72913978 | 7.15e-05 | 0.365 | `GGTTGAGTCAG` |
| MA0478.1 | chr10 | − | 112106407 | 112106417 | 7.15e-05 | 0.365 | `GGTTGAGTCAG` |
| MA0478.1 | chr22 | + | 35587297 | 35587307 | 7.15e-05 | 0.365 | `GGTTGAGTCAG` |
| MA0478.1 | chr7 | + | 120706229 | 120706239 | 7.27e-05 | 0.367 | `TGCTGACTCAT` |
| MA0478.1 | chr10 | − | 126879783 | 126879793 | 7.27e-05 | 0.367 | `TGCTGACTCAT` |
| MA0478.1 | chr11 | + | 1833007 | 1833017 | 7.27e-05 | 0.367 | `AGGTGACTAAG` |
| MA0478.1 | chr16 | − | 3161763 | 3161773 | 7.27e-05 | 0.367 | `TGCTGACTCAT` |
| MA0478.1 | chr22 | − | 49095898 | 49095908 | 7.27e-05 | 0.367 | `TGCTGACTCAT` |
| MA0478.1 | chr8 | + | 125003628 | 125003638 | 7.38e-05 | 0.37 | `TGGTGGCTCAC` |
| MA0478.1 | chr12 | − | 47807445 | 47807455 | 7.38e-05 | 0.37 | `TGGTGGCTCAC` |
| MA0478.1 | chr15 | − | 29342952 | 29342962 | 7.38e-05 | 0.37 | `GCATGATTCAG` |
| MA0478.1 | chr4 | + | 95123630 | 95123640 | 7.51e-05 | 0.371 | `GAATGGGTCAT` |
| MA0478.1 | chr5 | − | 6537059 | 6537069 | 7.51e-05 | 0.371 | `TGTTGACTCAC` |
| MA0478.1 | chr8 | + | 141670877 | 141670887 | 7.51e-05 | 0.371 | `AGATGACTAAC` |
| MA0478.1 | chr22 | − | 28027403 | 28027413 | 7.51e-05 | 0.371 | `GAATGGGTCAT` |
| MA0478.1 | chr11 | − | 103305488 | 103305498 | 7.58e-05 | 0.371 | `TGGTGAGTAAG` |
| MA0478.1 | chr3 | − | 109327469 | 109327479 | 7.6e-05 | 0.371 | `TAGTGGCTCAT` |
| MA0478.1 | chr14 | − | 34943888 | 34943898 | 7.6e-05 | 0.371 | `TAGTGGCTCAT` |
| MA0478.1 | chr1 | − | 158862660 | 158862670 | 7.73e-05 | 0.371 | `GGCTGACTCAC` |
| MA0478.1 | chr2 | + | 101207207 | 101207217 | 7.73e-05 | 0.371 | `AGGTGGCTCAT` |
| MA0478.1 | chr2 | − | 201689524 | 201689534 | 7.73e-05 | 0.371 | `TGCTGACTCAG` |
| MA0478.1 | chr4 | + | 39905424 | 39905434 | 7.73e-05 | 0.371 | `TGCTGACTCAG` |
| MA0478.1 | chr9 | − | 116151310 | 116151320 | 7.73e-05 | 0.371 | `GGCTGACTCAC` |
| MA0478.1 | chr10 | + | 71164925 | 71164935 | 7.73e-05 | 0.371 | `AGGTGGCTCAT` |
| MA0478.1 | chr10 | + | 112117880 | 112117890 | 7.73e-05 | 0.371 | `GGCTGACTCAC` |
| MA0478.1 | chr12 | + | 93478978 | 93478988 | 7.73e-05 | 0.371 | `TGCTGACTCAG` |
| MA0478.1 | chr16 | + | 11330068 | 11330078 | 7.73e-05 | 0.371 | `GGCTGACTCAC` |
| MA0478.1 | chr17 | − | 63799940 | 63799950 | 7.73e-05 | 0.371 | `TGCTGACTCAG` |
| MA0478.1 | chr19 | − | 33312882 | 33312892 | 7.73e-05 | 0.371 | `AGGTGGCTCAT` |
| MA0478.1 | chr20 | + | 33753638 | 33753648 | 7.73e-05 | 0.371 | `TGCTGACTCAG` |
| MA0478.1 | chr7 | − | 55569669 | 55569679 | 7.83e-05 | 0.374 | `AAATGACTAAT` |
| MA0478.1 | chr21 | + | 34269965 | 34269975 | 7.83e-05 | 0.374 | `AAATGACTAAT` |
| MA0478.1 | chr5 | − | 95245640 | 95245650 | 7.87e-05 | 0.375 | `GAGTGAGTAAT` |
| MA0478.1 | chr9 | − | 85942778 | 85942788 | 7.87e-05 | 0.375 | `GTATGATTCAG` |
| MA0478.1 | chr4 | + | 2908515 | 2908525 | 8.03e-05 | 0.38 | `GACTGACTCAT` |
| MA0478.1 | chr7 | + | 86941902 | 86941912 | 8.03e-05 | 0.38 | `AAATGATTCAC` |
| MA0478.1 | chr14 | − | 96843197 | 96843207 | 8.03e-05 | 0.38 | `AAATGATTCAC` |
| MA0478.1 | chr5 | + | 106934896 | 106934906 | 8.1e-05 | 0.38 | `TAGTGGCTCAG` |
| MA0478.1 | chr8 | + | 141670755 | 141670765 | 8.1e-05 | 0.38 | `TAATGAGTAAT` |
| MA0478.1 | chr12 | − | 55014448 | 55014458 | 8.1e-05 | 0.38 | `GAGTGGCTCAC` |
| MA0478.1 | chr14 | + | 69303728 | 69303738 | 8.1e-05 | 0.38 | `GAGTGGCTCAC` |
| MA0478.1 | chr11 | − | 59075023 | 59075033 | 8.13e-05 | 0.38 | `GCGTGATTCAT` |
| MA0478.1 | chr11 | + | 47386168 | 47386178 | 8.22e-05 | 0.383 | `CGATGAGTAAG` |
| MA0478.1 | chr13 | + | 41850096 | 41850106 | 8.22e-05 | 0.383 | `AGATGAGTAAT` |
| MA0478.1 | chr14 | − | 64838918 | 64838928 | 8.3e-05 | 0.383 | `CAATGGCTCAT` |
| MA0478.1 | chr16 | − | 11707306 | 11707316 | 8.3e-05 | 0.383 | `AAATGACTAAG` |
| MA0478.1 | chr17 | − | 23408964 | 23408974 | 8.3e-05 | 0.383 | `CAATGGCTCAT` |
| MA0478.1 | chr8 | − | 123864924 | 123864934 | 8.37e-05 | 0.383 | `GAGTGAGTAAG` |
| MA0478.1 | chr1 | − | 180662936 | 180662946 | 8.42e-05 | 0.383 | `AGTTGACTCAG` |
| MA0478.1 | chr8 | + | 10860748 | 10860758 | 8.42e-05 | 0.383 | `TCATGATTCAT` |
| MA0478.1 | chr3 | + | 53111876 | 53111886 | 8.46e-05 | 0.383 | `GGCTGAGTCAT` |
| MA0478.1 | chr7 | + | 42875700 | 42875710 | 8.46e-05 | 0.383 | `GGCTGAGTCAT` |
| MA0478.1 | chr14 | + | 93495646 | 93495656 | 8.46e-05 | 0.383 | `GGCTGAGTCAT` |
| MA0478.1 | chr14 | − | 105119555 | 105119565 | 8.46e-05 | 0.383 | `GGCTGAGTCAT` |
| MA0478.1 | chr14 | − | 105238484 | 105238494 | 8.46e-05 | 0.383 | `GGCTGAGTCAT` |
| MA0478.1 | chr16 | − | 11967852 | 11967862 | 8.46e-05 | 0.383 | `AGATGGCTCAC` |
| MA0478.1 | chr21 | + | 33373380 | 33373390 | 8.46e-05 | 0.383 | `GGCTGAGTCAT` |
| MA0478.1 | chr2 | + | 33558149 | 33558159 | 8.54e-05 | 0.383 | `GGGTGGGTCAC` |
| MA0478.1 | chr5 | − | 33886017 | 33886027 | 8.54e-05 | 0.383 | `GGGTGGGTCAC` |
| MA0478.1 | chr7 | + | 134504599 | 134504609 | 8.54e-05 | 0.383 | `GACTGACTCAG` |
| MA0478.1 | chr14 | − | 61199219 | 61199229 | 8.54e-05 | 0.383 | `GGGTGGGTCAC` |
| MA0478.1 | chr14 | − | 80991054 | 80991064 | 8.54e-05 | 0.383 | `TGGTGGGTCAG` |
| MA0478.1 | chr8 | − | 126233060 | 126233070 | 8.58e-05 | 0.384 | `GAATGAGTAAC` |
| MA0478.1 | chr7 | − | 139262949 | 139262959 | 8.65e-05 | 0.386 | `CAGTGACTAAT` |
| MA0478.1 | chr16 | − | 23254737 | 23254747 | 8.65e-05 | 0.386 | `GCATGACTAAT` |
| MA0478.1 | chr2 | + | 114360619 | 114360629 | 8.79e-05 | 0.389 | `AGATGAGTAAG` |
| MA0478.1 | chr3 | + | 151298151 | 151298161 | 8.79e-05 | 0.389 | `AGATGAGTAAG` |
| MA0478.1 | chr9 | + | 129883396 | 129883406 | 8.79e-05 | 0.389 | `TGTTGAGTCAG` |
| MA0478.1 | chr10 | + | 112118029 | 112118039 | 8.79e-05 | 0.389 | `GTGTGATTCAT` |
| MA0478.1 | chr19 | + | 13139412 | 13139422 | 8.88e-05 | 0.392 | `AAATGGCTCAT` |
| MA0478.1 | chr3 | − | 48909417 | 48909427 | 8.93e-05 | 0.393 | `GAGTGGGTCAT` |
| MA0478.1 | chr6 | − | 250694 | 250704 | 9e-05 | 0.393 | `CAGTGATTCAC` |
| MA0478.1 | chr16 | − | 11253535 | 11253545 | 9e-05 | 0.393 | `TCATGATTCAG` |
| MA0478.1 | chr8 | + | 130634116 | 130634126 | 9.04e-05 | 0.393 | `TTATGATTCAT` |
| MA0478.1 | chr12 | − | 93478980 | 93478990 | 9.04e-05 | 0.393 | `GGCTGAGTCAG` |
| MA0478.1 | chr17 | + | 63799938 | 63799948 | 9.04e-05 | 0.393 | `GGCTGAGTCAG` |
| MA0478.1 | chr19 | − | 44584028 | 44584038 | 9.04e-05 | 0.393 | `GGCTGAGTCAG` |
| MA0478.1 | chr3 | − | 109589907 | 109589917 | 9.19e-05 | 0.398 | `AAGTGACTAAT` |
| MA0478.1 | chr17 | − | 25714994 | 25715004 | 9.19e-05 | 0.398 | `CAGTGACTAAG` |
| MA0478.1 | chr6 | − | 31654438 | 31654448 | 9.29e-05 | 0.398 | `GTGTGATTCAG` |
| MA0478.1 | chr6 | + | 138070709 | 138070719 | 9.29e-05 | 0.398 | `AGATGGGTCAT` |
| MA0478.1 | chr10 | + | 51941854 | 51941864 | 9.29e-05 | 0.398 | `AGATGGGTCAT` |
| MA0478.1 | chr11 | − | 127844537 | 127844547 | 9.29e-05 | 0.398 | `AGATGGGTCAT` |
| MA0478.1 | chr17 | + | 44656359 | 44656369 | 9.29e-05 | 0.398 | `CGATGGGTCAG` |
| MA0478.1 | chr16 | + | 56135747 | 56135757 | 9.36e-05 | 0.401 | `AAATGGCTCAG` |
| MA0478.1 | chr3 | − | 49257469 | 49257479 | 9.44e-05 | 0.403 | `TGCTGACTCAC` |
| MA0478.1 | chr17 | + | 30414911 | 30414921 | 9.44e-05 | 0.403 | `TGCTGACTCAC` |
| MA0478.1 | chr7 | − | 134505134 | 134505144 | 9.54e-05 | 0.406 | `TTATGATTCAG` |
| MA0478.1 | chrX | − | 141893685 | 141893695 | 9.68e-05 | 0.411 | `AGGTGAGTAAT` |
| MA0478.1 | chr7 | − | 130295864 | 130295874 | 9.77e-05 | 0.412 | `AAGTGACTAAG` |
| MA0478.1 | chr8 | − | 142198279 | 142198289 | 9.77e-05 | 0.412 | `CAGTGGCTCAT` |
| MA0478.1 | chr14 | + | 95031919 | 95031929 | 9.77e-05 | 0.412 | `CAGTGGCTCAT` |
| MA0478.1 | chr15 | − | 29567749 | 29567759 | 9.77e-05 | 0.412 | `CAGTGGCTCAT` |
| MA0478.1 | chrX | + | 46373282 | 46373292 | 9.84e-05 | 0.413 | `TAGTGGCTCAC` |
| MA0478.1 | chr17 | − | 34278808 | 34278818 | 9.84e-05 | 0.413 | `AGATGGGTCAG` |
| MA0478.1 | chr7 | − | 126128454 | 126128464 | 9.91e-05 | 0.414 | `AGCTGACTCAT` |
| MA0478.1 | chr10 | − | 93851198 | 93851208 | 9.91e-05 | 0.414 | `AGCTGACTCAT` |
| MA0478.1 | chr12 | − | 120810851 | 120810861 | 9.91e-05 | 0.414 | `AGCTGACTCAT` |
| MA0478.1 | chr2 | − | 191533158 | 191533168 | 9.98e-05 | 0.415 | `CATTGACTCAT` |
| MA0478.1 | chr2 | − | 232244765 | 232244775 | 9.98e-05 | 0.415 | `AGGTGGCTCAC` |
| MA0478.1 | chr12 | − | 37455712 | 37455722 | 9.98e-05 | 0.415 | `CATTGACTCAT` |

---

**DEBUGGING INFORMATION**


---

Command line:

```
/ebi/sw/MEME/VM-cluster410/meme-versions/4.10.0/bin/fimo --parse-genomic-coord --verbosity 1 --oc fimo_out_16 --bgfile ./background --motif MA0478.1 db/JASPAR_CORE_2014_vertebrates.meme ./Supplementary_Table_1.500bp.fa
```

Settings:

```
|  |  |  |
| --- | --- | --- |
| output directory = fimo_out_16 | MEME file name = db/JASPAR_CORE_2014_vertebrates.meme | sequence file name = ./Supplementary_Table_1.500bp.fa |
| background file name = ./background | allow clobber = true | compute q-values = true |
| parse genomic coord. = true | text only = false | scan both strands = true |
| max sequence length = 250000000 | output threshold = 0.0001 | threshold type = p-value |
| max stored scores = 100000 | pseudocount = 0.1 | verbosity = 1 |
| selected motif = MA0478.1 |  |  |
```

This information can be useful in the event you wish to report a
problem with the FIMO software.

---

**Go to top**
