# Supplementary material for: The MEME Suite
Source: Nucleic Acids Res. 2015 May 7;43(Web Server issue):W39–49. doi: 10.1093/nar/gkv416 (PMC4489269; doi:10.1093/nar/gkv416)
Supplement: SUPPLEMENTARY DATA [file supp_gkv416_nar-00283-web-b-2015-File005.zip › case4/meme-chip/fimo_out_17/fimo.html]

FIMO Results


---

|  |  |  |
| --- | --- | --- |
| **Database and Motifs** | **High-scoring Motif Occurrences** | **Debugging Information** |

  
  

---

**FIMO - Motif search tool**


---

FIMO version 4.10.0,
(Release date: Wed May 21 10:35:36 2014 +1000)

For further information on how to interpret these results
or to get a copy of the FIMO software please access
http://meme.nbcr.net

If you use FIMO in your research, please cite the following paper:  
Charles E. Grant, Timothy L. Bailey, and William Stafford Noble,
"FIMO: Scanning for occurrences of a given motif",
*Bioinformatics*, **27**(7):1017-1018, 2011.
[full text]

---

**DATABASE AND MOTIFS**


---

DATABASE
./Supplementary\_Table\_1.500bp.fa  
Database contains
2776
sequences,
1388000
residues

MOTIFS
db/uniprobe\_mouse.meme
(nucleotide)

| MOTIF | WIDTH | BEST POSSIBLE MATCH |
| --- | --- | --- |
| UP00078\_1 | 17 | GGGTTTAATTAAAATTC |
| UP00059\_1 | 14 | CTAATATTGCTAAA |
| UP00099\_1 | 17 | CTCAGCAGCTGCTCCTG |
| UP00020\_1 | 16 | ACGATGACGTCATCGA |
| UP00012\_1 | 15 | TAATTCAATGAAGTG |
| UP00043\_1 | 16 | TCTTTCGAGGAATTTG |
| UP00050\_1 | 22 | GGAAGAGTCACGTGACCAATAC |
| UP00001\_1 | 15 | ATAAAGGCGCGCGAT |
| UP00003\_1 | 15 | ATAAGGGCGCGCGAT |
| UP00007\_1 | 14 | TCCGCCCCCGCATT |
| UP00015\_1 | 15 | AGGACCCGGAAGTAA |
| UP00407\_1 | 13 | TACAAGGAAGTAA |
| UP00068\_1 | 17 | TAAAAGGTGTGAAAATT |
| UP00079\_1 | 17 | TATTCAAGGTCATGCGA |
| UP00073\_1 | 17 | AAAAAGTAAACAAAGAC |
| UP00041\_1 | 16 | AAAGTAAACAAAAATT |
| UP00039\_1 | 17 | AAAAAGTAAACAAACCC |
| UP00025\_1 | 17 | AAAATGTAAACAAACAG |
| UP00061\_1 | 17 | TAAATGTAAACAAAGGT |
| UP00408\_1 | 17 | CAATACCGGAAGTGTAA |
| UP00032\_1 | 22 | TTTTTAGAGATAAGAAATAAAG |
| UP00080\_1 | 17 | TAAACTGATAAGAAGAT |
| UP00100\_1 | 17 | TATAGAGATAAGAATTG |
| UP00070\_1 | 16 | TCGTACCCGCATCATT |
| UP00024\_1 | 16 | TATCGACCCCCCACAG |
| UP00042\_1 | 17 | CAGATGTGCACATACGT |
| UP00084\_1 | 17 | GAGTGTACGTACGATGG |
| UP00055\_1 | 16 | ACTATGAATGAATGAT |
| UP00035\_1 | 16 | ACTATGCCAACCTACC |
| UP00066\_1 | 17 | CTTCAGGGGTCAATTGA |
| UP00391\_1 | 14 | TGGAGGTAATTAAC |
| UP00072\_1 | 16 | ATTTACGACAAATAGC |
| UP00086\_1 | 14 | GAGAACCGAAACTG |
| UP00018\_1 | 15 | CGTATCGAAACCAAA |
| UP00040\_1 | 15 | ATAAACCGAAACCAA |
| UP00011\_1 | 17 | CTGATCGAAACCAAAGT |
| UP00074\_1 | 15 | CAAAATCGAAACTAA |
| UP00103\_1 | 16 | CCGATGACGTCATCGT |
| UP00093\_1 | 16 | TCGACCCCGCCCCTAT |
| UP00067\_1 | 17 | AATCCCTTTGATCTATC |
| UP00045\_1 | 17 | AAATTTGCTGACTTAGC |
| UP00044\_1 | 15 | TAAAAATGCTGACTT |
| UP00060\_1 | 16 | TGACCACGTGGTCGGG |
| UP00097\_1 | 16 | GGGCCGTGTGCAAAAA |
| UP00092\_1 | 17 | ATGGAAACCGTTATTTT |
| UP00081\_1 | 17 | TTGAAAACCGTTAATTT |
| UP00036\_1 | 16 | GAAGAACAGGTGTCCG |
| UP00017\_1 | 17 | CTTAACCACTTAAGGAT |
| UP00009\_1 | 16 | TCTCAAAGGTCACGAG |
| UP00027\_1 | 16 | TTTTACAGTAGCAAAA |
| UP00052\_1 | 16 | ATGTACAGTAGCAAAG |
| UP00088\_1 | 16 | TTGGGGGCGCCCCTAG |
| UP00048\_1 | 16 | TCTCAAAGGTCACCTG |
| UP00098\_1 | 23 | TGTGACCCTTAGCAACCGATTAA |
| UP00056\_1 | 15 | TACCATAGCAACGGT |
| UP00076\_1 | 15 | CCGCATAGCAACGGA |
| UP00053\_1 | 17 | TGTCGTGACCCCTTAAT |
| UP00085\_1 | 14 | TTAAGAGGAAGTTA |
| UP00008\_1 | 17 | AATAGGGTATCATATAT |
| UP00000\_1 | 17 | CAAATCCAGACATCAGA |
| UP00030\_1 | 17 | ATAAGAACAAAGGACTA |
| UP00101\_1 | 14 | TAATTGTTCTAAAC |
| UP00096\_1 | 16 | TTAAGAACAATAATTT |
| UP00004\_1 | 16 | GCTAATTATAATTATC |
| UP00075\_1 | 17 | TAGTGAACAATAGATTT |
| UP00014\_1 | 15 | ATAAACAATTAATCA |
| UP00064\_1 | 16 | TTCAATTGTTCTAAAA |
| UP00069\_1 | 16 | AATCAATTCAATAATT |
| UP00071\_1 | 16 | TTTAATTATAATTAAG |
| UP00023\_1 | 16 | ATTGAACAATGGAATT |
| UP00062\_1 | 17 | AGAAGAACAAAGGACTA |
| UP00091\_1 | 16 | TTTAGAACAATAAAAT |
| UP00034\_1 | 22 | AATAAAGAACAATAGAATTTCA |
| UP00051\_1 | 17 | TTATCTATTGTTCTTTA |
| UP00049\_1 | 14 | ATTTTACGGAAAAT |
| UP00002\_1 | 17 | GGTCCCGCCCCCTTCTC |
| UP00406\_1 | 16 | GTACATCCGGATTTTT |
| UP00077\_1 | 14 | TTCCATATATGGAA |
| UP00016\_1 | 16 | TATAATTATAATATTC |
| UP00029\_1 | 16 | TCTTTATATATAAATA |
| UP00089\_1 | 17 | ACTTAGTTAACTAAAAA |
| UP00058\_1 | 17 | TATAGATCAAAGGAAAA |
| UP00054\_1 | 17 | TATAGATCAAAGGAAAA |
| UP00083\_1 | 17 | ATTTCCTTTGATCTATA |
| UP00005\_1 | 15 | ATTCCCTGAGGGGAA |
| UP00010\_1 | 14 | TTGCCCTAGGGCAT |
| UP00087\_1 | 15 | ATTGCCTGAGGCGAA |
| UP00028\_1 | 15 | ATTGCCTGAGGCGAT |
| UP00046\_1 | 17 | ATCCACAGGTGCGAAAA |
| UP00019\_1 | 17 | CTAAGGTTCTAGATCAC |
| UP00031\_1 | 17 | AATCGCACTGCATTCCG |
| UP00047\_1 | 15 | AAGCCCCCCAAAAAT |
| UP00037\_1 | 15 | AACAAACAACAAGAG |
| UP00094\_1 | 17 | TCTTTGGCGTACCCTAA |
| UP00065\_1 | 16 | TGGCGCGCGCGCCTGA |
| UP00082\_1 | 14 | TTATGTACTAATAA |
| UP00021\_1 | 15 | TCCCCCCCCCCCCCC |
| UP00033\_1 | 17 | TATTATGGGATGGATAA |
| UP00095\_1 | 17 | CGAACAGTGCTCACTAT |
| UP00022\_1 | 16 | CCCCCCCCCCCACTTG |
| UP00102\_1 | 14 | CACCCCCGGGGGGG |
| UP00057\_1 | 15 | CCCCCCCGGGGGGGT |
| UP00006\_1 | 15 | CCCCCCCGGGGGGGT |
| UP00026\_1 | 17 | TACATGTGCACATAAAA |
| UP00078\_2 | 15 | ACCCGTATCAAATTT |
| UP00059\_2 | 17 | CGTACAATACGAAATAA |
| UP00099\_2 | 16 | CTATCCCCGCCCTATT |
| UP00020\_2 | 14 | GAATGACGAATAAC |
| UP00012\_2 | 17 | TGATTGTTAACAGTTGG |
| UP00043\_2 | 16 | ATCCCCGCCCCTAAAA |
| UP00050\_2 | 23 | TGTCGTTACACGTGGAAGGCGGT |
| UP00001\_2 | 17 | CGTTCGGCGCCAAAAGG |
| UP00003\_2 | 17 | CGCTCGGCGCCAAAAGC |
| UP00007\_2 | 16 | TGCGGAGTGGGACTGG |
| UP00015\_2 | 16 | TAGTATTTCCGATCTT |
| UP00407\_2 | 17 | GTTCAAAAAAAAAATTC |
| UP00068\_2 | 16 | GCGGAGGTGTCGCCTC |
| UP00079\_2 | 17 | GGCGAGGGGTCAAGGGC |
| UP00073\_2 | 15 | AAAAATAACAAACGG |
| UP00041\_2 | 15 | ATGTCACAACAACAC |
| UP00039\_2 | 17 | AACACCAAAACAAAGGA |
| UP00025\_2 | 15 | CAAACAACAACACCT |
| UP00061\_2 | 16 | ATATCAAAACAAAACA |
| UP00408\_2 | 16 | CCGTCTTCCCCCTCAC |
| UP00032\_2 | 22 | TTTTGTAGATTTTATCGACTTA |
| UP00080\_2 | 17 | GACAGAGATATCAGTTT |
| UP00100\_2 | 17 | GCGGCGATATCGCAGCG |
| UP00070\_2 | 17 | TGCGCATAGGGGAGGAG |
| UP00024\_2 | 14 | AATATTAATAAAGA |
| UP00042\_2 | 16 | AGCGGCACACACGCAA |
| UP00084\_2 | 16 | TGGGCGACGTCGTTAA |
| UP00055\_2 | 17 | TGTTCCCATTGTGTACT |
| UP00035\_2 | 16 | GGGTGTGCCCAAAAGG |
| UP00066\_2 | 16 | TGCAAAAGTCCAATAT |
| UP00391\_2 | 14 | AAAAACCATTAAGG |
| UP00072\_2 | 16 | ATGGAAAGTCGTAAAA |
| UP00086\_2 | 14 | GGAGAAAGGTGCGA |
| UP00018\_2 | 15 | AGTATTCTCGGTTGC |
| UP00040\_2 | 15 | TTGATCGAGAATTCC |
| UP00011\_2 | 15 | ACCACTCTCGGTCAC |
| UP00074\_2 | 14 | GCAAAACATTACTA |
| UP00103\_2 | 16 | ATTGATGAGTCACCAA |
| UP00093\_2 | 17 | AAGCATACGCCCAACTT |
| UP00067\_2 | 16 | GAAGATCAATCACTTA |
| UP00045\_2 | 15 | CAATTGCAAAAATAT |
| UP00044\_2 | 15 | GAAAAAATTGCAAGG |
| UP00060\_2 | 14 | GTGCCACGCGACTG |
| UP00097\_2 | 14 | AAATAAGAAAAAAC |
| UP00092\_2 | 16 | CGACCAACTGCCATGC |
| UP00081\_2 | 15 | CGACCAACTGCCGTG |
| UP00036\_2 | 15 | AGCAACAGCCGCACC |
| UP00017\_2 | 17 | ACTCCAAGTACTTGGAA |
| UP00009\_2 | 16 | CGCGCCGGGTCACGTA |
| UP00027\_2 | 16 | ACATGCTACCTAATAC |
| UP00052\_2 | 16 | ACTTGCTACCTACACC |
| UP00088\_2 | 17 | GCTGGGGGGTACCCCTT |
| UP00048\_2 | 16 | AGAGCGGGGTCAAGTA |
| UP00098\_2 | 23 | ACTGACGCTTGGTTACCACAAAG |
| UP00056\_2 | 15 | TACCCTAGTTACCGA |
| UP00076\_2 | 17 | CTACTTGGATACGGAAT |
| UP00053\_2 | 16 | TCGCGAAGGTTGTACT |
| UP00085\_2 | 14 | CAAATTCCGGAACC |
| UP00008\_2 | 17 | ATGGGATATATCCGCCT |
| UP00000\_2 | 17 | TACGCCCCGCCACTCTG |
| UP00030\_2 | 14 | AAAATTGTTATGAA |
| UP00101\_2 | 16 | AAATAGACAAAGGAAT |
| UP00096\_2 | 17 | GTATTGGGTGGGTATTT |
| UP00004\_2 | 15 | CTCACACAATGGCGC |
| UP00075\_2 | 15 | TTGAATGAAATTCGA |
| UP00014\_2 | 17 | GACCACATTCATACAAT |
| UP00064\_2 | 16 | GGACTGAATTCATGCC |
| UP00069\_2 | 15 | CTATAATTGTTATCG |
| UP00071\_2 | 17 | CATCAATTGTTCCGCTA |
| UP00023\_2 | 16 | TAAGATTATAATACGG |
| UP00062\_2 | 17 | GGAAAAATTGTTAGGAA |
| UP00091\_2 | 15 | TATCATAATTAAGGA |
| UP00034\_2 | 22 | GTGCTAATTGTGTGTGTACGCT |
| UP00051\_2 | 14 | ACATTCATGACACG |
| UP00049\_2 | 15 | TCCGTCGCTTAAAAG |
| UP00002\_2 | 15 | CAAAGGCGTGGCCAG |
| UP00406\_2 | 16 | GATAACATCCTAGTAG |
| UP00077\_2 | 17 | GTTAAAAAAAAAAATTT |
| UP00016\_2 | 17 | TCACGGAACAATAGGTG |
| UP00029\_2 | 15 | CCGATTTAAGCGATC |
| UP00089\_2 | 14 | TTGCCCGGATTAGG |
| UP00058\_2 | 15 | AGCCGAAAAAAAAAT |
| UP00054\_2 | 15 | CCGTATTATAAACAA |
| UP00083\_2 | 16 | GAAGATCAATCACTAA |
| UP00005\_2 | 14 | TCACCTCTGGGCAG |
| UP00010\_2 | 15 | ATTGCCTCAGGCAAT |
| UP00087\_2 | 14 | CCGCCCAAGGGCAG |
| UP00028\_2 | 14 | TACTGGAAAAAAAA |
| UP00046\_2 | 17 | AAGGCCAGATGGTCCGG |
| UP00019\_2 | 15 | TATCATTAGAACGCT |
| UP00031\_2 | 16 | CAATCACTGGCAGAAT |
| UP00047\_2 | 17 | CTTAAGACCACCATTAC |
| UP00037\_2 | 17 | GTGGTTCAATAATTTTG |
| UP00094\_2 | 14 | TGTATATATATACC |
| UP00065\_2 | 14 | GCCGCGCAGTGCGT |
| UP00082\_2 | 16 | GAGCCCTTGTCCCTTG |
| UP00021\_2 | 17 | AGGAGACCCCCAATTTG |
| UP00033\_2 | 17 | TCACCCCGCCCCTAATT |
| UP00095\_2 | 17 | TACGAGACTCCTCTAAC |
| UP00022\_2 | 17 | AAATTCCCCCCGGAAGT |
| UP00102\_2 | 15 | CCACACAGCAGGAGA |
| UP00057\_2 | 15 | CCACACAGCAGGAGA |
| UP00006\_2 | 15 | GAGCACAGCAGGACA |
| UP00026\_2 | 16 | CGAAGCACACAAAATA |
| UP00108\_1 | 17 | TAAACTAATTAGCTGAG |
| UP00187\_1 | 17 | CGCATTAATTAATTACC |
| UP00152\_1 | 17 | GTCCATTAATTAATGGA |
| UP00228\_1 | 17 | CATAACCACTTAACAAC |
| UP00166\_1 | 16 | AACAACCAATTAATTC |
| UP00145\_1 | 16 | AAAAACCAATTAAGAA |
| UP00181\_1 | 16 | AAAGTAATTAGTGAAT |
| UP00151\_1 | 16 | TAAGTAATTAGTTATA |
| UP00138\_1 | 16 | CAGGTAATTACCTCAG |
| UP00209\_1 | 17 | CGAATTAATTAATCACC |
| UP00209\_2 | 17 | CGCATTAATTAATTGGC |
| UP00240\_1 | 16 | TAAGGTAATAAAATTA |
| UP00133\_1 | 16 | AACGGTAATAAAATTT |
| UP00198\_1 | 14 | ATGATCGAATCAAA |
| UP00176\_1 | 16 | CGTTGGGGATTAGCCT |
| UP00219\_1 | 17 | ACCGGTTGATCACCTGA |
| UP00219\_2 | 15 | TAATGATGATCACTA |
| UP00255\_1 | 17 | TAATTAATTAATAATTA |
| UP00218\_1 | 16 | TTTAATTAATTAATTC |
| UP00202\_1 | 14 | CTGAGGTAATTAAT |
| UP00126\_1 | 16 | GGAATAATTACTTCAG |
| UP00154\_1 | 17 | TCGCGATAATTACCGAC |
| UP00110\_1 | 17 | TCGCTATAATTACCGAC |
| UP00230\_1 | 16 | GGGGTAATTAGCTCTG |
| UP00111\_1 | 17 | TGAACCGGATTAATGAA |
| UP00232\_1 | 17 | TAAATAGATACCCCATA |
| UP00143\_1 | 17 | GGAAGGGATTAATTATC |
| UP00227\_1 | 17 | CGACCCAATCAACGGTG |
| UP00201\_1 | 17 | ACCACTAATTAGTGGAC |
| UP00167\_1 | 16 | GCGAACTAATTAATGC |
| UP00163\_1 | 17 | TGCACTAATTAGTGGAA |
| UP00251\_1 | 17 | ATCCATTAATTAATTGA |
| UP00162\_1 | 17 | AGAACTAATTAGTGGAC |
| UP00132\_1 | 17 | CACCGCTAATTAGCGTT |
| UP00204\_1 | 17 | TGCCACTAATTAGTGTA |
| UP00131\_1 | 17 | AGCGCTAATTAGCGATT |
| UP00112\_1 | 17 | AATCGTTAATCCCTTTA |
| UP00127\_1 | 16 | AGGTTAATTAGCTGAT |
| UP00148\_1 | 17 | AAGGCGAAATCATCGCA |
| UP00225\_1 | 15 | CCATAATTAATTACA |
| UP00123\_1 | 16 | GTACTAATTAGTGGCG |
| UP00161\_1 | 17 | GAAAACTAGTTAACATC |
| UP00104\_1 | 17 | ACAAGCAATTAATGAAT |
| UP00155\_1 | 17 | ACAAGCAATTAAAGAAT |
| UP00157\_1 | 17 | ACAAGCAATTAAAGAAT |
| UP00114\_1 | 17 | AAAACATCGTTTTTAAG |
| UP00264\_1 | 16 | CTGAGCTAATTACCGT |
| UP00217\_1 | 16 | TAGGTAATAAAATTCA |
| UP00246\_1 | 16 | TAAAGTCGTAAAACAT |
| UP00183\_1 | 16 | AAAGCTCGTAAAATTT |
| UP00174\_1 | 16 | AAGGTAATTAGCTCAT |
| UP00391\_3 | 14 | TTGAGGTAATTAGT |
| UP00196\_1 | 17 | GATTATTAATTAACTTG |
| UP00189\_1 | 16 | ACGGTAATTAGCTCAG |
| UP00182\_1 | 16 | AAGGTAATTACCTAAT |
| UP00164\_1 | 17 | CGAGTTAATTAATAAGC |
| UP00164\_2 | 16 | GTAGTAATTAATGGAA |
| UP00213\_1 | 17 | ACGGCCATAAAATTAAT |
| UP00134\_1 | 16 | AACCCAATAAAATTCG |
| UP00137\_1 | 17 | TGAGCTAATTAGTTGGA |
| UP00144\_1 | 17 | CGCGTTAATTAATTACC |
| UP00214\_1 | 16 | ACGGTAATTAGCTCAT |
| UP00259\_1 | 16 | TATTGGTAATTACCTT |
| UP00206\_1 | 16 | GTAGTAATTAATGCAA |
| UP00263\_1 | 16 | ACCGGCAATTAATAAA |
| UP00207\_1 | 16 | GGAGCCATAAAATTCG |
| UP00245\_1 | 16 | TAAAGTCGTAAAACGT |
| UP00235\_1 | 16 | TAAAGTCGTAAAATAG |
| UP00135\_1 | 17 | TTAGGTCGTAAAATTTC |
| UP00173\_1 | 16 | AAAGCTCGTAAAATTT |
| UP00113\_1 | 17 | CGAATTAATTAACAATA |
| UP00252\_1 | 17 | CGAATTAATTAATTACT |
| UP00260\_1 | 17 | CAAATTAATTAATAAAA |
| UP00242\_1 | 16 | TTGGGGTAATTAACGT |
| UP00197\_1 | 16 | GGAGGTCATTAATTAT |
| UP00140\_1 | 17 | TAAACTAATTAGCTGTA |
| UP00121\_1 | 17 | AATGCAATAAAATTTAT |
| UP00117\_1 | 17 | TAAGGTCGTAAAATCCT |
| UP00177\_1 | 17 | CAAGGTCGTAAAATCTT |
| UP00180\_1 | 16 | CTACCAATAAAATTCT |
| UP00241\_1 | 16 | TTGAGTTAATTAACCT |
| UP00168\_1 | 17 | TAATTAATTAATGGCTA |
| UP00124\_1 | 16 | AAGGTAATTAGCTCAT |
| UP00236\_1 | 17 | TAAATACATGTAAAATT |
| UP00223\_1 | 17 | AAAATACATGTAATACT |
| UP00223\_2 | 17 | AATATACATGTAATATT |
| UP00194\_1 | 17 | AATATACATGTAAAACA |
| UP00250\_1 | 17 | TATATACATGTAAAATT |
| UP00150\_1 | 17 | AAAATACATGTAAAAAT |
| UP00170\_1 | 16 | CAAAATCAATTAATTT |
| UP00243\_1 | 16 | ACTCCTAATTAGTCGT |
| UP00120\_1 | 17 | TGCATTAATTAATGCGA |
| UP00262\_1 | 17 | CGAATTAATTAATAATG |
| UP00115\_1 | 17 | TAAACTAATTAGTGAAC |
| UP00130\_1 | 17 | GTAATTAATTAAATAAT |
| UP00261\_1 | 17 | TAAACTAATTAGCTTTG |
| UP00212\_1 | 17 | CGAATTAATTAAATACT |
| UP00256\_1 | 17 | GAGCGTTAATTAATGTA |
| UP00256\_2 | 17 | TCCACTAATTAGCGGTT |
| UP00184\_1 | 17 | ACCCCTAATTAGCGGTG |
| UP00175\_1 | 17 | CCCATTAATTAATCACC |
| UP00188\_1 | 17 | CGAATTAATTAAAAACC |
| UP00169\_1 | 17 | AGTTTTTAATTAATTTG |
| UP00186\_1 | 16 | AAGGAGCTGTCAATAC |
| UP00233\_1 | 16 | GAGGTAATTACCTCAG |
| UP00226\_1 | 16 | AAAGACCTGTCAATAC |
| UP00210\_1 | 16 | AATTACCTGTCAATAC |
| UP00234\_1 | 16 | TGCAACTAATTAATTC |
| UP00156\_1 | 17 | GAAGACCAATTAGCGCT |
| UP00171\_1 | 16 | CAAAACCAATTAATTT |
| UP00220\_1 | 17 | TGCGCTAATTAGTGGGA |
| UP00139\_1 | 17 | GTGCACTAATTAGTGCA |
| UP00231\_1 | 17 | TTAACCACTTGAAAATT |
| UP00190\_1 | 16 | CTTTAAGTACTTAATG |
| UP00107\_1 | 16 | TAAGCCACTTGAAATT |
| UP00249\_1 | 16 | TAAGCCACTTGAATTT |
| UP00147\_1 | 16 | TAAGCCACTTAACATT |
| UP00119\_1 | 17 | TTTTAAGTACTTAAATT |
| UP00017\_3 | 17 | TACTAAGTACTTAAATG |
| UP00200\_1 | 17 | GAAAATTAATTACTTCG |
| UP00200\_2 | 16 | AGTAATTAATTACTTC |
| UP00238\_1 | 17 | GATAATTAATTACTTTG |
| UP00216\_1 | 17 | TTAAGGGGATTAACTAC |
| UP00239\_1 | 17 | TGAGGGGGATTAACTAT |
| UP00160\_1 | 17 | TGAGGGGGATTAACTAT |
| UP00208\_1 | 17 | TAGAGGGATTAAATTTC |
| UP00208\_2 | 17 | GATAATTAATCCCTCTT |
| UP00109\_1 | 15 | AAAAACGGATTATTG |
| UP00178\_1 | 17 | CGCGCTAATTAGGTATC |
| UP00237\_1 | 17 | CGTAATTAATTAATTGG |
| UP00229\_1 | 17 | GGAGGGGATTAATTTAT |
| UP00267\_1 | 17 | TGTAGGGATTAATTGTC |
| UP00247\_1 | 17 | TGAACTAATTAGCCCAC |
| UP00224\_1 | 16 | TGATTAATTAATTGAC |
| UP00248\_1 | 17 | CGAACTAATTAGTACTA |
| UP00185\_1 | 17 | TCACCCATCAATAATCA |
| UP00221\_1 | 16 | CAGCATTAATTAGTAG |
| UP00149\_1 | 17 | CGGAATTAATTAATAGG |
| UP00153\_1 | 17 | TTAGAGGGATTAACAAT |
| UP00125\_1 | 17 | TGAAGGGATTAATCATC |
| UP00265\_1 | 16 | AGGGGGATTAGCTGCC |
| UP00203\_1 | 16 | AAAGACCTGTCAATCC |
| UP00205\_1 | 16 | AAGCACCTGTCAATAT |
| UP00158\_1 | 17 | GATTAATTAATTAAGTC |
| UP00254\_1 | 16 | ATGTATTAATTAAGTA |
| UP00191\_1 | 16 | TTGTATGCAAATTAGA |
| UP00179\_1 | 16 | TTGTATGCAAATTAGA |
| UP00129\_1 | 17 | AATTAATTAATTAATTC |
| UP00128\_1 | 17 | GATAATTAATTAGTTTG |
| UP00211\_1 | 17 | AAAATATGCATAATAAA |
| UP00105\_1 | 17 | AATTAATTAATTAATTC |
| UP00118\_1 | 16 | AGTTATTAATGAGGTC |
| UP00146\_1 | 17 | GACGATAATGAGGTTGC |
| UP00146\_2 | 17 | AAACATAATGAGGTTGC |
| UP00172\_1 | 17 | CGAATTAATTAAGAAAC |
| UP00266\_1 | 17 | GTAACTAATTAACTACT |
| UP00136\_1 | 17 | AAAGCTAATTAGCGAAA |
| UP00253\_1 | 17 | TGCACTAATTAGCGCAC |
| UP00193\_1 | 17 | AAGACGCTGTAAAGCGA |
| UP00193\_2 | 17 | AGGACGCTGTAAAGGGA |
| UP00116\_1 | 17 | TGCCTTAATTAATGCTC |
| UP00257\_1 | 17 | CGCGTTAATTAATTGTG |
| UP00192\_1 | 17 | GATGGGGTATCATTTTT |
| UP00159\_1 | 17 | AATGGGGTATCACTTTT |
| UP00195\_1 | 17 | GATAGGGTATCACTTAT |
| UP00199\_1 | 17 | ATAAATGACACCTATCA |
| UP00008\_3 | 17 | AATAGGGTATCAATTAT |
| UP00008\_4 | 17 | AATAGGGTATCAATATT |
| UP00089\_3 | 17 | CCTTAGTTAACTAAAAT |
| UP00222\_1 | 17 | AGCTGTTAACTAGCCGT |
| UP00122\_1 | 17 | GATATTGACAGCTGCGT |
| UP00258\_1 | 16 | AACTAGCTGTCAATAC |
| UP00165\_1 | 16 | TAAGCCACTTGAAATT |
| UP00244\_1 | 17 | TAATTAATTAATAACTT |
| UP00142\_1 | 17 | CATAATTAATTAACGCG |
| UP00215\_1 | 16 | ACGTTAATTAACCCAG |
| UP00106\_1 | 16 | GTGCACTAATTAAGAC |
| UP00141\_1 | 17 | CGAGTTAATTAATAATT |

Random model letter frequencies
(from ./background):
  
A 0.241 C 0.259 G 0.259 T 0.241

---

**SECTION I: HIGH-SCORING MOTIF OCCURRENCES**


---

- There were
  220
  motif occurrences with a
  p-value less than
  0.0001.
- The p-value of a motif occurrence is defined as the
  probability of a random sequence of the same length as the motif
  matching that position of the sequence with as good or better a score.
- The score for the match of a position in a sequence to a motif
  is computed by summing the appropriate entries from each column of
  the position-dependent scoring matrix that represents the motif.
- The q-value of a motif occurrence is defined as the
  false discovery rate if the occurrence is accepted as significant.
- The table is sorted by increasing p-value.

| Motif | Sequence Name | Strand | Start | End | p-value | q-value | Matched Sequence |
| --- | --- | --- | --- | --- | --- | --- | --- |
| UP00060\_1 | chr19 | + | 2734545 | 2734560 | 1.05e-07 | 0.186 | `TGACCACGTGGTGCTG` |
| UP00060\_1 | chr7 | + | 4648277 | 4648292 | 3.07e-07 | 0.186 | `TGCGCACGTGGTCCGG` |
| UP00060\_1 | chr16 | + | 55523392 | 55523407 | 3.17e-07 | 0.186 | `TGCCCACGTGCTCGGT` |
| UP00060\_1 | chr19 | + | 12917496 | 12917511 | 3.47e-07 | 0.186 | `AGACCACGTGTGCGGG` |
| UP00060\_1 | chr19 | + | 17904526 | 17904541 | 1.14e-06 | 0.451 | `GGACCACGTGTGTAGT` |
| UP00060\_1 | chr12 | + | 120810944 | 120810959 | 1.26e-06 | 0.451 | `GGCGCACGTGTTCCGG` |
| UP00060\_1 | chr17 | − | 2561807 | 2561822 | 1.69e-06 | 0.478 | `GCACCACGTGGGCCGG` |
| UP00060\_1 | chr12 | + | 14410171 | 14410186 | 1.79e-06 | 0.478 | `GGAGCACGTGATCTTG` |
| UP00060\_1 | chr19 | + | 13126824 | 13126839 | 2.35e-06 | 0.558 | `TTACCACGTGGGCGAG` |
| UP00060\_1 | chr14 | − | 92240429 | 92240444 | 2.69e-06 | 0.576 | `GGACCACGTGTGCCTT` |
| UP00060\_1 | chr19 | − | 2734543 | 2734558 | 3.08e-06 | 0.599 | `GCACCACGTGGTCAGC` |
| UP00060\_1 | chr11 | + | 72531431 | 72531446 | 4.57e-06 | 0.766 | `CGCGCACGTGGGCGGG` |
| UP00060\_1 | chr1 | − | 171512897 | 171512912 | 4.87e-06 | 0.766 | `AAACCACGTGCTGCTT` |
| UP00060\_1 | chr17 | + | 2561809 | 2561824 | 5.25e-06 | 0.766 | `GGCCCACGTGGTGCGC` |
| UP00060\_1 | chr19 | − | 17904524 | 17904539 | 5.37e-06 | 0.766 | `TACACACGTGGTCCGG` |
| UP00060\_1 | chr17 | − | 35273949 | 35273964 | 5.98e-06 | 0.8 | `GGAGCACGTGGAGAGG` |
| UP00060\_1 | chr20 | + | 45847080 | 45847095 | 7.18e-06 | 0.829 | `GCCCCACGTGGGCTGG` |
| UP00060\_1 | chr20 | − | 45847078 | 45847093 | 9.53e-06 | 0.829 | `AGCCCACGTGGGGCTT` |
| UP00060\_1 | chr19 | − | 2559288 | 2559303 | 9.79e-06 | 0.829 | `ATCCCACGTGGGCGGA` |
| UP00060\_1 | chr9 | + | 37399246 | 37399261 | 9.99e-06 | 0.829 | `TGAACACGTGTTGAGC` |
| UP00060\_1 | chr6 | + | 151815045 | 151815060 | 1.01e-05 | 0.829 | `TGACCACGTGAGCCGC` |
| UP00060\_1 | chr12 | − | 120810942 | 120810957 | 1.01e-05 | 0.829 | `GGAACACGTGCGCCTG` |
| UP00060\_1 | chr11 | − | 85336834 | 85336849 | 1.03e-05 | 0.829 | `ATCCCACGTGGTTAAT` |
| UP00060\_1 | chr6 | − | 151815043 | 151815058 | 1.09e-05 | 0.829 | `GGCTCACGTGGTCAGA` |
| UP00060\_1 | chr16 | − | 55523390 | 55523405 | 1.14e-05 | 0.829 | `CGAGCACGTGGGCACG` |
| UP00060\_1 | chr11 | − | 61487608 | 61487623 | 1.16e-05 | 0.829 | `CTCCCACGTGATTGGG` |
| UP00060\_1 | chr1 | − | 154741575 | 154741590 | 1.17e-05 | 0.829 | `GGAGCACGTGACCGGG` |
| UP00060\_1 | chr10 | − | 73749843 | 73749858 | 1.2e-05 | 0.829 | `TACCCACGTGGGGCTG` |
| UP00060\_1 | chr12 | − | 120951554 | 120951569 | 1.33e-05 | 0.829 | `GTCCCACGTGTTCCAT` |
| UP00060\_1 | chr14 | + | 92240431 | 92240446 | 1.35e-05 | 0.829 | `GGCACACGTGGTCCAT` |
| UP00060\_1 | chr7 | − | 101869478 | 101869493 | 1.38e-05 | 0.829 | `TAAGCACGTGTTGCTA` |
| UP00060\_1 | chr17 | − | 46585947 | 46585962 | 1.53e-05 | 0.829 | `GACCCACGTGGTTCGC` |
| UP00060\_1 | chr1 | + | 110682961 | 110682976 | 1.57e-05 | 0.829 | `CAAGCACGTGTTCTAG` |
| UP00060\_1 | chr4 | + | 77338994 | 77339009 | 1.59e-05 | 0.829 | `AGATCACGTGCTGGAG` |
| UP00060\_1 | chr12 | − | 103617555 | 103617570 | 1.59e-05 | 0.829 | `AAACCACGTGTTATGC` |
| UP00060\_1 | chr7 | + | 101869480 | 101869495 | 1.64e-05 | 0.829 | `GCAACACGTGCTTATG` |
| UP00060\_1 | chr9 | − | 115212496 | 115212511 | 1.66e-05 | 0.829 | `AGGCCACGTGGGGCGG` |
| UP00060\_1 | chr12 | + | 4098221 | 4098236 | 1.72e-05 | 0.829 | `GGACCACATGGTGCTT` |
| UP00060\_1 | chr6 | + | 33237473 | 33237488 | 1.8e-05 | 0.829 | `CGCCCACGTGGGGCTT` |
| UP00060\_1 | chr16 | − | 23745674 | 23745689 | 1.8e-05 | 0.829 | `ATGCCACGTGGTCCTG` |
| UP00060\_1 | chr13 | − | 98870666 | 98870681 | 1.83e-05 | 0.829 | `TGCCCACGTGTGGGGC` |
| UP00060\_1 | chr15 | + | 29408616 | 29408631 | 1.83e-05 | 0.829 | `AAACCACGTGGCAGGT` |
| UP00060\_1 | chr7 | − | 138675866 | 138675881 | 1.84e-05 | 0.829 | `CGCCCACGTGAGGGGT` |
| UP00060\_1 | chr2 | − | 42182898 | 42182913 | 1.86e-05 | 0.829 | `AGAGCACGTGGTGGCC` |
| UP00060\_1 | chr20 | − | 61830254 | 61830269 | 1.9e-05 | 0.829 | `AAACCACATGGTGTGG` |
| UP00060\_1 | chr8 | + | 101802337 | 101802352 | 1.93e-05 | 0.829 | `TCAACACGTGGTATTT` |
| UP00060\_1 | chr2 | + | 88772445 | 88772460 | 1.96e-05 | 0.829 | `TTCCCACGTGCGGCTG` |
| UP00060\_1 | chr19 | − | 10625609 | 10625624 | 2e-05 | 0.829 | `TTACCACGTGATGCAA` |
| UP00060\_1 | chr1 | − | 110682959 | 110682974 | 2.08e-05 | 0.829 | `AGAACACGTGCTTGCT` |
| UP00060\_1 | chr19 | + | 10625611 | 10625626 | 2.11e-05 | 0.829 | `GCATCACGTGGTAATG` |
| UP00060\_1 | chr2 | + | 27433229 | 27433244 | 2.29e-05 | 0.829 | `TAAACACGTGTGCTTG` |
| UP00060\_1 | chr11 | + | 65443592 | 65443607 | 2.29e-05 | 0.829 | `TGAGCACGTGGCAGAG` |
| UP00060\_1 | chr1 | + | 143245682 | 143245697 | 2.32e-05 | 0.829 | `AGAGCACGTGGATGTT` |
| UP00060\_1 | chr1 | − | 190814943 | 190814958 | 2.38e-05 | 0.829 | `AAAACACGTGGGGGTT` |
| UP00060\_1 | chr6 | − | 31636201 | 31636216 | 2.38e-05 | 0.829 | `AAACCATGTGGTCAGA` |
| UP00060\_1 | chr17 | + | 39781241 | 39781256 | 2.38e-05 | 0.829 | `GGCACACGTGGTCTCT` |
| UP00060\_1 | chr15 | + | 86983784 | 86983799 | 2.42e-05 | 0.829 | `TCAGCACATGGTGGGG` |
| UP00060\_1 | chr17 | − | 73652082 | 73652097 | 2.42e-05 | 0.829 | `TCTCCACGTGGTGGTG` |
| UP00060\_1 | chr19 | + | 2559290 | 2559305 | 2.42e-05 | 0.829 | `CGCCCACGTGGGATTT` |
| UP00060\_1 | chr12 | + | 91321963 | 91321978 | 2.45e-05 | 0.829 | `TGAGCATGTGGGCTGT` |
| UP00060\_1 | chr17 | − | 39781239 | 39781254 | 2.47e-05 | 0.829 | `AGACCACGTGTGCCCA` |
| UP00060\_1 | chr8 | − | 101802335 | 101802350 | 2.55e-05 | 0.829 | `ATACCACGTGTTGAAC` |
| UP00060\_1 | chr9 | − | 37399244 | 37399259 | 2.59e-05 | 0.829 | `TCAACACGTGTTCACG` |
| UP00060\_1 | chr12 | + | 47810334 | 47810349 | 2.61e-05 | 0.829 | `GAAGCACGTGGCCAGA` |
| UP00060\_1 | chr15 | − | 29408614 | 29408629 | 2.71e-05 | 0.829 | `CTGCCACGTGGTTTGG` |
| UP00060\_1 | chr19 | − | 54162787 | 54162802 | 2.71e-05 | 0.829 | `GCATCACGTGTTCCTG` |
| UP00060\_1 | chr22 | + | 49315068 | 49315083 | 2.75e-05 | 0.829 | `GAGCCACGTGCTCCTG` |
| UP00060\_1 | chr7 | − | 4648275 | 4648290 | 2.88e-05 | 0.829 | `GGACCACGTGCGCACC` |
| UP00060\_1 | chr22 | − | 49315066 | 49315081 | 2.98e-05 | 0.829 | `GGAGCACGTGGCTCAG` |
| UP00060\_1 | chr15 | − | 56534217 | 56534232 | 3.03e-05 | 0.829 | `GCTGCACGTGGTAAGG` |
| UP00060\_1 | chr1 | + | 17104290 | 17104305 | 3.1e-05 | 0.829 | `AGAGCACATGTTATGG` |
| UP00060\_1 | chr9 | − | 113789047 | 113789062 | 3.1e-05 | 0.829 | `AAAGCACATGGTGTGG` |
| UP00060\_1 | chr12 | + | 122170430 | 122170445 | 3.1e-05 | 0.829 | `CACCCACGTGAGGAGG` |
| UP00060\_1 | chr9 | + | 6671530 | 6671545 | 3.12e-05 | 0.829 | `GGAGAACGTGGTCCGG` |
| UP00060\_1 | chr2 | + | 42182900 | 42182915 | 3.14e-05 | 0.829 | `CCACCACGTGCTCTCT` |
| UP00060\_1 | chr1 | − | 41967312 | 41967327 | 3.16e-05 | 0.829 | `GAAGCACGTGGCCAAG` |
| UP00060\_1 | chr2 | − | 20931831 | 20931846 | 3.19e-05 | 0.829 | `GGGCCACGTGGGAGGA` |
| UP00060\_1 | chr11 | + | 85336836 | 85336851 | 3.21e-05 | 0.829 | `TAACCACGTGGGATTC` |
| UP00060\_1 | chr7 | − | 55605133 | 55605148 | 3.24e-05 | 0.829 | `TTGCCACGTGGTTGAT` |
| UP00060\_1 | chr17 | + | 38024640 | 38024655 | 3.24e-05 | 0.829 | `GGAGCACGTGGCGAGC` |
| UP00060\_1 | chr2 | + | 201702234 | 201702249 | 3.26e-05 | 0.829 | `AGGCCACGTGGGTGGA` |
| UP00060\_1 | chr8 | + | 131329382 | 131329397 | 3.31e-05 | 0.829 | `ACACCACGTGGAGATG` |
| UP00060\_1 | chr1 | + | 3583628 | 3583643 | 3.33e-05 | 0.829 | `GTCACACGTGTGTGGG` |
| UP00060\_1 | chr15 | − | 29343034 | 29343049 | 3.33e-05 | 0.829 | `TATGCACGTGCTGTGG` |
| UP00060\_1 | chr8 | − | 131329380 | 131329395 | 3.43e-05 | 0.829 | `TCTCCACGTGGTGTTG` |
| UP00060\_1 | chr5 | + | 156862120 | 156862135 | 3.45e-05 | 0.829 | `ATGCCACGTGATCGTG` |
| UP00060\_1 | chr12 | − | 12054622 | 12054637 | 3.45e-05 | 0.829 | `AGGCCACGTGGCCGGG` |
| UP00060\_1 | chr9 | + | 135193061 | 135193076 | 3.5e-05 | 0.829 | `CCCGCACGTGTTCCTG` |
| UP00060\_1 | chr1 | − | 51198779 | 51198794 | 3.52e-05 | 0.829 | `TAACCACGTGAGGTGC` |
| UP00060\_1 | chr19 | + | 2734564 | 2734579 | 3.52e-05 | 0.829 | `CGGCCACGTGGTGCAG` |
| UP00060\_1 | chr19 | + | 59404119 | 59404134 | 3.52e-05 | 0.829 | `AAACCACGTGGAGAAG` |
| UP00060\_1 | chr1 | − | 35412493 | 35412508 | 3.71e-05 | 0.848 | `GGAGCACATGGTATTT` |
| UP00060\_1 | chr15 | + | 29310523 | 29310538 | 3.71e-05 | 0.848 | `GGAGCACGTGCAAGGA` |
| UP00060\_1 | chr1 | + | 51198781 | 51198796 | 3.76e-05 | 0.848 | `ACCTCACGTGGTTAGT` |
| UP00060\_1 | chr10 | − | 7560117 | 7560132 | 3.81e-05 | 0.848 | `AGTGCACGTGGGGCGT` |
| UP00060\_1 | chr10 | − | 95452347 | 95452362 | 3.81e-05 | 0.848 | `GTAACACGTGGCCGTG` |
| UP00060\_1 | chr17 | + | 35273951 | 35273966 | 3.84e-05 | 0.848 | `TCTCCACGTGCTCCTG` |
| UP00060\_1 | chr5 | − | 139907412 | 139907427 | 3.89e-05 | 0.851 | `CGACCACGTGCAGCTG` |
| UP00060\_1 | chr7 | + | 1510699 | 1510714 | 4e-05 | 0.854 | `CGCGCATGTGGTCCGG` |
| UP00060\_1 | chr1 | + | 171512899 | 171512914 | 4.03e-05 | 0.854 | `GCAGCACGTGGTTTCA` |
| UP00060\_1 | chr4 | − | 78294552 | 78294567 | 4.03e-05 | 0.854 | `ACAGCATGTGGTAGGG` |
| UP00060\_1 | chr10 | − | 73705319 | 73705334 | 4.09e-05 | 0.854 | `GGAGCACGTGCCTCTT` |
| UP00060\_1 | chr2 | + | 201831283 | 201831298 | 4.11e-05 | 0.854 | `TGAGCACGTGGAGTTA` |
| UP00060\_1 | chr2 | + | 191593357 | 191593372 | 4.17e-05 | 0.854 | `GCCCCACGTGCTTTCG` |
| UP00060\_1 | chr8 | + | 134563324 | 134563339 | 4.2e-05 | 0.854 | `AGAGCACATGTTCTTT` |
| UP00060\_1 | chr5 | + | 138753609 | 138753624 | 4.35e-05 | 0.854 | `ATGCCACGTGGGCATT` |
| UP00060\_1 | chr12 | − | 91321961 | 91321976 | 4.37e-05 | 0.854 | `AGCCCACATGCTCAGA` |
| UP00060\_1 | chr19 | − | 2734562 | 2734577 | 4.37e-05 | 0.854 | `GCACCACGTGGCCGGC` |
| UP00060\_1 | chr1 | + | 146273136 | 146273151 | 4.47e-05 | 0.854 | `GCCCCACGTGGAAAGG` |
| UP00060\_1 | chr1 | + | 190814945 | 190814960 | 4.47e-05 | 0.854 | `CCCCCACGTGTTTTGA` |
| UP00060\_1 | chr2 | + | 231233763 | 231233778 | 4.47e-05 | 0.854 | `AGACCACGTGAATAAG` |
| UP00060\_1 | chr7 | + | 25974526 | 25974541 | 4.47e-05 | 0.854 | `CAATCACGTGGTAGAG` |
| UP00060\_1 | chr6 | − | 32733871 | 32733886 | 4.68e-05 | 0.878 | `AAAGCATGTGCTGGGT` |
| UP00060\_1 | chr8 | − | 134563322 | 134563337 | 4.68e-05 | 0.878 | `AGAACATGTGCTCTGT` |
| UP00060\_1 | chr1 | + | 35412495 | 35412510 | 4.81e-05 | 0.88 | `ATACCATGTGCTCCTT` |
| UP00060\_1 | chr1 | − | 146273134 | 146273149 | 4.81e-05 | 0.88 | `TTTCCACGTGGGGCTG` |
| UP00060\_1 | chr5 | + | 180596297 | 180596312 | 4.84e-05 | 0.88 | `TCTGCACGTGGTATGT` |
| UP00060\_1 | chr16 | + | 1954802 | 1954817 | 4.87e-05 | 0.88 | `GACTCACGTGTTTTGT` |
| UP00060\_1 | chr6 | − | 33237471 | 33237486 | 4.93e-05 | 0.88 | `GCCCCACGTGGGCGTC` |
| UP00060\_1 | chr7 | + | 23024882 | 23024897 | 4.93e-05 | 0.88 | `AAATCACGTGGTGGAA` |
| UP00060\_1 | chr19 | + | 5741753 | 5741768 | 5.04e-05 | 0.89 | `AGAGCACATGCGCCGT` |
| UP00060\_1 | chr20 | + | 61830271 | 61830286 | 5.07e-05 | 0.89 | `AGAGCACGTTGTGAGT` |
| UP00060\_1 | chr7 | − | 20226520 | 20226535 | 5.37e-05 | 0.931 | `TGACAACGTGGTAAGA` |
| UP00060\_1 | chr2 | + | 233632933 | 233632948 | 5.52e-05 | 0.931 | `ATCCCACGTGGGTGTC` |
| UP00060\_1 | chr7 | − | 25974524 | 25974539 | 5.59e-05 | 0.931 | `CTACCACGTGATTGCA` |
| UP00060\_1 | chr12 | − | 47810332 | 47810347 | 5.59e-05 | 0.931 | `TGGCCACGTGCTTCCT` |
| UP00060\_1 | chr15 | − | 86983782 | 86983797 | 5.59e-05 | 0.931 | `CCACCATGTGCTGAGG` |
| UP00060\_1 | chr10 | + | 7560119 | 7560134 | 5.63e-05 | 0.931 | `GCCCCACGTGCACTGG` |
| UP00060\_1 | chr11 | − | 65076557 | 65076572 | 5.66e-05 | 0.931 | `AGACCACATGGGGGAT` |
| UP00060\_1 | chr9 | − | 85770726 | 85770741 | 5.7e-05 | 0.931 | `TGACCACATGGGATAG` |
| UP00060\_1 | chr15 | + | 29343523 | 29343538 | 5.7e-05 | 0.931 | `TCTCCACGTGTTTCTG` |
| UP00060\_1 | chr12 | − | 14410169 | 14410184 | 5.77e-05 | 0.936 | `AGATCACGTGCTCCCA` |
| UP00060\_1 | chr19 | − | 13126822 | 13126837 | 5.92e-05 | 0.937 | `CGCCCACGTGGTAACC` |
| UP00060\_1 | chr17\_random | − | 88269 | 88284 | 5.92e-05 | 0.937 | `TGAGCACGCGCTCTGG` |
| UP00060\_1 | chr17 | − | 7416567 | 7416582 | 5.96e-05 | 0.937 | `CTCACACGTGCGGAGG` |
| UP00060\_1 | chr15 | + | 56534269 | 56534284 | 5.99e-05 | 0.937 | `CGCCCACATGGGCTGG` |
| UP00060\_1 | chr16 | − | 1981302 | 1981317 | 5.99e-05 | 0.937 | `CCTGCACGTGGTGGGT` |
| UP00060\_1 | chr11 | − | 62146324 | 62146339 | 6.11e-05 | 0.941 | `GTACCACGTGCACCTA` |
| UP00060\_1 | chr19 | − | 47141429 | 47141444 | 6.11e-05 | 0.941 | `AGCCCACATGTTGATT` |
| UP00060\_1 | chr7 | + | 22621370 | 22621385 | 6.26e-05 | 0.95 | `AGAACATGTGGTAAGA` |
| UP00060\_1 | chr15 | + | 29343036 | 29343051 | 6.34e-05 | 0.95 | `ACAGCACGTGCATAGT` |
| UP00060\_1 | chr7 | − | 1510697 | 1510712 | 6.55e-05 | 0.95 | `GGACCACATGCGCGCG` |
| UP00060\_1 | chr2 | − | 231233761 | 231233776 | 6.62e-05 | 0.95 | `TATTCACGTGGTCTGT` |
| UP00060\_1 | chr8 | + | 126519546 | 126519561 | 6.62e-05 | 0.95 | `AAACCACGTGAAATGT` |
| UP00060\_1 | chr16 | − | 22216224 | 22216239 | 6.71e-05 | 0.95 | `CGGACACGTGGGGGGG` |
| UP00060\_1 | chr12 | + | 6923123 | 6923138 | 6.75e-05 | 0.95 | `TTACCACATGCGTCGT` |
| UP00060\_1 | chr12 | − | 102847284 | 102847299 | 6.84e-05 | 0.95 | `GAAGAACGTGGTTGGG` |
| UP00060\_1 | chr17 | + | 46585949 | 46585964 | 6.84e-05 | 0.95 | `GAACCACGTGGGTCCC` |
| UP00060\_1 | chr5 | + | 139907414 | 139907429 | 6.92e-05 | 0.95 | `GCTGCACGTGGTCGGC` |
| UP00060\_1 | chr15 | + | 66874754 | 66874769 | 7e-05 | 0.95 | `GGAGCACATGGCCCGG` |
| UP00060\_1 | chr15 | − | 68095994 | 68096009 | 7e-05 | 0.95 | `TGCCCATGTGATGTTG` |
| UP00060\_1 | chr16 | + | 87572576 | 87572591 | 7e-05 | 0.95 | `TGAGCATGTGGCTGGG` |
| UP00060\_1 | chr17 | + | 7416569 | 7416584 | 7e-05 | 0.95 | `TCCGCACGTGTGAGAG` |
| UP00060\_1 | chr1 | − | 181259669 | 181259684 | 7.05e-05 | 0.95 | `CCCACACGTGTTGGTG` |
| UP00060\_1 | chr11 | + | 62146326 | 62146341 | 7.14e-05 | 0.95 | `GGTGCACGTGGTACCT` |
| UP00060\_1 | chr10 | + | 73749845 | 73749860 | 7.18e-05 | 0.95 | `GCCCCACGTGGGTATC` |
| UP00060\_1 | chr11 | − | 82548974 | 82548989 | 7.18e-05 | 0.95 | `ACCCCACGTGTATAGG` |
| UP00060\_1 | chr14 | − | 96843159 | 96843174 | 7.18e-05 | 0.95 | `ATCTCACGTGGGTATG` |
| UP00060\_1 | chr20 | + | 17784877 | 17784892 | 7.18e-05 | 0.95 | `ATGGCACGTGGTTGTA` |
| UP00060\_1 | chr6 | − | 26135256 | 26135271 | 7.22e-05 | 0.95 | `GGAGAACGTGATCCGG` |
| UP00060\_1 | chr6 | + | 27215255 | 27215270 | 7.22e-05 | 0.95 | `GGAGAACGTGATCCGG` |
| UP00060\_1 | chr19 | − | 55991350 | 55991365 | 7.27e-05 | 0.95 | `GGCCCATGTGATGGTT` |
| UP00060\_1 | chr10 | + | 73765081 | 73765096 | 7.35e-05 | 0.95 | `TTCCCACATGGTTCTT` |
| UP00060\_1 | chr16 | + | 66527082 | 66527097 | 7.35e-05 | 0.95 | `CACCCACGTGGCCCTG` |
| UP00060\_1 | chr9 | − | 122704195 | 122704210 | 7.4e-05 | 0.95 | `GGGGCACGTGGCAGGG` |
| UP00060\_1 | chr19 | − | 1334713 | 1334728 | 7.4e-05 | 0.95 | `TGCGCACGTGGGGGCC` |
| UP00060\_1 | chr14 | + | 22095303 | 22095318 | 7.45e-05 | 0.95 | `AGGCCACGTGCCGAGG` |
| UP00060\_1 | chr9 | − | 135193059 | 135193074 | 7.54e-05 | 0.95 | `GGAACACGTGCGGGAC` |
| UP00060\_1 | chr15 | + | 56534219 | 56534234 | 7.54e-05 | 0.95 | `TTACCACGTGCAGCGC` |
| UP00060\_1 | chr17 | + | 35167905 | 35167920 | 7.54e-05 | 0.95 | `ACCACACGTGTGGTGG` |
| UP00060\_1 | chr7 | + | 4648266 | 4648281 | 7.63e-05 | 0.95 | `GCGCCACGTGGTGCGC` |
| UP00060\_1 | chr17 | + | 59577491 | 59577506 | 7.63e-05 | 0.95 | `AGAGCATGTGCTTGCG` |
| UP00060\_1 | chr6 | − | 106669305 | 106669320 | 7.81e-05 | 0.952 | `GGCCCACATGCGACGG` |
| UP00060\_1 | chr16 | + | 23745676 | 23745691 | 7.81e-05 | 0.952 | `GGACCACGTGGCATCA` |
| UP00060\_1 | chr16 | + | 66666408 | 66666423 | 7.91e-05 | 0.952 | `GGCCCATGTGCTTGAT` |
| UP00060\_1 | chr11 | + | 61487610 | 61487625 | 8e-05 | 0.952 | `CAATCACGTGGGAGGA` |
| UP00060\_1 | chr1 | + | 28083474 | 28083489 | 8.1e-05 | 0.952 | `TAATCACGTGTGGTAG` |
| UP00060\_1 | chr13 | − | 76799256 | 76799271 | 8.15e-05 | 0.952 | `TGAGCATGTGCGAGGA` |
| UP00060\_1 | chr2 | + | 233655593 | 233655608 | 8.2e-05 | 0.952 | `GGCCCACGTGTCATAG` |
| UP00060\_1 | chr10 | − | 97506871 | 97506886 | 8.2e-05 | 0.952 | `TAAGCATGTGGGAATG` |
| UP00060\_1 | chr19 | − | 51913683 | 51913698 | 8.3e-05 | 0.952 | `GCTCCACGTGGTGTGC` |
| UP00060\_1 | chr1 | − | 28399057 | 28399072 | 8.34e-05 | 0.952 | `AAACCACATGTGAAGT` |
| UP00060\_1 | chr3 | − | 76670032 | 76670047 | 8.34e-05 | 0.952 | `TGCCCACATGTGTATG` |
| UP00060\_1 | chr7 | + | 55605135 | 55605150 | 8.34e-05 | 0.952 | `CAACCACGTGGCAAAT` |
| UP00060\_1 | chr9 | − | 125141517 | 125141532 | 8.34e-05 | 0.952 | `AAACCATGTGTGACGG` |
| UP00060\_1 | chr2 | − | 201831281 | 201831296 | 8.39e-05 | 0.952 | `ACTCCACGTGCTCAGC` |
| UP00060\_1 | chr2 | − | 233632931 | 233632946 | 8.39e-05 | 0.952 | `CACCCACGTGGGATGC` |
| UP00060\_1 | chr19 | + | 44585151 | 44585166 | 8.39e-05 | 0.952 | `AGCACACGTGCCGCGG` |
| UP00060\_1 | chr1 | + | 158947252 | 158947267 | 8.44e-05 | 0.952 | `CTACCACATGTTTGTT` |
| UP00060\_1 | chr6 | + | 27889882 | 27889897 | 8.49e-05 | 0.952 | `AAAACACGTGTTAGCA` |
| UP00060\_1 | chr9 | − | 91268764 | 91268779 | 8.49e-05 | 0.952 | `AAAGCACGTGTCTAAG` |
| UP00060\_1 | chr9 | + | 85770728 | 85770743 | 8.55e-05 | 0.953 | `ATCCCATGTGGTCATA` |
| UP00060\_1 | chr7 | − | 23024880 | 23024895 | 8.6e-05 | 0.954 | `CCACCACGTGATTTTC` |
| UP00060\_1 | chr10 | + | 95452349 | 95452364 | 8.7e-05 | 0.954 | `CGGCCACGTGTTACAT` |
| UP00060\_1 | chr17 | + | 5283132 | 5283147 | 8.7e-05 | 0.954 | `CGAACACGTGCAGATG` |
| UP00060\_1 | chr6 | − | 36762772 | 36762787 | 8.8e-05 | 0.954 | `AGCCCACGTGGCATGC` |
| UP00060\_1 | chr11 | + | 82548976 | 82548991 | 8.85e-05 | 0.954 | `TATACACGTGGGGTGG` |
| UP00060\_1 | chr5 | − | 138753607 | 138753622 | 8.96e-05 | 0.954 | `TGCCCACGTGGCATCT` |
| UP00060\_1 | chr13 | + | 98870668 | 98870683 | 8.96e-05 | 0.954 | `CCCACACGTGGGCAGA` |
| UP00060\_1 | chr20 | − | 17784875 | 17784890 | 8.96e-05 | 0.954 | `CAACCACGTGCCATTG` |
| UP00060\_1 | chr20 | − | 45477525 | 45477540 | 8.96e-05 | 0.954 | `GAAACATGTGGTTATT` |
| UP00060\_1 | chr11 | − | 73360312 | 73360327 | 9.01e-05 | 0.955 | `AACACACGTGTTGACT` |
| UP00060\_1 | chr4 | − | 185633024 | 185633039 | 9.11e-05 | 0.957 | `GCCGCACGTGCGGGCG` |
| UP00060\_1 | chr7 | − | 101719110 | 101719125 | 9.16e-05 | 0.957 | `TGACCTCGTGTTGAGG` |
| UP00060\_1 | chr9 | − | 70445469 | 70445484 | 9.22e-05 | 0.957 | `TCAGCACATGCTTCTG` |
| UP00060\_1 | chr11 | + | 73360314 | 73360329 | 9.27e-05 | 0.957 | `TCAACACGTGTGTTAT` |
| UP00060\_1 | chr19 | − | 17238823 | 17238838 | 9.27e-05 | 0.957 | `TTCCCATGTGGGTGTG` |
| UP00060\_1 | chr2 | + | 6403330 | 6403345 | 9.33e-05 | 0.957 | `TCACCACATGTTTCAG` |
| UP00060\_1 | chr3 | − | 196349993 | 196350008 | 9.38e-05 | 0.957 | `GGAGCACGTGAAAAGC` |
| UP00060\_1 | chr9 | − | 37399236 | 37399251 | 9.38e-05 | 0.957 | `TGTTCACGTGTTTGGA` |
| UP00060\_1 | chr5 | − | 1846246 | 1846261 | 9.55e-05 | 0.965 | `TTACCACGTTGGCGTG` |
| UP00060\_1 | chr2 | − | 73365688 | 73365703 | 9.6e-05 | 0.965 | `GGTACACGTGTTCCTA` |
| UP00060\_1 | chr3 | − | 189012269 | 189012284 | 9.6e-05 | 0.965 | `CTTTCACGTGGTGGGG` |
| UP00060\_1 | chr3 | + | 157055230 | 157055245 | 9.65e-05 | 0.966 | `TAATCACGTGGGGTCT` |
| UP00060\_1 | chr2 | − | 6403328 | 6403343 | 9.71e-05 | 0.967 | `GAAACATGTGGTGAAG` |
| UP00060\_1 | chr19 | + | 54162789 | 54162804 | 9.76e-05 | 0.968 | `GGAACACGTGATGCCC` |
| UP00060\_1 | chr1 | + | 28399059 | 28399074 | 9.88e-05 | 0.971 | `TTCACATGTGGTTTGG` |
| UP00060\_1 | chr5 | − | 88675320 | 88675335 | 9.93e-05 | 0.971 | `TTGGCACGTGTGCCTT` |
| UP00060\_1 | chr7 | + | 101854098 | 101854113 | 9.93e-05 | 0.971 | `TGAGCACATGCCCTGG` |
| UP00060\_1 | chr20 | − | 29664364 | 29664379 | 9.99e-05 | 0.972 | `AGACCAGGTGCTCTGG` |

---

**DEBUGGING INFORMATION**


---

Command line:

```
/ebi/sw/MEME/VM-cluster410/meme-versions/4.10.0/bin/fimo --parse-genomic-coord --verbosity 1 --oc fimo_out_17 --bgfile ./background --motif UP00060_1 db/uniprobe_mouse.meme ./Supplementary_Table_1.500bp.fa
```

Settings:

```
|  |  |  |
| --- | --- | --- |
| output directory = fimo_out_17 | MEME file name = db/uniprobe_mouse.meme | sequence file name = ./Supplementary_Table_1.500bp.fa |
| background file name = ./background | allow clobber = true | compute q-values = true |
| parse genomic coord. = true | text only = false | scan both strands = true |
| max sequence length = 250000000 | output threshold = 0.0001 | threshold type = p-value |
| max stored scores = 100000 | pseudocount = 0.1 | verbosity = 1 |
| selected motif = UP00060_1 |  |  |
```

This information can be useful in the event you wish to report a
problem with the FIMO software.

---

**Go to top**
