# Supplementary material for: The MEME Suite
Source: Nucleic Acids Res. 2015 May 7;43(Web Server issue):W39–49. doi: 10.1093/nar/gkv416 (PMC4489269; doi:10.1093/nar/gkv416)
Supplement: SUPPLEMENTARY DATA [file supp_gkv416_nar-00283-web-b-2015-File005.zip › case4/meme-chip/fimo_out_18/fimo.html]

FIMO Results


---

|  |  |  |
| --- | --- | --- |
| **Database and Motifs** | **High-scoring Motif Occurrences** | **Debugging Information** |

  
  

---

**FIMO - Motif search tool**


---

FIMO version 4.10.0,
(Release date: Wed May 21 10:35:36 2014 +1000)

For further information on how to interpret these results
or to get a copy of the FIMO software please access
http://meme.nbcr.net

If you use FIMO in your research, please cite the following paper:  
Charles E. Grant, Timothy L. Bailey, and William Stafford Noble,
"FIMO: Scanning for occurrences of a given motif",
*Bioinformatics*, **27**(7):1017-1018, 2011.
[full text]

---

**DATABASE AND MOTIFS**


---

DATABASE
./Supplementary\_Table\_1.500bp.fa  
Database contains
2776
sequences,
1388000
residues

MOTIFS
dreme\_out/dreme.xml
(nucleotide)

| MOTIF | WIDTH | BEST POSSIBLE MATCH |
| --- | --- | --- |
| GGAARY | 6 | GGAAGT |
| AVTGAAA | 7 | ACTGAAA |
| RCAGCTGY | 8 | GCAGCTGC |
| AKAAAH | 6 | AGAAAA |
| RAGKTCA | 7 | GAGGTCA |
| CMCAGM | 6 | CCCAGC |
| CCCCRCCC | 8 | CCCCGCCC |
| AAATR | 5 | AAATG |
| GAAASCA | 7 | GAAAGCA |
| CCGSCTCC | 8 | CCGCCTCC |
| CCWCCTGC | 8 | CCACCTGC |

Random model letter frequencies
(from ./background):
  
A 0.241 C 0.259 G 0.259 T 0.241

---

**SECTION I: HIGH-SCORING MOTIF OCCURRENCES**


---

- There were
  517
  motif occurrences with a
  p-value less than
  0.0001.
- The p-value of a motif occurrence is defined as the
  probability of a random sequence of the same length as the motif
  matching that position of the sequence with as good or better a score.
- The score for the match of a position in a sequence to a motif
  is computed by summing the appropriate entries from each column of
  the position-dependent scoring matrix that represents the motif.
- The q-value of a motif occurrence is defined as the
  false discovery rate if the occurrence is accepted as significant.
- The table is sorted by increasing p-value.

| Motif | Sequence Name | Strand | Start | End | p-value | q-value | Matched Sequence |
| --- | --- | --- | --- | --- | --- | --- | --- |
| CCWCCTGC | chr1 | + | 2481018 | 2481025 | 1.76e-05 | 0.24 | `CCACCTGC` |
| CCWCCTGC | chr1 | + | 9409384 | 9409391 | 1.76e-05 | 0.24 | `CCACCTGC` |
| CCWCCTGC | chr1 | − | 28083493 | 28083500 | 1.76e-05 | 0.24 | `CCACCTGC` |
| CCWCCTGC | chr1 | − | 31669059 | 31669066 | 1.76e-05 | 0.24 | `CCACCTGC` |
| CCWCCTGC | chr1 | − | 36327120 | 36327127 | 1.76e-05 | 0.24 | `CCACCTGC` |
| CCWCCTGC | chr1 | + | 38228420 | 38228427 | 1.76e-05 | 0.24 | `CCACCTGC` |
| CCWCCTGC | chr1 | + | 111945328 | 111945335 | 1.76e-05 | 0.24 | `CCACCTGC` |
| CCWCCTGC | chr1 | + | 117998658 | 117998665 | 1.76e-05 | 0.24 | `CCACCTGC` |
| CCWCCTGC | chr1 | − | 148801390 | 148801397 | 1.76e-05 | 0.24 | `CCACCTGC` |
| CCWCCTGC | chr1 | − | 148852885 | 148852892 | 1.76e-05 | 0.24 | `CCACCTGC` |
| CCWCCTGC | chr1 | + | 158870702 | 158870709 | 1.76e-05 | 0.24 | `CCACCTGC` |
| CCWCCTGC | chr1 | − | 158911032 | 158911039 | 1.76e-05 | 0.24 | `CCACCTGC` |
| CCWCCTGC | chr1 | + | 161558288 | 161558295 | 1.76e-05 | 0.24 | `CCACCTGC` |
| CCWCCTGC | chr1 | + | 181259765 | 181259772 | 1.76e-05 | 0.24 | `CCACCTGC` |
| CCWCCTGC | chr1 | − | 220001462 | 220001469 | 1.76e-05 | 0.24 | `CCACCTGC` |
| CCWCCTGC | chr1 | + | 220016840 | 220016847 | 1.76e-05 | 0.24 | `CCACCTGC` |
| CCWCCTGC | chr2 | + | 8359860 | 8359867 | 1.76e-05 | 0.24 | `CCACCTGC` |
| CCWCCTGC | chr2 | + | 12786334 | 12786341 | 1.76e-05 | 0.24 | `CCACCTGC` |
| CCWCCTGC | chr2 | + | 33554811 | 33554818 | 1.76e-05 | 0.24 | `CCACCTGC` |
| CCWCCTGC | chr2 | + | 55349757 | 55349764 | 1.76e-05 | 0.24 | `CCACCTGC` |
| CCWCCTGC | chr2 | − | 98454306 | 98454313 | 1.76e-05 | 0.24 | `CCACCTGC` |
| CCWCCTGC | chr2 | − | 160276907 | 160276914 | 1.76e-05 | 0.24 | `CCACCTGC` |
| CCWCCTGC | chr2 | − | 201689611 | 201689618 | 1.76e-05 | 0.24 | `CCACCTGC` |
| CCWCCTGC | chr2 | − | 230989259 | 230989266 | 1.76e-05 | 0.24 | `CCACCTGC` |
| CCWCCTGC | chr2 | + | 231162516 | 231162523 | 1.76e-05 | 0.24 | `CCACCTGC` |
| CCWCCTGC | chr2 | − | 231233663 | 231233670 | 1.76e-05 | 0.24 | `CCACCTGC` |
| CCWCCTGC | chr2 | + | 241903545 | 241903552 | 1.76e-05 | 0.24 | `CCACCTGC` |
| CCWCCTGC | chr3 | − | 13088699 | 13088706 | 1.76e-05 | 0.24 | `CCACCTGC` |
| CCWCCTGC | chr3 | + | 46943535 | 46943542 | 1.76e-05 | 0.24 | `CCACCTGC` |
| CCWCCTGC | chr3 | + | 46943669 | 46943676 | 1.76e-05 | 0.24 | `CCACCTGC` |
| CCWCCTGC | chr3 | − | 128952064 | 128952071 | 1.76e-05 | 0.24 | `CCACCTGC` |
| CCWCCTGC | chr3 | + | 134692950 | 134692957 | 1.76e-05 | 0.24 | `CCACCTGC` |
| CCWCCTGC | chr3 | − | 157874771 | 157874778 | 1.76e-05 | 0.24 | `CCACCTGC` |
| CCWCCTGC | chr4 | − | 39878523 | 39878530 | 1.76e-05 | 0.24 | `CCACCTGC` |
| CCWCCTGC | chr4 | − | 39998485 | 39998492 | 1.76e-05 | 0.24 | `CCACCTGC` |
| CCWCCTGC | chr4 | − | 40002105 | 40002112 | 1.76e-05 | 0.24 | `CCACCTGC` |
| CCWCCTGC | chr4 | + | 100098231 | 100098238 | 1.76e-05 | 0.24 | `CCACCTGC` |
| CCWCCTGC | chr5 | − | 55474405 | 55474412 | 1.76e-05 | 0.24 | `CCACCTGC` |
| CCWCCTGC | chr5 | + | 126683845 | 126683852 | 1.76e-05 | 0.24 | `CCACCTGC` |
| CCWCCTGC | chr5 | − | 180170084 | 180170091 | 1.76e-05 | 0.24 | `CCACCTGC` |
| CCWCCTGC | chr6 | − | 7844273 | 7844280 | 1.76e-05 | 0.24 | `CCACCTGC` |
| CCWCCTGC | chr6 | − | 11942523 | 11942530 | 1.76e-05 | 0.24 | `CCACCTGC` |
| CCWCCTGC | chr6 | + | 16612267 | 16612274 | 1.76e-05 | 0.24 | `CCACCTGC` |
| CCWCCTGC | chr6 | − | 26141523 | 26141530 | 1.76e-05 | 0.24 | `CCACCTGC` |
| CCWCCTGC | chr6 | + | 26152420 | 26152427 | 1.76e-05 | 0.24 | `CCACCTGC` |
| CCWCCTGC | chr6 | − | 31241047 | 31241054 | 1.76e-05 | 0.24 | `CCACCTGC` |
| CCWCCTGC | chr6 | + | 33058244 | 33058251 | 1.76e-05 | 0.24 | `CCACCTGC` |
| CCWCCTGC | chr6 | + | 35544291 | 35544298 | 1.76e-05 | 0.24 | `CCACCTGC` |
| CCWCCTGC | chr6 | − | 45996821 | 45996828 | 1.76e-05 | 0.24 | `CCACCTGC` |
| CCWCCTGC | chr6 | − | 74282245 | 74282252 | 1.76e-05 | 0.24 | `CCACCTGC` |
| CCWCCTGC | chr6 | − | 106312557 | 106312564 | 1.76e-05 | 0.24 | `CCACCTGC` |
| CCWCCTGC | chr6 | − | 106670082 | 106670089 | 1.76e-05 | 0.24 | `CCACCTGC` |
| CCWCCTGC | chr6 | − | 106700385 | 106700392 | 1.76e-05 | 0.24 | `CCACCTGC` |
| CCWCCTGC | chr6 | − | 135685703 | 135685710 | 1.76e-05 | 0.24 | `CCACCTGC` |
| CCWCCTGC | chr6 | + | 150011454 | 150011461 | 1.76e-05 | 0.24 | `CCACCTGC` |
| CCWCCTGC | chr6 | − | 152185463 | 152185470 | 1.76e-05 | 0.24 | `CCACCTGC` |
| CCWCCTGC | chr7 | − | 999499 | 999506 | 1.76e-05 | 0.24 | `CCACCTGC` |
| CCWCCTGC | chr7 | + | 2900970 | 2900977 | 1.76e-05 | 0.24 | `CCACCTGC` |
| CCWCCTGC | chr7 | + | 5701422 | 5701429 | 1.76e-05 | 0.24 | `CCACCTGC` |
| CCWCCTGC | chr7 | + | 7951097 | 7951104 | 1.76e-05 | 0.24 | `CCACCTGC` |
| CCWCCTGC | chr7 | − | 30637317 | 30637324 | 1.76e-05 | 0.24 | `CCACCTGC` |
| CCWCCTGC | chr7 | − | 44071092 | 44071099 | 1.76e-05 | 0.24 | `CCACCTGC` |
| CCWCCTGC | chr7 | − | 44072926 | 44072933 | 1.76e-05 | 0.24 | `CCACCTGC` |
| CCWCCTGC | chr7 | + | 73263466 | 73263473 | 1.76e-05 | 0.24 | `CCACCTGC` |
| CCWCCTGC | chr7 | + | 100019350 | 100019357 | 1.76e-05 | 0.24 | `CCACCTGC` |
| CCWCCTGC | chr7 | − | 101287067 | 101287074 | 1.76e-05 | 0.24 | `CCACCTGC` |
| CCWCCTGC | chr7 | + | 148952855 | 148952862 | 1.76e-05 | 0.24 | `CCACCTGC` |
| CCWCCTGC | chr8 | − | 21953314 | 21953321 | 1.76e-05 | 0.24 | `CCACCTGC` |
| CCWCCTGC | chr8 | − | 21953339 | 21953346 | 1.76e-05 | 0.24 | `CCACCTGC` |
| CCWCCTGC | chr8 | − | 22518297 | 22518304 | 1.76e-05 | 0.24 | `CCACCTGC` |
| CCWCCTGC | chr8 | + | 28803393 | 28803400 | 1.76e-05 | 0.24 | `CCACCTGC` |
| CCWCCTGC | chr8 | − | 28803813 | 28803820 | 1.76e-05 | 0.24 | `CCACCTGC` |
| CCWCCTGC | chr8 | + | 37543679 | 37543686 | 1.76e-05 | 0.24 | `CCACCTGC` |
| CCWCCTGC | chr8 | + | 72918714 | 72918721 | 1.76e-05 | 0.24 | `CCACCTGC` |
| CCWCCTGC | chr8 | − | 102218681 | 102218688 | 1.76e-05 | 0.24 | `CCACCTGC` |
| CCWCCTGC | chr8 | + | 125558032 | 125558039 | 1.76e-05 | 0.24 | `CCACCTGC` |
| CCWCCTGC | chr8 | + | 141668443 | 141668450 | 1.76e-05 | 0.24 | `CCACCTGC` |
| CCWCCTGC | chr9 | + | 6671073 | 6671080 | 1.76e-05 | 0.24 | `CCACCTGC` |
| CCWCCTGC | chr9 | − | 24922917 | 24922924 | 1.76e-05 | 0.24 | `CCACCTGC` |
| CCWCCTGC | chr9 | + | 70445449 | 70445456 | 1.76e-05 | 0.24 | `CCACCTGC` |
| CCWCCTGC | chr9 | + | 94866580 | 94866587 | 1.76e-05 | 0.24 | `CCACCTGC` |
| CCWCCTGC | chr9 | + | 122699467 | 122699474 | 1.76e-05 | 0.24 | `CCACCTGC` |
| CCWCCTGC | chr9 | − | 131690185 | 131690192 | 1.76e-05 | 0.24 | `CCACCTGC` |
| CCWCCTGC | chrX | + | 96468119 | 96468126 | 1.76e-05 | 0.24 | `CCACCTGC` |
| CCWCCTGC | chr10 | + | 73404461 | 73404468 | 1.76e-05 | 0.24 | `CCACCTGC` |
| CCWCCTGC | chr10 | + | 82002404 | 82002411 | 1.76e-05 | 0.24 | `CCACCTGC` |
| CCWCCTGC | chr10 | + | 112137980 | 112137987 | 1.76e-05 | 0.24 | `CCACCTGC` |
| CCWCCTGC | chr10 | + | 112164447 | 112164454 | 1.76e-05 | 0.24 | `CCACCTGC` |
| CCWCCTGC | chr10 | + | 120914769 | 120914776 | 1.76e-05 | 0.24 | `CCACCTGC` |
| CCWCCTGC | chr11 | − | 2378216 | 2378223 | 1.76e-05 | 0.24 | `CCACCTGC` |
| CCWCCTGC | chr11 | + | 33665991 | 33665998 | 1.76e-05 | 0.24 | `CCACCTGC` |
| CCWCCTGC | chr11 | − | 48088195 | 48088202 | 1.76e-05 | 0.24 | `CCACCTGC` |
| CCWCCTGC | chr11 | − | 64375521 | 64375528 | 1.76e-05 | 0.24 | `CCACCTGC` |
| CCWCCTGC | chr11 | − | 65098313 | 65098320 | 1.76e-05 | 0.24 | `CCACCTGC` |
| CCWCCTGC | chr11 | − | 75190060 | 75190067 | 1.76e-05 | 0.24 | `CCACCTGC` |
| CCWCCTGC | chr11 | − | 101693509 | 101693516 | 1.76e-05 | 0.24 | `CCACCTGC` |
| CCWCCTGC | chr11 | + | 103305584 | 103305591 | 1.76e-05 | 0.24 | `CCACCTGC` |
| CCWCCTGC | chr11 | − | 115372271 | 115372278 | 1.76e-05 | 0.24 | `CCACCTGC` |
| CCWCCTGC | chr11 | − | 127844427 | 127844434 | 1.76e-05 | 0.24 | `CCACCTGC` |
| CCWCCTGC | chr12 | − | 8692566 | 8692573 | 1.76e-05 | 0.24 | `CCACCTGC` |
| CCWCCTGC | chr12 | − | 11880569 | 11880576 | 1.76e-05 | 0.24 | `CCACCTGC` |
| CCWCCTGC | chr12 | + | 14325222 | 14325229 | 1.76e-05 | 0.24 | `CCACCTGC` |
| CCWCCTGC | chr12 | + | 46563643 | 46563650 | 1.76e-05 | 0.24 | `CCACCTGC` |
| CCWCCTGC | chr12 | + | 47810635 | 47810642 | 1.76e-05 | 0.24 | `CCACCTGC` |
| CCWCCTGC | chr12 | − | 52966001 | 52966008 | 1.76e-05 | 0.24 | `CCACCTGC` |
| CCWCCTGC | chr12 | − | 56462733 | 56462740 | 1.76e-05 | 0.24 | `CCACCTGC` |
| CCWCCTGC | chr12 | + | 91389723 | 91389730 | 1.76e-05 | 0.24 | `CCACCTGC` |
| CCWCCTGC | chr12 | + | 107486866 | 107486873 | 1.76e-05 | 0.24 | `CCACCTGC` |
| CCWCCTGC | chr12 | + | 122477634 | 122477641 | 1.76e-05 | 0.24 | `CCACCTGC` |
| CCWCCTGC | chr12 | − | 132123967 | 132123974 | 1.76e-05 | 0.24 | `CCACCTGC` |
| CCWCCTGC | chr13 | + | 112910910 | 112910917 | 1.76e-05 | 0.24 | `CCACCTGC` |
| CCWCCTGC | chr14 | + | 60169383 | 60169390 | 1.76e-05 | 0.24 | `CCACCTGC` |
| CCWCCTGC | chr14 | − | 61105193 | 61105200 | 1.76e-05 | 0.24 | `CCACCTGC` |
| CCWCCTGC | chr14 | + | 105306712 | 105306719 | 1.76e-05 | 0.24 | `CCACCTGC` |
| CCWCCTGC | chr14 | + | 105308935 | 105308942 | 1.76e-05 | 0.24 | `CCACCTGC` |
| CCWCCTGC | chr14 | + | 105399490 | 105399497 | 1.76e-05 | 0.24 | `CCACCTGC` |
| CCWCCTGC | chr15 | − | 57059030 | 57059037 | 1.76e-05 | 0.24 | `CCACCTGC` |
| CCWCCTGC | chr15 | + | 67537022 | 67537029 | 1.76e-05 | 0.24 | `CCACCTGC` |
| CCWCCTGC | chr15 | − | 73126637 | 73126644 | 1.76e-05 | 0.24 | `CCACCTGC` |
| CCWCCTGC | chr15 | − | 87679228 | 87679235 | 1.76e-05 | 0.24 | `CCACCTGC` |
| CCWCCTGC | chr16 | + | 28741333 | 28741340 | 1.76e-05 | 0.24 | `CCACCTGC` |
| CCWCCTGC | chr16 | − | 30377668 | 30377675 | 1.76e-05 | 0.24 | `CCACCTGC` |
| CCWCCTGC | chr16 | − | 51685742 | 51685749 | 1.76e-05 | 0.24 | `CCACCTGC` |
| CCWCCTGC | chr16 | − | 66666493 | 66666500 | 1.76e-05 | 0.24 | `CCACCTGC` |
| CCWCCTGC | chr16 | − | 82679990 | 82679997 | 1.76e-05 | 0.24 | `CCACCTGC` |
| CCWCCTGC | chr17 | + | 5283395 | 5283402 | 1.76e-05 | 0.24 | `CCACCTGC` |
| CCWCCTGC | chr17 | − | 35166248 | 35166255 | 1.76e-05 | 0.24 | `CCACCTGC` |
| CCWCCTGC | chr17 | − | 38024281 | 38024288 | 1.76e-05 | 0.24 | `CCACCTGC` |
| CCWCCTGC | chr17 | + | 38087340 | 38087347 | 1.76e-05 | 0.24 | `CCACCTGC` |
| CCWCCTGC | chr17 | + | 44645831 | 44645838 | 1.76e-05 | 0.24 | `CCACCTGC` |
| CCWCCTGC | chr17 | − | 44656630 | 44656637 | 1.76e-05 | 0.24 | `CCACCTGC` |
| CCWCCTGC | chr17 | − | 59173145 | 59173152 | 1.76e-05 | 0.24 | `CCACCTGC` |
| CCWCCTGC | chr17 | − | 73682277 | 73682284 | 1.76e-05 | 0.24 | `CCACCTGC` |
| CCWCCTGC | chr17 | + | 73684383 | 73684390 | 1.76e-05 | 0.24 | `CCACCTGC` |
| CCWCCTGC | chr17 | + | 73863979 | 73863986 | 1.76e-05 | 0.24 | `CCACCTGC` |
| CCWCCTGC | chr17 | + | 74226039 | 74226046 | 1.76e-05 | 0.24 | `CCACCTGC` |
| CCWCCTGC | chr17 | + | 78000940 | 78000947 | 1.76e-05 | 0.24 | `CCACCTGC` |
| CCWCCTGC | chr18 | + | 12857859 | 12857866 | 1.76e-05 | 0.24 | `CCACCTGC` |
| CCWCCTGC | chr18 | − | 58973913 | 58973920 | 1.76e-05 | 0.24 | `CCACCTGC` |
| CCWCCTGC | chr19 | + | 2513222 | 2513229 | 1.76e-05 | 0.24 | `CCACCTGC` |
| CCWCCTGC | chr19 | + | 5741826 | 5741833 | 1.76e-05 | 0.24 | `CCACCTGC` |
| CCWCCTGC | chr19 | − | 6724554 | 6724561 | 1.76e-05 | 0.24 | `CCACCTGC` |
| CCWCCTGC | chr19 | − | 12764542 | 12764549 | 1.76e-05 | 0.24 | `CCACCTGC` |
| CCWCCTGC | chr19 | − | 18070876 | 18070883 | 1.76e-05 | 0.24 | `CCACCTGC` |
| CCWCCTGC | chr19 | + | 38463287 | 38463294 | 1.76e-05 | 0.24 | `CCACCTGC` |
| CCWCCTGC | chr19 | + | 43228776 | 43228783 | 1.76e-05 | 0.24 | `CCACCTGC` |
| CCWCCTGC | chr19 | − | 49309291 | 49309298 | 1.76e-05 | 0.24 | `CCACCTGC` |
| CCWCCTGC | chr19 | − | 53450884 | 53450891 | 1.76e-05 | 0.24 | `CCACCTGC` |
| CCWCCTGC | chr20 | − | 36896424 | 36896431 | 1.76e-05 | 0.24 | `CCACCTGC` |
| CCWCCTGC | chr20 | + | 36904735 | 36904742 | 1.76e-05 | 0.24 | `CCACCTGC` |
| CCWCCTGC | chr20 | − | 36937539 | 36937546 | 1.76e-05 | 0.24 | `CCACCTGC` |
| CCWCCTGC | chr21 | + | 44451510 | 44451517 | 1.76e-05 | 0.24 | `CCACCTGC` |
| CCWCCTGC | chr22 | − | 16080162 | 16080169 | 1.76e-05 | 0.24 | `CCACCTGC` |
| CCWCCTGC | chr22 | + | 16119217 | 16119224 | 1.76e-05 | 0.24 | `CCACCTGC` |
| CCWCCTGC | chr22 | + | 20879969 | 20879976 | 1.76e-05 | 0.24 | `CCACCTGC` |
| CCWCCTGC | chr22 | + | 35587131 | 35587138 | 1.76e-05 | 0.24 | `CCACCTGC` |
| CCWCCTGC | chr22 | + | 39140967 | 39140974 | 1.76e-05 | 0.24 | `CCACCTGC` |
| CCWCCTGC | chr22 | − | 40558711 | 40558718 | 1.76e-05 | 0.24 | `CCACCTGC` |
| CCWCCTGC | chr22 | − | 41993801 | 41993808 | 1.76e-05 | 0.24 | `CCACCTGC` |
| CCWCCTGC | chr1 | − | 25099331 | 25099338 | 3.53e-05 | 0.24 | `CCTCCTGC` |
| CCWCCTGC | chr1 | − | 25749168 | 25749175 | 3.53e-05 | 0.24 | `CCTCCTGC` |
| CCWCCTGC | chr1 | − | 27825273 | 27825280 | 3.53e-05 | 0.24 | `CCTCCTGC` |
| CCWCCTGC | chr1 | + | 28374696 | 28374703 | 3.53e-05 | 0.24 | `CCTCCTGC` |
| CCWCCTGC | chr1 | + | 31000967 | 31000974 | 3.53e-05 | 0.24 | `CCTCCTGC` |
| CCWCCTGC | chr1 | + | 32488581 | 32488588 | 3.53e-05 | 0.24 | `CCTCCTGC` |
| CCWCCTGC | chr1 | + | 67913185 | 67913192 | 3.53e-05 | 0.24 | `CCTCCTGC` |
| CCWCCTGC | chr1 | + | 84745307 | 84745314 | 3.53e-05 | 0.24 | `CCTCCTGC` |
| CCWCCTGC | chr1 | − | 111560306 | 111560313 | 3.53e-05 | 0.24 | `CCTCCTGC` |
| CCWCCTGC | chr1 | − | 117976471 | 117976478 | 3.53e-05 | 0.24 | `CCTCCTGC` |
| CCWCCTGC | chr1 | − | 152185081 | 152185088 | 3.53e-05 | 0.24 | `CCTCCTGC` |
| CCWCCTGC | chr1 | − | 152185207 | 152185214 | 3.53e-05 | 0.24 | `CCTCCTGC` |
| CCWCCTGC | chr1 | − | 155413455 | 155413462 | 3.53e-05 | 0.24 | `CCTCCTGC` |
| CCWCCTGC | chr1 | − | 158865052 | 158865059 | 3.53e-05 | 0.24 | `CCTCCTGC` |
| CCWCCTGC | chr1 | − | 201525705 | 201525712 | 3.53e-05 | 0.24 | `CCTCCTGC` |
| CCWCCTGC | chr1 | + | 202697641 | 202697648 | 3.53e-05 | 0.24 | `CCTCCTGC` |
| CCWCCTGC | chr1 | + | 210172851 | 210172858 | 3.53e-05 | 0.24 | `CCTCCTGC` |
| CCWCCTGC | chr1 | + | 211222054 | 211222061 | 3.53e-05 | 0.24 | `CCTCCTGC` |
| CCWCCTGC | chr1 | + | 228844923 | 228844930 | 3.53e-05 | 0.24 | `CCTCCTGC` |
| CCWCCTGC | chr1 | − | 243201020 | 243201027 | 3.53e-05 | 0.24 | `CCTCCTGC` |
| CCWCCTGC | chr2 | − | 9699540 | 9699547 | 3.53e-05 | 0.24 | `CCTCCTGC` |
| CCWCCTGC | chr2 | − | 12115204 | 12115211 | 3.53e-05 | 0.24 | `CCTCCTGC` |
| CCWCCTGC | chr2 | + | 20270376 | 20270383 | 3.53e-05 | 0.24 | `CCTCCTGC` |
| CCWCCTGC | chr2 | + | 27157807 | 27157814 | 3.53e-05 | 0.24 | `CCTCCTGC` |
| CCWCCTGC | chr2 | − | 70223431 | 70223438 | 3.53e-05 | 0.24 | `CCTCCTGC` |
| CCWCCTGC | chr2 | − | 129273364 | 129273371 | 3.53e-05 | 0.24 | `CCTCCTGC` |
| CCWCCTGC | chr2 | − | 171884064 | 171884071 | 3.53e-05 | 0.24 | `CCTCCTGC` |
| CCWCCTGC | chr2 | − | 231233751 | 231233758 | 3.53e-05 | 0.24 | `CCTCCTGC` |
| CCWCCTGC | chr2 | − | 238014521 | 238014528 | 3.53e-05 | 0.24 | `CCTCCTGC` |
| CCWCCTGC | chr3 | − | 9413636 | 9413643 | 3.53e-05 | 0.24 | `CCTCCTGC` |
| CCWCCTGC | chr3 | + | 13100562 | 13100569 | 3.53e-05 | 0.24 | `CCTCCTGC` |
| CCWCCTGC | chr3 | − | 17168012 | 17168019 | 3.53e-05 | 0.24 | `CCTCCTGC` |
| CCWCCTGC | chr3 | + | 99724390 | 99724397 | 3.53e-05 | 0.24 | `CCTCCTGC` |
| CCWCCTGC | chr3 | + | 99733930 | 99733937 | 3.53e-05 | 0.24 | `CCTCCTGC` |
| CCWCCTGC | chr3 | + | 116347869 | 116347876 | 3.53e-05 | 0.24 | `CCTCCTGC` |
| CCWCCTGC | chr4 | + | 7529312 | 7529319 | 3.53e-05 | 0.24 | `CCTCCTGC` |
| CCWCCTGC | chr4 | + | 55127673 | 55127680 | 3.53e-05 | 0.24 | `CCTCCTGC` |
| CCWCCTGC | chr4 | + | 77340061 | 77340068 | 3.53e-05 | 0.24 | `CCTCCTGC` |
| CCWCCTGC | chr4 | − | 100135247 | 100135254 | 3.53e-05 | 0.24 | `CCTCCTGC` |
| CCWCCTGC | chr4 | + | 100135442 | 100135449 | 3.53e-05 | 0.24 | `CCTCCTGC` |
| CCWCCTGC | chr4 | + | 103967876 | 103967883 | 3.53e-05 | 0.24 | `CCTCCTGC` |
| CCWCCTGC | chr4 | − | 185440163 | 185440170 | 3.53e-05 | 0.24 | `CCTCCTGC` |
| CCWCCTGC | chr4 | − | 185440221 | 185440228 | 3.53e-05 | 0.24 | `CCTCCTGC` |
| CCWCCTGC | chr4 | + | 185505760 | 185505767 | 3.53e-05 | 0.24 | `CCTCCTGC` |
| CCWCCTGC | chr5 | + | 134268257 | 134268264 | 3.53e-05 | 0.24 | `CCTCCTGC` |
| CCWCCTGC | chr5 | − | 172395269 | 172395276 | 3.53e-05 | 0.24 | `CCTCCTGC` |
| CCWCCTGC | chr6 | + | 273574 | 273581 | 3.53e-05 | 0.24 | `CCTCCTGC` |
| CCWCCTGC | chr6 | − | 337279 | 337286 | 3.53e-05 | 0.24 | `CCTCCTGC` |
| CCWCCTGC | chr6 | + | 7830256 | 7830263 | 3.53e-05 | 0.24 | `CCTCCTGC` |
| CCWCCTGC | chr6 | + | 16529602 | 16529609 | 3.53e-05 | 0.24 | `CCTCCTGC` |
| CCWCCTGC | chr6 | + | 26139982 | 26139989 | 3.53e-05 | 0.24 | `CCTCCTGC` |
| CCWCCTGC | chr6 | − | 26153896 | 26153903 | 3.53e-05 | 0.24 | `CCTCCTGC` |
| CCWCCTGC | chr6 | + | 26305172 | 26305179 | 3.53e-05 | 0.24 | `CCTCCTGC` |
| CCWCCTGC | chr6 | + | 26358527 | 26358534 | 3.53e-05 | 0.24 | `CCTCCTGC` |
| CCWCCTGC | chr6 | − | 33486270 | 33486277 | 3.53e-05 | 0.24 | `CCTCCTGC` |
| CCWCCTGC | chr6 | + | 36743147 | 36743154 | 3.53e-05 | 0.24 | `CCTCCTGC` |
| CCWCCTGC | chr6 | + | 166667104 | 166667111 | 3.53e-05 | 0.24 | `CCTCCTGC` |
| CCWCCTGC | chr7 | + | 25954459 | 25954466 | 3.53e-05 | 0.24 | `CCTCCTGC` |
| CCWCCTGC | chr7 | − | 86942197 | 86942204 | 3.53e-05 | 0.24 | `CCTCCTGC` |
| CCWCCTGC | chr7 | − | 100019482 | 100019489 | 3.53e-05 | 0.24 | `CCTCCTGC` |
| CCWCCTGC | chr7 | + | 134504579 | 134504586 | 3.53e-05 | 0.24 | `CCTCCTGC` |
| CCWCCTGC | chr7 | − | 154733343 | 154733350 | 3.53e-05 | 0.24 | `CCTCCTGC` |
| CCWCCTGC | chr8 | + | 72918472 | 72918479 | 3.53e-05 | 0.24 | `CCTCCTGC` |
| CCWCCTGC | chr8 | − | 101947828 | 101947835 | 3.53e-05 | 0.24 | `CCTCCTGC` |
| CCWCCTGC | chr8 | − | 125718981 | 125718988 | 3.53e-05 | 0.24 | `CCTCCTGC` |
| CCWCCTGC | chr8 | − | 126412156 | 126412163 | 3.53e-05 | 0.24 | `CCTCCTGC` |
| CCWCCTGC | chr8 | + | 134564005 | 134564012 | 3.53e-05 | 0.24 | `CCTCCTGC` |
| CCWCCTGC | chr8 | + | 141668350 | 141668357 | 3.53e-05 | 0.24 | `CCTCCTGC` |
| CCWCCTGC | chr9 | + | 3516601 | 3516608 | 3.53e-05 | 0.24 | `CCTCCTGC` |
| CCWCCTGC | chr9 | − | 85942922 | 85942929 | 3.53e-05 | 0.24 | `CCTCCTGC` |
| CCWCCTGC | chr9 | + | 115319671 | 115319678 | 3.53e-05 | 0.24 | `CCTCCTGC` |
| CCWCCTGC | chr9 | − | 129247753 | 129247760 | 3.53e-05 | 0.24 | `CCTCCTGC` |
| CCWCCTGC | chr9 | + | 130458946 | 130458953 | 3.53e-05 | 0.24 | `CCTCCTGC` |
| CCWCCTGC | chr9 | + | 135205007 | 135205014 | 3.53e-05 | 0.24 | `CCTCCTGC` |
| CCWCCTGC | chrX | − | 38667850 | 38667857 | 3.53e-05 | 0.24 | `CCTCCTGC` |
| CCWCCTGC | chrX | + | 70759203 | 70759210 | 3.53e-05 | 0.24 | `CCTCCTGC` |
| CCWCCTGC | chr10 | + | 11328642 | 11328649 | 3.53e-05 | 0.24 | `CCTCCTGC` |
| CCWCCTGC | chr10 | − | 45235328 | 45235335 | 3.53e-05 | 0.24 | `CCTCCTGC` |
| CCWCCTGC | chr10 | − | 70495660 | 70495667 | 3.53e-05 | 0.24 | `CCTCCTGC` |
| CCWCCTGC | chr10 | + | 73727618 | 73727625 | 3.53e-05 | 0.24 | `CCTCCTGC` |
| CCWCCTGC | chr10 | + | 82002503 | 82002510 | 3.53e-05 | 0.24 | `CCTCCTGC` |
| CCWCCTGC | chr10 | + | 82002539 | 82002546 | 3.53e-05 | 0.24 | `CCTCCTGC` |
| CCWCCTGC | chr10 | + | 82002611 | 82002618 | 3.53e-05 | 0.24 | `CCTCCTGC` |
| CCWCCTGC | chr10 | + | 82002683 | 82002690 | 3.53e-05 | 0.24 | `CCTCCTGC` |
| CCWCCTGC | chr10 | + | 82002755 | 82002762 | 3.53e-05 | 0.24 | `CCTCCTGC` |
| CCWCCTGC | chr10 | + | 104410889 | 104410896 | 3.53e-05 | 0.24 | `CCTCCTGC` |
| CCWCCTGC | chr10 | + | 125969096 | 125969103 | 3.53e-05 | 0.24 | `CCTCCTGC` |
| CCWCCTGC | chr11 | + | 35105746 | 35105753 | 3.53e-05 | 0.24 | `CCTCCTGC` |
| CCWCCTGC | chr11 | + | 59979535 | 59979542 | 3.53e-05 | 0.24 | `CCTCCTGC` |
| CCWCCTGC | chr11 | + | 65076802 | 65076809 | 3.53e-05 | 0.24 | `CCTCCTGC` |
| CCWCCTGC | chr11 | + | 65384184 | 65384191 | 3.53e-05 | 0.24 | `CCTCCTGC` |
| CCWCCTGC | chr11 | + | 72531335 | 72531342 | 3.53e-05 | 0.24 | `CCTCCTGC` |
| CCWCCTGC | chr11 | − | 72766015 | 72766022 | 3.53e-05 | 0.24 | `CCTCCTGC` |
| CCWCCTGC | chr11 | + | 110735298 | 110735305 | 3.53e-05 | 0.24 | `CCTCCTGC` |
| CCWCCTGC | chr11 | + | 117786429 | 117786436 | 3.53e-05 | 0.24 | `CCTCCTGC` |
| CCWCCTGC | chr12 | + | 6938620 | 6938627 | 3.53e-05 | 0.24 | `CCTCCTGC` |
| CCWCCTGC | chr12 | − | 9744081 | 9744088 | 3.53e-05 | 0.24 | `CCTCCTGC` |
| CCWCCTGC | chr12 | − | 22588525 | 22588532 | 3.53e-05 | 0.24 | `CCTCCTGC` |
| CCWCCTGC | chr12 | + | 46493447 | 46493454 | 3.53e-05 | 0.24 | `CCTCCTGC` |
| CCWCCTGC | chr12 | − | 91906123 | 91906130 | 3.53e-05 | 0.24 | `CCTCCTGC` |
| CCWCCTGC | chr12 | − | 99906256 | 99906263 | 3.53e-05 | 0.24 | `CCTCCTGC` |
| CCWCCTGC | chr12 | + | 102847101 | 102847108 | 3.53e-05 | 0.24 | `CCTCCTGC` |
| CCWCCTGC | chr12 | − | 107556187 | 107556194 | 3.53e-05 | 0.24 | `CCTCCTGC` |
| CCWCCTGC | chr12 | − | 121914810 | 121914817 | 3.53e-05 | 0.24 | `CCTCCTGC` |
| CCWCCTGC | chr13 | − | 112910845 | 112910852 | 3.53e-05 | 0.24 | `CCTCCTGC` |
| CCWCCTGC | chr14 | − | 49598284 | 49598291 | 3.53e-05 | 0.24 | `CCTCCTGC` |
| CCWCCTGC | chr14 | − | 49598434 | 49598441 | 3.53e-05 | 0.24 | `CCTCCTGC` |
| CCWCCTGC | chr14 | + | 50358431 | 50358438 | 3.53e-05 | 0.24 | `CCTCCTGC` |
| CCWCCTGC | chr14 | − | 54638803 | 54638810 | 3.53e-05 | 0.24 | `CCTCCTGC` |
| CCWCCTGC | chr14 | − | 105308435 | 105308442 | 3.53e-05 | 0.24 | `CCTCCTGC` |
| CCWCCTGC | chr14 | + | 106218767 | 106218774 | 3.53e-05 | 0.24 | `CCTCCTGC` |
| CCWCCTGC | chr15 | − | 19248885 | 19248892 | 3.53e-05 | 0.24 | `CCTCCTGC` |
| CCWCCTGC | chr15 | − | 29567975 | 29567982 | 3.53e-05 | 0.24 | `CCTCCTGC` |
| CCWCCTGC | chr15 | + | 38191431 | 38191438 | 3.53e-05 | 0.24 | `CCTCCTGC` |
| CCWCCTGC | chr15 | + | 61582679 | 61582686 | 3.53e-05 | 0.24 | `CCTCCTGC` |
| CCWCCTGC | chr15 | − | 62466953 | 62466960 | 3.53e-05 | 0.24 | `CCTCCTGC` |
| CCWCCTGC | chr15 | − | 73017339 | 73017346 | 3.53e-05 | 0.24 | `CCTCCTGC` |
| CCWCCTGC | chr15 | + | 73126451 | 73126458 | 3.53e-05 | 0.24 | `CCTCCTGC` |
| CCWCCTGC | chr15 | + | 73294217 | 73294224 | 3.53e-05 | 0.24 | `CCTCCTGC` |
| CCWCCTGC | chr15 | + | 91184160 | 91184167 | 3.53e-05 | 0.24 | `CCTCCTGC` |
| CCWCCTGC | chr16 | − | 1981187 | 1981194 | 3.53e-05 | 0.24 | `CCTCCTGC` |
| CCWCCTGC | chr16 | − | 2195264 | 2195271 | 3.53e-05 | 0.24 | `CCTCCTGC` |
| CCWCCTGC | chr16 | + | 2672395 | 2672402 | 3.53e-05 | 0.24 | `CCTCCTGC` |
| CCWCCTGC | chr16 | − | 31098946 | 31098953 | 3.53e-05 | 0.24 | `CCTCCTGC` |
| CCWCCTGC | chr17 | − | 1891814 | 1891821 | 3.53e-05 | 0.24 | `CCTCCTGC` |
| CCWCCTGC | chr17 | + | 39936314 | 39936321 | 3.53e-05 | 0.24 | `CCTCCTGC` |
| CCWCCTGC | chr17 | + | 43168282 | 43168289 | 3.53e-05 | 0.24 | `CCTCCTGC` |
| CCWCCTGC | chr17 | − | 59173132 | 59173139 | 3.53e-05 | 0.24 | `CCTCCTGC` |
| CCWCCTGC | chr17 | − | 63746870 | 63746877 | 3.53e-05 | 0.24 | `CCTCCTGC` |
| CCWCCTGC | chr17 | + | 63799734 | 63799741 | 3.53e-05 | 0.24 | `CCTCCTGC` |
| CCWCCTGC | chr17 | + | 73636818 | 73636825 | 3.53e-05 | 0.24 | `CCTCCTGC` |
| CCWCCTGC | chr17 | + | 73636818 | 73636825 | 3.53e-05 | 0.24 | `CCTCCTGC` |
| CCWCCTGC | chr17 | + | 74225837 | 74225844 | 3.53e-05 | 0.24 | `CCTCCTGC` |
| CCWCCTGC | chr18 | + | 11150092 | 11150099 | 3.53e-05 | 0.24 | `CCTCCTGC` |
| CCWCCTGC | chr18 | − | 19735210 | 19735217 | 3.53e-05 | 0.24 | `CCTCCTGC` |
| CCWCCTGC | chr18 | − | 45272782 | 45272789 | 3.53e-05 | 0.24 | `CCTCCTGC` |
| CCWCCTGC | chr19 | + | 4293743 | 4293750 | 3.53e-05 | 0.24 | `CCTCCTGC` |
| CCWCCTGC | chr19 | − | 4675552 | 4675559 | 3.53e-05 | 0.24 | `CCTCCTGC` |
| CCWCCTGC | chr19 | − | 6542039 | 6542046 | 3.53e-05 | 0.24 | `CCTCCTGC` |
| CCWCCTGC | chr19 | + | 7672798 | 7672805 | 3.53e-05 | 0.24 | `CCTCCTGC` |
| CCWCCTGC | chr19 | − | 17495324 | 17495331 | 3.53e-05 | 0.24 | `CCTCCTGC` |
| CCWCCTGC | chr19 | + | 40900245 | 40900252 | 3.53e-05 | 0.24 | `CCTCCTGC` |
| CCWCCTGC | chr19 | − | 44586480 | 44586487 | 3.53e-05 | 0.24 | `CCTCCTGC` |
| CCWCCTGC | chr19 | − | 47080260 | 47080267 | 3.53e-05 | 0.24 | `CCTCCTGC` |
| CCWCCTGC | chr19 | − | 47141714 | 47141721 | 3.53e-05 | 0.24 | `CCTCCTGC` |
| CCWCCTGC | chr19 | − | 48951124 | 48951131 | 3.53e-05 | 0.24 | `CCTCCTGC` |
| CCWCCTGC | chr19 | − | 52940635 | 52940642 | 3.53e-05 | 0.24 | `CCTCCTGC` |
| CCWCCTGC | chr19 | + | 53814220 | 53814227 | 3.53e-05 | 0.24 | `CCTCCTGC` |
| CCWCCTGC | chr19 | − | 55571672 | 55571679 | 3.53e-05 | 0.24 | `CCTCCTGC` |
| CCWCCTGC | chr20 | − | 5041828 | 5041835 | 3.53e-05 | 0.24 | `CCTCCTGC` |
| CCWCCTGC | chr20 | − | 29760161 | 29760168 | 3.53e-05 | 0.24 | `CCTCCTGC` |
| CCWCCTGC | chr20 | + | 31452831 | 31452838 | 3.53e-05 | 0.24 | `CCTCCTGC` |
| CCWCCTGC | chr20 | − | 33753582 | 33753589 | 3.53e-05 | 0.24 | `CCTCCTGC` |
| CCWCCTGC | chr21 | − | 39643465 | 39643472 | 3.53e-05 | 0.24 | `CCTCCTGC` |
| CCWCCTGC | chr21 | − | 44388064 | 44388071 | 3.53e-05 | 0.24 | `CCTCCTGC` |
| CCWCCTGC | chr22 | + | 20880306 | 20880313 | 3.53e-05 | 0.24 | `CCTCCTGC` |
| CCWCCTGC | chr22 | + | 20920197 | 20920204 | 3.53e-05 | 0.24 | `CCTCCTGC` |
| CCWCCTGC | chr22 | − | 25336570 | 25336577 | 3.53e-05 | 0.24 | `CCTCCTGC` |
| CCWCCTGC | chr22 | + | 41993795 | 41993802 | 3.53e-05 | 0.24 | `CCTCCTGC` |
| CCWCCTGC | chr22 | + | 49311562 | 49311569 | 3.53e-05 | 0.24 | `CCTCCTGC` |
| CCWCCTGC | chr1 | + | 16034130 | 16034137 | 7.33e-05 | 0.307 | `CCCCCTGC` |
| CCWCCTGC | chr1 | − | 25221623 | 25221630 | 7.33e-05 | 0.307 | `CCCCCTGC` |
| CCWCCTGC | chr1 | + | 26010053 | 26010060 | 7.33e-05 | 0.307 | `CCCCCTGC` |
| CCWCCTGC | chr1 | − | 26819502 | 26819509 | 7.33e-05 | 0.307 | `CCCCCTGC` |
| CCWCCTGC | chr1 | + | 28398990 | 28398997 | 7.33e-05 | 0.307 | `CCCCCTGC` |
| CCWCCTGC | chr1 | + | 28709132 | 28709139 | 7.33e-05 | 0.307 | `CCCCCTGC` |
| CCWCCTGC | chr1 | − | 30993052 | 30993059 | 7.33e-05 | 0.307 | `CCCCCTGC` |
| CCWCCTGC | chr1 | + | 31669149 | 31669156 | 7.33e-05 | 0.307 | `CCCCCTGC` |
| CCWCCTGC | chr1 | − | 33055822 | 33055829 | 7.33e-05 | 0.307 | `CCCCCTGC` |
| CCWCCTGC | chr1 | − | 93070079 | 93070086 | 7.33e-05 | 0.307 | `CCCCCTGC` |
| CCWCCTGC | chr1 | + | 147490257 | 147490264 | 7.33e-05 | 0.307 | `CCGCCTGC` |
| CCWCCTGC | chr1 | + | 149851133 | 149851140 | 7.33e-05 | 0.307 | `CCCCCTGC` |
| CCWCCTGC | chr1 | − | 154449384 | 154449391 | 7.33e-05 | 0.307 | `CCGCCTGC` |
| CCWCCTGC | chr1 | + | 154452965 | 154452972 | 7.33e-05 | 0.307 | `CCGCCTGC` |
| CCWCCTGC | chr1 | + | 171646807 | 171646814 | 7.33e-05 | 0.307 | `CCCCCTGC` |
| CCWCCTGC | chr1 | + | 172101701 | 172101708 | 7.33e-05 | 0.307 | `CCCCCTGC` |
| CCWCCTGC | chr1 | − | 180663165 | 180663172 | 7.33e-05 | 0.307 | `CCCCCTGC` |
| CCWCCTGC | chr1 | + | 243201261 | 243201268 | 7.33e-05 | 0.307 | `CCGCCTGC` |
| CCWCCTGC | chr2 | + | 3600542 | 3600549 | 7.33e-05 | 0.307 | `CCGCCTGC` |
| CCWCCTGC | chr2 | − | 37735857 | 37735864 | 7.33e-05 | 0.307 | `CCGCCTGC` |
| CCWCCTGC | chr2 | − | 70167480 | 70167487 | 7.33e-05 | 0.307 | `CCGCCTGC` |
| CCWCCTGC | chr2 | − | 74553323 | 74553330 | 7.33e-05 | 0.307 | `CCGCCTGC` |
| CCWCCTGC | chr2 | + | 125872893 | 125872900 | 7.33e-05 | 0.307 | `CCCCCTGC` |
| CCWCCTGC | chr2 | − | 177837888 | 177837895 | 7.33e-05 | 0.307 | `CCGCCTGC` |
| CCWCCTGC | chr2 | − | 178125563 | 178125570 | 7.33e-05 | 0.307 | `CCGCCTGC` |
| CCWCCTGC | chr2 | + | 201689873 | 201689880 | 7.33e-05 | 0.307 | `CCCCCTGC` |
| CCWCCTGC | chr2 | − | 203812191 | 203812198 | 7.33e-05 | 0.307 | `CCCCCTGC` |
| CCWCCTGC | chr2 | + | 231557746 | 231557753 | 7.33e-05 | 0.307 | `CCCCCTGC` |
| CCWCCTGC | chr2 | + | 232286944 | 232286951 | 7.33e-05 | 0.307 | `CCCCCTGC` |
| CCWCCTGC | chr2 | + | 235074107 | 235074114 | 7.33e-05 | 0.307 | `CCCCCTGC` |
| CCWCCTGC | chr3 | + | 9413094 | 9413101 | 7.33e-05 | 0.307 | `CCCCCTGC` |
| CCWCCTGC | chr3 | − | 9413544 | 9413551 | 7.33e-05 | 0.307 | `CCGCCTGC` |
| CCWCCTGC | chr3 | + | 10209720 | 10209727 | 7.33e-05 | 0.307 | `CCCCCTGC` |
| CCWCCTGC | chr3 | − | 12912760 | 12912767 | 7.33e-05 | 0.307 | `CCCCCTGC` |
| CCWCCTGC | chr3 | − | 23933469 | 23933476 | 7.33e-05 | 0.307 | `CCCCCTGC` |
| CCWCCTGC | chr3 | + | 23934060 | 23934067 | 7.33e-05 | 0.307 | `CCGCCTGC` |
| CCWCCTGC | chr3 | − | 49034483 | 49034490 | 7.33e-05 | 0.307 | `CCCCCTGC` |
| CCWCCTGC | chr3 | − | 130363119 | 130363126 | 7.33e-05 | 0.307 | `CCGCCTGC` |
| CCWCCTGC | chr3 | − | 157874940 | 157874947 | 7.33e-05 | 0.307 | `CCCCCTGC` |
| CCWCCTGC | chr3 | − | 158017546 | 158017553 | 7.33e-05 | 0.307 | `CCGCCTGC` |
| CCWCCTGC | chr3 | + | 184362452 | 184362459 | 7.33e-05 | 0.307 | `CCCCCTGC` |
| CCWCCTGC | chr4 | − | 7824419 | 7824426 | 7.33e-05 | 0.307 | `CCGCCTGC` |
| CCWCCTGC | chr5 | + | 53152662 | 53152669 | 7.33e-05 | 0.307 | `CCGCCTGC` |
| CCWCCTGC | chr5 | − | 75179065 | 75179072 | 7.33e-05 | 0.307 | `CCCCCTGC` |
| CCWCCTGC | chr5 | − | 96297402 | 96297409 | 7.33e-05 | 0.307 | `CCCCCTGC` |
| CCWCCTGC | chr5 | + | 131830295 | 131830302 | 7.33e-05 | 0.307 | `CCGCCTGC` |
| CCWCCTGC | chr5 | + | 138748224 | 138748231 | 7.33e-05 | 0.307 | `CCCCCTGC` |
| CCWCCTGC | chr5 | − | 139030048 | 139030055 | 7.33e-05 | 0.307 | `CCGCCTGC` |
| CCWCCTGC | chr5 | − | 149768283 | 149768290 | 7.33e-05 | 0.307 | `CCCCCTGC` |
| CCWCCTGC | chr5 | + | 176804610 | 176804617 | 7.33e-05 | 0.307 | `CCCCCTGC` |
| CCWCCTGC | chr6 | + | 237779 | 237786 | 7.33e-05 | 0.307 | `CCGCCTGC` |
| CCWCCTGC | chr6 | + | 250661 | 250668 | 7.33e-05 | 0.307 | `CCCCCTGC` |
| CCWCCTGC | chr6 | + | 21695882 | 21695889 | 7.33e-05 | 0.307 | `CCGCCTGC` |
| CCWCCTGC | chr6 | − | 21696647 | 21696654 | 7.33e-05 | 0.307 | `CCCCCTGC` |
| CCWCCTGC | chr6 | − | 21696647 | 21696654 | 7.33e-05 | 0.307 | `CCCCCTGC` |
| CCWCCTGC | chr6 | − | 24828339 | 24828346 | 7.33e-05 | 0.307 | `CCCCCTGC` |
| CCWCCTGC | chr6 | + | 26129769 | 26129776 | 7.33e-05 | 0.307 | `CCGCCTGC` |
| CCWCCTGC | chr6 | − | 26141673 | 26141680 | 7.33e-05 | 0.307 | `CCGCCTGC` |
| CCWCCTGC | chr6 | + | 27208925 | 27208932 | 7.33e-05 | 0.307 | `CCGCCTGC` |
| CCWCCTGC | chr6 | + | 27222982 | 27222989 | 7.33e-05 | 0.307 | `CCGCCTGC` |
| CCWCCTGC | chr6 | − | 27968804 | 27968811 | 7.33e-05 | 0.307 | `CCGCCTGC` |
| CCWCCTGC | chr6 | + | 30796691 | 30796698 | 7.33e-05 | 0.307 | `CCCCCTGC` |
| CCWCCTGC | chr6 | − | 33375184 | 33375191 | 7.33e-05 | 0.307 | `CCCCCTGC` |
| CCWCCTGC | chr6 | + | 36761699 | 36761706 | 7.33e-05 | 0.307 | `CCCCCTGC` |
| CCWCCTGC | chr6 | − | 42822018 | 42822025 | 7.33e-05 | 0.307 | `CCGCCTGC` |
| CCWCCTGC | chr6 | − | 106656530 | 106656537 | 7.33e-05 | 0.307 | `CCCCCTGC` |
| CCWCCTGC | chr6 | − | 133177463 | 133177470 | 7.33e-05 | 0.307 | `CCCCCTGC` |
| CCWCCTGC | chr6 | − | 166667159 | 166667166 | 7.33e-05 | 0.307 | `CCCCCTGC` |
| CCWCCTGC | chr7 | − | 999368 | 999375 | 7.33e-05 | 0.307 | `CCCCCTGC` |
| CCWCCTGC | chr7 | − | 44802603 | 44802610 | 7.33e-05 | 0.307 | `CCGCCTGC` |
| CCWCCTGC | chr7 | + | 73270250 | 73270257 | 7.33e-05 | 0.307 | `CCGCCTGC` |
| CCWCCTGC | chr7 | − | 75515504 | 75515511 | 7.33e-05 | 0.307 | `CCGCCTGC` |
| CCWCCTGC | chr7 | − | 101719344 | 101719351 | 7.33e-05 | 0.307 | `CCCCCTGC` |
| CCWCCTGC | chr7 | − | 101853251 | 101853258 | 7.33e-05 | 0.307 | `CCGCCTGC` |
| CCWCCTGC | chr7 | + | 128362299 | 128362306 | 7.33e-05 | 0.307 | `CCCCCTGC` |
| CCWCCTGC | chr7 | + | 139263106 | 139263113 | 7.33e-05 | 0.307 | `CCGCCTGC` |
| CCWCCTGC | chr7 | − | 148952677 | 148952684 | 7.33e-05 | 0.307 | `CCGCCTGC` |
| CCWCCTGC | chr8 | + | 6553521 | 6553528 | 7.33e-05 | 0.307 | `CCGCCTGC` |
| CCWCCTGC | chr8 | − | 6553565 | 6553572 | 7.33e-05 | 0.307 | `CCGCCTGC` |
| CCWCCTGC | chr8 | − | 10860479 | 10860486 | 7.33e-05 | 0.307 | `CCCCCTGC` |
| CCWCCTGC | chr8 | + | 22518314 | 22518321 | 7.33e-05 | 0.307 | `CCCCCTGC` |
| CCWCCTGC | chr8 | − | 29253954 | 29253961 | 7.33e-05 | 0.307 | `CCCCCTGC` |
| CCWCCTGC | chr8 | + | 57149817 | 57149824 | 7.33e-05 | 0.307 | `CCGCCTGC` |
| CCWCCTGC | chr8 | − | 61985205 | 61985212 | 7.33e-05 | 0.307 | `CCGCCTGC` |
| CCWCCTGC | chr8 | − | 90839384 | 90839391 | 7.33e-05 | 0.307 | `CCCCCTGC` |
| CCWCCTGC | chr8 | − | 101576497 | 101576504 | 7.33e-05 | 0.307 | `CCCCCTGC` |
| CCWCCTGC | chr8 | − | 141668362 | 141668369 | 7.33e-05 | 0.307 | `CCGCCTGC` |
| CCWCCTGC | chr8 | − | 142198397 | 142198404 | 7.33e-05 | 0.307 | `CCCCCTGC` |
| CCWCCTGC | chr9 | − | 6403009 | 6403016 | 7.33e-05 | 0.307 | `CCGCCTGC` |
| CCWCCTGC | chr9 | + | 36141773 | 36141780 | 7.33e-05 | 0.307 | `CCCCCTGC` |
| CCWCCTGC | chr9 | − | 94866788 | 94866795 | 7.33e-05 | 0.307 | `CCCCCTGC` |
| CCWCCTGC | chr9 | + | 99213923 | 99213930 | 7.33e-05 | 0.307 | `CCCCCTGC` |
| CCWCCTGC | chrX | − | 24078589 | 24078596 | 7.33e-05 | 0.307 | `CCGCCTGC` |
| CCWCCTGC | chrX | + | 48679442 | 48679449 | 7.33e-05 | 0.307 | `CCCCCTGC` |
| CCWCCTGC | chr10 | − | 3817587 | 3817594 | 7.33e-05 | 0.307 | `CCGCCTGC` |
| CCWCCTGC | chr10 | + | 7009926 | 7009933 | 7.33e-05 | 0.307 | `CCGCCTGC` |
| CCWCCTGC | chr10 | − | 35455818 | 35455825 | 7.33e-05 | 0.307 | `CCCCCTGC` |
| CCWCCTGC | chr10 | − | 73761786 | 73761793 | 7.33e-05 | 0.307 | `CCCCCTGC` |
| CCWCCTGC | chr10 | + | 104411227 | 104411234 | 7.33e-05 | 0.307 | `CCCCCTGC` |
| CCWCCTGC | chr11 | + | 7512013 | 7512020 | 7.33e-05 | 0.307 | `CCCCCTGC` |
| CCWCCTGC | chr11 | − | 61292581 | 61292588 | 7.33e-05 | 0.307 | `CCCCCTGC` |
| CCWCCTGC | chr11 | − | 62146020 | 62146027 | 7.33e-05 | 0.307 | `CCGCCTGC` |
| CCWCCTGC | chr11 | − | 62177338 | 62177345 | 7.33e-05 | 0.307 | `CCGCCTGC` |
| CCWCCTGC | chr11 | + | 62329588 | 62329595 | 7.33e-05 | 0.307 | `CCCCCTGC` |
| CCWCCTGC | chr11 | + | 63750883 | 63750890 | 7.33e-05 | 0.307 | `CCGCCTGC` |
| CCWCCTGC | chr11 | − | 64620178 | 64620185 | 7.33e-05 | 0.307 | `CCCCCTGC` |
| CCWCCTGC | chr11 | + | 64620897 | 64620904 | 7.33e-05 | 0.307 | `CCCCCTGC` |
| CCWCCTGC | chr11 | + | 64650898 | 64650905 | 7.33e-05 | 0.307 | `CCCCCTGC` |
| CCWCCTGC | chr11 | − | 65112228 | 65112235 | 7.33e-05 | 0.307 | `CCCCCTGC` |
| CCWCCTGC | chr11 | − | 65443660 | 65443667 | 7.33e-05 | 0.307 | `CCGCCTGC` |
| CCWCCTGC | chr11 | − | 69209769 | 69209776 | 7.33e-05 | 0.307 | `CCCCCTGC` |
| CCWCCTGC | chr12 | + | 6513889 | 6513896 | 7.33e-05 | 0.307 | `CCGCCTGC` |
| CCWCCTGC | chr12 | − | 54798335 | 54798342 | 7.33e-05 | 0.307 | `CCGCCTGC` |
| CCWCCTGC | chr12 | + | 93480264 | 93480271 | 7.33e-05 | 0.307 | `CCGCCTGC` |
| CCWCCTGC | chr12 | + | 107551753 | 107551760 | 7.33e-05 | 0.307 | `CCCCCTGC` |
| CCWCCTGC | chr12 | + | 109583748 | 109583755 | 7.33e-05 | 0.307 | `CCCCCTGC` |
| CCWCCTGC | chr12 | + | 119215435 | 119215442 | 7.33e-05 | 0.307 | `CCCCCTGC` |
| CCWCCTGC | chr12 | − | 123967880 | 123967887 | 7.33e-05 | 0.307 | `CCGCCTGC` |
| CCWCCTGC | chr14 | − | 23700453 | 23700460 | 7.33e-05 | 0.307 | `CCCCCTGC` |
| CCWCCTGC | chr14 | − | 23700733 | 23700740 | 7.33e-05 | 0.307 | `CCCCCTGC` |
| CCWCCTGC | chr14 | − | 68329691 | 68329698 | 7.33e-05 | 0.307 | `CCCCCTGC` |
| CCWCCTGC | chr14 | + | 68330928 | 68330935 | 7.33e-05 | 0.307 | `CCCCCTGC` |
| CCWCCTGC | chr14 | + | 70856754 | 70856761 | 7.33e-05 | 0.307 | `CCGCCTGC` |
| CCWCCTGC | chr14 | − | 89932975 | 89932982 | 7.33e-05 | 0.307 | `CCCCCTGC` |
| CCWCCTGC | chr14 | − | 89933184 | 89933191 | 7.33e-05 | 0.307 | `CCGCCTGC` |
| CCWCCTGC | chr14 | + | 104583100 | 104583107 | 7.33e-05 | 0.307 | `CCGCCTGC` |
| CCWCCTGC | chr14 | + | 106218845 | 106218852 | 7.33e-05 | 0.307 | `CCCCCTGC` |
| CCWCCTGC | chr14 | + | 106218883 | 106218890 | 7.33e-05 | 0.307 | `CCCCCTGC` |
| CCWCCTGC | chr14 | + | 106218961 | 106218968 | 7.33e-05 | 0.307 | `CCCCCTGC` |
| CCWCCTGC | chr14 | + | 106219098 | 106219105 | 7.33e-05 | 0.307 | `CCCCCTGC` |
| CCWCCTGC | chr15 | − | 29441066 | 29441073 | 7.33e-05 | 0.307 | `CCCCCTGC` |
| CCWCCTGC | chr15 | + | 38013464 | 38013471 | 7.33e-05 | 0.307 | `CCCCCTGC` |
| CCWCCTGC | chr15 | − | 43534418 | 43534425 | 7.33e-05 | 0.307 | `CCCCCTGC` |
| CCWCCTGC | chr15 | − | 66874746 | 66874753 | 7.33e-05 | 0.307 | `CCGCCTGC` |
| CCWCCTGC | chr15 | − | 68094614 | 68094621 | 7.33e-05 | 0.307 | `CCCCCTGC` |
| CCWCCTGC | chr15 | + | 70310691 | 70310698 | 7.33e-05 | 0.307 | `CCCCCTGC` |
| CCWCCTGC | chr15 | + | 91244681 | 91244688 | 7.33e-05 | 0.307 | `CCCCCTGC` |
| CCWCCTGC | chr15 | + | 99493525 | 99493532 | 7.33e-05 | 0.307 | `CCCCCTGC` |
| CCWCCTGC | chr15 | + | 99652870 | 99652877 | 7.33e-05 | 0.307 | `CCGCCTGC` |
| CCWCCTGC | chr16 | + | 11330321 | 11330328 | 7.33e-05 | 0.307 | `CCCCCTGC` |
| CCWCCTGC | chr16 | − | 18720634 | 18720641 | 7.33e-05 | 0.307 | `CCCCCTGC` |
| CCWCCTGC | chr16 | − | 19474186 | 19474193 | 7.33e-05 | 0.307 | `CCGCCTGC` |
| CCWCCTGC | chr16 | + | 22216108 | 22216115 | 7.33e-05 | 0.307 | `CCCCCTGC` |
| CCWCCTGC | chr16 | + | 28741530 | 28741537 | 7.33e-05 | 0.307 | `CCCCCTGC` |
| CCWCCTGC | chr16 | − | 28765372 | 28765379 | 7.33e-05 | 0.307 | `CCCCCTGC` |
| CCWCCTGC | chr16 | + | 29974022 | 29974029 | 7.33e-05 | 0.307 | `CCCCCTGC` |
| CCWCCTGC | chr16 | + | 29974166 | 29974173 | 7.33e-05 | 0.307 | `CCCCCTGC` |
| CCWCCTGC | chr16 | + | 31098876 | 31098883 | 7.33e-05 | 0.307 | `CCCCCTGC` |
| CCWCCTGC | chr16 | + | 55523226 | 55523233 | 7.33e-05 | 0.307 | `CCCCCTGC` |
| CCWCCTGC | chr16 | − | 66666610 | 66666617 | 7.33e-05 | 0.307 | `CCCCCTGC` |
| CCWCCTGC | chr16 | − | 68972609 | 68972616 | 7.33e-05 | 0.307 | `CCGCCTGC` |
| CCWCCTGC | chr16 | + | 86542385 | 86542392 | 7.33e-05 | 0.307 | `CCGCCTGC` |
| CCWCCTGC | chr16 | − | 87245477 | 87245484 | 7.33e-05 | 0.307 | `CCCCCTGC` |
| CCWCCTGC | chr17 | + | 1048144 | 1048151 | 7.33e-05 | 0.307 | `CCCCCTGC` |
| CCWCCTGC | chr17 | − | 7328317 | 7328324 | 7.33e-05 | 0.307 | `CCGCCTGC` |
| CCWCCTGC | chr17 | − | 7416781 | 7416788 | 7.33e-05 | 0.307 | `CCGCCTGC` |
| CCWCCTGC | chr17 | + | 24303370 | 24303377 | 7.33e-05 | 0.307 | `CCGCCTGC` |
| CCWCCTGC | chr17 | − | 33853682 | 33853689 | 7.33e-05 | 0.307 | `CCGCCTGC` |
| CCWCCTGC | chr17 | + | 35166217 | 35166224 | 7.33e-05 | 0.307 | `CCCCCTGC` |
| CCWCCTGC | chr17 | − | 36009043 | 36009050 | 7.33e-05 | 0.307 | `CCCCCTGC` |
| CCWCCTGC | chr17 | + | 44656587 | 44656594 | 7.33e-05 | 0.307 | `CCCCCTGC` |
| CCWCCTGC | chr17 | − | 44656593 | 44656600 | 7.33e-05 | 0.307 | `CCGCCTGC` |
| CCWCCTGC | chr17 | − | 53784673 | 53784680 | 7.33e-05 | 0.307 | `CCGCCTGC` |
| CCWCCTGC | chr17 | − | 71989144 | 71989151 | 7.33e-05 | 0.307 | `CCGCCTGC` |
| CCWCCTGC | chr17 | + | 75843173 | 75843180 | 7.33e-05 | 0.307 | `CCCCCTGC` |
| CCWCCTGC | chr18 | + | 12410432 | 12410439 | 7.33e-05 | 0.307 | `CCGCCTGC` |
| CCWCCTGC | chr18 | + | 53448329 | 53448336 | 7.33e-05 | 0.307 | `CCCCCTGC` |
| CCWCCTGC | chr18 | + | 55220755 | 55220762 | 7.33e-05 | 0.307 | `CCGCCTGC` |
| CCWCCTGC | chr19 | − | 1389544 | 1389551 | 7.33e-05 | 0.307 | `CCGCCTGC` |
| CCWCCTGC | chr19 | − | 1602711 | 1602718 | 7.33e-05 | 0.307 | `CCGCCTGC` |
| CCWCCTGC | chr19 | + | 2429981 | 2429988 | 7.33e-05 | 0.307 | `CCCCCTGC` |
| CCWCCTGC | chr19 | − | 4218921 | 4218928 | 7.33e-05 | 0.307 | `CCGCCTGC` |
| CCWCCTGC | chr19 | + | 4742267 | 4742274 | 7.33e-05 | 0.307 | `CCCCCTGC` |
| CCWCCTGC | chr19 | − | 5741913 | 5741920 | 7.33e-05 | 0.307 | `CCGCCTGC` |
| CCWCCTGC | chr19 | + | 7676576 | 7676583 | 7.33e-05 | 0.307 | `CCCCCTGC` |
| CCWCCTGC | chr19 | − | 8292479 | 8292486 | 7.33e-05 | 0.307 | `CCCCCTGC` |
| CCWCCTGC | chr19 | + | 10565807 | 10565814 | 7.33e-05 | 0.307 | `CCGCCTGC` |
| CCWCCTGC | chr19 | + | 12755709 | 12755716 | 7.33e-05 | 0.307 | `CCCCCTGC` |
| CCWCCTGC | chr19 | − | 12764703 | 12764710 | 7.33e-05 | 0.307 | `CCGCCTGC` |
| CCWCCTGC | chr19 | − | 12765576 | 12765583 | 7.33e-05 | 0.307 | `CCGCCTGC` |
| CCWCCTGC | chr19 | + | 17366913 | 17366920 | 7.33e-05 | 0.307 | `CCGCCTGC` |
| CCWCCTGC | chr19 | − | 38360028 | 38360035 | 7.33e-05 | 0.307 | `CCGCCTGC` |
| CCWCCTGC | chr19 | − | 38463575 | 38463582 | 7.33e-05 | 0.307 | `CCCCCTGC` |
| CCWCCTGC | chr19 | − | 42260982 | 42260989 | 7.33e-05 | 0.307 | `CCGCCTGC` |
| CCWCCTGC | chr19 | + | 44592720 | 44592727 | 7.33e-05 | 0.307 | `CCCCCTGC` |
| CCWCCTGC | chr19 | − | 46508120 | 46508127 | 7.33e-05 | 0.307 | `CCGCCTGC` |
| CCWCCTGC | chr19 | − | 50965502 | 50965509 | 7.33e-05 | 0.307 | `CCGCCTGC` |
| CCWCCTGC | chr19 | − | 63722895 | 63722902 | 7.33e-05 | 0.307 | `CCCCCTGC` |
| CCWCCTGC | chr20 | − | 45821093 | 45821100 | 7.33e-05 | 0.307 | `CCCCCTGC` |
| CCWCCTGC | chr20 | − | 56659645 | 56659652 | 7.33e-05 | 0.307 | `CCCCCTGC` |
| CCWCCTGC | chr22 | − | 16119032 | 16119039 | 7.33e-05 | 0.307 | `CCCCCTGC` |
| CCWCCTGC | chr22 | − | 17800017 | 17800024 | 7.33e-05 | 0.307 | `CCCCCTGC` |
| CCWCCTGC | chr22 | + | 21604147 | 21604154 | 7.33e-05 | 0.307 | `CCCCCTGC` |
| CCWCCTGC | chr22 | − | 21604303 | 21604310 | 7.33e-05 | 0.307 | `CCCCCTGC` |
| CCWCCTGC | chr22 | − | 36334601 | 36334608 | 7.33e-05 | 0.307 | `CCGCCTGC` |
| CCWCCTGC | chr22 | + | 46771086 | 46771093 | 7.33e-05 | 0.307 | `CCCCCTGC` |
| CCWCCTGC | chr22 | + | 49311768 | 49311775 | 7.33e-05 | 0.307 | `CCCCCTGC` |
| CCWCCTGC | chr22 | + | 49316201 | 49316208 | 7.33e-05 | 0.307 | `CCCCCTGC` |

---

**DEBUGGING INFORMATION**


---

Command line:

```
/ebi/sw/MEME/VM-cluster410/meme-versions/4.10.0/bin/fimo --parse-genomic-coord --verbosity 1 --oc fimo_out_18 --bgfile ./background --motif CCWCCTGC dreme_out/dreme.xml ./Supplementary_Table_1.500bp.fa
```

Settings:

```
|  |  |  |
| --- | --- | --- |
| output directory = fimo_out_18 | MEME file name = dreme_out/dreme.xml | sequence file name = ./Supplementary_Table_1.500bp.fa |
| background file name = ./background | allow clobber = true | compute q-values = true |
| parse genomic coord. = true | text only = false | scan both strands = true |
| max sequence length = 250000000 | output threshold = 0.0001 | threshold type = p-value |
| max stored scores = 100000 | pseudocount = 0.1 | verbosity = 1 |
| selected motif = CCWCCTGC |  |  |
```

This information can be useful in the event you wish to report a
problem with the FIMO software.

---

**Go to top**
