# Supplementary material for: The MEME Suite
Source: Nucleic Acids Res. 2015 May 7;43(Web Server issue):W39–49. doi: 10.1093/nar/gkv416 (PMC4489269; doi:10.1093/nar/gkv416)
Supplement: SUPPLEMENTARY DATA [file supp_gkv416_nar-00283-web-b-2015-File005.zip › case4/meme-chip/fimo_out_19/fimo.html]

FIMO Results


---

|  |  |  |
| --- | --- | --- |
| **Database and Motifs** | **High-scoring Motif Occurrences** | **Debugging Information** |

  
  

---

**FIMO - Motif search tool**


---

FIMO version 4.10.0,
(Release date: Wed May 21 10:35:36 2014 +1000)

For further information on how to interpret these results
or to get a copy of the FIMO software please access
http://meme.nbcr.net

If you use FIMO in your research, please cite the following paper:  
Charles E. Grant, Timothy L. Bailey, and William Stafford Noble,
"FIMO: Scanning for occurrences of a given motif",
*Bioinformatics*, **27**(7):1017-1018, 2011.
[full text]

---

**DATABASE AND MOTIFS**


---

DATABASE
./Supplementary\_Table\_1.500bp.fa  
Database contains
2776
sequences,
1388000
residues

MOTIFS
db/uniprobe\_mouse.meme
(nucleotide)

| MOTIF | WIDTH | BEST POSSIBLE MATCH |
| --- | --- | --- |
| UP00078\_1 | 17 | GGGTTTAATTAAAATTC |
| UP00059\_1 | 14 | CTAATATTGCTAAA |
| UP00099\_1 | 17 | CTCAGCAGCTGCTCCTG |
| UP00020\_1 | 16 | ACGATGACGTCATCGA |
| UP00012\_1 | 15 | TAATTCAATGAAGTG |
| UP00043\_1 | 16 | TCTTTCGAGGAATTTG |
| UP00050\_1 | 22 | GGAAGAGTCACGTGACCAATAC |
| UP00001\_1 | 15 | ATAAAGGCGCGCGAT |
| UP00003\_1 | 15 | ATAAGGGCGCGCGAT |
| UP00007\_1 | 14 | TCCGCCCCCGCATT |
| UP00015\_1 | 15 | AGGACCCGGAAGTAA |
| UP00407\_1 | 13 | TACAAGGAAGTAA |
| UP00068\_1 | 17 | TAAAAGGTGTGAAAATT |
| UP00079\_1 | 17 | TATTCAAGGTCATGCGA |
| UP00073\_1 | 17 | AAAAAGTAAACAAAGAC |
| UP00041\_1 | 16 | AAAGTAAACAAAAATT |
| UP00039\_1 | 17 | AAAAAGTAAACAAACCC |
| UP00025\_1 | 17 | AAAATGTAAACAAACAG |
| UP00061\_1 | 17 | TAAATGTAAACAAAGGT |
| UP00408\_1 | 17 | CAATACCGGAAGTGTAA |
| UP00032\_1 | 22 | TTTTTAGAGATAAGAAATAAAG |
| UP00080\_1 | 17 | TAAACTGATAAGAAGAT |
| UP00100\_1 | 17 | TATAGAGATAAGAATTG |
| UP00070\_1 | 16 | TCGTACCCGCATCATT |
| UP00024\_1 | 16 | TATCGACCCCCCACAG |
| UP00042\_1 | 17 | CAGATGTGCACATACGT |
| UP00084\_1 | 17 | GAGTGTACGTACGATGG |
| UP00055\_1 | 16 | ACTATGAATGAATGAT |
| UP00035\_1 | 16 | ACTATGCCAACCTACC |
| UP00066\_1 | 17 | CTTCAGGGGTCAATTGA |
| UP00391\_1 | 14 | TGGAGGTAATTAAC |
| UP00072\_1 | 16 | ATTTACGACAAATAGC |
| UP00086\_1 | 14 | GAGAACCGAAACTG |
| UP00018\_1 | 15 | CGTATCGAAACCAAA |
| UP00040\_1 | 15 | ATAAACCGAAACCAA |
| UP00011\_1 | 17 | CTGATCGAAACCAAAGT |
| UP00074\_1 | 15 | CAAAATCGAAACTAA |
| UP00103\_1 | 16 | CCGATGACGTCATCGT |
| UP00093\_1 | 16 | TCGACCCCGCCCCTAT |
| UP00067\_1 | 17 | AATCCCTTTGATCTATC |
| UP00045\_1 | 17 | AAATTTGCTGACTTAGC |
| UP00044\_1 | 15 | TAAAAATGCTGACTT |
| UP00060\_1 | 16 | TGACCACGTGGTCGGG |
| UP00097\_1 | 16 | GGGCCGTGTGCAAAAA |
| UP00092\_1 | 17 | ATGGAAACCGTTATTTT |
| UP00081\_1 | 17 | TTGAAAACCGTTAATTT |
| UP00036\_1 | 16 | GAAGAACAGGTGTCCG |
| UP00017\_1 | 17 | CTTAACCACTTAAGGAT |
| UP00009\_1 | 16 | TCTCAAAGGTCACGAG |
| UP00027\_1 | 16 | TTTTACAGTAGCAAAA |
| UP00052\_1 | 16 | ATGTACAGTAGCAAAG |
| UP00088\_1 | 16 | TTGGGGGCGCCCCTAG |
| UP00048\_1 | 16 | TCTCAAAGGTCACCTG |
| UP00098\_1 | 23 | TGTGACCCTTAGCAACCGATTAA |
| UP00056\_1 | 15 | TACCATAGCAACGGT |
| UP00076\_1 | 15 | CCGCATAGCAACGGA |
| UP00053\_1 | 17 | TGTCGTGACCCCTTAAT |
| UP00085\_1 | 14 | TTAAGAGGAAGTTA |
| UP00008\_1 | 17 | AATAGGGTATCATATAT |
| UP00000\_1 | 17 | CAAATCCAGACATCAGA |
| UP00030\_1 | 17 | ATAAGAACAAAGGACTA |
| UP00101\_1 | 14 | TAATTGTTCTAAAC |
| UP00096\_1 | 16 | TTAAGAACAATAATTT |
| UP00004\_1 | 16 | GCTAATTATAATTATC |
| UP00075\_1 | 17 | TAGTGAACAATAGATTT |
| UP00014\_1 | 15 | ATAAACAATTAATCA |
| UP00064\_1 | 16 | TTCAATTGTTCTAAAA |
| UP00069\_1 | 16 | AATCAATTCAATAATT |
| UP00071\_1 | 16 | TTTAATTATAATTAAG |
| UP00023\_1 | 16 | ATTGAACAATGGAATT |
| UP00062\_1 | 17 | AGAAGAACAAAGGACTA |
| UP00091\_1 | 16 | TTTAGAACAATAAAAT |
| UP00034\_1 | 22 | AATAAAGAACAATAGAATTTCA |
| UP00051\_1 | 17 | TTATCTATTGTTCTTTA |
| UP00049\_1 | 14 | ATTTTACGGAAAAT |
| UP00002\_1 | 17 | GGTCCCGCCCCCTTCTC |
| UP00406\_1 | 16 | GTACATCCGGATTTTT |
| UP00077\_1 | 14 | TTCCATATATGGAA |
| UP00016\_1 | 16 | TATAATTATAATATTC |
| UP00029\_1 | 16 | TCTTTATATATAAATA |
| UP00089\_1 | 17 | ACTTAGTTAACTAAAAA |
| UP00058\_1 | 17 | TATAGATCAAAGGAAAA |
| UP00054\_1 | 17 | TATAGATCAAAGGAAAA |
| UP00083\_1 | 17 | ATTTCCTTTGATCTATA |
| UP00005\_1 | 15 | ATTCCCTGAGGGGAA |
| UP00010\_1 | 14 | TTGCCCTAGGGCAT |
| UP00087\_1 | 15 | ATTGCCTGAGGCGAA |
| UP00028\_1 | 15 | ATTGCCTGAGGCGAT |
| UP00046\_1 | 17 | ATCCACAGGTGCGAAAA |
| UP00019\_1 | 17 | CTAAGGTTCTAGATCAC |
| UP00031\_1 | 17 | AATCGCACTGCATTCCG |
| UP00047\_1 | 15 | AAGCCCCCCAAAAAT |
| UP00037\_1 | 15 | AACAAACAACAAGAG |
| UP00094\_1 | 17 | TCTTTGGCGTACCCTAA |
| UP00065\_1 | 16 | TGGCGCGCGCGCCTGA |
| UP00082\_1 | 14 | TTATGTACTAATAA |
| UP00021\_1 | 15 | TCCCCCCCCCCCCCC |
| UP00033\_1 | 17 | TATTATGGGATGGATAA |
| UP00095\_1 | 17 | CGAACAGTGCTCACTAT |
| UP00022\_1 | 16 | CCCCCCCCCCCACTTG |
| UP00102\_1 | 14 | CACCCCCGGGGGGG |
| UP00057\_1 | 15 | CCCCCCCGGGGGGGT |
| UP00006\_1 | 15 | CCCCCCCGGGGGGGT |
| UP00026\_1 | 17 | TACATGTGCACATAAAA |
| UP00078\_2 | 15 | ACCCGTATCAAATTT |
| UP00059\_2 | 17 | CGTACAATACGAAATAA |
| UP00099\_2 | 16 | CTATCCCCGCCCTATT |
| UP00020\_2 | 14 | GAATGACGAATAAC |
| UP00012\_2 | 17 | TGATTGTTAACAGTTGG |
| UP00043\_2 | 16 | ATCCCCGCCCCTAAAA |
| UP00050\_2 | 23 | TGTCGTTACACGTGGAAGGCGGT |
| UP00001\_2 | 17 | CGTTCGGCGCCAAAAGG |
| UP00003\_2 | 17 | CGCTCGGCGCCAAAAGC |
| UP00007\_2 | 16 | TGCGGAGTGGGACTGG |
| UP00015\_2 | 16 | TAGTATTTCCGATCTT |
| UP00407\_2 | 17 | GTTCAAAAAAAAAATTC |
| UP00068\_2 | 16 | GCGGAGGTGTCGCCTC |
| UP00079\_2 | 17 | GGCGAGGGGTCAAGGGC |
| UP00073\_2 | 15 | AAAAATAACAAACGG |
| UP00041\_2 | 15 | ATGTCACAACAACAC |
| UP00039\_2 | 17 | AACACCAAAACAAAGGA |
| UP00025\_2 | 15 | CAAACAACAACACCT |
| UP00061\_2 | 16 | ATATCAAAACAAAACA |
| UP00408\_2 | 16 | CCGTCTTCCCCCTCAC |
| UP00032\_2 | 22 | TTTTGTAGATTTTATCGACTTA |
| UP00080\_2 | 17 | GACAGAGATATCAGTTT |
| UP00100\_2 | 17 | GCGGCGATATCGCAGCG |
| UP00070\_2 | 17 | TGCGCATAGGGGAGGAG |
| UP00024\_2 | 14 | AATATTAATAAAGA |
| UP00042\_2 | 16 | AGCGGCACACACGCAA |
| UP00084\_2 | 16 | TGGGCGACGTCGTTAA |
| UP00055\_2 | 17 | TGTTCCCATTGTGTACT |
| UP00035\_2 | 16 | GGGTGTGCCCAAAAGG |
| UP00066\_2 | 16 | TGCAAAAGTCCAATAT |
| UP00391\_2 | 14 | AAAAACCATTAAGG |
| UP00072\_2 | 16 | ATGGAAAGTCGTAAAA |
| UP00086\_2 | 14 | GGAGAAAGGTGCGA |
| UP00018\_2 | 15 | AGTATTCTCGGTTGC |
| UP00040\_2 | 15 | TTGATCGAGAATTCC |
| UP00011\_2 | 15 | ACCACTCTCGGTCAC |
| UP00074\_2 | 14 | GCAAAACATTACTA |
| UP00103\_2 | 16 | ATTGATGAGTCACCAA |
| UP00093\_2 | 17 | AAGCATACGCCCAACTT |
| UP00067\_2 | 16 | GAAGATCAATCACTTA |
| UP00045\_2 | 15 | CAATTGCAAAAATAT |
| UP00044\_2 | 15 | GAAAAAATTGCAAGG |
| UP00060\_2 | 14 | GTGCCACGCGACTG |
| UP00097\_2 | 14 | AAATAAGAAAAAAC |
| UP00092\_2 | 16 | CGACCAACTGCCATGC |
| UP00081\_2 | 15 | CGACCAACTGCCGTG |
| UP00036\_2 | 15 | AGCAACAGCCGCACC |
| UP00017\_2 | 17 | ACTCCAAGTACTTGGAA |
| UP00009\_2 | 16 | CGCGCCGGGTCACGTA |
| UP00027\_2 | 16 | ACATGCTACCTAATAC |
| UP00052\_2 | 16 | ACTTGCTACCTACACC |
| UP00088\_2 | 17 | GCTGGGGGGTACCCCTT |
| UP00048\_2 | 16 | AGAGCGGGGTCAAGTA |
| UP00098\_2 | 23 | ACTGACGCTTGGTTACCACAAAG |
| UP00056\_2 | 15 | TACCCTAGTTACCGA |
| UP00076\_2 | 17 | CTACTTGGATACGGAAT |
| UP00053\_2 | 16 | TCGCGAAGGTTGTACT |
| UP00085\_2 | 14 | CAAATTCCGGAACC |
| UP00008\_2 | 17 | ATGGGATATATCCGCCT |
| UP00000\_2 | 17 | TACGCCCCGCCACTCTG |
| UP00030\_2 | 14 | AAAATTGTTATGAA |
| UP00101\_2 | 16 | AAATAGACAAAGGAAT |
| UP00096\_2 | 17 | GTATTGGGTGGGTATTT |
| UP00004\_2 | 15 | CTCACACAATGGCGC |
| UP00075\_2 | 15 | TTGAATGAAATTCGA |
| UP00014\_2 | 17 | GACCACATTCATACAAT |
| UP00064\_2 | 16 | GGACTGAATTCATGCC |
| UP00069\_2 | 15 | CTATAATTGTTATCG |
| UP00071\_2 | 17 | CATCAATTGTTCCGCTA |
| UP00023\_2 | 16 | TAAGATTATAATACGG |
| UP00062\_2 | 17 | GGAAAAATTGTTAGGAA |
| UP00091\_2 | 15 | TATCATAATTAAGGA |
| UP00034\_2 | 22 | GTGCTAATTGTGTGTGTACGCT |
| UP00051\_2 | 14 | ACATTCATGACACG |
| UP00049\_2 | 15 | TCCGTCGCTTAAAAG |
| UP00002\_2 | 15 | CAAAGGCGTGGCCAG |
| UP00406\_2 | 16 | GATAACATCCTAGTAG |
| UP00077\_2 | 17 | GTTAAAAAAAAAAATTT |
| UP00016\_2 | 17 | TCACGGAACAATAGGTG |
| UP00029\_2 | 15 | CCGATTTAAGCGATC |
| UP00089\_2 | 14 | TTGCCCGGATTAGG |
| UP00058\_2 | 15 | AGCCGAAAAAAAAAT |
| UP00054\_2 | 15 | CCGTATTATAAACAA |
| UP00083\_2 | 16 | GAAGATCAATCACTAA |
| UP00005\_2 | 14 | TCACCTCTGGGCAG |
| UP00010\_2 | 15 | ATTGCCTCAGGCAAT |
| UP00087\_2 | 14 | CCGCCCAAGGGCAG |
| UP00028\_2 | 14 | TACTGGAAAAAAAA |
| UP00046\_2 | 17 | AAGGCCAGATGGTCCGG |
| UP00019\_2 | 15 | TATCATTAGAACGCT |
| UP00031\_2 | 16 | CAATCACTGGCAGAAT |
| UP00047\_2 | 17 | CTTAAGACCACCATTAC |
| UP00037\_2 | 17 | GTGGTTCAATAATTTTG |
| UP00094\_2 | 14 | TGTATATATATACC |
| UP00065\_2 | 14 | GCCGCGCAGTGCGT |
| UP00082\_2 | 16 | GAGCCCTTGTCCCTTG |
| UP00021\_2 | 17 | AGGAGACCCCCAATTTG |
| UP00033\_2 | 17 | TCACCCCGCCCCTAATT |
| UP00095\_2 | 17 | TACGAGACTCCTCTAAC |
| UP00022\_2 | 17 | AAATTCCCCCCGGAAGT |
| UP00102\_2 | 15 | CCACACAGCAGGAGA |
| UP00057\_2 | 15 | CCACACAGCAGGAGA |
| UP00006\_2 | 15 | GAGCACAGCAGGACA |
| UP00026\_2 | 16 | CGAAGCACACAAAATA |
| UP00108\_1 | 17 | TAAACTAATTAGCTGAG |
| UP00187\_1 | 17 | CGCATTAATTAATTACC |
| UP00152\_1 | 17 | GTCCATTAATTAATGGA |
| UP00228\_1 | 17 | CATAACCACTTAACAAC |
| UP00166\_1 | 16 | AACAACCAATTAATTC |
| UP00145\_1 | 16 | AAAAACCAATTAAGAA |
| UP00181\_1 | 16 | AAAGTAATTAGTGAAT |
| UP00151\_1 | 16 | TAAGTAATTAGTTATA |
| UP00138\_1 | 16 | CAGGTAATTACCTCAG |
| UP00209\_1 | 17 | CGAATTAATTAATCACC |
| UP00209\_2 | 17 | CGCATTAATTAATTGGC |
| UP00240\_1 | 16 | TAAGGTAATAAAATTA |
| UP00133\_1 | 16 | AACGGTAATAAAATTT |
| UP00198\_1 | 14 | ATGATCGAATCAAA |
| UP00176\_1 | 16 | CGTTGGGGATTAGCCT |
| UP00219\_1 | 17 | ACCGGTTGATCACCTGA |
| UP00219\_2 | 15 | TAATGATGATCACTA |
| UP00255\_1 | 17 | TAATTAATTAATAATTA |
| UP00218\_1 | 16 | TTTAATTAATTAATTC |
| UP00202\_1 | 14 | CTGAGGTAATTAAT |
| UP00126\_1 | 16 | GGAATAATTACTTCAG |
| UP00154\_1 | 17 | TCGCGATAATTACCGAC |
| UP00110\_1 | 17 | TCGCTATAATTACCGAC |
| UP00230\_1 | 16 | GGGGTAATTAGCTCTG |
| UP00111\_1 | 17 | TGAACCGGATTAATGAA |
| UP00232\_1 | 17 | TAAATAGATACCCCATA |
| UP00143\_1 | 17 | GGAAGGGATTAATTATC |
| UP00227\_1 | 17 | CGACCCAATCAACGGTG |
| UP00201\_1 | 17 | ACCACTAATTAGTGGAC |
| UP00167\_1 | 16 | GCGAACTAATTAATGC |
| UP00163\_1 | 17 | TGCACTAATTAGTGGAA |
| UP00251\_1 | 17 | ATCCATTAATTAATTGA |
| UP00162\_1 | 17 | AGAACTAATTAGTGGAC |
| UP00132\_1 | 17 | CACCGCTAATTAGCGTT |
| UP00204\_1 | 17 | TGCCACTAATTAGTGTA |
| UP00131\_1 | 17 | AGCGCTAATTAGCGATT |
| UP00112\_1 | 17 | AATCGTTAATCCCTTTA |
| UP00127\_1 | 16 | AGGTTAATTAGCTGAT |
| UP00148\_1 | 17 | AAGGCGAAATCATCGCA |
| UP00225\_1 | 15 | CCATAATTAATTACA |
| UP00123\_1 | 16 | GTACTAATTAGTGGCG |
| UP00161\_1 | 17 | GAAAACTAGTTAACATC |
| UP00104\_1 | 17 | ACAAGCAATTAATGAAT |
| UP00155\_1 | 17 | ACAAGCAATTAAAGAAT |
| UP00157\_1 | 17 | ACAAGCAATTAAAGAAT |
| UP00114\_1 | 17 | AAAACATCGTTTTTAAG |
| UP00264\_1 | 16 | CTGAGCTAATTACCGT |
| UP00217\_1 | 16 | TAGGTAATAAAATTCA |
| UP00246\_1 | 16 | TAAAGTCGTAAAACAT |
| UP00183\_1 | 16 | AAAGCTCGTAAAATTT |
| UP00174\_1 | 16 | AAGGTAATTAGCTCAT |
| UP00391\_3 | 14 | TTGAGGTAATTAGT |
| UP00196\_1 | 17 | GATTATTAATTAACTTG |
| UP00189\_1 | 16 | ACGGTAATTAGCTCAG |
| UP00182\_1 | 16 | AAGGTAATTACCTAAT |
| UP00164\_1 | 17 | CGAGTTAATTAATAAGC |
| UP00164\_2 | 16 | GTAGTAATTAATGGAA |
| UP00213\_1 | 17 | ACGGCCATAAAATTAAT |
| UP00134\_1 | 16 | AACCCAATAAAATTCG |
| UP00137\_1 | 17 | TGAGCTAATTAGTTGGA |
| UP00144\_1 | 17 | CGCGTTAATTAATTACC |
| UP00214\_1 | 16 | ACGGTAATTAGCTCAT |
| UP00259\_1 | 16 | TATTGGTAATTACCTT |
| UP00206\_1 | 16 | GTAGTAATTAATGCAA |
| UP00263\_1 | 16 | ACCGGCAATTAATAAA |
| UP00207\_1 | 16 | GGAGCCATAAAATTCG |
| UP00245\_1 | 16 | TAAAGTCGTAAAACGT |
| UP00235\_1 | 16 | TAAAGTCGTAAAATAG |
| UP00135\_1 | 17 | TTAGGTCGTAAAATTTC |
| UP00173\_1 | 16 | AAAGCTCGTAAAATTT |
| UP00113\_1 | 17 | CGAATTAATTAACAATA |
| UP00252\_1 | 17 | CGAATTAATTAATTACT |
| UP00260\_1 | 17 | CAAATTAATTAATAAAA |
| UP00242\_1 | 16 | TTGGGGTAATTAACGT |
| UP00197\_1 | 16 | GGAGGTCATTAATTAT |
| UP00140\_1 | 17 | TAAACTAATTAGCTGTA |
| UP00121\_1 | 17 | AATGCAATAAAATTTAT |
| UP00117\_1 | 17 | TAAGGTCGTAAAATCCT |
| UP00177\_1 | 17 | CAAGGTCGTAAAATCTT |
| UP00180\_1 | 16 | CTACCAATAAAATTCT |
| UP00241\_1 | 16 | TTGAGTTAATTAACCT |
| UP00168\_1 | 17 | TAATTAATTAATGGCTA |
| UP00124\_1 | 16 | AAGGTAATTAGCTCAT |
| UP00236\_1 | 17 | TAAATACATGTAAAATT |
| UP00223\_1 | 17 | AAAATACATGTAATACT |
| UP00223\_2 | 17 | AATATACATGTAATATT |
| UP00194\_1 | 17 | AATATACATGTAAAACA |
| UP00250\_1 | 17 | TATATACATGTAAAATT |
| UP00150\_1 | 17 | AAAATACATGTAAAAAT |
| UP00170\_1 | 16 | CAAAATCAATTAATTT |
| UP00243\_1 | 16 | ACTCCTAATTAGTCGT |
| UP00120\_1 | 17 | TGCATTAATTAATGCGA |
| UP00262\_1 | 17 | CGAATTAATTAATAATG |
| UP00115\_1 | 17 | TAAACTAATTAGTGAAC |
| UP00130\_1 | 17 | GTAATTAATTAAATAAT |
| UP00261\_1 | 17 | TAAACTAATTAGCTTTG |
| UP00212\_1 | 17 | CGAATTAATTAAATACT |
| UP00256\_1 | 17 | GAGCGTTAATTAATGTA |
| UP00256\_2 | 17 | TCCACTAATTAGCGGTT |
| UP00184\_1 | 17 | ACCCCTAATTAGCGGTG |
| UP00175\_1 | 17 | CCCATTAATTAATCACC |
| UP00188\_1 | 17 | CGAATTAATTAAAAACC |
| UP00169\_1 | 17 | AGTTTTTAATTAATTTG |
| UP00186\_1 | 16 | AAGGAGCTGTCAATAC |
| UP00233\_1 | 16 | GAGGTAATTACCTCAG |
| UP00226\_1 | 16 | AAAGACCTGTCAATAC |
| UP00210\_1 | 16 | AATTACCTGTCAATAC |
| UP00234\_1 | 16 | TGCAACTAATTAATTC |
| UP00156\_1 | 17 | GAAGACCAATTAGCGCT |
| UP00171\_1 | 16 | CAAAACCAATTAATTT |
| UP00220\_1 | 17 | TGCGCTAATTAGTGGGA |
| UP00139\_1 | 17 | GTGCACTAATTAGTGCA |
| UP00231\_1 | 17 | TTAACCACTTGAAAATT |
| UP00190\_1 | 16 | CTTTAAGTACTTAATG |
| UP00107\_1 | 16 | TAAGCCACTTGAAATT |
| UP00249\_1 | 16 | TAAGCCACTTGAATTT |
| UP00147\_1 | 16 | TAAGCCACTTAACATT |
| UP00119\_1 | 17 | TTTTAAGTACTTAAATT |
| UP00017\_3 | 17 | TACTAAGTACTTAAATG |
| UP00200\_1 | 17 | GAAAATTAATTACTTCG |
| UP00200\_2 | 16 | AGTAATTAATTACTTC |
| UP00238\_1 | 17 | GATAATTAATTACTTTG |
| UP00216\_1 | 17 | TTAAGGGGATTAACTAC |
| UP00239\_1 | 17 | TGAGGGGGATTAACTAT |
| UP00160\_1 | 17 | TGAGGGGGATTAACTAT |
| UP00208\_1 | 17 | TAGAGGGATTAAATTTC |
| UP00208\_2 | 17 | GATAATTAATCCCTCTT |
| UP00109\_1 | 15 | AAAAACGGATTATTG |
| UP00178\_1 | 17 | CGCGCTAATTAGGTATC |
| UP00237\_1 | 17 | CGTAATTAATTAATTGG |
| UP00229\_1 | 17 | GGAGGGGATTAATTTAT |
| UP00267\_1 | 17 | TGTAGGGATTAATTGTC |
| UP00247\_1 | 17 | TGAACTAATTAGCCCAC |
| UP00224\_1 | 16 | TGATTAATTAATTGAC |
| UP00248\_1 | 17 | CGAACTAATTAGTACTA |
| UP00185\_1 | 17 | TCACCCATCAATAATCA |
| UP00221\_1 | 16 | CAGCATTAATTAGTAG |
| UP00149\_1 | 17 | CGGAATTAATTAATAGG |
| UP00153\_1 | 17 | TTAGAGGGATTAACAAT |
| UP00125\_1 | 17 | TGAAGGGATTAATCATC |
| UP00265\_1 | 16 | AGGGGGATTAGCTGCC |
| UP00203\_1 | 16 | AAAGACCTGTCAATCC |
| UP00205\_1 | 16 | AAGCACCTGTCAATAT |
| UP00158\_1 | 17 | GATTAATTAATTAAGTC |
| UP00254\_1 | 16 | ATGTATTAATTAAGTA |
| UP00191\_1 | 16 | TTGTATGCAAATTAGA |
| UP00179\_1 | 16 | TTGTATGCAAATTAGA |
| UP00129\_1 | 17 | AATTAATTAATTAATTC |
| UP00128\_1 | 17 | GATAATTAATTAGTTTG |
| UP00211\_1 | 17 | AAAATATGCATAATAAA |
| UP00105\_1 | 17 | AATTAATTAATTAATTC |
| UP00118\_1 | 16 | AGTTATTAATGAGGTC |
| UP00146\_1 | 17 | GACGATAATGAGGTTGC |
| UP00146\_2 | 17 | AAACATAATGAGGTTGC |
| UP00172\_1 | 17 | CGAATTAATTAAGAAAC |
| UP00266\_1 | 17 | GTAACTAATTAACTACT |
| UP00136\_1 | 17 | AAAGCTAATTAGCGAAA |
| UP00253\_1 | 17 | TGCACTAATTAGCGCAC |
| UP00193\_1 | 17 | AAGACGCTGTAAAGCGA |
| UP00193\_2 | 17 | AGGACGCTGTAAAGGGA |
| UP00116\_1 | 17 | TGCCTTAATTAATGCTC |
| UP00257\_1 | 17 | CGCGTTAATTAATTGTG |
| UP00192\_1 | 17 | GATGGGGTATCATTTTT |
| UP00159\_1 | 17 | AATGGGGTATCACTTTT |
| UP00195\_1 | 17 | GATAGGGTATCACTTAT |
| UP00199\_1 | 17 | ATAAATGACACCTATCA |
| UP00008\_3 | 17 | AATAGGGTATCAATTAT |
| UP00008\_4 | 17 | AATAGGGTATCAATATT |
| UP00089\_3 | 17 | CCTTAGTTAACTAAAAT |
| UP00222\_1 | 17 | AGCTGTTAACTAGCCGT |
| UP00122\_1 | 17 | GATATTGACAGCTGCGT |
| UP00258\_1 | 16 | AACTAGCTGTCAATAC |
| UP00165\_1 | 16 | TAAGCCACTTGAAATT |
| UP00244\_1 | 17 | TAATTAATTAATAACTT |
| UP00142\_1 | 17 | CATAATTAATTAACGCG |
| UP00215\_1 | 16 | ACGTTAATTAACCCAG |
| UP00106\_1 | 16 | GTGCACTAATTAAGAC |
| UP00141\_1 | 17 | CGAGTTAATTAATAATT |

Random model letter frequencies
(from ./background):
  
A 0.241 C 0.259 G 0.259 T 0.241

---

**SECTION I: HIGH-SCORING MOTIF OCCURRENCES**


---

- There were
  263
  motif occurrences with a
  p-value less than
  0.0001.
- The p-value of a motif occurrence is defined as the
  probability of a random sequence of the same length as the motif
  matching that position of the sequence with as good or better a score.
- The score for the match of a position in a sequence to a motif
  is computed by summing the appropriate entries from each column of
  the position-dependent scoring matrix that represents the motif.
- The q-value of a motif occurrence is defined as the
  false discovery rate if the occurrence is accepted as significant.
- The table is sorted by increasing p-value.

| Motif | Sequence Name | Strand | Start | End | p-value | q-value | Matched Sequence |
| --- | --- | --- | --- | --- | --- | --- | --- |
| UP00009\_2 | chr17 | − | 70827921 | 70827936 | 4.55e-07 | 0.807 | `CTGGCCGGGTCACCCT` |
| UP00009\_2 | chr3 | − | 4994203 | 4994218 | 9.75e-07 | 0.807 | `CGCTTCGGGTCAGTCA` |
| UP00009\_2 | chr1 | − | 154452928 | 154452943 | 2.68e-06 | 0.807 | `CTAGCCGGGTCACCGG` |
| UP00009\_2 | chr6 | − | 33237335 | 33237350 | 3.09e-06 | 0.807 | `CGCACCGGGTCAGCTG` |
| UP00009\_2 | chr14 | − | 105119403 | 105119418 | 5.52e-06 | 0.807 | `AGCCCGGGGTCAGCTA` |
| UP00009\_2 | chr17 | + | 8139786 | 8139801 | 6.24e-06 | 0.807 | `CGGGTGGGGTCACATA` |
| UP00009\_2 | chr4 | − | 184602472 | 184602487 | 7.18e-06 | 0.807 | `CCCGCCGGGTCAGCTG` |
| UP00009\_2 | chr7 | − | 148952911 | 148952926 | 7.18e-06 | 0.807 | `GTCGCGGGGTCAGCTC` |
| UP00009\_2 | chr8 | − | 67507184 | 67507199 | 7.18e-06 | 0.807 | `TTCGCGGGGTCACCTG` |
| UP00009\_2 | chr3 | + | 49370799 | 49370814 | 7.56e-06 | 0.807 | `GGCGGCGGGTCACGTG` |
| UP00009\_2 | chr3 | − | 120781266 | 120781281 | 8.13e-06 | 0.807 | `GGAGCCGGGTCACCGG` |
| UP00009\_2 | chr14 | + | 34943760 | 34943775 | 8.21e-06 | 0.807 | `GGGGCGGGGTCAGGCT` |
| UP00009\_2 | chr17 | − | 30929635 | 30929650 | 8.21e-06 | 0.807 | `CGCCTGGGGTCAGCGA` |
| UP00009\_2 | chr22 | − | 41341295 | 41341310 | 8.74e-06 | 0.807 | `GTAGGCGGGTCACCTT` |
| UP00009\_2 | chr8 | + | 135682201 | 135682216 | 9.18e-06 | 0.807 | `TTGTCCGGGTCACCCT` |
| UP00009\_2 | chr11 | − | 67984110 | 67984125 | 9.28e-06 | 0.807 | `GTAGCGGGGTCAAGGT` |
| UP00009\_2 | chr13 | + | 30089627 | 30089642 | 9.56e-06 | 0.807 | `CTCCGCGGGTCACCCC` |
| UP00009\_2 | chr16 | + | 680489 | 680504 | 1.02e-05 | 0.807 | `AGTTCCGGGTCAGGGA` |
| UP00009\_2 | chr7 | + | 4689345 | 4689360 | 1.09e-05 | 0.807 | `CTCCTGGGGTCACCCC` |
| UP00009\_2 | chr6 | − | 42859502 | 42859517 | 1.12e-05 | 0.807 | `GGTGCGGGGTCAGGGA` |
| UP00009\_2 | chr1 | − | 148398467 | 148398482 | 1.26e-05 | 0.807 | `GTAGTGGGGTCAGGCA` |
| UP00009\_2 | chr2 | − | 235074100 | 235074115 | 1.26e-05 | 0.807 | `AGCAGGGGGTCACTTA` |
| UP00009\_2 | chr19 | + | 63679183 | 63679198 | 1.27e-05 | 0.807 | `GTCAGCGGGTCACGGT` |
| UP00009\_2 | chr20 | + | 31453013 | 31453028 | 1.27e-05 | 0.807 | `CAGGCCGGGTCATGCT` |
| UP00009\_2 | chr17 | + | 59577467 | 59577482 | 1.42e-05 | 0.807 | `CGCCGGGGGTCAATCT` |
| UP00009\_2 | chr4 | + | 55127461 | 55127476 | 1.44e-05 | 0.807 | `CTCCCTGGGTCACCTA` |
| UP00009\_2 | chr1 | − | 110682577 | 110682592 | 1.47e-05 | 0.807 | `AGCCTCGGGTCATGCT` |
| UP00009\_2 | chr12 | − | 122134566 | 122134581 | 1.52e-05 | 0.807 | `TTCCCGGGGTCACCCC` |
| UP00009\_2 | chr13 | + | 97939245 | 97939260 | 1.52e-05 | 0.807 | `CACACGGGGTCACAGA` |
| UP00009\_2 | chr6 | − | 30565753 | 30565768 | 1.53e-05 | 0.807 | `TGGCCGGGGTCACTCA` |
| UP00009\_2 | chr11 | − | 440395 | 440410 | 1.59e-05 | 0.807 | `CACACCGGGTCACCGG` |
| UP00009\_2 | chr22 | + | 49310965 | 49310980 | 1.64e-05 | 0.807 | `CGCCGGGGGTCACGTG` |
| UP00009\_2 | chr19 | − | 12266854 | 12266869 | 1.67e-05 | 0.807 | `CGGGTCGGGTCACAAC` |
| UP00009\_2 | chr1 | − | 84744992 | 84745007 | 1.68e-05 | 0.807 | `CGCCTCGGGTCAGCGG` |
| UP00009\_2 | chr5 | + | 134012117 | 134012132 | 1.74e-05 | 0.807 | `CGAGCGAGGTCACGTT` |
| UP00009\_2 | chr1 | + | 181259719 | 181259734 | 1.77e-05 | 0.807 | `TGACCGGGGTCACCAA` |
| UP00009\_2 | chr20 | + | 45847254 | 45847269 | 1.9e-05 | 0.807 | `CCAGCGGGGTCAAACA` |
| UP00009\_2 | chr17 | − | 35164372 | 35164387 | 1.98e-05 | 0.807 | `CTGCCCGGGTCACAGC` |
| UP00009\_2 | chr9 | + | 125939861 | 125939876 | 2.01e-05 | 0.807 | `GTCTTGGGGTCAACTT` |
| UP00009\_2 | chr19 | − | 5631839 | 5631854 | 2.01e-05 | 0.807 | `GTTTTCGGGTCACCTT` |
| UP00009\_2 | chr1 | − | 179370533 | 179370548 | 2.07e-05 | 0.807 | `AGATGCGGGTCACATA` |
| UP00009\_2 | chr12 | + | 120810927 | 120810942 | 2.12e-05 | 0.807 | `CCTGCCGGGTCACCAC` |
| UP00009\_2 | chr17 | − | 70803380 | 70803395 | 2.14e-05 | 0.807 | `CAATCCGGGTCAGCCC` |
| UP00009\_2 | chr8 | − | 98725246 | 98725261 | 2.17e-05 | 0.807 | `GGAACCGGGTCACCGG` |
| UP00009\_2 | chr11 | − | 48087992 | 48088007 | 2.17e-05 | 0.807 | `ACCTCGGGGTCAATTA` |
| UP00009\_2 | chr21 | + | 35160034 | 35160049 | 2.19e-05 | 0.807 | `AGCTCGGGGTCATTTC` |
| UP00009\_2 | chr12 | − | 122025134 | 122025149 | 2.24e-05 | 0.807 | `AGGGCGGGGTCAGGTG` |
| UP00009\_2 | chr17 | − | 22683791 | 22683806 | 2.29e-05 | 0.807 | `AGGCCCGGGTCACCTG` |
| UP00009\_2 | chr19 | − | 47141358 | 47141373 | 2.29e-05 | 0.807 | `GTCTGGGGGTCACTGA` |
| UP00009\_2 | chr11 | − | 65076715 | 65076730 | 2.38e-05 | 0.807 | `CACATGGGGTCAGGGA` |
| UP00009\_2 | chr11 | + | 72765941 | 72765956 | 2.38e-05 | 0.807 | `CGTGTGGGGTCAAGTG` |
| UP00009\_2 | chr9 | − | 127043808 | 127043823 | 2.42e-05 | 0.807 | `CCCGGGGGGTCACTCC` |
| UP00009\_2 | chr1 | + | 32488421 | 32488436 | 2.49e-05 | 0.807 | `CTGGTGGGGTCAAGGC` |
| UP00009\_2 | chr1 | + | 201544195 | 201544210 | 2.49e-05 | 0.807 | `CTGGTCGGGTCATAGA` |
| UP00009\_2 | chr22 | + | 37207730 | 37207745 | 2.51e-05 | 0.807 | `CTAGTGGGGTCAAAAT` |
| UP00009\_2 | chr9 | + | 35832904 | 35832919 | 2.53e-05 | 0.807 | `ATGGGCGGGTCAACCA` |
| UP00009\_2 | chr11 | + | 605839 | 605854 | 2.69e-05 | 0.807 | `CCTCCGGGGTCACGGA` |
| UP00009\_2 | chr16 | − | 66464214 | 66464229 | 2.71e-05 | 0.807 | `GGGACGGGGTCAACTT` |
| UP00009\_2 | chr15 | + | 29408668 | 29408683 | 2.73e-05 | 0.807 | `CATTCGGGGTCAGTTA` |
| UP00009\_2 | chr17 | + | 1920854 | 1920869 | 2.79e-05 | 0.807 | `GAGGCGGGGTCACTGA` |
| UP00009\_2 | chr17 | + | 59274278 | 59274293 | 2.79e-05 | 0.807 | `GGCGCGGGGTCAGAGG` |
| UP00009\_2 | chr1 | − | 148125037 | 148125052 | 2.91e-05 | 0.807 | `GGGGTGGGGTCAAGAA` |
| UP00009\_2 | chr10 | + | 135052701 | 135052716 | 2.93e-05 | 0.807 | `TTAGTGGGGTCAGTAA` |
| UP00009\_2 | chr1 | + | 201557099 | 201557114 | 2.95e-05 | 0.807 | `AGGGTGGGGTCAGGTC` |
| UP00009\_2 | chr3 | + | 49034500 | 49034515 | 2.95e-05 | 0.807 | `CACGCTGGGTCAGGCT` |
| UP00009\_2 | chr1 | + | 29957628 | 29957643 | 3.03e-05 | 0.807 | `CAAGTGGGGTCAATGT` |
| UP00009\_2 | chr11 | + | 601236 | 601251 | 3.14e-05 | 0.807 | `GGCCTGGGGTCAAGCC` |
| UP00009\_2 | chr7 | + | 149733413 | 149733428 | 3.16e-05 | 0.807 | `GGGGGCGGGTCACTGC` |
| UP00009\_2 | chr6 | − | 106669183 | 106669198 | 3.2e-05 | 0.807 | `CTGCTGGGGTCAGCCT` |
| UP00009\_2 | chr2 | − | 152740464 | 152740479 | 3.35e-05 | 0.807 | `GGCCGCGGGTCACCCG` |
| UP00009\_2 | chr3 | − | 109327260 | 109327275 | 3.35e-05 | 0.807 | `CTCCCTGGGTCACTCC` |
| UP00009\_2 | chr10 | − | 112145481 | 112145496 | 3.35e-05 | 0.807 | `GCAGCCGGGTCACTGG` |
| UP00009\_2 | chr1 | − | 39054365 | 39054380 | 3.37e-05 | 0.807 | `AGCTCTGGGTCAGTTA` |
| UP00009\_2 | chr12 | − | 122134647 | 122134662 | 3.53e-05 | 0.807 | `CTTCTGGGGTCACCAT` |
| UP00009\_2 | chr4 | − | 39876456 | 39876471 | 3.55e-05 | 0.807 | `TGAATGGGGTCACTTC` |
| UP00009\_2 | chr6 | − | 143310452 | 143310467 | 3.55e-05 | 0.807 | `CGCGCCAGGTCAAATC` |
| UP00009\_2 | chr5 | + | 43639515 | 43639530 | 3.59e-05 | 0.807 | `CGTACGGGGTCATTTC` |
| UP00009\_2 | chr15 | + | 86983768 | 86983783 | 3.61e-05 | 0.807 | `TCAGCGGGGTCACCCC` |
| UP00009\_2 | chr22 | + | 49314980 | 49314995 | 3.66e-05 | 0.807 | `TTCCCGGGGTCATCAA` |
| UP00009\_2 | chr14 | + | 61287829 | 61287844 | 3.7e-05 | 0.807 | `CGTGGGGGGTCAGCCC` |
| UP00009\_2 | chr1 | + | 224917436 | 224917451 | 3.73e-05 | 0.807 | `TCCCCCGGGTCATCTT` |
| UP00009\_2 | chr6 | + | 3313129 | 3313144 | 3.8e-05 | 0.807 | `CTCACTGGGTCAAGTC` |
| UP00009\_2 | chr8 | + | 29253704 | 29253719 | 3.8e-05 | 0.807 | `TTAATGGGGTCACTAA` |
| UP00009\_2 | chr11 | + | 47557308 | 47557323 | 3.8e-05 | 0.807 | `AGACCGGGGTCAGGCG` |
| UP00009\_2 | chr16 | + | 27150846 | 27150861 | 3.82e-05 | 0.807 | `GAATTCGGGTCAGGTT` |
| UP00009\_2 | chr19 | − | 11896620 | 11896635 | 3.87e-05 | 0.807 | `TGCACGGGGTCACAGG` |
| UP00009\_2 | chr19 | − | 10258521 | 10258536 | 3.99e-05 | 0.807 | `CTCCCCAGGTCAAGAA` |
| UP00009\_2 | chr7 | − | 4689676 | 4689691 | 4.04e-05 | 0.807 | `GGACTGGGGTCAAGGT` |
| UP00009\_2 | chr14 | − | 102855454 | 102855469 | 4.11e-05 | 0.807 | `TTTTTGGGGTCAGGTA` |
| UP00009\_2 | chr20 | − | 45725817 | 45725832 | 4.16e-05 | 0.807 | `GACATGGGGTCACTGA` |
| UP00009\_2 | chr11 | − | 85835756 | 85835771 | 4.19e-05 | 0.807 | `GCTACCGGGTCACCGA` |
| UP00009\_2 | chr1 | − | 28776569 | 28776584 | 4.31e-05 | 0.807 | `AGGATGGGGTCAGGGA` |
| UP00009\_2 | chr1 | + | 117998985 | 117999000 | 4.31e-05 | 0.807 | `TGAGCTGGGTCAGTTT` |
| UP00009\_2 | chr22 | + | 39141579 | 39141594 | 4.31e-05 | 0.807 | `ATCTCGGGGTCATCTG` |
| UP00009\_2 | chr3 | − | 72308570 | 72308585 | 4.36e-05 | 0.807 | `CTTACTGGGTCACCTA` |
| UP00009\_2 | chr5 | − | 86448794 | 86448809 | 4.36e-05 | 0.807 | `TAAGTGGGGTCACTGT` |
| UP00009\_2 | chr7 | − | 106597569 | 106597584 | 4.41e-05 | 0.807 | `CTCTTTGGGTCACACA` |
| UP00009\_2 | chr7 | − | 2901141 | 2901156 | 4.55e-05 | 0.807 | `CCCCTGGGGTCAACAA` |
| UP00009\_2 | chr14 | + | 34940640 | 34940655 | 4.55e-05 | 0.807 | `CCACTGGGGTCAGTCA` |
| UP00009\_2 | chr1 | − | 148398540 | 148398555 | 4.6e-05 | 0.807 | `GCTGCGGGGTCACTTC` |
| UP00009\_2 | chr4 | − | 84596056 | 84596071 | 4.6e-05 | 0.807 | `CGCGGCAGGTCACGTG` |
| UP00009\_2 | chr8 | − | 67507422 | 67507437 | 4.62e-05 | 0.807 | `CTGAGCGGGTCACACC` |
| UP00009\_2 | chr19 | + | 12910358 | 12910373 | 4.62e-05 | 0.807 | `AGGGCTGGGTCAGGTT` |
| UP00009\_2 | chr2 | + | 231233668 | 231233683 | 4.7e-05 | 0.807 | `TGGCCGGGGTCAAACA` |
| UP00009\_2 | chr10 | − | 13805636 | 13805651 | 4.84e-05 | 0.807 | `TTTTCGGGGTCATCTA` |
| UP00009\_2 | chr15 | − | 38195325 | 38195340 | 4.84e-05 | 0.807 | `CAGCCGGGGTCAGTGT` |
| UP00009\_2 | chr1 | + | 205170691 | 205170706 | 4.87e-05 | 0.807 | `AGGCTGGGGTCAACCA` |
| UP00009\_2 | chr2 | + | 233632669 | 233632684 | 4.87e-05 | 0.807 | `GTAATGGGGTCACAGA` |
| UP00009\_2 | chr15 | + | 81298656 | 81298671 | 4.87e-05 | 0.807 | `GTATCCAGGTCACGCA` |
| UP00009\_2 | chr9 | − | 131687171 | 131687186 | 4.89e-05 | 0.807 | `GGTCCCGGGTCAAATC` |
| UP00009\_2 | chr16 | + | 87245491 | 87245506 | 4.92e-05 | 0.807 | `GTCACCAGGTCACTGA` |
| UP00009\_2 | chr19 | + | 12764601 | 12764616 | 4.92e-05 | 0.807 | `TGCTTGGGGTCAAGGG` |
| UP00009\_2 | chr6 | − | 7844354 | 7844369 | 4.95e-05 | 0.807 | `CACCTGGGGTCAGGGC` |
| UP00009\_2 | chr15 | + | 56533999 | 56534014 | 5e-05 | 0.807 | `CAAATGGGGTCAGGAA` |
| UP00009\_2 | chr6 | + | 36828883 | 36828898 | 5.06e-05 | 0.807 | `AGCCTGGGGTCAGGGG` |
| UP00009\_2 | chr8 | + | 67755365 | 67755380 | 5.06e-05 | 0.807 | `TGCATGGGGTCACTAG` |
| UP00009\_2 | chr2 | + | 181885829 | 181885844 | 5.08e-05 | 0.807 | `TGCTCTGGGTCACCAT` |
| UP00009\_2 | chr13 | − | 45319999 | 45320014 | 5.14e-05 | 0.807 | `GGTCTGGGGTCAGTCA` |
| UP00009\_2 | chr17 | − | 73648066 | 73648081 | 5.14e-05 | 0.807 | `TTAGCCAGGTCACTGT` |
| UP00009\_2 | chr22 | + | 27518523 | 27518538 | 5.22e-05 | 0.807 | `ATCATGGGGTCAGCTG` |
| UP00009\_2 | chr22 | + | 40558452 | 40558467 | 5.25e-05 | 0.807 | `AGCGCGAGGTCACGGC` |
| UP00009\_2 | chr2 | − | 70170782 | 70170797 | 5.31e-05 | 0.807 | `CTCATCAGGTCAGGGA` |
| UP00009\_2 | chr15 | + | 54325655 | 54325670 | 5.31e-05 | 0.807 | `AGAGCCAGGTCACCCC` |
| UP00009\_2 | chr6 | + | 33046135 | 33046150 | 5.39e-05 | 0.807 | `CTCGGGGGGTCAGAAG` |
| UP00009\_2 | chr12 | + | 46493492 | 46493507 | 5.39e-05 | 0.807 | `GGGCTGGGGTCACCTC` |
| UP00009\_2 | chr12 | + | 67488878 | 67488893 | 5.45e-05 | 0.807 | `CCTGTCGGGTCACTAG` |
| UP00009\_2 | chr11 | − | 1830964 | 1830979 | 5.47e-05 | 0.807 | `GCACCCGGGTCACTCG` |
| UP00009\_2 | chr14 | − | 99601972 | 99601987 | 5.47e-05 | 0.807 | `AGTCTCGGGTCATTCT` |
| UP00009\_2 | chr4 | − | 39878171 | 39878186 | 5.5e-05 | 0.807 | `CCCCTGGGGTCATCCT` |
| UP00009\_2 | chr13 | − | 109922359 | 109922374 | 5.56e-05 | 0.807 | `CTCATCAGGTCACTTC` |
| UP00009\_2 | chr3 | − | 158017570 | 158017585 | 5.59e-05 | 0.807 | `CCCGGCAGGTCACGCA` |
| UP00009\_2 | chr1 | + | 117998703 | 117998718 | 5.62e-05 | 0.807 | `GTCTCTGGGTCAATCT` |
| UP00009\_2 | chr4 | − | 39876320 | 39876335 | 5.62e-05 | 0.807 | `ATCTCCAGGTCAGTCT` |
| UP00009\_2 | chr14 | + | 23700738 | 23700753 | 5.65e-05 | 0.807 | `GGGTGGGGGTCAGGCT` |
| UP00009\_2 | chr1 | + | 1701672 | 1701687 | 5.68e-05 | 0.807 | `GGCCCCAGGTCACACA` |
| UP00009\_2 | chr17 | + | 38087480 | 38087495 | 5.68e-05 | 0.807 | `TCTACGGGGTCACTTT` |
| UP00009\_2 | chr15 | + | 72483676 | 72483691 | 5.7e-05 | 0.807 | `GGGCTGGGGTCAGGAA` |
| UP00009\_2 | chr18 | − | 44733450 | 44733465 | 5.73e-05 | 0.807 | `GACCTGGGGTCAAGGA` |
| UP00009\_2 | chr17 | − | 39936476 | 39936491 | 5.76e-05 | 0.807 | `CGCTCCGGGTCGCGCG` |
| UP00009\_2 | chr10 | + | 11319965 | 11319980 | 5.79e-05 | 0.807 | `ATGGCCAGGTCAGGCA` |
| UP00009\_2 | chr16 | − | 51685879 | 51685894 | 5.79e-05 | 0.807 | `CACCTGGGGTCACCGG` |
| UP00009\_2 | chr6 | − | 26342299 | 26342314 | 5.82e-05 | 0.807 | `GGTTGGGGGTCAGTTA` |
| UP00009\_2 | chr14 | + | 64998833 | 64998848 | 5.82e-05 | 0.807 | `TTCTTCAGGTCACTTA` |
| UP00009\_2 | chr20 | + | 45847208 | 45847223 | 5.82e-05 | 0.807 | `GTCCGCGGGTCAGGGG` |
| UP00009\_2 | chr1 | + | 40929771 | 40929786 | 5.85e-05 | 0.807 | `GGGGCGGGGTCAAAAC` |
| UP00009\_2 | chr8 | + | 103945744 | 103945759 | 5.88e-05 | 0.807 | `GCGACCGGGTCATGCA` |
| UP00009\_2 | chr20 | + | 48560092 | 48560107 | 5.97e-05 | 0.807 | `CTCCCGGGGTCGCCTA` |
| UP00009\_2 | chr14 | − | 89932903 | 89932918 | 6e-05 | 0.807 | `GGTCTCGGGTCAGGTG` |
| UP00009\_2 | chr1 | − | 40278357 | 40278372 | 6.03e-05 | 0.807 | `CGCGGTGGGTCAGCCC` |
| UP00009\_2 | chr22 | − | 40558572 | 40558587 | 6.09e-05 | 0.807 | `GAGTCCGGGTCATCCA` |
| UP00009\_2 | chr19 | − | 16310636 | 16310651 | 6.12e-05 | 0.807 | `AACCTGGGGTCAAGGT` |
| UP00009\_2 | chr11 | − | 72765652 | 72765667 | 6.21e-05 | 0.807 | `CGCGCCGTGTCACCCA` |
| UP00009\_2 | chr1 | − | 180662950 | 180662965 | 6.24e-05 | 0.807 | `TTATTGGGGTCATTCT` |
| UP00009\_2 | chr11 | + | 72765796 | 72765811 | 6.27e-05 | 0.807 | `GACCCGGGGTCACTGG` |
| UP00009\_2 | chr18 | − | 19994095 | 19994110 | 6.27e-05 | 0.807 | `ATCTCCAGGTCAAGAA` |
| UP00009\_2 | chr16 | − | 9128929 | 9128944 | 6.34e-05 | 0.807 | `ATCTTTGGGTCACTGA` |
| UP00009\_2 | chr12 | − | 81276497 | 81276512 | 6.4e-05 | 0.807 | `GGTCCGGGGTCACCGG` |
| UP00009\_2 | chr1 | − | 84744936 | 84744951 | 6.43e-05 | 0.807 | `AAACTCGGGTCACAGA` |
| UP00009\_2 | chr3 | + | 13100744 | 13100759 | 6.46e-05 | 0.807 | `CACGGCAGGTCACCCA` |
| UP00009\_2 | chr15 | + | 29296589 | 29296604 | 6.46e-05 | 0.807 | `GAAACGGGGTCAGGAT` |
| UP00009\_2 | chr1 | − | 201556701 | 201556716 | 6.49e-05 | 0.807 | `GGAGGGGGGTCAGGGG` |
| UP00009\_2 | chr7 | + | 101421053 | 101421068 | 6.49e-05 | 0.807 | `CTTGTCAGGTCAGCTA` |
| UP00009\_2 | chr1 | − | 26010198 | 26010213 | 6.52e-05 | 0.807 | `CAAGGCGGGTCACAGG` |
| UP00009\_2 | chr10 | + | 45276632 | 45276647 | 6.56e-05 | 0.807 | `TTCTGGGGGTCACTGG` |
| UP00009\_2 | chr20 | + | 36904696 | 36904711 | 6.56e-05 | 0.807 | `TGACTGGGGTCAGGTG` |
| UP00009\_2 | chr20 | + | 5041956 | 5041971 | 6.65e-05 | 0.807 | `CTACCTGGGTCACAGT` |
| UP00009\_2 | chr8 | + | 17824629 | 17824644 | 6.75e-05 | 0.807 | `TCCGGCGGGTCACATG` |
| UP00009\_2 | chr16 | + | 2141327 | 2141342 | 6.78e-05 | 0.807 | `CTGGTGGGGTCATGAG` |
| UP00009\_2 | chr20 | − | 47735362 | 47735377 | 6.85e-05 | 0.807 | `CTGTCTGGGTCACAGA` |
| UP00009\_2 | chr1 | + | 33055479 | 33055494 | 6.88e-05 | 0.807 | `CTTCCTGGGTCACCGT` |
| UP00009\_2 | chr17 | + | 35992208 | 35992223 | 6.88e-05 | 0.807 | `AGCCTCAGGTCACCCT` |
| UP00009\_2 | chr12 | − | 112095586 | 112095601 | 6.91e-05 | 0.807 | `CTGCTGGGGTCACCAG` |
| UP00009\_2 | chr12 | + | 112095762 | 112095777 | 6.91e-05 | 0.807 | `AGGCGGGGGTCACACA` |
| UP00009\_2 | chr6 | + | 7834159 | 7834174 | 6.95e-05 | 0.807 | `TTGCCCGGGTCAGAGC` |
| UP00009\_2 | chr15 | − | 86983915 | 86983930 | 6.95e-05 | 0.807 | `CAAGTGGGGTCACAGG` |
| UP00009\_2 | chr11 | − | 65076749 | 65076764 | 6.98e-05 | 0.807 | `GGTCCGGGGTCACATG` |
| UP00009\_2 | chr3 | − | 58002816 | 58002831 | 7.11e-05 | 0.807 | `AGAATGGGGTCAACTG` |
| UP00009\_2 | chr4 | − | 185973729 | 185973744 | 7.11e-05 | 0.807 | `TTATCCAGGTCACTGA` |
| UP00009\_2 | chr7 | − | 4689773 | 4689788 | 7.11e-05 | 0.807 | `AGAACCAGGTCAGGCT` |
| UP00009\_2 | chr5 | + | 60661357 | 60661372 | 7.15e-05 | 0.807 | `CCGGCTGGGTCAGGCT` |
| UP00009\_2 | chr10 | + | 80514224 | 80514239 | 7.15e-05 | 0.807 | `AGGTTGGGGTCAAAGA` |
| UP00009\_2 | chr5 | − | 1369298 | 1369313 | 7.18e-05 | 0.807 | `CGCGGTGGGTCAGCGC` |
| UP00009\_2 | chr14 | − | 94850882 | 94850897 | 7.18e-05 | 0.807 | `TGGCTGGGGTCAGGCC` |
| UP00009\_2 | chr10 | − | 125969176 | 125969191 | 7.21e-05 | 0.807 | `CGGTTGGGGTCATCTG` |
| UP00009\_2 | chr4 | + | 77339929 | 77339944 | 7.28e-05 | 0.807 | `TGCGTGAGGTCACTGT` |
| UP00009\_2 | chr3 | + | 4994178 | 4994193 | 7.39e-05 | 0.807 | `ACAGCCAGGTCACTCA` |
| UP00009\_2 | chr19 | − | 6724405 | 6724420 | 7.39e-05 | 0.807 | `CTCTGGGGGTCAAAGG` |
| UP00009\_2 | chr16 | + | 79267969 | 79267984 | 7.45e-05 | 0.807 | `GAACTGGGGTCAAGTT` |
| UP00009\_2 | chr22 | + | 27526727 | 27526742 | 7.49e-05 | 0.807 | `TACTTGGGGTCATTTT` |
| UP00009\_2 | chr8 | − | 102218651 | 102218666 | 7.59e-05 | 0.807 | `CTGCTGGGGTCATACT` |
| UP00009\_2 | chr17 | + | 24320373 | 24320388 | 7.59e-05 | 0.807 | `TGGGCCAGGTCAAGGT` |
| UP00009\_2 | chr22 | + | 48051965 | 48051980 | 7.59e-05 | 0.807 | `TGTGCTGGGTCAGTGA` |
| UP00009\_2 | chr10 | + | 104144014 | 104144029 | 7.62e-05 | 0.807 | `GGCGCTGGGTCAGCGG` |
| UP00009\_2 | chr14 | − | 22071356 | 22071371 | 7.62e-05 | 0.807 | `GGGATGGGGTCAACAT` |
| UP00009\_2 | chr11 | + | 72765805 | 72765820 | 7.66e-05 | 0.807 | `TCACTGGGGTCACGAA` |
| UP00009\_2 | chr7 | − | 999351 | 999366 | 7.73e-05 | 0.807 | `CTGTCTGGGTCAGGCC` |
| UP00009\_2 | chr4 | − | 122280294 | 122280309 | 7.83e-05 | 0.807 | `ACAGTGGGGTCAAACT` |
| UP00009\_2 | chr22 | + | 48052234 | 48052249 | 7.97e-05 | 0.807 | `GTGCTGGGGTCAGCAA` |
| UP00009\_2 | chr8 | − | 61987041 | 61987056 | 8.01e-05 | 0.807 | `GTAACTGGGTCACTAA` |
| UP00009\_2 | chr9 | + | 67903541 | 67903556 | 8.01e-05 | 0.807 | `CCTCCGGGGTCACGAG` |
| UP00009\_2 | chr6 | − | 309989 | 310004 | 8.04e-05 | 0.807 | `GGCCGGGGGTCAGCAC` |
| UP00009\_2 | chrX | − | 48654371 | 48654386 | 8.04e-05 | 0.807 | `CCCGCCAGGTCAAAGT` |
| UP00009\_2 | chr17 | + | 35166471 | 35166486 | 8.04e-05 | 0.807 | `CAAAGGGGGTCAGGAT` |
| UP00009\_2 | chr19 | − | 19127330 | 19127345 | 8.04e-05 | 0.807 | `TGGAGGGGGTCAGTGA` |
| UP00009\_2 | chr18 | − | 45267817 | 45267832 | 8.08e-05 | 0.807 | `GTAGTCAGGTCAGGCT` |
| UP00009\_2 | chr11 | − | 64621010 | 64621025 | 8.15e-05 | 0.807 | `GTGTTCGGGTCAGGAG` |
| UP00009\_2 | chr12 | − | 120810973 | 120810988 | 8.15e-05 | 0.807 | `GGAGCTGGGTCACCGG` |
| UP00009\_2 | chr20 | + | 55184091 | 55184106 | 8.15e-05 | 0.807 | `GCATTCGGGTCAGGGC` |
| UP00009\_2 | chr6 | − | 26071015 | 26071030 | 8.18e-05 | 0.807 | `CTTCCTGGGTCAGGAA` |
| UP00009\_2 | chr11 | − | 103305555 | 103305570 | 8.22e-05 | 0.807 | `CACACTGGGTCACAGT` |
| UP00009\_2 | chr6 | + | 33237273 | 33237288 | 8.25e-05 | 0.807 | `CCTTCGGGGTCAGGGG` |
| UP00009\_2 | chr12 | + | 47810585 | 47810600 | 8.32e-05 | 0.807 | `AGATCTGGGTCAAGAT` |
| UP00009\_2 | chr12 | + | 98251426 | 98251441 | 8.43e-05 | 0.807 | `TTCTTTGGGTCACCAA` |
| UP00009\_2 | chr19 | + | 10565758 | 10565773 | 8.47e-05 | 0.807 | `TACTTGGGGTCAGAGA` |
| UP00009\_2 | chr5 | − | 172319177 | 172319192 | 8.5e-05 | 0.807 | `GTCGGGAGGTCACTCA` |
| UP00009\_2 | chr14 | − | 105306869 | 105306884 | 8.5e-05 | 0.807 | `AGAACCAGGTCAGCCT` |
| UP00009\_2 | chr22 | − | 36010269 | 36010284 | 8.5e-05 | 0.807 | `ATGGCTGGGTCACTGC` |
| UP00009\_2 | chr1 | − | 28752055 | 28752070 | 8.54e-05 | 0.807 | `CGGCCCGGGTCGGGCA` |
| UP00009\_2 | chr15 | − | 57623415 | 57623430 | 8.54e-05 | 0.807 | `TCTTTGGGGTCACCTT` |
| UP00009\_2 | chr7 | + | 4689514 | 4689529 | 8.58e-05 | 0.807 | `TCCCTGGGGTCATCTA` |
| UP00009\_2 | chr16 | + | 30794161 | 30794176 | 8.65e-05 | 0.807 | `CTCGGCGGGTCGGGAA` |
| UP00009\_2 | chr4 | − | 2759311 | 2759326 | 8.69e-05 | 0.807 | `CACGCCAGGTCAAGGG` |
| UP00009\_2 | chr10 | − | 42598253 | 42598268 | 8.69e-05 | 0.807 | `CTCCTCAGGTCAGCAT` |
| UP00009\_2 | chr20 | + | 45820731 | 45820746 | 8.76e-05 | 0.807 | `GGCCCCAGGTCAGACT` |
| UP00009\_2 | chr8 | + | 125688762 | 125688777 | 8.83e-05 | 0.807 | `CATCTGGGGTCATTTA` |
| UP00009\_2 | chr10 | − | 121454195 | 121454210 | 8.83e-05 | 0.807 | `CTTTTCGGGTCATCAG` |
| UP00009\_2 | chr14 | + | 64840502 | 64840517 | 8.83e-05 | 0.807 | `AACCTGGGGTCAGGTG` |
| UP00009\_2 | chr16 | − | 11678793 | 11678808 | 8.87e-05 | 0.807 | `AGAGCTGGGTCAGGGG` |
| UP00009\_2 | chr7 | − | 104411817 | 104411832 | 8.98e-05 | 0.807 | `GAAAGCGGGTCACAGA` |
| UP00009\_2 | chr15 | + | 86983759 | 86983774 | 8.98e-05 | 0.807 | `GAACCCGGGTCAGCGG` |
| UP00009\_2 | chr4 | − | 15365029 | 15365044 | 9.02e-05 | 0.807 | `ACAATGGGGTCACACT` |
| UP00009\_2 | chr6 | + | 21695931 | 21695946 | 9.06e-05 | 0.807 | `CACCCCGGGTCGAGTA` |
| UP00009\_2 | chr5 | + | 88675302 | 88675317 | 9.1e-05 | 0.807 | `AGTAGGGGGTCAGTAA` |
| UP00009\_2 | chr3 | − | 49257229 | 49257244 | 9.14e-05 | 0.807 | `AGGCTGGGGTCAGAAA` |
| UP00009\_2 | chr6 | − | 20985839 | 20985854 | 9.14e-05 | 0.807 | `AATTTCGGGTCACAAA` |
| UP00009\_2 | chr2 | − | 8361122 | 8361137 | 9.17e-05 | 0.807 | `TGCACGAGGTCAGGGT` |
| UP00009\_2 | chr11 | + | 13441572 | 13441587 | 9.17e-05 | 0.807 | `CAGCTCGGGTCAACCG` |
| UP00009\_2 | chr6 | − | 3313040 | 3313055 | 9.29e-05 | 0.807 | `CAGTTGGGGTCACTGG` |
| UP00009\_2 | chr8 | + | 61986951 | 61986966 | 9.29e-05 | 0.807 | `ATGGCTGGGTCATTTT` |
| UP00009\_2 | chr20 | + | 48560025 | 48560040 | 9.29e-05 | 0.807 | `CGGGCCGGGTCGGGGC` |
| UP00009\_2 | chr17 | − | 38024484 | 38024499 | 9.33e-05 | 0.807 | `TCCCTGGGGTCACATC` |
| UP00009\_2 | chr7 | − | 86686799 | 86686814 | 9.45e-05 | 0.807 | `CTCGGGAGGTCATGCT` |
| UP00009\_2 | chr22 | + | 48052032 | 48052047 | 9.49e-05 | 0.807 | `GTGCTGGGGTCAGAGA` |
| UP00009\_2 | chr6 | + | 106664956 | 106664971 | 9.53e-05 | 0.807 | `GAAGCTGGGTCAGGGA` |
| UP00009\_2 | chr1 | − | 12034719 | 12034734 | 9.57e-05 | 0.807 | `ATGACCAGGTCAGGCA` |
| UP00009\_2 | chr5 | + | 78089855 | 78089870 | 9.57e-05 | 0.807 | `ACCTTGGGGTCAAAAA` |
| UP00009\_2 | chrX | − | 119643318 | 119643333 | 9.57e-05 | 0.807 | `CCTGCTGGGTCAATGA` |
| UP00009\_2 | chr3 | + | 109327536 | 109327551 | 9.6e-05 | 0.807 | `GGACTGGGGTCACAGG` |
| UP00009\_2 | chr6 | − | 7829640 | 7829655 | 9.6e-05 | 0.807 | `TGGCGGGGGTCAAGAT` |
| UP00009\_2 | chr5 | − | 139907924 | 139907939 | 9.68e-05 | 0.807 | `CGGCTGAGGTCACGCT` |
| UP00009\_2 | chr6 | + | 2736666 | 2736681 | 9.68e-05 | 0.807 | `AGCTCCAGGTCACCAG` |
| UP00009\_2 | chr10 | + | 121413759 | 121413774 | 9.73e-05 | 0.807 | `CATCTGGGGTCATTTT` |
| UP00009\_2 | chr19 | − | 12755866 | 12755881 | 9.73e-05 | 0.807 | `ACCCGCGGGTCAGGAC` |
| UP00009\_2 | chr9 | + | 36995900 | 36995915 | 9.77e-05 | 0.807 | `TCTGTCGGGTCACAGC` |
| UP00009\_2 | chr11 | − | 1830778 | 1830793 | 9.77e-05 | 0.807 | `CGGCTTGGGTCAGGGT` |
| UP00009\_2 | chr6 | + | 26070801 | 26070816 | 9.81e-05 | 0.807 | `GTGTTGGGGTCACATG` |
| UP00009\_2 | chr7 | − | 154720881 | 154720896 | 9.81e-05 | 0.807 | `AGAAGGGGGTCATCAA` |
| UP00009\_2 | chr11 | − | 65112661 | 65112676 | 9.81e-05 | 0.807 | `GTGTCGGGGTCATGGG` |
| UP00009\_2 | chr11 | − | 76017286 | 76017301 | 9.81e-05 | 0.807 | `CAACTCGGGTCATAAT` |
| UP00009\_2 | chr14 | − | 105306571 | 105306586 | 9.81e-05 | 0.807 | `CTCTCGGGGTCGCGCG` |
| UP00009\_2 | chr1 | − | 120065774 | 120065789 | 9.85e-05 | 0.807 | `TGAACCAGGTCACAGA` |
| UP00009\_2 | chr1 | + | 42045221 | 42045236 | 9.93e-05 | 0.807 | `GTCACTGGGTCATCGA` |
| UP00009\_2 | chr3 | − | 4994146 | 4994161 | 9.93e-05 | 0.807 | `AAAATGGGGTCACTGC` |

---

**DEBUGGING INFORMATION**


---

Command line:

```
/ebi/sw/MEME/VM-cluster410/meme-versions/4.10.0/bin/fimo --parse-genomic-coord --verbosity 1 --oc fimo_out_19 --bgfile ./background --motif UP00009_2 db/uniprobe_mouse.meme ./Supplementary_Table_1.500bp.fa
```

Settings:

```
|  |  |  |
| --- | --- | --- |
| output directory = fimo_out_19 | MEME file name = db/uniprobe_mouse.meme | sequence file name = ./Supplementary_Table_1.500bp.fa |
| background file name = ./background | allow clobber = true | compute q-values = true |
| parse genomic coord. = true | text only = false | scan both strands = true |
| max sequence length = 250000000 | output threshold = 0.0001 | threshold type = p-value |
| max stored scores = 100000 | pseudocount = 0.1 | verbosity = 1 |
| selected motif = UP00009_2 |  |  |
```

This information can be useful in the event you wish to report a
problem with the FIMO software.

---

**Go to top**
