# Supplementary material for: The MEME Suite
Source: Nucleic Acids Res. 2015 May 7;43(Web Server issue):W39–49. doi: 10.1093/nar/gkv416 (PMC4489269; doi:10.1093/nar/gkv416)
Supplement: SUPPLEMENTARY DATA [file supp_gkv416_nar-00283-web-b-2015-File005.zip › case4/meme-chip/fimo_out_2/fimo.html]

FIMO Results


---

|  |  |  |
| --- | --- | --- |
| **Database and Motifs** | **High-scoring Motif Occurrences** | **Debugging Information** |

  
  

---

**FIMO - Motif search tool**


---

FIMO version 4.10.0,
(Release date: Wed May 21 10:35:36 2014 +1000)

For further information on how to interpret these results
or to get a copy of the FIMO software please access
http://meme.nbcr.net

If you use FIMO in your research, please cite the following paper:  
Charles E. Grant, Timothy L. Bailey, and William Stafford Noble,
"FIMO: Scanning for occurrences of a given motif",
*Bioinformatics*, **27**(7):1017-1018, 2011.
[full text]

---

**DATABASE AND MOTIFS**


---

DATABASE
./Supplementary\_Table\_1.500bp.fa  
Database contains
2776
sequences,
1388000
residues

MOTIFS
dreme\_out/dreme.xml
(nucleotide)

| MOTIF | WIDTH | BEST POSSIBLE MATCH |
| --- | --- | --- |
| GGAARY | 6 | GGAAGT |
| AVTGAAA | 7 | ACTGAAA |
| RCAGCTGY | 8 | GCAGCTGC |
| AKAAAH | 6 | AGAAAA |
| RAGKTCA | 7 | GAGGTCA |
| CMCAGM | 6 | CCCAGC |
| CCCCRCCC | 8 | CCCCGCCC |
| AAATR | 5 | AAATG |
| GAAASCA | 7 | GAAAGCA |
| CCGSCTCC | 8 | CCGCCTCC |
| CCWCCTGC | 8 | CCACCTGC |

Random model letter frequencies
(from ./background):
  
A 0.241 C 0.259 G 0.259 T 0.241

---

**SECTION I: HIGH-SCORING MOTIF OCCURRENCES**


---

- There were
  0
  motif occurrences with a
  p-value less than
  0.0001.
- The p-value of a motif occurrence is defined as the
  probability of a random sequence of the same length as the motif
  matching that position of the sequence with as good or better a score.
- The score for the match of a position in a sequence to a motif
  is computed by summing the appropriate entries from each column of
  the position-dependent scoring matrix that represents the motif.
- The table is sorted by increasing p-value.

| Motif | Sequence Name | Strand | Start | End | p-value | Matched Sequence |
| --- | --- | --- | --- | --- | --- | --- |

---

**DEBUGGING INFORMATION**


---

Command line:

```
/ebi/sw/MEME/VM-cluster410/meme-versions/4.10.0/bin/fimo --parse-genomic-coord --verbosity 1 --oc fimo_out_2 --bgfile ./background --motif GGAARY dreme_out/dreme.xml ./Supplementary_Table_1.500bp.fa
```

Settings:

```
|  |  |  |
| --- | --- | --- |
| output directory = fimo_out_2 | MEME file name = dreme_out/dreme.xml | sequence file name = ./Supplementary_Table_1.500bp.fa |
| background file name = ./background | allow clobber = true | compute q-values = true |
| parse genomic coord. = true | text only = false | scan both strands = true |
| max sequence length = 250000000 | output threshold = 0.0001 | threshold type = p-value |
| max stored scores = 100000 | pseudocount = 0.1 | verbosity = 1 |
| selected motif = GGAARY |  |  |
```

This information can be useful in the event you wish to report a
problem with the FIMO software.

---

**Go to top**
