# Supplementary material for: The MEME Suite
Source: Nucleic Acids Res. 2015 May 7;43(Web Server issue):W39–49. doi: 10.1093/nar/gkv416 (PMC4489269; doi:10.1093/nar/gkv416)
Supplement: SUPPLEMENTARY DATA [file supp_gkv416_nar-00283-web-b-2015-File005.zip › case4/meme-chip/fimo_out_3/fimo.html]

FIMO Results


---

|  |  |  |
| --- | --- | --- |
| **Database and Motifs** | **High-scoring Motif Occurrences** | **Debugging Information** |

  
  

---

**FIMO - Motif search tool**


---

FIMO version 4.10.0,
(Release date: Wed May 21 10:35:36 2014 +1000)

For further information on how to interpret these results
or to get a copy of the FIMO software please access
http://meme.nbcr.net

If you use FIMO in your research, please cite the following paper:  
Charles E. Grant, Timothy L. Bailey, and William Stafford Noble,
"FIMO: Scanning for occurrences of a given motif",
*Bioinformatics*, **27**(7):1017-1018, 2011.
[full text]

---

**DATABASE AND MOTIFS**


---

DATABASE
./Supplementary\_Table\_1.500bp.fa  
Database contains
2776
sequences,
1388000
residues

MOTIFS
dreme\_out/dreme.xml
(nucleotide)

| MOTIF | WIDTH | BEST POSSIBLE MATCH |
| --- | --- | --- |
| GGAARY | 6 | GGAAGT |
| AVTGAAA | 7 | ACTGAAA |
| RCAGCTGY | 8 | GCAGCTGC |
| AKAAAH | 6 | AGAAAA |
| RAGKTCA | 7 | GAGGTCA |
| CMCAGM | 6 | CCCAGC |
| CCCCRCCC | 8 | CCCCGCCC |
| AAATR | 5 | AAATG |
| GAAASCA | 7 | GAAAGCA |
| CCGSCTCC | 8 | CCGCCTCC |
| CCWCCTGC | 8 | CCACCTGC |

Random model letter frequencies
(from ./background):
  
A 0.241 C 0.259 G 0.259 T 0.241

---

**SECTION I: HIGH-SCORING MOTIF OCCURRENCES**


---

- There were
  439
  motif occurrences with a
  p-value less than
  0.0001.
- The p-value of a motif occurrence is defined as the
  probability of a random sequence of the same length as the motif
  matching that position of the sequence with as good or better a score.
- The score for the match of a position in a sequence to a motif
  is computed by summing the appropriate entries from each column of
  the position-dependent scoring matrix that represents the motif.
- The q-value of a motif occurrence is defined as the
  false discovery rate if the occurrence is accepted as significant.
- The table is sorted by increasing p-value.

| Motif | Sequence Name | Strand | Start | End | p-value | q-value | Matched Sequence |
| --- | --- | --- | --- | --- | --- | --- | --- |
| AVTGAAA | chr1 | + | 12034990 | 12034996 | 5.43e-05 | 0.269 | `ACTGAAA` |
| AVTGAAA | chr1 | − | 21489946 | 21489952 | 5.43e-05 | 0.269 | `ACTGAAA` |
| AVTGAAA | chr1 | − | 22318164 | 22318170 | 5.43e-05 | 0.269 | `ACTGAAA` |
| AVTGAAA | chr1 | + | 28083599 | 28083605 | 5.43e-05 | 0.269 | `ACTGAAA` |
| AVTGAAA | chr1 | + | 28398874 | 28398880 | 5.43e-05 | 0.269 | `ACTGAAA` |
| AVTGAAA | chr1 | + | 28847893 | 28847899 | 5.43e-05 | 0.269 | `ACTGAAA` |
| AVTGAAA | chr1 | − | 30992961 | 30992967 | 5.43e-05 | 0.269 | `ACTGAAA` |
| AVTGAAA | chr1 | + | 39054322 | 39054328 | 5.43e-05 | 0.269 | `ACTGAAA` |
| AVTGAAA | chr1 | + | 48998190 | 48998196 | 5.43e-05 | 0.269 | `ACTGAAA` |
| AVTGAAA | chr1 | + | 67913144 | 67913150 | 5.43e-05 | 0.269 | `ACTGAAA` |
| AVTGAAA | chr1 | + | 67998428 | 67998434 | 5.43e-05 | 0.269 | `ACTGAAA` |
| AVTGAAA | chr1 | − | 76034431 | 76034437 | 5.43e-05 | 0.269 | `ACTGAAA` |
| AVTGAAA | chr1 | + | 76218515 | 76218521 | 5.43e-05 | 0.269 | `ACTGAAA` |
| AVTGAAA | chr1 | − | 84744965 | 84744971 | 5.43e-05 | 0.269 | `ACTGAAA` |
| AVTGAAA | chr1 | − | 101645948 | 101645954 | 5.43e-05 | 0.269 | `ACTGAAA` |
| AVTGAAA | chr1 | + | 108051174 | 108051180 | 5.43e-05 | 0.269 | `ACTGAAA` |
| AVTGAAA | chr1 | + | 110378572 | 110378578 | 5.43e-05 | 0.269 | `ACTGAAA` |
| AVTGAAA | chr1 | + | 110378578 | 110378584 | 5.43e-05 | 0.269 | `ACTGAAA` |
| AVTGAAA | chr1 | − | 111548890 | 111548896 | 5.43e-05 | 0.269 | `ACTGAAA` |
| AVTGAAA | chr1 | + | 112040771 | 112040777 | 5.43e-05 | 0.269 | `ACTGAAA` |
| AVTGAAA | chr1 | − | 120065996 | 120066002 | 5.43e-05 | 0.269 | `ACTGAAA` |
| AVTGAAA | chr1 | + | 144094378 | 144094384 | 5.43e-05 | 0.269 | `ACTGAAA` |
| AVTGAAA | chr1 | − | 148125141 | 148125147 | 5.43e-05 | 0.269 | `ACTGAAA` |
| AVTGAAA | chr1 | − | 155418498 | 155418504 | 5.43e-05 | 0.269 | `ACTGAAA` |
| AVTGAAA | chr1 | + | 158860250 | 158860256 | 5.43e-05 | 0.269 | `ACTGAAA` |
| AVTGAAA | chr1 | + | 171448815 | 171448821 | 5.43e-05 | 0.269 | `ACTGAAA` |
| AVTGAAA | chr1 | + | 171449141 | 171449147 | 5.43e-05 | 0.269 | `ACTGAAA` |
| AVTGAAA | chr1 | + | 172099139 | 172099145 | 5.43e-05 | 0.269 | `ACTGAAA` |
| AVTGAAA | chr1 | − | 190812427 | 190812433 | 5.43e-05 | 0.269 | `ACTGAAA` |
| AVTGAAA | chr1 | + | 195123863 | 195123869 | 5.43e-05 | 0.269 | `ACTGAAA` |
| AVTGAAA | chr1 | + | 202742504 | 202742510 | 5.43e-05 | 0.269 | `ACTGAAA` |
| AVTGAAA | chr1 | − | 204458320 | 204458326 | 5.43e-05 | 0.269 | `ACTGAAA` |
| AVTGAAA | chr1 | − | 210173035 | 210173041 | 5.43e-05 | 0.269 | `ACTGAAA` |
| AVTGAAA | chr1 | − | 228325955 | 228325961 | 5.43e-05 | 0.269 | `ACTGAAA` |
| AVTGAAA | chr1 | − | 228391137 | 228391143 | 5.43e-05 | 0.269 | `ACTGAAA` |
| AVTGAAA | chr1 | + | 232899377 | 232899383 | 5.43e-05 | 0.269 | `ACTGAAA` |
| AVTGAAA | chr1 | − | 243201383 | 243201389 | 5.43e-05 | 0.269 | `ACTGAAA` |
| AVTGAAA | chr2 | + | 6403290 | 6403296 | 5.43e-05 | 0.269 | `ACTGAAA` |
| AVTGAAA | chr2 | + | 9838038 | 9838044 | 5.43e-05 | 0.269 | `ACTGAAA` |
| AVTGAAA | chr2 | − | 12786110 | 12786116 | 5.43e-05 | 0.269 | `ACTGAAA` |
| AVTGAAA | chr2 | − | 33575995 | 33576001 | 5.43e-05 | 0.269 | `ACTGAAA` |
| AVTGAAA | chr2 | − | 37735672 | 37735678 | 5.43e-05 | 0.269 | `ACTGAAA` |
| AVTGAAA | chr2 | − | 38015263 | 38015269 | 5.43e-05 | 0.269 | `ACTGAAA` |
| AVTGAAA | chr2 | − | 38015407 | 38015413 | 5.43e-05 | 0.269 | `ACTGAAA` |
| AVTGAAA | chr2 | − | 38015441 | 38015447 | 5.43e-05 | 0.269 | `ACTGAAA` |
| AVTGAAA | chr2 | − | 44812213 | 44812219 | 5.43e-05 | 0.269 | `ACTGAAA` |
| AVTGAAA | chr2 | + | 54650601 | 54650607 | 5.43e-05 | 0.269 | `ACTGAAA` |
| AVTGAAA | chr2 | + | 54660762 | 54660768 | 5.43e-05 | 0.269 | `ACTGAAA` |
| AVTGAAA | chr2 | − | 70167847 | 70167853 | 5.43e-05 | 0.269 | `ACTGAAA` |
| AVTGAAA | chr2 | − | 74051866 | 74051872 | 5.43e-05 | 0.269 | `ACTGAAA` |
| AVTGAAA | chr2 | − | 98453909 | 98453915 | 5.43e-05 | 0.269 | `ACTGAAA` |
| AVTGAAA | chr2 | + | 114360850 | 114360856 | 5.43e-05 | 0.269 | `ACTGAAA` |
| AVTGAAA | chr2 | − | 122005179 | 122005185 | 5.43e-05 | 0.269 | `ACTGAAA` |
| AVTGAAA | chr2 | + | 125846142 | 125846148 | 5.43e-05 | 0.269 | `ACTGAAA` |
| AVTGAAA | chr2 | − | 125872067 | 125872073 | 5.43e-05 | 0.269 | `ACTGAAA` |
| AVTGAAA | chr2 | − | 129273129 | 129273135 | 5.43e-05 | 0.269 | `ACTGAAA` |
| AVTGAAA | chr2 | − | 156082281 | 156082287 | 5.43e-05 | 0.269 | `ACTGAAA` |
| AVTGAAA | chr2 | − | 156082481 | 156082487 | 5.43e-05 | 0.269 | `ACTGAAA` |
| AVTGAAA | chr2 | − | 158006547 | 158006553 | 5.43e-05 | 0.269 | `ACTGAAA` |
| AVTGAAA | chr2 | − | 161269160 | 161269166 | 5.43e-05 | 0.269 | `ACTGAAA` |
| AVTGAAA | chr2 | − | 161269172 | 161269178 | 5.43e-05 | 0.269 | `ACTGAAA` |
| AVTGAAA | chr2 | + | 169060414 | 169060420 | 5.43e-05 | 0.269 | `ACTGAAA` |
| AVTGAAA | chr2 | − | 177572777 | 177572783 | 5.43e-05 | 0.269 | `ACTGAAA` |
| AVTGAAA | chr2 | − | 179103912 | 179103918 | 5.43e-05 | 0.269 | `ACTGAAA` |
| AVTGAAA | chr2 | + | 201690377 | 201690383 | 5.43e-05 | 0.269 | `ACTGAAA` |
| AVTGAAA | chr2 | − | 201796659 | 201796665 | 5.43e-05 | 0.269 | `ACTGAAA` |
| AVTGAAA | chr2 | + | 213589002 | 213589008 | 5.43e-05 | 0.269 | `ACTGAAA` |
| AVTGAAA | chr2 | − | 227409449 | 227409455 | 5.43e-05 | 0.269 | `ACTGAAA` |
| AVTGAAA | chr2 | − | 231232165 | 231232171 | 5.43e-05 | 0.269 | `ACTGAAA` |
| AVTGAAA | chr2 | − | 232600041 | 232600047 | 5.43e-05 | 0.269 | `ACTGAAA` |
| AVTGAAA | chr2 | − | 233673669 | 233673675 | 5.43e-05 | 0.269 | `ACTGAAA` |
| AVTGAAA | chr2 | − | 238014458 | 238014464 | 5.43e-05 | 0.269 | `ACTGAAA` |
| AVTGAAA | chr3 | − | 314771 | 314777 | 5.43e-05 | 0.269 | `ACTGAAA` |
| AVTGAAA | chr3 | + | 13107981 | 13107987 | 5.43e-05 | 0.269 | `ACTGAAA` |
| AVTGAAA | chr3 | + | 15378057 | 15378063 | 5.43e-05 | 0.269 | `ACTGAAA` |
| AVTGAAA | chr3 | − | 15378246 | 15378252 | 5.43e-05 | 0.269 | `ACTGAAA` |
| AVTGAAA | chr3 | − | 17198934 | 17198940 | 5.43e-05 | 0.269 | `ACTGAAA` |
| AVTGAAA | chr3 | − | 23670239 | 23670245 | 5.43e-05 | 0.269 | `ACTGAAA` |
| AVTGAAA | chr3 | + | 40977971 | 40977977 | 5.43e-05 | 0.269 | `ACTGAAA` |
| AVTGAAA | chr3 | − | 48909679 | 48909685 | 5.43e-05 | 0.269 | `ACTGAAA` |
| AVTGAAA | chr3 | + | 52005166 | 52005172 | 5.43e-05 | 0.269 | `ACTGAAA` |
| AVTGAAA | chr3 | − | 113661765 | 113661771 | 5.43e-05 | 0.269 | `ACTGAAA` |
| AVTGAAA | chr3 | − | 113662214 | 113662220 | 5.43e-05 | 0.269 | `ACTGAAA` |
| AVTGAAA | chr3 | + | 123304350 | 123304356 | 5.43e-05 | 0.269 | `ACTGAAA` |
| AVTGAAA | chr3 | − | 123811865 | 123811871 | 5.43e-05 | 0.269 | `ACTGAAA` |
| AVTGAAA | chr3 | − | 128952131 | 128952137 | 5.43e-05 | 0.269 | `ACTGAAA` |
| AVTGAAA | chr3 | − | 128957007 | 128957013 | 5.43e-05 | 0.269 | `ACTGAAA` |
| AVTGAAA | chr3 | + | 153533398 | 153533404 | 5.43e-05 | 0.269 | `ACTGAAA` |
| AVTGAAA | chr3 | − | 153533335 | 153533341 | 5.43e-05 | 0.269 | `ACTGAAA` |
| AVTGAAA | chr3 | − | 153533456 | 153533462 | 5.43e-05 | 0.269 | `ACTGAAA` |
| AVTGAAA | chr3 | − | 158289285 | 158289291 | 5.43e-05 | 0.269 | `ACTGAAA` |
| AVTGAAA | chr3 | + | 169252374 | 169252380 | 5.43e-05 | 0.269 | `ACTGAAA` |
| AVTGAAA | chr3 | + | 169252451 | 169252457 | 5.43e-05 | 0.269 | `ACTGAAA` |
| AVTGAAA | chr3 | + | 181479333 | 181479339 | 5.43e-05 | 0.269 | `ACTGAAA` |
| AVTGAAA | chr3 | + | 184727902 | 184727908 | 5.43e-05 | 0.269 | `ACTGAAA` |
| AVTGAAA | chr3 | − | 187984062 | 187984068 | 5.43e-05 | 0.269 | `ACTGAAA` |
| AVTGAAA | chr3 | − | 188216641 | 188216647 | 5.43e-05 | 0.269 | `ACTGAAA` |
| AVTGAAA | chr3 | − | 190689924 | 190689930 | 5.43e-05 | 0.269 | `ACTGAAA` |
| AVTGAAA | chr3 | − | 196740679 | 196740685 | 5.43e-05 | 0.269 | `ACTGAAA` |
| AVTGAAA | chr3 | − | 196746255 | 196746261 | 5.43e-05 | 0.269 | `ACTGAAA` |
| AVTGAAA | chr3 | − | 197307833 | 197307839 | 5.43e-05 | 0.269 | `ACTGAAA` |
| AVTGAAA | chr3 | + | 199168216 | 199168222 | 5.43e-05 | 0.269 | `ACTGAAA` |
| AVTGAAA | chr4 | + | 7824576 | 7824582 | 5.43e-05 | 0.269 | `ACTGAAA` |
| AVTGAAA | chr4 | + | 54309379 | 54309385 | 5.43e-05 | 0.269 | `ACTGAAA` |
| AVTGAAA | chr4 | + | 77441946 | 77441952 | 5.43e-05 | 0.269 | `ACTGAAA` |
| AVTGAAA | chr4 | − | 77441725 | 77441731 | 5.43e-05 | 0.269 | `ACTGAAA` |
| AVTGAAA | chr4 | − | 78294463 | 78294469 | 5.43e-05 | 0.269 | `ACTGAAA` |
| AVTGAAA | chr4 | − | 79754390 | 79754396 | 5.43e-05 | 0.269 | `ACTGAAA` |
| AVTGAAA | chr4 | − | 79779179 | 79779185 | 5.43e-05 | 0.269 | `ACTGAAA` |
| AVTGAAA | chr4 | + | 95123489 | 95123495 | 5.43e-05 | 0.269 | `ACTGAAA` |
| AVTGAAA | chr4 | − | 116475578 | 116475584 | 5.43e-05 | 0.269 | `ACTGAAA` |
| AVTGAAA | chr4 | + | 121207708 | 121207714 | 5.43e-05 | 0.269 | `ACTGAAA` |
| AVTGAAA | chr4 | + | 121207756 | 121207762 | 5.43e-05 | 0.269 | `ACTGAAA` |
| AVTGAAA | chr4 | − | 121207680 | 121207686 | 5.43e-05 | 0.269 | `ACTGAAA` |
| AVTGAAA | chr4 | + | 122280231 | 122280237 | 5.43e-05 | 0.269 | `ACTGAAA` |
| AVTGAAA | chr4 | + | 145688438 | 145688444 | 5.43e-05 | 0.269 | `ACTGAAA` |
| AVTGAAA | chr4 | − | 146332161 | 146332167 | 5.43e-05 | 0.269 | `ACTGAAA` |
| AVTGAAA | chr4 | + | 152885323 | 152885329 | 5.43e-05 | 0.269 | `ACTGAAA` |
| AVTGAAA | chr4 | + | 154628885 | 154628891 | 5.43e-05 | 0.269 | `ACTGAAA` |
| AVTGAAA | chr4 | + | 160318013 | 160318019 | 5.43e-05 | 0.269 | `ACTGAAA` |
| AVTGAAA | chr5 | + | 1369181 | 1369187 | 5.43e-05 | 0.269 | `ACTGAAA` |
| AVTGAAA | chr5 | + | 1369187 | 1369193 | 5.43e-05 | 0.269 | `ACTGAAA` |
| AVTGAAA | chr5 | − | 32622295 | 32622301 | 5.43e-05 | 0.269 | `ACTGAAA` |
| AVTGAAA | chr5 | + | 40442942 | 40442948 | 5.43e-05 | 0.269 | `ACTGAAA` |
| AVTGAAA | chr5 | − | 40871226 | 40871232 | 5.43e-05 | 0.269 | `ACTGAAA` |
| AVTGAAA | chr5 | + | 55474377 | 55474383 | 5.43e-05 | 0.269 | `ACTGAAA` |
| AVTGAAA | chr5 | + | 55474607 | 55474613 | 5.43e-05 | 0.269 | `ACTGAAA` |
| AVTGAAA | chr5 | + | 55480347 | 55480353 | 5.43e-05 | 0.269 | `ACTGAAA` |
| AVTGAAA | chr5 | − | 55480197 | 55480203 | 5.43e-05 | 0.269 | `ACTGAAA` |
| AVTGAAA | chr5 | + | 86448890 | 86448896 | 5.43e-05 | 0.269 | `ACTGAAA` |
| AVTGAAA | chr5 | + | 90714325 | 90714331 | 5.43e-05 | 0.269 | `ACTGAAA` |
| AVTGAAA | chr5 | + | 95245466 | 95245472 | 5.43e-05 | 0.269 | `ACTGAAA` |
| AVTGAAA | chr5 | − | 96529051 | 96529057 | 5.43e-05 | 0.269 | `ACTGAAA` |
| AVTGAAA | chr5 | + | 98391147 | 98391153 | 5.43e-05 | 0.269 | `ACTGAAA` |
| AVTGAAA | chr5 | + | 126684105 | 126684111 | 5.43e-05 | 0.269 | `ACTGAAA` |
| AVTGAAA | chr5 | − | 131037998 | 131038004 | 5.43e-05 | 0.269 | `ACTGAAA` |
| AVTGAAA | chr5 | + | 131830045 | 131830051 | 5.43e-05 | 0.269 | `ACTGAAA` |
| AVTGAAA | chr5 | + | 138748850 | 138748856 | 5.43e-05 | 0.269 | `ACTGAAA` |
| AVTGAAA | chr5 | + | 138937064 | 138937070 | 5.43e-05 | 0.269 | `ACTGAAA` |
| AVTGAAA | chr5 | + | 138995876 | 138995882 | 5.43e-05 | 0.269 | `ACTGAAA` |
| AVTGAAA | chr5 | + | 151022678 | 151022684 | 5.43e-05 | 0.269 | `ACTGAAA` |
| AVTGAAA | chr5 | − | 172394970 | 172394976 | 5.43e-05 | 0.269 | `ACTGAAA` |
| AVTGAAA | chr6 | + | 174477 | 174483 | 5.43e-05 | 0.269 | `ACTGAAA` |
| AVTGAAA | chr6 | − | 264423 | 264429 | 5.43e-05 | 0.269 | `ACTGAAA` |
| AVTGAAA | chr6 | + | 273400 | 273406 | 5.43e-05 | 0.269 | `ACTGAAA` |
| AVTGAAA | chr6 | + | 355357 | 355363 | 5.43e-05 | 0.269 | `ACTGAAA` |
| AVTGAAA | chr6 | − | 2736700 | 2736706 | 5.43e-05 | 0.269 | `ACTGAAA` |
| AVTGAAA | chr6 | + | 7828139 | 7828145 | 5.43e-05 | 0.269 | `ACTGAAA` |
| AVTGAAA | chr6 | + | 7829374 | 7829380 | 5.43e-05 | 0.269 | `ACTGAAA` |
| AVTGAAA | chr6 | + | 7834525 | 7834531 | 5.43e-05 | 0.269 | `ACTGAAA` |
| AVTGAAA | chr6 | + | 7862329 | 7862335 | 5.43e-05 | 0.269 | `ACTGAAA` |
| AVTGAAA | chr6 | + | 7862409 | 7862415 | 5.43e-05 | 0.269 | `ACTGAAA` |
| AVTGAAA | chr6 | − | 7862634 | 7862640 | 5.43e-05 | 0.269 | `ACTGAAA` |
| AVTGAAA | chr6 | − | 11942654 | 11942660 | 5.43e-05 | 0.269 | `ACTGAAA` |
| AVTGAAA | chr6 | − | 12119334 | 12119340 | 5.43e-05 | 0.269 | `ACTGAAA` |
| AVTGAAA | chr6 | + | 16529484 | 16529490 | 5.43e-05 | 0.269 | `ACTGAAA` |
| AVTGAAA | chr6 | + | 16581453 | 16581459 | 5.43e-05 | 0.269 | `ACTGAAA` |
| AVTGAAA | chr6 | − | 16581493 | 16581499 | 5.43e-05 | 0.269 | `ACTGAAA` |
| AVTGAAA | chr6 | − | 24829297 | 24829303 | 5.43e-05 | 0.269 | `ACTGAAA` |
| AVTGAAA | chr6 | − | 26129782 | 26129788 | 5.43e-05 | 0.269 | `ACTGAAA` |
| AVTGAAA | chr6 | + | 26265822 | 26265828 | 5.43e-05 | 0.269 | `ACTGAAA` |
| AVTGAAA | chr6 | + | 26313423 | 26313429 | 5.43e-05 | 0.269 | `ACTGAAA` |
| AVTGAAA | chr6 | + | 26358337 | 26358343 | 5.43e-05 | 0.269 | `ACTGAAA` |
| AVTGAAA | chr6 | − | 27222866 | 27222872 | 5.43e-05 | 0.269 | `ACTGAAA` |
| AVTGAAA | chr6 | − | 28734278 | 28734284 | 5.43e-05 | 0.269 | `ACTGAAA` |
| AVTGAAA | chr6 | + | 30189024 | 30189030 | 5.43e-05 | 0.269 | `ACTGAAA` |
| AVTGAAA | chr6 | − | 30565912 | 30565918 | 5.43e-05 | 0.269 | `ACTGAAA` |
| AVTGAAA | chr6 | − | 31728757 | 31728763 | 5.43e-05 | 0.269 | `ACTGAAA` |
| AVTGAAA | chr6 | + | 34732581 | 34732587 | 5.43e-05 | 0.269 | `ACTGAAA` |
| AVTGAAA | chr6 | + | 43005909 | 43005915 | 5.43e-05 | 0.269 | `ACTGAAA` |
| AVTGAAA | chr6 | + | 47513402 | 47513408 | 5.43e-05 | 0.269 | `ACTGAAA` |
| AVTGAAA | chr6 | + | 64272676 | 64272682 | 5.43e-05 | 0.269 | `ACTGAAA` |
| AVTGAAA | chr6 | − | 74282358 | 74282364 | 5.43e-05 | 0.269 | `ACTGAAA` |
| AVTGAAA | chr6 | + | 80381267 | 80381273 | 5.43e-05 | 0.269 | `ACTGAAA` |
| AVTGAAA | chr6 | + | 106653648 | 106653654 | 5.43e-05 | 0.269 | `ACTGAAA` |
| AVTGAAA | chr6 | − | 106656628 | 106656634 | 5.43e-05 | 0.269 | `ACTGAAA` |
| AVTGAAA | chr6 | + | 127081655 | 127081661 | 5.43e-05 | 0.269 | `ACTGAAA` |
| AVTGAAA | chr6 | + | 127081932 | 127081938 | 5.43e-05 | 0.269 | `ACTGAAA` |
| AVTGAAA | chr6 | + | 134610410 | 134610416 | 5.43e-05 | 0.269 | `ACTGAAA` |
| AVTGAAA | chr6 | − | 138312902 | 138312908 | 5.43e-05 | 0.269 | `ACTGAAA` |
| AVTGAAA | chr6 | + | 143166487 | 143166493 | 5.43e-05 | 0.269 | `ACTGAAA` |
| AVTGAAA | chr6 | + | 143170301 | 143170307 | 5.43e-05 | 0.269 | `ACTGAAA` |
| AVTGAAA | chr7 | − | 23024958 | 23024964 | 5.43e-05 | 0.269 | `ACTGAAA` |
| AVTGAAA | chr7 | − | 24924528 | 24924534 | 5.43e-05 | 0.269 | `ACTGAAA` |
| AVTGAAA | chr7 | + | 41991344 | 41991350 | 5.43e-05 | 0.269 | `ACTGAAA` |
| AVTGAAA | chr7 | + | 42875878 | 42875884 | 5.43e-05 | 0.269 | `ACTGAAA` |
| AVTGAAA | chr7 | + | 42876010 | 42876016 | 5.43e-05 | 0.269 | `ACTGAAA` |
| AVTGAAA | chr7 | − | 44070828 | 44070834 | 5.43e-05 | 0.269 | `ACTGAAA` |
| AVTGAAA | chr7 | + | 55605157 | 55605163 | 5.43e-05 | 0.269 | `ACTGAAA` |
| AVTGAAA | chr7 | + | 73270146 | 73270152 | 5.43e-05 | 0.269 | `ACTGAAA` |
| AVTGAAA | chr7 | − | 100515478 | 100515484 | 5.43e-05 | 0.269 | `ACTGAAA` |
| AVTGAAA | chr7 | + | 120706192 | 120706198 | 5.43e-05 | 0.269 | `ACTGAAA` |
| AVTGAAA | chr7 | − | 128362264 | 128362270 | 5.43e-05 | 0.269 | `ACTGAAA` |
| AVTGAAA | chr7 | + | 135300925 | 135300931 | 5.43e-05 | 0.269 | `ACTGAAA` |
| AVTGAAA | chr7 | + | 154722554 | 154722560 | 5.43e-05 | 0.269 | `ACTGAAA` |
| AVTGAAA | chr8 | − | 144179331 | 144179337 | 5.43e-05 | 0.269 | `ACTGAAA` |
| AVTGAAA | chr8 | − | 144179337 | 144179343 | 5.43e-05 | 0.269 | `ACTGAAA` |
| AVTGAAA | chr8 | − | 28691099 | 28691105 | 5.43e-05 | 0.269 | `ACTGAAA` |
| AVTGAAA | chr8 | − | 54975231 | 54975237 | 5.43e-05 | 0.269 | `ACTGAAA` |
| AVTGAAA | chr8 | − | 61978828 | 61978834 | 5.43e-05 | 0.269 | `ACTGAAA` |
| AVTGAAA | chr8 | − | 67026282 | 67026288 | 5.43e-05 | 0.269 | `ACTGAAA` |
| AVTGAAA | chr8 | + | 67056801 | 67056807 | 5.43e-05 | 0.269 | `ACTGAAA` |
| AVTGAAA | chr8 | − | 67057123 | 67057129 | 5.43e-05 | 0.269 | `ACTGAAA` |
| AVTGAAA | chr8 | − | 67755440 | 67755446 | 5.43e-05 | 0.269 | `ACTGAAA` |
| AVTGAAA | chr8 | + | 71021139 | 71021145 | 5.43e-05 | 0.269 | `ACTGAAA` |
| AVTGAAA | chr8 | + | 91065474 | 91065480 | 5.43e-05 | 0.269 | `ACTGAAA` |
| AVTGAAA | chr8 | + | 98712810 | 98712816 | 5.43e-05 | 0.269 | `ACTGAAA` |
| AVTGAAA | chr8 | − | 98712718 | 98712724 | 5.43e-05 | 0.269 | `ACTGAAA` |
| AVTGAAA | chr8 | + | 101576219 | 101576225 | 5.43e-05 | 0.269 | `ACTGAAA` |
| AVTGAAA | chr8 | − | 101998368 | 101998374 | 5.43e-05 | 0.269 | `ACTGAAA` |
| AVTGAAA | chr8 | − | 103610918 | 103610924 | 5.43e-05 | 0.269 | `ACTGAAA` |
| AVTGAAA | chr8 | − | 103612016 | 103612022 | 5.43e-05 | 0.269 | `ACTGAAA` |
| AVTGAAA | chr8 | + | 105750469 | 105750475 | 5.43e-05 | 0.269 | `ACTGAAA` |
| AVTGAAA | chr8 | − | 105750896 | 105750902 | 5.43e-05 | 0.269 | `ACTGAAA` |
| AVTGAAA | chr8 | + | 119361045 | 119361051 | 5.43e-05 | 0.269 | `ACTGAAA` |
| AVTGAAA | chr8 | − | 126233257 | 126233263 | 5.43e-05 | 0.269 | `ACTGAAA` |
| AVTGAAA | chr8 | + | 126412327 | 126412333 | 5.43e-05 | 0.269 | `ACTGAAA` |
| AVTGAAA | chr8 | + | 126415603 | 126415609 | 5.43e-05 | 0.269 | `ACTGAAA` |
| AVTGAAA | chr8 | − | 126520017 | 126520023 | 5.43e-05 | 0.269 | `ACTGAAA` |
| AVTGAAA | chr8 | + | 126683596 | 126683602 | 5.43e-05 | 0.269 | `ACTGAAA` |
| AVTGAAA | chr8 | − | 129303672 | 129303678 | 5.43e-05 | 0.269 | `ACTGAAA` |
| AVTGAAA | chr8 | + | 129395546 | 129395552 | 5.43e-05 | 0.269 | `ACTGAAA` |
| AVTGAAA | chr8 | + | 131329446 | 131329452 | 5.43e-05 | 0.269 | `ACTGAAA` |
| AVTGAAA | chr8 | − | 135801782 | 135801788 | 5.43e-05 | 0.269 | `ACTGAAA` |
| AVTGAAA | chr8 | − | 141668304 | 141668310 | 5.43e-05 | 0.269 | `ACTGAAA` |
| AVTGAAA | chr9 | − | 24922954 | 24922960 | 5.43e-05 | 0.269 | `ACTGAAA` |
| AVTGAAA | chr9 | − | 36994561 | 36994567 | 5.43e-05 | 0.269 | `ACTGAAA` |
| AVTGAAA | chr9 | + | 37392909 | 37392915 | 5.43e-05 | 0.269 | `ACTGAAA` |
| AVTGAAA | chr9 | + | 70787823 | 70787829 | 5.43e-05 | 0.269 | `ACTGAAA` |
| AVTGAAA | chr9 | − | 79948738 | 79948744 | 5.43e-05 | 0.269 | `ACTGAAA` |
| AVTGAAA | chr9 | + | 84959132 | 84959138 | 5.43e-05 | 0.269 | `ACTGAAA` |
| AVTGAAA | chr9 | − | 84959159 | 84959165 | 5.43e-05 | 0.269 | `ACTGAAA` |
| AVTGAAA | chr9 | + | 86499119 | 86499125 | 5.43e-05 | 0.269 | `ACTGAAA` |
| AVTGAAA | chr9 | + | 86499342 | 86499348 | 5.43e-05 | 0.269 | `ACTGAAA` |
| AVTGAAA | chr9 | + | 89269479 | 89269485 | 5.43e-05 | 0.269 | `ACTGAAA` |
| AVTGAAA | chr9 | − | 92950064 | 92950070 | 5.43e-05 | 0.269 | `ACTGAAA` |
| AVTGAAA | chr9 | − | 92950158 | 92950164 | 5.43e-05 | 0.269 | `ACTGAAA` |
| AVTGAAA | chr9 | − | 114586046 | 114586052 | 5.43e-05 | 0.269 | `ACTGAAA` |
| AVTGAAA | chr9 | + | 122699599 | 122699605 | 5.43e-05 | 0.269 | `ACTGAAA` |
| AVTGAAA | chr9 | + | 131833301 | 131833307 | 5.43e-05 | 0.269 | `ACTGAAA` |
| AVTGAAA | chr9 | + | 131833384 | 131833390 | 5.43e-05 | 0.269 | `ACTGAAA` |
| AVTGAAA | chrX | + | 7043409 | 7043415 | 5.43e-05 | 0.269 | `ACTGAAA` |
| AVTGAAA | chrX | + | 29483643 | 29483649 | 5.43e-05 | 0.269 | `ACTGAAA` |
| AVTGAAA | chrX | − | 29483674 | 29483680 | 5.43e-05 | 0.269 | `ACTGAAA` |
| AVTGAAA | chrX | − | 41095103 | 41095109 | 5.43e-05 | 0.269 | `ACTGAAA` |
| AVTGAAA | chrX | − | 41095888 | 41095894 | 5.43e-05 | 0.269 | `ACTGAAA` |
| AVTGAAA | chrX | − | 46373135 | 46373141 | 5.43e-05 | 0.269 | `ACTGAAA` |
| AVTGAAA | chrX | − | 46382723 | 46382729 | 5.43e-05 | 0.269 | `ACTGAAA` |
| AVTGAAA | chrX | + | 48654296 | 48654302 | 5.43e-05 | 0.269 | `ACTGAAA` |
| AVTGAAA | chrX | + | 48681098 | 48681104 | 5.43e-05 | 0.269 | `ACTGAAA` |
| AVTGAAA | chrX | + | 119643462 | 119643468 | 5.43e-05 | 0.269 | `ACTGAAA` |
| AVTGAAA | chrX | − | 119643535 | 119643541 | 5.43e-05 | 0.269 | `ACTGAAA` |
| AVTGAAA | chrX | + | 138635583 | 138635589 | 5.43e-05 | 0.269 | `ACTGAAA` |
| AVTGAAA | chr10 | + | 2980823 | 2980829 | 5.43e-05 | 0.269 | `ACTGAAA` |
| AVTGAAA | chr10 | − | 6430189 | 6430195 | 5.43e-05 | 0.269 | `ACTGAAA` |
| AVTGAAA | chr10 | + | 7554121 | 7554127 | 5.43e-05 | 0.269 | `ACTGAAA` |
| AVTGAAA | chr10 | − | 11319987 | 11319993 | 5.43e-05 | 0.269 | `ACTGAAA` |
| AVTGAAA | chr10 | + | 11368593 | 11368599 | 5.43e-05 | 0.269 | `ACTGAAA` |
| AVTGAAA | chr10 | − | 11691617 | 11691623 | 5.43e-05 | 0.269 | `ACTGAAA` |
| AVTGAAA | chr10 | − | 13839529 | 13839535 | 5.43e-05 | 0.269 | `ACTGAAA` |
| AVTGAAA | chr10 | + | 14729972 | 14729978 | 5.43e-05 | 0.269 | `ACTGAAA` |
| AVTGAAA | chr10 | + | 14730234 | 14730240 | 5.43e-05 | 0.269 | `ACTGAAA` |
| AVTGAAA | chr10 | + | 23030626 | 23030632 | 5.43e-05 | 0.269 | `ACTGAAA` |
| AVTGAAA | chr10 | − | 35478423 | 35478429 | 5.43e-05 | 0.269 | `ACTGAAA` |
| AVTGAAA | chr10 | − | 64062327 | 64062333 | 5.43e-05 | 0.269 | `ACTGAAA` |
| AVTGAAA | chr10 | + | 64067250 | 64067256 | 5.43e-05 | 0.269 | `ACTGAAA` |
| AVTGAAA | chr10 | − | 64067292 | 64067298 | 5.43e-05 | 0.269 | `ACTGAAA` |
| AVTGAAA | chr10 | + | 64083130 | 64083136 | 5.43e-05 | 0.269 | `ACTGAAA` |
| AVTGAAA | chr10 | − | 64085413 | 64085419 | 5.43e-05 | 0.269 | `ACTGAAA` |
| AVTGAAA | chr10 | + | 80514005 | 80514011 | 5.43e-05 | 0.269 | `ACTGAAA` |
| AVTGAAA | chr10 | − | 80514154 | 80514160 | 5.43e-05 | 0.269 | `ACTGAAA` |
| AVTGAAA | chr10 | − | 89840658 | 89840664 | 5.43e-05 | 0.269 | `ACTGAAA` |
| AVTGAAA | chr10 | + | 89841484 | 89841490 | 5.43e-05 | 0.269 | `ACTGAAA` |
| AVTGAAA | chr10 | − | 90010441 | 90010447 | 5.43e-05 | 0.269 | `ACTGAAA` |
| AVTGAAA | chr10 | − | 90020893 | 90020899 | 5.43e-05 | 0.269 | `ACTGAAA` |
| AVTGAAA | chr10 | − | 90021075 | 90021081 | 5.43e-05 | 0.269 | `ACTGAAA` |
| AVTGAAA | chr10 | − | 90021096 | 90021102 | 5.43e-05 | 0.269 | `ACTGAAA` |
| AVTGAAA | chr10 | − | 90165719 | 90165725 | 5.43e-05 | 0.269 | `ACTGAAA` |
| AVTGAAA | chr10 | − | 93851031 | 93851037 | 5.43e-05 | 0.269 | `ACTGAAA` |
| AVTGAAA | chr10 | − | 96986162 | 96986168 | 5.43e-05 | 0.269 | `ACTGAAA` |
| AVTGAAA | chr10 | + | 103609208 | 103609214 | 5.43e-05 | 0.269 | `ACTGAAA` |
| AVTGAAA | chr10 | − | 112552751 | 112552757 | 5.43e-05 | 0.269 | `ACTGAAA` |
| AVTGAAA | chr10 | + | 122356010 | 122356016 | 5.43e-05 | 0.269 | `ACTGAAA` |
| AVTGAAA | chr10 | − | 125964592 | 125964598 | 5.43e-05 | 0.269 | `ACTGAAA` |
| AVTGAAA | chr10 | + | 126375346 | 126375352 | 5.43e-05 | 0.269 | `ACTGAAA` |
| AVTGAAA | chr10 | + | 135052736 | 135052742 | 5.43e-05 | 0.269 | `ACTGAAA` |
| AVTGAAA | chr11 | + | 1830880 | 1830886 | 5.43e-05 | 0.269 | `ACTGAAA` |
| AVTGAAA | chr11 | + | 34222514 | 34222520 | 5.43e-05 | 0.269 | `ACTGAAA` |
| AVTGAAA | chr11 | − | 34222347 | 34222353 | 5.43e-05 | 0.269 | `ACTGAAA` |
| AVTGAAA | chr11 | + | 59075170 | 59075176 | 5.43e-05 | 0.269 | `ACTGAAA` |
| AVTGAAA | chr11 | − | 61487716 | 61487722 | 5.43e-05 | 0.269 | `ACTGAAA` |
| AVTGAAA | chr11 | − | 64651083 | 64651089 | 5.43e-05 | 0.269 | `ACTGAAA` |
| AVTGAAA | chr11 | + | 65021395 | 65021401 | 5.43e-05 | 0.269 | `ACTGAAA` |
| AVTGAAA | chr11 | + | 65112337 | 65112343 | 5.43e-05 | 0.269 | `ACTGAAA` |
| AVTGAAA | chr11 | − | 65661845 | 65661851 | 5.43e-05 | 0.269 | `ACTGAAA` |
| AVTGAAA | chr11 | + | 67790709 | 67790715 | 5.43e-05 | 0.269 | `ACTGAAA` |
| AVTGAAA | chr11 | + | 68580457 | 68580463 | 5.43e-05 | 0.269 | `ACTGAAA` |
| AVTGAAA | chr11 | − | 73168048 | 73168054 | 5.43e-05 | 0.269 | `ACTGAAA` |
| AVTGAAA | chr11 | + | 82450920 | 82450926 | 5.43e-05 | 0.269 | `ACTGAAA` |
| AVTGAAA | chr11 | − | 101693171 | 101693177 | 5.43e-05 | 0.269 | `ACTGAAA` |
| AVTGAAA | chr11 | + | 103305642 | 103305648 | 5.43e-05 | 0.269 | `ACTGAAA` |
| AVTGAAA | chr11 | − | 110747271 | 110747277 | 5.43e-05 | 0.269 | `ACTGAAA` |
| AVTGAAA | chr11 | − | 115372343 | 115372349 | 5.43e-05 | 0.269 | `ACTGAAA` |
| AVTGAAA | chr11 | + | 117786182 | 117786188 | 5.43e-05 | 0.269 | `ACTGAAA` |
| AVTGAAA | chr11 | + | 127928030 | 127928036 | 5.43e-05 | 0.269 | `ACTGAAA` |
| AVTGAAA | chr11 | − | 131733432 | 131733438 | 5.43e-05 | 0.269 | `ACTGAAA` |
| AVTGAAA | chr12 | + | 1534014 | 1534020 | 5.43e-05 | 0.269 | `ACTGAAA` |
| AVTGAAA | chr12 | + | 4122452 | 4122458 | 5.43e-05 | 0.269 | `ACTGAAA` |
| AVTGAAA | chr12 | + | 6642719 | 6642725 | 5.43e-05 | 0.269 | `ACTGAAA` |
| AVTGAAA | chr12 | + | 6923115 | 6923121 | 5.43e-05 | 0.269 | `ACTGAAA` |
| AVTGAAA | chr12 | − | 14325422 | 14325428 | 5.43e-05 | 0.269 | `ACTGAAA` |
| AVTGAAA | chr12 | + | 15832477 | 15832483 | 5.43e-05 | 0.269 | `ACTGAAA` |
| AVTGAAA | chr12 | + | 29196415 | 29196421 | 5.43e-05 | 0.269 | `ACTGAAA` |
| AVTGAAA | chr12 | + | 37455742 | 37455748 | 5.43e-05 | 0.269 | `ACTGAAA` |
| AVTGAAA | chr12 | − | 37455652 | 37455658 | 5.43e-05 | 0.269 | `ACTGAAA` |
| AVTGAAA | chr12 | − | 38300177 | 38300183 | 5.43e-05 | 0.269 | `ACTGAAA` |
| AVTGAAA | chr12 | − | 50651727 | 50651733 | 5.43e-05 | 0.269 | `ACTGAAA` |
| AVTGAAA | chr12 | − | 52965824 | 52965830 | 5.43e-05 | 0.269 | `ACTGAAA` |
| AVTGAAA | chr12 | + | 63346453 | 63346459 | 5.43e-05 | 0.269 | `ACTGAAA` |
| AVTGAAA | chr12 | + | 99103469 | 99103475 | 5.43e-05 | 0.269 | `ACTGAAA` |
| AVTGAAA | chr12 | + | 99242550 | 99242556 | 5.43e-05 | 0.269 | `ACTGAAA` |
| AVTGAAA | chr12 | + | 102867408 | 102867414 | 5.43e-05 | 0.269 | `ACTGAAA` |
| AVTGAAA | chr12 | + | 107486112 | 107486118 | 5.43e-05 | 0.269 | `ACTGAAA` |
| AVTGAAA | chr12 | − | 107556310 | 107556316 | 5.43e-05 | 0.269 | `ACTGAAA` |
| AVTGAAA | chr12 | + | 115844469 | 115844475 | 5.43e-05 | 0.269 | `ACTGAAA` |
| AVTGAAA | chr12 | − | 115844569 | 115844575 | 5.43e-05 | 0.269 | `ACTGAAA` |
| AVTGAAA | chr12 | + | 120913647 | 120913653 | 5.43e-05 | 0.269 | `ACTGAAA` |
| AVTGAAA | chr12 | + | 121952425 | 121952431 | 5.43e-05 | 0.269 | `ACTGAAA` |
| AVTGAAA | chr12 | + | 122025299 | 122025305 | 5.43e-05 | 0.269 | `ACTGAAA` |
| AVTGAAA | chr12 | − | 123967769 | 123967775 | 5.43e-05 | 0.269 | `ACTGAAA` |
| AVTGAAA | chr12 | − | 123972014 | 123972020 | 5.43e-05 | 0.269 | `ACTGAAA` |
| AVTGAAA | chr13 | + | 33201036 | 33201042 | 5.43e-05 | 0.269 | `ACTGAAA` |
| AVTGAAA | chr13 | − | 33201016 | 33201022 | 5.43e-05 | 0.269 | `ACTGAAA` |
| AVTGAAA | chr13 | + | 40243303 | 40243309 | 5.43e-05 | 0.269 | `ACTGAAA` |
| AVTGAAA | chr13 | + | 46123465 | 46123471 | 5.43e-05 | 0.269 | `ACTGAAA` |
| AVTGAAA | chr13 | − | 47964088 | 47964094 | 5.43e-05 | 0.269 | `ACTGAAA` |
| AVTGAAA | chr13 | + | 48034372 | 48034378 | 5.43e-05 | 0.269 | `ACTGAAA` |
| AVTGAAA | chr14 | − | 20779473 | 20779479 | 5.43e-05 | 0.269 | `ACTGAAA` |
| AVTGAAA | chr14 | + | 23700280 | 23700286 | 5.43e-05 | 0.269 | `ACTGAAA` |
| AVTGAAA | chr14 | − | 58712052 | 58712058 | 5.43e-05 | 0.269 | `ACTGAAA` |
| AVTGAAA | chr14 | − | 64693332 | 64693338 | 5.43e-05 | 0.269 | `ACTGAAA` |
| AVTGAAA | chr14 | + | 68325477 | 68325483 | 5.43e-05 | 0.269 | `ACTGAAA` |
| AVTGAAA | chr14 | + | 75016642 | 75016648 | 5.43e-05 | 0.269 | `ACTGAAA` |
| AVTGAAA | chr14 | − | 75023705 | 75023711 | 5.43e-05 | 0.269 | `ACTGAAA` |
| AVTGAAA | chr14 | + | 80495997 | 80496003 | 5.43e-05 | 0.269 | `ACTGAAA` |
| AVTGAAA | chr14 | − | 80495692 | 80495698 | 5.43e-05 | 0.269 | `ACTGAAA` |
| AVTGAAA | chr14 | + | 80755612 | 80755618 | 5.43e-05 | 0.269 | `ACTGAAA` |
| AVTGAAA | chr14 | + | 80755785 | 80755791 | 5.43e-05 | 0.269 | `ACTGAAA` |
| AVTGAAA | chr14 | + | 81010317 | 81010323 | 5.43e-05 | 0.269 | `ACTGAAA` |
| AVTGAAA | chr14 | + | 94798366 | 94798372 | 5.43e-05 | 0.269 | `ACTGAAA` |
| AVTGAAA | chr14 | + | 101412743 | 101412749 | 5.43e-05 | 0.269 | `ACTGAAA` |
| AVTGAAA | chr14 | − | 104464101 | 104464107 | 5.43e-05 | 0.269 | `ACTGAAA` |
| AVTGAAA | chr14 | − | 105399307 | 105399313 | 5.43e-05 | 0.269 | `ACTGAAA` |
| AVTGAAA | chr15 | − | 29342910 | 29342916 | 5.43e-05 | 0.269 | `ACTGAAA` |
| AVTGAAA | chr15 | + | 29408536 | 29408542 | 5.43e-05 | 0.269 | `ACTGAAA` |
| AVTGAAA | chr15 | + | 42790872 | 42790878 | 5.43e-05 | 0.269 | `ACTGAAA` |
| AVTGAAA | chr15 | − | 42793005 | 42793011 | 5.43e-05 | 0.269 | `ACTGAAA` |
| AVTGAAA | chr15 | + | 62237100 | 62237106 | 5.43e-05 | 0.269 | `ACTGAAA` |
| AVTGAAA | chr15 | + | 72476787 | 72476793 | 5.43e-05 | 0.269 | `ACTGAAA` |
| AVTGAAA | chr15 | + | 72476920 | 72476926 | 5.43e-05 | 0.269 | `ACTGAAA` |
| AVTGAAA | chr15 | + | 73126667 | 73126673 | 5.43e-05 | 0.269 | `ACTGAAA` |
| AVTGAAA | chr15 | − | 84035697 | 84035703 | 5.43e-05 | 0.269 | `ACTGAAA` |
| AVTGAAA | chr15 | − | 84035796 | 84035802 | 5.43e-05 | 0.269 | `ACTGAAA` |
| AVTGAAA | chr16 | − | 8963665 | 8963671 | 5.43e-05 | 0.269 | `ACTGAAA` |
| AVTGAAA | chr16 | − | 11679024 | 11679030 | 5.43e-05 | 0.269 | `ACTGAAA` |
| AVTGAAA | chr16 | − | 11690615 | 11690621 | 5.43e-05 | 0.269 | `ACTGAAA` |
| AVTGAAA | chr16 | + | 11735478 | 11735484 | 5.43e-05 | 0.269 | `ACTGAAA` |
| AVTGAAA | chr16 | + | 11735536 | 11735542 | 5.43e-05 | 0.269 | `ACTGAAA` |
| AVTGAAA | chr16 | + | 11775508 | 11775514 | 5.43e-05 | 0.269 | `ACTGAAA` |
| AVTGAAA | chr16 | + | 11775581 | 11775587 | 5.43e-05 | 0.269 | `ACTGAAA` |
| AVTGAAA | chr16 | − | 11775440 | 11775446 | 5.43e-05 | 0.269 | `ACTGAAA` |
| AVTGAAA | chr16 | − | 11967754 | 11967760 | 5.43e-05 | 0.269 | `ACTGAAA` |
| AVTGAAA | chr16 | + | 20793434 | 20793440 | 5.43e-05 | 0.269 | `ACTGAAA` |
| AVTGAAA | chr16 | − | 23745434 | 23745440 | 5.43e-05 | 0.269 | `ACTGAAA` |
| AVTGAAA | chr16 | + | 27151133 | 27151139 | 5.43e-05 | 0.269 | `ACTGAAA` |
| AVTGAAA | chr16 | + | 29735009 | 29735015 | 5.43e-05 | 0.269 | `ACTGAAA` |
| AVTGAAA | chr16 | − | 31099116 | 31099122 | 5.43e-05 | 0.269 | `ACTGAAA` |
| AVTGAAA | chr16 | − | 68938069 | 68938075 | 5.43e-05 | 0.269 | `ACTGAAA` |
| AVTGAAA | chr16 | + | 70408304 | 70408310 | 5.43e-05 | 0.269 | `ACTGAAA` |
| AVTGAAA | chr16 | + | 86365897 | 86365903 | 5.43e-05 | 0.269 | `ACTGAAA` |
| AVTGAAA | chr16 | − | 87562204 | 87562210 | 5.43e-05 | 0.269 | `ACTGAAA` |
| AVTGAAA | chr16 | + | 88158998 | 88159004 | 5.43e-05 | 0.269 | `ACTGAAA` |
| AVTGAAA | chr17 | + | 1567425 | 1567431 | 5.43e-05 | 0.269 | `ACTGAAA` |
| AVTGAAA | chr17 | + | 1920623 | 1920629 | 5.43e-05 | 0.269 | `ACTGAAA` |
| AVTGAAA | chr17 | − | 23408921 | 23408927 | 5.43e-05 | 0.269 | `ACTGAAA` |
| AVTGAAA | chr17 | − | 23408955 | 23408961 | 5.43e-05 | 0.269 | `ACTGAAA` |
| AVTGAAA | chr17 | − | 23409197 | 23409203 | 5.43e-05 | 0.269 | `ACTGAAA` |
| AVTGAAA | chr17 | − | 35167347 | 35167353 | 5.43e-05 | 0.269 | `ACTGAAA` |
| AVTGAAA | chr17 | − | 35167524 | 35167530 | 5.43e-05 | 0.269 | `ACTGAAA` |
| AVTGAAA | chr17 | + | 35274049 | 35274055 | 5.43e-05 | 0.269 | `ACTGAAA` |
| AVTGAAA | chr17 | − | 37099453 | 37099459 | 5.43e-05 | 0.269 | `ACTGAAA` |
| AVTGAAA | chr17 | − | 52790058 | 52790064 | 5.43e-05 | 0.269 | `ACTGAAA` |
| AVTGAAA | chr17 | − | 55273995 | 55274001 | 5.43e-05 | 0.269 | `ACTGAAA` |
| AVTGAAA | chr17 | + | 55275775 | 55275781 | 5.43e-05 | 0.269 | `ACTGAAA` |
| AVTGAAA | chr17 | − | 55275567 | 55275573 | 5.43e-05 | 0.269 | `ACTGAAA` |
| AVTGAAA | chr17 | − | 60412596 | 60412602 | 5.43e-05 | 0.269 | `ACTGAAA` |
| AVTGAAA | chr17 | − | 63077483 | 63077489 | 5.43e-05 | 0.269 | `ACTGAAA` |
| AVTGAAA | chr17 | + | 63745800 | 63745806 | 5.43e-05 | 0.269 | `ACTGAAA` |
| AVTGAAA | chr17 | + | 72000884 | 72000890 | 5.43e-05 | 0.269 | `ACTGAAA` |
| AVTGAAA | chr17 | − | 72615134 | 72615140 | 5.43e-05 | 0.269 | `ACTGAAA` |
| AVTGAAA | chr17 | + | 78000856 | 78000862 | 5.43e-05 | 0.269 | `ACTGAAA` |
| AVTGAAA | chr18 | + | 667310 | 667316 | 5.43e-05 | 0.269 | `ACTGAAA` |
| AVTGAAA | chr18 | − | 3576830 | 3576836 | 5.43e-05 | 0.269 | `ACTGAAA` |
| AVTGAAA | chr18 | − | 7943336 | 7943342 | 5.43e-05 | 0.269 | `ACTGAAA` |
| AVTGAAA | chr18 | − | 7943486 | 7943492 | 5.43e-05 | 0.269 | `ACTGAAA` |
| AVTGAAA | chr18 | + | 17966485 | 17966491 | 5.43e-05 | 0.269 | `ACTGAAA` |
| AVTGAAA | chr18 | + | 19994021 | 19994027 | 5.43e-05 | 0.269 | `ACTGAAA` |
| AVTGAAA | chr18 | − | 54888788 | 54888794 | 5.43e-05 | 0.269 | `ACTGAAA` |
| AVTGAAA | chr18 | − | 58324636 | 58324642 | 5.43e-05 | 0.269 | `ACTGAAA` |
| AVTGAAA | chr18 | − | 58324672 | 58324678 | 5.43e-05 | 0.269 | `ACTGAAA` |
| AVTGAAA | chr18 | + | 58975973 | 58975979 | 5.43e-05 | 0.269 | `ACTGAAA` |
| AVTGAAA | chr18 | + | 58976402 | 58976408 | 5.43e-05 | 0.269 | `ACTGAAA` |
| AVTGAAA | chr18 | + | 58977268 | 58977274 | 5.43e-05 | 0.269 | `ACTGAAA` |
| AVTGAAA | chr18 | + | 58999529 | 58999535 | 5.43e-05 | 0.269 | `ACTGAAA` |
| AVTGAAA | chr18 | − | 59755418 | 59755424 | 5.43e-05 | 0.269 | `ACTGAAA` |
| AVTGAAA | chr18 | − | 59755599 | 59755605 | 5.43e-05 | 0.269 | `ACTGAAA` |
| AVTGAAA | chr18 | + | 65767319 | 65767325 | 5.43e-05 | 0.269 | `ACTGAAA` |
| AVTGAAA | chr18 | + | 65767353 | 65767359 | 5.43e-05 | 0.269 | `ACTGAAA` |
| AVTGAAA | chr18 | − | 75297026 | 75297032 | 5.43e-05 | 0.269 | `ACTGAAA` |
| AVTGAAA | chr19 | + | 1599763 | 1599769 | 5.43e-05 | 0.269 | `ACTGAAA` |
| AVTGAAA | chr19 | − | 1599690 | 1599696 | 5.43e-05 | 0.269 | `ACTGAAA` |
| AVTGAAA | chr19 | − | 2561877 | 2561883 | 5.43e-05 | 0.269 | `ACTGAAA` |
| AVTGAAA | chr19 | − | 2561931 | 2561937 | 5.43e-05 | 0.269 | `ACTGAAA` |
| AVTGAAA | chr19 | + | 2649721 | 2649727 | 5.43e-05 | 0.269 | `ACTGAAA` |
| AVTGAAA | chr19 | − | 10566122 | 10566128 | 5.43e-05 | 0.269 | `ACTGAAA` |
| AVTGAAA | chr19 | − | 17097297 | 17097303 | 5.43e-05 | 0.269 | `ACTGAAA` |
| AVTGAAA | chr19 | − | 17097444 | 17097450 | 5.43e-05 | 0.269 | `ACTGAAA` |
| AVTGAAA | chr19 | + | 38463354 | 38463360 | 5.43e-05 | 0.269 | `ACTGAAA` |
| AVTGAAA | chr19 | + | 57182114 | 57182120 | 5.43e-05 | 0.269 | `ACTGAAA` |
| AVTGAAA | chr20 | + | 17785241 | 17785247 | 5.43e-05 | 0.269 | `ACTGAAA` |
| AVTGAAA | chr20 | + | 42708450 | 42708456 | 5.43e-05 | 0.269 | `ACTGAAA` |
| AVTGAAA | chr20 | − | 61112390 | 61112396 | 5.43e-05 | 0.269 | `ACTGAAA` |
| AVTGAAA | chr21 | − | 26029150 | 26029156 | 5.43e-05 | 0.269 | `ACTGAAA` |
| AVTGAAA | chr21 | − | 34326523 | 34326529 | 5.43e-05 | 0.269 | `ACTGAAA` |
| AVTGAAA | chr22 | − | 27525132 | 27525138 | 5.43e-05 | 0.269 | `ACTGAAA` |
| AVTGAAA | chr22 | − | 27518211 | 27518217 | 5.43e-05 | 0.269 | `ACTGAAA` |
| AVTGAAA | chr22 | + | 35255398 | 35255404 | 5.43e-05 | 0.269 | `ACTGAAA` |
| AVTGAAA | chr22 | − | 39141032 | 39141038 | 5.43e-05 | 0.269 | `ACTGAAA` |
| AVTGAAA | chr22 | − | 49089851 | 49089857 | 5.43e-05 | 0.269 | `ACTGAAA` |

---

**DEBUGGING INFORMATION**


---

Command line:

```
/ebi/sw/MEME/VM-cluster410/meme-versions/4.10.0/bin/fimo --parse-genomic-coord --verbosity 1 --oc fimo_out_3 --bgfile ./background --motif AVTGAAA dreme_out/dreme.xml ./Supplementary_Table_1.500bp.fa
```

Settings:

```
|  |  |  |
| --- | --- | --- |
| output directory = fimo_out_3 | MEME file name = dreme_out/dreme.xml | sequence file name = ./Supplementary_Table_1.500bp.fa |
| background file name = ./background | allow clobber = true | compute q-values = true |
| parse genomic coord. = true | text only = false | scan both strands = true |
| max sequence length = 250000000 | output threshold = 0.0001 | threshold type = p-value |
| max stored scores = 100000 | pseudocount = 0.1 | verbosity = 1 |
| selected motif = AVTGAAA |  |  |
```

This information can be useful in the event you wish to report a
problem with the FIMO software.

---

**Go to top**
