# Supplementary material for: The MEME Suite
Source: Nucleic Acids Res. 2015 May 7;43(Web Server issue):W39–49. doi: 10.1093/nar/gkv416 (PMC4489269; doi:10.1093/nar/gkv416)
Supplement: SUPPLEMENTARY DATA [file supp_gkv416_nar-00283-web-b-2015-File005.zip › case4/meme-chip/fimo_out_5/fimo.html]

FIMO Results


---

|  |  |  |
| --- | --- | --- |
| **Database and Motifs** | **High-scoring Motif Occurrences** | **Debugging Information** |

  
  

---

**FIMO - Motif search tool**


---

FIMO version 4.10.0,
(Release date: Wed May 21 10:35:36 2014 +1000)

For further information on how to interpret these results
or to get a copy of the FIMO software please access
http://meme.nbcr.net

If you use FIMO in your research, please cite the following paper:  
Charles E. Grant, Timothy L. Bailey, and William Stafford Noble,
"FIMO: Scanning for occurrences of a given motif",
*Bioinformatics*, **27**(7):1017-1018, 2011.
[full text]

---

**DATABASE AND MOTIFS**


---

DATABASE
./Supplementary\_Table\_1.500bp.fa  
Database contains
2776
sequences,
1388000
residues

MOTIFS
dreme\_out/dreme.xml
(nucleotide)

| MOTIF | WIDTH | BEST POSSIBLE MATCH |
| --- | --- | --- |
| GGAARY | 6 | GGAAGT |
| AVTGAAA | 7 | ACTGAAA |
| RCAGCTGY | 8 | GCAGCTGC |
| AKAAAH | 6 | AGAAAA |
| RAGKTCA | 7 | GAGGTCA |
| CMCAGM | 6 | CCCAGC |
| CCCCRCCC | 8 | CCCCGCCC |
| AAATR | 5 | AAATG |
| GAAASCA | 7 | GAAAGCA |
| CCGSCTCC | 8 | CCGCCTCC |
| CCWCCTGC | 8 | CCACCTGC |

Random model letter frequencies
(from ./background):
  
A 0.241 C 0.259 G 0.259 T 0.241

---

**SECTION I: HIGH-SCORING MOTIF OCCURRENCES**


---

- There were
  921
  motif occurrences with a
  p-value less than
  0.0001.
- The p-value of a motif occurrence is defined as the
  probability of a random sequence of the same length as the motif
  matching that position of the sequence with as good or better a score.
- The score for the match of a position in a sequence to a motif
  is computed by summing the appropriate entries from each column of
  the position-dependent scoring matrix that represents the motif.
- The q-value of a motif occurrence is defined as the
  false discovery rate if the occurrence is accepted as significant.
- The table is sorted by increasing p-value.

| Motif | Sequence Name | Strand | Start | End | p-value | q-value | Matched Sequence |
| --- | --- | --- | --- | --- | --- | --- | --- |
| RCAGCTGY | chr1 | + | 177209227 | 177209234 | 1.76e-05 | 0.148 | `GCAGCTGC` |
| RCAGCTGY | chr1 | − | 177209227 | 177209234 | 1.76e-05 | 0.148 | `GCAGCTGC` |
| RCAGCTGY | chr1 | + | 1700295 | 1700302 | 1.76e-05 | 0.148 | `GCAGCTGC` |
| RCAGCTGY | chr1 | − | 1700295 | 1700302 | 1.76e-05 | 0.148 | `GCAGCTGC` |
| RCAGCTGY | chr1 | + | 1701728 | 1701735 | 1.76e-05 | 0.148 | `GCAGCTGC` |
| RCAGCTGY | chr1 | − | 1701728 | 1701735 | 1.76e-05 | 0.148 | `GCAGCTGC` |
| RCAGCTGY | chr1 | + | 27824305 | 27824312 | 1.76e-05 | 0.148 | `GCAGCTGC` |
| RCAGCTGY | chr1 | − | 27824305 | 27824312 | 1.76e-05 | 0.148 | `GCAGCTGC` |
| RCAGCTGY | chr1 | + | 28083158 | 28083165 | 1.76e-05 | 0.148 | `GCAGCTGC` |
| RCAGCTGY | chr1 | − | 28083158 | 28083165 | 1.76e-05 | 0.148 | `GCAGCTGC` |
| RCAGCTGY | chr1 | + | 31669164 | 31669171 | 1.76e-05 | 0.148 | `GCAGCTGC` |
| RCAGCTGY | chr1 | − | 31669164 | 31669171 | 1.76e-05 | 0.148 | `GCAGCTGC` |
| RCAGCTGY | chr1 | + | 32065598 | 32065605 | 1.76e-05 | 0.148 | `GCAGCTGC` |
| RCAGCTGY | chr1 | − | 32065598 | 32065605 | 1.76e-05 | 0.148 | `GCAGCTGC` |
| RCAGCTGY | chr1 | + | 41967080 | 41967087 | 1.76e-05 | 0.148 | `GCAGCTGC` |
| RCAGCTGY | chr1 | − | 41967080 | 41967087 | 1.76e-05 | 0.148 | `GCAGCTGC` |
| RCAGCTGY | chr1 | + | 41967210 | 41967217 | 1.76e-05 | 0.148 | `GCAGCTGC` |
| RCAGCTGY | chr1 | − | 41967210 | 41967217 | 1.76e-05 | 0.148 | `GCAGCTGC` |
| RCAGCTGY | chr1 | + | 51198675 | 51198682 | 1.76e-05 | 0.148 | `GCAGCTGC` |
| RCAGCTGY | chr1 | − | 51198675 | 51198682 | 1.76e-05 | 0.148 | `GCAGCTGC` |
| RCAGCTGY | chr1 | + | 76218408 | 76218415 | 1.76e-05 | 0.148 | `GCAGCTGC` |
| RCAGCTGY | chr1 | − | 76218408 | 76218415 | 1.76e-05 | 0.148 | `GCAGCTGC` |
| RCAGCTGY | chr1 | + | 87344572 | 87344579 | 1.76e-05 | 0.148 | `GCAGCTGC` |
| RCAGCTGY | chr1 | − | 87344572 | 87344579 | 1.76e-05 | 0.148 | `GCAGCTGC` |
| RCAGCTGY | chr1 | + | 101474128 | 101474135 | 1.76e-05 | 0.148 | `GCAGCTGC` |
| RCAGCTGY | chr1 | − | 101474128 | 101474135 | 1.76e-05 | 0.148 | `GCAGCTGC` |
| RCAGCTGY | chr1 | + | 114249388 | 114249395 | 1.76e-05 | 0.148 | `GCAGCTGC` |
| RCAGCTGY | chr1 | − | 114249388 | 114249395 | 1.76e-05 | 0.148 | `GCAGCTGC` |
| RCAGCTGY | chr1 | + | 154741458 | 154741465 | 1.76e-05 | 0.148 | `GCAGCTGC` |
| RCAGCTGY | chr1 | − | 154741458 | 154741465 | 1.76e-05 | 0.148 | `GCAGCTGC` |
| RCAGCTGY | chr1 | + | 158804919 | 158804926 | 1.76e-05 | 0.148 | `GCAGCTGC` |
| RCAGCTGY | chr1 | − | 158804919 | 158804926 | 1.76e-05 | 0.148 | `GCAGCTGC` |
| RCAGCTGY | chr1 | + | 171646682 | 171646689 | 1.76e-05 | 0.148 | `GCAGCTGC` |
| RCAGCTGY | chr1 | − | 171646682 | 171646689 | 1.76e-05 | 0.148 | `GCAGCTGC` |
| RCAGCTGY | chr1 | + | 201556943 | 201556950 | 1.76e-05 | 0.148 | `GCAGCTGC` |
| RCAGCTGY | chr1 | − | 201556943 | 201556950 | 1.76e-05 | 0.148 | `GCAGCTGC` |
| RCAGCTGY | chr2 | + | 26578476 | 26578483 | 1.76e-05 | 0.148 | `GCAGCTGC` |
| RCAGCTGY | chr2 | − | 26578476 | 26578483 | 1.76e-05 | 0.148 | `GCAGCTGC` |
| RCAGCTGY | chr2 | + | 30413761 | 30413768 | 1.76e-05 | 0.148 | `GCAGCTGC` |
| RCAGCTGY | chr2 | − | 30413761 | 30413768 | 1.76e-05 | 0.148 | `GCAGCTGC` |
| RCAGCTGY | chr2 | + | 33572893 | 33572900 | 1.76e-05 | 0.148 | `GCAGCTGC` |
| RCAGCTGY | chr2 | − | 33572893 | 33572900 | 1.76e-05 | 0.148 | `GCAGCTGC` |
| RCAGCTGY | chr2 | + | 74553285 | 74553292 | 1.76e-05 | 0.148 | `GCAGCTGC` |
| RCAGCTGY | chr2 | − | 74553285 | 74553292 | 1.76e-05 | 0.148 | `GCAGCTGC` |
| RCAGCTGY | chr2 | + | 85408730 | 85408737 | 1.76e-05 | 0.148 | `GCAGCTGC` |
| RCAGCTGY | chr2 | − | 85408730 | 85408737 | 1.76e-05 | 0.148 | `GCAGCTGC` |
| RCAGCTGY | chr2 | + | 98443909 | 98443916 | 1.76e-05 | 0.148 | `GCAGCTGC` |
| RCAGCTGY | chr2 | − | 98443909 | 98443916 | 1.76e-05 | 0.148 | `GCAGCTGC` |
| RCAGCTGY | chr2 | + | 178125638 | 178125645 | 1.76e-05 | 0.148 | `GCAGCTGC` |
| RCAGCTGY | chr2 | − | 178125638 | 178125645 | 1.76e-05 | 0.148 | `GCAGCTGC` |
| RCAGCTGY | chr3 | + | 13100696 | 13100703 | 1.76e-05 | 0.148 | `GCAGCTGC` |
| RCAGCTGY | chr3 | − | 13100696 | 13100703 | 1.76e-05 | 0.148 | `GCAGCTGC` |
| RCAGCTGY | chr3 | + | 13348863 | 13348870 | 1.76e-05 | 0.148 | `GCAGCTGC` |
| RCAGCTGY | chr3 | − | 13348863 | 13348870 | 1.76e-05 | 0.148 | `GCAGCTGC` |
| RCAGCTGY | chr3 | + | 39430997 | 39431004 | 1.76e-05 | 0.148 | `GCAGCTGC` |
| RCAGCTGY | chr3 | − | 39430997 | 39431004 | 1.76e-05 | 0.148 | `GCAGCTGC` |
| RCAGCTGY | chr3 | + | 49371105 | 49371112 | 1.76e-05 | 0.148 | `GCAGCTGC` |
| RCAGCTGY | chr3 | − | 49371105 | 49371112 | 1.76e-05 | 0.148 | `GCAGCTGC` |
| RCAGCTGY | chr3 | + | 109112534 | 109112541 | 1.76e-05 | 0.148 | `GCAGCTGC` |
| RCAGCTGY | chr3 | − | 109112534 | 109112541 | 1.76e-05 | 0.148 | `GCAGCTGC` |
| RCAGCTGY | chr3 | + | 126342578 | 126342585 | 1.76e-05 | 0.148 | `GCAGCTGC` |
| RCAGCTGY | chr3 | − | 126342578 | 126342585 | 1.76e-05 | 0.148 | `GCAGCTGC` |
| RCAGCTGY | chr3 | + | 179722864 | 179722871 | 1.76e-05 | 0.148 | `GCAGCTGC` |
| RCAGCTGY | chr3 | − | 179722864 | 179722871 | 1.76e-05 | 0.148 | `GCAGCTGC` |
| RCAGCTGY | chr3 | + | 185085251 | 185085258 | 1.76e-05 | 0.148 | `GCAGCTGC` |
| RCAGCTGY | chr3 | − | 185085251 | 185085258 | 1.76e-05 | 0.148 | `GCAGCTGC` |
| RCAGCTGY | chr4 | + | 77339924 | 77339931 | 1.76e-05 | 0.148 | `GCAGCTGC` |
| RCAGCTGY | chr4 | − | 77339924 | 77339931 | 1.76e-05 | 0.148 | `GCAGCTGC` |
| RCAGCTGY | chr4 | + | 154629932 | 154629939 | 1.76e-05 | 0.148 | `GCAGCTGC` |
| RCAGCTGY | chr4 | − | 154629932 | 154629939 | 1.76e-05 | 0.148 | `GCAGCTGC` |
| RCAGCTGY | chr5 | + | 96297450 | 96297457 | 1.76e-05 | 0.148 | `GCAGCTGC` |
| RCAGCTGY | chr5 | − | 96297450 | 96297457 | 1.76e-05 | 0.148 | `GCAGCTGC` |
| RCAGCTGY | chr5 | + | 107746568 | 107746575 | 1.76e-05 | 0.148 | `GCAGCTGC` |
| RCAGCTGY | chr5 | − | 107746568 | 107746575 | 1.76e-05 | 0.148 | `GCAGCTGC` |
| RCAGCTGY | chr5 | + | 110616873 | 110616880 | 1.76e-05 | 0.148 | `GCAGCTGC` |
| RCAGCTGY | chr5 | − | 110616873 | 110616880 | 1.76e-05 | 0.148 | `GCAGCTGC` |
| RCAGCTGY | chr5 | + | 131830244 | 131830251 | 1.76e-05 | 0.148 | `GCAGCTGC` |
| RCAGCTGY | chr5 | − | 131830244 | 131830251 | 1.76e-05 | 0.148 | `GCAGCTGC` |
| RCAGCTGY | chr5 | + | 134268125 | 134268132 | 1.76e-05 | 0.148 | `GCAGCTGC` |
| RCAGCTGY | chr5 | − | 134268125 | 134268132 | 1.76e-05 | 0.148 | `GCAGCTGC` |
| RCAGCTGY | chr5 | + | 139907411 | 139907418 | 1.76e-05 | 0.148 | `GCAGCTGC` |
| RCAGCTGY | chr5 | − | 139907411 | 139907418 | 1.76e-05 | 0.148 | `GCAGCTGC` |
| RCAGCTGY | chr6 | + | 21696449 | 21696456 | 1.76e-05 | 0.148 | `GCAGCTGC` |
| RCAGCTGY | chr6 | − | 21696449 | 21696456 | 1.76e-05 | 0.148 | `GCAGCTGC` |
| RCAGCTGY | chr6 | + | 31343901 | 31343908 | 1.76e-05 | 0.148 | `GCAGCTGC` |
| RCAGCTGY | chr6 | − | 31343901 | 31343908 | 1.76e-05 | 0.148 | `GCAGCTGC` |
| RCAGCTGY | chr6 | + | 33353010 | 33353017 | 1.76e-05 | 0.148 | `GCAGCTGC` |
| RCAGCTGY | chr6 | − | 33353010 | 33353017 | 1.76e-05 | 0.148 | `GCAGCTGC` |
| RCAGCTGY | chr6 | + | 42822095 | 42822102 | 1.76e-05 | 0.148 | `GCAGCTGC` |
| RCAGCTGY | chr6 | − | 42822095 | 42822102 | 1.76e-05 | 0.148 | `GCAGCTGC` |
| RCAGCTGY | chr7 | + | 2900986 | 2900993 | 1.76e-05 | 0.148 | `GCAGCTGC` |
| RCAGCTGY | chr7 | − | 2900986 | 2900993 | 1.76e-05 | 0.148 | `GCAGCTGC` |
| RCAGCTGY | chr7 | + | 25974939 | 25974946 | 1.76e-05 | 0.148 | `GCAGCTGC` |
| RCAGCTGY | chr7 | − | 25974939 | 25974946 | 1.76e-05 | 0.148 | `GCAGCTGC` |
| RCAGCTGY | chr7 | + | 35731679 | 35731686 | 1.76e-05 | 0.148 | `GCAGCTGC` |
| RCAGCTGY | chr7 | − | 35731679 | 35731686 | 1.76e-05 | 0.148 | `GCAGCTGC` |
| RCAGCTGY | chr7 | + | 55569596 | 55569603 | 1.76e-05 | 0.148 | `GCAGCTGC` |
| RCAGCTGY | chr7 | − | 55569596 | 55569603 | 1.76e-05 | 0.148 | `GCAGCTGC` |
| RCAGCTGY | chr7 | + | 86686967 | 86686974 | 1.76e-05 | 0.148 | `GCAGCTGC` |
| RCAGCTGY | chr7 | − | 86686967 | 86686974 | 1.76e-05 | 0.148 | `GCAGCTGC` |
| RCAGCTGY | chr7 | + | 97599054 | 97599061 | 1.76e-05 | 0.148 | `GCAGCTGC` |
| RCAGCTGY | chr7 | − | 97599054 | 97599061 | 1.76e-05 | 0.148 | `GCAGCTGC` |
| RCAGCTGY | chr7 | + | 129903993 | 129904000 | 1.76e-05 | 0.148 | `GCAGCTGC` |
| RCAGCTGY | chr7 | − | 129903993 | 129904000 | 1.76e-05 | 0.148 | `GCAGCTGC` |
| RCAGCTGY | chr7 | + | 134506127 | 134506134 | 1.76e-05 | 0.148 | `GCAGCTGC` |
| RCAGCTGY | chr7 | − | 134506127 | 134506134 | 1.76e-05 | 0.148 | `GCAGCTGC` |
| RCAGCTGY | chr8 | + | 10860607 | 10860614 | 1.76e-05 | 0.148 | `GCAGCTGC` |
| RCAGCTGY | chr8 | − | 10860607 | 10860614 | 1.76e-05 | 0.148 | `GCAGCTGC` |
| RCAGCTGY | chr8 | + | 96312866 | 96312873 | 1.76e-05 | 0.148 | `GCAGCTGC` |
| RCAGCTGY | chr8 | − | 96312866 | 96312873 | 1.76e-05 | 0.148 | `GCAGCTGC` |
| RCAGCTGY | chr8 | + | 125003603 | 125003610 | 1.76e-05 | 0.148 | `GCAGCTGC` |
| RCAGCTGY | chr8 | − | 125003603 | 125003610 | 1.76e-05 | 0.148 | `GCAGCTGC` |
| RCAGCTGY | chr8 | + | 134563925 | 134563932 | 1.76e-05 | 0.148 | `GCAGCTGC` |
| RCAGCTGY | chr8 | − | 134563925 | 134563932 | 1.76e-05 | 0.148 | `GCAGCTGC` |
| RCAGCTGY | chr9 | + | 115384211 | 115384218 | 1.76e-05 | 0.148 | `GCAGCTGC` |
| RCAGCTGY | chr9 | − | 115384211 | 115384218 | 1.76e-05 | 0.148 | `GCAGCTGC` |
| RCAGCTGY | chr9 | + | 125141767 | 125141774 | 1.76e-05 | 0.148 | `GCAGCTGC` |
| RCAGCTGY | chr9 | − | 125141767 | 125141774 | 1.76e-05 | 0.148 | `GCAGCTGC` |
| RCAGCTGY | chr9 | + | 129581660 | 129581667 | 1.76e-05 | 0.148 | `GCAGCTGC` |
| RCAGCTGY | chr9 | − | 129581660 | 129581667 | 1.76e-05 | 0.148 | `GCAGCTGC` |
| RCAGCTGY | chrX | + | 48679553 | 48679560 | 1.76e-05 | 0.148 | `GCAGCTGC` |
| RCAGCTGY | chrX | − | 48679553 | 48679560 | 1.76e-05 | 0.148 | `GCAGCTGC` |
| RCAGCTGY | chr10 | + | 7010136 | 7010143 | 1.76e-05 | 0.148 | `GCAGCTGC` |
| RCAGCTGY | chr10 | − | 7010136 | 7010143 | 1.76e-05 | 0.148 | `GCAGCTGC` |
| RCAGCTGY | chr10 | + | 7554166 | 7554173 | 1.76e-05 | 0.148 | `GCAGCTGC` |
| RCAGCTGY | chr10 | − | 7554166 | 7554173 | 1.76e-05 | 0.148 | `GCAGCTGC` |
| RCAGCTGY | chr10 | + | 11324486 | 11324493 | 1.76e-05 | 0.148 | `GCAGCTGC` |
| RCAGCTGY | chr10 | − | 11324486 | 11324493 | 1.76e-05 | 0.148 | `GCAGCTGC` |
| RCAGCTGY | chr10 | + | 11327635 | 11327642 | 1.76e-05 | 0.148 | `GCAGCTGC` |
| RCAGCTGY | chr10 | − | 11327635 | 11327642 | 1.76e-05 | 0.148 | `GCAGCTGC` |
| RCAGCTGY | chr10 | + | 27571276 | 27571283 | 1.76e-05 | 0.148 | `GCAGCTGC` |
| RCAGCTGY | chr10 | − | 27571276 | 27571283 | 1.76e-05 | 0.148 | `GCAGCTGC` |
| RCAGCTGY | chr10 | + | 35791569 | 35791576 | 1.76e-05 | 0.148 | `GCAGCTGC` |
| RCAGCTGY | chr10 | − | 35791569 | 35791576 | 1.76e-05 | 0.148 | `GCAGCTGC` |
| RCAGCTGY | chr10 | + | 73703432 | 73703439 | 1.76e-05 | 0.148 | `GCAGCTGC` |
| RCAGCTGY | chr10 | − | 73703432 | 73703439 | 1.76e-05 | 0.148 | `GCAGCTGC` |
| RCAGCTGY | chr10 | + | 73705569 | 73705576 | 1.76e-05 | 0.148 | `GCAGCTGC` |
| RCAGCTGY | chr10 | − | 73705569 | 73705576 | 1.76e-05 | 0.148 | `GCAGCTGC` |
| RCAGCTGY | chr10 | + | 82002374 | 82002381 | 1.76e-05 | 0.148 | `GCAGCTGC` |
| RCAGCTGY | chr10 | − | 82002374 | 82002381 | 1.76e-05 | 0.148 | `GCAGCTGC` |
| RCAGCTGY | chr10 | + | 95452183 | 95452190 | 1.76e-05 | 0.148 | `GCAGCTGC` |
| RCAGCTGY | chr10 | − | 95452183 | 95452190 | 1.76e-05 | 0.148 | `GCAGCTGC` |
| RCAGCTGY | chr10 | + | 103609093 | 103609100 | 1.76e-05 | 0.148 | `GCAGCTGC` |
| RCAGCTGY | chr10 | − | 103609093 | 103609100 | 1.76e-05 | 0.148 | `GCAGCTGC` |
| RCAGCTGY | chr10 | + | 104144439 | 104144446 | 1.76e-05 | 0.148 | `GCAGCTGC` |
| RCAGCTGY | chr10 | − | 104144439 | 104144446 | 1.76e-05 | 0.148 | `GCAGCTGC` |
| RCAGCTGY | chr10 | + | 121239223 | 121239230 | 1.76e-05 | 0.148 | `GCAGCTGC` |
| RCAGCTGY | chr10 | − | 121239223 | 121239230 | 1.76e-05 | 0.148 | `GCAGCTGC` |
| RCAGCTGY | chr11 | + | 606214 | 606221 | 1.76e-05 | 0.148 | `GCAGCTGC` |
| RCAGCTGY | chr11 | − | 606214 | 606221 | 1.76e-05 | 0.148 | `GCAGCTGC` |
| RCAGCTGY | chr11 | + | 10786844 | 10786851 | 1.76e-05 | 0.148 | `GCAGCTGC` |
| RCAGCTGY | chr11 | − | 10786844 | 10786851 | 1.76e-05 | 0.148 | `GCAGCTGC` |
| RCAGCTGY | chr11 | + | 10786856 | 10786863 | 1.76e-05 | 0.148 | `GCAGCTGC` |
| RCAGCTGY | chr11 | − | 10786856 | 10786863 | 1.76e-05 | 0.148 | `GCAGCTGC` |
| RCAGCTGY | chr11 | + | 64620308 | 64620315 | 1.76e-05 | 0.148 | `GCAGCTGC` |
| RCAGCTGY | chr11 | − | 64620308 | 64620315 | 1.76e-05 | 0.148 | `GCAGCTGC` |
| RCAGCTGY | chr11 | + | 65076201 | 65076208 | 1.76e-05 | 0.148 | `GCAGCTGC` |
| RCAGCTGY | chr11 | − | 65076201 | 65076208 | 1.76e-05 | 0.148 | `GCAGCTGC` |
| RCAGCTGY | chr11 | + | 68821569 | 68821576 | 1.76e-05 | 0.148 | `GCAGCTGC` |
| RCAGCTGY | chr11 | − | 68821569 | 68821576 | 1.76e-05 | 0.148 | `GCAGCTGC` |
| RCAGCTGY | chr11 | + | 69209838 | 69209845 | 1.76e-05 | 0.148 | `GCAGCTGC` |
| RCAGCTGY | chr11 | − | 69209838 | 69209845 | 1.76e-05 | 0.148 | `GCAGCTGC` |
| RCAGCTGY | chr12 | + | 1533891 | 1533898 | 1.76e-05 | 0.148 | `GCAGCTGC` |
| RCAGCTGY | chr12 | − | 1533891 | 1533898 | 1.76e-05 | 0.148 | `GCAGCTGC` |
| RCAGCTGY | chr12 | + | 6431458 | 6431465 | 1.76e-05 | 0.148 | `GCAGCTGC` |
| RCAGCTGY | chr12 | − | 6431458 | 6431465 | 1.76e-05 | 0.148 | `GCAGCTGC` |
| RCAGCTGY | chr12 | + | 107486573 | 107486580 | 1.76e-05 | 0.148 | `GCAGCTGC` |
| RCAGCTGY | chr12 | − | 107486573 | 107486580 | 1.76e-05 | 0.148 | `GCAGCTGC` |
| RCAGCTGY | chr12 | + | 112095598 | 112095605 | 1.76e-05 | 0.148 | `GCAGCTGC` |
| RCAGCTGY | chr12 | − | 112095598 | 112095605 | 1.76e-05 | 0.148 | `GCAGCTGC` |
| RCAGCTGY | chr12 | + | 120913621 | 120913628 | 1.76e-05 | 0.148 | `GCAGCTGC` |
| RCAGCTGY | chr12 | − | 120913621 | 120913628 | 1.76e-05 | 0.148 | `GCAGCTGC` |
| RCAGCTGY | chr13 | + | 45862267 | 45862274 | 1.76e-05 | 0.148 | `GCAGCTGC` |
| RCAGCTGY | chr13 | − | 45862267 | 45862274 | 1.76e-05 | 0.148 | `GCAGCTGC` |
| RCAGCTGY | chr14 | + | 91046425 | 91046432 | 1.76e-05 | 0.148 | `GCAGCTGC` |
| RCAGCTGY | chr14 | − | 91046425 | 91046432 | 1.76e-05 | 0.148 | `GCAGCTGC` |
| RCAGCTGY | chr14 | + | 94801995 | 94802002 | 1.76e-05 | 0.148 | `GCAGCTGC` |
| RCAGCTGY | chr14 | − | 94801995 | 94802002 | 1.76e-05 | 0.148 | `GCAGCTGC` |
| RCAGCTGY | chr14 | + | 95198925 | 95198932 | 1.76e-05 | 0.148 | `GCAGCTGC` |
| RCAGCTGY | chr14 | − | 95198925 | 95198932 | 1.76e-05 | 0.148 | `GCAGCTGC` |
| RCAGCTGY | chr14 | + | 104583230 | 104583237 | 1.76e-05 | 0.148 | `GCAGCTGC` |
| RCAGCTGY | chr14 | − | 104583230 | 104583237 | 1.76e-05 | 0.148 | `GCAGCTGC` |
| RCAGCTGY | chr14 | + | 105836165 | 105836172 | 1.76e-05 | 0.148 | `GCAGCTGC` |
| RCAGCTGY | chr14 | − | 105836165 | 105836172 | 1.76e-05 | 0.148 | `GCAGCTGC` |
| RCAGCTGY | chr15 | + | 19248879 | 19248886 | 1.76e-05 | 0.148 | `GCAGCTGC` |
| RCAGCTGY | chr15 | − | 19248879 | 19248886 | 1.76e-05 | 0.148 | `GCAGCTGC` |
| RCAGCTGY | chr15 | + | 19249038 | 19249045 | 1.76e-05 | 0.148 | `GCAGCTGC` |
| RCAGCTGY | chr15 | − | 19249038 | 19249045 | 1.76e-05 | 0.148 | `GCAGCTGC` |
| RCAGCTGY | chr15 | + | 29425839 | 29425846 | 1.76e-05 | 0.148 | `GCAGCTGC` |
| RCAGCTGY | chr15 | − | 29425839 | 29425846 | 1.76e-05 | 0.148 | `GCAGCTGC` |
| RCAGCTGY | chr15 | + | 91184201 | 91184208 | 1.76e-05 | 0.148 | `GCAGCTGC` |
| RCAGCTGY | chr15 | − | 91184201 | 91184208 | 1.76e-05 | 0.148 | `GCAGCTGC` |
| RCAGCTGY | chr16 | + | 23254836 | 23254843 | 1.76e-05 | 0.148 | `GCAGCTGC` |
| RCAGCTGY | chr16 | − | 23254836 | 23254843 | 1.76e-05 | 0.148 | `GCAGCTGC` |
| RCAGCTGY | chr16 | + | 51690600 | 51690607 | 1.76e-05 | 0.148 | `GCAGCTGC` |
| RCAGCTGY | chr16 | − | 51690600 | 51690607 | 1.76e-05 | 0.148 | `GCAGCTGC` |
| RCAGCTGY | chr17 | + | 1891861 | 1891868 | 1.76e-05 | 0.148 | `GCAGCTGC` |
| RCAGCTGY | chr17 | − | 1891861 | 1891868 | 1.76e-05 | 0.148 | `GCAGCTGC` |
| RCAGCTGY | chr17 | + | 35963953 | 35963960 | 1.76e-05 | 0.148 | `GCAGCTGC` |
| RCAGCTGY | chr17 | − | 35963953 | 35963960 | 1.76e-05 | 0.148 | `GCAGCTGC` |
| RCAGCTGY | chr17 | + | 52789251 | 52789258 | 1.76e-05 | 0.148 | `GCAGCTGC` |
| RCAGCTGY | chr17 | − | 52789251 | 52789258 | 1.76e-05 | 0.148 | `GCAGCTGC` |
| RCAGCTGY | chr17 | + | 73620702 | 73620709 | 1.76e-05 | 0.148 | `GCAGCTGC` |
| RCAGCTGY | chr17 | − | 73620702 | 73620709 | 1.76e-05 | 0.148 | `GCAGCTGC` |
| RCAGCTGY | chr18 | + | 3584113 | 3584120 | 1.76e-05 | 0.148 | `GCAGCTGC` |
| RCAGCTGY | chr18 | − | 3584113 | 3584120 | 1.76e-05 | 0.148 | `GCAGCTGC` |
| RCAGCTGY | chr18 | + | 19735217 | 19735224 | 1.76e-05 | 0.148 | `GCAGCTGC` |
| RCAGCTGY | chr18 | − | 19735217 | 19735224 | 1.76e-05 | 0.148 | `GCAGCTGC` |
| RCAGCTGY | chr18 | + | 42007949 | 42007956 | 1.76e-05 | 0.148 | `GCAGCTGC` |
| RCAGCTGY | chr18 | − | 42007949 | 42007956 | 1.76e-05 | 0.148 | `GCAGCTGC` |
| RCAGCTGY | chr18 | + | 55220832 | 55220839 | 1.76e-05 | 0.148 | `GCAGCTGC` |
| RCAGCTGY | chr18 | − | 55220832 | 55220839 | 1.76e-05 | 0.148 | `GCAGCTGC` |
| RCAGCTGY | chr18 | + | 64811259 | 64811266 | 1.76e-05 | 0.148 | `GCAGCTGC` |
| RCAGCTGY | chr18 | − | 64811259 | 64811266 | 1.76e-05 | 0.148 | `GCAGCTGC` |
| RCAGCTGY | chr19 | + | 2512957 | 2512964 | 1.76e-05 | 0.148 | `GCAGCTGC` |
| RCAGCTGY | chr19 | − | 2512957 | 2512964 | 1.76e-05 | 0.148 | `GCAGCTGC` |
| RCAGCTGY | chr19 | + | 10085338 | 10085345 | 1.76e-05 | 0.148 | `GCAGCTGC` |
| RCAGCTGY | chr19 | − | 10085338 | 10085345 | 1.76e-05 | 0.148 | `GCAGCTGC` |
| RCAGCTGY | chr19 | + | 17904543 | 17904550 | 1.76e-05 | 0.148 | `GCAGCTGC` |
| RCAGCTGY | chr19 | − | 17904543 | 17904550 | 1.76e-05 | 0.148 | `GCAGCTGC` |
| RCAGCTGY | chr19 | + | 45623896 | 45623903 | 1.76e-05 | 0.148 | `GCAGCTGC` |
| RCAGCTGY | chr19 | − | 45623896 | 45623903 | 1.76e-05 | 0.148 | `GCAGCTGC` |
| RCAGCTGY | chr19 | + | 45975055 | 45975062 | 1.76e-05 | 0.148 | `GCAGCTGC` |
| RCAGCTGY | chr19 | − | 45975055 | 45975062 | 1.76e-05 | 0.148 | `GCAGCTGC` |
| RCAGCTGY | chr19 | + | 47067262 | 47067269 | 1.76e-05 | 0.148 | `GCAGCTGC` |
| RCAGCTGY | chr19 | − | 47067262 | 47067269 | 1.76e-05 | 0.148 | `GCAGCTGC` |
| RCAGCTGY | chr19 | + | 55091000 | 55091007 | 1.76e-05 | 0.148 | `GCAGCTGC` |
| RCAGCTGY | chr19 | − | 55091000 | 55091007 | 1.76e-05 | 0.148 | `GCAGCTGC` |
| RCAGCTGY | chr19 | + | 60462411 | 60462418 | 1.76e-05 | 0.148 | `GCAGCTGC` |
| RCAGCTGY | chr19 | − | 60462411 | 60462418 | 1.76e-05 | 0.148 | `GCAGCTGC` |
| RCAGCTGY | chr20 | + | 17785079 | 17785086 | 1.76e-05 | 0.148 | `GCAGCTGC` |
| RCAGCTGY | chr20 | − | 17785079 | 17785086 | 1.76e-05 | 0.148 | `GCAGCTGC` |
| RCAGCTGY | chr20 | + | 29664383 | 29664390 | 1.76e-05 | 0.148 | `GCAGCTGC` |
| RCAGCTGY | chr20 | − | 29664383 | 29664390 | 1.76e-05 | 0.148 | `GCAGCTGC` |
| RCAGCTGY | chr20 | + | 36937711 | 36937718 | 1.76e-05 | 0.148 | `GCAGCTGC` |
| RCAGCTGY | chr20 | − | 36937711 | 36937718 | 1.76e-05 | 0.148 | `GCAGCTGC` |
| RCAGCTGY | chr20 | + | 44192721 | 44192728 | 1.76e-05 | 0.148 | `GCAGCTGC` |
| RCAGCTGY | chr20 | − | 44192721 | 44192728 | 1.76e-05 | 0.148 | `GCAGCTGC` |
| RCAGCTGY | chr20 | + | 46788711 | 46788718 | 1.76e-05 | 0.148 | `GCAGCTGC` |
| RCAGCTGY | chr20 | − | 46788711 | 46788718 | 1.76e-05 | 0.148 | `GCAGCTGC` |
| RCAGCTGY | chr20 | + | 51889718 | 51889725 | 1.76e-05 | 0.148 | `GCAGCTGC` |
| RCAGCTGY | chr20 | − | 51889718 | 51889725 | 1.76e-05 | 0.148 | `GCAGCTGC` |
| RCAGCTGY | chr21 | + | 41754313 | 41754320 | 1.76e-05 | 0.148 | `GCAGCTGC` |
| RCAGCTGY | chr21 | − | 41754313 | 41754320 | 1.76e-05 | 0.148 | `GCAGCTGC` |
| RCAGCTGY | chr22 | + | 16119321 | 16119328 | 1.76e-05 | 0.148 | `GCAGCTGC` |
| RCAGCTGY | chr22 | + | 16119327 | 16119334 | 1.76e-05 | 0.148 | `GCAGCTGC` |
| RCAGCTGY | chr22 | − | 16119321 | 16119328 | 1.76e-05 | 0.148 | `GCAGCTGC` |
| RCAGCTGY | chr22 | − | 16119327 | 16119334 | 1.76e-05 | 0.148 | `GCAGCTGC` |
| RCAGCTGY | chr22 | + | 21107247 | 21107254 | 1.76e-05 | 0.148 | `GCAGCTGC` |
| RCAGCTGY | chr22 | − | 21107247 | 21107254 | 1.76e-05 | 0.148 | `GCAGCTGC` |
| RCAGCTGY | chr22 | + | 35587174 | 35587181 | 1.76e-05 | 0.148 | `GCAGCTGC` |
| RCAGCTGY | chr22 | − | 35587174 | 35587181 | 1.76e-05 | 0.148 | `GCAGCTGC` |
| RCAGCTGY | chr22 | + | 38038085 | 38038092 | 1.76e-05 | 0.148 | `GCAGCTGC` |
| RCAGCTGY | chr22 | − | 38038085 | 38038092 | 1.76e-05 | 0.148 | `GCAGCTGC` |
| RCAGCTGY | chr22 | + | 49086524 | 49086531 | 1.76e-05 | 0.148 | `GCAGCTGC` |
| RCAGCTGY | chr22 | − | 49086524 | 49086531 | 1.76e-05 | 0.148 | `GCAGCTGC` |
| RCAGCTGY | chr22 | + | 49311586 | 49311593 | 1.76e-05 | 0.148 | `GCAGCTGC` |
| RCAGCTGY | chr22 | − | 49311586 | 49311593 | 1.76e-05 | 0.148 | `GCAGCTGC` |
| RCAGCTGY | chr1 | + | 177209236 | 177209243 | 3.4e-05 | 0.188 | `ACAGCTGC` |
| RCAGCTGY | chr1 | + | 21492839 | 21492846 | 3.4e-05 | 0.188 | `ACAGCTGC` |
| RCAGCTGY | chr1 | − | 28776552 | 28776559 | 3.4e-05 | 0.188 | `ACAGCTGC` |
| RCAGCTGY | chr1 | + | 148806923 | 148806930 | 3.4e-05 | 0.188 | `ACAGCTGC` |
| RCAGCTGY | chr1 | − | 155418451 | 155418458 | 3.4e-05 | 0.188 | `ACAGCTGC` |
| RCAGCTGY | chr1 | − | 210172869 | 210172876 | 3.4e-05 | 0.188 | `ACAGCTGC` |
| RCAGCTGY | chr1 | − | 211221958 | 211221965 | 3.4e-05 | 0.188 | `ACAGCTGC` |
| RCAGCTGY | chr1 | − | 228312489 | 228312496 | 3.4e-05 | 0.188 | `ACAGCTGC` |
| RCAGCTGY | chr2 | + | 27157639 | 27157646 | 3.4e-05 | 0.188 | `ACAGCTGC` |
| RCAGCTGY | chr2 | + | 70170303 | 70170310 | 3.4e-05 | 0.188 | `ACAGCTGC` |
| RCAGCTGY | chr2 | − | 99163833 | 99163840 | 3.4e-05 | 0.188 | `ACAGCTGC` |
| RCAGCTGY | chr2 | − | 136680727 | 136680734 | 3.4e-05 | 0.188 | `ACAGCTGC` |
| RCAGCTGY | chr2 | + | 232281198 | 232281205 | 3.4e-05 | 0.188 | `ACAGCTGC` |
| RCAGCTGY | chr2 | + | 233656354 | 233656361 | 3.4e-05 | 0.188 | `ACAGCTGC` |
| RCAGCTGY | chr3 | − | 6277384 | 6277391 | 3.4e-05 | 0.188 | `ACAGCTGC` |
| RCAGCTGY | chr3 | + | 10240475 | 10240482 | 3.4e-05 | 0.188 | `ACAGCTGC` |
| RCAGCTGY | chr3 | + | 45570351 | 45570358 | 3.4e-05 | 0.188 | `ACAGCTGC` |
| RCAGCTGY | chr3 | + | 45978649 | 45978656 | 3.4e-05 | 0.188 | `ACAGCTGC` |
| RCAGCTGY | chr3 | + | 53112053 | 53112060 | 3.4e-05 | 0.188 | `ACAGCTGC` |
| RCAGCTGY | chr3 | + | 128956860 | 128956867 | 3.4e-05 | 0.188 | `ACAGCTGC` |
| RCAGCTGY | chr3 | − | 169274294 | 169274301 | 3.4e-05 | 0.188 | `ACAGCTGC` |
| RCAGCTGY | chr3 | − | 179719905 | 179719912 | 3.4e-05 | 0.188 | `ACAGCTGC` |
| RCAGCTGY | chr3 | − | 198923535 | 198923542 | 3.4e-05 | 0.188 | `ACAGCTGC` |
| RCAGCTGY | chr4 | − | 2718045 | 2718052 | 3.4e-05 | 0.188 | `ACAGCTGC` |
| RCAGCTGY | chr4 | − | 7529690 | 7529697 | 3.4e-05 | 0.188 | `ACAGCTGC` |
| RCAGCTGY | chr4 | + | 55127438 | 55127445 | 3.4e-05 | 0.188 | `ACAGCTGC` |
| RCAGCTGY | chr4 | + | 77339116 | 77339123 | 3.4e-05 | 0.188 | `ACAGCTGC` |
| RCAGCTGY | chr4 | − | 95123453 | 95123460 | 3.4e-05 | 0.188 | `ACAGCTGC` |
| RCAGCTGY | chr5 | + | 1845946 | 1845953 | 3.4e-05 | 0.188 | `ACAGCTGC` |
| RCAGCTGY | chr5 | − | 43639467 | 43639474 | 3.4e-05 | 0.188 | `ACAGCTGC` |
| RCAGCTGY | chr5 | + | 132608037 | 132608044 | 3.4e-05 | 0.188 | `ACAGCTGC` |
| RCAGCTGY | chr5 | − | 149764039 | 149764046 | 3.4e-05 | 0.188 | `ACAGCTGC` |
| RCAGCTGY | chr6 | − | 309758 | 309765 | 3.4e-05 | 0.188 | `ACAGCTGC` |
| RCAGCTGY | chr6 | − | 310122 | 310129 | 3.4e-05 | 0.188 | `ACAGCTGC` |
| RCAGCTGY | chr6 | − | 337371 | 337378 | 3.4e-05 | 0.188 | `ACAGCTGC` |
| RCAGCTGY | chr6 | + | 22106998 | 22107005 | 3.4e-05 | 0.188 | `ACAGCTGC` |
| RCAGCTGY | chr6 | + | 31431156 | 31431163 | 3.4e-05 | 0.188 | `ACAGCTGC` |
| RCAGCTGY | chr6 | + | 90123980 | 90123987 | 3.4e-05 | 0.188 | `ACAGCTGC` |
| RCAGCTGY | chr7 | + | 7950943 | 7950950 | 3.4e-05 | 0.188 | `ACAGCTGC` |
| RCAGCTGY | chr7 | + | 28487758 | 28487765 | 3.4e-05 | 0.188 | `ACAGCTGC` |
| RCAGCTGY | chr7 | + | 47944570 | 47944577 | 3.4e-05 | 0.188 | `ACAGCTGC` |
| RCAGCTGY | chr7 | − | 92280795 | 92280802 | 3.4e-05 | 0.188 | `ACAGCTGC` |
| RCAGCTGY | chr7 | + | 97600568 | 97600575 | 3.4e-05 | 0.188 | `ACAGCTGC` |
| RCAGCTGY | chr8 | + | 8179713 | 8179720 | 3.4e-05 | 0.188 | `ACAGCTGC` |
| RCAGCTGY | chr8 | + | 72918481 | 72918488 | 3.4e-05 | 0.188 | `ACAGCTGC` |
| RCAGCTGY | chr8 | + | 103617735 | 103617742 | 3.4e-05 | 0.188 | `ACAGCTGC` |
| RCAGCTGY | chr8 | + | 104021693 | 104021700 | 3.4e-05 | 0.188 | `ACAGCTGC` |
| RCAGCTGY | chr8 | − | 125003651 | 125003658 | 3.4e-05 | 0.188 | `ACAGCTGC` |
| RCAGCTGY | chr8 | − | 126415657 | 126415664 | 3.4e-05 | 0.188 | `ACAGCTGC` |
| RCAGCTGY | chr9 | − | 37399278 | 37399285 | 3.4e-05 | 0.188 | `ACAGCTGC` |
| RCAGCTGY | chr9 | − | 114585971 | 114585978 | 3.4e-05 | 0.188 | `ACAGCTGC` |
| RCAGCTGY | chr9 | − | 127043701 | 127043708 | 3.4e-05 | 0.188 | `ACAGCTGC` |
| RCAGCTGY | chrX | + | 38667669 | 38667676 | 3.4e-05 | 0.188 | `ACAGCTGC` |
| RCAGCTGY | chrX | + | 38668085 | 38668092 | 3.4e-05 | 0.188 | `ACAGCTGC` |
| RCAGCTGY | chrX | + | 70759157 | 70759164 | 3.4e-05 | 0.188 | `ACAGCTGC` |
| RCAGCTGY | chr10 | + | 2980582 | 2980589 | 3.4e-05 | 0.188 | `ACAGCTGC` |
| RCAGCTGY | chr10 | + | 69761674 | 69761681 | 3.4e-05 | 0.188 | `ACAGCTGC` |
| RCAGCTGY | chr10 | + | 73765388 | 73765395 | 3.4e-05 | 0.188 | `ACAGCTGC` |
| RCAGCTGY | chr10 | − | 82002338 | 82002345 | 3.4e-05 | 0.188 | `ACAGCTGC` |
| RCAGCTGY | chr10 | − | 82002509 | 82002516 | 3.4e-05 | 0.188 | `ACAGCTGC` |
| RCAGCTGY | chr10 | − | 82002545 | 82002552 | 3.4e-05 | 0.188 | `ACAGCTGC` |
| RCAGCTGY | chr10 | − | 82002653 | 82002660 | 3.4e-05 | 0.188 | `ACAGCTGC` |
| RCAGCTGY | chr10 | − | 82002689 | 82002696 | 3.4e-05 | 0.188 | `ACAGCTGC` |
| RCAGCTGY | chr10 | − | 82002725 | 82002732 | 3.4e-05 | 0.188 | `ACAGCTGC` |
| RCAGCTGY | chr10 | − | 82002761 | 82002768 | 3.4e-05 | 0.188 | `ACAGCTGC` |
| RCAGCTGY | chr10 | + | 90020872 | 90020879 | 3.4e-05 | 0.188 | `ACAGCTGC` |
| RCAGCTGY | chr10 | + | 93851229 | 93851236 | 3.4e-05 | 0.188 | `ACAGCTGC` |
| RCAGCTGY | chr10 | + | 104411191 | 104411198 | 3.4e-05 | 0.188 | `ACAGCTGC` |
| RCAGCTGY | chr10 | + | 126397743 | 126397750 | 3.4e-05 | 0.188 | `ACAGCTGC` |
| RCAGCTGY | chr11 | + | 602165 | 602172 | 3.4e-05 | 0.188 | `ACAGCTGC` |
| RCAGCTGY | chr11 | + | 63750928 | 63750935 | 3.4e-05 | 0.188 | `ACAGCTGC` |
| RCAGCTGY | chr11 | + | 64375737 | 64375744 | 3.4e-05 | 0.188 | `ACAGCTGC` |
| RCAGCTGY | chr11 | − | 65027225 | 65027232 | 3.4e-05 | 0.188 | `ACAGCTGC` |
| RCAGCTGY | chr11 | + | 71823171 | 71823178 | 3.4e-05 | 0.188 | `ACAGCTGC` |
| RCAGCTGY | chr11 | − | 103274671 | 103274678 | 3.4e-05 | 0.188 | `ACAGCTGC` |
| RCAGCTGY | chr11 | − | 117387136 | 117387143 | 3.4e-05 | 0.188 | `ACAGCTGC` |
| RCAGCTGY | chr11 | − | 118068633 | 118068640 | 3.4e-05 | 0.188 | `ACAGCTGC` |
| RCAGCTGY | chr12 | + | 2335372 | 2335379 | 3.4e-05 | 0.188 | `ACAGCTGC` |
| RCAGCTGY | chr12 | − | 6938500 | 6938507 | 3.4e-05 | 0.188 | `ACAGCTGC` |
| RCAGCTGY | chr12 | − | 12054486 | 12054493 | 3.4e-05 | 0.188 | `ACAGCTGC` |
| RCAGCTGY | chr12 | − | 67522030 | 67522037 | 3.4e-05 | 0.188 | `ACAGCTGC` |
| RCAGCTGY | chr12 | + | 74640243 | 74640250 | 3.4e-05 | 0.188 | `ACAGCTGC` |
| RCAGCTGY | chr12 | − | 91321757 | 91321764 | 3.4e-05 | 0.188 | `ACAGCTGC` |
| RCAGCTGY | chr12 | + | 93480066 | 93480073 | 3.4e-05 | 0.188 | `ACAGCTGC` |
| RCAGCTGY | chr12 | + | 93480281 | 93480288 | 3.4e-05 | 0.188 | `ACAGCTGC` |
| RCAGCTGY | chr12 | + | 107551668 | 107551675 | 3.4e-05 | 0.188 | `ACAGCTGC` |
| RCAGCTGY | chr12 | − | 112130475 | 112130482 | 3.4e-05 | 0.188 | `ACAGCTGC` |
| RCAGCTGY | chr12 | − | 123961128 | 123961135 | 3.4e-05 | 0.188 | `ACAGCTGC` |
| RCAGCTGY | chr13 | + | 48916131 | 48916138 | 3.4e-05 | 0.188 | `ACAGCTGC` |
| RCAGCTGY | chr13 | − | 101837215 | 101837222 | 3.4e-05 | 0.188 | `ACAGCTGC` |
| RCAGCTGY | chr13 | + | 109922411 | 109922418 | 3.4e-05 | 0.188 | `ACAGCTGC` |
| RCAGCTGY | chr14 | + | 64693560 | 64693567 | 3.4e-05 | 0.188 | `ACAGCTGC` |
| RCAGCTGY | chr14 | − | 90909883 | 90909890 | 3.4e-05 | 0.188 | `ACAGCTGC` |
| RCAGCTGY | chr14 | − | 92240567 | 92240574 | 3.4e-05 | 0.188 | `ACAGCTGC` |
| RCAGCTGY | chr14 | + | 95198919 | 95198926 | 3.4e-05 | 0.188 | `ACAGCTGC` |
| RCAGCTGY | chr14 | + | 102306413 | 102306420 | 3.4e-05 | 0.188 | `ACAGCTGC` |
| RCAGCTGY | chr14 | + | 106240915 | 106240922 | 3.4e-05 | 0.188 | `ACAGCTGC` |
| RCAGCTGY | chr15 | − | 29346120 | 29346127 | 3.4e-05 | 0.188 | `ACAGCTGC` |
| RCAGCTGY | chr15 | − | 29440973 | 29440980 | 3.4e-05 | 0.188 | `ACAGCTGC` |
| RCAGCTGY | chr15 | − | 91184099 | 91184106 | 3.4e-05 | 0.188 | `ACAGCTGC` |
| RCAGCTGY | chr15 | + | 91184054 | 91184061 | 3.4e-05 | 0.188 | `ACAGCTGC` |
| RCAGCTGY | chr16 | + | 10767153 | 10767160 | 3.4e-05 | 0.188 | `ACAGCTGC` |
| RCAGCTGY | chr16 | − | 11330024 | 11330031 | 3.4e-05 | 0.188 | `ACAGCTGC` |
| RCAGCTGY | chr16 | + | 19474212 | 19474219 | 3.4e-05 | 0.188 | `ACAGCTGC` |
| RCAGCTGY | chr16 | − | 23253412 | 23253419 | 3.4e-05 | 0.188 | `ACAGCTGC` |
| RCAGCTGY | chr16 | − | 27321124 | 27321131 | 3.4e-05 | 0.188 | `ACAGCTGC` |
| RCAGCTGY | chr16 | − | 55536822 | 55536829 | 3.4e-05 | 0.188 | `ACAGCTGC` |
| RCAGCTGY | chr16 | + | 66109714 | 66109721 | 3.4e-05 | 0.188 | `ACAGCTGC` |
| RCAGCTGY | chr17 | + | 2665243 | 2665250 | 3.4e-05 | 0.188 | `ACAGCTGC` |
| RCAGCTGY | chr17 | + | 16833080 | 16833087 | 3.4e-05 | 0.188 | `ACAGCTGC` |
| RCAGCTGY | chr17 | − | 37984359 | 37984366 | 3.4e-05 | 0.188 | `ACAGCTGC` |
| RCAGCTGY | chr17 | − | 55274126 | 55274133 | 3.4e-05 | 0.188 | `ACAGCTGC` |
| RCAGCTGY | chr17 | + | 59451691 | 59451698 | 3.4e-05 | 0.188 | `ACAGCTGC` |
| RCAGCTGY | chr17 | + | 60412768 | 60412775 | 3.4e-05 | 0.188 | `ACAGCTGC` |
| RCAGCTGY | chr17 | + | 63077474 | 63077481 | 3.4e-05 | 0.188 | `ACAGCTGC` |
| RCAGCTGY | chr17 | − | 70246252 | 70246259 | 3.4e-05 | 0.188 | `ACAGCTGC` |
| RCAGCTGY | chr17 | − | 70246314 | 70246321 | 3.4e-05 | 0.188 | `ACAGCTGC` |
| RCAGCTGY | chr17 | − | 71988938 | 71988945 | 3.4e-05 | 0.188 | `ACAGCTGC` |
| RCAGCTGY | chr17 | − | 77090980 | 77090987 | 3.4e-05 | 0.188 | `ACAGCTGC` |
| RCAGCTGY | chr17 | − | 77993186 | 77993193 | 3.4e-05 | 0.188 | `ACAGCTGC` |
| RCAGCTGY | chr18 | + | 12857890 | 12857897 | 3.4e-05 | 0.188 | `ACAGCTGC` |
| RCAGCTGY | chr19 | + | 2734611 | 2734618 | 3.4e-05 | 0.188 | `ACAGCTGC` |
| RCAGCTGY | chr19 | + | 12910054 | 12910061 | 3.4e-05 | 0.188 | `ACAGCTGC` |
| RCAGCTGY | chr19 | − | 16557911 | 16557918 | 3.4e-05 | 0.188 | `ACAGCTGC` |
| RCAGCTGY | chr19 | − | 33312865 | 33312872 | 3.4e-05 | 0.188 | `ACAGCTGC` |
| RCAGCTGY | chr19 | + | 44584390 | 44584397 | 3.4e-05 | 0.188 | `ACAGCTGC` |
| RCAGCTGY | chr19 | − | 54070460 | 54070467 | 3.4e-05 | 0.188 | `ACAGCTGC` |
| RCAGCTGY | chr20 | − | 34667299 | 34667306 | 3.4e-05 | 0.188 | `ACAGCTGC` |
| RCAGCTGY | chr20 | + | 49437327 | 49437334 | 3.4e-05 | 0.188 | `ACAGCTGC` |
| RCAGCTGY | chr20 | + | 51889647 | 51889654 | 3.4e-05 | 0.188 | `ACAGCTGC` |
| RCAGCTGY | chr21 | − | 34314346 | 34314353 | 3.4e-05 | 0.188 | `ACAGCTGC` |
| RCAGCTGY | chr22 | + | 27518338 | 27518345 | 3.4e-05 | 0.188 | `ACAGCTGC` |
| RCAGCTGY | chr22 | − | 35588462 | 35588469 | 3.4e-05 | 0.188 | `ACAGCTGC` |
| RCAGCTGY | chr22 | − | 49090131 | 49090138 | 3.4e-05 | 0.188 | `ACAGCTGC` |
| RCAGCTGY | chr22 | − | 49095698 | 49095705 | 3.4e-05 | 0.188 | `ACAGCTGC` |
| RCAGCTGY | chr1 | − | 177209236 | 177209243 | 5.04e-05 | 0.207 | `GCAGCTGT` |
| RCAGCTGY | chr1 | − | 21492839 | 21492846 | 5.04e-05 | 0.207 | `GCAGCTGT` |
| RCAGCTGY | chr1 | + | 28776552 | 28776559 | 5.04e-05 | 0.207 | `GCAGCTGT` |
| RCAGCTGY | chr1 | − | 148806923 | 148806930 | 5.04e-05 | 0.207 | `GCAGCTGT` |
| RCAGCTGY | chr1 | + | 155418451 | 155418458 | 5.04e-05 | 0.207 | `GCAGCTGT` |
| RCAGCTGY | chr1 | + | 210172869 | 210172876 | 5.04e-05 | 0.207 | `GCAGCTGT` |
| RCAGCTGY | chr1 | + | 211221958 | 211221965 | 5.04e-05 | 0.207 | `GCAGCTGT` |
| RCAGCTGY | chr1 | + | 228312489 | 228312496 | 5.04e-05 | 0.207 | `GCAGCTGT` |
| RCAGCTGY | chr2 | − | 27157639 | 27157646 | 5.04e-05 | 0.207 | `GCAGCTGT` |
| RCAGCTGY | chr2 | − | 70170303 | 70170310 | 5.04e-05 | 0.207 | `GCAGCTGT` |
| RCAGCTGY | chr2 | + | 99163833 | 99163840 | 5.04e-05 | 0.207 | `GCAGCTGT` |
| RCAGCTGY | chr2 | + | 136680727 | 136680734 | 5.04e-05 | 0.207 | `GCAGCTGT` |
| RCAGCTGY | chr2 | − | 232281198 | 232281205 | 5.04e-05 | 0.207 | `GCAGCTGT` |
| RCAGCTGY | chr2 | − | 233656354 | 233656361 | 5.04e-05 | 0.207 | `GCAGCTGT` |
| RCAGCTGY | chr3 | + | 6277384 | 6277391 | 5.04e-05 | 0.207 | `GCAGCTGT` |
| RCAGCTGY | chr3 | − | 10240475 | 10240482 | 5.04e-05 | 0.207 | `GCAGCTGT` |
| RCAGCTGY | chr3 | − | 45570351 | 45570358 | 5.04e-05 | 0.207 | `GCAGCTGT` |
| RCAGCTGY | chr3 | − | 45978649 | 45978656 | 5.04e-05 | 0.207 | `GCAGCTGT` |
| RCAGCTGY | chr3 | − | 53112053 | 53112060 | 5.04e-05 | 0.207 | `GCAGCTGT` |
| RCAGCTGY | chr3 | − | 128956860 | 128956867 | 5.04e-05 | 0.207 | `GCAGCTGT` |
| RCAGCTGY | chr3 | + | 169274294 | 169274301 | 5.04e-05 | 0.207 | `GCAGCTGT` |
| RCAGCTGY | chr3 | + | 179719905 | 179719912 | 5.04e-05 | 0.207 | `GCAGCTGT` |
| RCAGCTGY | chr3 | + | 198923535 | 198923542 | 5.04e-05 | 0.207 | `GCAGCTGT` |
| RCAGCTGY | chr4 | + | 2718045 | 2718052 | 5.04e-05 | 0.207 | `GCAGCTGT` |
| RCAGCTGY | chr4 | + | 7529690 | 7529697 | 5.04e-05 | 0.207 | `GCAGCTGT` |
| RCAGCTGY | chr4 | − | 55127438 | 55127445 | 5.04e-05 | 0.207 | `GCAGCTGT` |
| RCAGCTGY | chr4 | − | 77339116 | 77339123 | 5.04e-05 | 0.207 | `GCAGCTGT` |
| RCAGCTGY | chr4 | + | 95123453 | 95123460 | 5.04e-05 | 0.207 | `GCAGCTGT` |
| RCAGCTGY | chr5 | − | 1845946 | 1845953 | 5.04e-05 | 0.207 | `GCAGCTGT` |
| RCAGCTGY | chr5 | + | 43639467 | 43639474 | 5.04e-05 | 0.207 | `GCAGCTGT` |
| RCAGCTGY | chr5 | − | 132608037 | 132608044 | 5.04e-05 | 0.207 | `GCAGCTGT` |
| RCAGCTGY | chr5 | + | 149764039 | 149764046 | 5.04e-05 | 0.207 | `GCAGCTGT` |
| RCAGCTGY | chr6 | + | 309758 | 309765 | 5.04e-05 | 0.207 | `GCAGCTGT` |
| RCAGCTGY | chr6 | + | 310122 | 310129 | 5.04e-05 | 0.207 | `GCAGCTGT` |
| RCAGCTGY | chr6 | + | 337371 | 337378 | 5.04e-05 | 0.207 | `GCAGCTGT` |
| RCAGCTGY | chr6 | − | 22106998 | 22107005 | 5.04e-05 | 0.207 | `GCAGCTGT` |
| RCAGCTGY | chr6 | − | 31431156 | 31431163 | 5.04e-05 | 0.207 | `GCAGCTGT` |
| RCAGCTGY | chr6 | − | 90123980 | 90123987 | 5.04e-05 | 0.207 | `GCAGCTGT` |
| RCAGCTGY | chr7 | − | 7950943 | 7950950 | 5.04e-05 | 0.207 | `GCAGCTGT` |
| RCAGCTGY | chr7 | − | 28487758 | 28487765 | 5.04e-05 | 0.207 | `GCAGCTGT` |
| RCAGCTGY | chr7 | − | 47944570 | 47944577 | 5.04e-05 | 0.207 | `GCAGCTGT` |
| RCAGCTGY | chr7 | + | 92280795 | 92280802 | 5.04e-05 | 0.207 | `GCAGCTGT` |
| RCAGCTGY | chr7 | − | 97600568 | 97600575 | 5.04e-05 | 0.207 | `GCAGCTGT` |
| RCAGCTGY | chr8 | − | 8179713 | 8179720 | 5.04e-05 | 0.207 | `GCAGCTGT` |
| RCAGCTGY | chr8 | − | 72918481 | 72918488 | 5.04e-05 | 0.207 | `GCAGCTGT` |
| RCAGCTGY | chr8 | − | 103617735 | 103617742 | 5.04e-05 | 0.207 | `GCAGCTGT` |
| RCAGCTGY | chr8 | − | 104021693 | 104021700 | 5.04e-05 | 0.207 | `GCAGCTGT` |
| RCAGCTGY | chr8 | + | 125003651 | 125003658 | 5.04e-05 | 0.207 | `GCAGCTGT` |
| RCAGCTGY | chr8 | + | 126415657 | 126415664 | 5.04e-05 | 0.207 | `GCAGCTGT` |
| RCAGCTGY | chr9 | + | 37399278 | 37399285 | 5.04e-05 | 0.207 | `GCAGCTGT` |
| RCAGCTGY | chr9 | + | 114585971 | 114585978 | 5.04e-05 | 0.207 | `GCAGCTGT` |
| RCAGCTGY | chr9 | + | 127043701 | 127043708 | 5.04e-05 | 0.207 | `GCAGCTGT` |
| RCAGCTGY | chrX | − | 38667669 | 38667676 | 5.04e-05 | 0.207 | `GCAGCTGT` |
| RCAGCTGY | chrX | − | 38668085 | 38668092 | 5.04e-05 | 0.207 | `GCAGCTGT` |
| RCAGCTGY | chrX | − | 70759157 | 70759164 | 5.04e-05 | 0.207 | `GCAGCTGT` |
| RCAGCTGY | chr10 | − | 2980582 | 2980589 | 5.04e-05 | 0.207 | `GCAGCTGT` |
| RCAGCTGY | chr10 | − | 69761674 | 69761681 | 5.04e-05 | 0.207 | `GCAGCTGT` |
| RCAGCTGY | chr10 | − | 73765388 | 73765395 | 5.04e-05 | 0.207 | `GCAGCTGT` |
| RCAGCTGY | chr10 | + | 82002338 | 82002345 | 5.04e-05 | 0.207 | `GCAGCTGT` |
| RCAGCTGY | chr10 | + | 82002509 | 82002516 | 5.04e-05 | 0.207 | `GCAGCTGT` |
| RCAGCTGY | chr10 | + | 82002545 | 82002552 | 5.04e-05 | 0.207 | `GCAGCTGT` |
| RCAGCTGY | chr10 | + | 82002653 | 82002660 | 5.04e-05 | 0.207 | `GCAGCTGT` |
| RCAGCTGY | chr10 | + | 82002689 | 82002696 | 5.04e-05 | 0.207 | `GCAGCTGT` |
| RCAGCTGY | chr10 | + | 82002725 | 82002732 | 5.04e-05 | 0.207 | `GCAGCTGT` |
| RCAGCTGY | chr10 | + | 82002761 | 82002768 | 5.04e-05 | 0.207 | `GCAGCTGT` |
| RCAGCTGY | chr10 | − | 90020872 | 90020879 | 5.04e-05 | 0.207 | `GCAGCTGT` |
| RCAGCTGY | chr10 | − | 93851229 | 93851236 | 5.04e-05 | 0.207 | `GCAGCTGT` |
| RCAGCTGY | chr10 | − | 104411191 | 104411198 | 5.04e-05 | 0.207 | `GCAGCTGT` |
| RCAGCTGY | chr10 | − | 126397743 | 126397750 | 5.04e-05 | 0.207 | `GCAGCTGT` |
| RCAGCTGY | chr11 | − | 602165 | 602172 | 5.04e-05 | 0.207 | `GCAGCTGT` |
| RCAGCTGY | chr11 | − | 63750928 | 63750935 | 5.04e-05 | 0.207 | `GCAGCTGT` |
| RCAGCTGY | chr11 | − | 64375737 | 64375744 | 5.04e-05 | 0.207 | `GCAGCTGT` |
| RCAGCTGY | chr11 | + | 65027225 | 65027232 | 5.04e-05 | 0.207 | `GCAGCTGT` |
| RCAGCTGY | chr11 | − | 71823171 | 71823178 | 5.04e-05 | 0.207 | `GCAGCTGT` |
| RCAGCTGY | chr11 | + | 103274671 | 103274678 | 5.04e-05 | 0.207 | `GCAGCTGT` |
| RCAGCTGY | chr11 | + | 117387136 | 117387143 | 5.04e-05 | 0.207 | `GCAGCTGT` |
| RCAGCTGY | chr11 | + | 118068633 | 118068640 | 5.04e-05 | 0.207 | `GCAGCTGT` |
| RCAGCTGY | chr12 | − | 2335372 | 2335379 | 5.04e-05 | 0.207 | `GCAGCTGT` |
| RCAGCTGY | chr12 | + | 6938500 | 6938507 | 5.04e-05 | 0.207 | `GCAGCTGT` |
| RCAGCTGY | chr12 | + | 12054486 | 12054493 | 5.04e-05 | 0.207 | `GCAGCTGT` |
| RCAGCTGY | chr12 | + | 67522030 | 67522037 | 5.04e-05 | 0.207 | `GCAGCTGT` |
| RCAGCTGY | chr12 | − | 74640243 | 74640250 | 5.04e-05 | 0.207 | `GCAGCTGT` |
| RCAGCTGY | chr12 | + | 91321757 | 91321764 | 5.04e-05 | 0.207 | `GCAGCTGT` |
| RCAGCTGY | chr12 | − | 93480066 | 93480073 | 5.04e-05 | 0.207 | `GCAGCTGT` |
| RCAGCTGY | chr12 | − | 93480281 | 93480288 | 5.04e-05 | 0.207 | `GCAGCTGT` |
| RCAGCTGY | chr12 | − | 107551668 | 107551675 | 5.04e-05 | 0.207 | `GCAGCTGT` |
| RCAGCTGY | chr12 | + | 112130475 | 112130482 | 5.04e-05 | 0.207 | `GCAGCTGT` |
| RCAGCTGY | chr12 | + | 123961128 | 123961135 | 5.04e-05 | 0.207 | `GCAGCTGT` |
| RCAGCTGY | chr13 | − | 48916131 | 48916138 | 5.04e-05 | 0.207 | `GCAGCTGT` |
| RCAGCTGY | chr13 | + | 101837215 | 101837222 | 5.04e-05 | 0.207 | `GCAGCTGT` |
| RCAGCTGY | chr13 | − | 109922411 | 109922418 | 5.04e-05 | 0.207 | `GCAGCTGT` |
| RCAGCTGY | chr14 | − | 64693560 | 64693567 | 5.04e-05 | 0.207 | `GCAGCTGT` |
| RCAGCTGY | chr14 | + | 90909883 | 90909890 | 5.04e-05 | 0.207 | `GCAGCTGT` |
| RCAGCTGY | chr14 | + | 92240567 | 92240574 | 5.04e-05 | 0.207 | `GCAGCTGT` |
| RCAGCTGY | chr14 | − | 95198919 | 95198926 | 5.04e-05 | 0.207 | `GCAGCTGT` |
| RCAGCTGY | chr14 | − | 102306413 | 102306420 | 5.04e-05 | 0.207 | `GCAGCTGT` |
| RCAGCTGY | chr14 | − | 106240915 | 106240922 | 5.04e-05 | 0.207 | `GCAGCTGT` |
| RCAGCTGY | chr15 | + | 29346120 | 29346127 | 5.04e-05 | 0.207 | `GCAGCTGT` |
| RCAGCTGY | chr15 | + | 29440973 | 29440980 | 5.04e-05 | 0.207 | `GCAGCTGT` |
| RCAGCTGY | chr15 | − | 91184054 | 91184061 | 5.04e-05 | 0.207 | `GCAGCTGT` |
| RCAGCTGY | chr15 | + | 91184099 | 91184106 | 5.04e-05 | 0.207 | `GCAGCTGT` |
| RCAGCTGY | chr16 | − | 10767153 | 10767160 | 5.04e-05 | 0.207 | `GCAGCTGT` |
| RCAGCTGY | chr16 | + | 11330024 | 11330031 | 5.04e-05 | 0.207 | `GCAGCTGT` |
| RCAGCTGY | chr16 | − | 19474212 | 19474219 | 5.04e-05 | 0.207 | `GCAGCTGT` |
| RCAGCTGY | chr16 | + | 23253412 | 23253419 | 5.04e-05 | 0.207 | `GCAGCTGT` |
| RCAGCTGY | chr16 | + | 27321124 | 27321131 | 5.04e-05 | 0.207 | `GCAGCTGT` |
| RCAGCTGY | chr16 | + | 55536822 | 55536829 | 5.04e-05 | 0.207 | `GCAGCTGT` |
| RCAGCTGY | chr16 | − | 66109714 | 66109721 | 5.04e-05 | 0.207 | `GCAGCTGT` |
| RCAGCTGY | chr17 | − | 2665243 | 2665250 | 5.04e-05 | 0.207 | `GCAGCTGT` |
| RCAGCTGY | chr17 | − | 16833080 | 16833087 | 5.04e-05 | 0.207 | `GCAGCTGT` |
| RCAGCTGY | chr17 | + | 37984359 | 37984366 | 5.04e-05 | 0.207 | `GCAGCTGT` |
| RCAGCTGY | chr17 | + | 55274126 | 55274133 | 5.04e-05 | 0.207 | `GCAGCTGT` |
| RCAGCTGY | chr17 | − | 59451691 | 59451698 | 5.04e-05 | 0.207 | `GCAGCTGT` |
| RCAGCTGY | chr17 | − | 60412768 | 60412775 | 5.04e-05 | 0.207 | `GCAGCTGT` |
| RCAGCTGY | chr17 | − | 63077474 | 63077481 | 5.04e-05 | 0.207 | `GCAGCTGT` |
| RCAGCTGY | chr17 | + | 70246252 | 70246259 | 5.04e-05 | 0.207 | `GCAGCTGT` |
| RCAGCTGY | chr17 | + | 70246314 | 70246321 | 5.04e-05 | 0.207 | `GCAGCTGT` |
| RCAGCTGY | chr17 | + | 71988938 | 71988945 | 5.04e-05 | 0.207 | `GCAGCTGT` |
| RCAGCTGY | chr17 | + | 77090980 | 77090987 | 5.04e-05 | 0.207 | `GCAGCTGT` |
| RCAGCTGY | chr17 | + | 77993186 | 77993193 | 5.04e-05 | 0.207 | `GCAGCTGT` |
| RCAGCTGY | chr18 | − | 12857890 | 12857897 | 5.04e-05 | 0.207 | `GCAGCTGT` |
| RCAGCTGY | chr19 | − | 2734611 | 2734618 | 5.04e-05 | 0.207 | `GCAGCTGT` |
| RCAGCTGY | chr19 | − | 12910054 | 12910061 | 5.04e-05 | 0.207 | `GCAGCTGT` |
| RCAGCTGY | chr19 | + | 16557911 | 16557918 | 5.04e-05 | 0.207 | `GCAGCTGT` |
| RCAGCTGY | chr19 | + | 33312865 | 33312872 | 5.04e-05 | 0.207 | `GCAGCTGT` |
| RCAGCTGY | chr19 | − | 44584390 | 44584397 | 5.04e-05 | 0.207 | `GCAGCTGT` |
| RCAGCTGY | chr19 | + | 54070460 | 54070467 | 5.04e-05 | 0.207 | `GCAGCTGT` |
| RCAGCTGY | chr20 | + | 34667299 | 34667306 | 5.04e-05 | 0.207 | `GCAGCTGT` |
| RCAGCTGY | chr20 | − | 49437327 | 49437334 | 5.04e-05 | 0.207 | `GCAGCTGT` |
| RCAGCTGY | chr20 | − | 51889647 | 51889654 | 5.04e-05 | 0.207 | `GCAGCTGT` |
| RCAGCTGY | chr21 | + | 34314346 | 34314353 | 5.04e-05 | 0.207 | `GCAGCTGT` |
| RCAGCTGY | chr22 | − | 27518338 | 27518345 | 5.04e-05 | 0.207 | `GCAGCTGT` |
| RCAGCTGY | chr22 | + | 35588462 | 35588469 | 5.04e-05 | 0.207 | `GCAGCTGT` |
| RCAGCTGY | chr22 | + | 49090131 | 49090138 | 5.04e-05 | 0.207 | `GCAGCTGT` |
| RCAGCTGY | chr22 | + | 49095698 | 49095705 | 5.04e-05 | 0.207 | `GCAGCTGT` |
| RCAGCTGY | chr1 | + | 67998485 | 67998492 | 6.55e-05 | 0.222 | `ACAGCTGT` |
| RCAGCTGY | chr1 | − | 67998485 | 67998492 | 6.55e-05 | 0.222 | `ACAGCTGT` |
| RCAGCTGY | chr1 | + | 101473710 | 101473717 | 6.55e-05 | 0.222 | `ACAGCTGT` |
| RCAGCTGY | chr1 | − | 101473710 | 101473717 | 6.55e-05 | 0.222 | `ACAGCTGT` |
| RCAGCTGY | chr1 | + | 171651684 | 171651691 | 6.55e-05 | 0.222 | `ACAGCTGT` |
| RCAGCTGY | chr1 | − | 171651684 | 171651691 | 6.55e-05 | 0.222 | `ACAGCTGT` |
| RCAGCTGY | chr1 | + | 172099125 | 172099132 | 6.55e-05 | 0.222 | `ACAGCTGT` |
| RCAGCTGY | chr1 | − | 172099125 | 172099132 | 6.55e-05 | 0.222 | `ACAGCTGT` |
| RCAGCTGY | chr2 | + | 20931813 | 20931820 | 6.55e-05 | 0.222 | `ACAGCTGT` |
| RCAGCTGY | chr2 | − | 20931813 | 20931820 | 6.55e-05 | 0.222 | `ACAGCTGT` |
| RCAGCTGY | chr2 | + | 158004401 | 158004408 | 6.55e-05 | 0.222 | `ACAGCTGT` |
| RCAGCTGY | chr2 | − | 158004401 | 158004408 | 6.55e-05 | 0.222 | `ACAGCTGT` |
| RCAGCTGY | chr2 | + | 230989472 | 230989479 | 6.55e-05 | 0.222 | `ACAGCTGT` |
| RCAGCTGY | chr2 | − | 230989472 | 230989479 | 6.55e-05 | 0.222 | `ACAGCTGT` |
| RCAGCTGY | chr2 | + | 231443936 | 231443943 | 6.55e-05 | 0.222 | `ACAGCTGT` |
| RCAGCTGY | chr2 | − | 231443936 | 231443943 | 6.55e-05 | 0.222 | `ACAGCTGT` |
| RCAGCTGY | chr3 | + | 314642 | 314649 | 6.55e-05 | 0.222 | `ACAGCTGT` |
| RCAGCTGY | chr3 | − | 314642 | 314649 | 6.55e-05 | 0.222 | `ACAGCTGT` |
| RCAGCTGY | chr3 | + | 4606548 | 4606555 | 6.55e-05 | 0.222 | `ACAGCTGT` |
| RCAGCTGY | chr3 | − | 4606548 | 4606555 | 6.55e-05 | 0.222 | `ACAGCTGT` |
| RCAGCTGY | chr3 | + | 16528849 | 16528856 | 6.55e-05 | 0.222 | `ACAGCTGT` |
| RCAGCTGY | chr3 | − | 16528849 | 16528856 | 6.55e-05 | 0.222 | `ACAGCTGT` |
| RCAGCTGY | chr3 | + | 45570325 | 45570332 | 6.55e-05 | 0.222 | `ACAGCTGT` |
| RCAGCTGY | chr3 | − | 45570325 | 45570332 | 6.55e-05 | 0.222 | `ACAGCTGT` |
| RCAGCTGY | chr3 | + | 45571020 | 45571027 | 6.55e-05 | 0.222 | `ACAGCTGT` |
| RCAGCTGY | chr3 | − | 45571020 | 45571027 | 6.55e-05 | 0.222 | `ACAGCTGT` |
| RCAGCTGY | chr3 | + | 134692768 | 134692775 | 6.55e-05 | 0.222 | `ACAGCTGT` |
| RCAGCTGY | chr3 | − | 134692768 | 134692775 | 6.55e-05 | 0.222 | `ACAGCTGT` |
| RCAGCTGY | chr3 | + | 197308147 | 197308154 | 6.55e-05 | 0.222 | `ACAGCTGT` |
| RCAGCTGY | chr3 | − | 197308147 | 197308154 | 6.55e-05 | 0.222 | `ACAGCTGT` |
| RCAGCTGY | chr4 | + | 54309598 | 54309605 | 6.55e-05 | 0.222 | `ACAGCTGT` |
| RCAGCTGY | chr4 | − | 54309598 | 54309605 | 6.55e-05 | 0.222 | `ACAGCTGT` |
| RCAGCTGY | chr6 | + | 7838725 | 7838732 | 6.55e-05 | 0.222 | `ACAGCTGT` |
| RCAGCTGY | chr6 | − | 7838725 | 7838732 | 6.55e-05 | 0.222 | `ACAGCTGT` |
| RCAGCTGY | chr6 | + | 7844240 | 7844247 | 6.55e-05 | 0.222 | `ACAGCTGT` |
| RCAGCTGY | chr6 | − | 7844240 | 7844247 | 6.55e-05 | 0.222 | `ACAGCTGT` |
| RCAGCTGY | chr6 | + | 119805777 | 119805784 | 6.55e-05 | 0.222 | `ACAGCTGT` |
| RCAGCTGY | chr6 | − | 119805777 | 119805784 | 6.55e-05 | 0.222 | `ACAGCTGT` |
| RCAGCTGY | chr7 | + | 7951242 | 7951249 | 6.55e-05 | 0.222 | `ACAGCTGT` |
| RCAGCTGY | chr7 | − | 7951242 | 7951249 | 6.55e-05 | 0.222 | `ACAGCTGT` |
| RCAGCTGY | chr7 | + | 101853328 | 101853335 | 6.55e-05 | 0.222 | `ACAGCTGT` |
| RCAGCTGY | chr7 | − | 101853328 | 101853335 | 6.55e-05 | 0.222 | `ACAGCTGT` |
| RCAGCTGY | chr7 | + | 139113837 | 139113844 | 6.55e-05 | 0.222 | `ACAGCTGT` |
| RCAGCTGY | chr7 | − | 139113837 | 139113844 | 6.55e-05 | 0.222 | `ACAGCTGT` |
| RCAGCTGY | chr8 | + | 101998711 | 101998718 | 6.55e-05 | 0.222 | `ACAGCTGT` |
| RCAGCTGY | chr8 | − | 101998711 | 101998718 | 6.55e-05 | 0.222 | `ACAGCTGT` |
| RCAGCTGY | chr8 | + | 119361027 | 119361034 | 6.55e-05 | 0.222 | `ACAGCTGT` |
| RCAGCTGY | chr8 | − | 119361027 | 119361034 | 6.55e-05 | 0.222 | `ACAGCTGT` |
| RCAGCTGY | chr8 | + | 125719199 | 125719206 | 6.55e-05 | 0.222 | `ACAGCTGT` |
| RCAGCTGY | chr8 | − | 125719199 | 125719206 | 6.55e-05 | 0.222 | `ACAGCTGT` |
| RCAGCTGY | chr8 | + | 129303795 | 129303802 | 6.55e-05 | 0.222 | `ACAGCTGT` |
| RCAGCTGY | chr8 | − | 129303795 | 129303802 | 6.55e-05 | 0.222 | `ACAGCTGT` |
| RCAGCTGY | chr9 | + | 129883285 | 129883292 | 6.55e-05 | 0.222 | `ACAGCTGT` |
| RCAGCTGY | chr9 | − | 129883285 | 129883292 | 6.55e-05 | 0.222 | `ACAGCTGT` |
| RCAGCTGY | chrX | + | 7043755 | 7043762 | 6.55e-05 | 0.222 | `ACAGCTGT` |
| RCAGCTGY | chrX | − | 7043755 | 7043762 | 6.55e-05 | 0.222 | `ACAGCTGT` |
| RCAGCTGY | chrX | + | 129054074 | 129054081 | 6.55e-05 | 0.222 | `ACAGCTGT` |
| RCAGCTGY | chrX | − | 129054074 | 129054081 | 6.55e-05 | 0.222 | `ACAGCTGT` |
| RCAGCTGY | chr10 | + | 301113 | 301120 | 6.55e-05 | 0.222 | `ACAGCTGT` |
| RCAGCTGY | chr10 | − | 301113 | 301120 | 6.55e-05 | 0.222 | `ACAGCTGT` |
| RCAGCTGY | chr10 | + | 11324528 | 11324535 | 6.55e-05 | 0.222 | `ACAGCTGT` |
| RCAGCTGY | chr10 | − | 11324528 | 11324535 | 6.55e-05 | 0.222 | `ACAGCTGT` |
| RCAGCTGY | chr11 | + | 64656448 | 64656455 | 6.55e-05 | 0.222 | `ACAGCTGT` |
| RCAGCTGY | chr11 | − | 64656448 | 64656455 | 6.55e-05 | 0.222 | `ACAGCTGT` |
| RCAGCTGY | chr11 | + | 64656691 | 64656698 | 6.55e-05 | 0.222 | `ACAGCTGT` |
| RCAGCTGY | chr11 | − | 64656691 | 64656698 | 6.55e-05 | 0.222 | `ACAGCTGT` |
| RCAGCTGY | chr11 | + | 110726311 | 110726318 | 6.55e-05 | 0.222 | `ACAGCTGT` |
| RCAGCTGY | chr11 | − | 110726311 | 110726318 | 6.55e-05 | 0.222 | `ACAGCTGT` |
| RCAGCTGY | chr11 | + | 128216880 | 128216887 | 6.55e-05 | 0.222 | `ACAGCTGT` |
| RCAGCTGY | chr11 | − | 128216880 | 128216887 | 6.55e-05 | 0.222 | `ACAGCTGT` |
| RCAGCTGY | chr12 | + | 63346235 | 63346242 | 6.55e-05 | 0.222 | `ACAGCTGT` |
| RCAGCTGY | chr12 | − | 63346235 | 63346242 | 6.55e-05 | 0.222 | `ACAGCTGT` |
| RCAGCTGY | chr12 | + | 67487730 | 67487737 | 6.55e-05 | 0.222 | `ACAGCTGT` |
| RCAGCTGY | chr12 | − | 67487730 | 67487737 | 6.55e-05 | 0.222 | `ACAGCTGT` |
| RCAGCTGY | chr12 | + | 67487856 | 67487863 | 6.55e-05 | 0.222 | `ACAGCTGT` |
| RCAGCTGY | chr12 | − | 67487856 | 67487863 | 6.55e-05 | 0.222 | `ACAGCTGT` |
| RCAGCTGY | chr12 | + | 91905974 | 91905981 | 6.55e-05 | 0.222 | `ACAGCTGT` |
| RCAGCTGY | chr12 | − | 91905974 | 91905981 | 6.55e-05 | 0.222 | `ACAGCTGT` |
| RCAGCTGY | chr12 | + | 120951353 | 120951360 | 6.55e-05 | 0.222 | `ACAGCTGT` |
| RCAGCTGY | chr12 | − | 120951353 | 120951360 | 6.55e-05 | 0.222 | `ACAGCTGT` |
| RCAGCTGY | chr12 | + | 122134728 | 122134735 | 6.55e-05 | 0.222 | `ACAGCTGT` |
| RCAGCTGY | chr12 | − | 122134728 | 122134735 | 6.55e-05 | 0.222 | `ACAGCTGT` |
| RCAGCTGY | chr14 | + | 49598373 | 49598380 | 6.55e-05 | 0.222 | `ACAGCTGT` |
| RCAGCTGY | chr14 | − | 49598373 | 49598380 | 6.55e-05 | 0.222 | `ACAGCTGT` |
| RCAGCTGY | chr14 | + | 54638945 | 54638952 | 6.55e-05 | 0.222 | `ACAGCTGT` |
| RCAGCTGY | chr14 | − | 54638945 | 54638952 | 6.55e-05 | 0.222 | `ACAGCTGT` |
| RCAGCTGY | chr14 | + | 58698413 | 58698420 | 6.55e-05 | 0.222 | `ACAGCTGT` |
| RCAGCTGY | chr14 | − | 58698413 | 58698420 | 6.55e-05 | 0.222 | `ACAGCTGT` |
| RCAGCTGY | chr16 | + | 8937581 | 8937588 | 6.55e-05 | 0.222 | `ACAGCTGT` |
| RCAGCTGY | chr16 | − | 8937581 | 8937588 | 6.55e-05 | 0.222 | `ACAGCTGT` |
| RCAGCTGY | chr16 | + | 27151068 | 27151075 | 6.55e-05 | 0.222 | `ACAGCTGT` |
| RCAGCTGY | chr16 | − | 27151068 | 27151075 | 6.55e-05 | 0.222 | `ACAGCTGT` |
| RCAGCTGY | chr17 | + | 9300494 | 9300501 | 6.55e-05 | 0.222 | `ACAGCTGT` |
| RCAGCTGY | chr17 | − | 9300494 | 9300501 | 6.55e-05 | 0.222 | `ACAGCTGT` |
| RCAGCTGY | chr17 | + | 30724174 | 30724181 | 6.55e-05 | 0.222 | `ACAGCTGT` |
| RCAGCTGY | chr17 | − | 30724174 | 30724181 | 6.55e-05 | 0.222 | `ACAGCTGT` |
| RCAGCTGY | chr17 | + | 72001053 | 72001060 | 6.55e-05 | 0.222 | `ACAGCTGT` |
| RCAGCTGY | chr17 | − | 72001053 | 72001060 | 6.55e-05 | 0.222 | `ACAGCTGT` |
| RCAGCTGY | chr18 | + | 64811094 | 64811101 | 6.55e-05 | 0.222 | `ACAGCTGT` |
| RCAGCTGY | chr18 | − | 64811094 | 64811101 | 6.55e-05 | 0.222 | `ACAGCTGT` |
| RCAGCTGY | chr19 | + | 50901785 | 50901792 | 6.55e-05 | 0.222 | `ACAGCTGT` |
| RCAGCTGY | chr19 | − | 50901785 | 50901792 | 6.55e-05 | 0.222 | `ACAGCTGT` |
| RCAGCTGY | chr20 | + | 46788913 | 46788920 | 6.55e-05 | 0.222 | `ACAGCTGT` |
| RCAGCTGY | chr20 | − | 46788913 | 46788920 | 6.55e-05 | 0.222 | `ACAGCTGT` |
| RCAGCTGY | chr22 | + | 38248979 | 38248986 | 6.55e-05 | 0.222 | `ACAGCTGT` |
| RCAGCTGY | chr22 | − | 38248979 | 38248986 | 6.55e-05 | 0.222 | `ACAGCTGT` |
| RCAGCTGY | chr22 | + | 48052026 | 48052033 | 6.55e-05 | 0.222 | `ACAGCTGT` |
| RCAGCTGY | chr22 | − | 48052026 | 48052033 | 6.55e-05 | 0.222 | `ACAGCTGT` |
| RCAGCTGY | chr22 | + | 48052100 | 48052107 | 6.55e-05 | 0.222 | `ACAGCTGT` |
| RCAGCTGY | chr22 | − | 48052100 | 48052107 | 6.55e-05 | 0.222 | `ACAGCTGT` |
| RCAGCTGY | chr22 | + | 48052160 | 48052167 | 6.55e-05 | 0.222 | `ACAGCTGT` |
| RCAGCTGY | chr22 | − | 48052160 | 48052167 | 6.55e-05 | 0.222 | `ACAGCTGT` |
| RCAGCTGY | chr22 | + | 48052228 | 48052235 | 6.55e-05 | 0.222 | `ACAGCTGT` |
| RCAGCTGY | chr22 | − | 48052228 | 48052235 | 6.55e-05 | 0.222 | `ACAGCTGT` |
| RCAGCTGY | chr1 | + | 177209144 | 177209151 | 9.95e-05 | 0.235 | `CCAGCTGC` |
| RCAGCTGY | chr1 | − | 3583659 | 3583666 | 9.95e-05 | 0.235 | `CCAGCTGC` |
| RCAGCTGY | chr1 | + | 11892301 | 11892308 | 9.95e-05 | 0.235 | `CCAGCTGC` |
| RCAGCTGY | chr1 | + | 12034634 | 12034641 | 9.95e-05 | 0.235 | `CCAGCTGC` |
| RCAGCTGY | chr1 | + | 12039482 | 12039489 | 9.95e-05 | 0.235 | `CCAGCTGC` |
| RCAGCTGY | chr1 | − | 21492017 | 21492024 | 9.95e-05 | 0.235 | `CCAGCTGC` |
| RCAGCTGY | chr1 | − | 28847802 | 28847809 | 9.95e-05 | 0.235 | `CCAGCTGC` |
| RCAGCTGY | chr1 | + | 30978122 | 30978129 | 9.95e-05 | 0.235 | `CCAGCTGC` |
| RCAGCTGY | chr1 | − | 32488310 | 32488317 | 9.95e-05 | 0.235 | `TCAGCTGC` |
| RCAGCTGY | chr1 | + | 38713279 | 38713286 | 9.95e-05 | 0.235 | `TCAGCTGC` |
| RCAGCTGY | chr1 | − | 45048069 | 45048076 | 9.95e-05 | 0.235 | `CCAGCTGC` |
| RCAGCTGY | chr1 | + | 59206653 | 59206660 | 9.95e-05 | 0.235 | `CCAGCTGC` |
| RCAGCTGY | chr1 | + | 67913131 | 67913138 | 9.95e-05 | 0.235 | `CCAGCTGC` |
| RCAGCTGY | chr1 | + | 110979085 | 110979092 | 9.95e-05 | 0.235 | `CCAGCTGC` |
| RCAGCTGY | chr1 | − | 111937213 | 111937220 | 9.95e-05 | 0.235 | `TCAGCTGC` |
| RCAGCTGY | chr1 | + | 115417565 | 115417572 | 9.95e-05 | 0.235 | `TCAGCTGC` |
| RCAGCTGY | chr1 | + | 148398765 | 148398772 | 9.95e-05 | 0.235 | `TCAGCTGC` |
| RCAGCTGY | chr1 | − | 152185309 | 152185316 | 9.95e-05 | 0.235 | `CCAGCTGC` |
| RCAGCTGY | chr1 | − | 154452852 | 154452859 | 9.95e-05 | 0.235 | `TCAGCTGC` |
| RCAGCTGY | chr1 | − | 154741495 | 154741502 | 9.95e-05 | 0.235 | `TCAGCTGC` |
| RCAGCTGY | chr1 | + | 154741848 | 154741855 | 9.95e-05 | 0.235 | `CCAGCTGC` |
| RCAGCTGY | chr1 | − | 155413476 | 155413483 | 9.95e-05 | 0.235 | `TCAGCTGC` |
| RCAGCTGY | chr1 | − | 158910983 | 158910990 | 9.95e-05 | 0.235 | `CCAGCTGC` |
| RCAGCTGY | chr1 | + | 165898953 | 165898960 | 9.95e-05 | 0.235 | `CCAGCTGC` |
| RCAGCTGY | chr1 | + | 165899202 | 165899209 | 9.95e-05 | 0.235 | `CCAGCTGC` |
| RCAGCTGY | chr1 | + | 165899215 | 165899222 | 9.95e-05 | 0.235 | `CCAGCTGC` |
| RCAGCTGY | chr1 | + | 176817231 | 176817238 | 9.95e-05 | 0.235 | `CCAGCTGC` |
| RCAGCTGY | chr1 | − | 179634370 | 179634377 | 9.95e-05 | 0.235 | `CCAGCTGC` |
| RCAGCTGY | chr1 | − | 179634533 | 179634540 | 9.95e-05 | 0.235 | `CCAGCTGC` |
| RCAGCTGY | chr1 | − | 201556667 | 201556674 | 9.95e-05 | 0.235 | `CCAGCTGC` |
| RCAGCTGY | chr1 | + | 201556783 | 201556790 | 9.95e-05 | 0.235 | `CCAGCTGC` |
| RCAGCTGY | chr1 | − | 201597887 | 201597894 | 9.95e-05 | 0.235 | `TCAGCTGC` |
| RCAGCTGY | chr1 | + | 203524226 | 203524233 | 9.95e-05 | 0.235 | `TCAGCTGC` |
| RCAGCTGY | chr1 | + | 203524266 | 203524273 | 9.95e-05 | 0.235 | `CCAGCTGC` |
| RCAGCTGY | chr1 | − | 232725060 | 232725067 | 9.95e-05 | 0.235 | `CCAGCTGC` |
| RCAGCTGY | chr1 | + | 232725178 | 232725185 | 9.95e-05 | 0.235 | `CCAGCTGC` |
| RCAGCTGY | chr2 | + | 8359918 | 8359925 | 9.95e-05 | 0.235 | `TCAGCTGC` |
| RCAGCTGY | chr2 | − | 28663494 | 28663501 | 9.95e-05 | 0.235 | `TCAGCTGC` |
| RCAGCTGY | chr2 | − | 33556092 | 33556099 | 9.95e-05 | 0.235 | `TCAGCTGC` |
| RCAGCTGY | chr2 | + | 37735813 | 37735820 | 9.95e-05 | 0.235 | `CCAGCTGC` |
| RCAGCTGY | chr2 | + | 111631887 | 111631894 | 9.95e-05 | 0.235 | `TCAGCTGC` |
| RCAGCTGY | chr2 | + | 111632151 | 111632158 | 9.95e-05 | 0.235 | `TCAGCTGC` |
| RCAGCTGY | chr2 | − | 114360701 | 114360708 | 9.95e-05 | 0.235 | `TCAGCTGC` |
| RCAGCTGY | chr2 | − | 129273283 | 129273290 | 9.95e-05 | 0.235 | `CCAGCTGC` |
| RCAGCTGY | chr2 | − | 136680869 | 136680876 | 9.95e-05 | 0.235 | `CCAGCTGC` |
| RCAGCTGY | chr2 | + | 160277030 | 160277037 | 9.95e-05 | 0.235 | `CCAGCTGC` |
| RCAGCTGY | chr2 | − | 201696922 | 201696929 | 9.95e-05 | 0.235 | `CCAGCTGC` |
| RCAGCTGY | chr2 | − | 204600200 | 204600207 | 9.95e-05 | 0.235 | `CCAGCTGC` |
| RCAGCTGY | chr2 | − | 216531473 | 216531480 | 9.95e-05 | 0.235 | `CCAGCTGC` |
| RCAGCTGY | chr2 | + | 231444206 | 231444213 | 9.95e-05 | 0.235 | `CCAGCTGC` |
| RCAGCTGY | chr2 | + | 231557783 | 231557790 | 9.95e-05 | 0.235 | `CCAGCTGC` |
| RCAGCTGY | chr2 | + | 232026053 | 232026060 | 9.95e-05 | 0.235 | `CCAGCTGC` |
| RCAGCTGY | chr3 | + | 45612932 | 45612939 | 9.95e-05 | 0.235 | `TCAGCTGC` |
| RCAGCTGY | chr3 | − | 45612990 | 45612997 | 9.95e-05 | 0.235 | `CCAGCTGC` |
| RCAGCTGY | chr3 | + | 12912619 | 12912626 | 9.95e-05 | 0.235 | `CCAGCTGC` |
| RCAGCTGY | chr3 | − | 16529029 | 16529036 | 9.95e-05 | 0.235 | `TCAGCTGC` |
| RCAGCTGY | chr3 | + | 45978693 | 45978700 | 9.95e-05 | 0.235 | `CCAGCTGC` |
| RCAGCTGY | chr3 | − | 47397241 | 47397248 | 9.95e-05 | 0.235 | `CCAGCTGC` |
| RCAGCTGY | chr3 | − | 49019615 | 49019622 | 9.95e-05 | 0.235 | `CCAGCTGC` |
| RCAGCTGY | chr3 | + | 53118187 | 53118194 | 9.95e-05 | 0.235 | `CCAGCTGC` |
| RCAGCTGY | chr3 | + | 109327247 | 109327254 | 9.95e-05 | 0.235 | `TCAGCTGC` |
| RCAGCTGY | chr3 | − | 120781538 | 120781545 | 9.95e-05 | 0.235 | `TCAGCTGC` |
| RCAGCTGY | chr3 | + | 134692734 | 134692741 | 9.95e-05 | 0.235 | `TCAGCTGC` |
| RCAGCTGY | chr3 | − | 157886378 | 157886385 | 9.95e-05 | 0.235 | `TCAGCTGC` |
| RCAGCTGY | chr3 | − | 184362380 | 184362387 | 9.95e-05 | 0.235 | `CCAGCTGC` |
| RCAGCTGY | chr3 | + | 185085370 | 185085377 | 9.95e-05 | 0.235 | `CCAGCTGC` |
| RCAGCTGY | chr3 | − | 187989521 | 187989528 | 9.95e-05 | 0.235 | `CCAGCTGC` |
| RCAGCTGY | chr3 | + | 189012048 | 189012055 | 9.95e-05 | 0.235 | `TCAGCTGC` |
| RCAGCTGY | chr3 | − | 196349701 | 196349708 | 9.95e-05 | 0.235 | `CCAGCTGC` |
| RCAGCTGY | chr3 | − | 198154026 | 198154033 | 9.95e-05 | 0.235 | `CCAGCTGC` |
| RCAGCTGY | chr4 | − | 2759224 | 2759231 | 9.95e-05 | 0.235 | `TCAGCTGC` |
| RCAGCTGY | chr4 | + | 2908470 | 2908477 | 9.95e-05 | 0.235 | `CCAGCTGC` |
| RCAGCTGY | chr4 | + | 7529643 | 7529650 | 9.95e-05 | 0.235 | `CCAGCTGC` |
| RCAGCTGY | chr4 | − | 54309362 | 54309369 | 9.95e-05 | 0.235 | `CCAGCTGC` |
| RCAGCTGY | chr4 | − | 54309495 | 54309502 | 9.95e-05 | 0.235 | `TCAGCTGC` |
| RCAGCTGY | chr4 | + | 103967848 | 103967855 | 9.95e-05 | 0.235 | `CCAGCTGC` |
| RCAGCTGY | chr4 | + | 114705732 | 114705739 | 9.95e-05 | 0.235 | `CCAGCTGC` |
| RCAGCTGY | chr4 | + | 121207774 | 121207781 | 9.95e-05 | 0.235 | `CCAGCTGC` |
| RCAGCTGY | chr4 | − | 154628982 | 154628989 | 9.95e-05 | 0.235 | `CCAGCTGC` |
| RCAGCTGY | chr4 | − | 184602471 | 184602478 | 9.95e-05 | 0.235 | `TCAGCTGC` |
| RCAGCTGY | chr4 | − | 186301290 | 186301297 | 9.95e-05 | 0.235 | `CCAGCTGC` |
| RCAGCTGY | chr5 | − | 10660674 | 10660681 | 9.95e-05 | 0.235 | `TCAGCTGC` |
| RCAGCTGY | chr5 | − | 32212567 | 32212574 | 9.95e-05 | 0.235 | `CCAGCTGC` |
| RCAGCTGY | chr5 | + | 32573978 | 32573985 | 9.95e-05 | 0.235 | `TCAGCTGC` |
| RCAGCTGY | chr5 | − | 37774308 | 37774315 | 9.95e-05 | 0.235 | `TCAGCTGC` |
| RCAGCTGY | chr5 | − | 43639604 | 43639611 | 9.95e-05 | 0.235 | `CCAGCTGC` |
| RCAGCTGY | chr5 | + | 78059821 | 78059828 | 9.95e-05 | 0.235 | `CCAGCTGC` |
| RCAGCTGY | chr5 | + | 88675401 | 88675408 | 9.95e-05 | 0.235 | `CCAGCTGC` |
| RCAGCTGY | chr5 | − | 98297392 | 98297399 | 9.95e-05 | 0.235 | `CCAGCTGC` |
| RCAGCTGY | chr5 | + | 138996140 | 138996147 | 9.95e-05 | 0.235 | `TCAGCTGC` |
| RCAGCTGY | chr5 | + | 139907548 | 139907555 | 9.95e-05 | 0.235 | `CCAGCTGC` |
| RCAGCTGY | chr5 | + | 149760722 | 149760729 | 9.95e-05 | 0.235 | `CCAGCTGC` |
| RCAGCTGY | chr5 | − | 150455366 | 150455373 | 9.95e-05 | 0.235 | `CCAGCTGC` |
| RCAGCTGY | chr5 | − | 156862066 | 156862073 | 9.95e-05 | 0.235 | `CCAGCTGC` |
| RCAGCTGY | chr6 | − | 309682 | 309689 | 9.95e-05 | 0.235 | `TCAGCTGC` |
| RCAGCTGY | chr6 | + | 349463 | 349470 | 9.95e-05 | 0.235 | `TCAGCTGC` |
| RCAGCTGY | chr6 | + | 7832955 | 7832962 | 9.95e-05 | 0.235 | `CCAGCTGC` |
| RCAGCTGY | chr6 | + | 11942699 | 11942706 | 9.95e-05 | 0.235 | `CCAGCTGC` |
| RCAGCTGY | chr6 | − | 18264207 | 18264214 | 9.95e-05 | 0.235 | `CCAGCTGC` |
| RCAGCTGY | chr6 | − | 20985780 | 20985787 | 9.95e-05 | 0.235 | `CCAGCTGC` |
| RCAGCTGY | chr6 | − | 26152426 | 26152433 | 9.95e-05 | 0.235 | `CCAGCTGC` |
| RCAGCTGY | chr6 | − | 26152462 | 26152469 | 9.95e-05 | 0.235 | `CCAGCTGC` |
| RCAGCTGY | chr6 | + | 26307192 | 26307199 | 9.95e-05 | 0.235 | `CCAGCTGC` |
| RCAGCTGY | chr6 | + | 27948009 | 27948016 | 9.95e-05 | 0.235 | `CCAGCTGC` |
| RCAGCTGY | chr6 | + | 27968648 | 27968655 | 9.95e-05 | 0.235 | `CCAGCTGC` |
| RCAGCTGY | chr6 | + | 30793343 | 30793350 | 9.95e-05 | 0.235 | `CCAGCTGC` |
| RCAGCTGY | chr6 | − | 31654385 | 31654392 | 9.95e-05 | 0.235 | `CCAGCTGC` |
| RCAGCTGY | chr6 | − | 36103584 | 36103591 | 9.95e-05 | 0.235 | `CCAGCTGC` |
| RCAGCTGY | chr6 | − | 42822317 | 42822324 | 9.95e-05 | 0.235 | `CCAGCTGC` |
| RCAGCTGY | chr6 | + | 43790835 | 43790842 | 9.95e-05 | 0.235 | `CCAGCTGC` |
| RCAGCTGY | chr6 | + | 106656897 | 106656904 | 9.95e-05 | 0.235 | `TCAGCTGC` |
| RCAGCTGY | chr6 | + | 106718519 | 106718526 | 9.95e-05 | 0.235 | `CCAGCTGC` |
| RCAGCTGY | chr6 | + | 111912053 | 111912060 | 9.95e-05 | 0.235 | `TCAGCTGC` |
| RCAGCTGY | chr6 | − | 131991377 | 131991384 | 9.95e-05 | 0.235 | `TCAGCTGC` |
| RCAGCTGY | chr6 | − | 143813457 | 143813464 | 9.95e-05 | 0.235 | `CCAGCTGC` |
| RCAGCTGY | chr6 | − | 157061317 | 157061324 | 9.95e-05 | 0.235 | `CCAGCTGC` |
| RCAGCTGY | chr7 | − | 999379 | 999386 | 9.95e-05 | 0.235 | `CCAGCTGC` |
| RCAGCTGY | chr7 | + | 25954028 | 25954035 | 9.95e-05 | 0.235 | `TCAGCTGC` |
| RCAGCTGY | chr7 | + | 35736816 | 35736823 | 9.95e-05 | 0.235 | `TCAGCTGC` |
| RCAGCTGY | chr7 | + | 100515343 | 100515350 | 9.95e-05 | 0.235 | `CCAGCTGC` |
| RCAGCTGY | chr7 | + | 126128496 | 126128503 | 9.95e-05 | 0.235 | `TCAGCTGC` |
| RCAGCTGY | chr7 | + | 128362236 | 128362243 | 9.95e-05 | 0.235 | `TCAGCTGC` |
| RCAGCTGY | chr7 | + | 130459746 | 130459753 | 9.95e-05 | 0.235 | `CCAGCTGC` |
| RCAGCTGY | chr8 | − | 8228651 | 8228658 | 9.95e-05 | 0.235 | `CCAGCTGC` |
| RCAGCTGY | chr8 | − | 10822423 | 10822430 | 9.95e-05 | 0.235 | `CCAGCTGC` |
| RCAGCTGY | chr8 | + | 38934311 | 38934318 | 9.95e-05 | 0.235 | `TCAGCTGC` |
| RCAGCTGY | chr8 | + | 38934469 | 38934476 | 9.95e-05 | 0.235 | `CCAGCTGC` |
| RCAGCTGY | chr8 | + | 41600134 | 41600141 | 9.95e-05 | 0.235 | `TCAGCTGC` |
| RCAGCTGY | chr8 | − | 61985380 | 61985387 | 9.95e-05 | 0.235 | `CCAGCTGC` |
| RCAGCTGY | chr8 | − | 101557609 | 101557616 | 9.95e-05 | 0.235 | `CCAGCTGC` |
| RCAGCTGY | chr8 | + | 125003645 | 125003652 | 9.95e-05 | 0.235 | `TCAGCTGC` |
| RCAGCTGY | chr8 | + | 125719026 | 125719033 | 9.95e-05 | 0.235 | `CCAGCTGC` |
| RCAGCTGY | chr8 | + | 129395293 | 129395300 | 9.95e-05 | 0.235 | `TCAGCTGC` |
| RCAGCTGY | chr9 | + | 17853465 | 17853472 | 9.95e-05 | 0.235 | `TCAGCTGC` |
| RCAGCTGY | chr9 | + | 113814576 | 113814583 | 9.95e-05 | 0.235 | `CCAGCTGC` |
| RCAGCTGY | chr9 | − | 114585827 | 114585834 | 9.95e-05 | 0.235 | `CCAGCTGC` |
| RCAGCTGY | chr9 | + | 115325660 | 115325667 | 9.95e-05 | 0.235 | `TCAGCTGC` |
| RCAGCTGY | chr9 | − | 125939801 | 125939808 | 9.95e-05 | 0.235 | `CCAGCTGC` |
| RCAGCTGY | chr9 | − | 125939834 | 125939841 | 9.95e-05 | 0.235 | `CCAGCTGC` |
| RCAGCTGY | chr9 | − | 129883373 | 129883380 | 9.95e-05 | 0.235 | `CCAGCTGC` |
| RCAGCTGY | chr9 | − | 130459211 | 130459218 | 9.95e-05 | 0.235 | `TCAGCTGC` |
| RCAGCTGY | chr9 | − | 131687567 | 131687574 | 9.95e-05 | 0.235 | `TCAGCTGC` |
| RCAGCTGY | chrX | − | 7012253 | 7012260 | 9.95e-05 | 0.235 | `CCAGCTGC` |
| RCAGCTGY | chrX | − | 70759225 | 70759232 | 9.95e-05 | 0.235 | `CCAGCTGC` |
| RCAGCTGY | chrX | − | 119643354 | 119643361 | 9.95e-05 | 0.235 | `CCAGCTGC` |
| RCAGCTGY | chr10 | − | 70495387 | 70495394 | 9.95e-05 | 0.235 | `CCAGCTGC` |
| RCAGCTGY | chr10 | − | 73705277 | 73705284 | 9.95e-05 | 0.235 | `CCAGCTGC` |
| RCAGCTGY | chr10 | − | 73765613 | 73765620 | 9.95e-05 | 0.235 | `CCAGCTGC` |
| RCAGCTGY | chr10 | − | 82002617 | 82002624 | 9.95e-05 | 0.235 | `CCAGCTGC` |
| RCAGCTGY | chr10 | + | 97506764 | 97506771 | 9.95e-05 | 0.235 | `CCAGCTGC` |
| RCAGCTGY | chr10 | + | 103608909 | 103608916 | 9.95e-05 | 0.235 | `TCAGCTGC` |
| RCAGCTGY | chr10 | − | 104411116 | 104411123 | 9.95e-05 | 0.235 | `CCAGCTGC` |
| RCAGCTGY | chr10 | − | 104411180 | 104411187 | 9.95e-05 | 0.235 | `CCAGCTGC` |
| RCAGCTGY | chr10 | + | 112106478 | 112106485 | 9.95e-05 | 0.235 | `CCAGCTGC` |
| RCAGCTGY | chr10 | + | 126398173 | 126398180 | 9.95e-05 | 0.235 | `CCAGCTGC` |
| RCAGCTGY | chr11 | + | 62081448 | 62081455 | 9.95e-05 | 0.235 | `TCAGCTGC` |
| RCAGCTGY | chr11 | − | 62378954 | 62378961 | 9.95e-05 | 0.235 | `TCAGCTGC` |
| RCAGCTGY | chr11 | + | 63750992 | 63750999 | 9.95e-05 | 0.235 | `TCAGCTGC` |
| RCAGCTGY | chr11 | − | 64620678 | 64620685 | 9.95e-05 | 0.235 | `TCAGCTGC` |
| RCAGCTGY | chr11 | + | 64620977 | 64620984 | 9.95e-05 | 0.235 | `TCAGCTGC` |
| RCAGCTGY | chr11 | + | 64621042 | 64621049 | 9.95e-05 | 0.235 | `TCAGCTGC` |
| RCAGCTGY | chr11 | + | 65021488 | 65021495 | 9.95e-05 | 0.235 | `CCAGCTGC` |
| RCAGCTGY | chr11 | + | 65076514 | 65076521 | 9.95e-05 | 0.235 | `CCAGCTGC` |
| RCAGCTGY | chr11 | − | 67790768 | 67790775 | 9.95e-05 | 0.235 | `CCAGCTGC` |
| RCAGCTGY | chr11 | − | 74489315 | 74489322 | 9.95e-05 | 0.235 | `TCAGCTGC` |
| RCAGCTGY | chr11 | − | 95588522 | 95588529 | 9.95e-05 | 0.235 | `CCAGCTGC` |
| RCAGCTGY | chr11 | + | 118068453 | 118068460 | 9.95e-05 | 0.235 | `TCAGCTGC` |
| RCAGCTGY | chr11 | + | 124445912 | 124445919 | 9.95e-05 | 0.235 | `CCAGCTGC` |
| RCAGCTGY | chr12 | + | 1533923 | 1533930 | 9.95e-05 | 0.235 | `CCAGCTGC` |
| RCAGCTGY | chr12 | − | 6941225 | 6941232 | 9.95e-05 | 0.235 | `CCAGCTGC` |
| RCAGCTGY | chr12 | + | 22588973 | 22588980 | 9.95e-05 | 0.235 | `CCAGCTGC` |
| RCAGCTGY | chr12 | + | 46550457 | 46550464 | 9.95e-05 | 0.235 | `TCAGCTGC` |
| RCAGCTGY | chr12 | + | 46563518 | 46563525 | 9.95e-05 | 0.235 | `CCAGCTGC` |
| RCAGCTGY | chr12 | − | 46563563 | 46563570 | 9.95e-05 | 0.235 | `CCAGCTGC` |
| RCAGCTGY | chr12 | − | 74640358 | 74640365 | 9.95e-05 | 0.235 | `CCAGCTGC` |
| RCAGCTGY | chr12 | + | 91063683 | 91063690 | 9.95e-05 | 0.235 | `CCAGCTGC` |
| RCAGCTGY | chr12 | − | 91321912 | 91321919 | 9.95e-05 | 0.235 | `TCAGCTGC` |
| RCAGCTGY | chr12 | − | 93480243 | 93480250 | 9.95e-05 | 0.235 | `CCAGCTGC` |
| RCAGCTGY | chr12 | + | 99103807 | 99103814 | 9.95e-05 | 0.235 | `TCAGCTGC` |
| RCAGCTGY | chr12 | − | 105275829 | 105275836 | 9.95e-05 | 0.235 | `CCAGCTGC` |
| RCAGCTGY | chr12 | − | 122134303 | 122134310 | 9.95e-05 | 0.235 | `CCAGCTGC` |
| RCAGCTGY | chr12 | − | 122134604 | 122134611 | 9.95e-05 | 0.235 | `CCAGCTGC` |
| RCAGCTGY | chr12 | − | 123967937 | 123967944 | 9.95e-05 | 0.235 | `CCAGCTGC` |
| RCAGCTGY | chr13 | − | 35519041 | 35519048 | 9.95e-05 | 0.235 | `TCAGCTGC` |
| RCAGCTGY | chr13 | − | 39917756 | 39917763 | 9.95e-05 | 0.235 | `CCAGCTGC` |
| RCAGCTGY | chr13 | + | 39917878 | 39917885 | 9.95e-05 | 0.235 | `CCAGCTGC` |
| RCAGCTGY | chr13 | + | 47509945 | 47509952 | 9.95e-05 | 0.235 | `CCAGCTGC` |
| RCAGCTGY | chr14 | − | 21715717 | 21715724 | 9.95e-05 | 0.235 | `CCAGCTGC` |
| RCAGCTGY | chr14 | + | 64839093 | 64839100 | 9.95e-05 | 0.235 | `TCAGCTGC` |
| RCAGCTGY | chr14 | + | 64840418 | 64840425 | 9.95e-05 | 0.235 | `CCAGCTGC` |
| RCAGCTGY | chr14 | + | 69440780 | 69440787 | 9.95e-05 | 0.235 | `CCAGCTGC` |
| RCAGCTGY | chr14 | − | 75016717 | 75016724 | 9.95e-05 | 0.235 | `TCAGCTGC` |
| RCAGCTGY | chr14 | − | 94801781 | 94801788 | 9.95e-05 | 0.235 | `CCAGCTGC` |
| RCAGCTGY | chr14 | − | 95032001 | 95032008 | 9.95e-05 | 0.235 | `CCAGCTGC` |
| RCAGCTGY | chr14 | + | 104463956 | 104463963 | 9.95e-05 | 0.235 | `TCAGCTGC` |
| RCAGCTGY | chr14 | + | 104585162 | 104585169 | 9.95e-05 | 0.235 | `CCAGCTGC` |
| RCAGCTGY | chr15 | + | 19249138 | 19249145 | 9.95e-05 | 0.235 | `CCAGCTGC` |
| RCAGCTGY | chr15 | − | 29310139 | 29310146 | 9.95e-05 | 0.235 | `TCAGCTGC` |
| RCAGCTGY | chr15 | + | 29310348 | 29310355 | 9.95e-05 | 0.235 | `TCAGCTGC` |
| RCAGCTGY | chr15 | + | 29310363 | 29310370 | 9.95e-05 | 0.235 | `TCAGCTGC` |
| RCAGCTGY | chr15 | − | 29343872 | 29343879 | 9.95e-05 | 0.235 | `CCAGCTGC` |
| RCAGCTGY | chr15 | − | 29346070 | 29346077 | 9.95e-05 | 0.235 | `CCAGCTGC` |
| RCAGCTGY | chr15 | − | 39196234 | 39196241 | 9.95e-05 | 0.235 | `CCAGCTGC` |
| RCAGCTGY | chr15 | − | 57623266 | 57623273 | 9.95e-05 | 0.235 | `TCAGCTGC` |
| RCAGCTGY | chr15 | + | 61583971 | 61583978 | 9.95e-05 | 0.235 | `CCAGCTGC` |
| RCAGCTGY | chr15 | + | 61757278 | 61757285 | 9.95e-05 | 0.235 | `TCAGCTGC` |
| RCAGCTGY | chr15 | + | 62224040 | 62224047 | 9.95e-05 | 0.235 | `CCAGCTGC` |
| RCAGCTGY | chr15 | − | 68584091 | 68584098 | 9.95e-05 | 0.235 | `TCAGCTGC` |
| RCAGCTGY | chr15 | − | 72482538 | 72482545 | 9.95e-05 | 0.235 | `CCAGCTGC` |
| RCAGCTGY | chr15 | − | 72879578 | 72879585 | 9.95e-05 | 0.235 | `TCAGCTGC` |
| RCAGCTGY | chr15 | − | 73123013 | 73123020 | 9.95e-05 | 0.235 | `CCAGCTGC` |
| RCAGCTGY | chr15 | − | 73447574 | 73447581 | 9.95e-05 | 0.235 | `CCAGCTGC` |
| RCAGCTGY | chr15 | + | 91184176 | 91184183 | 9.95e-05 | 0.235 | `CCAGCTGC` |
| RCAGCTGY | chr16 | − | 11363603 | 11363610 | 9.95e-05 | 0.235 | `CCAGCTGC` |
| RCAGCTGY | chr16 | − | 11364685 | 11364692 | 9.95e-05 | 0.235 | `CCAGCTGC` |
| RCAGCTGY | chr16 | + | 17136021 | 17136028 | 9.95e-05 | 0.235 | `CCAGCTGC` |
| RCAGCTGY | chr16 | + | 19474446 | 19474453 | 9.95e-05 | 0.235 | `CCAGCTGC` |
| RCAGCTGY | chr16 | + | 27320936 | 27320943 | 9.95e-05 | 0.235 | `CCAGCTGC` |
| RCAGCTGY | chr16 | + | 27321235 | 27321242 | 9.95e-05 | 0.235 | `CCAGCTGC` |
| RCAGCTGY | chr16 | − | 51690471 | 51690478 | 9.95e-05 | 0.235 | `TCAGCTGC` |
| RCAGCTGY | chr16 | − | 51690565 | 51690572 | 9.95e-05 | 0.235 | `CCAGCTGC` |
| RCAGCTGY | chr16 | − | 70408071 | 70408078 | 9.95e-05 | 0.235 | `TCAGCTGC` |
| RCAGCTGY | chr16 | − | 86542429 | 86542436 | 9.95e-05 | 0.235 | `CCAGCTGC` |
| RCAGCTGY | chr16 | + | 87562373 | 87562380 | 9.95e-05 | 0.235 | `TCAGCTGC` |
| RCAGCTGY | chr17 | − | 2665051 | 2665058 | 9.95e-05 | 0.235 | `TCAGCTGC` |
| RCAGCTGY | chr17 | − | 2665231 | 2665238 | 9.95e-05 | 0.235 | `CCAGCTGC` |
| RCAGCTGY | chr17 | + | 4784323 | 4784330 | 9.95e-05 | 0.235 | `TCAGCTGC` |
| RCAGCTGY | chr17 | − | 23409360 | 23409367 | 9.95e-05 | 0.235 | `TCAGCTGC` |
| RCAGCTGY | chr17 | + | 33853348 | 33853355 | 9.95e-05 | 0.235 | `CCAGCTGC` |
| RCAGCTGY | chr17 | − | 35273987 | 35273994 | 9.95e-05 | 0.235 | `CCAGCTGC` |
| RCAGCTGY | chr17 | − | 35963851 | 35963858 | 9.95e-05 | 0.235 | `CCAGCTGC` |
| RCAGCTGY | chr17 | − | 38114339 | 38114346 | 9.95e-05 | 0.235 | `CCAGCTGC` |
| RCAGCTGY | chr17 | − | 45580853 | 45580860 | 9.95e-05 | 0.235 | `CCAGCTGC` |
| RCAGCTGY | chr17 | + | 45584521 | 45584528 | 9.95e-05 | 0.235 | `CCAGCTGC` |
| RCAGCTGY | chr17 | − | 46586011 | 46586018 | 9.95e-05 | 0.235 | `CCAGCTGC` |
| RCAGCTGY | chr17 | + | 71779549 | 71779556 | 9.95e-05 | 0.235 | `TCAGCTGC` |
| RCAGCTGY | chr17 | − | 72614752 | 72614759 | 9.95e-05 | 0.235 | `TCAGCTGC` |
| RCAGCTGY | chr17 | − | 73682221 | 73682228 | 9.95e-05 | 0.235 | `TCAGCTGC` |
| RCAGCTGY | chr18 | − | 3237967 | 3237974 | 9.95e-05 | 0.235 | `CCAGCTGC` |
| RCAGCTGY | chr18 | − | 10473521 | 10473528 | 9.95e-05 | 0.235 | `CCAGCTGC` |
| RCAGCTGY | chr18 | − | 19076279 | 19076286 | 9.95e-05 | 0.235 | `CCAGCTGC` |
| RCAGCTGY | chr18 | + | 47227690 | 47227697 | 9.95e-05 | 0.235 | `TCAGCTGC` |
| RCAGCTGY | chr18 | + | 58973707 | 58973714 | 9.95e-05 | 0.235 | `CCAGCTGC` |
| RCAGCTGY | chr18 | − | 59136522 | 59136529 | 9.95e-05 | 0.235 | `CCAGCTGC` |
| RCAGCTGY | chr19 | − | 1334303 | 1334310 | 9.95e-05 | 0.235 | `CCAGCTGC` |
| RCAGCTGY | chr19 | + | 12764593 | 12764600 | 9.95e-05 | 0.235 | `TCAGCTGC` |
| RCAGCTGY | chr19 | − | 12871662 | 12871669 | 9.95e-05 | 0.235 | `TCAGCTGC` |
| RCAGCTGY | chr19 | − | 13139513 | 13139520 | 9.95e-05 | 0.235 | `CCAGCTGC` |
| RCAGCTGY | chr19 | + | 14353166 | 14353173 | 9.95e-05 | 0.235 | `CCAGCTGC` |
| RCAGCTGY | chr19 | − | 17391579 | 17391586 | 9.95e-05 | 0.235 | `TCAGCTGC` |
| RCAGCTGY | chr19 | + | 38359677 | 38359684 | 9.95e-05 | 0.235 | `TCAGCTGC` |
| RCAGCTGY | chr19 | − | 44590163 | 44590170 | 9.95e-05 | 0.235 | `CCAGCTGC` |
| RCAGCTGY | chr19 | − | 44628082 | 44628089 | 9.95e-05 | 0.235 | `CCAGCTGC` |
| RCAGCTGY | chr19 | + | 55091331 | 55091338 | 9.95e-05 | 0.235 | `CCAGCTGC` |
| RCAGCTGY | chr19 | + | 59404225 | 59404232 | 9.95e-05 | 0.235 | `CCAGCTGC` |
| RCAGCTGY | chr19 | + | 63598815 | 63598822 | 9.95e-05 | 0.235 | `CCAGCTGC` |
| RCAGCTGY | chr20 | − | 42335474 | 42335481 | 9.95e-05 | 0.235 | `CCAGCTGC` |
| RCAGCTGY | chr20 | − | 42708479 | 42708486 | 9.95e-05 | 0.235 | `CCAGCTGC` |
| RCAGCTGY | chr20 | − | 42708536 | 42708543 | 9.95e-05 | 0.235 | `CCAGCTGC` |
| RCAGCTGY | chr20 | − | 45477450 | 45477457 | 9.95e-05 | 0.235 | `CCAGCTGC` |
| RCAGCTGY | chr21 | + | 41739378 | 41739385 | 9.95e-05 | 0.235 | `CCAGCTGC` |
| RCAGCTGY | chr22 | + | 16119153 | 16119160 | 9.95e-05 | 0.235 | `CCAGCTGC` |
| RCAGCTGY | chr22 | − | 17799912 | 17799919 | 9.95e-05 | 0.235 | `TCAGCTGC` |
| RCAGCTGY | chr22 | − | 20715154 | 20715161 | 9.95e-05 | 0.235 | `CCAGCTGC` |
| RCAGCTGY | chr22 | − | 21604007 | 21604014 | 9.95e-05 | 0.235 | `CCAGCTGC` |
| RCAGCTGY | chr22 | − | 21604197 | 21604204 | 9.95e-05 | 0.235 | `CCAGCTGC` |
| RCAGCTGY | chr22 | − | 21607837 | 21607844 | 9.95e-05 | 0.235 | `CCAGCTGC` |
| RCAGCTGY | chr22 | + | 25336412 | 25336419 | 9.95e-05 | 0.235 | `CCAGCTGC` |
| RCAGCTGY | chr22 | + | 35588575 | 35588582 | 9.95e-05 | 0.235 | `TCAGCTGC` |
| RCAGCTGY | chr22 | + | 36334615 | 36334622 | 9.95e-05 | 0.235 | `CCAGCTGC` |
| RCAGCTGY | chr22 | − | 38037797 | 38037804 | 9.95e-05 | 0.235 | `CCAGCTGC` |
| RCAGCTGY | chr22 | + | 39140957 | 39140964 | 9.95e-05 | 0.235 | `CCAGCTGC` |
| RCAGCTGY | chr22 | − | 40558654 | 40558661 | 9.95e-05 | 0.235 | `CCAGCTGC` |
| RCAGCTGY | chr22 | + | 40662558 | 40662565 | 9.95e-05 | 0.235 | `CCAGCTGC` |
| RCAGCTGY | chr22 | + | 41341512 | 41341519 | 9.95e-05 | 0.235 | `CCAGCTGC` |
| RCAGCTGY | chr22 | − | 41993821 | 41993828 | 9.95e-05 | 0.235 | `CCAGCTGC` |
| RCAGCTGY | chr22 | + | 46770908 | 46770915 | 9.95e-05 | 0.235 | `CCAGCTGC` |
| RCAGCTGY | chr22 | − | 49313988 | 49313995 | 9.95e-05 | 0.235 | `CCAGCTGC` |
| RCAGCTGY | chr17\_random | + | 88131 | 88138 | 9.95e-05 | 0.235 | `TCAGCTGC` |

---

**DEBUGGING INFORMATION**


---

Command line:

```
/ebi/sw/MEME/VM-cluster410/meme-versions/4.10.0/bin/fimo --parse-genomic-coord --verbosity 1 --oc fimo_out_5 --bgfile ./background --motif RCAGCTGY dreme_out/dreme.xml ./Supplementary_Table_1.500bp.fa
```

Settings:

```
|  |  |  |
| --- | --- | --- |
| output directory = fimo_out_5 | MEME file name = dreme_out/dreme.xml | sequence file name = ./Supplementary_Table_1.500bp.fa |
| background file name = ./background | allow clobber = true | compute q-values = true |
| parse genomic coord. = true | text only = false | scan both strands = true |
| max sequence length = 250000000 | output threshold = 0.0001 | threshold type = p-value |
| max stored scores = 100000 | pseudocount = 0.1 | verbosity = 1 |
| selected motif = RCAGCTGY |  |  |
```

This information can be useful in the event you wish to report a
problem with the FIMO software.

---

**Go to top**
