# Supplementary material for: The MEME Suite
Source: Nucleic Acids Res. 2015 May 7;43(Web Server issue):W39–49. doi: 10.1093/nar/gkv416 (PMC4489269; doi:10.1093/nar/gkv416)
Supplement: SUPPLEMENTARY DATA [file supp_gkv416_nar-00283-web-b-2015-File005.zip › case4/meme-chip/fimo_out_6/fimo.html]

FIMO Results


---

|  |  |  |
| --- | --- | --- |
| **Database and Motifs** | **High-scoring Motif Occurrences** | **Debugging Information** |

  
  

---

**FIMO - Motif search tool**


---

FIMO version 4.10.0,
(Release date: Wed May 21 10:35:36 2014 +1000)

For further information on how to interpret these results
or to get a copy of the FIMO software please access
http://meme.nbcr.net

If you use FIMO in your research, please cite the following paper:  
Charles E. Grant, Timothy L. Bailey, and William Stafford Noble,
"FIMO: Scanning for occurrences of a given motif",
*Bioinformatics*, **27**(7):1017-1018, 2011.
[full text]

---

**DATABASE AND MOTIFS**


---

DATABASE
./Supplementary\_Table\_1.500bp.fa  
Database contains
2776
sequences,
1388000
residues

MOTIFS
db/JASPAR\_CORE\_2014\_vertebrates.meme
(nucleotide)

| MOTIF | WIDTH | BEST POSSIBLE MATCH |
| --- | --- | --- |
| MA0004.1 | 6 | CACGTG |
| MA0006.1 | 6 | TGCGTG |
| MA0009.1 | 11 | CTAGGTGTGAA |
| MA0017.1 | 14 | TGACCTTTGAACCT |
| MA0019.1 | 12 | AGATGCAATCCC |
| MA0025.1 | 11 | TTATGTAACGT |
| MA0027.1 | 11 | AAGTAGTGGCC |
| MA0028.1 | 10 | GAGCCGGAAG |
| MA0029.1 | 14 | AAGATAAGATAAGA |
| MA0030.1 | 14 | CAAACGTAAACAAT |
| MA0031.1 | 8 | GTAAACAT |
| MA0032.1 | 8 | GGTAAGTA |
| MA0033.1 | 8 | TATACATA |
| MA0038.1 | 10 | CAAATCACTG |
| MA0040.1 | 11 | TATTGTTTATT |
| MA0041.1 | 12 | GAATGTTTGTTT |
| MA0042.1 | 12 | GGATGTTTGTTT |
| MA0043.1 | 12 | GGTTACGCAATC |
| MA0046.1 | 14 | GGTTAATAATTACC |
| MA0048.1 | 12 | GCGCAGCTGCGT |
| MA0051.1 | 18 | GGAAAGCGAAAGCAAAAC |
| MA0056.1 | 6 | TGGGGA |
| MA0057.1 | 10 | GGAGGGGGAA |
| MA0059.1 | 11 | GAGCACGTGGT |
| MA0063.1 | 7 | TTAATTG |
| MA0066.1 | 20 | GTAGGTCACGGTGACCTACT |
| MA0067.1 | 8 | AGTCACGG |
| MA0068.1 | 30 | GAAAAATTTCCCATACTCCACTCCCCCCCC |
| MA0069.1 | 14 | TTCACGCATGAGTT |
| MA0070.1 | 12 | CCATCAATCAAA |
| MA0071.1 | 10 | ATCAAGGTCA |
| MA0072.1 | 14 | TATAAGTAGGTCAA |
| MA0073.1 | 20 | CCCCAAACCACCCCCCCCCC |
| MA0074.1 | 15 | GGGTCATCGGGTTCA |
| MA0075.1 | 5 | AATTA |
| MA0077.1 | 9 | CCATTGTTC |
| MA0078.1 | 9 | CTCATTGTC |
| MA0081.1 | 7 | AGAGGAA |
| MA0084.1 | 9 | GTAAACAAT |
| MA0087.1 | 7 | ATTGTTT |
| MA0088.1 | 20 | GATTTCCCATCATGCCTTGC |
| MA0089.1 | 6 | CATGAC |
| MA0090.1 | 12 | CACATTCCTCCG |
| MA0091.1 | 12 | CGACCATCTGTT |
| MA0092.1 | 10 | GGTCTGGCAT |
| MA0101.1 | 10 | GGGGATTTCC |
| MA0107.1 | 10 | GGGAATTTCC |
| MA0108.2 | 15 | GTATAAAAGGCGGGG |
| MA0109.1 | 10 | AACCTTATAT |
| MA0111.1 | 11 | AGGGTAACAGC |
| MA0115.1 | 17 | AAAGGTCAAAGGTCAAC |
| MA0116.1 | 15 | GGCACCCAGGGGTGC |
| MA0117.1 | 8 | GCTGACGG |
| MA0119.1 | 14 | TGGCACCATGCCAA |
| MA0122.1 | 9 | TTAAGTGGA |
| MA0124.1 | 7 | ATACTTA |
| MA0125.1 | 8 | TAATTGGT |
| MA0130.1 | 6 | ATCCAC |
| MA0131.1 | 10 | TAACGTCCGC |
| MA0132.1 | 6 | CTAATT |
| MA0133.1 | 7 | ACAACAC |
| MA0135.1 | 13 | AAATTAATTAATC |
| MA0136.1 | 9 | TACTTCCTT |
| MA0139.1 | 19 | TGGCCACCAGGGGGCGCTA |
| MA0142.1 | 15 | CTTTGTTATGCAAAT |
| MA0149.1 | 18 | GGAAGGAAGGAAGGAAGG |
| MA0062.2 | 11 | CCGGAAGTGGC |
| MA0039.2 | 10 | TGGGTGGGGC |
| MA0138.2 | 21 | TTCAGCACCATGGACAGCGCC |
| MA0002.2 | 11 | GTCTGTGGTTT |
| MA0047.2 | 12 | TGTTTACTTAGG |
| MA0112.2 | 20 | GGCCCAGGTCACCCTGACCT |
| MA0065.2 | 15 | GTAGGGCAAAGGTCA |
| MA0151.1 | 6 | ATTAAA |
| MA0152.1 | 7 | TTTTCCA |
| MA0153.1 | 12 | TTAATATTTAAC |
| MA0155.1 | 12 | TGTCAGGGGGCG |
| MA0156.1 | 8 | CAGGAAAT |
| MA0157.1 | 8 | TGTAAACA |
| MA0158.1 | 8 | CACTAATT |
| MA0159.1 | 17 | AGGTCACGGAGAGGTCA |
| MA0160.1 | 8 | AAGGTCAC |
| MA0161.1 | 6 | TTGGCA |
| MA0163.1 | 14 | GGGGCCCAAGGGGG |
| MA0164.1 | 7 | CAAGCTT |
| MA0018.2 | 8 | TGACGTCA |
| MA0099.2 | 7 | TGACTCA |
| MA0259.1 | 8 | GGACGTGC |
| MA0442.1 | 6 | CTTTGT |
| MA0141.2 | 12 | AGGTCAAGGTCA |
| MA0145.2 | 14 | CCAGTTCAAACCAG |
| MA0146.2 | 14 | GGGGCCGAGGCCTG |
| MA0461.1 | 8 | CAGATGGC |
| MA0462.1 | 11 | GAAATGACTCA |
| MA0463.1 | 14 | TTTCCTAGAAAGCA |
| MA0464.1 | 11 | CTCACGTGCAC |
| MA0465.1 | 11 | AAGCCATAAAA |
| MA0466.1 | 11 | TATTGCACAAT |
| MA0467.1 | 11 | AAGAGGATTAG |
| MA0468.1 | 11 | TAATTTAATCA |
| MA0469.1 | 15 | CTCCCGCCCCCACTC |
| MA0470.1 | 11 | GGGCGGGAAGG |
| MA0471.1 | 11 | GGGCGGGAAGG |
| MA0472.1 | 15 | CCCCCGCCCACGCAC |
| MA0473.1 | 13 | GAACCAGGAAGTG |
| MA0474.1 | 11 | ACAGGAAGTGG |
| MA0475.1 | 11 | ACAGGAAGTGG |
| MA0476.1 | 11 | TGTGACTCATT |
| MA0477.1 | 11 | GGTGACTCATG |
| MA0478.1 | 11 | GGATGACTCAT |
| MA0479.1 | 11 | TCCAATCCACA |
| MA0480.1 | 11 | TCCTGTTTACA |
| MA0481.1 | 15 | CAAAAGTAAACAAAG |
| MA0482.1 | 11 | TCTTATCTCCC |
| MA0483.1 | 11 | AAATCACAGCA |
| MA0484.1 | 15 | AGAGTCCAAAGTCCA |
| MA0485.1 | 13 | GGCCATAAATCAC |
| MA0486.1 | 15 | CTTCTAGAAGGTTCT |
| MA0488.1 | 13 | AAGATGATGTCAT |
| MA0489.1 | 14 | AGGAGATGACTCAT |
| MA0490.1 | 11 | GGATGACTCAT |
| MA0491.1 | 11 | GGTGACTCATC |
| MA0492.1 | 15 | AAAGATGATGTCATC |
| MA0493.1 | 11 | GGCCACACCCA |
| MA0494.1 | 19 | TGACCTAAAGTAACCTCTG |
| MA0495.1 | 18 | GCTGAGTCAGCAATTTTT |
| MA0496.1 | 15 | CTGAGTCAGCAATTT |
| MA0497.1 | 15 | ATGCTAAAAATAGAA |
| MA0498.1 | 15 | AGCTGTCACTCACCT |
| MA0499.1 | 13 | TGCAGCTGTCCCT |
| MA0500.1 | 11 | GACAGCTGCAG |
| MA0501.1 | 15 | ATGACTCAGCAATTT |
| MA0502.1 | 15 | AAATGGACCAATCAG |
| MA0503.1 | 11 | AGCCACTCAAG |
| MA0504.1 | 15 | AGGGGTCAGAGGTCA |
| MA0505.1 | 15 | AAGTTCAAGGTCAGC |
| MA0506.1 | 11 | GCGCCTGCGCA |
| MA0507.1 | 13 | TTCATTTGCATAT |
| MA0508.1 | 15 | AGAAAGTGAAAGTGA |
| MA0509.1 | 14 | GTTGCCATGGCAAC |
| MA0510.1 | 15 | CTCCCTGGCAACAGC |
| MA0511.1 | 15 | GGGGTTTGTGGTTTG |
| MA0512.1 | 11 | CAAAGGTCAGA |
| MA0513.1 | 13 | CTGTCTGTCACCT |
| MA0514.1 | 10 | CCTTTGTTTT |
| MA0515.1 | 10 | CCATTGTTTT |
| MA0516.1 | 15 | GCCCCGCCCCCTCCC |
| MA0517.1 | 15 | TCAGTTTCATTTTCC |
| MA0518.1 | 14 | TTTCCAGGAAATGG |
| MA0519.1 | 11 | ATTTCCAAGAA |
| MA0520.1 | 15 | CATTTCCTGAGAAAT |
| MA0521.1 | 11 | AACAGCTGCAG |
| MA0522.1 | 11 | CACAGCTGCAG |
| MA0523.1 | 14 | AAAGATCAAAGGAA |
| MA0524.1 | 15 | CATGGCCCCAGGGCA |
| MA0525.1 | 20 | AGACATGCCCAGACATGCCC |
| MA0526.1 | 11 | GTCATGTGACC |
| MA0527.1 | 15 | CTCTCGCGAGATCTG |
| MA0528.1 | 21 | GGAGGAGGAGGGGGAGGAGGA |
| MA0007.2 | 15 | AAGAACAGAATGTTC |
| MA0102.3 | 11 | ATTGCACAATA |
| MA0024.2 | 11 | CGGGCGGGAGG |
| MA0154.2 | 11 | GTCCCCAGGGA |
| MA0162.2 | 14 | CCCCCGCCCCCGCC |
| MA0076.2 | 11 | CCACTTCCGGC |
| MA0258.2 | 15 | AGGTCACCCTGACCT |
| MA0098.2 | 15 | CCCACTTCCTGTCTC |
| MA0148.3 | 15 | TCCATGTTTACTTTG |
| MA0035.3 | 11 | TTCTTATCTGT |
| MA0036.2 | 14 | AGATTCTTATCTGT |
| MA0037.2 | 8 | AGATAAGA |
| MA0114.2 | 15 | CTGGACTTTGGACTC |
| MA0050.2 | 21 | TTTTACTTTCACTTTCACTTT |
| MA0058.2 | 10 | AAGCACATGG |
| MA0052.2 | 15 | AGCTAAAAATAGCAT |
| MA0100.2 | 10 | CCAACTGCCA |
| MA0147.2 | 10 | CCATGTGCTT |
| MA0104.3 | 8 | GCCACGTG |
| MA0150.2 | 15 | CAGCATGACTCAGCA |
| MA0105.3 | 11 | GGGAATTTCCC |
| MA0060.2 | 18 | AGAGTGCTGATTGGTCCA |
| MA0014.2 | 19 | GAGGGCAGCCAAGCGTGAC |
| MA0080.3 | 15 | AAAAAGAGGAAGTGA |
| MA0143.3 | 8 | CCTTTGTT |
| MA0079.3 | 11 | GCCCCGCCCCC |
| MA0083.2 | 18 | CATGCCCAAATAAGGCAA |
| MA0137.3 | 11 | TTTCCAGGAAA |
| MA0144.2 | 11 | CTTCTGGGAAA |
| MA0140.2 | 18 | CTTATCTGTGAGGAGCAG |
| MA0003.2 | 15 | CATTGCCTCAGGGCA |
| MA0106.2 | 15 | ACATGCCCAGACATG |
| MA0093.2 | 11 | GCCACGTGACC |
| MA0095.2 | 12 | CAAGATGGCGGC |
| MA0103.2 | 9 | CCTCACCTG |
| MA0591.1 | 15 | AGGATGACTCAGCAC |
| MA0592.1 | 11 | CCAAGGTCACA |
| MA0593.1 | 11 | AAGTAAACAAA |
| MA0594.1 | 11 | GCCATAAATCA |
| MA0595.1 | 10 | ATCACCCCAC |
| MA0596.1 | 10 | ATGGGGTGAT |
| MA0597.1 | 9 | CTGCCCGCA |
| MA0598.1 | 8 | CCTTCCTG |
| MA0599.1 | 10 | GCCCCGCCCC |
| MA0600.1 | 19 | GTTGCCATGGCAACCGCGG |
| MA0113.2 | 15 | AGAACAGAATGTTCT |

Random model letter frequencies
(from ./background):
  
A 0.241 C 0.259 G 0.259 T 0.241

---

**SECTION I: HIGH-SCORING MOTIF OCCURRENCES**


---

- There were
  343
  motif occurrences with a
  p-value less than
  0.0001.
- The p-value of a motif occurrence is defined as the
  probability of a random sequence of the same length as the motif
  matching that position of the sequence with as good or better a score.
- The score for the match of a position in a sequence to a motif
  is computed by summing the appropriate entries from each column of
  the position-dependent scoring matrix that represents the motif.
- The q-value of a motif occurrence is defined as the
  false discovery rate if the occurrence is accepted as significant.
- The table is sorted by increasing p-value.

| Motif | Sequence Name | Strand | Start | End | p-value | q-value | Matched Sequence |
| --- | --- | --- | --- | --- | --- | --- | --- |
| MA0071.1 | chr8 | − | 62073472 | 62073481 | 8.78e-07 | 0.52 | `ATCAAGGTCA` |
| MA0071.1 | chr10 | + | 93982891 | 93982900 | 1.76e-06 | 0.52 | `ATCTAGGTCA` |
| MA0071.1 | chr2 | − | 125846210 | 125846219 | 2.63e-06 | 0.52 | `AACAAGGTCA` |
| MA0071.1 | chr3 | − | 178559357 | 178559366 | 2.63e-06 | 0.52 | `AACAAGGTCA` |
| MA0071.1 | chr10 | + | 97587625 | 97587634 | 2.63e-06 | 0.52 | `AACAAGGTCA` |
| MA0071.1 | chr13 | + | 97962908 | 97962917 | 2.63e-06 | 0.52 | `AACAAGGTCA` |
| MA0071.1 | chr17 | + | 25651392 | 25651401 | 2.63e-06 | 0.52 | `AACAAGGTCA` |
| MA0071.1 | chr17 | − | 70246195 | 70246204 | 2.63e-06 | 0.52 | `AACAAGGTCA` |
| MA0071.1 | chr1 | + | 36381743 | 36381752 | 3.45e-06 | 0.52 | `ATAAAGGTCA` |
| MA0071.1 | chr1 | + | 36381752 | 36381761 | 3.45e-06 | 0.52 | `ATAAAGGTCA` |
| MA0071.1 | chr1 | − | 1701887 | 1701896 | 4.33e-06 | 0.52 | `TTCAAGGTCA` |
| MA0071.1 | chr1 | + | 243175478 | 243175487 | 4.33e-06 | 0.52 | `TTCAAGGTCA` |
| MA0071.1 | chr7 | − | 47677036 | 47677045 | 4.33e-06 | 0.52 | `TTCAAGGTCA` |
| MA0071.1 | chr10 | + | 64085392 | 64085401 | 4.33e-06 | 0.52 | `TTCAAGGTCA` |
| MA0071.1 | chr10 | + | 73603914 | 73603923 | 4.33e-06 | 0.52 | `TTCAAGGTCA` |
| MA0071.1 | chr12 | − | 91281272 | 91281281 | 4.33e-06 | 0.52 | `TTCAAGGTCA` |
| MA0071.1 | chr20 | − | 45820995 | 45821004 | 4.33e-06 | 0.52 | `TTCAAGGTCA` |
| MA0071.1 | chrX | + | 46382466 | 46382475 | 5.27e-06 | 0.52 | `ATCGAGGTCA` |
| MA0071.1 | chr16 | + | 18720510 | 18720519 | 5.27e-06 | 0.52 | `ATCGAGGTCA` |
| MA0071.1 | chr19 | − | 62483354 | 62483363 | 5.27e-06 | 0.52 | `ATCGAGGTCA` |
| MA0071.1 | chr18 | − | 4116001 | 4116010 | 6.15e-06 | 0.52 | `AACTAGGTCA` |
| MA0071.1 | chr20 | + | 29759943 | 29759952 | 6.15e-06 | 0.52 | `AACTAGGTCA` |
| MA0071.1 | chr7 | − | 7950972 | 7950981 | 6.97e-06 | 0.52 | `ATATAGGTCA` |
| MA0071.1 | chr9 | + | 4752993 | 4753002 | 7.85e-06 | 0.52 | `ATGAAGGTCA` |
| MA0071.1 | chr10 | − | 90165761 | 90165770 | 7.85e-06 | 0.52 | `ATGAAGGTCA` |
| MA0071.1 | chr16 | + | 11330195 | 11330204 | 7.85e-06 | 0.52 | `ATGAAGGTCA` |
| MA0071.1 | chr6 | − | 80416352 | 80416361 | 8.66e-06 | 0.52 | `AAAAAGGTCA` |
| MA0071.1 | chr7 | + | 23359456 | 23359465 | 8.66e-06 | 0.52 | `AAAAAGGTCA` |
| MA0071.1 | chr7 | + | 28487747 | 28487756 | 8.66e-06 | 0.52 | `AAAAAGGTCA` |
| MA0071.1 | chr14 | + | 63405024 | 63405033 | 8.66e-06 | 0.52 | `AAAAAGGTCA` |
| MA0071.1 | chr19 | − | 2379058 | 2379067 | 8.66e-06 | 0.52 | `AAAAAGGTCA` |
| MA0071.1 | chr7 | + | 114344979 | 114344988 | 9.54e-06 | 0.52 | `TTCTAGGTCA` |
| MA0071.1 | chr12 | − | 123967711 | 123967720 | 9.54e-06 | 0.52 | `TTCTAGGTCA` |
| MA0071.1 | chr17 | + | 46585658 | 46585667 | 9.54e-06 | 0.52 | `TTCTAGGTCA` |
| MA0071.1 | chr19 | + | 2036583 | 2036592 | 9.54e-06 | 0.52 | `TTCTAGGTCA` |
| MA0071.1 | chr6 | + | 86439945 | 86439954 | 1.04e-05 | 0.52 | `TACAAGGTCA` |
| MA0071.1 | chr9 | + | 131689328 | 131689337 | 1.04e-05 | 0.52 | `TACAAGGTCA` |
| MA0071.1 | chr1 | − | 39229126 | 39229135 | 1.3e-05 | 0.52 | `TTAAAGGTCA` |
| MA0071.1 | chr3 | − | 13349042 | 13349051 | 1.3e-05 | 0.52 | `TTAAAGGTCA` |
| MA0071.1 | chr5 | − | 78089928 | 78089937 | 1.3e-05 | 0.52 | `TTAAAGGTCA` |
| MA0071.1 | chr10 | + | 64067473 | 64067482 | 1.3e-05 | 0.52 | `TTAAAGGTCA` |
| MA0071.1 | chr14 | − | 99601832 | 99601841 | 1.3e-05 | 0.52 | `TTAAAGGTCA` |
| MA0071.1 | chr15 | + | 70554395 | 70554404 | 1.3e-05 | 0.52 | `AACGAGGTCA` |
| MA0071.1 | chr20 | + | 36896593 | 36896602 | 1.3e-05 | 0.52 | `TTAAAGGTCA` |
| MA0071.1 | chr3 | − | 25235067 | 25235076 | 1.39e-05 | 0.52 | `ATAGAGGTCA` |
| MA0071.1 | chr7 | + | 92188270 | 92188279 | 1.39e-05 | 0.52 | `ATAGAGGTCA` |
| MA0071.1 | chr8 | − | 125277455 | 125277464 | 1.39e-05 | 0.52 | `ATAGAGGTCA` |
| MA0071.1 | chr18 | − | 17966496 | 17966505 | 1.39e-05 | 0.52 | `ATAGAGGTCA` |
| MA0071.1 | chr19 | − | 57181831 | 57181840 | 1.39e-05 | 0.52 | `ATAGAGGTCA` |
| MA0071.1 | chr7 | − | 20226641 | 20226650 | 1.48e-05 | 0.52 | `ATGTAGGTCA` |
| MA0071.1 | chr13 | − | 35519055 | 35519064 | 1.48e-05 | 0.52 | `ATGTAGGTCA` |
| MA0071.1 | chr9 | + | 92726169 | 92726178 | 1.56e-05 | 0.52 | `AAATAGGTCA` |
| MA0071.1 | chr17 | − | 4789082 | 4789091 | 1.56e-05 | 0.52 | `AAATAGGTCA` |
| MA0071.1 | chr18 | − | 667341 | 667350 | 1.56e-05 | 0.52 | `AAATAGGTCA` |
| MA0071.1 | chr1 | − | 95141292 | 95141301 | 1.64e-05 | 0.52 | `AAGAAGGTCA` |
| MA0071.1 | chr2 | − | 54660642 | 54660651 | 1.64e-05 | 0.52 | `AAGAAGGTCA` |
| MA0071.1 | chrX | − | 7043387 | 7043396 | 1.64e-05 | 0.52 | `AAGAAGGTCA` |
| MA0071.1 | chrX | − | 7043673 | 7043682 | 1.64e-05 | 0.52 | `AAGAAGGTCA` |
| MA0071.1 | chr10 | + | 11327382 | 11327391 | 1.64e-05 | 0.52 | `AAGAAGGTCA` |
| MA0071.1 | chr12 | − | 2335729 | 2335738 | 1.64e-05 | 0.52 | `AAGAAGGTCA` |
| MA0071.1 | chr19 | − | 46508273 | 46508282 | 1.64e-05 | 0.52 | `AAGAAGGTCA` |
| MA0071.1 | chr2 | − | 12114948 | 12114957 | 1.74e-05 | 0.52 | `TTCGAGGTCA` |
| MA0071.1 | chr19 | − | 17097240 | 17097249 | 1.83e-05 | 0.52 | `TACTAGGTCA` |
| MA0071.1 | chr2 | − | 149133349 | 149133358 | 1.92e-05 | 0.52 | `ATCAGGGTCA` |
| MA0071.1 | chr7 | − | 5564534 | 5564543 | 1.92e-05 | 0.52 | `ATCAGGGTCA` |
| MA0071.1 | chr14 | + | 49598357 | 49598366 | 1.92e-05 | 0.52 | `ATCAGGGTCA` |
| MA0071.1 | chr14 | + | 61135695 | 61135704 | 2e-05 | 0.52 | `ATTTAGGTCA` |
| MA0071.1 | chr15 | + | 42808834 | 42808843 | 2e-05 | 0.52 | `ATTTAGGTCA` |
| MA0071.1 | chr1 | − | 165905546 | 165905555 | 2.1e-05 | 0.52 | `ATCCAGGTCA` |
| MA0071.1 | chr3 | + | 45978774 | 45978783 | 2.1e-05 | 0.52 | `ATCCAGGTCA` |
| MA0071.1 | chr4 | − | 185973733 | 185973742 | 2.1e-05 | 0.52 | `ATCCAGGTCA` |
| MA0071.1 | chr6 | + | 143170186 | 143170195 | 2.1e-05 | 0.52 | `ATCCAGGTCA` |
| MA0071.1 | chrX | − | 46373163 | 46373172 | 2.1e-05 | 0.52 | `ATCCAGGTCA` |
| MA0071.1 | chr15 | + | 81298658 | 81298667 | 2.1e-05 | 0.52 | `ATCCAGGTCA` |
| MA0071.1 | chr2 | − | 191537131 | 191537140 | 2.26e-05 | 0.52 | `TTATAGGTCA` |
| MA0071.1 | chr3 | − | 187989740 | 187989749 | 2.26e-05 | 0.52 | `AATAAGGTCA` |
| MA0071.1 | chr6 | + | 26139642 | 26139651 | 2.26e-05 | 0.52 | `AATAAGGTCA` |
| MA0071.1 | chr10 | − | 90165912 | 90165921 | 2.26e-05 | 0.52 | `AATAAGGTCA` |
| MA0071.1 | chr11 | − | 34761663 | 34761672 | 2.26e-05 | 0.52 | `TTATAGGTCA` |
| MA0071.1 | chr13 | − | 45849748 | 45849757 | 2.26e-05 | 0.52 | `AATAAGGTCA` |
| MA0071.1 | chr2 | + | 54650676 | 54650685 | 2.35e-05 | 0.52 | `TTGAAGGTCA` |
| MA0071.1 | chr4 | − | 14467273 | 14467282 | 2.35e-05 | 0.52 | `TTGAAGGTCA` |
| MA0071.1 | chr6 | − | 36828847 | 36828856 | 2.35e-05 | 0.52 | `TTGAAGGTCA` |
| MA0071.1 | chr1 | − | 38713329 | 38713338 | 2.43e-05 | 0.52 | `TAAAAGGTCA` |
| MA0071.1 | chr4 | + | 25694983 | 25694992 | 2.43e-05 | 0.52 | `TAAAAGGTCA` |
| MA0071.1 | chr16 | − | 55663380 | 55663389 | 2.43e-05 | 0.52 | `TAAAAGGTCA` |
| MA0071.1 | chr17 | − | 63077721 | 63077730 | 2.43e-05 | 0.52 | `TAAAAGGTCA` |
| MA0071.1 | chr8 | + | 105750560 | 105750569 | 2.52e-05 | 0.52 | `ATGGAGGTCA` |
| MA0071.1 | chr8 | − | 146074892 | 146074901 | 2.52e-05 | 0.52 | `ATGGAGGTCA` |
| MA0071.1 | chr9 | − | 6403172 | 6403181 | 2.52e-05 | 0.52 | `ATGGAGGTCA` |
| MA0071.1 | chr17 | + | 70803256 | 70803265 | 2.52e-05 | 0.52 | `ATGGAGGTCA` |
| MA0071.1 | chr2 | − | 179103636 | 179103645 | 2.61e-05 | 0.52 | `AAAGAGGTCA` |
| MA0071.1 | chr11 | − | 117786189 | 117786198 | 2.61e-05 | 0.52 | `AAAGAGGTCA` |
| MA0071.1 | chr13 | − | 76218243 | 76218252 | 2.61e-05 | 0.52 | `AAAGAGGTCA` |
| MA0071.1 | chr15 | + | 61582864 | 61582873 | 2.61e-05 | 0.52 | `AAAGAGGTCA` |
| MA0071.1 | chr15 | + | 84036093 | 84036102 | 2.61e-05 | 0.52 | `AAAGAGGTCA` |
| MA0071.1 | chr16 | − | 23745399 | 23745408 | 2.61e-05 | 0.52 | `AAAGAGGTCA` |
| MA0071.1 | chr6 | − | 138312891 | 138312900 | 2.7e-05 | 0.52 | `AAGTAGGTCA` |
| MA0071.1 | chr8 | + | 119361313 | 119361322 | 2.7e-05 | 0.52 | `AAGTAGGTCA` |
| MA0071.1 | chr14 | + | 67747314 | 67747323 | 2.7e-05 | 0.52 | `AAGTAGGTCA` |
| MA0071.1 | chr1 | − | 195123770 | 195123779 | 2.78e-05 | 0.52 | `TTTAAGGTCA` |
| MA0071.1 | chr3 | − | 99724072 | 99724081 | 2.78e-05 | 0.52 | `TTTAAGGTCA` |
| MA0071.1 | chr5 | + | 106759275 | 106759284 | 2.78e-05 | 0.52 | `TTTAAGGTCA` |
| MA0071.1 | chr9 | − | 91231360 | 91231369 | 2.78e-05 | 0.52 | `TTTAAGGTCA` |
| MA0071.1 | chr10 | − | 7553715 | 7553724 | 2.78e-05 | 0.52 | `TTTAAGGTCA` |
| MA0071.1 | chr12 | + | 122170647 | 122170656 | 2.78e-05 | 0.52 | `TTTAAGGTCA` |
| MA0071.1 | chr18 | − | 9056851 | 9056860 | 2.78e-05 | 0.52 | `TTTAAGGTCA` |
| MA0071.1 | chr19 | + | 17367131 | 17367140 | 2.78e-05 | 0.52 | `TTTAAGGTCA` |
| MA0071.1 | chr2 | + | 38715515 | 38715524 | 2.88e-05 | 0.52 | `GTCAAGGTCA` |
| MA0071.1 | chr3 | − | 197293652 | 197293661 | 2.88e-05 | 0.52 | `GTCAAGGTCA` |
| MA0071.1 | chr5 | + | 139029843 | 139029852 | 2.88e-05 | 0.52 | `GTCAAGGTCA` |
| MA0071.1 | chr21 | − | 41739737 | 41739746 | 2.88e-05 | 0.52 | `GTCAAGGTCA` |
| MA0071.1 | chr16 | − | 17226482 | 17226491 | 2.97e-05 | 0.52 | `TACGAGGTCA` |
| MA0071.1 | chr2 | + | 12064865 | 12064874 | 3.06e-05 | 0.52 | `ATTGAGGTCA` |
| MA0071.1 | chr3 | + | 187985992 | 187986001 | 3.06e-05 | 0.52 | `ATTGAGGTCA` |
| MA0071.1 | chr4 | − | 182481474 | 182481483 | 3.06e-05 | 0.52 | `ATTGAGGTCA` |
| MA0071.1 | chr6 | + | 51969577 | 51969586 | 3.06e-05 | 0.52 | `ATTGAGGTCA` |
| MA0071.1 | chr6 | − | 64120743 | 64120752 | 3.06e-05 | 0.52 | `ATTGAGGTCA` |
| MA0071.1 | chr19 | + | 52153994 | 52154003 | 3.06e-05 | 0.52 | `ATTGAGGTCA` |
| MA0071.1 | chr2 | − | 122173805 | 122173814 | 3.15e-05 | 0.52 | `ATCTGGGTCA` |
| MA0071.1 | chr6 | − | 39891369 | 39891378 | 3.15e-05 | 0.52 | `ATCTGGGTCA` |
| MA0071.1 | chr7 | − | 4689438 | 4689447 | 3.15e-05 | 0.52 | `ATCTGGGTCA` |
| MA0071.1 | chr8 | + | 135683137 | 135683146 | 3.15e-05 | 0.52 | `ATCTGGGTCA` |
| MA0071.1 | chr11 | + | 63794118 | 63794127 | 3.15e-05 | 0.52 | `ATCTGGGTCA` |
| MA0071.1 | chr11 | − | 65112685 | 65112694 | 3.15e-05 | 0.52 | `ATCTGGGTCA` |
| MA0071.1 | chr12 | + | 47810587 | 47810596 | 3.15e-05 | 0.52 | `ATCTGGGTCA` |
| MA0071.1 | chr17 | + | 30415246 | 30415255 | 3.15e-05 | 0.52 | `ATCTGGGTCA` |
| MA0071.1 | chr2 | + | 204600359 | 204600368 | 3.24e-05 | 0.52 | `TTAGAGGTCA` |
| MA0071.1 | chr19 | − | 55536286 | 55536295 | 3.24e-05 | 0.52 | `TTAGAGGTCA` |
| MA0071.1 | chr2 | − | 98454203 | 98454212 | 3.34e-05 | 0.52 | `AACAGGGTCA` |
| MA0071.1 | chr2 | + | 125846123 | 125846132 | 3.34e-05 | 0.52 | `AACAGGGTCA` |
| MA0071.1 | chrX | + | 70760629 | 70760638 | 3.34e-05 | 0.52 | `AACAGGGTCA` |
| MA0071.1 | chr11 | − | 74489162 | 74489171 | 3.34e-05 | 0.52 | `AACAGGGTCA` |
| MA0071.1 | chr12 | − | 55014468 | 55014477 | 3.34e-05 | 0.52 | `AACAGGGTCA` |
| MA0071.1 | chr15 | − | 62233168 | 62233177 | 3.34e-05 | 0.52 | `AACAGGGTCA` |
| MA0071.1 | chr1 | − | 28847964 | 28847973 | 3.42e-05 | 0.52 | `AATTAGGTCA` |
| MA0071.1 | chr14 | − | 34938462 | 34938471 | 3.42e-05 | 0.52 | `AATTAGGTCA` |
| MA0071.1 | chr14 | + | 68331122 | 68331131 | 3.42e-05 | 0.52 | `AATTAGGTCA` |
| MA0071.1 | chr1 | − | 110975671 | 110975680 | 3.51e-05 | 0.52 | `AACCAGGTCA` |
| MA0071.1 | chr1 | − | 120065778 | 120065787 | 3.51e-05 | 0.52 | `AACCAGGTCA` |
| MA0071.1 | chr2 | + | 65004025 | 65004034 | 3.51e-05 | 0.52 | `AACCAGGTCA` |
| MA0071.1 | chr3 | + | 49731892 | 49731901 | 3.51e-05 | 0.52 | `AACCAGGTCA` |
| MA0071.1 | chr6 | + | 91240052 | 91240061 | 3.51e-05 | 0.52 | `AACCAGGTCA` |
| MA0071.1 | chr7 | − | 4689777 | 4689786 | 3.51e-05 | 0.52 | `AACCAGGTCA` |
| MA0071.1 | chr9 | + | 35832915 | 35832924 | 3.51e-05 | 0.52 | `AACCAGGTCA` |
| MA0071.1 | chr14 | − | 105306873 | 105306882 | 3.51e-05 | 0.52 | `AACCAGGTCA` |
| MA0071.1 | chr3 | + | 128956991 | 128957000 | 3.6e-05 | 0.52 | `TTGTAGGTCA` |
| MA0071.1 | chr9 | − | 85942738 | 85942747 | 3.6e-05 | 0.52 | `TTGTAGGTCA` |
| MA0071.1 | chr14 | + | 61199078 | 61199087 | 3.6e-05 | 0.52 | `TTGTAGGTCA` |
| MA0071.1 | chr1 | + | 172097953 | 172097962 | 3.68e-05 | 0.52 | `TAATAGGTCA` |
| MA0071.1 | chr6 | − | 174441 | 174450 | 3.77e-05 | 0.52 | `ATAAGGGTCA` |
| MA0071.1 | chr9 | − | 78277221 | 78277230 | 3.77e-05 | 0.52 | `ATAAGGGTCA` |
| MA0071.1 | chrX | + | 7043506 | 7043515 | 3.77e-05 | 0.52 | `ATAAGGGTCA` |
| MA0071.1 | chr1 | + | 179634721 | 179634730 | 3.86e-05 | 0.52 | `TAGAAGGTCA` |
| MA0071.1 | chr15 | − | 82440779 | 82440788 | 3.86e-05 | 0.52 | `TAGAAGGTCA` |
| MA0071.1 | chr21 | − | 25866238 | 25866247 | 3.86e-05 | 0.52 | `TAGAAGGTCA` |
| MA0071.1 | chr3 | + | 184362223 | 184362232 | 3.95e-05 | 0.52 | `ATACAGGTCA` |
| MA0071.1 | chr6 | + | 64287537 | 64287546 | 3.95e-05 | 0.52 | `ATACAGGTCA` |
| MA0071.1 | chr3 | − | 196746162 | 196746171 | 4.04e-05 | 0.52 | `AAGGAGGTCA` |
| MA0071.1 | chr4 | − | 7529615 | 7529624 | 4.04e-05 | 0.52 | `AAGGAGGTCA` |
| MA0071.1 | chrX | + | 46373503 | 46373512 | 4.04e-05 | 0.52 | `AAGGAGGTCA` |
| MA0071.1 | chr10 | − | 89612030 | 89612039 | 4.04e-05 | 0.52 | `AAGGAGGTCA` |
| MA0071.1 | chr15 | + | 68542831 | 68542840 | 4.04e-05 | 0.52 | `AAGGAGGTCA` |
| MA0071.1 | chr17 | + | 74225778 | 74225787 | 4.04e-05 | 0.52 | `AAGGAGGTCA` |
| MA0071.1 | chr1 | − | 27824154 | 27824163 | 4.13e-05 | 0.52 | `TTCAGGGTCA` |
| MA0071.1 | chr1 | − | 108051393 | 108051402 | 4.13e-05 | 0.52 | `TTCAGGGTCA` |
| MA0071.1 | chr6 | + | 32734233 | 32734242 | 4.13e-05 | 0.52 | `TTCAGGGTCA` |
| MA0071.1 | chr6 | + | 33375373 | 33375382 | 4.13e-05 | 0.52 | `TTCAGGGTCA` |
| MA0071.1 | chr10 | − | 73764812 | 73764821 | 4.13e-05 | 0.52 | `TTCAGGGTCA` |
| MA0071.1 | chr11 | − | 65076584 | 65076593 | 4.13e-05 | 0.52 | `TTCAGGGTCA` |
| MA0071.1 | chr11 | + | 128217143 | 128217152 | 4.13e-05 | 0.52 | `TTCAGGGTCA` |
| MA0071.1 | chr17 | + | 77093684 | 77093693 | 4.13e-05 | 0.52 | `TTCAGGGTCA` |
| MA0071.1 | chr3 | + | 113554290 | 113554299 | 4.22e-05 | 0.521 | `TTTTAGGTCA` |
| MA0071.1 | chr7 | − | 44757557 | 44757566 | 4.22e-05 | 0.521 | `TTTTAGGTCA` |
| MA0071.1 | chr17 | + | 63077738 | 63077747 | 4.22e-05 | 0.521 | `TTTTAGGTCA` |
| MA0071.1 | chr6 | − | 264724 | 264733 | 4.41e-05 | 0.53 | `TTCCAGGTCA` |
| MA0071.1 | chr7 | + | 4689725 | 4689734 | 4.41e-05 | 0.53 | `TTCCAGGTCA` |
| MA0071.1 | chr8 | − | 98725236 | 98725245 | 4.41e-05 | 0.53 | `GTCTAGGTCA` |
| MA0071.1 | chr12 | − | 2335610 | 2335619 | 4.41e-05 | 0.53 | `GTCTAGGTCA` |
| MA0071.1 | chr19 | + | 44589860 | 44589869 | 4.41e-05 | 0.53 | `TTCCAGGTCA` |
| MA0071.1 | chr6 | − | 106670135 | 106670144 | 4.68e-05 | 0.539 | `ATCGGGGTCA` |
| MA0071.1 | chr2 | + | 191535315 | 191535324 | 4.77e-05 | 0.539 | `AATGAGGTCA` |
| MA0071.1 | chr3 | − | 116347706 | 116347715 | 4.77e-05 | 0.539 | `AATGAGGTCA` |
| MA0071.1 | chr9 | + | 116483896 | 116483905 | 4.77e-05 | 0.539 | `AATGAGGTCA` |
| MA0071.1 | chr10 | − | 82002464 | 82002473 | 4.77e-05 | 0.539 | `AATGAGGTCA` |
| MA0071.1 | chr17 | − | 1457484 | 1457493 | 4.77e-05 | 0.539 | `AATGAGGTCA` |
| MA0071.1 | chr8 | − | 61987045 | 61987054 | 4.87e-05 | 0.539 | `AACTGGGTCA` |
| MA0071.1 | chr8 | + | 141668280 | 141668289 | 4.87e-05 | 0.539 | `AACTGGGTCA` |
| MA0071.1 | chr17 | − | 22683952 | 22683961 | 4.87e-05 | 0.539 | `AACTGGGTCA` |
| MA0071.1 | chr12 | + | 67488811 | 67488820 | 4.95e-05 | 0.539 | `GTAAAGGTCA` |
| MA0071.1 | chr16 | − | 66666507 | 66666516 | 4.95e-05 | 0.539 | `GTAAAGGTCA` |
| MA0071.1 | chr19 | − | 52153952 | 52153961 | 4.95e-05 | 0.539 | `GTAAAGGTCA` |
| MA0071.1 | chr20 | − | 47337881 | 47337890 | 4.95e-05 | 0.539 | `GTAAAGGTCA` |
| MA0071.1 | chr2 | + | 58127930 | 58127939 | 5.05e-05 | 0.539 | `TTGGAGGTCA` |
| MA0071.1 | chr5 | − | 75179345 | 75179354 | 5.05e-05 | 0.539 | `TTGGAGGTCA` |
| MA0071.1 | chrX | + | 151750339 | 151750348 | 5.05e-05 | 0.539 | `TTGGAGGTCA` |
| MA0071.1 | chr9 | + | 113814378 | 113814387 | 5.14e-05 | 0.539 | `TAAGAGGTCA` |
| MA0071.1 | chr2 | − | 231557812 | 231557821 | 5.33e-05 | 0.539 | `CTCAAGGTCA` |
| MA0071.1 | chr3 | − | 134774479 | 134774488 | 5.33e-05 | 0.539 | `ACCAAGGTCA` |
| MA0071.1 | chr3 | + | 179719781 | 179719790 | 5.33e-05 | 0.539 | `ACCAAGGTCA` |
| MA0071.1 | chr5 | − | 156862324 | 156862333 | 5.33e-05 | 0.539 | `CTCAAGGTCA` |
| MA0071.1 | chr7 | + | 92277071 | 92277080 | 5.33e-05 | 0.539 | `ACCAAGGTCA` |
| MA0071.1 | chr8 | − | 144179090 | 144179099 | 5.33e-05 | 0.539 | `CTCAAGGTCA` |
| MA0071.1 | chr8 | + | 28252533 | 28252542 | 5.33e-05 | 0.539 | `ACCAAGGTCA` |
| MA0071.1 | chr8 | − | 67056925 | 67056934 | 5.33e-05 | 0.539 | `ACCAAGGTCA` |
| MA0071.1 | chr8 | + | 104021944 | 104021953 | 5.33e-05 | 0.539 | `ACCAAGGTCA` |
| MA0071.1 | chr15 | − | 42795856 | 42795865 | 5.33e-05 | 0.539 | `CTCAAGGTCA` |
| MA0071.1 | chr15 | − | 56533959 | 56533968 | 5.33e-05 | 0.539 | `CTCAAGGTCA` |
| MA0071.1 | chr17 | + | 70803314 | 70803323 | 5.33e-05 | 0.539 | `CTCAAGGTCA` |
| MA0071.1 | chr18 | + | 22256654 | 22256663 | 5.33e-05 | 0.539 | `ACCAAGGTCA` |
| MA0071.1 | chr19 | + | 42553653 | 42553662 | 5.33e-05 | 0.539 | `CTCAAGGTCA` |
| MA0071.1 | chr20 | + | 48666787 | 48666796 | 5.33e-05 | 0.539 | `CTCAAGGTCA` |
| MA0071.1 | chr4 | + | 185440075 | 185440084 | 5.5e-05 | 0.539 | `TAGTAGGTCA` |
| MA0071.1 | chr2 | − | 122798093 | 122798102 | 5.6e-05 | 0.539 | `ATGAGGGTCA` |
| MA0071.1 | chr3 | − | 113661545 | 113661554 | 5.6e-05 | 0.539 | `ATGAGGGTCA` |
| MA0071.1 | chr3 | − | 187990719 | 187990728 | 5.6e-05 | 0.539 | `ATGAGGGTCA` |
| MA0071.1 | chr6 | + | 119805861 | 119805870 | 5.6e-05 | 0.539 | `ATGAGGGTCA` |
| MA0071.1 | chr1 | + | 176895772 | 176895781 | 5.68e-05 | 0.539 | `AAAAGGGTCA` |
| MA0071.1 | chr11 | + | 90673046 | 90673055 | 5.68e-05 | 0.539 | `AAAAGGGTCA` |
| MA0071.1 | chr12 | + | 112095848 | 112095857 | 5.78e-05 | 0.539 | `ATGCAGGTCA` |
| MA0071.1 | chr16 | − | 11364739 | 11364748 | 5.78e-05 | 0.539 | `ATGCAGGTCA` |
| MA0071.1 | chr22 | − | 40589050 | 40589059 | 5.78e-05 | 0.539 | `ATGCAGGTCA` |
| MA0071.1 | chr1 | − | 23755927 | 23755936 | 5.87e-05 | 0.539 | `AAACAGGTCA` |
| MA0071.1 | chr13 | + | 97962685 | 97962694 | 5.87e-05 | 0.539 | `AAACAGGTCA` |
| MA0071.1 | chr17 | + | 63746882 | 63746891 | 5.87e-05 | 0.539 | `AAACAGGTCA` |
| MA0071.1 | chr19 | + | 43228717 | 43228726 | 5.87e-05 | 0.539 | `AAACAGGTCA` |
| MA0071.1 | chr20 | + | 45847265 | 45847274 | 5.87e-05 | 0.539 | `AAACAGGTCA` |
| MA0071.1 | chr1 | + | 204920092 | 204920101 | 5.95e-05 | 0.539 | `TTTGAGGTCA` |
| MA0071.1 | chr3 | − | 113554025 | 113554034 | 5.95e-05 | 0.539 | `TTTGAGGTCA` |
| MA0071.1 | chr3 | + | 196746170 | 196746179 | 5.95e-05 | 0.539 | `TTTGAGGTCA` |
| MA0071.1 | chr6 | + | 16581729 | 16581738 | 5.95e-05 | 0.539 | `TTTGAGGTCA` |
| MA0071.1 | chr10 | + | 26816847 | 26816856 | 5.95e-05 | 0.539 | `TTTGAGGTCA` |
| MA0071.1 | chr10 | + | 45236750 | 45236759 | 5.95e-05 | 0.539 | `TTTGAGGTCA` |
| MA0071.1 | chr12 | − | 29196653 | 29196662 | 5.95e-05 | 0.539 | `TTTGAGGTCA` |
| MA0071.1 | chr12 | − | 91090831 | 91090840 | 5.95e-05 | 0.539 | `TTTGAGGTCA` |
| MA0071.1 | chr17 | + | 44650645 | 44650654 | 5.95e-05 | 0.539 | `TTTGAGGTCA` |
| MA0071.1 | chr17 | + | 53282792 | 53282801 | 5.95e-05 | 0.539 | `TTTGAGGTCA` |
| MA0071.1 | chr17 | − | 73634996 | 73635005 | 5.95e-05 | 0.539 | `TTTGAGGTCA` |
| MA0071.1 | chr19 | + | 59404072 | 59404081 | 5.95e-05 | 0.539 | `TTTGAGGTCA` |
| MA0071.1 | chr1 | + | 204920104 | 204920113 | 6.05e-05 | 0.541 | `TTCTGGGTCA` |
| MA0071.1 | chr6 | − | 106670144 | 106670153 | 6.05e-05 | 0.541 | `TTCTGGGTCA` |
| MA0071.1 | chr12 | − | 93479124 | 93479133 | 6.05e-05 | 0.541 | `TTCTGGGTCA` |
| MA0071.1 | chr15 | + | 42795980 | 42795989 | 6.25e-05 | 0.556 | `TACAGGGTCA` |
| MA0071.1 | chr7 | − | 114344749 | 114344758 | 6.33e-05 | 0.559 | `TATTAGGTCA` |
| MA0071.1 | chr20 | + | 23263029 | 23263038 | 6.33e-05 | 0.559 | `TATTAGGTCA` |
| MA0071.1 | chr3 | − | 123811540 | 123811549 | 6.6e-05 | 0.575 | `GACTAGGTCA` |
| MA0071.1 | chr5 | + | 126684013 | 126684022 | 6.6e-05 | 0.575 | `ATTAGGGTCA` |
| MA0071.1 | chr20 | + | 45820743 | 45820752 | 6.6e-05 | 0.575 | `GACTAGGTCA` |
| MA0071.1 | chr1 | − | 207900017 | 207900026 | 6.69e-05 | 0.575 | `ATTCAGGTCA` |
| MA0071.1 | chr6 | + | 138312989 | 138312998 | 6.69e-05 | 0.575 | `ATTCAGGTCA` |
| MA0071.1 | chr10 | + | 23030609 | 23030618 | 6.69e-05 | 0.575 | `ATTCAGGTCA` |
| MA0071.1 | chr16 | − | 23745482 | 23745491 | 6.69e-05 | 0.575 | `ATTCAGGTCA` |
| MA0071.1 | chr8 | + | 72913758 | 72913767 | 6.88e-05 | 0.586 | `TTAAGGGTCA` |
| MA0071.1 | chr15 | + | 29296591 | 29296600 | 6.88e-05 | 0.586 | `AACGGGGTCA` |
| MA0071.1 | chr17 | + | 63745659 | 63745668 | 7.06e-05 | 0.586 | `GTATAGGTCA` |
| MA0071.1 | chr3 | + | 187984154 | 187984163 | 7.15e-05 | 0.586 | `GTGAAGGTCA` |
| MA0071.1 | chr17 | − | 24320451 | 24320460 | 7.15e-05 | 0.586 | `GTGAAGGTCA` |
| MA0071.1 | chr1 | − | 204957584 | 204957593 | 7.52e-05 | 0.586 | `CTCTAGGTCA` |
| MA0071.1 | chr3 | − | 23670151 | 23670160 | 7.52e-05 | 0.586 | `GAAAAGGTCA` |
| MA0071.1 | chr3 | + | 113529142 | 113529151 | 7.52e-05 | 0.586 | `CTCTAGGTCA` |
| MA0071.1 | chr3 | + | 116349184 | 116349193 | 7.52e-05 | 0.586 | `GAAAAGGTCA` |
| MA0071.1 | chr5 | − | 78059913 | 78059922 | 7.52e-05 | 0.586 | `CTCTAGGTCA` |
| MA0071.1 | chr5 | + | 130742684 | 130742693 | 7.52e-05 | 0.586 | `CTCTAGGTCA` |
| MA0071.1 | chr6 | + | 16581629 | 16581638 | 7.52e-05 | 0.586 | `GAAAAGGTCA` |
| MA0071.1 | chr6 | − | 26468909 | 26468918 | 7.52e-05 | 0.586 | `ATAGGGGTCA` |
| MA0071.1 | chr6 | + | 106737096 | 106737105 | 7.52e-05 | 0.586 | `GAAAAGGTCA` |
| MA0071.1 | chr11 | − | 101693078 | 101693087 | 7.52e-05 | 0.586 | `GAAAAGGTCA` |
| MA0071.1 | chr18 | + | 58979357 | 58979366 | 7.52e-05 | 0.586 | `GAAAAGGTCA` |
| MA0071.1 | chr19 | + | 7676821 | 7676830 | 7.52e-05 | 0.586 | `ACCTAGGTCA` |
| MA0071.1 | chr19 | − | 7676819 | 7676828 | 7.52e-05 | 0.586 | `ACCTAGGTCA` |
| MA0071.1 | chr19 | + | 16343341 | 16343350 | 7.52e-05 | 0.586 | `GAAAAGGTCA` |
| MA0071.1 | chr22 | + | 36010478 | 36010487 | 7.52e-05 | 0.586 | `GAAAAGGTCA` |
| MA0071.1 | chr17 | + | 38794052 | 38794061 | 7.62e-05 | 0.586 | `TAGGAGGTCA` |
| MA0071.1 | chr1 | − | 195123929 | 195123938 | 7.71e-05 | 0.586 | `CACAAGGTCA` |
| MA0071.1 | chr2 | + | 20270477 | 20270486 | 7.71e-05 | 0.586 | `CACAAGGTCA` |
| MA0071.1 | chr3 | − | 45001004 | 45001013 | 7.71e-05 | 0.586 | `CACAAGGTCA` |
| MA0071.1 | chr3 | + | 57077752 | 57077761 | 7.71e-05 | 0.586 | `CACAAGGTCA` |
| MA0071.1 | chr9 | − | 17853607 | 17853616 | 7.71e-05 | 0.586 | `CACAAGGTCA` |
| MA0071.1 | chr11 | − | 7490430 | 7490439 | 7.71e-05 | 0.586 | `CACAAGGTCA` |
| MA0071.1 | chr11 | − | 7520598 | 7520607 | 7.71e-05 | 0.586 | `CACAAGGTCA` |
| MA0071.1 | chr2 | + | 181885595 | 181885604 | 7.81e-05 | 0.586 | `ATGTGGGTCA` |
| MA0071.1 | chr4 | − | 185440099 | 185440108 | 7.81e-05 | 0.586 | `ATGTGGGTCA` |
| MA0071.1 | chr6 | + | 340164 | 340173 | 7.81e-05 | 0.586 | `ATGTGGGTCA` |
| MA0071.1 | chr11 | − | 82548728 | 82548737 | 7.81e-05 | 0.586 | `ATGTGGGTCA` |
| MA0071.1 | chr12 | − | 92013287 | 92013296 | 7.81e-05 | 0.586 | `ATGTGGGTCA` |
| MA0071.1 | chr2 | + | 70170543 | 70170552 | 7.9e-05 | 0.586 | `AAATGGGTCA` |
| MA0071.1 | chr7 | + | 139113924 | 139113933 | 7.9e-05 | 0.586 | `AAATGGGTCA` |
| MA0071.1 | chr1 | − | 111548252 | 111548261 | 7.99e-05 | 0.586 | `AAGAGGGTCA` |
| MA0071.1 | chr5 | + | 138925131 | 138925140 | 7.99e-05 | 0.586 | `AAGAGGGTCA` |
| MA0071.1 | chr1 | + | 155418418 | 155418427 | 8.09e-05 | 0.586 | `TTCGGGGTCA` |
| MA0071.1 | chr4 | + | 40909461 | 40909470 | 8.09e-05 | 0.586 | `TTCGGGGTCA` |
| MA0071.1 | chr6 | + | 33237275 | 33237284 | 8.09e-05 | 0.586 | `TTCGGGGTCA` |
| MA0071.1 | chr10 | − | 13805640 | 13805649 | 8.09e-05 | 0.586 | `TTCGGGGTCA` |
| MA0071.1 | chr15 | + | 29408670 | 29408679 | 8.09e-05 | 0.586 | `TTCGGGGTCA` |
| MA0071.1 | chr1 | + | 76034556 | 76034565 | 8.27e-05 | 0.586 | `CTAAAGGTCA` |
| MA0071.1 | chr2 | − | 230830903 | 230830912 | 8.27e-05 | 0.586 | `CTAAAGGTCA` |
| MA0071.1 | chr4 | + | 88068936 | 88068945 | 8.27e-05 | 0.586 | `CTAAAGGTCA` |
| MA0071.1 | chr5 | − | 138995996 | 138996005 | 8.27e-05 | 0.586 | `CTAAAGGTCA` |
| MA0071.1 | chr5 | + | 139205131 | 139205140 | 8.27e-05 | 0.586 | `ACAAAGGTCA` |
| MA0071.1 | chr6 | + | 31343809 | 31343818 | 8.27e-05 | 0.586 | `ACAAAGGTCA` |
| MA0071.1 | chr10 | − | 14742078 | 14742087 | 8.27e-05 | 0.586 | `CTAAAGGTCA` |
| MA0071.1 | chr16 | − | 70408087 | 70408096 | 8.27e-05 | 0.586 | `CTAAAGGTCA` |
| MA0071.1 | chr22 | − | 46762853 | 46762862 | 8.27e-05 | 0.586 | `CTAAAGGTCA` |
| MA0071.1 | chr2 | − | 201690591 | 201690600 | 8.36e-05 | 0.586 | `AAGCAGGTCA` |
| MA0071.1 | chr8 | + | 131329123 | 131329132 | 8.36e-05 | 0.586 | `AAGCAGGTCA` |
| MA0071.1 | chr14 | − | 105399476 | 105399485 | 8.36e-05 | 0.586 | `AAGCAGGTCA` |
| MA0071.1 | chr16 | + | 11775626 | 11775635 | 8.36e-05 | 0.586 | `AAGCAGGTCA` |
| MA0071.1 | chr17 | − | 37099584 | 37099593 | 8.36e-05 | 0.586 | `AAGCAGGTCA` |
| MA0071.1 | chr6 | + | 88670680 | 88670689 | 8.45e-05 | 0.586 | `GTTAAGGTCA` |
| MA0071.1 | chr6 | − | 127082012 | 127082021 | 8.45e-05 | 0.586 | `GTTAAGGTCA` |
| MA0071.1 | chr12 | − | 26844078 | 26844087 | 8.45e-05 | 0.586 | `GTTAAGGTCA` |
| MA0071.1 | chr14 | + | 61135686 | 61135695 | 8.45e-05 | 0.586 | `GTTAAGGTCA` |
| MA0071.1 | chr3 | − | 72308574 | 72308583 | 8.63e-05 | 0.595 | `TACTGGGTCA` |
| MA0071.1 | chr10 | − | 112524119 | 112524128 | 8.63e-05 | 0.595 | `TACTGGGTCA` |
| MA0071.1 | chr2 | + | 111649759 | 111649768 | 8.82e-05 | 0.604 | `ATTTGGGTCA` |
| MA0071.1 | chr6 | + | 340024 | 340033 | 8.82e-05 | 0.604 | `ATTTGGGTCA` |
| MA0071.1 | chr17 | − | 70803384 | 70803393 | 8.93e-05 | 0.608 | `ATCCGGGTCA` |
| MA0071.1 | chr2 | + | 9699599 | 9699608 | 9.02e-05 | 0.608 | `TCCAAGGTCA` |
| MA0071.1 | chr5 | − | 131786458 | 131786467 | 9.02e-05 | 0.608 | `TCCAAGGTCA` |
| MA0071.1 | chr10 | − | 115732603 | 115732612 | 9.02e-05 | 0.608 | `TCCAAGGTCA` |
| MA0071.1 | chr14 | + | 75016811 | 75016820 | 9.02e-05 | 0.608 | `TCCAAGGTCA` |
| MA0071.1 | chr3 | + | 52004977 | 52004986 | 9.2e-05 | 0.616 | `AATAGGGTCA` |
| MA0071.1 | chr9 | − | 131690329 | 131690338 | 9.2e-05 | 0.616 | `TTATGGGTCA` |
| MA0071.1 | chr14 | + | 96424450 | 96424459 | 9.29e-05 | 0.619 | `GTAGAGGTCA` |
| MA0071.1 | chr17 | − | 60412707 | 60412716 | 9.29e-05 | 0.619 | `GTAGAGGTCA` |
| MA0071.1 | chr8 | + | 67057039 | 67057048 | 9.39e-05 | 0.623 | `TTGAGGGTCA` |
| MA0071.1 | chr1 | − | 35412800 | 35412809 | 9.47e-05 | 0.627 | `AATCAGGTCA` |
| MA0071.1 | chr6 | − | 30569857 | 30569866 | 9.56e-05 | 0.627 | `TAAAGGGTCA` |
| MA0071.1 | chr12 | + | 15832911 | 15832920 | 9.56e-05 | 0.627 | `TAAAGGGTCA` |
| MA0071.1 | chr16 | + | 45510577 | 45510586 | 9.56e-05 | 0.627 | `TAAAGGGTCA` |
| MA0071.1 | chr6 | + | 150011791 | 150011800 | 9.77e-05 | 0.628 | `ACCGAGGTCA` |
| MA0071.1 | chr11 | − | 85835751 | 85835760 | 9.77e-05 | 0.628 | `ACCGAGGTCA` |
| MA0071.1 | chr17 | + | 2116777 | 2116786 | 9.77e-05 | 0.628 | `ACCGAGGTCA` |
| MA0071.1 | chr1 | + | 204439883 | 204439892 | 9.96e-05 | 0.628 | `TTGCAGGTCA` |
| MA0071.1 | chr1 | − | 223683146 | 223683155 | 9.96e-05 | 0.628 | `TTGCAGGTCA` |
| MA0071.1 | chr6 | + | 26135239 | 26135248 | 9.96e-05 | 0.628 | `GTGTAGGTCA` |
| MA0071.1 | chr6 | − | 27215278 | 27215287 | 9.96e-05 | 0.628 | `GTGTAGGTCA` |
| MA0071.1 | chr6 | − | 45906559 | 45906568 | 9.96e-05 | 0.628 | `TTGCAGGTCA` |
| MA0071.1 | chr7 | + | 7950989 | 7950998 | 9.96e-05 | 0.628 | `GTGTAGGTCA` |
| MA0071.1 | chr8 | + | 131329096 | 131329105 | 9.96e-05 | 0.628 | `TTGCAGGTCA` |
| MA0071.1 | chr11 | − | 64650913 | 64650922 | 9.96e-05 | 0.628 | `TTGCAGGTCA` |
| MA0071.1 | chr14 | + | 93495693 | 93495702 | 9.96e-05 | 0.628 | `GTGTAGGTCA` |
| MA0071.1 | chr18 | − | 19187592 | 19187601 | 9.96e-05 | 0.628 | `TTGCAGGTCA` |

---

**DEBUGGING INFORMATION**


---

Command line:

```
/ebi/sw/MEME/VM-cluster410/meme-versions/4.10.0/bin/fimo --parse-genomic-coord --verbosity 1 --oc fimo_out_6 --bgfile ./background --motif MA0071.1 db/JASPAR_CORE_2014_vertebrates.meme ./Supplementary_Table_1.500bp.fa
```

Settings:

```
|  |  |  |
| --- | --- | --- |
| output directory = fimo_out_6 | MEME file name = db/JASPAR_CORE_2014_vertebrates.meme | sequence file name = ./Supplementary_Table_1.500bp.fa |
| background file name = ./background | allow clobber = true | compute q-values = true |
| parse genomic coord. = true | text only = false | scan both strands = true |
| max sequence length = 250000000 | output threshold = 0.0001 | threshold type = p-value |
| max stored scores = 100000 | pseudocount = 0.1 | verbosity = 1 |
| selected motif = MA0071.1 |  |  |
```

This information can be useful in the event you wish to report a
problem with the FIMO software.

---

**Go to top**
