# Supplementary material for: The MEME Suite
Source: Nucleic Acids Res. 2015 May 7;43(Web Server issue):W39–49. doi: 10.1093/nar/gkv416 (PMC4489269; doi:10.1093/nar/gkv416)
Supplement: SUPPLEMENTARY DATA [file supp_gkv416_nar-00283-web-b-2015-File005.zip › case4/meme-chip/fimo_out_7/fimo.html]

FIMO Results


---

|  |  |  |
| --- | --- | --- |
| **Database and Motifs** | **High-scoring Motif Occurrences** | **Debugging Information** |

  
  

---

**FIMO - Motif search tool**


---

FIMO version 4.10.0,
(Release date: Wed May 21 10:35:36 2014 +1000)

For further information on how to interpret these results
or to get a copy of the FIMO software please access
http://meme.nbcr.net

If you use FIMO in your research, please cite the following paper:  
Charles E. Grant, Timothy L. Bailey, and William Stafford Noble,
"FIMO: Scanning for occurrences of a given motif",
*Bioinformatics*, **27**(7):1017-1018, 2011.
[full text]

---

**DATABASE AND MOTIFS**


---

DATABASE
./Supplementary\_Table\_1.500bp.fa  
Database contains
2776
sequences,
1388000
residues

MOTIFS
db/uniprobe\_mouse.meme
(nucleotide)

| MOTIF | WIDTH | BEST POSSIBLE MATCH |
| --- | --- | --- |
| UP00078\_1 | 17 | GGGTTTAATTAAAATTC |
| UP00059\_1 | 14 | CTAATATTGCTAAA |
| UP00099\_1 | 17 | CTCAGCAGCTGCTCCTG |
| UP00020\_1 | 16 | ACGATGACGTCATCGA |
| UP00012\_1 | 15 | TAATTCAATGAAGTG |
| UP00043\_1 | 16 | TCTTTCGAGGAATTTG |
| UP00050\_1 | 22 | GGAAGAGTCACGTGACCAATAC |
| UP00001\_1 | 15 | ATAAAGGCGCGCGAT |
| UP00003\_1 | 15 | ATAAGGGCGCGCGAT |
| UP00007\_1 | 14 | TCCGCCCCCGCATT |
| UP00015\_1 | 15 | AGGACCCGGAAGTAA |
| UP00407\_1 | 13 | TACAAGGAAGTAA |
| UP00068\_1 | 17 | TAAAAGGTGTGAAAATT |
| UP00079\_1 | 17 | TATTCAAGGTCATGCGA |
| UP00073\_1 | 17 | AAAAAGTAAACAAAGAC |
| UP00041\_1 | 16 | AAAGTAAACAAAAATT |
| UP00039\_1 | 17 | AAAAAGTAAACAAACCC |
| UP00025\_1 | 17 | AAAATGTAAACAAACAG |
| UP00061\_1 | 17 | TAAATGTAAACAAAGGT |
| UP00408\_1 | 17 | CAATACCGGAAGTGTAA |
| UP00032\_1 | 22 | TTTTTAGAGATAAGAAATAAAG |
| UP00080\_1 | 17 | TAAACTGATAAGAAGAT |
| UP00100\_1 | 17 | TATAGAGATAAGAATTG |
| UP00070\_1 | 16 | TCGTACCCGCATCATT |
| UP00024\_1 | 16 | TATCGACCCCCCACAG |
| UP00042\_1 | 17 | CAGATGTGCACATACGT |
| UP00084\_1 | 17 | GAGTGTACGTACGATGG |
| UP00055\_1 | 16 | ACTATGAATGAATGAT |
| UP00035\_1 | 16 | ACTATGCCAACCTACC |
| UP00066\_1 | 17 | CTTCAGGGGTCAATTGA |
| UP00391\_1 | 14 | TGGAGGTAATTAAC |
| UP00072\_1 | 16 | ATTTACGACAAATAGC |
| UP00086\_1 | 14 | GAGAACCGAAACTG |
| UP00018\_1 | 15 | CGTATCGAAACCAAA |
| UP00040\_1 | 15 | ATAAACCGAAACCAA |
| UP00011\_1 | 17 | CTGATCGAAACCAAAGT |
| UP00074\_1 | 15 | CAAAATCGAAACTAA |
| UP00103\_1 | 16 | CCGATGACGTCATCGT |
| UP00093\_1 | 16 | TCGACCCCGCCCCTAT |
| UP00067\_1 | 17 | AATCCCTTTGATCTATC |
| UP00045\_1 | 17 | AAATTTGCTGACTTAGC |
| UP00044\_1 | 15 | TAAAAATGCTGACTT |
| UP00060\_1 | 16 | TGACCACGTGGTCGGG |
| UP00097\_1 | 16 | GGGCCGTGTGCAAAAA |
| UP00092\_1 | 17 | ATGGAAACCGTTATTTT |
| UP00081\_1 | 17 | TTGAAAACCGTTAATTT |
| UP00036\_1 | 16 | GAAGAACAGGTGTCCG |
| UP00017\_1 | 17 | CTTAACCACTTAAGGAT |
| UP00009\_1 | 16 | TCTCAAAGGTCACGAG |
| UP00027\_1 | 16 | TTTTACAGTAGCAAAA |
| UP00052\_1 | 16 | ATGTACAGTAGCAAAG |
| UP00088\_1 | 16 | TTGGGGGCGCCCCTAG |
| UP00048\_1 | 16 | TCTCAAAGGTCACCTG |
| UP00098\_1 | 23 | TGTGACCCTTAGCAACCGATTAA |
| UP00056\_1 | 15 | TACCATAGCAACGGT |
| UP00076\_1 | 15 | CCGCATAGCAACGGA |
| UP00053\_1 | 17 | TGTCGTGACCCCTTAAT |
| UP00085\_1 | 14 | TTAAGAGGAAGTTA |
| UP00008\_1 | 17 | AATAGGGTATCATATAT |
| UP00000\_1 | 17 | CAAATCCAGACATCAGA |
| UP00030\_1 | 17 | ATAAGAACAAAGGACTA |
| UP00101\_1 | 14 | TAATTGTTCTAAAC |
| UP00096\_1 | 16 | TTAAGAACAATAATTT |
| UP00004\_1 | 16 | GCTAATTATAATTATC |
| UP00075\_1 | 17 | TAGTGAACAATAGATTT |
| UP00014\_1 | 15 | ATAAACAATTAATCA |
| UP00064\_1 | 16 | TTCAATTGTTCTAAAA |
| UP00069\_1 | 16 | AATCAATTCAATAATT |
| UP00071\_1 | 16 | TTTAATTATAATTAAG |
| UP00023\_1 | 16 | ATTGAACAATGGAATT |
| UP00062\_1 | 17 | AGAAGAACAAAGGACTA |
| UP00091\_1 | 16 | TTTAGAACAATAAAAT |
| UP00034\_1 | 22 | AATAAAGAACAATAGAATTTCA |
| UP00051\_1 | 17 | TTATCTATTGTTCTTTA |
| UP00049\_1 | 14 | ATTTTACGGAAAAT |
| UP00002\_1 | 17 | GGTCCCGCCCCCTTCTC |
| UP00406\_1 | 16 | GTACATCCGGATTTTT |
| UP00077\_1 | 14 | TTCCATATATGGAA |
| UP00016\_1 | 16 | TATAATTATAATATTC |
| UP00029\_1 | 16 | TCTTTATATATAAATA |
| UP00089\_1 | 17 | ACTTAGTTAACTAAAAA |
| UP00058\_1 | 17 | TATAGATCAAAGGAAAA |
| UP00054\_1 | 17 | TATAGATCAAAGGAAAA |
| UP00083\_1 | 17 | ATTTCCTTTGATCTATA |
| UP00005\_1 | 15 | ATTCCCTGAGGGGAA |
| UP00010\_1 | 14 | TTGCCCTAGGGCAT |
| UP00087\_1 | 15 | ATTGCCTGAGGCGAA |
| UP00028\_1 | 15 | ATTGCCTGAGGCGAT |
| UP00046\_1 | 17 | ATCCACAGGTGCGAAAA |
| UP00019\_1 | 17 | CTAAGGTTCTAGATCAC |
| UP00031\_1 | 17 | AATCGCACTGCATTCCG |
| UP00047\_1 | 15 | AAGCCCCCCAAAAAT |
| UP00037\_1 | 15 | AACAAACAACAAGAG |
| UP00094\_1 | 17 | TCTTTGGCGTACCCTAA |
| UP00065\_1 | 16 | TGGCGCGCGCGCCTGA |
| UP00082\_1 | 14 | TTATGTACTAATAA |
| UP00021\_1 | 15 | TCCCCCCCCCCCCCC |
| UP00033\_1 | 17 | TATTATGGGATGGATAA |
| UP00095\_1 | 17 | CGAACAGTGCTCACTAT |
| UP00022\_1 | 16 | CCCCCCCCCCCACTTG |
| UP00102\_1 | 14 | CACCCCCGGGGGGG |
| UP00057\_1 | 15 | CCCCCCCGGGGGGGT |
| UP00006\_1 | 15 | CCCCCCCGGGGGGGT |
| UP00026\_1 | 17 | TACATGTGCACATAAAA |
| UP00078\_2 | 15 | ACCCGTATCAAATTT |
| UP00059\_2 | 17 | CGTACAATACGAAATAA |
| UP00099\_2 | 16 | CTATCCCCGCCCTATT |
| UP00020\_2 | 14 | GAATGACGAATAAC |
| UP00012\_2 | 17 | TGATTGTTAACAGTTGG |
| UP00043\_2 | 16 | ATCCCCGCCCCTAAAA |
| UP00050\_2 | 23 | TGTCGTTACACGTGGAAGGCGGT |
| UP00001\_2 | 17 | CGTTCGGCGCCAAAAGG |
| UP00003\_2 | 17 | CGCTCGGCGCCAAAAGC |
| UP00007\_2 | 16 | TGCGGAGTGGGACTGG |
| UP00015\_2 | 16 | TAGTATTTCCGATCTT |
| UP00407\_2 | 17 | GTTCAAAAAAAAAATTC |
| UP00068\_2 | 16 | GCGGAGGTGTCGCCTC |
| UP00079\_2 | 17 | GGCGAGGGGTCAAGGGC |
| UP00073\_2 | 15 | AAAAATAACAAACGG |
| UP00041\_2 | 15 | ATGTCACAACAACAC |
| UP00039\_2 | 17 | AACACCAAAACAAAGGA |
| UP00025\_2 | 15 | CAAACAACAACACCT |
| UP00061\_2 | 16 | ATATCAAAACAAAACA |
| UP00408\_2 | 16 | CCGTCTTCCCCCTCAC |
| UP00032\_2 | 22 | TTTTGTAGATTTTATCGACTTA |
| UP00080\_2 | 17 | GACAGAGATATCAGTTT |
| UP00100\_2 | 17 | GCGGCGATATCGCAGCG |
| UP00070\_2 | 17 | TGCGCATAGGGGAGGAG |
| UP00024\_2 | 14 | AATATTAATAAAGA |
| UP00042\_2 | 16 | AGCGGCACACACGCAA |
| UP00084\_2 | 16 | TGGGCGACGTCGTTAA |
| UP00055\_2 | 17 | TGTTCCCATTGTGTACT |
| UP00035\_2 | 16 | GGGTGTGCCCAAAAGG |
| UP00066\_2 | 16 | TGCAAAAGTCCAATAT |
| UP00391\_2 | 14 | AAAAACCATTAAGG |
| UP00072\_2 | 16 | ATGGAAAGTCGTAAAA |
| UP00086\_2 | 14 | GGAGAAAGGTGCGA |
| UP00018\_2 | 15 | AGTATTCTCGGTTGC |
| UP00040\_2 | 15 | TTGATCGAGAATTCC |
| UP00011\_2 | 15 | ACCACTCTCGGTCAC |
| UP00074\_2 | 14 | GCAAAACATTACTA |
| UP00103\_2 | 16 | ATTGATGAGTCACCAA |
| UP00093\_2 | 17 | AAGCATACGCCCAACTT |
| UP00067\_2 | 16 | GAAGATCAATCACTTA |
| UP00045\_2 | 15 | CAATTGCAAAAATAT |
| UP00044\_2 | 15 | GAAAAAATTGCAAGG |
| UP00060\_2 | 14 | GTGCCACGCGACTG |
| UP00097\_2 | 14 | AAATAAGAAAAAAC |
| UP00092\_2 | 16 | CGACCAACTGCCATGC |
| UP00081\_2 | 15 | CGACCAACTGCCGTG |
| UP00036\_2 | 15 | AGCAACAGCCGCACC |
| UP00017\_2 | 17 | ACTCCAAGTACTTGGAA |
| UP00009\_2 | 16 | CGCGCCGGGTCACGTA |
| UP00027\_2 | 16 | ACATGCTACCTAATAC |
| UP00052\_2 | 16 | ACTTGCTACCTACACC |
| UP00088\_2 | 17 | GCTGGGGGGTACCCCTT |
| UP00048\_2 | 16 | AGAGCGGGGTCAAGTA |
| UP00098\_2 | 23 | ACTGACGCTTGGTTACCACAAAG |
| UP00056\_2 | 15 | TACCCTAGTTACCGA |
| UP00076\_2 | 17 | CTACTTGGATACGGAAT |
| UP00053\_2 | 16 | TCGCGAAGGTTGTACT |
| UP00085\_2 | 14 | CAAATTCCGGAACC |
| UP00008\_2 | 17 | ATGGGATATATCCGCCT |
| UP00000\_2 | 17 | TACGCCCCGCCACTCTG |
| UP00030\_2 | 14 | AAAATTGTTATGAA |
| UP00101\_2 | 16 | AAATAGACAAAGGAAT |
| UP00096\_2 | 17 | GTATTGGGTGGGTATTT |
| UP00004\_2 | 15 | CTCACACAATGGCGC |
| UP00075\_2 | 15 | TTGAATGAAATTCGA |
| UP00014\_2 | 17 | GACCACATTCATACAAT |
| UP00064\_2 | 16 | GGACTGAATTCATGCC |
| UP00069\_2 | 15 | CTATAATTGTTATCG |
| UP00071\_2 | 17 | CATCAATTGTTCCGCTA |
| UP00023\_2 | 16 | TAAGATTATAATACGG |
| UP00062\_2 | 17 | GGAAAAATTGTTAGGAA |
| UP00091\_2 | 15 | TATCATAATTAAGGA |
| UP00034\_2 | 22 | GTGCTAATTGTGTGTGTACGCT |
| UP00051\_2 | 14 | ACATTCATGACACG |
| UP00049\_2 | 15 | TCCGTCGCTTAAAAG |
| UP00002\_2 | 15 | CAAAGGCGTGGCCAG |
| UP00406\_2 | 16 | GATAACATCCTAGTAG |
| UP00077\_2 | 17 | GTTAAAAAAAAAAATTT |
| UP00016\_2 | 17 | TCACGGAACAATAGGTG |
| UP00029\_2 | 15 | CCGATTTAAGCGATC |
| UP00089\_2 | 14 | TTGCCCGGATTAGG |
| UP00058\_2 | 15 | AGCCGAAAAAAAAAT |
| UP00054\_2 | 15 | CCGTATTATAAACAA |
| UP00083\_2 | 16 | GAAGATCAATCACTAA |
| UP00005\_2 | 14 | TCACCTCTGGGCAG |
| UP00010\_2 | 15 | ATTGCCTCAGGCAAT |
| UP00087\_2 | 14 | CCGCCCAAGGGCAG |
| UP00028\_2 | 14 | TACTGGAAAAAAAA |
| UP00046\_2 | 17 | AAGGCCAGATGGTCCGG |
| UP00019\_2 | 15 | TATCATTAGAACGCT |
| UP00031\_2 | 16 | CAATCACTGGCAGAAT |
| UP00047\_2 | 17 | CTTAAGACCACCATTAC |
| UP00037\_2 | 17 | GTGGTTCAATAATTTTG |
| UP00094\_2 | 14 | TGTATATATATACC |
| UP00065\_2 | 14 | GCCGCGCAGTGCGT |
| UP00082\_2 | 16 | GAGCCCTTGTCCCTTG |
| UP00021\_2 | 17 | AGGAGACCCCCAATTTG |
| UP00033\_2 | 17 | TCACCCCGCCCCTAATT |
| UP00095\_2 | 17 | TACGAGACTCCTCTAAC |
| UP00022\_2 | 17 | AAATTCCCCCCGGAAGT |
| UP00102\_2 | 15 | CCACACAGCAGGAGA |
| UP00057\_2 | 15 | CCACACAGCAGGAGA |
| UP00006\_2 | 15 | GAGCACAGCAGGACA |
| UP00026\_2 | 16 | CGAAGCACACAAAATA |
| UP00108\_1 | 17 | TAAACTAATTAGCTGAG |
| UP00187\_1 | 17 | CGCATTAATTAATTACC |
| UP00152\_1 | 17 | GTCCATTAATTAATGGA |
| UP00228\_1 | 17 | CATAACCACTTAACAAC |
| UP00166\_1 | 16 | AACAACCAATTAATTC |
| UP00145\_1 | 16 | AAAAACCAATTAAGAA |
| UP00181\_1 | 16 | AAAGTAATTAGTGAAT |
| UP00151\_1 | 16 | TAAGTAATTAGTTATA |
| UP00138\_1 | 16 | CAGGTAATTACCTCAG |
| UP00209\_1 | 17 | CGAATTAATTAATCACC |
| UP00209\_2 | 17 | CGCATTAATTAATTGGC |
| UP00240\_1 | 16 | TAAGGTAATAAAATTA |
| UP00133\_1 | 16 | AACGGTAATAAAATTT |
| UP00198\_1 | 14 | ATGATCGAATCAAA |
| UP00176\_1 | 16 | CGTTGGGGATTAGCCT |
| UP00219\_1 | 17 | ACCGGTTGATCACCTGA |
| UP00219\_2 | 15 | TAATGATGATCACTA |
| UP00255\_1 | 17 | TAATTAATTAATAATTA |
| UP00218\_1 | 16 | TTTAATTAATTAATTC |
| UP00202\_1 | 14 | CTGAGGTAATTAAT |
| UP00126\_1 | 16 | GGAATAATTACTTCAG |
| UP00154\_1 | 17 | TCGCGATAATTACCGAC |
| UP00110\_1 | 17 | TCGCTATAATTACCGAC |
| UP00230\_1 | 16 | GGGGTAATTAGCTCTG |
| UP00111\_1 | 17 | TGAACCGGATTAATGAA |
| UP00232\_1 | 17 | TAAATAGATACCCCATA |
| UP00143\_1 | 17 | GGAAGGGATTAATTATC |
| UP00227\_1 | 17 | CGACCCAATCAACGGTG |
| UP00201\_1 | 17 | ACCACTAATTAGTGGAC |
| UP00167\_1 | 16 | GCGAACTAATTAATGC |
| UP00163\_1 | 17 | TGCACTAATTAGTGGAA |
| UP00251\_1 | 17 | ATCCATTAATTAATTGA |
| UP00162\_1 | 17 | AGAACTAATTAGTGGAC |
| UP00132\_1 | 17 | CACCGCTAATTAGCGTT |
| UP00204\_1 | 17 | TGCCACTAATTAGTGTA |
| UP00131\_1 | 17 | AGCGCTAATTAGCGATT |
| UP00112\_1 | 17 | AATCGTTAATCCCTTTA |
| UP00127\_1 | 16 | AGGTTAATTAGCTGAT |
| UP00148\_1 | 17 | AAGGCGAAATCATCGCA |
| UP00225\_1 | 15 | CCATAATTAATTACA |
| UP00123\_1 | 16 | GTACTAATTAGTGGCG |
| UP00161\_1 | 17 | GAAAACTAGTTAACATC |
| UP00104\_1 | 17 | ACAAGCAATTAATGAAT |
| UP00155\_1 | 17 | ACAAGCAATTAAAGAAT |
| UP00157\_1 | 17 | ACAAGCAATTAAAGAAT |
| UP00114\_1 | 17 | AAAACATCGTTTTTAAG |
| UP00264\_1 | 16 | CTGAGCTAATTACCGT |
| UP00217\_1 | 16 | TAGGTAATAAAATTCA |
| UP00246\_1 | 16 | TAAAGTCGTAAAACAT |
| UP00183\_1 | 16 | AAAGCTCGTAAAATTT |
| UP00174\_1 | 16 | AAGGTAATTAGCTCAT |
| UP00391\_3 | 14 | TTGAGGTAATTAGT |
| UP00196\_1 | 17 | GATTATTAATTAACTTG |
| UP00189\_1 | 16 | ACGGTAATTAGCTCAG |
| UP00182\_1 | 16 | AAGGTAATTACCTAAT |
| UP00164\_1 | 17 | CGAGTTAATTAATAAGC |
| UP00164\_2 | 16 | GTAGTAATTAATGGAA |
| UP00213\_1 | 17 | ACGGCCATAAAATTAAT |
| UP00134\_1 | 16 | AACCCAATAAAATTCG |
| UP00137\_1 | 17 | TGAGCTAATTAGTTGGA |
| UP00144\_1 | 17 | CGCGTTAATTAATTACC |
| UP00214\_1 | 16 | ACGGTAATTAGCTCAT |
| UP00259\_1 | 16 | TATTGGTAATTACCTT |
| UP00206\_1 | 16 | GTAGTAATTAATGCAA |
| UP00263\_1 | 16 | ACCGGCAATTAATAAA |
| UP00207\_1 | 16 | GGAGCCATAAAATTCG |
| UP00245\_1 | 16 | TAAAGTCGTAAAACGT |
| UP00235\_1 | 16 | TAAAGTCGTAAAATAG |
| UP00135\_1 | 17 | TTAGGTCGTAAAATTTC |
| UP00173\_1 | 16 | AAAGCTCGTAAAATTT |
| UP00113\_1 | 17 | CGAATTAATTAACAATA |
| UP00252\_1 | 17 | CGAATTAATTAATTACT |
| UP00260\_1 | 17 | CAAATTAATTAATAAAA |
| UP00242\_1 | 16 | TTGGGGTAATTAACGT |
| UP00197\_1 | 16 | GGAGGTCATTAATTAT |
| UP00140\_1 | 17 | TAAACTAATTAGCTGTA |
| UP00121\_1 | 17 | AATGCAATAAAATTTAT |
| UP00117\_1 | 17 | TAAGGTCGTAAAATCCT |
| UP00177\_1 | 17 | CAAGGTCGTAAAATCTT |
| UP00180\_1 | 16 | CTACCAATAAAATTCT |
| UP00241\_1 | 16 | TTGAGTTAATTAACCT |
| UP00168\_1 | 17 | TAATTAATTAATGGCTA |
| UP00124\_1 | 16 | AAGGTAATTAGCTCAT |
| UP00236\_1 | 17 | TAAATACATGTAAAATT |
| UP00223\_1 | 17 | AAAATACATGTAATACT |
| UP00223\_2 | 17 | AATATACATGTAATATT |
| UP00194\_1 | 17 | AATATACATGTAAAACA |
| UP00250\_1 | 17 | TATATACATGTAAAATT |
| UP00150\_1 | 17 | AAAATACATGTAAAAAT |
| UP00170\_1 | 16 | CAAAATCAATTAATTT |
| UP00243\_1 | 16 | ACTCCTAATTAGTCGT |
| UP00120\_1 | 17 | TGCATTAATTAATGCGA |
| UP00262\_1 | 17 | CGAATTAATTAATAATG |
| UP00115\_1 | 17 | TAAACTAATTAGTGAAC |
| UP00130\_1 | 17 | GTAATTAATTAAATAAT |
| UP00261\_1 | 17 | TAAACTAATTAGCTTTG |
| UP00212\_1 | 17 | CGAATTAATTAAATACT |
| UP00256\_1 | 17 | GAGCGTTAATTAATGTA |
| UP00256\_2 | 17 | TCCACTAATTAGCGGTT |
| UP00184\_1 | 17 | ACCCCTAATTAGCGGTG |
| UP00175\_1 | 17 | CCCATTAATTAATCACC |
| UP00188\_1 | 17 | CGAATTAATTAAAAACC |
| UP00169\_1 | 17 | AGTTTTTAATTAATTTG |
| UP00186\_1 | 16 | AAGGAGCTGTCAATAC |
| UP00233\_1 | 16 | GAGGTAATTACCTCAG |
| UP00226\_1 | 16 | AAAGACCTGTCAATAC |
| UP00210\_1 | 16 | AATTACCTGTCAATAC |
| UP00234\_1 | 16 | TGCAACTAATTAATTC |
| UP00156\_1 | 17 | GAAGACCAATTAGCGCT |
| UP00171\_1 | 16 | CAAAACCAATTAATTT |
| UP00220\_1 | 17 | TGCGCTAATTAGTGGGA |
| UP00139\_1 | 17 | GTGCACTAATTAGTGCA |
| UP00231\_1 | 17 | TTAACCACTTGAAAATT |
| UP00190\_1 | 16 | CTTTAAGTACTTAATG |
| UP00107\_1 | 16 | TAAGCCACTTGAAATT |
| UP00249\_1 | 16 | TAAGCCACTTGAATTT |
| UP00147\_1 | 16 | TAAGCCACTTAACATT |
| UP00119\_1 | 17 | TTTTAAGTACTTAAATT |
| UP00017\_3 | 17 | TACTAAGTACTTAAATG |
| UP00200\_1 | 17 | GAAAATTAATTACTTCG |
| UP00200\_2 | 16 | AGTAATTAATTACTTC |
| UP00238\_1 | 17 | GATAATTAATTACTTTG |
| UP00216\_1 | 17 | TTAAGGGGATTAACTAC |
| UP00239\_1 | 17 | TGAGGGGGATTAACTAT |
| UP00160\_1 | 17 | TGAGGGGGATTAACTAT |
| UP00208\_1 | 17 | TAGAGGGATTAAATTTC |
| UP00208\_2 | 17 | GATAATTAATCCCTCTT |
| UP00109\_1 | 15 | AAAAACGGATTATTG |
| UP00178\_1 | 17 | CGCGCTAATTAGGTATC |
| UP00237\_1 | 17 | CGTAATTAATTAATTGG |
| UP00229\_1 | 17 | GGAGGGGATTAATTTAT |
| UP00267\_1 | 17 | TGTAGGGATTAATTGTC |
| UP00247\_1 | 17 | TGAACTAATTAGCCCAC |
| UP00224\_1 | 16 | TGATTAATTAATTGAC |
| UP00248\_1 | 17 | CGAACTAATTAGTACTA |
| UP00185\_1 | 17 | TCACCCATCAATAATCA |
| UP00221\_1 | 16 | CAGCATTAATTAGTAG |
| UP00149\_1 | 17 | CGGAATTAATTAATAGG |
| UP00153\_1 | 17 | TTAGAGGGATTAACAAT |
| UP00125\_1 | 17 | TGAAGGGATTAATCATC |
| UP00265\_1 | 16 | AGGGGGATTAGCTGCC |
| UP00203\_1 | 16 | AAAGACCTGTCAATCC |
| UP00205\_1 | 16 | AAGCACCTGTCAATAT |
| UP00158\_1 | 17 | GATTAATTAATTAAGTC |
| UP00254\_1 | 16 | ATGTATTAATTAAGTA |
| UP00191\_1 | 16 | TTGTATGCAAATTAGA |
| UP00179\_1 | 16 | TTGTATGCAAATTAGA |
| UP00129\_1 | 17 | AATTAATTAATTAATTC |
| UP00128\_1 | 17 | GATAATTAATTAGTTTG |
| UP00211\_1 | 17 | AAAATATGCATAATAAA |
| UP00105\_1 | 17 | AATTAATTAATTAATTC |
| UP00118\_1 | 16 | AGTTATTAATGAGGTC |
| UP00146\_1 | 17 | GACGATAATGAGGTTGC |
| UP00146\_2 | 17 | AAACATAATGAGGTTGC |
| UP00172\_1 | 17 | CGAATTAATTAAGAAAC |
| UP00266\_1 | 17 | GTAACTAATTAACTACT |
| UP00136\_1 | 17 | AAAGCTAATTAGCGAAA |
| UP00253\_1 | 17 | TGCACTAATTAGCGCAC |
| UP00193\_1 | 17 | AAGACGCTGTAAAGCGA |
| UP00193\_2 | 17 | AGGACGCTGTAAAGGGA |
| UP00116\_1 | 17 | TGCCTTAATTAATGCTC |
| UP00257\_1 | 17 | CGCGTTAATTAATTGTG |
| UP00192\_1 | 17 | GATGGGGTATCATTTTT |
| UP00159\_1 | 17 | AATGGGGTATCACTTTT |
| UP00195\_1 | 17 | GATAGGGTATCACTTAT |
| UP00199\_1 | 17 | ATAAATGACACCTATCA |
| UP00008\_3 | 17 | AATAGGGTATCAATTAT |
| UP00008\_4 | 17 | AATAGGGTATCAATATT |
| UP00089\_3 | 17 | CCTTAGTTAACTAAAAT |
| UP00222\_1 | 17 | AGCTGTTAACTAGCCGT |
| UP00122\_1 | 17 | GATATTGACAGCTGCGT |
| UP00258\_1 | 16 | AACTAGCTGTCAATAC |
| UP00165\_1 | 16 | TAAGCCACTTGAAATT |
| UP00244\_1 | 17 | TAATTAATTAATAACTT |
| UP00142\_1 | 17 | CATAATTAATTAACGCG |
| UP00215\_1 | 16 | ACGTTAATTAACCCAG |
| UP00106\_1 | 16 | GTGCACTAATTAAGAC |
| UP00141\_1 | 17 | CGAGTTAATTAATAATT |

Random model letter frequencies
(from ./background):
  
A 0.241 C 0.259 G 0.259 T 0.241

---

**SECTION I: HIGH-SCORING MOTIF OCCURRENCES**


---

- There were
  469
  motif occurrences with a
  p-value less than
  0.0001.
- The p-value of a motif occurrence is defined as the
  probability of a random sequence of the same length as the motif
  matching that position of the sequence with as good or better a score.
- The score for the match of a position in a sequence to a motif
  is computed by summing the appropriate entries from each column of
  the position-dependent scoring matrix that represents the motif.
- The q-value of a motif occurrence is defined as the
  false discovery rate if the occurrence is accepted as significant.
- The table is sorted by increasing p-value.

| Motif | Sequence Name | Strand | Start | End | p-value | q-value | Matched Sequence |
| --- | --- | --- | --- | --- | --- | --- | --- |
| UP00048\_1 | chr16 | − | 27320854 | 27320869 | 1.18e-07 | 0.252 | `CACCAAAGGTCACCTG` |
| UP00048\_1 | chr12 | − | 115481660 | 115481675 | 2.69e-07 | 0.287 | `TCTCAAAGGTCATGAT` |
| UP00048\_1 | chr5 | − | 138995992 | 138996007 | 5.3e-07 | 0.318 | `TCCTAAAGGTCACCCA` |
| UP00048\_1 | chr18 | + | 58979355 | 58979370 | 7.72e-07 | 0.318 | `TTGAAAAGGTCACCTC` |
| UP00048\_1 | chr20 | − | 55184001 | 55184016 | 7.72e-07 | 0.318 | `TCTGAAAGGTCATCAG` |
| UP00048\_1 | chr10 | + | 64067471 | 64067486 | 1.24e-06 | 0.318 | `TCTTAAAGGTCATGGT` |
| UP00048\_1 | chr16 | + | 11735572 | 11735587 | 1.31e-06 | 0.318 | `TTTCAAAGGTCATAAT` |
| UP00048\_1 | chr17 | − | 63077717 | 63077732 | 1.4e-06 | 0.318 | `CTTAAAAGGTCAACTG` |
| UP00048\_1 | chr12 | − | 29196561 | 29196576 | 1.49e-06 | 0.318 | `TACAAAAGGTCACTAG` |
| UP00048\_1 | chr1 | + | 76034554 | 76034569 | 1.53e-06 | 0.318 | `TTCTAAAGGTCACCAC` |
| UP00048\_1 | chr20 | + | 36896591 | 36896606 | 1.68e-06 | 0.318 | `TTTTAAAGGTCATCCA` |
| UP00048\_1 | chr14 | − | 99601828 | 99601843 | 1.89e-06 | 0.318 | `CATTAAAGGTCACAGG` |
| UP00048\_1 | chr10 | − | 14742074 | 14742089 | 1.95e-06 | 0.318 | `TGCTAAAGGTCACCTA` |
| UP00048\_1 | chr16 | − | 66666503 | 66666518 | 2.14e-06 | 0.318 | `TTGTAAAGGTCACCCC` |
| UP00048\_1 | chr16 | − | 70408083 | 70408098 | 2.34e-06 | 0.318 | `CTCTAAAGGTCACATG` |
| UP00048\_1 | chrX | + | 7043734 | 7043749 | 2.61e-06 | 0.318 | `TCTGAAAGGTCACTGT` |
| UP00048\_1 | chr11 | − | 34222414 | 34222429 | 2.66e-06 | 0.318 | `TTTCAAAGGTCAGGGG` |
| UP00048\_1 | chr15 | + | 72879849 | 72879864 | 2.82e-06 | 0.318 | `AACCAAAGGTCATGGA` |
| UP00048\_1 | chr4 | − | 79779230 | 79779245 | 3.13e-06 | 0.318 | `TCACAGAGGTCACCTG` |
| UP00048\_1 | chr10 | + | 112145783 | 112145798 | 3.18e-06 | 0.318 | `GGGCAAAGGTCATGTG` |
| UP00048\_1 | chr22 | − | 46762849 | 46762864 | 3.22e-06 | 0.318 | `GCCTAAAGGTCAAGGG` |
| UP00048\_1 | chr9 | + | 126002485 | 126002500 | 3.27e-06 | 0.318 | `CCCCAAAGGTCATGGA` |
| UP00048\_1 | chr19 | − | 1334731 | 1334746 | 3.68e-06 | 0.33 | `GCGGAAAGGTCATCGG` |
| UP00048\_1 | chr4 | + | 88068934 | 88068949 | 3.71e-06 | 0.33 | `TTCTAAAGGTCACTAT` |
| UP00048\_1 | chr8 | − | 126519976 | 126519991 | 4.11e-06 | 0.332 | `TGCCAAAGGTCATCGA` |
| UP00048\_1 | chr1 | − | 39229122 | 39229137 | 4.49e-06 | 0.332 | `TGTTAAAGGTCATCAA` |
| UP00048\_1 | chr12 | + | 67488809 | 67488824 | 4.53e-06 | 0.332 | `GGGTAAAGGTCACGGG` |
| UP00048\_1 | chr6 | + | 30189024 | 30189039 | 4.56e-06 | 0.332 | `ACTGAAAGGTCACAGC` |
| UP00048\_1 | chr11 | + | 72582589 | 72582604 | 4.91e-06 | 0.332 | `TCTGAGAGGTCACCAT` |
| UP00048\_1 | chr10 | + | 96986307 | 96986322 | 5.03e-06 | 0.332 | `AATGAAAGGTCATGAA` |
| UP00048\_1 | chr16 | − | 55663376 | 55663391 | 5.11e-06 | 0.332 | `AGTAAAAGGTCATGTA` |
| UP00048\_1 | chr5 | − | 40864421 | 40864436 | 5.23e-06 | 0.332 | `GCCAAGAGGTCACGAG` |
| UP00048\_1 | chr10 | − | 89860772 | 89860787 | 5.27e-06 | 0.332 | `CATGAAAGGTCACTGG` |
| UP00048\_1 | chr10 | + | 112118232 | 112118247 | 5.69e-06 | 0.332 | `GTTGAAAGGTCAAGAT` |
| UP00048\_1 | chr17 | − | 39781438 | 39781453 | 5.74e-06 | 0.332 | `CCCCAGAGGTCACCAT` |
| UP00048\_1 | chr8 | − | 96219453 | 96219468 | 6.14e-06 | 0.332 | `CAGCAAAGGTCAAGGA` |
| UP00048\_1 | chr7 | + | 28487745 | 28487760 | 6.19e-06 | 0.332 | `TTAAAAAGGTCACACA` |
| UP00048\_1 | chr6 | − | 80416348 | 80416363 | 6.24e-06 | 0.332 | `TTAAAAAGGTCAAGAG` |
| UP00048\_1 | chr2 | + | 204600357 | 204600372 | 6.42e-06 | 0.332 | `GCTTAGAGGTCACATT` |
| UP00048\_1 | chr9 | + | 70787890 | 70787905 | 6.66e-06 | 0.332 | `TTGCAAAGGTCAAAAC` |
| UP00048\_1 | chr3 | − | 53112145 | 53112160 | 7e-06 | 0.332 | `GAGCAAAGGTCATTAA` |
| UP00048\_1 | chr3 | − | 13349038 | 13349053 | 7.15e-06 | 0.332 | `TCTTAAAGGTCAGCCC` |
| UP00048\_1 | chrX | + | 48679659 | 48679674 | 7.36e-06 | 0.332 | `ATGGAAAGGTCAACGG` |
| UP00048\_1 | chr4 | + | 25694981 | 25694996 | 7.47e-06 | 0.332 | `CTTAAAAGGTCAAGAC` |
| UP00048\_1 | chr1 | + | 36381750 | 36381765 | 7.52e-06 | 0.332 | `TCATAAAGGTCAACCA` |
| UP00048\_1 | chr3 | − | 40977894 | 40977909 | 7.58e-06 | 0.332 | `TCAGAAAGGTCACAAA` |
| UP00048\_1 | chr20 | − | 47337877 | 47337892 | 7.58e-06 | 0.332 | `TTGTAAAGGTCAGCAG` |
| UP00048\_1 | chr16 | + | 1981213 | 1981228 | 7.73e-06 | 0.332 | `CCTCAGAGGTCAACGG` |
| UP00048\_1 | chr19 | + | 12266621 | 12266636 | 7.84e-06 | 0.332 | `GAACAGAGGTCACCAG` |
| UP00048\_1 | chr19 | + | 16343339 | 16343354 | 7.84e-06 | 0.332 | `GTGAAAAGGTCATTAG` |
| UP00048\_1 | chr6 | + | 44333465 | 44333480 | 8.36e-06 | 0.332 | `TCCCAGAGGTCATGGT` |
| UP00048\_1 | chr11 | − | 1832949 | 1832964 | 8.54e-06 | 0.332 | `TCTGAAAGGTCAGGCT` |
| UP00048\_1 | chr19 | − | 52153948 | 52153963 | 8.54e-06 | 0.332 | `CTGTAAAGGTCAAATG` |
| UP00048\_1 | chr9 | + | 93010356 | 93010371 | 8.59e-06 | 0.332 | `TTGGAAAGGTCAAGAA` |
| UP00048\_1 | chr5 | − | 78089924 | 78089939 | 8.65e-06 | 0.332 | `GGTTAAAGGTCATAGG` |
| UP00048\_1 | chr8 | − | 71021150 | 71021165 | 8.71e-06 | 0.332 | `CCTGAAAGGTCAACGC` |
| UP00048\_1 | chr19 | − | 55536282 | 55536297 | 9.01e-06 | 0.338 | `TCTTAGAGGTCAAATG` |
| UP00048\_1 | chr3 | − | 196349895 | 196349910 | 9.33e-06 | 0.343 | `TTTAAAAGTTCACCAC` |
| UP00048\_1 | chr8 | − | 61984399 | 61984414 | 9.57e-06 | 0.343 | `GATGAAAGGTCAATTA` |
| UP00048\_1 | chr5 | − | 5414352 | 5414367 | 9.77e-06 | 0.343 | `AACCAGAGGTCAACAG` |
| UP00048\_1 | chr3 | − | 157886305 | 157886320 | 1.01e-05 | 0.343 | `AAGCAAAGTTCACCCT` |
| UP00048\_1 | chr5 | + | 139205095 | 139205110 | 1.06e-05 | 0.343 | `CCAGAAAGGTCACACG` |
| UP00048\_1 | chr4 | + | 185973704 | 185973719 | 1.07e-05 | 0.343 | `TCGCAGAGGTCACAGC` |
| UP00048\_1 | chr17 | − | 57526189 | 57526204 | 1.07e-05 | 0.343 | `AGGGAAAGGTCATGTG` |
| UP00048\_1 | chr22 | + | 36010476 | 36010491 | 1.07e-05 | 0.343 | `GAGAAAAGGTCATTTC` |
| UP00048\_1 | chr1 | + | 36381741 | 36381756 | 1.08e-05 | 0.343 | `TCATAAAGGTCATAAA` |
| UP00048\_1 | chr9 | − | 24923031 | 24923046 | 1.08e-05 | 0.343 | `CTTCAAAGTTCAACTG` |
| UP00048\_1 | chr12 | + | 122170645 | 122170660 | 1.11e-05 | 0.343 | `GATTTAAGGTCACCTT` |
| UP00048\_1 | chr15 | − | 73122644 | 73122659 | 1.11e-05 | 0.343 | `ACCCAAAGGTCAGGCC` |
| UP00048\_1 | chr5 | + | 139205129 | 139205144 | 1.13e-05 | 0.345 | `ACACAAAGGTCAATGG` |
| UP00048\_1 | chr3 | − | 14668140 | 14668155 | 1.18e-05 | 0.351 | `TTGGAAAGGTCATAGA` |
| UP00048\_1 | chr19 | − | 2036308 | 2036323 | 1.19e-05 | 0.351 | `TTCCAAGGGTCACTTG` |
| UP00048\_1 | chr3 | − | 15378081 | 15378096 | 1.22e-05 | 0.351 | `CTTCAAAGTTCACATA` |
| UP00048\_1 | chr8 | − | 135681867 | 135681882 | 1.23e-05 | 0.351 | `TCTTAAAGTTCACACA` |
| UP00048\_1 | chr2 | − | 179103632 | 179103647 | 1.29e-05 | 0.351 | `CAAAAGAGGTCACCTG` |
| UP00048\_1 | chr1 | − | 38713325 | 38713340 | 1.3e-05 | 0.351 | `CATAAAAGGTCAGAGG` |
| UP00048\_1 | chr2 | − | 230830899 | 230830914 | 1.3e-05 | 0.351 | `CACTAAAGGTCAGCTA` |
| UP00048\_1 | chr20 | − | 57013447 | 57013462 | 1.32e-05 | 0.351 | `ATAGAAAGGTCAACAG` |
| UP00048\_1 | chr3 | − | 13104825 | 13104840 | 1.33e-05 | 0.351 | `GAGCAGAGGTCAAGCG` |
| UP00048\_1 | chr19 | + | 44584208 | 44584223 | 1.35e-05 | 0.351 | `AACCAAAGTTCATCGG` |
| UP00048\_1 | chr17 | − | 16130096 | 16130111 | 1.36e-05 | 0.351 | `GAGCAAAGGTCAGTAG` |
| UP00048\_1 | chr7 | + | 23359454 | 23359469 | 1.37e-05 | 0.351 | `CCAAAAAGGTCACTGA` |
| UP00048\_1 | chr3 | + | 6277315 | 6277330 | 1.38e-05 | 0.351 | `AAGCAGAGGTCATCTC` |
| UP00048\_1 | chr8 | − | 81105049 | 81105064 | 1.4e-05 | 0.351 | `GGTCAAAGGTCAGAGG` |
| UP00048\_1 | chr2 | + | 25048009 | 25048024 | 1.43e-05 | 0.351 | `ACGGAAAGGTCAAAAA` |
| UP00048\_1 | chr13 | + | 47614828 | 47614843 | 1.43e-05 | 0.351 | `CCTGAAAGGTCATTGA` |
| UP00048\_1 | chr13 | + | 97939031 | 97939046 | 1.44e-05 | 0.351 | `TGGCAGAGGTCACACG` |
| UP00048\_1 | chr15 | + | 80365925 | 80365940 | 1.45e-05 | 0.351 | `TCTGAAAGTTCAACAG` |
| UP00048\_1 | chr16 | + | 27369624 | 27369639 | 1.47e-05 | 0.351 | `CCCCAAAGGTCAGACT` |
| UP00048\_1 | chr3 | + | 48909699 | 48909714 | 1.5e-05 | 0.351 | `GTGCAGAGGTCATCCA` |
| UP00048\_1 | chr3 | + | 116349182 | 116349197 | 1.51e-05 | 0.351 | `CTGAAAAGGTCAATCG` |
| UP00048\_1 | chr2 | + | 158004750 | 158004765 | 1.52e-05 | 0.351 | `GCTCAAAGTTCAACAC` |
| UP00048\_1 | chr17 | + | 63800089 | 63800104 | 1.53e-05 | 0.351 | `ACCCCAAGGTCACCGT` |
| UP00048\_1 | chr5 | + | 106935123 | 106935138 | 1.58e-05 | 0.351 | `TACCAAGGGTCATCCT` |
| UP00048\_1 | chr14 | + | 23700574 | 23700589 | 1.61e-05 | 0.351 | `AGGCAAAGGTCAGGAG` |
| UP00048\_1 | chr9 | + | 113787626 | 113787641 | 1.62e-05 | 0.351 | `ACTCACAGGTCACCTG` |
| UP00048\_1 | chrX | + | 141893745 | 141893760 | 1.62e-05 | 0.351 | `ACCCAGAGGTCAAGCA` |
| UP00048\_1 | chr16 | + | 11743045 | 11743060 | 1.63e-05 | 0.351 | `GCAAAAAGTTCACCCG` |
| UP00048\_1 | chrX | + | 46373438 | 46373453 | 1.65e-05 | 0.351 | `TTCAAAAGGTCAGTGG` |
| UP00048\_1 | chr17 | − | 60412703 | 60412718 | 1.65e-05 | 0.351 | `CCGTAGAGGTCACAAG` |
| UP00048\_1 | chr7 | + | 100515544 | 100515559 | 1.66e-05 | 0.351 | `TCCTAGAGGTCAAGAT` |
| UP00048\_1 | chr17 | − | 20542992 | 20543007 | 1.7e-05 | 0.356 | `AGGGAAAGGTCACTGG` |
| UP00048\_1 | chr19 | − | 2379054 | 2379069 | 1.73e-05 | 0.357 | `GGAAAAAGGTCACTGG` |
| UP00048\_1 | chr16 | − | 56135776 | 56135791 | 1.74e-05 | 0.357 | `TCAGAGAGGTCACCAT` |
| UP00048\_1 | chr15 | + | 42028612 | 42028627 | 1.76e-05 | 0.357 | `CAGCAAGGGTCACCAA` |
| UP00048\_1 | chr11 | − | 68580547 | 68580562 | 1.8e-05 | 0.357 | `CCCCAGAGGTCACAGC` |
| UP00048\_1 | chr10 | − | 115732599 | 115732614 | 1.81e-05 | 0.357 | `CCTCCAAGGTCATCTT` |
| UP00048\_1 | chr7 | + | 130459590 | 130459605 | 1.82e-05 | 0.357 | `GTCAAGAGGTCACAGA` |
| UP00048\_1 | chr16 | − | 8937712 | 8937727 | 1.82e-05 | 0.357 | `GATGAGAGGTCAAGAG` |
| UP00048\_1 | chr15 | + | 38191447 | 38191462 | 1.88e-05 | 0.361 | `TCTCTAAGGTCAAGCA` |
| UP00048\_1 | chr1 | − | 148801703 | 148801718 | 1.9e-05 | 0.361 | `TTTCAGAGGTCAGCGA` |
| UP00048\_1 | chr6 | − | 27215552 | 27215567 | 1.91e-05 | 0.361 | `GGCCCAAGGTCACCTG` |
| UP00048\_1 | chr2 | − | 161268852 | 161268867 | 1.93e-05 | 0.361 | `CCACAGAGGTCACGGA` |
| UP00048\_1 | chr12 | + | 107486035 | 107486050 | 1.95e-05 | 0.361 | `AAAGAAAGGTCATCAC` |
| UP00048\_1 | chr6 | − | 36828843 | 36828858 | 1.97e-05 | 0.361 | `TTTTGAAGGTCACCCT` |
| UP00048\_1 | chr9 | + | 91269422 | 91269437 | 1.97e-05 | 0.361 | `TCACAGAGGTCAACCA` |
| UP00048\_1 | chr2 | − | 125872047 | 125872062 | 1.98e-05 | 0.361 | `CCTGAGAGGTCACTTT` |
| UP00048\_1 | chr3 | − | 158289268 | 158289283 | 1.99e-05 | 0.361 | `CTTCTAAGGTCACGGA` |
| UP00048\_1 | chr11 | − | 101693074 | 101693089 | 2.04e-05 | 0.364 | `TGGAAAAGGTCAATAT` |
| UP00048\_1 | chr19 | − | 3136495 | 3136510 | 2.04e-05 | 0.364 | `CACAAAGGGTCACGGG` |
| UP00048\_1 | chr6 | − | 30569853 | 30569868 | 2.08e-05 | 0.365 | `GTTAAAGGGTCATGGG` |
| UP00048\_1 | chr7 | + | 5701238 | 5701253 | 2.08e-05 | 0.365 | `ACCCAAAGTTCATATA` |
| UP00048\_1 | chr10 | − | 112593010 | 112593025 | 2.11e-05 | 0.365 | `AAGGAGAGGTCACAAG` |
| UP00048\_1 | chr12 | + | 123961348 | 123961363 | 2.12e-05 | 0.365 | `ATCCAGAGGTCAATTG` |
| UP00048\_1 | chr11 | − | 33662628 | 33662643 | 2.15e-05 | 0.367 | `GTACAGAGGTCATGAG` |
| UP00048\_1 | chr7 | + | 24924318 | 24924333 | 2.2e-05 | 0.372 | `CCTGAGAGGTCAAGAG` |
| UP00048\_1 | chr17 | − | 33853759 | 33853774 | 2.21e-05 | 0.372 | `TCTCTAAGGTCAGCAG` |
| UP00048\_1 | chr2 | + | 9838038 | 9838053 | 2.27e-05 | 0.373 | `ACTGAAAGTTCACTTT` |
| UP00048\_1 | chr6 | + | 106737094 | 106737109 | 2.29e-05 | 0.373 | `ATGAAAAGGTCAGAAT` |
| UP00048\_1 | chr6 | + | 16581627 | 16581642 | 2.31e-05 | 0.373 | `GTGAAAAGGTCAGATC` |
| UP00048\_1 | chr2 | + | 231549798 | 231549813 | 2.33e-05 | 0.373 | `ATCCAGAGGTCATGCC` |
| UP00048\_1 | chr8 | + | 67057151 | 67057166 | 2.35e-05 | 0.373 | `GACTAAAGTTCAACAG` |
| UP00048\_1 | chr11 | − | 64650955 | 64650970 | 2.35e-05 | 0.373 | `GGTGAAAGGTCAGCAT` |
| UP00048\_1 | chr8 | − | 125693909 | 125693924 | 2.42e-05 | 0.373 | `CCTGAAAGGTCAGTTT` |
| UP00048\_1 | chr11 | − | 65076596 | 65076611 | 2.45e-05 | 0.373 | `TTTCAGGGGTCACATG` |
| UP00048\_1 | chr16 | + | 45510575 | 45510590 | 2.45e-05 | 0.373 | `GCTAAAGGGTCAGCTG` |
| UP00048\_1 | chr1 | + | 111566124 | 111566139 | 2.49e-05 | 0.373 | `CCCAAGAGGTCACTCT` |
| UP00048\_1 | chr12 | − | 127881142 | 127881157 | 2.51e-05 | 0.373 | `TCGCAAGGGTCAAATA` |
| UP00048\_1 | chr8 | + | 105667458 | 105667473 | 2.52e-05 | 0.373 | `TGGCAGAGGTCAACCT` |
| UP00048\_1 | chr19 | + | 62885004 | 62885019 | 2.54e-05 | 0.373 | `TCGCAGAGGTCAAAGC` |
| UP00048\_1 | chr8 | − | 101998431 | 101998446 | 2.55e-05 | 0.373 | `AGGGAAAGGTCAAGAA` |
| UP00048\_1 | chr2 | + | 198073073 | 198073088 | 2.58e-05 | 0.373 | `GCTCAAGGGTCAAATC` |
| UP00048\_1 | chr5 | + | 149764165 | 149764180 | 2.58e-05 | 0.373 | `TGAGAGAGGTCACCTG` |
| UP00048\_1 | chr6 | − | 106664805 | 106664820 | 2.58e-05 | 0.373 | `TGTAAAAGTTCACAAA` |
| UP00048\_1 | chr1 | − | 228391076 | 228391091 | 2.61e-05 | 0.373 | `AAGCAAGGGTCACAGA` |
| UP00048\_1 | chr14 | + | 63405022 | 63405037 | 2.61e-05 | 0.373 | `GGAAAAAGGTCAAATT` |
| UP00048\_1 | chr17 | − | 73652171 | 73652186 | 2.63e-05 | 0.373 | `AGCCAGAGGTCACTGG` |
| UP00048\_1 | chr1 | − | 195123766 | 195123781 | 2.64e-05 | 0.373 | `GCTTTAAGGTCACTTA` |
| UP00048\_1 | chr1 | + | 232899483 | 232899498 | 2.66e-05 | 0.373 | `TGTGAAAGGTCAGATA` |
| UP00048\_1 | chr1 | + | 210172908 | 210172923 | 2.68e-05 | 0.373 | `GCCAAGAGGTCATAGA` |
| UP00048\_1 | chr5 | − | 43639626 | 43639641 | 2.68e-05 | 0.373 | `CCTCAAGGGTCAAGCA` |
| UP00048\_1 | chr3 | − | 45001000 | 45001015 | 2.71e-05 | 0.373 | `GCCACAAGGTCACAGG` |
| UP00048\_1 | chr19 | + | 2558981 | 2558996 | 2.71e-05 | 0.373 | `CCAGAAAGTTCACCTG` |
| UP00048\_1 | chr6 | + | 31343807 | 31343822 | 2.72e-05 | 0.373 | `CAACAAAGGTCAGTTG` |
| UP00048\_1 | chr8 | − | 10839364 | 10839379 | 2.72e-05 | 0.373 | `TTCGAAAGGTCAGTGG` |
| UP00048\_1 | chr1 | + | 159282718 | 159282733 | 2.74e-05 | 0.373 | `TTTGAAAGGTTACCTG` |
| UP00048\_1 | chr12 | + | 92118594 | 92118609 | 2.74e-05 | 0.373 | `CACAAAAGGTCAGTGG` |
| UP00048\_1 | chr3 | − | 72308399 | 72308414 | 2.8e-05 | 0.376 | `TTGCAGAGGTCAGCCT` |
| UP00048\_1 | chr4 | − | 185542473 | 185542488 | 2.82e-05 | 0.376 | `TTTCAGAGTTCACATA` |
| UP00048\_1 | chr6 | − | 250669 | 250684 | 2.84e-05 | 0.376 | `CTCTGAAGGTCACCTG` |
| UP00048\_1 | chr19 | − | 2734432 | 2734447 | 2.85e-05 | 0.376 | `TCGCAGAGGTCAGTTG` |
| UP00048\_1 | chr21 | − | 35159842 | 35159857 | 2.87e-05 | 0.376 | `TGTCAGAGTTCACCAG` |
| UP00048\_1 | chr22 | − | 46762842 | 46762857 | 2.87e-05 | 0.376 | `GGTCAAGGGTCACAGT` |
| UP00048\_1 | chr15 | + | 49308048 | 49308063 | 2.92e-05 | 0.381 | `ATGCAAAGTTCACTGT` |
| UP00048\_1 | chr20 | + | 36937737 | 36937752 | 2.96e-05 | 0.383 | `TTCAGAAGGTCACGCT` |
| UP00048\_1 | chr20 | + | 47835867 | 47835882 | 2.99e-05 | 0.385 | `TTCTAGAGGTCAGCAG` |
| UP00048\_1 | chr19 | − | 54157630 | 54157645 | 3.02e-05 | 0.387 | `TGTCTAAGGTCACTGG` |
| UP00048\_1 | chr16 | − | 80674028 | 80674043 | 3.1e-05 | 0.389 | `AACTAAAGTTCAAGTT` |
| UP00048\_1 | chr11 | − | 61292765 | 61292780 | 3.11e-05 | 0.389 | `CCCAAGAGGTCAACAC` |
| UP00048\_1 | chr2 | − | 122798136 | 122798151 | 3.15e-05 | 0.389 | `CTGAAGAGGTCATCGA` |
| UP00048\_1 | chr6 | + | 237854 | 237869 | 3.15e-05 | 0.389 | `GCCGAGAGGTCAGCTG` |
| UP00048\_1 | chr10 | + | 26816561 | 26816576 | 3.15e-05 | 0.389 | `AGGGAGAGGTCACGAG` |
| UP00048\_1 | chr15 | − | 29342961 | 29342976 | 3.15e-05 | 0.389 | `GCCAAAAGGTCAGTGC` |
| UP00048\_1 | chr16 | + | 55596559 | 55596574 | 3.17e-05 | 0.389 | `CTTGAAAGTTCATCGG` |
| UP00048\_1 | chr1 | − | 108051149 | 108051164 | 3.24e-05 | 0.389 | `TCTGAAAGTTCAGCTT` |
| UP00048\_1 | chr19 | − | 55991456 | 55991471 | 3.24e-05 | 0.389 | `GCTCAAAGGGCACCCG` |
| UP00048\_1 | chr2 | + | 61097594 | 61097609 | 3.28e-05 | 0.389 | `TTGAAAAGTTCATCCC` |
| UP00048\_1 | chr3 | − | 14668105 | 14668120 | 3.3e-05 | 0.389 | `CTGCTAAGGTCATCTT` |
| UP00048\_1 | chr7 | + | 4648147 | 4648162 | 3.32e-05 | 0.389 | `GGTCAGAGGTCAGCGG` |
| UP00048\_1 | chr17 | + | 7328246 | 7328261 | 3.39e-05 | 0.389 | `TTCAAAAGGTCGCCCA` |
| UP00048\_1 | chr20 | + | 36896486 | 36896501 | 3.39e-05 | 0.389 | `TCTGAAAGTTCATAGA` |
| UP00048\_1 | chr18 | − | 17966492 | 17966507 | 3.43e-05 | 0.389 | `TCATAGAGGTCACAGC` |
| UP00048\_1 | chr15 | + | 61757289 | 61757304 | 3.45e-05 | 0.389 | `TCTGAGAGGTCAGCGA` |
| UP00048\_1 | chr17 | + | 71779402 | 71779417 | 3.47e-05 | 0.389 | `ACCCAAAGTTCAATTA` |
| UP00048\_1 | chr18 | − | 7943240 | 7943255 | 3.55e-05 | 0.389 | `CACAAAGGGTCATGAG` |
| UP00048\_1 | chr21 | − | 25880572 | 25880587 | 3.59e-05 | 0.389 | `TCCTAAAGTTCAGCCG` |
| UP00048\_1 | chr7 | + | 97599076 | 97599091 | 3.74e-05 | 0.389 | `TACCAAAGTTCAATGT` |
| UP00048\_1 | chr11 | + | 118071602 | 118071617 | 3.74e-05 | 0.389 | `AAAAAAAGTTCACCAC` |
| UP00048\_1 | chr12 | + | 75061730 | 75061745 | 3.74e-05 | 0.389 | `TCCGAAAGTTCAAGAA` |
| UP00048\_1 | chr9 | − | 114586008 | 114586023 | 3.76e-05 | 0.389 | `AAGCCAAGGTCACTGG` |
| UP00048\_1 | chr14 | − | 93499476 | 93499491 | 3.78e-05 | 0.389 | `CAGCCAAGGTCAAGTG` |
| UP00048\_1 | chr15 | + | 43279789 | 43279804 | 3.78e-05 | 0.389 | `TCTCAAATGTCACAAG` |
| UP00048\_1 | chr10 | − | 35791487 | 35791502 | 3.82e-05 | 0.389 | `TCTGAAAGTTCAATCG` |
| UP00048\_1 | chr1 | − | 114156840 | 114156855 | 3.84e-05 | 0.389 | `GAGGAAGGGTCACAGG` |
| UP00048\_1 | chr6 | − | 45996849 | 45996864 | 3.86e-05 | 0.389 | `CTAGAAAGGTCACTGC` |
| UP00048\_1 | chr17 | + | 34235097 | 34235112 | 3.86e-05 | 0.389 | `CTCTAGAGGTCACTTC` |
| UP00048\_1 | chr17 | + | 71775148 | 71775163 | 3.86e-05 | 0.389 | `TCAAAAAGTTCATGGT` |
| UP00048\_1 | chr19 | − | 10566059 | 10566074 | 3.86e-05 | 0.389 | `TGGCAGAGGTCAGCAG` |
| UP00048\_1 | chr6 | − | 47513522 | 47513537 | 3.91e-05 | 0.389 | `TGACAGAGGTCATCAA` |
| UP00048\_1 | chr8 | − | 131329418 | 131329433 | 3.91e-05 | 0.389 | `TTTTAAAGGGCACCTG` |
| UP00048\_1 | chr17 | + | 73635093 | 73635108 | 3.91e-05 | 0.389 | `ACACAGAGGTCACTCC` |
| UP00048\_1 | chr17 | + | 3561018 | 3561033 | 3.93e-05 | 0.389 | `ATCCAAAGTTCAGCGG` |
| UP00048\_1 | chr17 | + | 59274292 | 59274307 | 3.93e-05 | 0.389 | `GGTCTAAGGTCACTTA` |
| UP00048\_1 | chr1 | − | 87344547 | 87344562 | 3.95e-05 | 0.389 | `TTTAAGAGTTCACAGG` |
| UP00048\_1 | chr2 | + | 28663554 | 28663569 | 3.95e-05 | 0.389 | `AACTAAAGTTCATGCA` |
| UP00048\_1 | chr6 | + | 133181489 | 133181504 | 3.95e-05 | 0.389 | `GAGGAAAGTTCACTTT` |
| UP00048\_1 | chr5 | + | 139204991 | 139205006 | 3.97e-05 | 0.389 | `ACCAAGAGGTCATTCA` |
| UP00048\_1 | chr5 | − | 149764042 | 149764057 | 3.97e-05 | 0.389 | `GCGCCAAGGTCACAGC` |
| UP00048\_1 | chr8 | − | 126780082 | 126780097 | 3.97e-05 | 0.389 | `CCCAAAGGGTCAAATG` |
| UP00048\_1 | chr2 | − | 156965845 | 156965860 | 4e-05 | 0.389 | `CCTCAAAGTTCAATAA` |
| UP00048\_1 | chr10 | − | 126397983 | 126397998 | 4.02e-05 | 0.389 | `GAGAAGAGTTCACCTT` |
| UP00048\_1 | chr2 | + | 65635618 | 65635633 | 4.04e-05 | 0.389 | `AAGAAGAGGTCAAGCC` |
| UP00048\_1 | chr17 | − | 2562060 | 2562075 | 4.04e-05 | 0.389 | `CACCCAAGGTCACAGT` |
| UP00048\_1 | chr18 | + | 19734969 | 19734984 | 4.04e-05 | 0.389 | `CTCCAAAGTTCAAGGA` |
| UP00048\_1 | chr13 | + | 45849965 | 45849980 | 4.06e-05 | 0.389 | `TGTCTAAGGTCACAGC` |
| UP00048\_1 | chr1 | − | 12061819 | 12061834 | 4.09e-05 | 0.389 | `AGTTAAAGTTCACTTT` |
| UP00048\_1 | chr5 | + | 37774020 | 37774035 | 4.09e-05 | 0.389 | `TGTCAGAGTTCATCTG` |
| UP00048\_1 | chr2 | + | 54650674 | 54650689 | 4.11e-05 | 0.389 | `GTTTGAAGGTCACAAT` |
| UP00048\_1 | chr2 | − | 156082066 | 156082081 | 4.13e-05 | 0.389 | `ACGTAAAGTTCACTGA` |
| UP00048\_1 | chr11 | + | 63794248 | 63794263 | 4.15e-05 | 0.389 | `GAGCGAAGGTCACTGG` |
| UP00048\_1 | chr15 | − | 82440775 | 82440790 | 4.15e-05 | 0.389 | `TTTAGAAGGTCATGCA` |
| UP00048\_1 | chr11 | − | 34222278 | 34222293 | 4.18e-05 | 0.389 | `CTCCAGAGGTCAGCAT` |
| UP00048\_1 | chr11 | + | 90673044 | 90673059 | 4.18e-05 | 0.389 | `GAAAAAGGGTCACTTG` |
| UP00048\_1 | chr1 | − | 1701883 | 1701898 | 4.2e-05 | 0.389 | `TCTTCAAGGTCATATC` |
| UP00048\_1 | chr12 | + | 15832909 | 15832924 | 4.2e-05 | 0.389 | `CCTAAAGGGTCATTTT` |
| UP00048\_1 | chr14 | + | 34940782 | 34940797 | 4.2e-05 | 0.389 | `ACAAAAAGTTCACAAA` |
| UP00048\_1 | chr1 | − | 143245785 | 143245800 | 4.22e-05 | 0.389 | `CATGTAAGGTCATCTT` |
| UP00048\_1 | chr19 | − | 9799341 | 9799356 | 4.22e-05 | 0.389 | `AGTCAGAGGTCAGGCG` |
| UP00048\_1 | chrX | + | 29483615 | 29483630 | 4.27e-05 | 0.389 | `CTTCAAAGTTCATTGT` |
| UP00048\_1 | chr17 | − | 67930390 | 67930405 | 4.27e-05 | 0.389 | `GAGCAGAGTTCACCCA` |
| UP00048\_1 | chr10 | − | 11368647 | 11368662 | 4.29e-05 | 0.389 | `CGTCAAGGGTCACACA` |
| UP00048\_1 | chr13 | + | 96724897 | 96724912 | 4.29e-05 | 0.389 | `TATGTAAGGTCATTTG` |
| UP00048\_1 | chr1 | − | 195123745 | 195123760 | 4.34e-05 | 0.389 | `TATCAAAGTTCAGAGA` |
| UP00048\_1 | chr14 | − | 105307278 | 105307293 | 4.34e-05 | 0.389 | `CCCCTGAGGTCACGTG` |
| UP00048\_1 | chr22 | + | 40588175 | 40588190 | 4.34e-05 | 0.389 | `CCATAAAGTTCATGTG` |
| UP00048\_1 | chr3 | − | 49257220 | 49257235 | 4.39e-05 | 0.389 | `TCAGAAAGTTCATGTT` |
| UP00048\_1 | chr12 | + | 6431376 | 6431391 | 4.39e-05 | 0.389 | `GTGCAGAGGTCAGGTC` |
| UP00048\_1 | chr15 | − | 66048562 | 66048577 | 4.39e-05 | 0.389 | `CTCCCAAGGTCATGGG` |
| UP00048\_1 | chr12 | + | 67526802 | 67526817 | 4.44e-05 | 0.389 | `AAACAAAGTTCATAAG` |
| UP00048\_1 | chr20 | − | 31934924 | 31934939 | 4.44e-05 | 0.389 | `GAGCTGAGGTCACCAG` |
| UP00048\_1 | chr9 | − | 17853603 | 17853618 | 4.49e-05 | 0.389 | `TTCACAAGGTCAACCT` |
| UP00048\_1 | chr6 | + | 30189070 | 30189085 | 4.51e-05 | 0.389 | `ATGCAAAGTTCAAGCC` |
| UP00048\_1 | chr2 | − | 38715461 | 38715476 | 4.54e-05 | 0.389 | `TGAGAAAGGTCAAACT` |
| UP00048\_1 | chr3 | − | 25235063 | 25235078 | 4.54e-05 | 0.389 | `AAATAGAGGTCATCCA` |
| UP00048\_1 | chr1 | + | 201597822 | 201597837 | 4.56e-05 | 0.389 | `TCAAAGGGGTCACCTG` |
| UP00048\_1 | chr17 | + | 53282790 | 53282805 | 4.56e-05 | 0.389 | `GATTTGAGGTCACCAG` |
| UP00048\_1 | chr1 | + | 111560407 | 111560422 | 4.58e-05 | 0.389 | `TCCCCAAGGTCATTAT` |
| UP00048\_1 | chr10 | − | 71785484 | 71785499 | 4.58e-05 | 0.389 | `CCCCAAGGGTCACTGC` |
| UP00048\_1 | chr2 | + | 232244800 | 232244815 | 4.61e-05 | 0.389 | `GACCAGAGGTCAGGAC` |
| UP00048\_1 | chr6 | − | 133181350 | 133181365 | 4.61e-05 | 0.389 | `ATGCTAAGGTCAACCA` |
| UP00048\_1 | chr8 | + | 98712793 | 98712808 | 4.61e-05 | 0.389 | `TCAAAAAGTTCAAGAA` |
| UP00048\_1 | chr11 | + | 34601813 | 34601828 | 4.61e-05 | 0.389 | `GGCCAAAGTTCACTTC` |
| UP00048\_1 | chr14 | + | 75016809 | 75016824 | 4.61e-05 | 0.389 | `AGTCCAAGGTCACAGT` |
| UP00048\_1 | chr17 | + | 44657666 | 44657681 | 4.71e-05 | 0.396 | `TAACAAGGGTCAAAAG` |
| UP00048\_1 | chr3 | − | 99724068 | 99724083 | 4.74e-05 | 0.396 | `CCTTTAAGGTCATTTG` |
| UP00048\_1 | chr20 | + | 45549809 | 45549824 | 4.74e-05 | 0.396 | `TCTCACAGGTCAACCT` |
| UP00048\_1 | chr8 | + | 129303802 | 129303817 | 4.79e-05 | 0.398 | `TACAAAAGTTCAGCTC` |
| UP00048\_1 | chr8 | + | 110415825 | 110415840 | 4.84e-05 | 0.399 | `GCGCAAGGGTCATTTC` |
| UP00048\_1 | chr20 | + | 5983960 | 5983975 | 4.9e-05 | 0.399 | `TCTGAGAGTTCATCAG` |
| UP00048\_1 | chr1 | + | 243175476 | 243175491 | 4.92e-05 | 0.399 | `AGTTCAAGGTCACGAT` |
| UP00048\_1 | chr6 | − | 7827918 | 7827933 | 4.92e-05 | 0.399 | `ATCTTAAGGTCACAGT` |
| UP00048\_1 | chr6 | − | 131991378 | 131991393 | 4.92e-05 | 0.399 | `TCCAGAAGGTCAGCTG` |
| UP00048\_1 | chr10 | − | 73764822 | 73764837 | 4.92e-05 | 0.399 | `AACAGAAGGTCATGAG` |
| UP00048\_1 | chr5 | + | 141761040 | 141761055 | 4.95e-05 | 0.399 | `TCTCAGGGGTCATGGA` |
| UP00048\_1 | chr8 | + | 72913756 | 72913771 | 4.95e-05 | 0.399 | `TTTTAAGGGTCATTGT` |
| UP00048\_1 | chr20 | + | 45847121 | 45847136 | 5e-05 | 0.402 | `CCCTAAGGGTCAACAA` |
| UP00048\_1 | chr10 | + | 11324658 | 11324673 | 5.08e-05 | 0.404 | `AATCGAAGGTCAGCTT` |
| UP00048\_1 | chr6 | − | 39891463 | 39891478 | 5.11e-05 | 0.404 | `CCATAAAGTTCACCGC` |
| UP00048\_1 | chr19 | + | 3136594 | 3136609 | 5.11e-05 | 0.404 | `GTCGCAAGGTCACGTC` |
| UP00048\_1 | chr6 | − | 22107013 | 22107028 | 5.14e-05 | 0.404 | `GACAAAAGTTCAATGG` |
| UP00048\_1 | chr1 | + | 232575844 | 232575859 | 5.17e-05 | 0.404 | `TCGGAAGGGTCAAAAG` |
| UP00048\_1 | chr6 | + | 107887609 | 107887624 | 5.17e-05 | 0.404 | `CAGCAAAGGTCGTCAG` |
| UP00048\_1 | chr6 | + | 27884491 | 27884506 | 5.19e-05 | 0.404 | `CTCCAAAGGTTACGTA` |
| UP00048\_1 | chr9 | + | 113814376 | 113814391 | 5.19e-05 | 0.404 | `CTTAAGAGGTCAGGCT` |
| UP00048\_1 | chrX | + | 153282888 | 153282903 | 5.19e-05 | 0.404 | `CTGCTGAGGTCACCTG` |
| UP00048\_1 | chr16 | − | 79267831 | 79267846 | 5.22e-05 | 0.404 | `ACTGAGAGGTCAGGAA` |
| UP00048\_1 | chr2 | − | 105740125 | 105740140 | 5.33e-05 | 0.407 | `AAACAGAGGTCAGGAG` |
| UP00048\_1 | chr3 | + | 179719779 | 179719794 | 5.33e-05 | 0.407 | `TTACCAAGGTCAAGAG` |
| UP00048\_1 | chr2 | + | 9699597 | 9699612 | 5.36e-05 | 0.407 | `CATCCAAGGTCACTCC` |
| UP00048\_1 | chr3 | − | 134774475 | 134774490 | 5.36e-05 | 0.407 | `TTACCAAGGTCAAGTT` |
| UP00048\_1 | chr6 | + | 33352621 | 33352636 | 5.36e-05 | 0.407 | `TTTAAGGGGTCATCTA` |
| UP00048\_1 | chr1 | + | 232899682 | 232899697 | 5.39e-05 | 0.407 | `GCTTAGAGTTCAACTT` |
| UP00048\_1 | chr3 | + | 173307802 | 173307817 | 5.45e-05 | 0.407 | `AAGAAAAGTTCATAGA` |
| UP00048\_1 | chr8 | − | 42158986 | 42159001 | 5.51e-05 | 0.407 | `GCTGCAAGGTCAAGAT` |
| UP00048\_1 | chr17 | − | 33203625 | 33203640 | 5.51e-05 | 0.407 | `TTGCCAAGGTCATTTA` |
| UP00048\_1 | chr17 | − | 52790259 | 52790274 | 5.51e-05 | 0.407 | `TCACTAAGGTCAAAGG` |
| UP00048\_1 | chr9 | − | 114585954 | 114585969 | 5.54e-05 | 0.407 | `TCTCACAGGTCACTCA` |
| UP00048\_1 | chr13 | − | 47614744 | 47614759 | 5.54e-05 | 0.407 | `TATCTGAGGTCACTTA` |
| UP00048\_1 | chr18 | − | 17966501 | 17966516 | 5.54e-05 | 0.407 | `GAGGAGAGGTCATAGA` |
| UP00048\_1 | chr3 | − | 113554021 | 113554036 | 5.57e-05 | 0.407 | `TTTTTGAGGTCACCAA` |
| UP00048\_1 | chr7 | + | 23024668 | 23024683 | 5.57e-05 | 0.407 | `CCTGAGAGGTCATTCA` |
| UP00048\_1 | chr16 | − | 11671906 | 11671921 | 5.57e-05 | 0.407 | `GACATGAGGTCACCAG` |
| UP00048\_1 | chr3 | + | 181479346 | 181479361 | 5.6e-05 | 0.407 | `ATACAAAGTTCACTGA` |
| UP00048\_1 | chr17 | − | 73634992 | 73635007 | 5.6e-05 | 0.407 | `TCTTTGAGGTCACAGG` |
| UP00048\_1 | chr16 | + | 66526935 | 66526950 | 5.63e-05 | 0.408 | `AGGCCAAGGTCACAGG` |
| UP00048\_1 | chr5 | − | 106934969 | 106934984 | 5.66e-05 | 0.408 | `AGGAAAAGTTCACAGT` |
| UP00048\_1 | chr10 | − | 63326677 | 63326692 | 5.72e-05 | 0.411 | `TGGGAAGGGTCATGTG` |
| UP00048\_1 | chr7 | − | 73270016 | 73270031 | 5.78e-05 | 0.413 | `AATCAAAGGACACCTG` |
| UP00048\_1 | chr17 | + | 39936545 | 39936560 | 5.78e-05 | 0.413 | `AGGGAAAGGTCAGAGG` |
| UP00048\_1 | chr6 | + | 27968498 | 27968513 | 5.84e-05 | 0.414 | `TTTTAAAGTTCAGCCC` |
| UP00048\_1 | chr9 | − | 115384178 | 115384193 | 5.84e-05 | 0.414 | `TGTCAGGGGTCACGCT` |
| UP00048\_1 | chr10 | + | 11327531 | 11327546 | 5.9e-05 | 0.418 | `AAACAAAGTTCAGGTG` |
| UP00048\_1 | chr7 | − | 26195975 | 26195990 | 5.93e-05 | 0.418 | `TTTTAAAGGTTAACCG` |
| UP00048\_1 | chr17 | − | 3561285 | 3561300 | 5.96e-05 | 0.419 | `CTCCAAAGTTCAGATG` |
| UP00048\_1 | chr2 | + | 38015378 | 38015393 | 6.06e-05 | 0.424 | `CTCTTAAGGTCACTAG` |
| UP00048\_1 | chr2 | + | 38715513 | 38715528 | 6.09e-05 | 0.424 | `CTGTCAAGGTCACGCA` |
| UP00048\_1 | chr19 | + | 17367129 | 17367144 | 6.09e-05 | 0.424 | `CCTTTAAGGTCATTTT` |
| UP00048\_1 | chr3 | + | 10237487 | 10237502 | 6.12e-05 | 0.425 | `TGAGAGAGGTCACCAC` |
| UP00048\_1 | chr19 | + | 63611494 | 63611509 | 6.22e-05 | 0.427 | `ACTGTGAGGTCACCGG` |
| UP00048\_1 | chrX | − | 7043383 | 7043398 | 6.25e-05 | 0.427 | `AAAAGAAGGTCACCAA` |
| UP00048\_1 | chr3 | + | 123304339 | 123304354 | 6.28e-05 | 0.427 | `TTAGAGAGGTCACTGA` |
| UP00048\_1 | chr16 | + | 88159131 | 88159146 | 6.28e-05 | 0.427 | `AGCAAGAGGTCATTTT` |
| UP00048\_1 | chr2 | − | 191243778 | 191243793 | 6.35e-05 | 0.427 | `CCTCAGAGTTCAACAA` |
| UP00048\_1 | chr9 | + | 84959092 | 84959107 | 6.42e-05 | 0.427 | `TTTGAAAGTTCAAACC` |
| UP00048\_1 | chr17 | + | 16130242 | 16130257 | 6.42e-05 | 0.427 | `TATTAAAGTTCAGAGT` |
| UP00048\_1 | chr18 | + | 58999404 | 58999419 | 6.42e-05 | 0.427 | `TTTTAGAGTTCATCTC` |
| UP00048\_1 | chr1 | − | 87346630 | 87346645 | 6.45e-05 | 0.427 | `CATAAAAGGTTACAGG` |
| UP00048\_1 | chr5 | − | 33886200 | 33886215 | 6.45e-05 | 0.427 | `TCCCAGAGGTCGCCGT` |
| UP00048\_1 | chr10 | + | 64085390 | 64085405 | 6.45e-05 | 0.427 | `AATTCAAGGTCATTTT` |
| UP00048\_1 | chr14 | − | 105536918 | 105536933 | 6.45e-05 | 0.427 | `CACCAGGGGTCACCCA` |
| UP00048\_1 | chr15 | − | 19962665 | 19962680 | 6.45e-05 | 0.427 | `CACCAGGGGTCACCCA` |
| UP00048\_1 | chr3 | − | 23670147 | 23670162 | 6.48e-05 | 0.427 | `GGGAAAAGGTCAGTGA` |
| UP00048\_1 | chr14 | − | 67747673 | 67747688 | 6.48e-05 | 0.427 | `GGGAAAGGGTCACAAA` |
| UP00048\_1 | chr11 | − | 63750710 | 63750725 | 6.52e-05 | 0.427 | `GCAGCAAGGTCACCTC` |
| UP00048\_1 | chr1 | − | 31668861 | 31668876 | 6.55e-05 | 0.427 | `TAGCCGAGGTCACGCT` |
| UP00048\_1 | chr3 | + | 4994408 | 4994423 | 6.55e-05 | 0.427 | `GTGAGAAGGTCACAGA` |
| UP00048\_1 | chr6 | − | 133182391 | 133182406 | 6.55e-05 | 0.427 | `TGGTAAAGTTCACTTC` |
| UP00048\_1 | chr20 | + | 48666785 | 48666800 | 6.55e-05 | 0.427 | `CCCTCAAGGTCAAGGG` |
| UP00048\_1 | chr9 | − | 33294548 | 33294563 | 6.62e-05 | 0.428 | `TCAAAGAGTTCACCAA` |
| UP00048\_1 | chr2 | + | 201696891 | 201696906 | 6.66e-05 | 0.428 | `CCTGAAAGTTCAGGTT` |
| UP00048\_1 | chr7 | + | 4689665 | 4689680 | 6.66e-05 | 0.428 | `TTACTGAGGTCACCTT` |
| UP00048\_1 | chr1 | + | 31668983 | 31668998 | 6.69e-05 | 0.428 | `CCTGAAAGTTCAATGG` |
| UP00048\_1 | chr3 | + | 99738195 | 99738210 | 6.72e-05 | 0.428 | `TACTAGGGGTCACAAG` |
| UP00048\_1 | chr2 | − | 125846206 | 125846221 | 6.76e-05 | 0.428 | `TTAACAAGGTCACTTT` |
| UP00048\_1 | chr22 | − | 27525022 | 27525037 | 6.76e-05 | 0.428 | `TACTTAAGGTCAAACT` |
| UP00048\_1 | chr5 | − | 5414277 | 5414292 | 6.79e-05 | 0.428 | `GTTCAGAGTTCACTGA` |
| UP00048\_1 | chr7 | − | 48116549 | 48116564 | 6.79e-05 | 0.428 | `GTATAGAGTTCACCTG` |
| UP00048\_1 | chr19 | − | 17837655 | 17837670 | 6.79e-05 | 0.428 | `AAGGTAAGGTCACATC` |
| UP00048\_1 | chr8 | + | 33531898 | 33531913 | 6.83e-05 | 0.428 | `GGGCAGAGGTCAGCTC` |
| UP00048\_1 | chr8 | − | 125277451 | 125277466 | 6.83e-05 | 0.428 | `TTATAGAGGTCAATCG` |
| UP00048\_1 | chr10 | − | 112145638 | 112145653 | 6.83e-05 | 0.428 | `ACACAGAGGTCAGGCA` |
| UP00048\_1 | chr13 | + | 46123383 | 46123398 | 6.9e-05 | 0.43 | `CATCAAAGTTCAGAAA` |
| UP00048\_1 | chr6 | + | 7834534 | 7834549 | 6.94e-05 | 0.43 | `TTTCAAGGTTCATCTC` |
| UP00048\_1 | chr22 | − | 46762723 | 46762738 | 6.94e-05 | 0.43 | `ACACAGAGGTCAGGGA` |
| UP00048\_1 | chr3 | + | 187984152 | 187984167 | 6.97e-05 | 0.43 | `TAGTGAAGGTCATAGG` |
| UP00048\_1 | chr5 | + | 106759273 | 106759288 | 6.97e-05 | 0.43 | `GCTTTAAGGTCAGCCA` |
| UP00048\_1 | chr6 | + | 27208650 | 27208665 | 7.01e-05 | 0.43 | `CGTCAGAGGTCAGGTC` |
| UP00048\_1 | chr1 | − | 148801487 | 148801502 | 7.04e-05 | 0.43 | `CCTGAGAGGTCAGAGG` |
| UP00048\_1 | chr17 | + | 63746880 | 63746895 | 7.04e-05 | 0.43 | `GAAAACAGGTCACCTG` |
| UP00048\_1 | chr20 | − | 45820991 | 45821006 | 7.04e-05 | 0.43 | `CTTTCAAGGTCACAGC` |
| UP00048\_1 | chr3 | + | 4994274 | 4994289 | 7.08e-05 | 0.43 | `GCGCAGAGGTCAGTGA` |
| UP00048\_1 | chr1 | + | 201557048 | 201557063 | 7.12e-05 | 0.43 | `TGGAAGAGGTCATTTC` |
| UP00048\_1 | chr4 | − | 106734774 | 106734789 | 7.12e-05 | 0.43 | `AATCAAAGGTTAAAAG` |
| UP00048\_1 | chr6 | − | 30188889 | 30188904 | 7.12e-05 | 0.43 | `GTCCAAAGTTCAGGAC` |
| UP00048\_1 | chr11 | − | 7520594 | 7520609 | 7.3e-05 | 0.438 | `GACACAAGGTCATGGC` |
| UP00048\_1 | chr13 | + | 21587542 | 21587557 | 7.3e-05 | 0.438 | `GTGAAAAGTTCAGCTC` |
| UP00048\_1 | chr9 | − | 78277217 | 78277232 | 7.53e-05 | 0.449 | `GCATAAGGGTCAAGAT` |
| UP00048\_1 | chr13 | − | 109922262 | 109922277 | 7.53e-05 | 0.449 | `CTTCTGAGGTCACCAC` |
| UP00048\_1 | chr3 | − | 23874324 | 23874339 | 7.61e-05 | 0.449 | `TAGCTAAGGTCATTGC` |
| UP00048\_1 | chr10 | + | 2980961 | 2980976 | 7.61e-05 | 0.449 | `TGCCAAAGTTCAATAA` |
| UP00048\_1 | chr11 | − | 117786185 | 117786200 | 7.61e-05 | 0.449 | `GAAAAGAGGTCATTTC` |
| UP00048\_1 | chr12 | + | 112130531 | 112130546 | 7.61e-05 | 0.449 | `ATCAAGAGTTCATCTT` |
| UP00048\_1 | chr1 | + | 179634719 | 179634734 | 7.65e-05 | 0.45 | `TGTAGAAGGTCACTTC` |
| UP00048\_1 | chr5 | − | 5414246 | 5414261 | 7.68e-05 | 0.45 | `ACCAAGAGTTCACAGT` |
| UP00048\_1 | chr17 | + | 38801022 | 38801037 | 7.68e-05 | 0.45 | `ATCCTGAGGTCACGGT` |
| UP00048\_1 | chr4 | − | 14467269 | 14467284 | 7.72e-05 | 0.45 | `CCTTGAAGGTCAAGGA` |
| UP00048\_1 | chr16 | + | 22217226 | 22217241 | 7.72e-05 | 0.45 | `TCCCAGAGGTTACGTT` |
| UP00048\_1 | chr14 | − | 105238295 | 105238310 | 7.8e-05 | 0.452 | `AGGCAGAGGTCAGCCA` |
| UP00048\_1 | chr21 | + | 15516386 | 15516401 | 7.8e-05 | 0.452 | `TCAGGAAGGTCACAGG` |
| UP00048\_1 | chr4 | + | 152885563 | 152885578 | 7.84e-05 | 0.452 | `TGACAAGGGTCACTAT` |
| UP00048\_1 | chr18 | − | 42008023 | 42008038 | 7.84e-05 | 0.452 | `AAGAAAATGTCACCAG` |
| UP00048\_1 | chr21 | − | 41739733 | 41739748 | 7.88e-05 | 0.452 | `GTGTCAAGGTCATTTG` |
| UP00048\_1 | chr11 | + | 65112265 | 65112280 | 7.96e-05 | 0.452 | `TCGTGGAGGTCACGTT` |
| UP00048\_1 | chr12 | − | 100962059 | 100962074 | 7.96e-05 | 0.452 | `CTTTAAATGTCACCAG` |
| UP00048\_1 | chr2 | − | 12114944 | 12114959 | 8.01e-05 | 0.452 | `ATTTCGAGGTCACATG` |
| UP00048\_1 | chr3 | + | 53780089 | 53780104 | 8.01e-05 | 0.452 | `CTGGAAGGGTCACAGA` |
| UP00048\_1 | chr1 | + | 87344779 | 87344794 | 8.13e-05 | 0.452 | `CCTTAAAGGTTATGGG` |
| UP00048\_1 | chr6 | + | 135685889 | 135685904 | 8.13e-05 | 0.452 | `ATGCAAAGGTTACAAA` |
| UP00048\_1 | chr6 | + | 135685889 | 135685904 | 8.13e-05 | 0.452 | `ATGCAAAGGTTACAAA` |
| UP00048\_1 | chr16 | − | 45510560 | 45510575 | 8.13e-05 | 0.452 | `CCTCTGAGGTCACACA` |
| UP00048\_1 | chr18 | − | 9056847 | 9056862 | 8.13e-05 | 0.452 | `CATTTAAGGTCAAGCC` |
| UP00048\_1 | chr7 | − | 7974292 | 7974307 | 8.17e-05 | 0.452 | `TAGCAGGGGTCAACAA` |
| UP00048\_1 | chr13 | − | 45862096 | 45862111 | 8.21e-05 | 0.452 | `TCTGGAAGTTCACCTT` |
| UP00048\_1 | chr2 | − | 98443950 | 98443965 | 8.25e-05 | 0.452 | `CCCATGAGGTCACGGG` |
| UP00048\_1 | chr2 | − | 231444146 | 231444161 | 8.25e-05 | 0.452 | `ATTTAGGGGTCAAGTG` |
| UP00048\_1 | chr20 | − | 47338217 | 47338232 | 8.25e-05 | 0.452 | `ACGTAAGGGTCATTGT` |
| UP00048\_1 | chr1 | − | 201597864 | 201597879 | 8.29e-05 | 0.452 | `ATTCTGAGGTCACAGA` |
| UP00048\_1 | chr8 | + | 141546561 | 141546576 | 8.29e-05 | 0.452 | `AAATAAAGTTCAAGAA` |
| UP00048\_1 | chr12 | + | 92093641 | 92093656 | 8.29e-05 | 0.452 | `TAAAAAAGTTCAGCAT` |
| UP00048\_1 | chr4 | − | 88068753 | 88068768 | 8.34e-05 | 0.452 | `TGGCACAGGTCATCTG` |
| UP00048\_1 | chr6 | − | 344865 | 344880 | 8.34e-05 | 0.452 | `TCCCAAAGGGCATCCT` |
| UP00048\_1 | chr10 | + | 63326736 | 63326751 | 8.34e-05 | 0.452 | `GCTCCAAGTTCATGAG` |
| UP00048\_1 | chr14 | + | 96424448 | 96424463 | 8.38e-05 | 0.452 | `TTGTAGAGGTCAGAGA` |
| UP00048\_1 | chr12 | + | 109583764 | 109583779 | 8.42e-05 | 0.452 | `ACCATGAGGTCACGGT` |
| UP00048\_1 | chr21 | − | 25866234 | 25866249 | 8.42e-05 | 0.452 | `ACTAGAAGGTCATTCA` |
| UP00048\_1 | chr21 | + | 15516454 | 15516469 | 8.46e-05 | 0.452 | `TACCCAAGTTCACAAG` |
| UP00048\_1 | chr2 | + | 111642873 | 111642888 | 8.55e-05 | 0.452 | `TCAAAGGGGTCACCCA` |
| UP00048\_1 | chr18 | − | 19735188 | 19735203 | 8.55e-05 | 0.452 | `GCACGAAGGTCACTGA` |
| UP00048\_1 | chr8 | + | 28252531 | 28252546 | 8.59e-05 | 0.452 | `GCACCAAGGTCATGGC` |
| UP00048\_1 | chr21 | + | 44451305 | 44451320 | 8.63e-05 | 0.452 | `GTGCAGGGGTCACTTT` |
| UP00048\_1 | chr7 | + | 999619 | 999634 | 8.68e-05 | 0.452 | `GCTGGAAGGTCAGCTT` |
| UP00048\_1 | chr7 | − | 114345143 | 114345158 | 8.68e-05 | 0.452 | `ATTAAAGGTTCACATT` |
| UP00048\_1 | chr9 | − | 70787772 | 70787787 | 8.68e-05 | 0.452 | `TAGTAAAGGTTATATG` |
| UP00048\_1 | chr12 | − | 91281268 | 91281283 | 8.68e-05 | 0.452 | `GATTCAAGGTCAGCTC` |
| UP00048\_1 | chr15 | − | 42795852 | 42795867 | 8.68e-05 | 0.452 | `TTCTCAAGGTCAAAAA` |
| UP00048\_1 | chr19 | + | 12764559 | 12764574 | 8.68e-05 | 0.452 | `AACAGAAGGTCATGAC` |
| UP00048\_1 | chr19 | + | 54070693 | 54070708 | 8.68e-05 | 0.452 | `CCGAGAAGGTCACTGT` |
| UP00048\_1 | chr1 | − | 158860126 | 158860141 | 8.72e-05 | 0.452 | `TCCAAAAAGTCACCAG` |
| UP00048\_1 | chr1 | + | 41973760 | 41973775 | 8.76e-05 | 0.452 | `TCACAGAGGTCAGTGT` |
| UP00048\_1 | chr2 | − | 149133254 | 149133269 | 8.76e-05 | 0.452 | `CCAGAAAGTTCACAGA` |
| UP00048\_1 | chr19 | + | 63679233 | 63679248 | 8.76e-05 | 0.452 | `GTTCAAAGGGCATGGG` |
| UP00048\_1 | chr2 | − | 54650656 | 54650671 | 8.81e-05 | 0.452 | `CATCAAAAGTCACCAG` |
| UP00048\_1 | chr20 | + | 23262908 | 23262923 | 8.81e-05 | 0.452 | `ATTCTGAGGTCAAGGG` |
| UP00048\_1 | chr2 | + | 25498042 | 25498057 | 8.85e-05 | 0.452 | `TATCCGAGGTCAAAAG` |
| UP00048\_1 | chr2 | − | 201690457 | 201690472 | 8.9e-05 | 0.452 | `TTTCAAATGTCAACCA` |
| UP00048\_1 | chr4 | − | 121207795 | 121207810 | 8.9e-05 | 0.452 | `CACTAGGGGTCACATG` |
| UP00048\_1 | chr22 | − | 27521828 | 27521843 | 8.9e-05 | 0.452 | `GTCCCGAGGTCATCTT` |
| UP00048\_1 | chr8 | − | 67057113 | 67057128 | 8.94e-05 | 0.452 | `CTGAAAGGGTCATTTA` |
| UP00048\_1 | chr10 | − | 5944466 | 5944481 | 8.94e-05 | 0.452 | `CCCCCAAGGTCAGCTC` |
| UP00048\_1 | chr5 | + | 55480471 | 55480486 | 8.99e-05 | 0.452 | `GCTGAGAGGTCAGTAA` |
| UP00048\_1 | chr5 | + | 124068076 | 124068091 | 8.99e-05 | 0.452 | `CCTTAGAGGTCGCCAG` |
| UP00048\_1 | chr13 | + | 48004292 | 48004307 | 8.99e-05 | 0.452 | `GCAAAAAGTTCATTCT` |
| UP00048\_1 | chr14 | − | 94798505 | 94798520 | 9.03e-05 | 0.452 | `TCATAAATGTCACGTG` |
| UP00048\_1 | chr7 | − | 47944710 | 47944725 | 9.08e-05 | 0.452 | `AGGCGAAGGTCACACA` |
| UP00048\_1 | chr11 | − | 85336743 | 85336758 | 9.08e-05 | 0.452 | `GGCAAGAGGTCAGCGA` |
| UP00048\_1 | chr19 | + | 4218769 | 4218784 | 9.08e-05 | 0.452 | `CAGGTAAGGTCACAGA` |
| UP00048\_1 | chr9 | + | 24922972 | 24922987 | 9.12e-05 | 0.452 | `TTCTTGAGGTCACATT` |
| UP00048\_1 | chr17 | + | 78000946 | 78000961 | 9.12e-05 | 0.452 | `GCTGAAAGTTCAGACT` |
| UP00048\_1 | chr20 | − | 61730050 | 61730065 | 9.12e-05 | 0.452 | `CTGGAGAGGTCAGGAG` |
| UP00048\_1 | chr21 | − | 25880832 | 25880847 | 9.17e-05 | 0.452 | `ATGTACAGGTCACATG` |
| UP00048\_1 | chr22 | + | 41341278 | 41341293 | 9.21e-05 | 0.452 | `GGGTAAAGGTCGCCCT` |
| UP00048\_1 | chr11 | − | 86826012 | 86826027 | 9.26e-05 | 0.452 | `AGGCTAAGGTCAAGAA` |
| UP00048\_1 | chr13 | − | 76218239 | 76218254 | 9.26e-05 | 0.452 | `CAAAAGAGGTCAATGG` |
| UP00048\_1 | chr15 | + | 29408724 | 29408739 | 9.26e-05 | 0.452 | `GCTCAGAGTTCAGCTC` |
| UP00048\_1 | chr12 | − | 26844074 | 26844089 | 9.31e-05 | 0.452 | `TAGTTAAGGTCAGCTC` |
| UP00048\_1 | chr12 | + | 91906004 | 91906019 | 9.31e-05 | 0.452 | `TTTCAGAGTTCAGCAA` |
| UP00048\_1 | chr3 | + | 99758020 | 99758035 | 9.35e-05 | 0.452 | `ACGCAAAGTTCAGAAC` |
| UP00048\_1 | chr10 | + | 73603912 | 73603927 | 9.35e-05 | 0.452 | `AGTTCAAGGTCATGTC` |
| UP00048\_1 | chr15 | − | 61553755 | 61553770 | 9.35e-05 | 0.452 | `TTGGGAAGGTCAACAA` |
| UP00048\_1 | chr17 | + | 38801073 | 38801088 | 9.35e-05 | 0.452 | `TCCCTAAGTTCACACA` |
| UP00048\_1 | chr2 | + | 9755734 | 9755749 | 9.4e-05 | 0.452 | `GCACAGAGTTCACGCC` |
| UP00048\_1 | chr2 | − | 74051831 | 74051846 | 9.4e-05 | 0.452 | `CATCTGAGGTCAAGTT` |
| UP00048\_1 | chr17 | + | 4246022 | 4246037 | 9.4e-05 | 0.452 | `CCCTAAAGGGCACCAG` |
| UP00048\_1 | chr6 | − | 91240135 | 91240150 | 9.44e-05 | 0.452 | `ACTAAAGGTTCATGTA` |
| UP00048\_1 | chr17 | + | 70803312 | 70803327 | 9.44e-05 | 0.452 | `CCCTCAAGGTCAGGTG` |
| UP00048\_1 | chr6 | + | 7828111 | 7828126 | 9.49e-05 | 0.452 | `AAGAGAAGGTCAAAGG` |
| UP00048\_1 | chr1 | − | 177209408 | 177209423 | 9.54e-05 | 0.452 | `TCTTAGAGTTCAAAGT` |
| UP00048\_1 | chr14 | + | 63404664 | 63404679 | 9.54e-05 | 0.452 | `TTTGAAAGGTGACCTA` |
| UP00048\_1 | chr21 | − | 34271198 | 34271213 | 9.54e-05 | 0.452 | `AATTTAAGTTCATCTG` |
| UP00048\_1 | chr5 | + | 139205149 | 139205164 | 9.59e-05 | 0.452 | `CCCAAGAGTTCATATG` |
| UP00048\_1 | chr15 | + | 49308117 | 49308132 | 9.59e-05 | 0.452 | `TCTCCAGGGTCACAGA` |
| UP00048\_1 | chr16 | − | 11678916 | 11678931 | 9.59e-05 | 0.452 | `ACTTCAAGTTCACCTC` |
| UP00048\_1 | chr17 | + | 33203200 | 33203215 | 9.59e-05 | 0.452 | `TCTCAAAGGACAACAG` |
| UP00048\_1 | chr8 | − | 144179086 | 144179101 | 9.63e-05 | 0.453 | `TGCTCAAGGTCATGTC` |
| UP00048\_1 | chr20 | + | 22170581 | 22170596 | 9.68e-05 | 0.454 | `AAGAAAGGGTCAATTC` |
| UP00048\_1 | chr1 | − | 172102346 | 172102361 | 9.73e-05 | 0.454 | `CACCTAAGTTCACATG` |
| UP00048\_1 | chr8 | − | 125003636 | 125003651 | 9.73e-05 | 0.454 | `CAGCTGAGGTCATGTG` |
| UP00048\_1 | chr11 | − | 7490426 | 7490441 | 9.78e-05 | 0.454 | `CACACAAGGTCATGGC` |
| UP00048\_1 | chr14 | − | 61198983 | 61198998 | 9.88e-05 | 0.454 | `AGGAGAAGGTCACCGC` |
| UP00048\_1 | chr17 | − | 71779161 | 71779176 | 9.88e-05 | 0.454 | `AAGGGAAGGTCACAGA` |
| UP00048\_1 | chr1 | − | 16713159 | 16713174 | 9.93e-05 | 0.454 | `CGCCAAGGGTCATATC` |
| UP00048\_1 | chr2 | + | 157821386 | 157821401 | 9.93e-05 | 0.454 | `GCCCAAATGTCATCTC` |
| UP00048\_1 | chr6 | − | 100123379 | 100123394 | 9.93e-05 | 0.454 | `CTCCAAGGGTCAGCAC` |
| UP00048\_1 | chr7 | + | 4689757 | 4689772 | 9.93e-05 | 0.454 | `ACTCCAGGGTCACCCC` |
| UP00048\_1 | chr13 | + | 33201118 | 33201133 | 9.93e-05 | 0.454 | `GCTGAGAGTTCATGTC` |
| UP00048\_1 | chr15 | − | 29433077 | 29433092 | 9.93e-05 | 0.454 | `AATCTGAGGTCAGCTG` |
| UP00048\_1 | chr11 | − | 86825984 | 86825999 | 9.98e-05 | 0.454 | `TGACAGAGTTCACATG` |
| UP00048\_1 | chr12 | + | 26343045 | 26343060 | 9.98e-05 | 0.454 | `TCTGTGAGGTCACTGG` |
| UP00048\_1 | chr18 | − | 58978957 | 58978972 | 9.98e-05 | 0.454 | `TTTGACAGGTCACTAG` |

---

**DEBUGGING INFORMATION**


---

Command line:

```
/ebi/sw/MEME/VM-cluster410/meme-versions/4.10.0/bin/fimo --parse-genomic-coord --verbosity 1 --oc fimo_out_7 --bgfile ./background --motif UP00048_1 db/uniprobe_mouse.meme ./Supplementary_Table_1.500bp.fa
```

Settings:

```
|  |  |  |
| --- | --- | --- |
| output directory = fimo_out_7 | MEME file name = db/uniprobe_mouse.meme | sequence file name = ./Supplementary_Table_1.500bp.fa |
| background file name = ./background | allow clobber = true | compute q-values = true |
| parse genomic coord. = true | text only = false | scan both strands = true |
| max sequence length = 250000000 | output threshold = 0.0001 | threshold type = p-value |
| max stored scores = 100000 | pseudocount = 0.1 | verbosity = 1 |
| selected motif = UP00048_1 |  |  |
```

This information can be useful in the event you wish to report a
problem with the FIMO software.

---

**Go to top**
