# Supplementary material for: The MEME Suite
Source: Nucleic Acids Res. 2015 May 7;43(Web Server issue):W39–49. doi: 10.1093/nar/gkv416 (PMC4489269; doi:10.1093/nar/gkv416)
Supplement: SUPPLEMENTARY DATA [file supp_gkv416_nar-00283-web-b-2015-File005.zip › case4/meme-chip/fimo_out_8/fimo.html]

FIMO Results


---

|  |  |  |
| --- | --- | --- |
| **Database and Motifs** | **High-scoring Motif Occurrences** | **Debugging Information** |

  
  

---

**FIMO - Motif search tool**


---

FIMO version 4.10.0,
(Release date: Wed May 21 10:35:36 2014 +1000)

For further information on how to interpret these results
or to get a copy of the FIMO software please access
http://meme.nbcr.net

If you use FIMO in your research, please cite the following paper:  
Charles E. Grant, Timothy L. Bailey, and William Stafford Noble,
"FIMO: Scanning for occurrences of a given motif",
*Bioinformatics*, **27**(7):1017-1018, 2011.
[full text]

---

**DATABASE AND MOTIFS**


---

DATABASE
./Supplementary\_Table\_1.500bp.fa  
Database contains
2776
sequences,
1388000
residues

MOTIFS
db/uniprobe\_mouse.meme
(nucleotide)

| MOTIF | WIDTH | BEST POSSIBLE MATCH |
| --- | --- | --- |
| UP00078\_1 | 17 | GGGTTTAATTAAAATTC |
| UP00059\_1 | 14 | CTAATATTGCTAAA |
| UP00099\_1 | 17 | CTCAGCAGCTGCTCCTG |
| UP00020\_1 | 16 | ACGATGACGTCATCGA |
| UP00012\_1 | 15 | TAATTCAATGAAGTG |
| UP00043\_1 | 16 | TCTTTCGAGGAATTTG |
| UP00050\_1 | 22 | GGAAGAGTCACGTGACCAATAC |
| UP00001\_1 | 15 | ATAAAGGCGCGCGAT |
| UP00003\_1 | 15 | ATAAGGGCGCGCGAT |
| UP00007\_1 | 14 | TCCGCCCCCGCATT |
| UP00015\_1 | 15 | AGGACCCGGAAGTAA |
| UP00407\_1 | 13 | TACAAGGAAGTAA |
| UP00068\_1 | 17 | TAAAAGGTGTGAAAATT |
| UP00079\_1 | 17 | TATTCAAGGTCATGCGA |
| UP00073\_1 | 17 | AAAAAGTAAACAAAGAC |
| UP00041\_1 | 16 | AAAGTAAACAAAAATT |
| UP00039\_1 | 17 | AAAAAGTAAACAAACCC |
| UP00025\_1 | 17 | AAAATGTAAACAAACAG |
| UP00061\_1 | 17 | TAAATGTAAACAAAGGT |
| UP00408\_1 | 17 | CAATACCGGAAGTGTAA |
| UP00032\_1 | 22 | TTTTTAGAGATAAGAAATAAAG |
| UP00080\_1 | 17 | TAAACTGATAAGAAGAT |
| UP00100\_1 | 17 | TATAGAGATAAGAATTG |
| UP00070\_1 | 16 | TCGTACCCGCATCATT |
| UP00024\_1 | 16 | TATCGACCCCCCACAG |
| UP00042\_1 | 17 | CAGATGTGCACATACGT |
| UP00084\_1 | 17 | GAGTGTACGTACGATGG |
| UP00055\_1 | 16 | ACTATGAATGAATGAT |
| UP00035\_1 | 16 | ACTATGCCAACCTACC |
| UP00066\_1 | 17 | CTTCAGGGGTCAATTGA |
| UP00391\_1 | 14 | TGGAGGTAATTAAC |
| UP00072\_1 | 16 | ATTTACGACAAATAGC |
| UP00086\_1 | 14 | GAGAACCGAAACTG |
| UP00018\_1 | 15 | CGTATCGAAACCAAA |
| UP00040\_1 | 15 | ATAAACCGAAACCAA |
| UP00011\_1 | 17 | CTGATCGAAACCAAAGT |
| UP00074\_1 | 15 | CAAAATCGAAACTAA |
| UP00103\_1 | 16 | CCGATGACGTCATCGT |
| UP00093\_1 | 16 | TCGACCCCGCCCCTAT |
| UP00067\_1 | 17 | AATCCCTTTGATCTATC |
| UP00045\_1 | 17 | AAATTTGCTGACTTAGC |
| UP00044\_1 | 15 | TAAAAATGCTGACTT |
| UP00060\_1 | 16 | TGACCACGTGGTCGGG |
| UP00097\_1 | 16 | GGGCCGTGTGCAAAAA |
| UP00092\_1 | 17 | ATGGAAACCGTTATTTT |
| UP00081\_1 | 17 | TTGAAAACCGTTAATTT |
| UP00036\_1 | 16 | GAAGAACAGGTGTCCG |
| UP00017\_1 | 17 | CTTAACCACTTAAGGAT |
| UP00009\_1 | 16 | TCTCAAAGGTCACGAG |
| UP00027\_1 | 16 | TTTTACAGTAGCAAAA |
| UP00052\_1 | 16 | ATGTACAGTAGCAAAG |
| UP00088\_1 | 16 | TTGGGGGCGCCCCTAG |
| UP00048\_1 | 16 | TCTCAAAGGTCACCTG |
| UP00098\_1 | 23 | TGTGACCCTTAGCAACCGATTAA |
| UP00056\_1 | 15 | TACCATAGCAACGGT |
| UP00076\_1 | 15 | CCGCATAGCAACGGA |
| UP00053\_1 | 17 | TGTCGTGACCCCTTAAT |
| UP00085\_1 | 14 | TTAAGAGGAAGTTA |
| UP00008\_1 | 17 | AATAGGGTATCATATAT |
| UP00000\_1 | 17 | CAAATCCAGACATCAGA |
| UP00030\_1 | 17 | ATAAGAACAAAGGACTA |
| UP00101\_1 | 14 | TAATTGTTCTAAAC |
| UP00096\_1 | 16 | TTAAGAACAATAATTT |
| UP00004\_1 | 16 | GCTAATTATAATTATC |
| UP00075\_1 | 17 | TAGTGAACAATAGATTT |
| UP00014\_1 | 15 | ATAAACAATTAATCA |
| UP00064\_1 | 16 | TTCAATTGTTCTAAAA |
| UP00069\_1 | 16 | AATCAATTCAATAATT |
| UP00071\_1 | 16 | TTTAATTATAATTAAG |
| UP00023\_1 | 16 | ATTGAACAATGGAATT |
| UP00062\_1 | 17 | AGAAGAACAAAGGACTA |
| UP00091\_1 | 16 | TTTAGAACAATAAAAT |
| UP00034\_1 | 22 | AATAAAGAACAATAGAATTTCA |
| UP00051\_1 | 17 | TTATCTATTGTTCTTTA |
| UP00049\_1 | 14 | ATTTTACGGAAAAT |
| UP00002\_1 | 17 | GGTCCCGCCCCCTTCTC |
| UP00406\_1 | 16 | GTACATCCGGATTTTT |
| UP00077\_1 | 14 | TTCCATATATGGAA |
| UP00016\_1 | 16 | TATAATTATAATATTC |
| UP00029\_1 | 16 | TCTTTATATATAAATA |
| UP00089\_1 | 17 | ACTTAGTTAACTAAAAA |
| UP00058\_1 | 17 | TATAGATCAAAGGAAAA |
| UP00054\_1 | 17 | TATAGATCAAAGGAAAA |
| UP00083\_1 | 17 | ATTTCCTTTGATCTATA |
| UP00005\_1 | 15 | ATTCCCTGAGGGGAA |
| UP00010\_1 | 14 | TTGCCCTAGGGCAT |
| UP00087\_1 | 15 | ATTGCCTGAGGCGAA |
| UP00028\_1 | 15 | ATTGCCTGAGGCGAT |
| UP00046\_1 | 17 | ATCCACAGGTGCGAAAA |
| UP00019\_1 | 17 | CTAAGGTTCTAGATCAC |
| UP00031\_1 | 17 | AATCGCACTGCATTCCG |
| UP00047\_1 | 15 | AAGCCCCCCAAAAAT |
| UP00037\_1 | 15 | AACAAACAACAAGAG |
| UP00094\_1 | 17 | TCTTTGGCGTACCCTAA |
| UP00065\_1 | 16 | TGGCGCGCGCGCCTGA |
| UP00082\_1 | 14 | TTATGTACTAATAA |
| UP00021\_1 | 15 | TCCCCCCCCCCCCCC |
| UP00033\_1 | 17 | TATTATGGGATGGATAA |
| UP00095\_1 | 17 | CGAACAGTGCTCACTAT |
| UP00022\_1 | 16 | CCCCCCCCCCCACTTG |
| UP00102\_1 | 14 | CACCCCCGGGGGGG |
| UP00057\_1 | 15 | CCCCCCCGGGGGGGT |
| UP00006\_1 | 15 | CCCCCCCGGGGGGGT |
| UP00026\_1 | 17 | TACATGTGCACATAAAA |
| UP00078\_2 | 15 | ACCCGTATCAAATTT |
| UP00059\_2 | 17 | CGTACAATACGAAATAA |
| UP00099\_2 | 16 | CTATCCCCGCCCTATT |
| UP00020\_2 | 14 | GAATGACGAATAAC |
| UP00012\_2 | 17 | TGATTGTTAACAGTTGG |
| UP00043\_2 | 16 | ATCCCCGCCCCTAAAA |
| UP00050\_2 | 23 | TGTCGTTACACGTGGAAGGCGGT |
| UP00001\_2 | 17 | CGTTCGGCGCCAAAAGG |
| UP00003\_2 | 17 | CGCTCGGCGCCAAAAGC |
| UP00007\_2 | 16 | TGCGGAGTGGGACTGG |
| UP00015\_2 | 16 | TAGTATTTCCGATCTT |
| UP00407\_2 | 17 | GTTCAAAAAAAAAATTC |
| UP00068\_2 | 16 | GCGGAGGTGTCGCCTC |
| UP00079\_2 | 17 | GGCGAGGGGTCAAGGGC |
| UP00073\_2 | 15 | AAAAATAACAAACGG |
| UP00041\_2 | 15 | ATGTCACAACAACAC |
| UP00039\_2 | 17 | AACACCAAAACAAAGGA |
| UP00025\_2 | 15 | CAAACAACAACACCT |
| UP00061\_2 | 16 | ATATCAAAACAAAACA |
| UP00408\_2 | 16 | CCGTCTTCCCCCTCAC |
| UP00032\_2 | 22 | TTTTGTAGATTTTATCGACTTA |
| UP00080\_2 | 17 | GACAGAGATATCAGTTT |
| UP00100\_2 | 17 | GCGGCGATATCGCAGCG |
| UP00070\_2 | 17 | TGCGCATAGGGGAGGAG |
| UP00024\_2 | 14 | AATATTAATAAAGA |
| UP00042\_2 | 16 | AGCGGCACACACGCAA |
| UP00084\_2 | 16 | TGGGCGACGTCGTTAA |
| UP00055\_2 | 17 | TGTTCCCATTGTGTACT |
| UP00035\_2 | 16 | GGGTGTGCCCAAAAGG |
| UP00066\_2 | 16 | TGCAAAAGTCCAATAT |
| UP00391\_2 | 14 | AAAAACCATTAAGG |
| UP00072\_2 | 16 | ATGGAAAGTCGTAAAA |
| UP00086\_2 | 14 | GGAGAAAGGTGCGA |
| UP00018\_2 | 15 | AGTATTCTCGGTTGC |
| UP00040\_2 | 15 | TTGATCGAGAATTCC |
| UP00011\_2 | 15 | ACCACTCTCGGTCAC |
| UP00074\_2 | 14 | GCAAAACATTACTA |
| UP00103\_2 | 16 | ATTGATGAGTCACCAA |
| UP00093\_2 | 17 | AAGCATACGCCCAACTT |
| UP00067\_2 | 16 | GAAGATCAATCACTTA |
| UP00045\_2 | 15 | CAATTGCAAAAATAT |
| UP00044\_2 | 15 | GAAAAAATTGCAAGG |
| UP00060\_2 | 14 | GTGCCACGCGACTG |
| UP00097\_2 | 14 | AAATAAGAAAAAAC |
| UP00092\_2 | 16 | CGACCAACTGCCATGC |
| UP00081\_2 | 15 | CGACCAACTGCCGTG |
| UP00036\_2 | 15 | AGCAACAGCCGCACC |
| UP00017\_2 | 17 | ACTCCAAGTACTTGGAA |
| UP00009\_2 | 16 | CGCGCCGGGTCACGTA |
| UP00027\_2 | 16 | ACATGCTACCTAATAC |
| UP00052\_2 | 16 | ACTTGCTACCTACACC |
| UP00088\_2 | 17 | GCTGGGGGGTACCCCTT |
| UP00048\_2 | 16 | AGAGCGGGGTCAAGTA |
| UP00098\_2 | 23 | ACTGACGCTTGGTTACCACAAAG |
| UP00056\_2 | 15 | TACCCTAGTTACCGA |
| UP00076\_2 | 17 | CTACTTGGATACGGAAT |
| UP00053\_2 | 16 | TCGCGAAGGTTGTACT |
| UP00085\_2 | 14 | CAAATTCCGGAACC |
| UP00008\_2 | 17 | ATGGGATATATCCGCCT |
| UP00000\_2 | 17 | TACGCCCCGCCACTCTG |
| UP00030\_2 | 14 | AAAATTGTTATGAA |
| UP00101\_2 | 16 | AAATAGACAAAGGAAT |
| UP00096\_2 | 17 | GTATTGGGTGGGTATTT |
| UP00004\_2 | 15 | CTCACACAATGGCGC |
| UP00075\_2 | 15 | TTGAATGAAATTCGA |
| UP00014\_2 | 17 | GACCACATTCATACAAT |
| UP00064\_2 | 16 | GGACTGAATTCATGCC |
| UP00069\_2 | 15 | CTATAATTGTTATCG |
| UP00071\_2 | 17 | CATCAATTGTTCCGCTA |
| UP00023\_2 | 16 | TAAGATTATAATACGG |
| UP00062\_2 | 17 | GGAAAAATTGTTAGGAA |
| UP00091\_2 | 15 | TATCATAATTAAGGA |
| UP00034\_2 | 22 | GTGCTAATTGTGTGTGTACGCT |
| UP00051\_2 | 14 | ACATTCATGACACG |
| UP00049\_2 | 15 | TCCGTCGCTTAAAAG |
| UP00002\_2 | 15 | CAAAGGCGTGGCCAG |
| UP00406\_2 | 16 | GATAACATCCTAGTAG |
| UP00077\_2 | 17 | GTTAAAAAAAAAAATTT |
| UP00016\_2 | 17 | TCACGGAACAATAGGTG |
| UP00029\_2 | 15 | CCGATTTAAGCGATC |
| UP00089\_2 | 14 | TTGCCCGGATTAGG |
| UP00058\_2 | 15 | AGCCGAAAAAAAAAT |
| UP00054\_2 | 15 | CCGTATTATAAACAA |
| UP00083\_2 | 16 | GAAGATCAATCACTAA |
| UP00005\_2 | 14 | TCACCTCTGGGCAG |
| UP00010\_2 | 15 | ATTGCCTCAGGCAAT |
| UP00087\_2 | 14 | CCGCCCAAGGGCAG |
| UP00028\_2 | 14 | TACTGGAAAAAAAA |
| UP00046\_2 | 17 | AAGGCCAGATGGTCCGG |
| UP00019\_2 | 15 | TATCATTAGAACGCT |
| UP00031\_2 | 16 | CAATCACTGGCAGAAT |
| UP00047\_2 | 17 | CTTAAGACCACCATTAC |
| UP00037\_2 | 17 | GTGGTTCAATAATTTTG |
| UP00094\_2 | 14 | TGTATATATATACC |
| UP00065\_2 | 14 | GCCGCGCAGTGCGT |
| UP00082\_2 | 16 | GAGCCCTTGTCCCTTG |
| UP00021\_2 | 17 | AGGAGACCCCCAATTTG |
| UP00033\_2 | 17 | TCACCCCGCCCCTAATT |
| UP00095\_2 | 17 | TACGAGACTCCTCTAAC |
| UP00022\_2 | 17 | AAATTCCCCCCGGAAGT |
| UP00102\_2 | 15 | CCACACAGCAGGAGA |
| UP00057\_2 | 15 | CCACACAGCAGGAGA |
| UP00006\_2 | 15 | GAGCACAGCAGGACA |
| UP00026\_2 | 16 | CGAAGCACACAAAATA |
| UP00108\_1 | 17 | TAAACTAATTAGCTGAG |
| UP00187\_1 | 17 | CGCATTAATTAATTACC |
| UP00152\_1 | 17 | GTCCATTAATTAATGGA |
| UP00228\_1 | 17 | CATAACCACTTAACAAC |
| UP00166\_1 | 16 | AACAACCAATTAATTC |
| UP00145\_1 | 16 | AAAAACCAATTAAGAA |
| UP00181\_1 | 16 | AAAGTAATTAGTGAAT |
| UP00151\_1 | 16 | TAAGTAATTAGTTATA |
| UP00138\_1 | 16 | CAGGTAATTACCTCAG |
| UP00209\_1 | 17 | CGAATTAATTAATCACC |
| UP00209\_2 | 17 | CGCATTAATTAATTGGC |
| UP00240\_1 | 16 | TAAGGTAATAAAATTA |
| UP00133\_1 | 16 | AACGGTAATAAAATTT |
| UP00198\_1 | 14 | ATGATCGAATCAAA |
| UP00176\_1 | 16 | CGTTGGGGATTAGCCT |
| UP00219\_1 | 17 | ACCGGTTGATCACCTGA |
| UP00219\_2 | 15 | TAATGATGATCACTA |
| UP00255\_1 | 17 | TAATTAATTAATAATTA |
| UP00218\_1 | 16 | TTTAATTAATTAATTC |
| UP00202\_1 | 14 | CTGAGGTAATTAAT |
| UP00126\_1 | 16 | GGAATAATTACTTCAG |
| UP00154\_1 | 17 | TCGCGATAATTACCGAC |
| UP00110\_1 | 17 | TCGCTATAATTACCGAC |
| UP00230\_1 | 16 | GGGGTAATTAGCTCTG |
| UP00111\_1 | 17 | TGAACCGGATTAATGAA |
| UP00232\_1 | 17 | TAAATAGATACCCCATA |
| UP00143\_1 | 17 | GGAAGGGATTAATTATC |
| UP00227\_1 | 17 | CGACCCAATCAACGGTG |
| UP00201\_1 | 17 | ACCACTAATTAGTGGAC |
| UP00167\_1 | 16 | GCGAACTAATTAATGC |
| UP00163\_1 | 17 | TGCACTAATTAGTGGAA |
| UP00251\_1 | 17 | ATCCATTAATTAATTGA |
| UP00162\_1 | 17 | AGAACTAATTAGTGGAC |
| UP00132\_1 | 17 | CACCGCTAATTAGCGTT |
| UP00204\_1 | 17 | TGCCACTAATTAGTGTA |
| UP00131\_1 | 17 | AGCGCTAATTAGCGATT |
| UP00112\_1 | 17 | AATCGTTAATCCCTTTA |
| UP00127\_1 | 16 | AGGTTAATTAGCTGAT |
| UP00148\_1 | 17 | AAGGCGAAATCATCGCA |
| UP00225\_1 | 15 | CCATAATTAATTACA |
| UP00123\_1 | 16 | GTACTAATTAGTGGCG |
| UP00161\_1 | 17 | GAAAACTAGTTAACATC |
| UP00104\_1 | 17 | ACAAGCAATTAATGAAT |
| UP00155\_1 | 17 | ACAAGCAATTAAAGAAT |
| UP00157\_1 | 17 | ACAAGCAATTAAAGAAT |
| UP00114\_1 | 17 | AAAACATCGTTTTTAAG |
| UP00264\_1 | 16 | CTGAGCTAATTACCGT |
| UP00217\_1 | 16 | TAGGTAATAAAATTCA |
| UP00246\_1 | 16 | TAAAGTCGTAAAACAT |
| UP00183\_1 | 16 | AAAGCTCGTAAAATTT |
| UP00174\_1 | 16 | AAGGTAATTAGCTCAT |
| UP00391\_3 | 14 | TTGAGGTAATTAGT |
| UP00196\_1 | 17 | GATTATTAATTAACTTG |
| UP00189\_1 | 16 | ACGGTAATTAGCTCAG |
| UP00182\_1 | 16 | AAGGTAATTACCTAAT |
| UP00164\_1 | 17 | CGAGTTAATTAATAAGC |
| UP00164\_2 | 16 | GTAGTAATTAATGGAA |
| UP00213\_1 | 17 | ACGGCCATAAAATTAAT |
| UP00134\_1 | 16 | AACCCAATAAAATTCG |
| UP00137\_1 | 17 | TGAGCTAATTAGTTGGA |
| UP00144\_1 | 17 | CGCGTTAATTAATTACC |
| UP00214\_1 | 16 | ACGGTAATTAGCTCAT |
| UP00259\_1 | 16 | TATTGGTAATTACCTT |
| UP00206\_1 | 16 | GTAGTAATTAATGCAA |
| UP00263\_1 | 16 | ACCGGCAATTAATAAA |
| UP00207\_1 | 16 | GGAGCCATAAAATTCG |
| UP00245\_1 | 16 | TAAAGTCGTAAAACGT |
| UP00235\_1 | 16 | TAAAGTCGTAAAATAG |
| UP00135\_1 | 17 | TTAGGTCGTAAAATTTC |
| UP00173\_1 | 16 | AAAGCTCGTAAAATTT |
| UP00113\_1 | 17 | CGAATTAATTAACAATA |
| UP00252\_1 | 17 | CGAATTAATTAATTACT |
| UP00260\_1 | 17 | CAAATTAATTAATAAAA |
| UP00242\_1 | 16 | TTGGGGTAATTAACGT |
| UP00197\_1 | 16 | GGAGGTCATTAATTAT |
| UP00140\_1 | 17 | TAAACTAATTAGCTGTA |
| UP00121\_1 | 17 | AATGCAATAAAATTTAT |
| UP00117\_1 | 17 | TAAGGTCGTAAAATCCT |
| UP00177\_1 | 17 | CAAGGTCGTAAAATCTT |
| UP00180\_1 | 16 | CTACCAATAAAATTCT |
| UP00241\_1 | 16 | TTGAGTTAATTAACCT |
| UP00168\_1 | 17 | TAATTAATTAATGGCTA |
| UP00124\_1 | 16 | AAGGTAATTAGCTCAT |
| UP00236\_1 | 17 | TAAATACATGTAAAATT |
| UP00223\_1 | 17 | AAAATACATGTAATACT |
| UP00223\_2 | 17 | AATATACATGTAATATT |
| UP00194\_1 | 17 | AATATACATGTAAAACA |
| UP00250\_1 | 17 | TATATACATGTAAAATT |
| UP00150\_1 | 17 | AAAATACATGTAAAAAT |
| UP00170\_1 | 16 | CAAAATCAATTAATTT |
| UP00243\_1 | 16 | ACTCCTAATTAGTCGT |
| UP00120\_1 | 17 | TGCATTAATTAATGCGA |
| UP00262\_1 | 17 | CGAATTAATTAATAATG |
| UP00115\_1 | 17 | TAAACTAATTAGTGAAC |
| UP00130\_1 | 17 | GTAATTAATTAAATAAT |
| UP00261\_1 | 17 | TAAACTAATTAGCTTTG |
| UP00212\_1 | 17 | CGAATTAATTAAATACT |
| UP00256\_1 | 17 | GAGCGTTAATTAATGTA |
| UP00256\_2 | 17 | TCCACTAATTAGCGGTT |
| UP00184\_1 | 17 | ACCCCTAATTAGCGGTG |
| UP00175\_1 | 17 | CCCATTAATTAATCACC |
| UP00188\_1 | 17 | CGAATTAATTAAAAACC |
| UP00169\_1 | 17 | AGTTTTTAATTAATTTG |
| UP00186\_1 | 16 | AAGGAGCTGTCAATAC |
| UP00233\_1 | 16 | GAGGTAATTACCTCAG |
| UP00226\_1 | 16 | AAAGACCTGTCAATAC |
| UP00210\_1 | 16 | AATTACCTGTCAATAC |
| UP00234\_1 | 16 | TGCAACTAATTAATTC |
| UP00156\_1 | 17 | GAAGACCAATTAGCGCT |
| UP00171\_1 | 16 | CAAAACCAATTAATTT |
| UP00220\_1 | 17 | TGCGCTAATTAGTGGGA |
| UP00139\_1 | 17 | GTGCACTAATTAGTGCA |
| UP00231\_1 | 17 | TTAACCACTTGAAAATT |
| UP00190\_1 | 16 | CTTTAAGTACTTAATG |
| UP00107\_1 | 16 | TAAGCCACTTGAAATT |
| UP00249\_1 | 16 | TAAGCCACTTGAATTT |
| UP00147\_1 | 16 | TAAGCCACTTAACATT |
| UP00119\_1 | 17 | TTTTAAGTACTTAAATT |
| UP00017\_3 | 17 | TACTAAGTACTTAAATG |
| UP00200\_1 | 17 | GAAAATTAATTACTTCG |
| UP00200\_2 | 16 | AGTAATTAATTACTTC |
| UP00238\_1 | 17 | GATAATTAATTACTTTG |
| UP00216\_1 | 17 | TTAAGGGGATTAACTAC |
| UP00239\_1 | 17 | TGAGGGGGATTAACTAT |
| UP00160\_1 | 17 | TGAGGGGGATTAACTAT |
| UP00208\_1 | 17 | TAGAGGGATTAAATTTC |
| UP00208\_2 | 17 | GATAATTAATCCCTCTT |
| UP00109\_1 | 15 | AAAAACGGATTATTG |
| UP00178\_1 | 17 | CGCGCTAATTAGGTATC |
| UP00237\_1 | 17 | CGTAATTAATTAATTGG |
| UP00229\_1 | 17 | GGAGGGGATTAATTTAT |
| UP00267\_1 | 17 | TGTAGGGATTAATTGTC |
| UP00247\_1 | 17 | TGAACTAATTAGCCCAC |
| UP00224\_1 | 16 | TGATTAATTAATTGAC |
| UP00248\_1 | 17 | CGAACTAATTAGTACTA |
| UP00185\_1 | 17 | TCACCCATCAATAATCA |
| UP00221\_1 | 16 | CAGCATTAATTAGTAG |
| UP00149\_1 | 17 | CGGAATTAATTAATAGG |
| UP00153\_1 | 17 | TTAGAGGGATTAACAAT |
| UP00125\_1 | 17 | TGAAGGGATTAATCATC |
| UP00265\_1 | 16 | AGGGGGATTAGCTGCC |
| UP00203\_1 | 16 | AAAGACCTGTCAATCC |
| UP00205\_1 | 16 | AAGCACCTGTCAATAT |
| UP00158\_1 | 17 | GATTAATTAATTAAGTC |
| UP00254\_1 | 16 | ATGTATTAATTAAGTA |
| UP00191\_1 | 16 | TTGTATGCAAATTAGA |
| UP00179\_1 | 16 | TTGTATGCAAATTAGA |
| UP00129\_1 | 17 | AATTAATTAATTAATTC |
| UP00128\_1 | 17 | GATAATTAATTAGTTTG |
| UP00211\_1 | 17 | AAAATATGCATAATAAA |
| UP00105\_1 | 17 | AATTAATTAATTAATTC |
| UP00118\_1 | 16 | AGTTATTAATGAGGTC |
| UP00146\_1 | 17 | GACGATAATGAGGTTGC |
| UP00146\_2 | 17 | AAACATAATGAGGTTGC |
| UP00172\_1 | 17 | CGAATTAATTAAGAAAC |
| UP00266\_1 | 17 | GTAACTAATTAACTACT |
| UP00136\_1 | 17 | AAAGCTAATTAGCGAAA |
| UP00253\_1 | 17 | TGCACTAATTAGCGCAC |
| UP00193\_1 | 17 | AAGACGCTGTAAAGCGA |
| UP00193\_2 | 17 | AGGACGCTGTAAAGGGA |
| UP00116\_1 | 17 | TGCCTTAATTAATGCTC |
| UP00257\_1 | 17 | CGCGTTAATTAATTGTG |
| UP00192\_1 | 17 | GATGGGGTATCATTTTT |
| UP00159\_1 | 17 | AATGGGGTATCACTTTT |
| UP00195\_1 | 17 | GATAGGGTATCACTTAT |
| UP00199\_1 | 17 | ATAAATGACACCTATCA |
| UP00008\_3 | 17 | AATAGGGTATCAATTAT |
| UP00008\_4 | 17 | AATAGGGTATCAATATT |
| UP00089\_3 | 17 | CCTTAGTTAACTAAAAT |
| UP00222\_1 | 17 | AGCTGTTAACTAGCCGT |
| UP00122\_1 | 17 | GATATTGACAGCTGCGT |
| UP00258\_1 | 16 | AACTAGCTGTCAATAC |
| UP00165\_1 | 16 | TAAGCCACTTGAAATT |
| UP00244\_1 | 17 | TAATTAATTAATAACTT |
| UP00142\_1 | 17 | CATAATTAATTAACGCG |
| UP00215\_1 | 16 | ACGTTAATTAACCCAG |
| UP00106\_1 | 16 | GTGCACTAATTAAGAC |
| UP00141\_1 | 17 | CGAGTTAATTAATAATT |

Random model letter frequencies
(from ./background):
  
A 0.241 C 0.259 G 0.259 T 0.241

---

**SECTION I: HIGH-SCORING MOTIF OCCURRENCES**


---

- There were
  277
  motif occurrences with a
  p-value less than
  0.0001.
- The p-value of a motif occurrence is defined as the
  probability of a random sequence of the same length as the motif
  matching that position of the sequence with as good or better a score.
- The score for the match of a position in a sequence to a motif
  is computed by summing the appropriate entries from each column of
  the position-dependent scoring matrix that represents the motif.
- The q-value of a motif occurrence is defined as the
  false discovery rate if the occurrence is accepted as significant.
- The table is sorted by increasing p-value.

| Motif | Sequence Name | Strand | Start | End | p-value | q-value | Matched Sequence |
| --- | --- | --- | --- | --- | --- | --- | --- |
| UP00018\_1 | chr12 | + | 9692967 | 9692981 | 1.57e-07 | 0.338 | `AGAACCGAAACTAAA` |
| UP00018\_1 | chr19 | + | 16339233 | 16339247 | 3.4e-07 | 0.365 | `TGTACCGAAACCACA` |
| UP00018\_1 | chr8 | − | 126233394 | 126233408 | 9.34e-07 | 0.638 | `AATAACGAAACCAGA` |
| UP00018\_1 | chr12 | + | 67488985 | 67488999 | 1.25e-06 | 0.638 | `CGTTCCGAAACTGCA` |
| UP00018\_1 | chr2 | + | 33554763 | 33554777 | 2.03e-06 | 0.638 | `GAAAACGAAACCAAA` |
| UP00018\_1 | chr4 | + | 185426245 | 185426259 | 2.26e-06 | 0.638 | `CATAGCGAAACCGAA` |
| UP00018\_1 | chr18 | − | 58324637 | 58324651 | 2.54e-06 | 0.638 | `TGAAACGAAACTGAA` |
| UP00018\_1 | chr11 | + | 64375390 | 64375404 | 2.67e-06 | 0.638 | `AGAACCGAAACCAGG` |
| UP00018\_1 | chr15 | − | 43534475 | 43534489 | 2.77e-06 | 0.638 | `AGAAACGAAACCCCA` |
| UP00018\_1 | chr6 | − | 26349040 | 26349054 | 3.09e-06 | 0.638 | `CAAAACGAAACCCAA` |
| UP00018\_1 | chr7 | + | 135301204 | 135301218 | 3.27e-06 | 0.638 | `TGTAACGAAACTGTA` |
| UP00018\_1 | chr16 | − | 11679867 | 11679881 | 4.03e-06 | 0.698 | `AAAACCGAAACTTCA` |
| UP00018\_1 | chr14 | − | 105399308 | 105399322 | 4.22e-06 | 0.698 | `CTGACCGAAACTGAA` |
| UP00018\_1 | chr1 | − | 30992956 | 30992970 | 5.18e-06 | 0.711 | `AGAACTGAAACCAAA` |
| UP00018\_1 | chr9 | + | 36984066 | 36984080 | 5.38e-06 | 0.711 | `GGAACCGAAACCTGA` |
| UP00018\_1 | chr19 | + | 16090573 | 16090587 | 6.25e-06 | 0.711 | `CAGACCGAAACCCCA` |
| UP00018\_1 | chr13 | + | 97939124 | 97939138 | 6.48e-06 | 0.711 | `GAATTCGAAACTGCA` |
| UP00018\_1 | chr4 | + | 2668053 | 2668067 | 6.53e-06 | 0.711 | `AGCTCCGAAACCAAA` |
| UP00018\_1 | chr4 | + | 185426251 | 185426265 | 6.61e-06 | 0.711 | `GAAACCGAAACCTCA` |
| UP00018\_1 | chr5 | + | 138748841 | 138748855 | 6.61e-06 | 0.711 | `ACAAACGAAACTGAA` |
| UP00018\_1 | chr5 | + | 132608048 | 132608062 | 7.72e-06 | 0.734 | `AGTAACGAAACCCAC` |
| UP00018\_1 | chr5 | − | 158208397 | 158208411 | 7.84e-06 | 0.734 | `AATATCGAAACCCTC` |
| UP00018\_1 | chr21 | − | 41719918 | 41719932 | 8.02e-06 | 0.734 | `AGAAACGAAACCTAG` |
| UP00018\_1 | chr2 | − | 38015408 | 38015422 | 9.46e-06 | 0.734 | `AAAATTGAAACTGAA` |
| UP00018\_1 | chr11 | − | 131733030 | 131733044 | 9.91e-06 | 0.734 | `CATCTCGAAACTGAA` |
| UP00018\_1 | chr11 | + | 8660719 | 8660733 | 1.29e-05 | 0.734 | `CTCACCGAAACCGTA` |
| UP00018\_1 | chr19 | − | 16050726 | 16050740 | 1.42e-05 | 0.734 | `CTAAACGAAACCAGC` |
| UP00018\_1 | chr17 | + | 67930067 | 67930081 | 1.43e-05 | 0.734 | `AATAATGAAACTAAA` |
| UP00018\_1 | chr15 | − | 57059255 | 57059269 | 1.55e-05 | 0.734 | `AGAAATGAAACCAGA` |
| UP00018\_1 | chr1 | + | 1700279 | 1700293 | 1.56e-05 | 0.734 | `GGAACCGAAACTTAG` |
| UP00018\_1 | chr11 | − | 72541789 | 72541803 | 1.57e-05 | 0.734 | `AGAATTGAAACCTGA` |
| UP00018\_1 | chr14 | − | 80495693 | 80495707 | 1.57e-05 | 0.734 | `CATAATGAAACTGAA` |
| UP00018\_1 | chr19 | − | 51913562 | 51913576 | 1.58e-05 | 0.734 | `AGAATCGATACTGTA` |
| UP00018\_1 | chr15 | + | 57623308 | 57623322 | 1.61e-05 | 0.734 | `AGAATTGAAACTGAT` |
| UP00018\_1 | chr19 | + | 2429912 | 2429926 | 1.63e-05 | 0.734 | `TAAACCGAAACTGCG` |
| UP00018\_1 | chr5 | + | 156862332 | 156862346 | 1.71e-05 | 0.734 | `AGTTTTGAAACCAAT` |
| UP00018\_1 | chr8 | − | 98712713 | 98712727 | 1.75e-05 | 0.734 | `AAAACTGAAACTGAA` |
| UP00018\_1 | chr17 | − | 23409253 | 23409267 | 1.85e-05 | 0.734 | `CATTACGAAACTATG` |
| UP00018\_1 | chr11 | + | 67790706 | 67790720 | 1.88e-05 | 0.734 | `AGAACTGAAACTGGA` |
| UP00018\_1 | chr19 | + | 9799451 | 9799465 | 1.9e-05 | 0.734 | `GACACCGAAACCGTA` |
| UP00018\_1 | chr15 | + | 39196132 | 39196146 | 1.93e-05 | 0.734 | `TCAATCGAAACCGCC` |
| UP00018\_1 | chr4 | − | 40002395 | 40002409 | 1.94e-05 | 0.734 | `CAGACCGAAACTGTC` |
| UP00018\_1 | chr11 | + | 103305426 | 103305440 | 1.94e-05 | 0.734 | `CTATTTGAAACCAAA` |
| UP00018\_1 | chr19 | + | 17495167 | 17495181 | 2.02e-05 | 0.734 | `GGAGCCGAAACCACA` |
| UP00018\_1 | chr21 | + | 33836810 | 33836824 | 2.02e-05 | 0.734 | `CCTGTCGAAACTGAA` |
| UP00018\_1 | chr2 | + | 213588914 | 213588928 | 2.03e-05 | 0.734 | `CAAAACGAAACCTGG` |
| UP00018\_1 | chr16 | − | 20793471 | 20793485 | 2.12e-05 | 0.734 | `GGAATTGAAACCAAG` |
| UP00018\_1 | chr17 | + | 54091754 | 54091768 | 2.12e-05 | 0.734 | `CGCTTCGAAACTACC` |
| UP00018\_1 | chr18 | − | 58324667 | 58324681 | 2.17e-05 | 0.734 | `AGAACTGAAACTTAA` |
| UP00018\_1 | chr2 | − | 9755694 | 9755708 | 2.21e-05 | 0.734 | `AATATTGAAACCAGG` |
| UP00018\_1 | chr18 | − | 3576825 | 3576839 | 2.21e-05 | 0.734 | `AGAACTGAAACCAAC` |
| UP00018\_1 | chr22 | − | 16119083 | 16119097 | 2.23e-05 | 0.734 | `GGTTCTGAAACCAAA` |
| UP00018\_1 | chr3 | − | 153533330 | 153533344 | 2.26e-05 | 0.734 | `AATACTGAAACCAAG` |
| UP00018\_1 | chr1 | − | 190812422 | 190812436 | 2.37e-05 | 0.734 | `CAAACTGAAACTGTA` |
| UP00018\_1 | chr11 | + | 34083355 | 34083369 | 2.37e-05 | 0.734 | `CCAAGCGAAACCAAT` |
| UP00018\_1 | chr2 | − | 70167494 | 70167508 | 2.43e-05 | 0.734 | `AAGACCGAAACCCAC` |
| UP00018\_1 | chr5 | + | 1369178 | 1369192 | 2.47e-05 | 0.734 | `GATACTGAAACTGAA` |
| UP00018\_1 | chr19 | + | 12754595 | 12754609 | 2.47e-05 | 0.734 | `CGATCCGAAACTTGT` |
| UP00018\_1 | chr11 | − | 82450819 | 82450833 | 2.48e-05 | 0.734 | `CTTTCTGAAACCAAA` |
| UP00018\_1 | chr7 | + | 129903815 | 129903829 | 2.5e-05 | 0.734 | `AATTCTGAAACTAAA` |
| UP00018\_1 | chr6 | − | 27914205 | 27914219 | 2.53e-05 | 0.734 | `TAAAGCGAAACTGTA` |
| UP00018\_1 | chr1 | + | 101473838 | 101473852 | 2.54e-05 | 0.734 | `CCAATTGAAACTGCA` |
| UP00018\_1 | chr19 | − | 60588891 | 60588905 | 2.54e-05 | 0.734 | `GTTTTCGAAACTCTA` |
| UP00018\_1 | chr6 | + | 26303773 | 26303787 | 2.61e-05 | 0.734 | `ATGTCCGAAACTGTA` |
| UP00018\_1 | chr5 | − | 32567375 | 32567389 | 2.65e-05 | 0.734 | `AGTTCTGAAACTAGA` |
| UP00018\_1 | chr8 | − | 126519814 | 126519828 | 2.73e-05 | 0.734 | `ATTTACGAAACCAGG` |
| UP00018\_1 | chr14 | + | 61198369 | 61198383 | 2.78e-05 | 0.734 | `GAAAATGAAACCAAA` |
| UP00018\_1 | chr14 | + | 61198369 | 61198383 | 2.78e-05 | 0.734 | `GAAAATGAAACCAAA` |
| UP00018\_1 | chr6 | − | 86444743 | 86444757 | 2.79e-05 | 0.734 | `TGTATTGAAACTGAG` |
| UP00018\_1 | chr17 | − | 4246406 | 4246420 | 2.81e-05 | 0.734 | `ATGACCGAAACTGGT` |
| UP00018\_1 | chr17 | − | 30724122 | 30724136 | 2.81e-05 | 0.734 | `AATATAGAAACTAAA` |
| UP00018\_1 | chr1 | + | 28374399 | 28374413 | 2.87e-05 | 0.734 | `GAGATCGAAACCCCG` |
| UP00018\_1 | chr4 | + | 182481371 | 182481385 | 2.88e-05 | 0.734 | `AGAAATGAAACCTCA` |
| UP00018\_1 | chr5 | + | 139205238 | 139205252 | 2.92e-05 | 0.734 | `AACTACGAAACCAGA` |
| UP00018\_1 | chr6 | + | 16529481 | 16529495 | 2.93e-05 | 0.734 | `CAAACTGAAACCAAC` |
| UP00018\_1 | chr4 | + | 160318010 | 160318024 | 3.02e-05 | 0.734 | `AGTACTGAAACTATG` |
| UP00018\_1 | chr4 | + | 185632674 | 185632688 | 3.07e-05 | 0.734 | `CAGATCGAAAGCAAA` |
| UP00018\_1 | chr3 | − | 178559044 | 178559058 | 3.12e-05 | 0.734 | `AATTTTGAAACTGGA` |
| UP00018\_1 | chr5 | + | 55474604 | 55474618 | 3.12e-05 | 0.734 | `CTGACTGAAACCACA` |
| UP00018\_1 | chr17 | + | 38800747 | 38800761 | 3.12e-05 | 0.734 | `CATAATGAAACCAAC` |
| UP00018\_1 | chr2 | + | 179103579 | 179103593 | 3.14e-05 | 0.734 | `AATAATGAAACTGGA` |
| UP00018\_1 | chr9 | + | 6402965 | 6402979 | 3.19e-05 | 0.734 | `TCTTCCGAAACCCAA` |
| UP00018\_1 | chr6 | + | 7825573 | 7825587 | 3.22e-05 | 0.734 | `TAACTCGAAACCAGA` |
| UP00018\_1 | chr11 | − | 124446047 | 124446061 | 3.22e-05 | 0.734 | `TATTTCGAAACCTTG` |
| UP00018\_1 | chr3 | − | 23670234 | 23670248 | 3.28e-05 | 0.734 | `GAAACTGAAACTGAA` |
| UP00018\_1 | chr8 | − | 144179332 | 144179346 | 3.28e-05 | 0.734 | `GAAACTGAAACTGAA` |
| UP00018\_1 | chr2 | + | 44812277 | 44812291 | 3.3e-05 | 0.734 | `CAGATTGAAACCCCA` |
| UP00018\_1 | chr12 | − | 120951603 | 120951617 | 3.32e-05 | 0.734 | `CCAGCCGAAACCACA` |
| UP00018\_1 | chr1 | − | 201525925 | 201525939 | 3.34e-05 | 0.734 | `CCTATTGAAACTCAA` |
| UP00018\_1 | chr17 | − | 25715073 | 25715087 | 3.34e-05 | 0.734 | `AAGTTTGAAACCACA` |
| UP00018\_1 | chr7 | − | 100515473 | 100515487 | 3.38e-05 | 0.734 | `AAAACTGAAACTAAT` |
| UP00018\_1 | chr1 | + | 110378569 | 110378583 | 3.39e-05 | 0.734 | `TAAACTGAAACTGAA` |
| UP00018\_1 | chr17 | − | 77091414 | 77091428 | 3.47e-05 | 0.734 | `CAAATCGAAAGTGCA` |
| UP00018\_1 | chr1 | + | 67998425 | 67998439 | 3.48e-05 | 0.734 | `ATTACTGAAACCAAG` |
| UP00018\_1 | chr11 | − | 82450570 | 82450584 | 3.63e-05 | 0.734 | `TAATTCGAAACCTTT` |
| UP00018\_1 | chr12 | − | 123972009 | 123972023 | 3.65e-05 | 0.734 | `ATGACTGAAACCACA` |
| UP00018\_1 | chr20 | − | 3749676 | 3749690 | 3.72e-05 | 0.734 | `AAAATAGAAACTAAA` |
| UP00018\_1 | chr7 | + | 25974814 | 25974828 | 3.74e-05 | 0.734 | `CAAAGTGAAACCACA` |
| UP00018\_1 | chr19 | − | 2561872 | 2561886 | 3.74e-05 | 0.734 | `CCGACTGAAACCAAA` |
| UP00018\_1 | chr6 | − | 12119329 | 12119343 | 3.84e-05 | 0.734 | `TGAACTGAAACCACT` |
| UP00018\_1 | chr6 | + | 106722147 | 106722161 | 3.84e-05 | 0.734 | `AGAAATGAAACCAGT` |
| UP00018\_1 | chr7 | − | 135311814 | 135311828 | 3.86e-05 | 0.734 | `AGTACCGAAAATAGA` |
| UP00018\_1 | chr14 | + | 61287572 | 61287586 | 3.9e-05 | 0.734 | `GGATGCGAAACCCAA` |
| UP00018\_1 | chr5 | − | 96296559 | 96296573 | 3.94e-05 | 0.734 | `GAAACCGAAACTCCC` |
| UP00018\_1 | chr19 | + | 38463351 | 38463365 | 4e-05 | 0.734 | `CAAACTGAAACTCCA` |
| UP00018\_1 | chr10 | + | 64083246 | 64083260 | 4.04e-05 | 0.734 | `ATGAACGAAACCTAG` |
| UP00018\_1 | chr10 | − | 112106457 | 112106471 | 4.04e-05 | 0.734 | `CGAAACGAAACAAAG` |
| UP00018\_1 | chr6 | − | 143310616 | 143310630 | 4.1e-05 | 0.734 | `AAATTTGAAACTGGA` |
| UP00018\_1 | chr7 | + | 26195757 | 26195771 | 4.12e-05 | 0.734 | `ATTATCAAAACCAAA` |
| UP00018\_1 | chr3 | − | 9413461 | 9413475 | 4.15e-05 | 0.734 | `ATGAGCGAAACCACT` |
| UP00018\_1 | chr16 | + | 18720342 | 18720356 | 4.21e-05 | 0.734 | `CCAACCGAAACCCGC` |
| UP00018\_1 | chr10 | + | 121454137 | 121454151 | 4.23e-05 | 0.734 | `GTTTTCGAAACCTCC` |
| UP00018\_1 | chr5 | − | 32622290 | 32622304 | 4.28e-05 | 0.734 | `GATACTGAAACCACT` |
| UP00018\_1 | chr10 | − | 96986157 | 96986171 | 4.28e-05 | 0.734 | `AGAACTGAAACTGTT` |
| UP00018\_1 | chr4 | − | 147239883 | 147239897 | 4.3e-05 | 0.734 | `AGAAAAGAAACCAAA` |
| UP00018\_1 | chr2 | − | 156082276 | 156082290 | 4.35e-05 | 0.734 | `GCTACTGAAACTAAA` |
| UP00018\_1 | chr4 | − | 1684562 | 1684576 | 4.35e-05 | 0.734 | `GCAACCGAAACTAGC` |
| UP00018\_1 | chr11 | − | 71822924 | 71822938 | 4.35e-05 | 0.734 | `CGCTTCGATACCAAA` |
| UP00018\_1 | chr3 | − | 45570227 | 45570241 | 4.37e-05 | 0.734 | `CAATTCGAAAACAAA` |
| UP00018\_1 | chr8 | − | 126689527 | 126689541 | 4.37e-05 | 0.734 | `CATTCTGAAACCCAA` |
| UP00018\_1 | chr18 | − | 22256962 | 22256976 | 4.37e-05 | 0.734 | `GACAACGAAACCAAC` |
| UP00018\_1 | chr11 | − | 65661840 | 65661854 | 4.39e-05 | 0.734 | `GCAACTGAAACCAAA` |
| UP00018\_1 | chr2 | − | 64998735 | 64998749 | 4.41e-05 | 0.734 | `AAATCCGAAACTTCC` |
| UP00018\_1 | chr3 | + | 113662178 | 113662192 | 4.46e-05 | 0.734 | `ATTTTTGAAACTGTA` |
| UP00018\_1 | chr2 | − | 201796654 | 201796668 | 4.48e-05 | 0.734 | `AAAACTGAAACCCTA` |
| UP00018\_1 | chr16 | + | 80402079 | 80402093 | 4.57e-05 | 0.734 | `AGCAGCGAAACTGAT` |
| UP00018\_1 | chr4 | − | 78294458 | 78294472 | 4.6e-05 | 0.734 | `GAGACTGAAACTAAA` |
| UP00018\_1 | chr5 | + | 86448887 | 86448901 | 4.65e-05 | 0.734 | `GGAACTGAAACCATT` |
| UP00018\_1 | chr5 | + | 98391144 | 98391158 | 4.65e-05 | 0.734 | `AAAACTGAAACTCCA` |
| UP00018\_1 | chr5 | + | 123774459 | 123774473 | 4.67e-05 | 0.734 | `CATATCAAAACTGCA` |
| UP00018\_1 | chr22 | − | 39677619 | 39677633 | 4.79e-05 | 0.734 | `CACAGCGAAACCAAC` |
| UP00018\_1 | chr11 | − | 63794231 | 63794245 | 4.81e-05 | 0.734 | `CATCCCGAAACTCCA` |
| UP00018\_1 | chr17 | + | 2116724 | 2116738 | 4.84e-05 | 0.734 | `CATATTGAAACAAAA` |
| UP00018\_1 | chr6 | − | 41782069 | 41782083 | 4.91e-05 | 0.734 | `CAGATCGAAAGCAGA` |
| UP00018\_1 | chr7 | − | 101421163 | 101421177 | 4.91e-05 | 0.734 | `AGAAGTGAAACTAGA` |
| UP00018\_1 | chr7 | − | 101854159 | 101854173 | 4.93e-05 | 0.734 | `AAAACCGAAACACAA` |
| UP00018\_1 | chr2 | − | 74553465 | 74553479 | 4.96e-05 | 0.734 | `TGTGCCGAAACCACC` |
| UP00018\_1 | chr19 | + | 1602459 | 1602473 | 4.96e-05 | 0.734 | `GACTACGAAACCGCA` |
| UP00018\_1 | chr1 | − | 154213109 | 154213123 | 5.01e-05 | 0.734 | `TTTTTTGAAACCGCA` |
| UP00018\_1 | chr2 | − | 157826125 | 157826139 | 5.01e-05 | 0.734 | `AGTATAGAAACTAAG` |
| UP00018\_1 | chr2 | − | 33556999 | 33557013 | 5.03e-05 | 0.734 | `AAATCTGAAACTATA` |
| UP00018\_1 | chr20 | − | 45549674 | 45549688 | 5.03e-05 | 0.734 | `AGAACAGAAACTGAA` |
| UP00018\_1 | chr17 | + | 20542851 | 20542865 | 5.06e-05 | 0.734 | `GAAATAGAAACCAAA` |
| UP00018\_1 | chr6 | − | 26382170 | 26382184 | 5.08e-05 | 0.734 | `AAAAACGAAAACAAA` |
| UP00018\_1 | chr2 | + | 218979533 | 218979547 | 5.11e-05 | 0.734 | `CTTTGCGAAACCGCG` |
| UP00018\_1 | chr5 | + | 138937061 | 138937075 | 5.13e-05 | 0.734 | `AGTACTGAAACTTAC` |
| UP00018\_1 | chr1 | + | 21492753 | 21492767 | 5.21e-05 | 0.734 | `GGTAGTGAAACTGAA` |
| UP00018\_1 | chr5 | + | 131830042 | 131830056 | 5.21e-05 | 0.734 | `AAAACTGAAACCGCC` |
| UP00018\_1 | chr15 | + | 72476917 | 72476931 | 5.23e-05 | 0.734 | `AGAACTGAAACTGGG` |
| UP00018\_1 | chr12 | − | 98251296 | 98251310 | 5.26e-05 | 0.734 | `AGTAATGAAACTGTG` |
| UP00018\_1 | chr15 | + | 62236838 | 62236852 | 5.26e-05 | 0.734 | `AACACCGAAACCCTT` |
| UP00018\_1 | chr7 | + | 120706189 | 120706203 | 5.29e-05 | 0.734 | `AATACTGAAACCTAG` |
| UP00018\_1 | chr17 | − | 1573233 | 1573247 | 5.34e-05 | 0.734 | `GGAATAGAAACCAGA` |
| UP00018\_1 | chr3 | − | 48489496 | 48489510 | 5.37e-05 | 0.734 | `GAAATTGAAACTGAG` |
| UP00018\_1 | chr3 | + | 10209821 | 10209835 | 5.4e-05 | 0.734 | `CATTCCGAAAACAAA` |
| UP00018\_1 | chr1 | − | 118000607 | 118000621 | 5.46e-05 | 0.734 | `GCTTCCGAAACCGCC` |
| UP00018\_1 | chr3 | − | 196746250 | 196746264 | 5.46e-05 | 0.734 | `AAAACTGAAACCAGC` |
| UP00018\_1 | chr6 | + | 21696131 | 21696145 | 5.46e-05 | 0.734 | `AGACCCGAAACCGCC` |
| UP00018\_1 | chr17\_random | − | 86099 | 86113 | 5.49e-05 | 0.734 | `CTTTATGAAACTACA` |
| UP00018\_1 | chr6 | − | 91240443 | 91240457 | 5.51e-05 | 0.734 | `CAAAATGAAACTCCA` |
| UP00018\_1 | chr12 | − | 67487868 | 67487882 | 5.54e-05 | 0.734 | `CAAAAAGAAACCAAA` |
| UP00018\_1 | chr20 | − | 45549639 | 45549653 | 5.54e-05 | 0.734 | `AGAAAAGAAACTAAA` |
| UP00018\_1 | chr5 | − | 96529337 | 96529351 | 5.59e-05 | 0.737 | `AATATTGAAACAAAA` |
| UP00018\_1 | chr10 | − | 64067287 | 64067301 | 5.65e-05 | 0.74 | `GCAACTGAAACTAAA` |
| UP00018\_1 | chr19 | + | 10625771 | 10625785 | 5.79e-05 | 0.745 | `CCTATCGAAAGCGGA` |
| UP00018\_1 | chr20 | − | 47339269 | 47339283 | 5.79e-05 | 0.745 | `CCAAATGAAACTGCA` |
| UP00018\_1 | chr5 | + | 180595007 | 180595021 | 5.9e-05 | 0.745 | `ATGATTGAAACCACG` |
| UP00018\_1 | chr21 | − | 15500935 | 15500949 | 5.9e-05 | 0.745 | `CTAATTGAAACTGTG` |
| UP00018\_1 | chr12 | + | 22588766 | 22588780 | 5.92e-05 | 0.745 | `CGGGGCGAAACTAAA` |
| UP00018\_1 | chr14 | + | 23700277 | 23700291 | 5.92e-05 | 0.745 | `AGAACTGAAACTTAG` |
| UP00018\_1 | chr2 | − | 12786105 | 12786119 | 5.95e-05 | 0.745 | `TGAACTGAAACTGAG` |
| UP00018\_1 | chr1 | + | 85101278 | 85101292 | 6.04e-05 | 0.745 | `TTCTACGAAACCACA` |
| UP00018\_1 | chr21 | − | 15500916 | 15500930 | 6.04e-05 | 0.745 | `CAAATCAAAACTGCA` |
| UP00018\_1 | chr13 | + | 48004006 | 48004020 | 6.13e-05 | 0.745 | `AGTCACGAAACTGAC` |
| UP00018\_1 | chr4 | − | 79779039 | 79779053 | 6.17e-05 | 0.745 | `GGAAATGAAACTCAA` |
| UP00018\_1 | chr5 | + | 95245463 | 95245477 | 6.17e-05 | 0.745 | `AATACTGAAACCCCT` |
| UP00018\_1 | chr1 | + | 228326248 | 228326262 | 6.23e-05 | 0.745 | `GATACAGAAACCAAA` |
| UP00018\_1 | chr1 | − | 110682818 | 110682832 | 6.28e-05 | 0.745 | `GAGTTCGAAACTTCT` |
| UP00018\_1 | chr13 | + | 35518932 | 35518946 | 6.32e-05 | 0.745 | `AAATTTGAAACTCCA` |
| UP00018\_1 | chr6 | − | 26313354 | 26313368 | 6.35e-05 | 0.745 | `TATGCCGAAACCCCA` |
| UP00018\_1 | chr4 | − | 111131664 | 111131678 | 6.47e-05 | 0.745 | `AGAAGTGAAACCTCA` |
| UP00018\_1 | chr1 | − | 42045365 | 42045379 | 6.54e-05 | 0.745 | `AGTTTTGAAACCTGT` |
| UP00018\_1 | chr8 | − | 141547034 | 141547048 | 6.57e-05 | 0.745 | `CGCATCAAAACCAAA` |
| UP00018\_1 | chr15 | + | 88506336 | 88506350 | 6.57e-05 | 0.745 | `GGAAATGAAACTTCA` |
| UP00018\_1 | chr3 | + | 170496573 | 170496587 | 6.66e-05 | 0.745 | `GGAAATGAAACCTGA` |
| UP00018\_1 | chr8 | − | 144179326 | 144179340 | 6.66e-05 | 0.745 | `GAAACTGAAACTCAA` |
| UP00018\_1 | chr17 | + | 30929717 | 30929731 | 6.66e-05 | 0.745 | `GGAAATGAAACCACC` |
| UP00018\_1 | chr1 | + | 233214212 | 233214226 | 6.79e-05 | 0.745 | `AATGTTGAAACCAGA` |
| UP00018\_1 | chr13 | − | 49837959 | 49837973 | 6.79e-05 | 0.745 | `GTTAACGAAACTCTG` |
| UP00018\_1 | chr16 | − | 31099215 | 31099229 | 6.83e-05 | 0.745 | `AGGAGCGAAACAAAA` |
| UP00018\_1 | chr7 | + | 92280967 | 92280981 | 6.86e-05 | 0.745 | `TGTATTGAAACTTCC` |
| UP00018\_1 | chr2 | − | 74051861 | 74051875 | 6.97e-05 | 0.745 | `GGCACTGAAACTGAA` |
| UP00018\_1 | chr1 | + | 154462722 | 154462736 | 7e-05 | 0.745 | `AGTTTTGAAACTCAG` |
| UP00018\_1 | chr6 | − | 106669276 | 106669290 | 7.06e-05 | 0.745 | `CACAATGAAACTGAA` |
| UP00018\_1 | chr2 | + | 98443724 | 98443738 | 7.1e-05 | 0.745 | `AGAAATGAAACCCAG` |
| UP00018\_1 | chr14 | + | 81010314 | 81010328 | 7.1e-05 | 0.745 | `GAAACTGAAACTTCA` |
| UP00018\_1 | chr2 | + | 177572542 | 177572556 | 7.13e-05 | 0.745 | `CTAACAGAAACCACA` |
| UP00018\_1 | chr2 | + | 54650598 | 54650612 | 7.2e-05 | 0.745 | `CCAACTGAAACCATT` |
| UP00018\_1 | chr2 | + | 134757020 | 134757034 | 7.2e-05 | 0.745 | `AAAACCAAAACCGAA` |
| UP00018\_1 | chr6 | + | 174404 | 174418 | 7.2e-05 | 0.745 | `CAAATTGAAACTCCC` |
| UP00018\_1 | chr4 | − | 39906842 | 39906856 | 7.26e-05 | 0.745 | `CCACACGAAACCAGA` |
| UP00018\_1 | chr8 | + | 128291460 | 128291474 | 7.26e-05 | 0.745 | `AAAACAGAAACCAGA` |
| UP00018\_1 | chr17 | − | 73653226 | 73653240 | 7.3e-05 | 0.745 | `CAGAATGAAACCCCA` |
| UP00018\_1 | chr22 | − | 16141337 | 16141351 | 7.3e-05 | 0.745 | `GAAAATGAAACCAAG` |
| UP00018\_1 | chr1 | − | 59207044 | 59207058 | 7.33e-05 | 0.745 | `AGACTTGAAACTGAA` |
| UP00018\_1 | chr5 | + | 60661802 | 60661816 | 7.33e-05 | 0.745 | `AGAAATGAAACCCCT` |
| UP00018\_1 | chr14 | + | 81010308 | 81010322 | 7.41e-05 | 0.745 | `AAAACAGAAACTGAA` |
| UP00018\_1 | chr4 | + | 39906980 | 39906994 | 7.48e-05 | 0.745 | `GGTAACGATACCAGT` |
| UP00018\_1 | chr4 | − | 147239928 | 147239942 | 7.48e-05 | 0.745 | `TAATTTGAAACCAAG` |
| UP00018\_1 | chr4 | + | 40002371 | 40002385 | 7.55e-05 | 0.745 | `AAGTGCGAAACCATT` |
| UP00018\_1 | chr6 | − | 2736695 | 2736709 | 7.58e-05 | 0.745 | `AATACTGAAACTCAG` |
| UP00018\_1 | chr12 | + | 1534011 | 1534025 | 7.58e-05 | 0.745 | `ATAACTGAAACCATC` |
| UP00018\_1 | chr6 | + | 106312514 | 106312528 | 7.62e-05 | 0.745 | `GAAGCCGAAACCAAC` |
| UP00018\_1 | chr1 | + | 66520747 | 66520761 | 7.65e-05 | 0.745 | `AGTAGTGAAACTGAG` |
| UP00018\_1 | chr10 | − | 64085381 | 64085395 | 7.65e-05 | 0.745 | `TGAATTGAAACTCCT` |
| UP00018\_1 | chr17 | − | 72615090 | 72615104 | 7.69e-05 | 0.745 | `TAGTTTGAAACTACA` |
| UP00018\_1 | chr11 | + | 75624175 | 75624189 | 7.76e-05 | 0.745 | `CAAAGCGAAACGAAA` |
| UP00018\_1 | chr2 | − | 33575990 | 33576004 | 7.84e-05 | 0.745 | `GTAACTGAAACCAAG` |
| UP00018\_1 | chr12 | + | 107486109 | 107486123 | 7.84e-05 | 0.745 | `GGTACTGAAACCTTT` |
| UP00018\_1 | chr1 | − | 148806696 | 148806710 | 7.88e-05 | 0.745 | `AAAAACAAAACCAAA` |
| UP00018\_1 | chr2 | − | 98453948 | 98453962 | 7.92e-05 | 0.745 | `GTTTCTGAAACTACA` |
| UP00018\_1 | chr3 | − | 158289298 | 158289312 | 7.92e-05 | 0.745 | `AAAAACGATACCGCT` |
| UP00018\_1 | chr19 | + | 16604104 | 16604118 | 7.92e-05 | 0.745 | `AAAAGTGAAACCAAT` |
| UP00018\_1 | chr1 | − | 172097871 | 172097885 | 7.96e-05 | 0.745 | `ATGAATGAAACTGCA` |
| UP00018\_1 | chr3 | − | 188201286 | 188201300 | 7.96e-05 | 0.745 | `AGAAGTGAAACCTTA` |
| UP00018\_1 | chr12 | − | 91389646 | 91389660 | 8e-05 | 0.745 | `TGTAATGAAACTGTT` |
| UP00018\_1 | chr17 | − | 34871268 | 34871282 | 8e-05 | 0.745 | `TAAACCGAAACGGCA` |
| UP00018\_1 | chr4 | − | 77441720 | 77441734 | 8.04e-05 | 0.745 | `CTAACTGAAACTGTT` |
| UP00018\_1 | chr2 | + | 231498620 | 231498634 | 8.07e-05 | 0.745 | `GGATTTGAAACTGCT` |
| UP00018\_1 | chr16 | − | 51690772 | 51690786 | 8.07e-05 | 0.745 | `CTAAAAGAAACCAAA` |
| UP00018\_1 | chr16 | − | 55297213 | 55297227 | 8.11e-05 | 0.745 | `AAAAAAGAAACTAAA` |
| UP00018\_1 | chr15 | − | 63384244 | 63384258 | 8.15e-05 | 0.745 | `AGAACCGAAAACTTA` |
| UP00018\_1 | chr6 | − | 26382182 | 26382196 | 8.18e-05 | 0.745 | `TAAAGTGAAACTAAA` |
| UP00018\_1 | chr1 | − | 181706195 | 181706209 | 8.22e-05 | 0.745 | `GGAAACGAAAGTACA` |
| UP00018\_1 | chr6 | − | 26070976 | 26070990 | 8.22e-05 | 0.745 | `AGAAGTGAAACTACT` |
| UP00018\_1 | chr7 | − | 135311790 | 135311804 | 8.22e-05 | 0.745 | `AATTCTGAAACTGAG` |
| UP00018\_1 | chr13 | − | 46123443 | 46123457 | 8.22e-05 | 0.745 | `TAAATCAAAACTAAA` |
| UP00018\_1 | chr6 | − | 114059198 | 114059212 | 8.3e-05 | 0.746 | `TGATCTGAAACCTCA` |
| UP00018\_1 | chr7 | + | 101854119 | 101854133 | 8.3e-05 | 0.746 | `CATTTTGAAACCTGT` |
| UP00018\_1 | chr11 | + | 64641744 | 64641758 | 8.34e-05 | 0.746 | `TAGTTCGAAACTTCC` |
| UP00018\_1 | chr2 | − | 8361348 | 8361362 | 8.42e-05 | 0.746 | `GGAACAGAAACCAGA` |
| UP00018\_1 | chr7 | − | 100515479 | 100515493 | 8.46e-05 | 0.746 | `AGAAACAAAACTGAA` |
| UP00018\_1 | chr10 | + | 14742001 | 14742015 | 8.46e-05 | 0.746 | `GGAAATGAAACTTGA` |
| UP00018\_1 | chr16 | − | 11679906 | 11679920 | 8.5e-05 | 0.746 | `AGAAGTGAAACTGAT` |
| UP00018\_1 | chr16 | + | 68938494 | 68938508 | 8.5e-05 | 0.746 | `CCAAGCGAAACCTCC` |
| UP00018\_1 | chr6 | − | 27883916 | 27883930 | 8.62e-05 | 0.747 | `TGAAGTGAAACTAGA` |
| UP00018\_1 | chr10 | + | 69761722 | 69761736 | 8.65e-05 | 0.747 | `CCATGCGAAACCGAC` |
| UP00018\_1 | chr19 | + | 17495125 | 17495139 | 8.65e-05 | 0.747 | `GGAATTGAAACCTGC` |
| UP00018\_1 | chr9 | − | 24922949 | 24922963 | 8.69e-05 | 0.747 | `GAAACTGAAACTATT` |
| UP00018\_1 | chr9 | + | 86499110 | 86499124 | 8.69e-05 | 0.747 | `CAAAAAGAAACTGAA` |
| UP00018\_1 | chr2 | − | 11888026 | 11888040 | 8.74e-05 | 0.748 | `CAAAGTGAAACTTAA` |
| UP00018\_1 | chr6 | − | 91240472 | 91240486 | 8.82e-05 | 0.752 | `CATTTTGAAACTTAC` |
| UP00018\_1 | chr6 | − | 155714577 | 155714591 | 8.86e-05 | 0.752 | `AAAATTGAAACAGAA` |
| UP00018\_1 | chr4 | + | 54309376 | 54309390 | 8.9e-05 | 0.752 | `GGAACTGAAACTGGG` |
| UP00018\_1 | chr12 | − | 31941088 | 31941102 | 8.95e-05 | 0.752 | `ACTACAGAAACCACA` |
| UP00018\_1 | chr1 | + | 42045368 | 42045382 | 8.99e-05 | 0.752 | `GGTTTCAAAACTAAA` |
| UP00018\_1 | chr11 | − | 131733165 | 131733179 | 8.99e-05 | 0.752 | `CCTACCGAAACAATT` |
| UP00018\_1 | chr18 | − | 58977364 | 58977378 | 9.03e-05 | 0.752 | `CATCTTGAAACTAGA` |
| UP00018\_1 | chr1 | − | 101645943 | 101645957 | 9.08e-05 | 0.753 | `TGAACTGAAACCTAC` |
| UP00018\_1 | chr3 | + | 13107978 | 13107992 | 9.16e-05 | 0.757 | `CCTACTGAAACCCAT` |
| UP00018\_1 | chr6 | + | 74287182 | 74287196 | 9.2e-05 | 0.758 | `CTTATCGAAAGCAGC` |
| UP00018\_1 | chr1 | + | 148806909 | 148806923 | 9.24e-05 | 0.758 | `AGCAGTGAAACCGAA` |
| UP00018\_1 | chr8 | − | 126718919 | 126718933 | 9.38e-05 | 0.765 | `CTAATAGAAACCAAG` |
| UP00018\_1 | chr13 | − | 33014792 | 33014806 | 9.42e-05 | 0.765 | `AGAACCGGAACCGAA` |
| UP00018\_1 | chr6 | − | 26379075 | 26379089 | 9.47e-05 | 0.765 | `ATCTTTGAAACTAAA` |
| UP00018\_1 | chr9 | − | 131841924 | 131841938 | 9.47e-05 | 0.765 | `CTTTTAGAAACTACA` |
| UP00018\_1 | chr6 | + | 22106984 | 22106998 | 9.51e-05 | 0.766 | `AGAGCTGAAACTGAA` |
| UP00018\_1 | chr12 | + | 107486776 | 107486790 | 9.6e-05 | 0.767 | `CAAATTGAAACAAGA` |
| UP00018\_1 | chr19 | + | 50270511 | 50270525 | 9.6e-05 | 0.767 | `AGAAGTGAAACCAGG` |
| UP00018\_1 | chr17 | − | 59924398 | 59924412 | 9.77e-05 | 0.771 | `TAAATAGAAACTGCA` |
| UP00018\_1 | chr11 | + | 1832961 | 1832975 | 9.81e-05 | 0.771 | `CAGAATGAAACTGAG` |
| UP00018\_1 | chr15 | + | 65180736 | 65180750 | 9.81e-05 | 0.771 | `GTAAATGAAACTGTA` |
| UP00018\_1 | chr17 | + | 73652306 | 73652320 | 9.81e-05 | 0.771 | `GAAACCGAAAACAGA` |
| UP00018\_1 | chr20 | + | 62053335 | 62053349 | 9.86e-05 | 0.771 | `AAATCTGAAACTCCA` |
| UP00018\_1 | chr11 | + | 68580454 | 68580468 | 9.91e-05 | 0.771 | `GGAACTGAAACTGGC` |
| UP00018\_1 | chr12 | + | 46493175 | 46493189 | 9.91e-05 | 0.771 | `CTAACCGAAACAAGG` |
| UP00018\_1 | chr13 | − | 46123431 | 46123445 | 9.95e-05 | 0.772 | `AAAAACAAAACTAAA` |

---

**DEBUGGING INFORMATION**


---

Command line:

```
/ebi/sw/MEME/VM-cluster410/meme-versions/4.10.0/bin/fimo --parse-genomic-coord --verbosity 1 --oc fimo_out_8 --bgfile ./background --motif UP00018_1 db/uniprobe_mouse.meme ./Supplementary_Table_1.500bp.fa
```

Settings:

```
|  |  |  |
| --- | --- | --- |
| output directory = fimo_out_8 | MEME file name = db/uniprobe_mouse.meme | sequence file name = ./Supplementary_Table_1.500bp.fa |
| background file name = ./background | allow clobber = true | compute q-values = true |
| parse genomic coord. = true | text only = false | scan both strands = true |
| max sequence length = 250000000 | output threshold = 0.0001 | threshold type = p-value |
| max stored scores = 100000 | pseudocount = 0.1 | verbosity = 1 |
| selected motif = UP00018_1 |  |  |
```

This information can be useful in the event you wish to report a
problem with the FIMO software.

---

**Go to top**
