# Supplementary material for: The MEME Suite
Source: Nucleic Acids Res. 2015 May 7;43(Web Server issue):W39–49. doi: 10.1093/nar/gkv416 (PMC4489269; doi:10.1093/nar/gkv416)
Supplement: SUPPLEMENTARY DATA [file supp_gkv416_nar-00283-web-b-2015-File006.pdf]

Published online 2015 *Nucleic Acids Research, 2015, Vol. , No.* **1–2**
doi:10.1093/nar/gkn000

Supplement to "The MEME Suite"

Timothy L. Bailey[1*], James Johnson[1], Charles E. Grant[2] and William S. Noble[2*]

[1]Institute for Molecular Bioscience, The University of Queensland, Brisbane 4072, Queensland, Australia
[2]Department of Genome Sciences, Department of Computer Science and Engineering, University of Washington, Seattle, WA 98109, USA

- Discovery of the huge pentatricopeptide family of plant proteins, which play an essential role in organelle biogenesis (1, 2).
- Discovery and characterization of post-transcriptional RNA regulatory motifs in plant families and of alternative RNA processing in the brain (3, 4).
- Investigation of the genomic expression programs of yeast responding to its environment (5).
- Discovery of transcription factor binding motifs in a wide variety of ChIP-seq data sets (6, 7, 8, 9, 10, 11, 12, 13, 14).
- Mapping of transcriptional circuits that control cell states in hematapoiesis (15).
- Discovery of a group of cyclic peptides in plants (16).
- Profiling protein motifs involved in lipid-protein interactions (17).
- Characterization of a new superfamily of fungal effector proteins (18).
- Exploration of DNA recognition by homeodomain proteins (19)
- Discovery of motifs involved in origins of replication in yeast (20)
- Discovery of a purine motif involved in chromatin silencing at the nuclear lamina (21).

Table 1. Recent applications of the MEME Suite.

REFERENCES

1. I. D. Small and N. Peeters. The PPR motif - a TPR-related motif prevalent in plant organellar proteins. *Trends Biochem Sci*, 25(2):46–47, Feb 2000. [PubMed:http://www.ncbi.nlm.nih.gov/pubmed/10664580].
2. C. Lurin, C. Andrés, S. Aubourg, M. Bellaoui, F. Bitton, C. Bruyère, M. Caboche, C. Debast, J. Gualberto, B. Hoffmann, A. Lecharny, M. L. Ret, M.-L. Martin-Magniette, H. Mireau, N. Peeters, J.-P. Renou, B. Szurek, L. Taconnat, and I. Small. Genome-wide analysis of arabidopsis pentatricopeptide repeat proteins reveals their essential role in organelle biogenesis. *Plant Cell*, 16(8):2089–2103, Aug 2004. [PubMed:http://www.ncbi.nlm.nih.gov/pubmed/15269332] [PubMed Central:http://www.ncbi.nlm.nih.gov/pmc/articles/PMC519200] [doi:http://dx.doi.org/10.1105/tpc.104.022236].
3. J. N. Vaughn, S. R. Ellingson, F. Mignone, and A. von Arnim. Known and novel post-transcriptional regulatory sequences are conserved across plant families. *RNA*, 18(3):368–384, Mar 2012. [PubMed:http://www.ncbi.nlm.nih.gov/pubmed/22237150] [PubMed Central:http://www.ncbi.nlm.nih.gov/pmc/articles/PMC3285926] [doi:http://dx.doi.org/10.1261/rna.031179.111].
4. D. D. Licatalosi, A. Mele, J. J. Fak, J. Ule, M. Kayikci, S. W. Chi, T. A. Clark, A. C. Schweitzer, J. E. Blume, X. Wang, J. C. Darnell, and R. B. Darnell. HITS-CLIP yields genome-wide insights into brain alternative RNA processing. *Nature*, 456(7221):464–469, Nov 2008. [PubMed:http://www.ncbi.nlm.nih.gov/pubmed/18978773] [PubMed Central:http://www.ncbi.nlm.nih.gov/pmc/articles/PMC2597294] [doi:http://dx.doi.org/10.1038/nature07488].
5. A. P. Gasch, P. T. Spellman, C. M. Kao, O. Carmel-Harel, M. B. Eisen, G. Storz, D. Botstein, and P. O. Brown. Genomic expression programs in the response of yeast cells to environmental changes. *Mol Biol Cell*, 11(12):4241–4257, Dec 2000. [PubMed:http://www.ncbi.nlm.nih.gov/pubmed/11102521] [PubMed Central:http://www.ncbi.nlm.nih.gov/pmc/articles/PMC15070].
6. D. S. Johnson, A. Mortazavi, R. M. Myers, and B. Wold. Genome-wide mapping of in vivo protein-DNA interactions. *Science*, 316(5830):1497–1502, Jun 2007. [PubMed:http://www.ncbi.nlm.nih.gov/pubmed/17540862] [doi:http://dx.doi.org/10.1126/science.1141319].
7. R. Jothi, S. Cuddapah, A. Barski, K. Cui, and K. Zhao. Genome-wide identification of in vivo protein-DNA binding sites from ChIP-Seq data. *Nucleic Acids Res*, 36(16):5221–5231, Sep 2008. [PubMed:http://www.ncbi.nlm.nih.gov/pubmed/18684996] [PubMed Central:http://www.ncbi.nlm.nih.gov/pmc/articles/PMC2532738] [doi:http://dx.doi.org/10.1093/nar/gkn488].
8. T. Fujiwara, H. O'Geen, S. Keles, K. Blahnik, A. K. Linnemann, Y.-A. Kang, K. Choi, P. J. Farnham, and E. H. Bresnick. Discovering hematopoietic mechanisms through genome-wide analysis of gata factor chromatin occupancy. *Mol Cell*, 36(4):667–681, Nov 2009. [PubMed:http://www.ncbi.nlm.nih.gov/pubmed/19941826] [PubMed Central:http://www.ncbi.nlm.nih.gov/pmc/articles/PMC2784893] [doi:http://dx.doi.org/10.1016/j.molcel.2009.11.001].
9. F. Vallania, D. Schiavone, S. Dewilde, E. Pupo, S. Garbay, R. Calogero, M. Pontoglio, P. Provero, and V. Poli. Genome-wide discovery of functional transcription factor binding sites by comparative genomics: the case of Stat3. *Proc Natl Acad Sci U S A*, 106(13):5117–5122, Mar 2009. [PubMed:http://www.ncbi.nlm.nih.gov/pubmed/19282476] [PubMed Central:http://www.ncbi.nlm.nih.gov/pmc/articles/PMC2664024] [doi:http://dx.doi.org/10.1073/pnas.0900473106].
10. D. Malhotra, E. Portales-Casamar, A. Singh, S. Srivastava, D. Arenillas, C. Happel, C. Shyr, N. Wakabayashi, T. W. Kensler, W. W. Wasserman, and S. Biswal. Global mapping of binding sites for Nrf2 identifies novel targets in cell survival response through ChIP-Seq profiling and network analysis. *Nucleic Acids Res*, 38(17):5718–5734, Sep 2010. [PubMed:http://www.ncbi.nlm.nih.gov/pubmed/20460467] [PubMed Central:http://www.ncbi.nlm.nih.gov/pmc/articles/PMC2943601] [doi:http://dx.doi.org/10.1093/nar/gkq212].
11. S. V. Ramagopalan, A. Heger, A. J. Berlanga, N. J. Maugeri, M. R. Lincoln, A. Burrell, L. Handunnetthi, A. E. Handel, G. Disanto, S.-M. Orton, C. T. Watson, J. M. Morahan, G. Giovannoni, C. P. Ponting, G. C. Ebers, and J. C. Knight. A ChIP-seq defined genome-wide map of vitamin D receptor binding: associations with disease and evolution. *Genome Res*, 20(10):1352–1360, Oct 2010. [PubMed:http://www.ncbi.nlm.nih.gov/pubmed/20736230] [PubMed Central:http://www.ncbi.nlm.nih.gov/pmc/articles/PMC2945184] [doi:http://dx.doi.org/10.1101/gr.107920.110].

*To whom correspondence should be addressed. Email: t.bailey@uq.edu.au, william-noble@uw.edu

© 2015 The Author(s)
This is an Open Access article distributed under the terms of the Creative Commons Attribution Non-Commercial License (http://creativecommons.org/licenses/by-nc/2.0/uk/) which permits unrestricted non-commercial use, distribution, and reproduction in any medium, provided the original work is properly cited.

12. E. Metzakopian, W. Lin, M. Salmon-Divon, H. Dvinge, E. Andersson, J. Ericson, T. Perlmann, J. A. Whitsett, P. Bertone, and S.-L. Ang. Genome-wide characterization of Foxa2 targets reveals upregulation of floor plate genes and repression of ventrolateral genes in midbrain dopaminergic progenitors. *Development*, 139(14):2625–2634, Jul 2012. [PubMed:http://www.ncbi.nlm.nih.gov/pubmed/22696295] [PubMed Central:http://www.ncbi.nlm.nih.gov/pmc/articles/PMC3383233] [doi:http://dx.doi.org/10.1242/dev.081034].
13. G. Terrados, F. Finkernagel, B. Stielow, D. Sadic, J. Neubert, O. Herdt, M. Krause, M. Scharfe, M. Jarek, and G. Suske. Genome-wide localization and expression profiling establish Sp2 as a sequence-specific transcription factor regulating vitally important genes. *Nucleic Acids Res*, Jun 2012. [PubMed:http://www.ncbi.nlm.nih.gov/pubmed/22684502] [PubMed Central:http://www.ncbi.nlm.nih.gov/pmc/articles/PMC3439921] [doi:http://dx.doi.org/10.1093/nar/gks544].
14. P. D. Anderson, S. A. McKissic, M. Logan, M. Roh, O. E. Franco, J. Wang, I. Doubinskaia, R. van der Meer, S. W. Hayward, C. M. Eischen, I.-E. Eltoum, and S. A. Abdulkadir. Nkx3.1 and Myc crossregulate shared target genes in mouse and human prostate tumorigenesis. *J Clin Invest*, 122(5):1907–1919, May 2012. [PubMed:http://www.ncbi.nlm.nih.gov/pubmed/22484818] [PubMed Central:http://www.ncbi.nlm.nih.gov/pmc/articles/PMC3336973] [doi:http://dx.doi.org/10.1172/JCI58540].
15. N. Novershtern, A. Subramanian, L. N. Lawton, R. H. Mak, W. N. Haining, M. E. McConkey, N. Habib, N. Yosef, C. Y. Chang, T. Shay, G. M. Frampton, A. C. B. Drake, I. Leskov, B. Nilsson, F. Preffer, D. Dombkowski, J. W. Evans, T. Liefeld, J. S. Smutko, J. Chen, N. Friedman, R. A. Young, T. R. Golub, A. Regev, and B. L. Ebert. Densely interconnected transcriptional circuits control cell states in human hematopoiesis. *Cell*, 144(2):296–309, Jan 2011. [PubMed:http://www.ncbi.nlm.nih.gov/pubmed/21241896] [PubMed Central:http://www.ncbi.nlm.nih.gov/pmc/articles/PMC3049864] [doi:http://dx.doi.org/10.1016/j.cell.2011.01.004].
16. J. S. Mylne, L. Y. Chan, A. H. Chanson, N. L. Daly, H. Schaefer, T. L. Bailey, P. Nguyencong, L. Cascales, and D. J. Craik. Cyclic peptides arising by evolutionary parallelism via asparaginyl-endopeptidase-mediated biosynthesis. *Plant Cell*, 24(7):2765–2778, 2012. [PubMed:http://www.ncbi.nlm.nih.gov/pubmed/22822203] [PubMed Central:http://www.ncbi.nlm.nih.gov/pmc/articles/PMC3426113] [doi:http://dx.doi.org/10.1105/tpc.112.099085].
17. K.-Y. Lu, S.-C. Tao, T.-C. Yang, Y.-H. Ho, C.-H. Lee, C.-C. Lin, H.-F. Juan, H.-C. Huang, C.-Y. Yang, M.-S. Chen, Y.-Y. Lin, J.-Y. Lu, H. Zhu, and C.-S. Chen. Profiling lipid-protein interactions using non-quenched fluorescent liposomal nanovesicles and proteome microarrays. *Molecular and Cellular Proteomics*, 2012. [PubMed:http://www.ncbi.nlm.nih.gov/pubmed/22843995] [PubMed Central:http://www.ncbi.nlm.nih.gov/pmc/articles/PMC3494200] [doi:http://dx.doi.org/10.1074/mcp.M112.017426].
18. I. Stergiopoulos, J. Collemare, R. Mehrabi, and P. J. G. M. De Wit. Phytotoxic secondary metabolites and peptides produced by plant pathogenic *Dothideomycete* fungi. *FEMS Microbiology Review*, 2012. [PubMed:http://www.ncbi.nlm.nih.gov/pubmed/22931103] [doi:http://dx.doi.org/10.1111/j.1574-6976.2012.00349.x].
19. S. W. Chu, M. B. Noyes, R. G. Christensen, B. G. Pierce, L. J. Zhu, Z. Weng, G. D. Stormo, and S. A. Wolfe. Exploring the DNA-recognition potential of homeodomains. *Genome Research*, 22(10):1889–1898, 2012. [PubMed:http://www.ncbi.nlm.nih.gov/pubmed/22539651] [PubMed Central:http://www.ncbi.nlm.nih.gov/pmc/articles/PMC3460184] [doi:http://dx.doi.org/10.1101/gr.139014.112].
20. S. C. D. Rienzi, K. C. Lindstrom, T. Mann, W. S. Noble, M. K. Raghuraman, and B. J. Brewer. Maintaining replication origins in the face of genomic change. *Genome Research*, 22(10):1940–1952, 2012. [PubMed:http://www.ncbi.nlm.nih.gov/pubmed/22665441] [PubMed Central:http://www.ncbi.nlm.nih.gov/pmc/articles/PMC3460189] [doi:http://dx.doi.org/10.1101/gr.138248.112].
21. J. M. Zullo, I. A. Demarco, R. Piqué-Regi, D. J. Gaffney, C. B. Epstein, C. J. Spooner, T. R. Luperchio, B. E. Bernstein, J. K. Pritchard, K. L. Reddy, and H. Singh. DNA sequence-dependent compartmentalization and silencing of chromatin at the nuclear lamina. *Cell*, 149(7):1474–1487, 2012. [PubMed:http://www.ncbi.nlm.nih.gov/pubmed/22726435] [doi:http://dx.doi.org/10.1016/j.cell.2012.04.035].
