# Supplementary material for: The MEME Suite
Source: Nucleic Acids Res. 2015 May 7;43(Web Server issue):W39–49. doi: 10.1093/nar/gkv416 (PMC4489269; doi:10.1093/nar/gkv416)
Supplement: SUPPLEMENTARY DATA [file supp_gkv416_nar-00283-web-b-2015-File005.zip › case4/meme-chip/spamo_out_1/spamo.html]

SpaMo - Spaced Motif Analysis


The name of the primary motif.

[
close ]

The logo of the primary motif.

Sections of the motif with a gray background have been trimmed and
were not used for scanning.

[
close ]

The number of secondary motifs found that had significant spacings in
the tested region.

[
close ]

The list of secondary motifs found that had significant spacings in
the tested region.

[
close ]

The name of the sequence database.

[
close ]

The last modified date of the sequence database.

[
close ]

The number of sequences in the sequence database.

[
close ]

The number of sequences in the sequence database which were excluded
because they were shorter than twice the margin plus the primary motif
length.

[
close ]

The number of sequences in the sequence database which were excluded
because no match to the primary motif could be found at a distance to
the edges larger than the margin.

[
close ]

The number of sequences in the sequence database which were excluded
because they were largly identical to other sequences when aligned on
the primary motif site.

[
close ]

The number of sequences which were scanned with the secondary
motifs.

[
close ]

The name of the motif database derived from the file name.

[
close ]

The date that the motif database was last modified.

[
close ]

The number of motifs loaded from the motif database. Some motifs may
have been excluded.

[
close ]

The number of motifs with significant *E*-values whose
significant spacings were not considered too similar to those of another motif.

[
close ]

The number of motifs that while having significant spacings were less
signficant than another motif that matched
most of the same sites.

[
close ]

The primary motif is used as the reference point for all spacing
calculation.

Sections of the motif with a gray background have been trimmed and
were not used for scanning.

[
close ]

The secondary motif occurs at the spacings relative to the primary
shown in the histogram below.

Sections of the motif with a gray background have been trimmed and
were not used for scanning.

[
close ]

The *E*-value is the lowest *p*-value of any spacing of the
secondary motif times the number of secondary motifs.
It estimates the expected number of *random* secondary motifs that
would have the observed minimum *p*-value or less.

[
close ]

The histogram below shows the frequency of spacings from the primary
motif to the secondary motif. Red bars
indicate that a spacing has occured a statistically significant number
of times.

**Upstream**
:   These are sequences where the secondary motif occurs before the
    primary motif.

**Downstream**
:   These are sequences where the secondary motif occurs after the
    primary motif.

**Same Strand**
:   These are sequences where the secondary motif is on the same strand
    as the primary motif.

**Opposite Strand**
:   These are sequences where the secondary motif is on the opposite
    strand to the primary motif.

[
close ]

The details of the significant spacings are shown in the four
quadrants as they relate to the quadrants of the graph.

**P-value**
:   is the probability of the observed number (or more) sequences
    having the observed spacing between the primary and secondary motif,
    adjusted for multiple tests. The number of multiple tests is the
    number of spacing bins (the number of bars in the histogram)
    tested for significance.

**Gap**
:   is the space between the primary and secondary motifs where a value
    of zero means there is no space between them.

**#**
:   is the number of sequences where that spacing was observed.

[
close ]

The total number of sequences that have a match for both the primary
motif and this secondary motif.

[
close ]

The motif database which this secondary motif came from.

[
close ]

The list of secondary motifs which have been identified as possibly
similar to this motif because their most significant spacings
overlaped with the most significant spacing of this motif. Check the
boxes to show the possibly similar secondary motifs.

[
close ]

This shows the first secondary motif aligned with the current
secondary motif by the most significant spacing.

Sections of the motif with a gray background have been trimmed and
were not used for scanning.

[
close ]

# SpaMo

## Spaced Motif Analysis Tool

For further information on how to interpret these results or to get a
copy of the MEME software please access
http://meme.nbcr.net.

If you use SpaMo in your research please cite the following paper:  
Tom Whitington, Martin C. Frith, James Johnson and Timothy L. Bailey,
"Inferring transcription factor complexes from ChIP-seq data",
*Nucleic Acids Research*, **39**(15):e98, 2011.
[full text]

Primary Motif  |  Sequence Database  |  Secondary Databases  |  Spacing Analysis  |  Program information

|  |  |
| --- | --- |
| Primary Motif | Next Top |

| Name | Preview | Significant Secondaries | List |
| --- | --- | --- | --- |
| 1 (MEME) | AGGTCACAGAGGTCA | 3 | 2 (MEME),  MA0046.1 (HNF1A),  UP00232 1 (Dobox4 3956.2) |

|  |  |
| --- | --- |
| Sequence Database | Previous Next Top |

| Name | Last Modified | Loaded | Too Short | No Primary | Too Similar | Used |
| --- | --- | --- | --- | --- | --- | --- |
| Supplementary Table 1.500bp | Sat Jan 24 06:59:23 2015 | 2776 | 0 | 2142 | 80 | 554 |

|  |  |
| --- | --- |
| Secondary Databases | Previous Next Top |

| Name | Last Modified | Number of Motifs | Motifs Significant | Motifs Redundant |
| --- | --- | --- | --- | --- |
| meme.xml | Sat Jan 24 07:19:52 2015 | 2 | 1 | 0 |
| dreme.xml | Sat Jan 24 07:23:27 2015 | 11 | 0 | 0 |
| JASPAR CORE 2014 vertebrates | Wed Nov 26 13:17:40 2014 | 205 | 1 | 0 |
| uniprobe mouse | Wed Nov 26 13:17:43 2014 | 386 | 1 | 0 |

|  |  |
| --- | --- |
| Spacings of "2 (MEME)" relative to "1 (MEME)" | Previous Next Top |

| Primary: 1 (MEME) | Secondary: 2 (MEME) | *E*-value |
| --- | --- | --- |
| AGGTCACAGAGGTCA | AAAAAAATAAGTTTTTTTAAA | 6.6 |

| Motif Spacing Histogram | |  | Significant Motif Spacings (*p*<0.05) | |  |
| --- | --- | --- | --- | --- | --- |
| Upstream | Downstream |  | Upstream | Downstream | Other Details |
|  | | Same Strand  Opposite Strand | P-value \| Gap \| # \|; 0.011 \| 116 \| 4 \| | | Total sequences with primary and secondary motif 82  Motif Database meme.xml |

|  |  |
| --- | --- |
| Spacings of "MA0046.1 (HNF1A)" relative to "1 (MEME)" | Previous Next Top |

| Primary: 1 (MEME) | Secondary: MA0046.1 (HNF1A) | *E*-value |
| --- | --- | --- |
| AGGTCACAGAGGTCA | GGTTAATAATTACC | 9.3 |

| Motif Spacing Histogram | |  | Significant Motif Spacings (*p*<0.05) | |  |
| --- | --- | --- | --- | --- | --- |
| Upstream | Downstream |  | Upstream | Downstream | Other Details |
|  | | Same Strand  Opposite Strand | P-value \| Gap \| # \|; 0.015 \| 130 \| 4 \| | | Total sequences with primary and secondary motif 93  Motif Database JASPAR CORE 2014 vertebrates |

|  |  |
| --- | --- |
| Spacings of "UP00232 1 (Dobox4 3956.2)" relative to "1 (MEME)" | Previous Next Top |

| Primary: 1 (MEME) | Secondary: UP00232 1 (Dobox4 3956.2) | *E*-value |
| --- | --- | --- |
| AGGTCACAGAGGTCA | TAAATAGATACCCCATA | 9.9 |

| Motif Spacing Histogram | |  | Significant Motif Spacings (*p*<0.05) | |  |
| --- | --- | --- | --- | --- | --- |
| Upstream | Downstream |  | Upstream | Downstream | Other Details |
|  | | Same Strand  Opposite Strand | P-value \| Gap \| # \|; 0.016 \| 113 \| 4 \| | | Total sequences with primary and secondary motif 95  Motif Database uniprobe mouse |

Previous Top

##### SpaMo version

4.10.0 (Release date: Wed May 21 10:35:36 2014 +1000)

##### Reference

Tom Whitington, Martin C. Frith, James Johnson and Timothy L. Bailey,
"Inferring transcription factor complexes from ChIP-seq data",
*Nucleic Acids Research*, **39**(15):e98, 2011.

##### Command line summary

spamo -verbosity 1 -oc spamo\_out\_1 -bgfile ./background -primary 1 ./Supplementary\_Table\_1.500bp.fa meme\_out/meme.xml meme\_out/meme.xml dreme\_out/dreme.xml db/JASPAR\_CORE\_2014\_vertebrates.meme db/uniprobe\_mouse.meme  
Result calculation took 28 seconds  
Note that the random number generator was initilized with a seed of 1 so you need
"-numgen 1" in the list of arguments
to replicate the experiment.

show model parameters...

##### Model parameters

seed = 1
margin = 150
bin\_size = 1
bin\_pvalue\_calc\_range = 150
bin\_pvalue\_cutoff = 0.05
motif\_evalue\_cutoff = 10
seq\_max\_shared\_fract = 0.5
seq\_min\_hit\_score = 7
redundant\_overlap = 2
redundant\_joint = 0.5
motif\_pseudocount = 0.1
motif\_trim = 0.25
bin\_max = 4
host = b03b32
when = Sat Jan 24 07:24:37 2015

hide model parameters...
